# Genomic and transcriptomic heterogeneity in metaplastic carcinomas of the breast

**DOI:** 10.1038/s41523-017-0048-0

**Published:** 2017-12-01

**Authors:** Salvatore Piscuoglio, Charlotte K. Y. Ng, Felipe C. Geyer, Kathleen A. Burke, Catherine F. Cowell, Luciano G. Martelotto, Rachael Natrajan, Tatiana Popova, Christopher A. Maher, Raymond S. Lim, Ino de Bruijn, Odette Mariani, Larry Norton, Anne Vincent-Salomon, Britta Weigelt, Jorge S. Reis-Filho

**Affiliations:** 1 Department of Pathology, Memorial Sloan Kettering Cancer Center, New York, NY USA; 2 Institute of Pathology, University Hospital Basel, Basel, Switzerland; 3 Department of Biomedicine, University of Basel, Basel, Switzerland; 4 Department of Pathology, Hospital Israelita Albert Einstein, Instituto Israelita de Ensino e Pesquisa, São Paulo, Brazil; 5 The Breakthrough Breast Cancer Research Centre, The Institute of Cancer Research, London, UK; 6 INSERM U934, Institut Curie, Paris, France; 7 McDonnell Genome Institute, Washington University School of Medicine, St. Louis, MO USA; 8 Department of Internal Medicine, Washington University School of Medicine, St. Louis, MO USA; 9 Alvin J. Siteman Cancer Center, Washington University School of Medicine, St. Louis, MO USA; 10 Department of Biomedical Engineering, Washington University School of Medicine, St. Louis, MO USA; 11 Department of Medicine, Memorial Sloan Kettering Cancer Center, New York, NY USA; 12 Department of Tumor Biology, Institut Curie, Paris, France; 13 Human Oncology and Pathogenesis Program, Memorial Sloan Kettering Cancer Center, New York, NY USA

## Abstract

Metaplastic breast cancer (MBC) is a rare special histologic type of triple-negative breast cancer, characterized by the presence of neoplastic cells showing differentiation towards squamous epithelium and/or mesenchymal elements. Here we sought to define whether histologically distinct subgroups of MBCs would be underpinned by distinct genomic and/or transcriptomic alterations. Microarray-based copy number profiling identified limited but significant differences between the distinct MBC subtypes studied here, despite the limited sample size (*n* = 17). In particular, we found that, compared to MBCs with chondroid or squamous cell metaplasia, MBCs with spindle cell differentiation less frequently harbored gain of 7q11.22-23 encompassing *CLDN3* and *CLDN4*, consistent with their lower expression of claudins and their association with the claudin-low molecular classification. Microarray-based and RNA-sequencing-based gene expression profiling revealed that MBCs with spindle cell differentiation differ from MBCs with chondroid or squamous cell metaplasia on the expression of epithelial-to-mesenchymal transition-related genes, including down-regulation of *CDH1* and *EPCAM*. In addition, RNA-sequencing revealed that the histologic patterns observed in MBCs are unlikely to be underpinned by a highly recurrent expressed fusion gene or a pathognomonic expressed mutation in cancer genes. Loss of PTEN expression or mutations affecting *PIK3CA* or *TSC2* observed in 8/17 MBCs support the contention that PI3K pathway activation plays a role in the development of MBCs. Our data demonstrate that despite harboring largely similar patterns of gene copy number alterations, MBCs with spindle cell, chondroid and squamous differentiation are distinct at the transcriptomic level but are unlikely to be defined by specific pathognomonic genetic alterations.

## Genetics: no common genetic basis found for metaplastic breast carcinoma

Recurrent somatic genetic alterations in any known cancer gene are unlikely to underpin metaplastic breast carcinoma (MBC), a rare breast cancer type. Jorge Reis-Filho from Memorial Sloan Kettering Cancer Center in New York, NY, USA, and colleagues performed complete genomic work-ups of 17 MBCs, an invasive form of breast cancer characterized by tumor cells displaying differentiation towards other cell types. Albeit limited, important differences in gene copy number and gene expression profiles between different subtypes of MBC were observed. These tumors were incredibly variable from patient to patient, and there were no defining genomic features that could demarcate the disease on a genetic level from other forms of triple-negative breast cancer. The authors suggest that further studies are needed to explain the basis of the diversity seen in MBCs.

## Introduction

Metaplastic breast carcinoma (MBC) is a rare special histologic type of breast cancer, accounting for 0.2–5% of invasive breast cancers.^1,2^ MBCs comprise a heterogeneous group of tumors characterized by the presence of malignant epithelial cells showing differentiation towards squamous epithelium and/or mesenchymal elements, such as spindle, chondroid, osseous or rhabdoid cells.^1,2^ Most MBCs display a triple-negative (TN) phenotype (i.e., lack of expression of estrogen receptor (ER), progesterone receptor (PR), and HER2), and are classified as basal-like or claudin-low molecular subtypes.^3–5^ Akin to the basal-like subtype, the claudin-low subtype is enriched for triple-negative breast cancers (TNBCs), but is further characterized by a gene expression profile associated with epithelial-to-mesenchymal transition (EMT) and cancer stem cells. MBCs have an aggressive clinical behavior, and unlike other forms of TNBCs, these tumors seem not to respond to conventional chemotherapy regimens.^6^

Despite their distinctive histologic and clinical features, relatively few molecular alterations that discriminate MBCs from histologic grade- and ER-matched invasive ductal carcinomas of no special type (IDC-NSTs) have been found. Overall, MBCs and histologic grade-matched and ER-matched IDC-NSTs display similar transcriptomic and copy number profiles.^4,7^ Akin to TN IDC-NSTs, MBCs also frequently harbor *TP53* somatic mutations,^8,9^ loss of *CDKN2A*, and *EGFR* overexpression and amplification.^10^ Compared to TN IDC-NSTs, MBCs frequently display an EMT-like gene signature, display downregulation of DNA damage response pathways,^4^ and harbor frequent genetic alterations affecting the PI3K and Wnt pathways.^3,9,11^ Importantly, however, different histologic subtypes of or components within MBCs are associated with specific transcriptomic subtypes,^5^ and may be underpinned by distinct copy number alterations (CNAs)^8^ and mutational profiles.^9^ These data provided evidence to suggest that stratification of MBCs according to their histologic subtypes may be required to identify molecular alterations and targets specific for this disease.

There is burgeoning evidence to suggest that some special histologic subtypes of TNBC may be driven by recurrent fusion genes. For example, secretory carcinomas are characterized by the *ETV6-NTRK3* fusion gene,^12^ whereas adenoid cystic carcinomas are underpinned the *MYB-NFIB* fusion gene^13^ or rearrangements of the *MYBL1* gene.^14^ Genomic analyses investigating the presence of fusion genes in MBCs have yet to be described.

Given that MBCs of different histologic subtypes are likely to have distinct molecular features, here we sought to define whether histologically distinct subgroups of MBCs would be underpinned by distinct copy number or gene expression profiles. Furthermore, we sought to determine whether MBCs, akin to other special histologic types of TNBC, would be underpinned by a recurrent fusion gene. To this end, MBCs of distinct histologic subtypes (spindle cell, squamous, and chondroid) were subjected to array-based gene expression, gene copy number profiling, and RNA-sequencing.

## Results

### Histopathologic and immunophenotypic characterization of MBCs

**Fig. 1:**
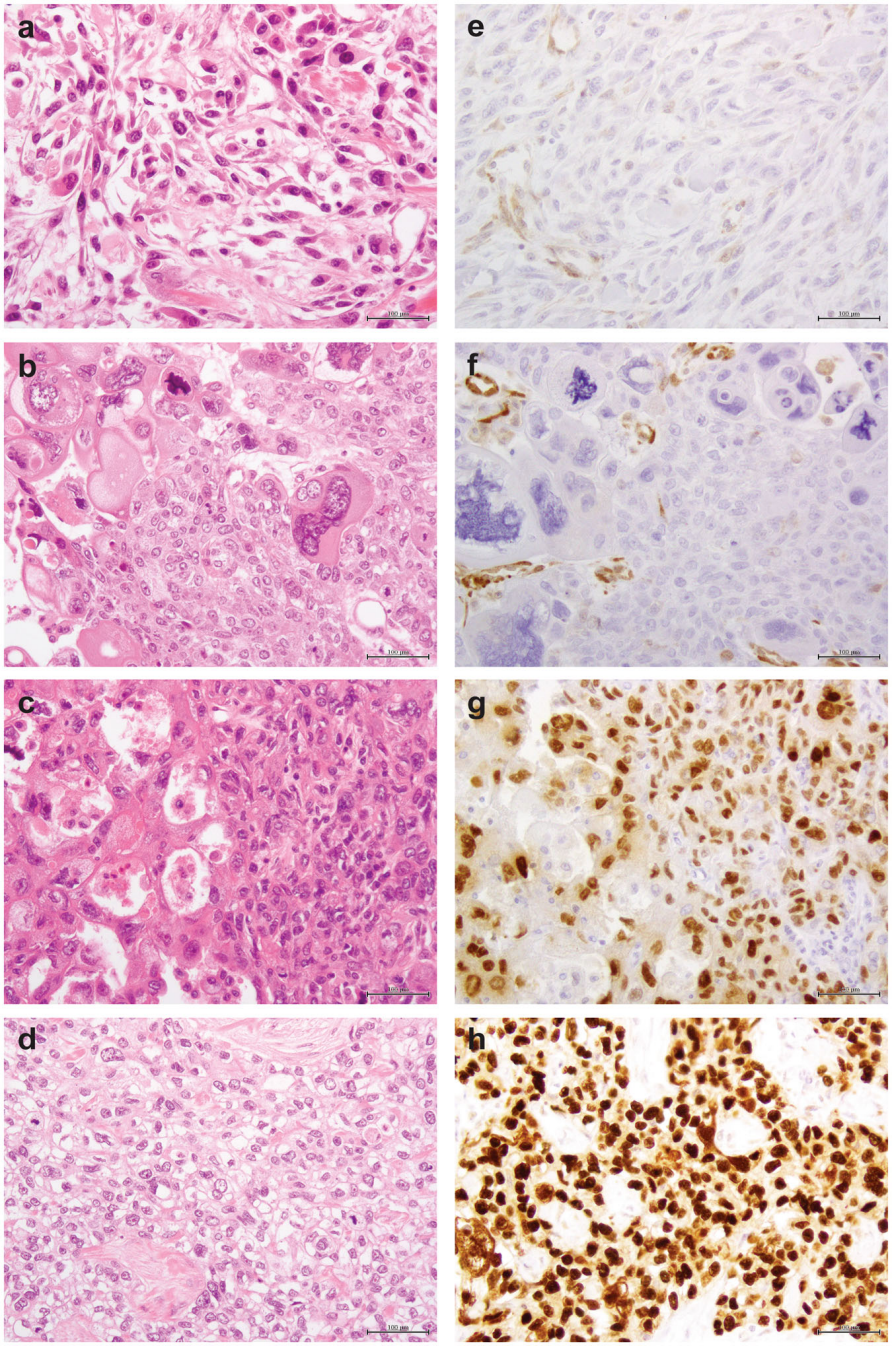
Immunohistochemical analysis of p53 and PTEN in MBCs. Representative micrographs of metaplastic breast carcinomas (MBCs), **a** with spindle cell (META32) and **b-d** with squamous metaplasia (META41, META37, and META42, respectively). Immunohistochemical analysis showed loss of PTEN expression in **e** META32 and **f** META41 and p53 overexpression in **g** META37 and **h** META42. In **e** and **f**, note the positive internal control for PTEN in blood vessels. Scale bars, 100 µm

Of the 17 MBCs included in this study (Supplementary Fig. 1), seven (41%), five (29.5%) and five (29.5%) cases were classified as chondroid, squamous or spindle cell subtypes, respectively, and the vast majority were of high histologic grade (15/17, 88% grade 3; 2/17, 12% grade 2). Immunophenotypic assessment revealed that all MBCs, irrespective of histologic subtype, were of TN phenotype and expressed at least one basal marker, either high molecular weight cytokeratins (CKs), including CK14, CK17, CK5/6, or EGFR^10^ (Supplementary Tables 1 and 2). Additionally, seven (41%) MBCs showed strong overexpression of p53, a surrogate marker for the presence of *TP53* missense mutations,^15^ and four cases lacked PTEN expression by immunohistochemistry (Fig. 1 and Supplementary Table 1).

### Histologic subtypes of MBCs harbor similar patterns of gene copy number alterations

Compared to a set of TN IDC-NSTs^16^ matched in a 2:1 ratio with MBCs, both MBCs and TN IDC-NSTs exhibited similar patterns of gene CNAs, with frequent gains of 1q and 8q, and losses of 5q and 12q (Supplementary Fig. 2 and Supplementary Table 3). In the MBCs analyzed, homozygous deletions of *PTEN* (10q23) were found in two cases (META32 and META41; Supplementary Table 4), and were associated with loss of PTEN protein expression as assessed by immunohistochemistry (Fig. 1). Additional two cases harbored homozygous deletions of the *CDKN2A/CDKN2B* and *MTAP* gene loci (9p21, META42, and META49; Supplementary Table 4). Recurrent focal amplification of *ZNF703/FGFR1/BRF2* (8p11.23-11.22) was found in two cases (META31 and META39). Additional recurrent focal amplifications included those affecting *WNT9A/WNT3A* (1q42.12-42.2, META41, and META52) and *TNIK* (3q26.2-26.31, META39 and META42), a Wnt target gene activator.^17^

**Fig. 2:**
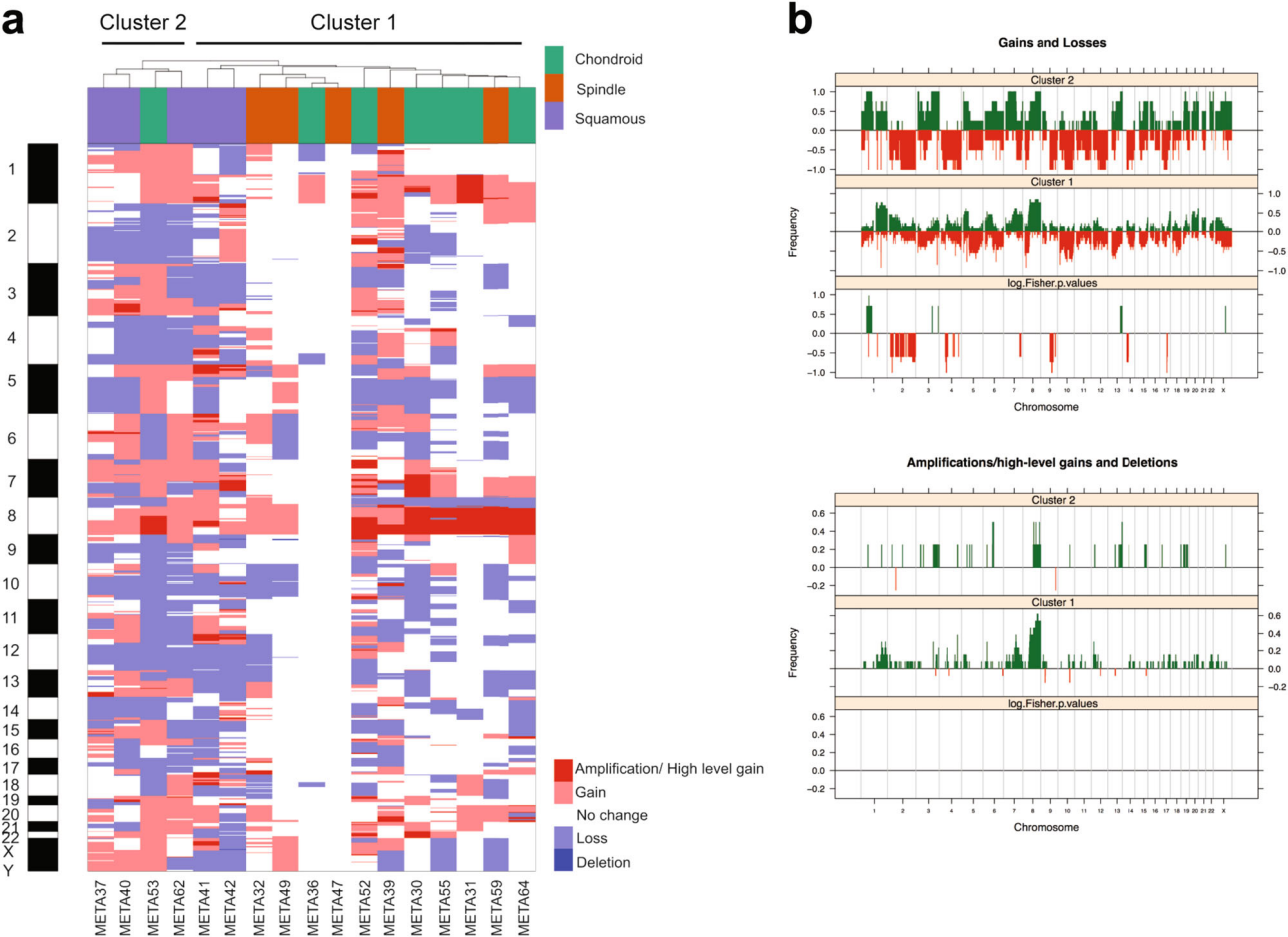
Landscape of gene copy number alterations in MBCs. **a** Hierarchical cluster analysis performed with SNP 6.0 defined copy number alterations (i.e., gains, losses, amplifications/high-level gains and deletions) using Euclidean distance metric and Ward’s algorithm. Histologic subtypes of MBCs are color-coded according to the legend. **b** Frequency plot of (top) copy number gains and losses and (bottom) amplifications/high-level gains and deletions in copy number cluster 1 and copy number cluster 2 identified by hierarchical clustering. The proportion of tumors in which each probe is gained/ amplified (green bars) or lost/ deleted (red bars) is plotted (*y*-axis) for each probe according to its genomic position (*x*-axis). Inverse Log_10_ values of Fisher’s exact *p* value are plotted according to genomic position (*x*-axis) at the bottom of each graph

Given that MBCs of distinct histologic subtypes have been reported to display different gene expression profiles^5^ and mutational repertoires^9^, we performed an exploratory analysis to define whether the histologic diversity would be underpinned by distinct patterns of gene CNAs. Hierarchical clustering of the categorical CNA states revealed that the three MBC histologic subtypes did not form distinct clusters (Fig. 2a). We observed, however, two stable clusters, one composed predominantly of MBCs with squamous differentiation (cluster 2, consisting of META37, META40, META53, and META62), and the other composed mainly of MBCs with mesenchymal differentiation (chondroid or spindle cell differentiation; cluster 1, consisting of all other samples), with a significant enrichment of MBCs with squamous differentiation in cluster 2 (*p*-value = 0.004, Fisher’s exact test). The clusters differed predominantly by gains of 1p and losses of chromosome 2 and 9q (Fig. 2b, Supplementary Table 5). Indeed, direct comparisons between the distinct histologic MBC subtypes revealed that MBCs with squamous differentiation had higher fractions of the genome altered compared to non-squamous MBCs (*p*-value = 0.02, Mann–Whitney *U* test, Supplementary Fig. 3), but few CNAs were significantly different between them. MBCs with chondroid differentiation were characterized by 21q11.2 gains and high level gain/ amplification of 8q21.11-24.3 compared to non-chondroid MBCs (both *p*-value < 0.05, Fisher’s exact tests, Supplementary Fig. 3a, Supplementary Table 6). MBCs with spindle cells harbored similar frequencies of gene CNAs as MBCs with squamous or chondroid differentiation, apart from the less frequently observed 7q11.22-23 gain encompassing *CLDN3* and *CLDN4* (*p*-value < 0.05, Fisher’s exact test, Supplementary Fig. 3b, Supplementary Table 7 *)*. Compared to MBCs with spindle cell or chondroid differentiation, MBCs with squamous differentiation infrequently harbored amplification/ high-level gain of 8q24, but harbored more frequent losses on 7q and 12q and gains on 11p and 12q (all *p*-value < 0.05, Fisher’s exact tests, Supplementary Fig. 3c–d, Supplementary Table 8). Furthermore, we noticed that two cases harbored very few or no CNAs (META36 and META47). The tumor cell content for META36, a chondroid MBC, was estimated to be 87 and 80% by ABSOLUTE and pathology review, respectively, whereas META47, a spindle cell MBC, did not harbor any CNA and its tumor cell content was estimated to be 70% by pathology review but could not be estimated by ABSOLUTE (Supplementary Table 1). It should be noted, however, that META47 was classified as claudin-low, as opposed to normal breast-like, based on intrinsic subtyping.^5^ These analyses support the contention that the lack of CNAs was not a result of insufficient tumor cell content. In fact, gene copy number analysis of the samples of the METABRIC study has revealed the existence of a subset of TNBCs lacking CNAs despite the adequate tumor cell content.^18^

Taken together, MBCs seem not to be underpinned by a pathognomonic CNA. In fact, these tumors display CNAs similar to those observed in conventional forms of high-grade TNBCs, and, regardless of the histologic subtype, MBCs harbor similar CNA profiles. Significant differences in the CNAs according to subtype were identified, however these are unlikely to account for the histologic diversity seen in these cancers. Further studies, with a larger sample size, are however required to refine the boundaries of the regions differentially gained or lost in the histologically distinct subtypes of MBC.

### MBCs with spindle cell differentiation show distinct transcriptomic profiles

**Fig. 3:**
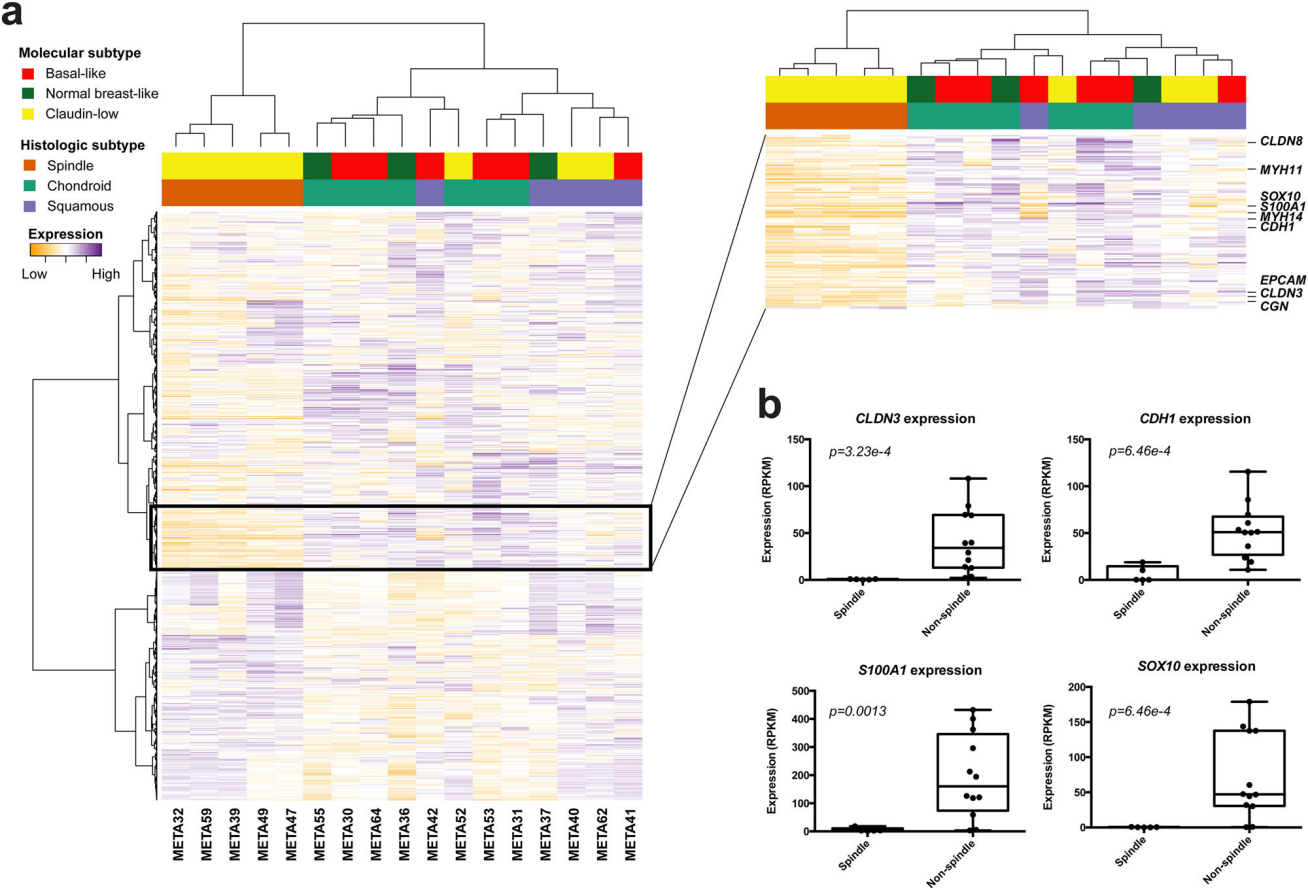
MBCs of spindle subtype have a unique transcriptomic profile amongst MBCs. **a** Unsupervised hierarchical clustering of MBCs based on gene expression arrays using Pearson’s correlation and Ward’s algorithm. Histologic subtypes of MBCs are color-coded according to the legend. Zoomed-in heatmap on the right shows genes differentially expressed between MBCs with spindle cells as compared to chondroid and squamous MBCs, including tight-junction related genes, such as *CLDN3*, and *MYH11*, and EMT-related genes, such as *CDH1* and *EPCAM*. **b** Validation of significantly differentially expressed genes between spindle and non-spindle MBCs identified by microarray-based gene expression analysis was performed using RNA-sequencing data. For each gene, normalized expression value is plotted for each MBC, grouped by histologic subtype. *p*-values were calculated from the differential expression analysis using limma^46^ (see Methods)

Consistent with our previous observations,^5^ using the PAM50 and claudin-low intrinsic gene lists,^19–22^ here we observed that the five MBCs with spindle cell morphology were classified as of claudin-low intrinsic subtype, compared to only three of the 12 MBCs with squamous or chondroid metaplasia (Fig. 3a and Supplementary Tables 1 and 2). By contrast, MBCs with chondroid and squamous differentiation were variably classified as of normal-like, basal-like or claudin-low intrinsic subtypes. Given the heterogeneity observed in the intrinsic subtypes between MBCs of different morphologic subtypes,^2^ we further explored the overall gene expression profiles of these tumors using unsupervised and supervised methods.

Unsupervised hierarchical clustering of gene expression data revealed that the five MBCs with spindle cells formed a stable cluster distinct from the remaining MBCs (*p* < 0.05, Fisher’s exact test, Fig. 3a and Supplementary Fig. 4). The second cluster comprised two sub-clusters, with one encompassing most MBCs with chondroid differentiation and the other comprising most MBCs with squamous differentiation (Fig. 3a). These two sub-clusters were, however, unstable, and the separation between the two histologic subtypes was not statistically significant (*p* = 0.2424, Fisher’s exact test, Supplementary Fig. 4). Consistent with our findings that spindle cell MBCs are more frequently of claudin-low subtype^5^, these data provide evidence that MBCs with spindle cell metaplasia harbor distinct transcriptomic profiles compared to other forms of MBCs.

Significance analysis of microarrays (SAM) revealed that 190 transcripts were downregulated in spindle cell compared to non-spindle cell MBCs (Supplementary Table 9). Functional annotation and pathway analysis of these transcripts using the Ingenuity Pathway Analysis (IPA) software revealed that MBCs with spindle cells displayed lower expression of tight-junction-related genes, including *CLDN3, CLDN8, CGN*, *MYH11*, and *MYH14* (*p*-value < 0.05, Supplementary Fig. 5) than other forms of MBCs. Additionally, spindle cell MBCs differentially expressed EMT-related genes compared to squamous and chondroid MBCs, including reduced *CDH1* and *EPCAM* expression (Supplementary Table 9). The down-regulation of EMT-related genes in spindle cell MBCs, including claudins, is consistent with their claudin-low intrinsic subtype. Consistent with the enrichment of tight-junction and EMT-related genes found by IPA, g:Profiler^23^ pathway analysis of the differentially expressed genes revealed an enrichment for genes involved in cell–cell junction, tight junction and cell junction organization (*p*-value < 0.05) and genes enriched in extracellular matrix organization and cell adhesion (*p*-value < 0.05, Supplementary Table 10).

The comparison between MBCs with and without chondroid differentiation identified 48 differentially expressed transcripts (Supplementary Table 11). Interestingly, chondroid tumors showed over-expression of *EXTL1*, a gene encoding exostosin-like glycosyltransferase 1 and involved in chain elongation of heparan sulfate,^24^ an integral proteoglycan of cartilage, and genes involved in chondrocyte differentiation, including *COL9A1*, *COL11A2,* and *ACAN*. Aggrecan (encoded by *ACAN*) is a proteoglycan and major extracellular matrix component of cartilaginous tissues.^25^ One could posit that overexpression of these genes may contribute to their cartilaginous histologic appearance; however, further studies are warranted to define the basis of their overexpression in MBCs with chondroid differentiation.

Only 19 transcripts were found to be differentially expressed between MBCs with and without squamous differentiation, including the upregulation of *MAPK13*, a gene involved in cell cycle, as well as two genes that regulate proliferation, *EHF* and *MMP7* (Supplementary Table 12).

To validate our gene expression results using an orthogonal method, we performed hierarchical clustering and differential gene expression analysis using the data generated from RNA-sequencing. Transcriptomic profiling by RNA-sequencing generally recapitulated the unsupervised clustering analysis made using gene expression arrays with the MBCs with spindle cells preferentially clustering together (Supplementary Fig. 6). Supervised analysis of gene expression based on the RNA-sequencing data further confirmed the gene expression microarray analysis findings in that *CLDN3* and *CDH1* were downregulated in MBCs with spindle cells; *MAPK13*, *EHF,* and *MMP7* were upregulated in squamous MBCs; and *ACAN*, *COL9A1*, *COL11A2*, and *EXTL1* were upregulated in chondroid MBCs (Fig. 3b, Supplementary Fig. 7 and Supplementary Tables 13–15). These findings confirm our previous observations,^5^ and demonstrate that the gene expression profiles of MBCs vary according to the type of metaplastic elements present.

### Genes amplified and overexpressed in MBCs

**Fig. 4:**
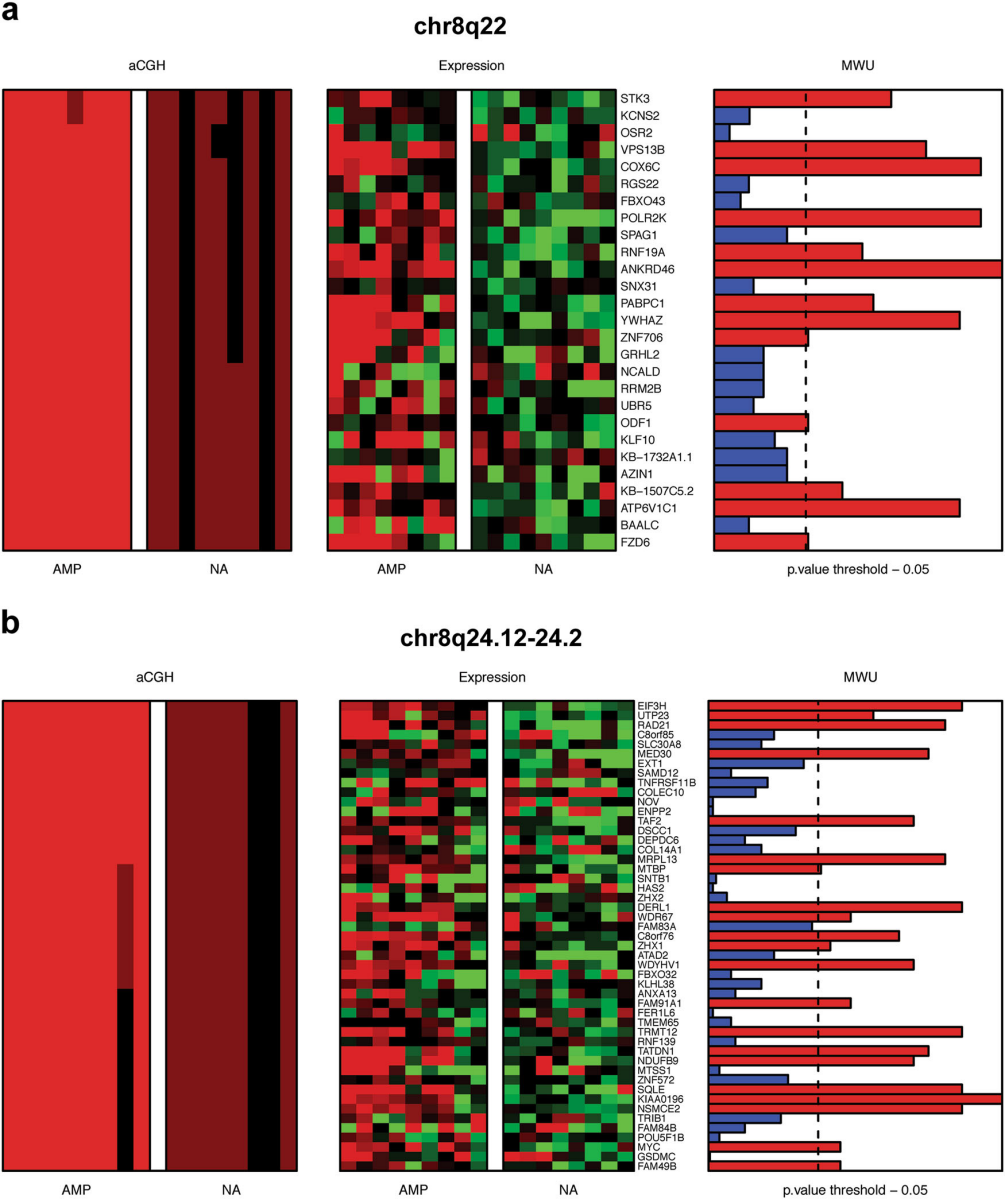
Genes significantly overexpressed when amplified in MBCs. Composite heatmaps of copy number (left) and gene expression (middle) of genes mapping to **a** the 8q22 amplicon and **b** the 8q24.12-24.2 amplicon. Microarray-based gene expression and SNP 6.0 copy number values are depicted in two matching heatmaps, with SNP 6.0 states on the left and expression values on the right, in which the genes are ordered according to their chromosomal positions. Bar plots on the right show the results of Mann–Whitney *U* tests for expression as a continuous variable and gene amplification as the grouping variable. Bars in red show adjusted *p*-values < 0.05. SNP 6.0: copy number loss (green), no copy number change (black), copy number gain (dark red), amplification (bright red). Gene expression: downregulation (green), upregulation (red). *AMP* amplified, *MWU* Mann–Whitney *U* test, *NA* not amplified

Integration of copy number and gene expression data for all 17 MBCs included in this study identified 3071 copy number regulated probes, encoding 2605 genes (Supplementary Table 16). To identify potential amplicon drivers, we interrogated for genes significantly overexpressed when amplified. Pathway analysis of the 153 genes overexpressed when amplified (Supplementary Table 17) using IPA and g:Profiler demonstrated an enrichment of genes involved in oxidative phosphorylation, cellular metabolic pathways and cell cycle (Supplementary Fig. 8 and Supplementary Table 18). Interestingly, several of these genes were present on 8q, a recurrently amplified region in MBCs and TNBCs, including *COX6C*, *YWHAZ*, and *ATP6V1C1* on 8q22 (Fig. 4a) and *RAD21*, *NDUFB9,* and *MYC* on 8q24 (Fig. 4b). As an exploratory, hypothesis-generating analysis, we sought to define whether the repertoire of genes overexpressed when amplified would vary according to the histologic subtype of MBCs. This analysis did not reveal genes significantly overexpressed when amplified when the three subtypes were analyzed separately; it should be noted, however, that this negative finding may stem from the small sample size.

### MBCs are not driven by a recurrent fusion gene or an expressed pathognomonic mutation in cancer genes

**Fig. 5:**
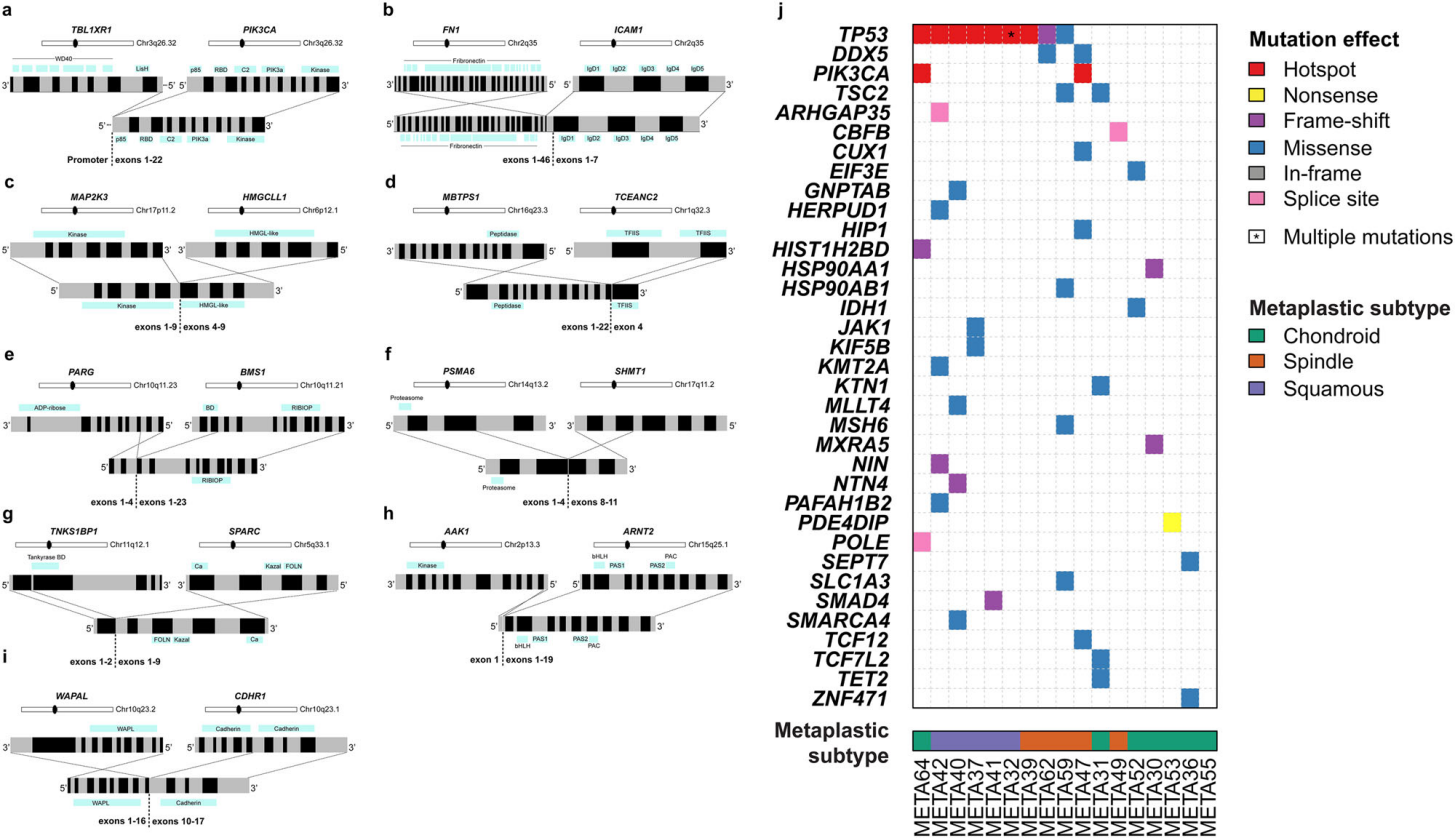
Schematic representation of nine validated expressed in-frame fusion transcripts and repertoire of expressed non-synonymous mutations defined using RNA-sequencing in MBCs. Reverse transcription (RT)-PCR was used to validate in-frame fusion transcripts identified by RNA-sequencing to comprise open reading frames with known associated functions and that harbored intact functional domains. **a** *TBL1XR1-PIK3CA*, **b** *FN1-ICAM1*, **c** *MAP2K3-HMGCLL1*, **d** *MBTPS1-TCEANC2*, **e** *PARG-BMS1,* **f** *PSMA6-SHMT1*, **g** *TNKS1BP1- SPARC,* **h** *AAK1-ARNT2*, **i** *WAPAL-CDHR1*. In each panel, chromosomes are indicated in alternating black and gray boxes, with the 5′ and the 3′ marked at the corresponding end of each gene. Light blue boxes above the schematics of the wild-type genes and below the schematics of the fusion genes indicate the protein domains present. Fusion junctions with respective exon numbers are shown. **j** Expressed non-synonymous mutations identified in 17 MBCs subjected to RNA-sequencing. Mutations affecting genes included in the cancer gene lists^29–31^ are reported. The effects of the mutations are color-coded according to the color key, with hotspot^48^ mutations colored in red. The presence of multiple non-synonymous mutations in the same gene is represented by an asterisk. The metaplastic subtype of each MBC is indicated below the heatmap according to the color key

To determine whether MBCs are driven by a highly recurrent fusion gene, we subjected the 17 MBCs to paired-end RNA-sequencing. Analysis using defuse^26^ and ChimeraScan^27^ identified 43 fusion transcripts (Supplementary Table 19). Akin to the gain-of-function oncogenic fusion genes identified in other special histologic types of breast cancer,^12,13^ we prioritized fusion transcripts that comprised open reading frames with known associated functions, and that harbored intact functional domains. Using the above criteria, we identified and validated by RT-PCR nine in-frame fusion transcripts in the index cases (Figs. 5a–i and Supplementary Table 19), though none was recurrent in our series. Importantly, however, we identified several interesting chimeric transcripts including a promoter swap of *TBL1XR1-PIK3CA* that has previously been described in breast and prostate cancers,^28^ and additional fusions of *WAPAL-CDHR1, MAP2K3-HMGCLL1, PARG-BMS1, FN1-ICAM1, AAK1-ARNT2, TNKS1BP1-SPARC*, *MBTPS1-TCEANC2,* and *PSMA6-SHMT1* (Figs. 5a–i and Supplementary Fig. 9).

To define the repertoire of expressed mutations in MBCs, we performed a mutational analysis of the RNA-sequencing data. Given the challenges for de novo identification of mutations based on RNA-sequencing analysis, we rigorously curated the putative mutations to exclude likely germline polymorphisms and likely passenger mutations and focused on previously described cancer genes included in Kandoth et al. (127 significantly mutated genes),^29^ the Cancer Gene Census^30^ or Lawrence et al. (Cancer5000-S gene set).^31^ The mutational analysis revealed that *TP53* (9/17, 53%), *PIK3CA*, *DDX5,* and *TSC2* (each 2/17, 12%, Fig. 5j and Supplementary Table 20) were recurrently mutated, including *TP53* hotspot mutations C176F, N239S, R249G, R273C, C275R, P278L, and c.673-1G>T splice site, each found in one MBC. Both *PIK3CA* mutations found were the hotspot H1047R mutation. Mutations in other cancer genes including *CBFB*, *IDH1*, *JAK1*, *KMT2A*, *SMAD4,* and *SMARCA4* were found in one MBC each. As a hypothesis-generating analysis, we compared the mutational frequency of *TP53* between MBC subtypes. All MBCs with squamous metaplasia harbored *TP53* mutation, compared to 33% of MBCs of other subtypes (*p*-value = 0.03, Fisher’s exact test, Fig. 5j), similar to our recent report based on whole-exome sequencing.^9^

These data suggest that although MBCs are not driven by a pathognomonic gene fusion or a pathognomonic expressed mutation in previously described cancer genes^29–31^, some of these genetic alterations may play a role in individual MBCs.

## Discussion

Here we have characterized the patterns of CNAs, gene expression, expressed fusion genes and expressed mutations affecting previously described cancer genes in 17 MBCs. Our results demonstrate that MBCs are highly heterogeneous tumors at the genomic and transcriptomic levels, and that they are unlikely to be underpinned by a highly recurrent fusion gene or a pathognomonic expressed mutation in previously described cancer genes^29–31^ (Table 1).

**Table 1:** Summary of molecular features of MBCs

	Metaplastic breast cancer			Triple-negative breast cancer of no special type
Histologic subtype	Chondroid	Spindle	Squamous	
Intrinsic subtype	Predominantly basal-like	Claudin-low	Basal-like, claudin-low and normal breast-like	Predominantly basal-like
Triple-negative breast cancer subtypes	Mesenchymal	Predominantly mesenchymal stem-like and unstable	Various, except luminal androgen receptor and immunomodulatory	All types
Integrative clusters	Preferentially 9	Predominantly 4	Various	Predominantly 10, 4, 9
BRCAness	Preferentially non-BRCAness	Preferentially non-BRCAness	Preferentially BRCAness	Roughly equal
*TP53* mutations	75%	50%	78%	81%
Mutations in PI3K/AKT/mTOR pathway	44%	70%	67%	22%
Mutations in canonical Wnt pathway	56%	50%	44%	28%
Chromosomal instability	+	+	++	++
Copy number alterations	Frequent gains of 1q and 8q, losses of 5q and 12q			
	Frequent high-level gain of 8q21.11-24.3^a^	Infrequent copy number gain of CLDN3/4^a^	Infrequent amplification of 8q24, frequent losses of 7q and 12q^a^	
Gene expression	Up-regulation of genes involved in chondrocyte differentiation^a^	Down-regulation of claudins, E-cadherin and EpCAM^a^	Up-regulation of cell cycle-related genes^a^	
Pathognomonic fusion gene	Lack of pathognomonic fusion gene			

The main molecular features of metaplastic breast carcinomas (MBCs) according to histologic subtypes, compared to those of triple-negative breast cancers of no special type. The table summarizes the main findings from Weigelt et al.,^5^ Ng et al.^9^ and the current study

^a^ Compared to other histologic subtypes of metaplastic breast cancer.

We have previously demonstrated that based on PAM50/claudin-low molecular subtyping, MBCs with spindle metaplasia are preferentially classified as of claudin-low subtype, whereas MBCs with squamous or chondroid metaplasia are more frequently classified as of basal-like or even normal breast-like subtypes than as of claudin-low subtype (Table 1).^5^ Here we expand on our previous observations, and demonstrate that MBCs with spindle cell metaplasia display transcriptomic features characterized by an EMT-like gene expression profile, which is not as conspicuous in MBCs with other forms of metaplasia. MBCs with chondroid or squamous metaplasia, but not MBCs with spindle metaplasia, were found to harbor recurrent copy number gains of 7q11.22-23, a region that encompasses *CLDN3* and *CLDN4*. Given that the lack of 7q11.22-23 gain was the only CNA feature found to distinguish MBCs with spindle cells and the remaining MBCs, the vast majority of the differences in gene expression between these subsets of MBCs are therefore unlikely to be copy number-driven. Although MBCs with spindle cell metaplasia more frequently displayed *PIK3CA* mutations than MBCs with other types of metaplasia, *PIK3CA* mutations were also found in MBCs with squamous differentiation.^9^ These results suggest that the differences in gene expression between MBCs with or without spindle cell metaplasia may stem from other types of somatic genetic alterations or epigenetic modifications or distinct cells of origin. Our findings also contextualize specific aspects related to the molecular classification of MBCs; the characteristic EMT-like transcriptomic profiles and high prevalence of claudin-low subtype reported in studies on MBCs^3,21,22^ may actually stem from the fact that those studies primarily focused on MBCs that displayed a spindle cell/sarcomatoid histologic subtype.

In terms of CNAs, MBCs as a group have been shown to be remarkably similar to other TNBCs (Table 1).^7^ When we compared the copy number profiles of the 17 MBCs in the current cohort to those of 34 grade-matched TNBCs,^16^ the frequency of CNAs was similar between MBCs and TNBCs. We did identify, however, some regions that were altered significantly more frequently in MBCs, including losses of 6q13-14.1, 6q21, 10p13-15.3, 10q11.21-21.1, and 12p13.31 (Supplementary Fig. 2 and Supplementary Table 3). Furthermore, integration of genomic and transcriptomic information yielded the amplification of 8q as a CNA that significantly affects gene expression in MBCs. Amplification of 8q, however, is a relatively frequent finding in other forms of TNBCs.

At variance with other rare forms of TNBC,^12–14,32^ MBCs were found not to harbor a highly recurrent expressed fusion gene or a highly recurrent expressed mutation affecting previously described cancer genes. In addition, the histologic differences observed in distinct subtypes of MBCs appear not to be underpinned by specific expressed fusion genes or expressed mutations affecting previously described cancer genes. Potentially pathogenic fusion genes private to individual tumors were, however, identified, some of which comprised partner genes involved in the PI3K pathway and/or DNA damage repair mechanisms. Specifically, the *TBL1XR1-PIK3CA* fusion gene, which was detected in two ER-negative invasive breast cancers from the TCGA breast cancer study and may result in PIK3CA over-expression,^28^ was detected in META52; however, in this case, the PIK3CA expression levels were not significantly higher than in other MBCs studied here (Supplementary Figs. 10a and 10b). The fusion genes *PARG-BMS1* and *WAPAL-CDHR1* contain partners whose protein products are involved in genomic integrity; disruption of these genes by a rearrangement could, theoretically, contribute to the complex patterns of genomic rearrangements observed in MBCs. The *WAPAL-CDHR1* fusion transcript contains the first 16 exons of the Wings apart-like protein homolog (*WAPAL*) gene and the last seven exons of the Cadherin Related Family Member 1 (*CDHR1*) gene. *WAPAL* encodes a cohesin binding protein necessary for sister chromatid resolution which is truncated at residue 1107 in the chimeric transcript. Double mutations in *WAPAL* at M1116A/I1120A have been found to result in a functionally defective protein that can still bind cohesin.^33^ These data suggest that this chimeric transcript may potentially result in a defective form of the *WAPAL* gene product that may affect chromatid cohesion and subsequently the rate of segregation errors and aneuploidy. Of the other fusion genes identified and validated in our study, *PARG-BMS1* and *TNKS1BP1-SPARC* may also have an impact on genomic integrity, given that the Poly(ADP-ribose) glycohydrolase (PARG) protein hydrolyzes PAR (poly(ADP-ribose)) and plays an important role in DNA damage repair,^34^ whereas the Tankyrase 1 Binding Protein 1 (TNKS1BP1) protein is a binding partner of TANK1,^35^ a member of the PARP superfamily that regulates telomere length, DNA repair and WNT signaling.^36^

Here, we found that 53% of MBCs expressed *TP53* mutations. Apart from frequent *TP53* mutations, we have recently demonstrated by whole-exome sequencing analysis that *PIK3CA*, *PIK3R1*, and *PTEN* are significantly mutated in MBCs and that up to 60% of MBCs harbor somatic mutations in the PI3K/AKT/mTOR pathway (Table 1).^9^ These observations are in agreement with a previous study, in which sequencing analyses of known pathogenic *PIK3CA* and *PTEN* mutations revealed their involvement in 53% of MBCs.^3^ Here we have also detected homozygous deletions of *PTEN* in two MBCs (META32 and META41) and loss of expression by immunohistochemistry in additional two cases (META49 and META62), suggesting that 24% of MBCs likely displayed PTEN loss-of-function. In an additional four cases (META31, META47, META59, and META64), we identified either bona fide activating *PIK3CA* mutation or *TSC2* missense mutations predicted to be likely pathogenic by mutation function predictors.^37^ These findings are consistent with the notion that dysregulation of the PI3K/AKT pathway is likely to play a pivotal role in MBCs. Of note, however, *PIK3CA* and *PTEN* mutations in TNBCs are not restricted to MBCs. In fact, of the TNBCs from The Cancer Genome Atlas breast cancer study, approximately 10% of cases harbor amplification or somatic mutations in *PIK3CA* and 8% harbor homozygous deletions or somatic mutations in *PTEN*.^38^ Therefore, these recurrent alterations affecting PI3K pathway-related genes are unlikely to explain the unique histologic features of MBCs. We also reported that MBCs frequently harbored mutations in the Wnt pathway.^9^ Consistent with our observations from whole-exome sequencing analysis of MBCs,^9^ we did not identify expressed mutations affecting *CTNNB1*. We did, however, detect an expressed frameshift *SMAD4* mutation and an expressed missense *TCF7L2* mutation. Of note, most of the Wnt pathway-related genes previously found to be mutated in MBCs are not in the cancer gene lists employed for the RNA-sequencing-based mutation analysis^29–31^ and were not within the scope of the mutational analysis performed in this study, given the challenges in identifying somatic mutations using RNA-sequencing alone.

This study has several limitations. Owing to the rarity of MBCs and the challenges in having frozen blocks representative of the metaplastic elements of MBC, our sample size was small. It should be noted, however, that despite the small sample size, we could detect transcriptomic features and CNAs that varied between the subtypes of MBC. Importantly, our study was sufficiently powered to have detected a pathognomonic fusion gene, a pathognomonic expressed mutation affecting a previously reported cancer gene, or a pathognomonic CNA in at least two cases. This suggests that, unlike other rare forms of TNBC,^12–14,32^ this rare and aggressive histologic special type of TNBCs is unlikely to be driven by a highly recurrent fusion gene, expressed mutation in a known cancer gene or CNA. Second, we have focused on the repertoire of CNAs, transcriptomic changes, fusion genes and expressed mutations in MBCs, and were unable to identify pathognomonic genetic alterations that define each histologic subtype of MBCs. Although mutations were identified using RNA-sequencing, defining the repertoire of somatic mutations using RNA-sequencing data has proven to be fraught with difficulties, with high rates of false-negative and false-positive results. Through a series of rigorous filters, we were able to identify recurrently expressed mutations in MBCs, which were consistent with the observations we have made by whole-exome sequencing of 35 MBCs.^9^ Further studies are warranted to define whether the distinctive features of MBCs and of each subtype of the disease would be driven by somatic genetic alterations affecting non-coding regions of the genome or by specific repertoires of epigenetic alterations.

Despite these limitations, our study demonstrates that MBCs resemble other high-grade TN IDC-NSTs at the genomic level. We have provided direct evidence that MBCs are unlikely to be driven by a highly-recurrent/ pathognomonic expressed fusion gene or expressed mutation affecting known cancer genes, and that transcriptomic profiles and, to a lesser extent, gene CNAs varied between the metaplastic elements present in MBCs, corroborating the notion that MBCs comprise a heterogeneous group of breast cancers whose common denominator is the presence of metaplastic elements. Further studies are warranted to define whether the histologic diversity of MBCs stems from distinct non-coding somatic genetic alterations and/or epigenetic events, distinct cells of origin, or the interactions between specific genetic/ epigenetic alterations and cell of origin.

## Methods

### Case selection and nucleic acids extraction

Cases diagnosed as MBCs were selected from a previously described cohort.^5^ In this study we included cases for which both gene expression microarray profiling and SNP 6.0 copy number profiling were available from our previous study,^5^ and for which sufficient RNA was available for RNA-sequencing (see below, Supplementary Fig. 1 and Supplementary Table 1). All cases were independently reviewed by three pathologists with an interest and expertize in breast cancer (FCG, AV-S and JSR-F), who classified the tumors into three groups: MBCs with spindle cell metaplasia (*n* = 5), squamous metaplasia (*n* = 5) or chondroid metaplasia (*n* = 7) according to the most prevalent component in the sample subjected to molecular analyses (Supplementary Table 1), given that the most abundant metaplastic component has been shown to have a substantial impact on the transcriptomic profiles of each tumor.^5^ Tumors were graded according to the Nottingham grading system^39^ and tumor cell content for each lesion was defined semi-quantitatively (AV-S and JSR-F). Samples were anonymized prior to analysis. This study was approved by the local research ethics committees of the authors’ institutions. Written consent was obtained from patients alive and contactable prior to the start of the study. Methods were performed in accordance with relevant regulations and guidelines. Nucleic acid extraction and quality control were performed as previously described.^5^

### Immunohistochemistry

Immunohistochemical analysis was performed for ER, PR, and HER2, CK5/6, CK14, CK17, PTEN, p53, p63, c-KIT, and EGFR on representative sections from formalin-fixed paraffin-embedded (FFPE) tissue blocks (Supplementary Table 21 and Supplementary Methods).

### Gene copy number analysis

Copy number profiling data using the human SNP Array 6.0 (Affymetrix)^5^ were re-analyzed (Supplementary Methods). Focal amplifications were defined as amplifications/high-level gains that were smaller than 25% of the respective chromosome arm and visually inspected using genome plots. Homozygous deletions were further defined using ASCAT^40^ and ABSOLUTE^41^ (Supplementary Methods). Tumor cell content was estimated using ABSOLUTE.^41^ Statistically significant differences between CNAs of different morphologic subtypes of MBCs were defined using multi-Fisher’s exact tests and unsupervised hierarchical cluster analysis was performed as previously described (Supplementary Methods).^7^ For the comparison of gene copy number profiles of MBCs with those of common-type TNBCs, SNP 6.0 array data were obtained from triple-negative IDC-NSTs^16^ and matched in a 2:1 ratio with MBCs and processed using the aroma.affymetrix and DNAcopy packages as described above.

### Gene expression profiling

Gene expression profiling data using the HumanHT-12 v4 Expression BeadChip Kit (Illumina)^5^ were re-normalized and annotated (Supplementary Methods). Unsupervised hierarchical clustering analysis was performed as previously described^42^ (Supplementary Methods). Differentially expressed genes were determined by significance analysis of microarrays (SAM),^43^ adopting a false discovery rate of 1% after 100 permutations.

### Integration of copy number and expression data

To identify genes whose expression levels correlate with CNAs and genes that were up-regulated when gained, down-regulated when lost or over-expressed when amplified, Pearson’s correlations and Mann–Whitney *U* tests were used as previously described^7^ (Supplementary Methods).

### Pathway analysis

Significantly regulated pathways and networks in the gene expression data were determined using Ingenuity Pathway Analysis (IPA, http://www.ingenuity.com,) and g:Profiler^23^ (Supplementary Methods).

### RNA-sequencing and fusion transcript identification

RNA-sequencing (2 × 54 bp) was performed for all 17 MBCs included in this study using the standard Illumina mRNA library protocol on a Genome Analyzer II (Illumina) as previously described.^42^ deFuse^26^ and ChimeraScan^27^ were used to identify mate-pairs supporting novel chimeric transcripts as previously described^42^ (Supplementary Methods). Candidates that resulted in open reading frames were annotated using OncoFuse.^44^ Nominated in-frame fusion gene candidates, candidates identified by both deFuse and ChimeraScan, as well as those with known associated functions, and those that harbored intact functional domains were prioritized for validation in the index cases by reverse transcription (RT)-PCR (Supplementary Table 22 and Supplementary Methods). Fusion genes validated in the index cases were further screened in all cases in the cohort for which RNA samples were available.

### RNA-sequencing gene expression and mutation analysis

For gene expression analysis, RNA-sequencing data were aligned to the transcriptome (GRCh37) using STAR^45^ and differential expression analyses were performed using limma (Supplementary Methods).^46^ Mutation analysis was performed according to the Genome Analysis Toolkit^47^ Best Practices workflow for single nucleotide variant (SNVs) and small insertion and deletion (indel) calling on RNA-seq data (http://gatkforums.broadinstitute.org/gatk/discussion/3891/calling-variants-in-rnaseq). Variants affecting hotspots^48^ were white-listed, and additional mutations were defined using GATK HaplotypeCaller, removing likely polymorphisms and likely passenger variants (Supplementary Methods). Only variants covered by at least five reads, with at least two reads supporting the variant and at least two reads supporting the reference allele were included, as those devoid of a reference allele were highly enriched for germline variations.^42^ Mutations affecting cancer genes included in the lists described by Kandoth et al., (127 significantly mutated genes),^29^ the Cancer Gene Census^30^ and/or Lawrence et al., (Cancer5000-S gene set)^31^ and their functional effects (Supplementary Methods) were reported.

### Power calculations

The binomial probability was used to estimate the statistical power of identifying a pathognomonic alteration, focusing on those that would be detected in at least two cases (i.e., recurrent). To calculate the statistical power, we made the assumption that a pathognomonic alteration in MBC would be present in ≥70% of cases, which is a conservative estimate and one that is substantially lower than the observed >95% frequency of *ETV6-NTRK3* fusion in breast secretory carcinomas (originally identified in a cohort of six)^12^ and >90% frequency of *MYB-NFIB* fusion in adenoid cystic carcinoma (originally identified in a cohort of ten, of which only four were breast adenoid cystic carcinomas).^13^ Thus by sequencing five samples (the smallest number of cases for any MBC histologic subtype), we would have 97% statistical power to detect a pathognomonic alteration in at least two cases. Even if we lower the assumed frequency of a pathognomonic driver alteration to be present in 50% of the cases, we would still have 80% statistical power to detect the pathognomonic alteration in at least two cases.

### Code availability

The R script of all microarray-based gene expression profiling and gene copy number analyses performed is available at GitHub (https://github.com/charlottekyng/piscuoglio-et-al-metaplastic-breast-carcinoma).

### Data availability

The raw and processed gene expression and SNP 6.0 data are available on Gene Expression Omnibus (GSE57549). RNA-sequencing data have been deposited to the Sequence Read Archive (SRP070780).

## Electronic supplementary material

Supplementary Methods

Supplementary Legends

Supplementary Figure 1

Supplementary Figure 2

Supplementary Figure 3

Supplementary Figure 4

Supplementary Figure 5

Supplementary Figure 6

Supplementary Figure 7

Supplementary Figure 8

Supplementary Figure 9

Supplementary Figure 10

Supplementary Table 1

Supplementary Table 2

Supplementary Table 3

Supplementary Table 4

Supplementary Table 5

Supplementary Table 6

Supplementary Table 7

Supplementary Table 8

Supplementary Table 9

Supplementary Table 10

Supplementary Table 11

Supplementary Table 12

Supplementary Table 13

Supplementary Table 14

Supplementary Table 15

Supplementary Table 16

Supplementary Table 17

Supplementary Table 18

Supplementary Table 19

Supplementary Table 20

Supplementary Table 21

Supplementary Table 22
