# Supplementary material for: Genomic and transcriptomic heterogeneity in metaplastic carcinomas of the breast
Source: NPJ Breast Cancer. 2017 Dec 1;3:48. doi: 10.1038/s41523-017-0048-0 (PMC5711926; doi:10.1038/s41523-017-0048-0)
Supplement: Supplementary file 1 — Supplementary Methods [file 41523_2017_48_MOESM1_ESM.docx]

**Genomic and transcriptomic heterogeneity in metaplastic carcinomas of the breast**

**Piscuoglio et al.**

**Supplementary Information**

**SUPPLEMENTARY METHODS**

**Immunohistochemistry**

Immunohistochemical analysis was performed for ER, PR and HER2, cytokeratin (CK) 5/6, CK14, CK17, PTEN, p53, p63, c-KIT and EGFR on representative sections from formalin-fixed paraffin-embedded (FFPE) tissue blocks, as previously described^1-3^ (Supplementary Table 21). Positive and negative controls were included in each slide run. The results of the immunohistochemical analyses were interpreted independently by three pathologists (FCG, AV-S and JSR-F); discordances were resolved by reviewing the cases together on a multi-headed microscope.

**Gene copy number analysis**

Raw data from the human SNP Array 6.0 (Affymetrix) were processed using the CRMAv2 algorithm in the aroma.affymetrix package.^4^ Log_2_ ratios obtained from CRMAv2 were smoothed using the circular binary segmentation (cbs) algorithm in the DNAcopy package in R.^5^ cbs-smoothed Log_2_ ratios were then used in all subsequent analyses to define losses, gains and amplifications/ high-level gains as previously described.^6^ Low level gains were defined as cbs-smoothed Log_2_ ratios of 0.15 to 0.5 and gene amplifications/ high-level gains >0.5. Losses were defined as cbs-smoothed Log_2_ ratios of -0.15 to -1 and deletions <-1. Focal amplifications were defined as amplifications/ high-level gains that were smaller than 25% of the respective chromosome arm and visually inspected using genome plots of cbs-smoothed Log_2_ ratios.

For the analysis with ASCAT,^7^ raw data were processed using Affymetrix Power Tools and Log_2_ ratios were transformed and extracted according to the PennCNV-Affy protocol (http://penncnv.openbioinformatics.org/en/latest/user-guide/affy/), using the default canonical clustering file for the SNP 6.0 arrays, then segmented using ASCAT.^7^ Using ASCAT, homozygous deletions were then defined as regions with total copy number 0. For the analysis with ABSOLUTE, raw data were processed using the HAPSEG package^8^ supplied with the ABSOLUTE software bundle and output from HAPSEG was used as input to ABSOLUTE (v1.0.6).^9^ Solutions from ABSOLUTE were manually reviewed as recommended,^9,10^ and homozygous deletions were defined as regions with modal copy number 0. Homozygous deletions were only considered valid if they were defined by both ASCAT and ABSOLUTE.

Categorical copy number data were subjected to multi-Fisher’s exact test with adjustment for multiple testing using the Benjamini-Hochberg method to identify statistically significant differences between the genomic profiles of the different morphologic subtypes of MBCs.^11^ Unsupervised hierarchical cluster analysis was performed with categorical states (i.e. deletions, gains, losses and amplifications/ high-level gains), using Euclidean distance and Ward’s clustering algorithm as previously described.^11^ Cluster stability was assessed using pvclust.^12^

**Gene expression profiling**

Raw gene expression values from the HumanHT-12 v4 Expression BeadChip Kit (Illumina) were robust-spline normalized using the Bioconductor lumi package in R.^13^ Genes were mapped to their genomic location using the Ensembl database, resulting in a dataset of 34,305 probes with accurate and unequivocal mapping information.

For unsupervised hierarchical clustering analysis, probes that had a median absolute deviation (MAD) greater than 1.2 were included and replicate probes were filtered based on the MAD, resulting in a set of 1,411 probes for clustering. Probes were median centered and clustered using Ward’s hierarchical clustering using Pearson’s correlation as the distance metric. A threshold of 1.2 was selected based on an assessment of cluster stability using pvclust.^12^

**Integration of copy number and expression data**

To identify genes whose expression levels correlate with copy number changes, cbs-smoothed Log_2_ ratios from SNP 6.0 data were used to assign the copy number states for each of the 34,305 probes in the gene expression data set using the median values for all probes that overlap with the genomic position of each gene, essentially as previously described.^11,14^

To define genes that were up-regulated when gained, down-regulated when lost or over-expressed when amplified, we performed a Mann–Whitney U-test using categorical copy number states (i.e. gain vs. no gain, loss vs. no loss or amplification/ high-level gain vs. no amplification/ high-level gain) as the grouping variable and the expression of genes as the dependent variable as previously described.^11,14^

**Pathway analysis**

For the IPA analysis, the fold difference of differentially expressed transcripts identified by SAM were mapped to pathways and networks available in the Ingenuity database and ranked by score. The IPA score indicates the likelihood of the genes in a pathway/ network being found together due to chance. Using a 99% confidence level, scores of ≥3 were considered significant.^14,15^ To validate the IPA findings, additional pathway analyses were performed using g:Profiler,^16^ using the differentially expressed genes identified by SAM.

**RNA-sequencing and fusion transcript identification**

deFuse^17^ and ChimeraScan^18^ were used to identify mate-pairs supporting novel chimeric transcripts as previously described.^15^ To remove normal transcriptional variants, we excluded candidates that were identified in a set of 47 normal breast tissues in The Cancer Genome Atlas (TCGA)^19^ using the same algorithms. Candidates with at least 2 split reads supporting the fusion and those that resulted in open reading frames were retained. Reading frames of the fusion transcripts were annotated using OncoFuse.^20^ Nominated in-frame fusion gene candidates identified by RNA-sequencing were prioritized for validation in the index cases. Candidates identified by both deFuse and ChimeraScan, as well as those with known associated functions, and those that harbored intact functional domains were also prioritized. Validation was performed by reverse transcription (RT)-PCR. The split sequences (sequences encompassing the breakage point/fusion junctions) were used as reference to design primer sets for each fusion gene pair (Supplementary Table 21). For this validation, 100 ng of total RNA was reverse transcribed using Superscript III (Life Technologies) and PCR was performed using the GoTaq Green Master Mix Kit (Promega). Fusion genes validated in the index cases were further screened in all cases in the cohort for which RNA samples were available.

**RNA-sequencing gene expression and mutation analysis**

For gene expression analysis, RNA-sequencing data were aligned to the transcriptome (based on the human reference genome GRCh37) using STAR.^21^ TMM normalization and the voom transformation^22^ was applied to per-gene read counts using the limma package.^23^ Differential expression between the spindle, squamous and chondroid MBCs was performed using the limma package.^23^

For mutation analysis, STAR-aligned BAM files were post-processed using the MarkDuplicates, SplitNCigarReads and BaseRecalibration tools in the Genome Analysis Toolkit^24^ according to the Best Practices workflow for single nucleotide variant (SNVs) and small insertion and deletion (indel) calling on RNA-seq data (<http://gatkforums.broadinstitute.org/gatk/discussion/3891/calling-variants-in-rnaseq)>. Hotspots^25^ supported by at least two reads, with QD (QualityByDepth) >2 and not present as germline variants in any of the 1000 Genomes, ESP-6500 and the Exome Aggregation Consortium (ExAC) datasets were white-listed. To discover additional mutations, SNVs and indels were defined using the GATK HaplotypeCaller. To remove germline polymorphisms and artifacts, we filtered out mutation calls from the RNA-sequencing data of 13 normal breast tissue from The Cancer Genome Atlas (TCGA)^19^ processed using the same analysis pipeline, present as germline variants in any of the 1000 Genomes, ESP-6500 and the Exome Aggregation Consortium (ExAC) datasets. Missense SNVs defined as non-deleterious/ passenger by both MutationTaster^26^ and CHASM (breast),^27^ a combination of mutation function predictors shown to have a high negative predictive value,^28^ were considered likely passenger alterations and excluded. Variants found in the COSMIC dataset^29^ were white-listed. Only SNVs or indels with a minimum read depth of five, with at least two reads supporting the variant and at least two reads supporting the reference alleles were included, as those devoid of a reference allele were highly enriched for germline variations.^15^ Mutations affecting genes included in the cancer gene lists described by Kandoth et al. (127 significantly mutated genes),^30^ the Cancer Gene Census^31^ or Lawrence et al. (Cancer5000-S gene set)^32^ were reported and their effect annotated using MutationTaster,^26^ CHASM (breast)^27^ and FATHMM.^33^

**SUPPLEMENTARY REFERENCES**

1. Geyer, F.C. *et al.* Molecular analysis reveals a genetic basis for the phenotypic diversity of metaplastic breast carcinomas. *J Pathol* **220**, 562-73 (2010).

2. Lacroix-Triki, M. *et al.* Mucinous carcinoma of the breast is genomically distinct from invasive ductal carcinomas of no special type. *J Pathol* **222**, 282-98 (2010).

3. Sakr, R.A. *et al.* PI3K pathway activation in high-grade ductal carcinoma in situ--implications for progression to invasive breast carcinoma. *Clin Cancer Res* **20**, 2326-37 (2014).

4. Bengtsson, H., Wirapati, P. & Speed, T.P. A single-array preprocessing method for estimating full-resolution raw copy numbers from all Affymetrix genotyping arrays including GenomeWideSNP 5 & 6. *Bioinformatics* **25**, 2149-56 (2009).

5. Olshen, A.B., Venkatraman, E.S., Lucito, R. & Wigler, M. Circular binary segmentation for the analysis of array-based DNA copy number data. *Biostatistics* **5**, 557-72 (2004).

6. Natrajan, R. *et al.* A whole-genome massively parallel sequencing analysis of BRCA1 mutant oestrogen receptor-negative and -positive breast cancers. *J Pathol* **227**, 29-41 (2012).

7. Van Loo, P. *et al.* Allele-specific copy number analysis of tumors. *Proc Natl Acad Sci U S A* **107**, 16910-5 (2010).

8. Carter, S.L., Meyerson, M. & Getz, G. Accurate estimation of homologue-specific DNA concentration-ratios in cancer samples allows long-range haplotyping. *Scott L. Carter*, 59 (2011).

9. Carter, S.L. *et al.* Absolute quantification of somatic DNA alterations in human cancer. *Nat Biotechnol* **30**, 413-21 (2012).

10. Landau, D.A. *et al.* Evolution and impact of subclonal mutations in chronic lymphocytic leukemia. *Cell* **152**, 714-26 (2013).

11. Horlings, H.M. *et al.* Genomic profiling of histological special types of breast cancer. *Breast Cancer Res Treat* **142**, 257-69 (2013).

12. Suzuki, R. & Shimodaira, H. Pvclust: an R package for assessing the uncertainty in hierarchical clustering. *Bioinformatics* **22**, 1540-2 (2006).

13. Du, P., Kibbe, W.A. & Lin, S.M. lumi: a pipeline for processing Illumina microarray. *Bioinformatics* **24**, 1547-8 (2008).

14. Lopez-Garcia, M.A. *et al.* Transcriptomic analysis of tubular carcinomas of the breast reveals similarities and differences with molecular subtype-matched ductal and lobular carcinomas. *J Pathol* **222**, 64-75 (2010).

15. Piscuoglio, S. *et al.* Integrative genomic and transcriptomic characterization of papillary carcinomas of the breast. *Mol Oncol* **8**, 1588-602 (2014).

16. Reimand, J. *et al.* g:Profiler-a web server for functional interpretation of gene lists (2016 update). *Nucleic Acids Res* **44**, W83-9 (2016).

17. McPherson, A. *et al.* deFuse: an algorithm for gene fusion discovery in tumor RNA-Seq data. *PLoS Comput Biol* **7**, e1001138 (2011).

18. Iyer, M.K., Chinnaiyan, A.M. & Maher, C.A. ChimeraScan: a tool for identifying chimeric transcription in sequencing data. *Bioinformatics* **27**, 2903-4 (2011).

19. Cancer Genome Atlas, N. Comprehensive molecular portraits of human breast tumours. *Nature* **490**, 61-70 (2012).

20. Shugay, M., Ortiz de Mendibil, I., Vizmanos, J.L. & Novo, F.J. Oncofuse: a computational framework for the prediction of the oncogenic potential of gene fusions. *Bioinformatics* **29**, 2539-46 (2013).

21. Dobin, A. *et al.* STAR: ultrafast universal RNA-seq aligner. *Bioinformatics* **29**, 15-21 (2013).

22. Law, C.W., Chen, Y., Shi, W. & Smyth, G.K. voom: Precision weights unlock linear model analysis tools for RNA-seq read counts. *Genome Biol* **15**, R29 (2014).

23. Ritchie, M.E. *et al.* limma powers differential expression analyses for RNA-sequencing and microarray studies. *Nucleic Acids Res* **43**, e47 (2015).

24. McKenna, A. *et al.* The Genome Analysis Toolkit: a MapReduce framework for analyzing next-generation DNA sequencing data. *Genome Res* **20**, 1297-303 (2010).

25. Chang, M.T. *et al.* Identifying recurrent mutations in cancer reveals widespread lineage diversity and mutational specificity. *Nat Biotechnol* **34**, 155-63 (2016).

26. Schwarz, J.M., Rodelsperger, C., Schuelke, M. & Seelow, D. MutationTaster evaluates disease-causing potential of sequence alterations. *Nat Methods* **7**, 575-6 (2010).

27. Carter, H. *et al.* Cancer-specific high-throughput annotation of somatic mutations: computational prediction of driver missense mutations. *Cancer Res* **69**, 6660-7 (2009).

28. Martelotto, L.G. *et al.* Benchmarking mutation effect prediction algorithms using functionally validated cancer-related missense mutations. *Genome Biol* **15**, 484 (2014).

29. Forbes, S.A. *et al.* COSMIC: exploring the world's knowledge of somatic mutations in human cancer. *Nucleic Acids Res* **43**, D805-11 (2015).

30. Kandoth, C. *et al.* Mutational landscape and significance across 12 major cancer types. *Nature* **502**, 333-9 (2013).

31. Futreal, P.A. *et al.* A census of human cancer genes. *Nat Rev Cancer* **4**, 177-83 (2004).

32. Lawrence, M.S. *et al.* Discovery and saturation analysis of cancer genes across 21 tumour types. *Nature* **505**, 495-501 (2014).

33. Shihab, H.A. *et al.* Predicting the functional, molecular, and phenotypic consequences of amino acid substitutions using hidden Markov models. *Hum Mutat* **34**, 57-65 (2013).
