# Supplementary material for: Genomic and transcriptomic heterogeneity in metaplastic carcinomas of the breast
Source: NPJ Breast Cancer. 2017 Dec 1;3:48. doi: 10.1038/s41523-017-0048-0 (PMC5711926; doi:10.1038/s41523-017-0048-0)
Supplement: Supplementary file 2 — Supplementary Legends [file 41523_2017_48_MOESM2_ESM.docx]

**SUPPLEMENTARY FIGURE AND TABLE LEGENDS**

**Supplementary Figure 1: Schematic representation of the cohorts included in Weigelt et al.,**[**^5^**](#_ENREF_5) **Ng et al.**[**^9^**](#_ENREF_9) **and the current study.**

Cases with genomic and transcriptomic profiling data generated with SNP6 arrays, expression microarrays and RNA-sequencing were included in the current study.

**Supplementary Figure 2: Comparison between the genomic profiles of the 17 MBCs included in this study and 34 histologic grade-matched triple-negative breast carcinomas (TNBCs) of no special type.**

Frequency plots of copy number gains (green bars) or losses (red bars) in MBCs versus TNBCs (y-axis). Inverse Log_10_ values of Fisher’s exact *p-*values are plotted according to genomic position (x-axis).

**Supplementary Figure 3: Comparative frequency plots of copy number alterations between histologic subtypes of MBCs, for a) spindle vs non-spindle MBCs, b) squamous vs non-squamous MBCs, and c) chondroid vs non-chondroid MBCs.**

In each panel (**a-c**), on the left is the proportion of tumors in which each probe is gained (green bars) or lost (red bars) plotted (y-axis) for each probe according to its genomic position (x-axis). The right graph shows amplifications (green) and deletions (red). Inverse Log_10_ values of Fisher’s exact *p-*values are plotted according to genomic position (x-axis) at the bottom of each graph. (**d**) Fraction of genome altered in non-squamous vs squamous MBCs, non-chondroid vs chondroid MBCs, and spindle vs non-spindle MBCs. Statistical comparisons were performed using Mann-Whitney U tests.

**Supplementary Figure 4: Cluster stability of unsupervised hierarchical clustering of MBCs based on gene expression arrays using pvclust**.

**Supplementary Figure 5: Schematic representation of tight-junction canonical pathway based on the genes differentially expressed between spindle cell vs non-spindle cell MBCs generated using the Ingenuity Pathway Analysis software**.

**Supplementary Figure 6:** **Hierarchical cluster analysis of gene expression of MBCs based on RNA-sequencing.**

(**a**) Hierarchical clustering was based on the Ward’s algorithm and Pearson correlation as the distance metric. (**b**) Cluster stability of unsupervised hierarchical clustering of MBCs based on RNA-sequencing using pvclust.

**Supplementary Figure 7:** **Validation of genes identified as significantly differentially expressed by microarray-based gene expression analysis between squamous vs non-squamous MBCs and chondroid vs non-chondroid MBCs using RNA-sequencing.**

Validation of significantly differentially expressed genes between (**a**) squamous and non-squamous MBCs and (**b**) chondroid and non-chondroid MBCs was performed using RNA-sequencing data. For each gene, normalized expression value is plotted for each MBC, grouped by histologic subtype. *p*-values were calculated from the differential expression analysis using limma.[^47^](#_ENREF_47)

**Supplementary Figure 8:** **Genes involved in oxidative phosphorylation and the cell cycle are copy number related in MBCs.**

(**a**) Genes involved in oxidative phosphorylation are amplified and overexpressed in MBCs as defined by Ingenuity Pathway (IPA) analysis. (**b**) Cell cycle, cell movement and connective tissue disorder related genes are upregulated in MBCs. (**c**) Gene expression, protein synthesis and cell cycle genes are upregulated in MBCs. Pathways and networks were generated using the IPA software.

**Supplementary Figure 9: Sanger sequencing and schematic representation of selected validated in-frame fusion genes in MBCs.**

Sequence chromatograms spanning the junction (dotted line) of the fusion transcripts are shown on the left. A schematic representation of nominated fusion transcripts is shown on the right. Chromosomes are indicated in alternating black and grey boxes, with the 5’ and the 3’ marked at the corresponding end of each gene. Light blue boxes above the schematics of the wild-type genes and below the schematics of the fusion genes indicate the protein domains present. Fusion junctions with respective exon numbers are shown.

**Supplementary Figure 10: *PIK3CA* and *TBL1XR1* expression in MBCs with and without *TBL1XR1-PIK3CA* fusion gene.**

Barplots illustrate the normalized expression values of (**a**) *PIK3CA* and (**b**) *TBL1XR1*.

**Supplementary Table 1: Immunohistochemical features, morphologic subtype, histologic grade, PAM50/ claudin-low molecular subtype classification and clinical information of metaplastic carcinomas of the breast.**

**Supplementary Table 2: Summary of clinical, pathologic and immunohistochemical features and molecular subtypes according to the histologic subtypes of the 17 metaplastic breast cancers included in the study.**

**Supplementary Table 3: Summary of chromosomal regions differentially gained, lost, amplified or deleted between 17 MBCs and 34 TNBCs.**

Protein coding genes and microRNAs mapping to these regions are listed. Chrom: chromosome; MB: megabase.

**Supplementary Table 4: Recurrent focal amplifications and homozygous deletions in metaplastic carcinomas of the breast.**

Focal amplifications were defined as amplification/high-level gain that span <25% of the chromosome arm. Recurrent homozygous deletions of *PTEN* and *CDKN2A/B* were defined based on SNP6.0 data using ABSOLUTE and ASCAT algorithms.

**Supplementary Table 5: Summary of chromosomal regions differentially gained, lost, amplified or deleted between copy number cluster 1 (CN1) and copy number cluster 2 (CN2).**

Protein coding genes and microRNAs mapping to these regions are listed. Chrom: chromosome; MB: megabase.

**Supplementary Table 6: Summary of chromosomal regions differentially gained, lost, amplified or deleted between chondroid and non-chondroid tumors.**

Protein coding genes and microRNAs mapping to these regions are listed. Chrom: chromosome; MB: megabase.

**Supplementary Table 7: Summary of chromosomal regions differentially gained, lost, amplified or deleted between spindle and non-spindle tumors.**

Protein coding genes and microRNAs mapping to these regions are listed. Chrom: chromosome; MB: megabase.

**Supplementary Table 8: Summary of chromosomal regions differentially gained, lost, amplified or deleted between squamous and non-squamous tumors.**

Protein coding genes and microRNAs mapping to these regions are listed. Chrom: chromosome; MB: megabase.

**Supplementary Table 9: List of 190 differentially expressed transcripts in non-spindle and spindle tumors identified by SAM of the gene expression microarrays.**

**Supplementary Table 10: Pathway analysis of genes differentially expressed between spindle and non-splindle tumors using g:Profiler.**

**Supplementary Table 11: List of 48 differentially expressed transcripts in non-chondroid vs chondroid tumors identified by SAM of the gene expression microarrays.**

**Supplementary Table 12: List of 19 differentially expressed transcripts in non-squamous vs squamous tumors identified by SAM of the gene expression microarrays.**

**Supplementary Table 13: Summary of differentially expressed genes between spindle and non-spindle MBCs according to RNA-sequencing.**

Statistics calculated using the limma package.[^47^](#_ENREF_47) Genes whose differences in expression levels reached statistical significance (unadjusted p-value <0.05) have been included.

**Supplementary Table 14: Summary of differentially expressed genes between chondroid and non-chondroid MBCs according to RNA-sequencing.**

Statistics calculated using the limma package.[^47^](#_ENREF_47) Genes with p-value <0.05 have been included.

**Supplementary Table 15: Summary of differentially expressed genes between squamous and non-squamous MBCs according to RNA-sequencing.**

Statistics calculated using the limma package.[^47^](#_ENREF_47) Genes with p-value <0.05 have been included.

**Supplementary Table 16: List of transcripts for which gene expression significantly correlates with gene copy number.**

**Supplementary Table 17: List of genes overexpressed when amplified in metaplastic carcinomas of the breast.**

**Supplementary Table 18: Pathway analysis of the genes overexpressed when amplified using g:Profiler.**

**Supplementary Table 19: Fusion transcripts identified by deFuse and/ or ChimeraScan.**

**Supplementary Table 20: List of non-synonymous mutations in cancer genes identified from RNA-sequencing analysis of 17 metaplastic carcinomas of the breast.**

**Supplementary Table 21: Summary of antibodies, clones, dilutions and antigen retrieval methods.**

**Supplementary Table 22: Oligonucleotide primers used for the validation of fusion transcripts by reverse transcription PCR (RT-PCR).**
