# Supplementary material for: Genomic and transcriptomic heterogeneity in metaplastic carcinomas of the breast
Source: NPJ Breast Cancer. 2017 Dec 1;3:48. doi: 10.1038/s41523-017-0048-0 (PMC5711926; doi:10.1038/s41523-017-0048-0)
Supplement: Supplementary file 13 — Supplementary Table 1 [file 41523_2017_48_MOESM13_ESM.pdf]

Supplementary Table 1: Immunohistochemical features, morphologic subtype, histologic grade, PAM50/ claudin-low molecular subtype classification and clinical information of metaplastic carcinomas of the breast.

Sample ID	Histologic Subtype	Molecular Subtype*	Tumor cell content (%)**	Histologic grade***	Triple-negative phenotype****	CK5/6	EGFR	CK14	CK17	PTEN	p53	c-KIT	p63	Age at diagnosis	Tumor size (cm)	pT stage	pN stage	M stage
META30	Chondroid	Basal-like	60	3	yes	1+ (<1%)	1+	3+ (>90%)	1+ (<1%)	1+ (10%)	0	2+ (80%)	0	71	4.5	T2	N0	M0
META31	Chondroid	Basal-like	80	2	yes	3+ (15%)	NP	3+ (90%)	1+ (<1%)	1+ (>90%)	NP	NP	NP	59	1.2	T1c	N0	M0
META32	Spindle	Claudin-low	80	3	yes	0	3+	0	0	0	NP	2+ (80%)	0	56	24	T4b	N2a	NA
META36	Chondroid	Normal breast-like	80	2	yes	3+ (80%)	1+	3+ (30%)	1+ (<1%)	2+ (80%)	NP	1+ (30%)	NP	74	3.2	T2	N0	M0
META37	Squamous	Normal breast-like	80	3	yes	3+ (50%)	2+	3+ (90%)	1+ (30%)	1+ (>90%)	3+ (>90%)	1+ (>90%)	0	33	1.4	T1c	N0	M0
META39	Spindle	Claudin-low	80	3	yes	0	1+	0	0	2+ (40%)	3+ (80%)	2+ (60%)	1+ (<1%)	45	5.5	T3	N0	M0
META40	Squamous	Claudin-low	90	3	yes	2+ (70%)	2+	3+ (90%)	2+ (80%)	1+ (>90%)	3+ (>90%)	NP	0	42	4	T2	N0	M0
META41	Squamous	Basal-like	80	3	yes	2+ (80%)	3+	3+ (30%)	2+ (60%)	0	3+ (>90%)	1+ (70%)	2+ (60%)	57	2.5	T2	N0	M0
META42	Squamous	Basal-like	70	3	yes	3+ (10%)	3+	3+ (30%)	3+ (20%)	2+ (>90%)	3+ (>90%)	2+ (30%)	0	48	3.1	T2	N0	M0
META47	Spindle	Claudin-low	70	3	yes	3+ (90%)	2+	2+ (80%)	2+ (80%)	1+ (>90%)	3+ (60%)	1+ (<1%)	1+ (90%)	73	2	T1c	N0	M0
META49	Spindle	Claudin-low	80	3	yes	3+ (80%)	2+	3+ (70%)	2+ (60%)	0	1+ (<1%)	NP	1+ (50%)	77	4	T2	N0	M0
META52	Chondroid	Claudin-low	95	3	yes	3+ (<1%)	3+	2+ (<1%)	0	2+ (>90%)	NP	1+ (20%)	0	59	3.3	T2	N0	M0
META53	Chondroid	Basal-like	60	3	yes	3+ (>90%)	NP	3+ (10%)	0	2+ (>90%)	NP	NP	NP	71	1.2	T1c	N0	M0
META55	Chondroid	Normal breast-like	80	3	yes	1+ (<1%)	1+	3+ (25%)	1+ (<1%)	1+ (>90%)	2+ (50%)	2+ (30%)	0	50	9	T3	N1a	M0
META59	Spindle	Claudin-low	90	3	yes	0	2+	2+ (<1%)	0	2+ (20%)	2+ (60%)	1+ (25%)	0	53	6	T3	N0	M0
META62	Squamous	Claudin-low	80	3	yes	3+ (60%)	2+	3+ (60%)	2+ (10%)	0	NP	1+ (15%)	1+ (20%)	77	5	T2	N0	M0
META64	Chondroid	Basal-like	70	3	yes	0	2+	2+ (70%)	0	2+ (>90%)	2+ (80%)	1+ (70%)	0	73	1.7	T1c	N0	M0

CK, cytokeratin; EGFR: epidermal growth factor receptor; NP, not performed; *, PAM50/ Claudin-low molecular subtype classification was retrieved from Weigelt et al. **, tumor cell content as defined by pathology review; ***, according to Nottingham grading index; ****, triple-negative phenotype: lack of estrogen receptor, progesterone receptor and HER2 expression. Immunohistochemical results were scored semi-quantitatively based on intensity (0: no staining; 1+: weak; 2+: moderate; 3+: strong) and percentage of positive cells in parentheses. For PTEN expression, normal epithelium and stroma served as an internal control, and tumor immunoreactivity was scored using a semi-quantitative system: score 0 = no immunoreaction; score 1 = reduced intensity of immunoreaction compared to normal epithelium; and score 2 = intensity equal to normal epithelium.
