# Supplementary material for: Genomic and transcriptomic heterogeneity in metaplastic carcinomas of the breast
Source: NPJ Breast Cancer. 2017 Dec 1;3:48. doi: 10.1038/s41523-017-0048-0 (PMC5711926; doi:10.1038/s41523-017-0048-0)
Supplement: Supplementary file 14 — Supplementary Table 2 [file 41523_2017_48_MOESM14_ESM.pdf]

Supplementary Table 2: Summary of clinical, pathologic and immunohistochemical features and molecular subtypes according to the histologic subtypes of the 17 metaplastic breast cancers included in the study.

		Histologic Subtype		
		Chondroid (n=7)	Spindle (n=5)	Squamous (n=5)
**Median age at diagnosis (range)**		71 (50-74)	56 (45-77)	48 (33-77)
**Median tumor size (cm, range)**		3.2 (1.2-9.0)	5.5 (2.0-24)	3.1 (1.4-5.0)
**pT stage**	**1c**	3	1	1
	**2**	3	1	4
	**3**	1	2	0
	**4b**	0	1	0
**pN stage**	**0**	6	4	5
	**1a**	1	0	0
	**2a**	0	1	0
**M stage**	**0**	7	4	5
	**N/A**	0	1	0
**Histologic grade***	**2**	2	0	0
	**3**	5	5	5
**Triple-negative phenotype****	**No**	0	0	0
	**Yes**	7	5	5
**CK5/6**	**0**	1	3	0
	**1+**	2	0	0
	**2+**	0	0	2
	**3+**	4	2	3
**EGFR**	**0**	0	0	0
	**1+**	3	1	0
	**2+**	1	3	3
	**3+**	1	1	2
	**NP**	2	0	0
**CK14**	**0**	0	2	0
	**1+**	0	0	0
	**2+**	2	2	0
	**3+**	5	1	5
**CK17**	**0**	3	3	0
	**1+**	4	0	1
	**2+**	0	2	3
	**3+**	0	0	1
**PTEN**	**0**	0	2	2
	**1+**	3	1	2
	**2+**	4	2	1
**p53**	**0**	1	0	0
	**1+**	0	1	0
	**2+**	2	1	0
	**3+**	0	2	4
	**NP**	4	1	1
**c-KIT**	**0**	0	0	0
	**1+**	3	2	3
	**2+**	2	2	1
	**3+**	0	0	0
	**NP**	2	1	1
**p63**	**0**	4	2	3
	**1+**	0	3	1
	**2+**	0	0	1
	**3+**	0	0	0
	**NP**	3	0	0
**Molecular Subtype*****	**Basal-like**	4	0	2
	**Claudin-low**	1	5	2
	**Normal breast-like**	2	0	1

CK, cytokeratin; EGFR: epidermal growth factor receptor; NP, not performed; *, according to Nottingham grading index; **, triple-negative phenotype: lack of estrogen receptor, progesterone receptor and HER2 expression;***, PAM50/ Claudin-low molecular subtype classification was retrieved from Weigelt et al. Immunohistochemical results were scored semi-quantitatively based on intensity (0: no staining; 1+: weak; 2+: moderate; 3+: strong) and percentage of positive cells in parentheses. For PTEN expression, normal epithelium and stroma served as an internal control, and tumor immunoreactivity was scored using a semi-quantitative system: score 0 = no immunoreaction; score 1 = reduced intensity of immunoreaction compared to normal epithelium; and score 2 = intensity equal to normal epithelium.
