# Supplementary material for: Genomic and transcriptomic heterogeneity in metaplastic carcinomas of the breast
Source: NPJ Breast Cancer. 2017 Dec 1;3:48. doi: 10.1038/s41523-017-0048-0 (PMC5711926; doi:10.1038/s41523-017-0048-0)
Supplement: Supplementary file 15 — Supplementary Table 3 [file 41523_2017_48_MOESM15_ESM.pdf]

Supplementary Table 3: Summary of chromosomal regions differentially gained, lost, amplified or deleted between 17 MBCs and 34 TNBCs. Protein coding genes and microRNAs mapping to these regions are listed. Chrom: chromosome; MB: megabase.

Regions differentially gained in MBCs and TNBCs

Chromosome	Cytobands	Start MB	End MB	Start Probe	End Probe	BACs	Length MB	maxM	Max.Overlap	Cases	Gains in Metaplastic	Gains in TNBC	Genes	miRNAs
3	q26.32	178.89	178.89	CN_981449	CN_239983	5	0.000222	1.39	22	20, 21, 22, 24, 27, 28, 29, 31, 33, 35, 36, 38, 39, 43, 48, 50, 51, 52, 53, 54, 56, 57	0	22	PIK3CA	
10	p15.3	0.35	0.44	CN_506571	SNP_A-8492212	38	0.087	2.14	29	7, 14, 18, 19, 20, 22, 25, 28, 29, 30, 33, 34, 35, 36, 37, 38, 40, 41, 42, 43, 47, 48, 49, 50, 52, 53, 54, 55, 56	2	27	DIP2C	

Regions differentially lost in MBCs and TNBCs

Chromosome	Cytobands	Start MB	End MB	Start Probe	End Probe	BACs	Length MB	maxM	Max.Overlap	Cases	Losses in Metaplastic	Losses in TNBC	Genes	miRNAs
1	q23.1	158.51	158.52	CN_450937	CN_450944	9	0.000581	-2.2	10	2, 3, 4, 5, 6, 9, 13, 15, 41, 42	8	2		
1	q31.1	190.56	190.56	SNP_A-8620594	SNP_A-4210207	5	0.00107	-1.9	19	20, 23, 26, 27, 28, 30, 34, 36, 37, 39, 40, 41, 43, 46, 49, 50, 51, 54, 57	0	19		
1	q31.2	192.59	192.59	CN_474280	SNP_A-8389098	4	0.000571	-1.9	11	1, 3, 4, 5, 6, 7, 8, 10, 12, 13, 16	11	0		
1	q31.2	193.21	193.21	CN_012041	CN_478810	4	0.000108	-2.7	20	1, 2, 3, 4, 5, 6, 7, 8, 9, 10, 11, 12, 13, 14, 16, 17, 34, 40, 51, 53	16	4	CDC73	
2	p16.1	55.63	55.63	SNP_A-8403428	SNP_A-8589243	3	0.000298	-1.89	8	5, 8, 9, 10, 13, 14, 16, 26	7	1	CCDC88A	
3	q26.32	178.89	178.89	CN_981454	CN_981453	7	0.000267	-4.74	17	1, 2, 3, 5, 6, 7, 8, 9, 10, 12, 13, 14, 15, 16, 17, 26, 45	15	2	PIK3CA	
4	q12	55.12	55.12	CN_1065114	CN_1065119	6	8.20E-05	-2.12	34	7, 13, 18, 19, 20, 22, 23, 24, 25, 26, 27, 28, 29, 30, 31, 32, 33, 34, 35, 36, 38, 39, 40, 42, 43, 45, 47, 49, 50, 51, 52, 53, 55, 56	2	32	PDGFRA	
5	p13.3	30.63	30.63	SNP_A-8423901	CN_1123395	3	0.000466	-3	20	1, 2, 3, 4, 5, 6, 7, 8, 9, 11, 12, 15, 16, 27, 38, 45, 47, 52, 53, 56	13	7		
6	q13-q14.1	74.51	76.64	CN_1215496	CN_1193540	955	2.131892	-3.98	8	1, 6, 9, 11, 12, 13, 15, 26	7	1	CD109, COL12A1, COX7A2, TMEM30A, FILIP1, SENP6, MYO6, IMPG1	
6	q14.1	76.67	79.54	CN_1193551	SNP_A-1859272	1057	2.869304	-4.53	9	1, 6, 9, 11, 12, 13, 15, 17, 26	8	1	IMPG1, HTR1B	
6	q16.3	102.91	102.94	CN_1138168	SNP_A-8549211	12	0.03119	-1.99	14	1, 2, 3, 4, 6, 7, 8, 10, 11, 12, 13, 15, 16, 26	13	1		
6	q16.3	104.22	104.22	CN_1147136	SNP_A-2230662	4	0.00137	-1.85	8	1, 6, 11, 12, 13, 14, 15, 26	7	1		
6	q16.3	105.07	105.2	SNP_A-4237086	CN_1151639	73	0.122022	-2.25	8	1, 6, 11, 12, 13, 14, 15, 26	7	1	HACE1	
6	q21	106.4	106.82	SNP_A-8524059	CN_1129306	257	0.423087	-2.87	8	1, 8, 11, 12, 13, 14, 15, 26	7	1	PRDM1, ATG5	
6	q21	107.21	107.31	SNP_A-8352698	CN_1131582	72	0.095646	-2.66	8	1, 8, 11, 12, 13, 14, 15, 50	7	1		hsa-mir-587
7	p22.3	0.88	0.89	SNP_A-2216384	CN_1227403	3	0.000416	-2.69	32	7, 18, 19, 20, 22, 23, 26, 28, 29, 30, 32, 33, 34, 35, 36, 37, 38, 39, 40, 41, 43, 45, 46, 47, 49, 50, 51, 52, 53, 54, 56, 57	1	31	UNC84A	
10	p15.3	0.22	1.31	CN_501962	SNP_A-8327028	365	1.087704	-2.44	13	1, 3, 6, 8, 11, 12, 13, 15, 16, 24, 26, 39, 45	9	4	ZMYND11, DIP2C, C10orf108, LARP5, GTPBP4, IDI2, IDI1, WDR37, ADARB2	
10	p15.3-p15.1	1.35	4.24	CN_037495	SNP_A-2053864	1840	2.888367	-3.67	15	1, 2, 3, 4, 6, 8, 11, 12, 13, 14, 15, 16, 17, 24, 26, 35, 39, 45	13	5	ADARB2, PFKP, PITRM1, KLF6	
10	p15.1-p14	4.24	10.61	SNP_A-2041266	SNP_A-1995038	3340	6.366198	-3.63	11	1, 3, 6, 11, 12, 13, 15, 16, 17, 24, 35	9	2	AKR1CL2, AKR1C1, AKR1C2, AKR1C3, AKR1CL1, AKR1C4, UCN3, TUBAL3, NET1, CALML5, CALML3, ASB13, C10orf18, GDI2, ANKRD16, FBXO18, IL15RA, IL2RA, RBM17, PFKFB3, PRKCQ, SFMBT2, ITIH5, ITIH2, KIN, ATP5C1, TAF3, GATA3	
10	p14-p13	10.63	15.22	CN_496683	CN_501774	2866	4.586529	-3.77	12	1, 3, 6, 11, 12, 13, 15, 16, 17, 24, 35, 46, 54	9	4	CUGBP2, C10orf31, USP6NL, ECHDC3, C10orf47, UPF2, DHTKD1, SEC61A2, NUDT5, CDC123, CAMK1D, CCDC3, OPTN, MCM10, UCMA, PHYH, SEPHS1, BEND7, PRPF18, FRMD4A, FAM107B, ARMETL1, HSPA14, SUV39H2, DCLRE1C, MEIG1, OLAH, ACBD7, NMT2, C10orf111, RPP38	hsa-mir-1265

10	q11.21	43.62	45.04	CN_541545	CN_552165	992	1.419583	-4.44	15	1, 3, 7, 8, 9, 11, 13, 15, 16, 17, 24, 30, 42, 49, 50	10	5	RET, CSGALNACT2, RASGEF1A, FXYD4, HNRNPF, ZNF487, ZNF239, ZNF485, ZNF32, C10orf136, CXCL12	
10	q11.22-q11.23	49.45	52.09	SNP_A-8409113	SNP_A-8635227	1305	2.635664	-4.27	21	1, 3, 5, 7, 8, 9, 11, 13, 14, 15, 16, 17, 22, 24, 25, 30, 37, 38, 42, 45, 49, 50	12	10	FRMPD2, MAPK8, ARHGAP22, WDFY4, LRRC18, C10orf72, FAM170B, C10orf128, C10orf71, DRGX, ERCC6, CHAT, SLC18A3, C10orf53, OGDHL, PARG, FAM21D, AGAP8, TIMM23B, AGAP7, MSMB, NCOA4, TIMM23, AGAP6, FAM21A, ASAH2, SGMS1	
10	q11.23	52.28	52.5	CN_543752	SNP_A-8542166	45	0.217956	-5.1	21	1, 3, 5, 7, 8, 9, 11, 13, 14, 15, 16, 17, 22, 24, 25, 37, 38, 42, 45, 49, 50	12	9	SGMS1	
10	q11.23-q21.1	52.59	60.57	CN_545871	CN_526831	2162	7.984171	-5.29	24	1, 3, 5, 6, 7, 8, 9, 11, 13, 14, 15, 16, 17, 22, 24, 25, 31, 37, 38, 42, 45, 49, 50, 51	13	11	A1CF, PRKG1, CSTF2T, DKK1, MBL2, PCDH15, ZWINT, IPMK, CISD1, UBE2D1, TFAM, BICC1	hsa-mir-605, hsa-mir-548f-1
10	q21.3	66.52	66.52	SNP_A-8484096	CN_526964	3	0.00069	-2.52	27	1, 2, 3, 5, 6, 7, 8, 9, 11, 13, 14, 15, 16, 17, 25, 30, 31, 35, 37, 38, 39, 41, 42, 45, 49, 50, 56	14	13		
10	q21.3	68.51	68.53	CN_539895	SNP_A-2240253	13	0.020341	-2.1	27	1, 3, 5, 6, 7, 8, 9, 11, 12, 13, 14, 15, 16, 17, 25, 30, 31, 35, 37, 38, 39, 41, 42, 45, 49, 50, 56	14	13	CTNNA3	
11	q22.3	105.9	105.9	SNP_A-8654024	CN_545049	3	0.000385	-2.79	39	5, 6, 13, 14, 16, 18, 19, 20, 22, 23, 24, 26, 27, 28, 30, 31, 33, 34, 35, 36, 37, 39, 40, 41, 42, 43, 44, 45, 46, 47, 48, 50, 51, 52, 53, 54, 55, 56, 57	5	34		
12	p13.31	6.1	6.11	SNP_A-8474288	SNP_A-2192742	16	0.011005	-1.66	8	3, 6, 12, 13, 15, 16, 17, 32	7	1	VWF	
12	p13.31	7.35	7.54	CN_614371	SNP_A-2103790	88	0.188985	-1.74	10	3, 6, 12, 13, 14, 15, 16, 17, 32, 53	8	2	PEX5, ACSM4, CD163L1	
18	p11.31	4.98	4.98	SNP_A-1919216	CN_786211	6	0.00059	-2.66	12	1, 2, 3, 4, 5, 6, 7, 9, 13, 14, 47, 48	10	2		
18	q12.3	38.26	38.26	CN_781572	SNP_A-1881947	4	0.000151	-2.72	47	3, 4, 6, 8, 9, 12, 13, 14, 15, 18, 19, 20, 22, 23, 24, 26, 27, 28, 29, 30, 31, 32, 33, 34, 35, 36, 37, 38, 39, 40, 41, 42, 43, 44, 45, 46, 47, 48, 49, 50, 51, 52, 53, 54, 55, 56, 57	9	38		
