# Supplementary material for: Genomic and transcriptomic heterogeneity in metaplastic carcinomas of the breast
Source: NPJ Breast Cancer. 2017 Dec 1;3:48. doi: 10.1038/s41523-017-0048-0 (PMC5711926; doi:10.1038/s41523-017-0048-0)
Supplement: Supplementary file 16 — Supplementary Table 4 [file 41523_2017_48_MOESM16_ESM.pdf]

Supplementary Table 4: Recurrent focal amplifications and homozygous deletions in metaplastic carcinomas of the breast.
Focal amplifications were defined as amplification/high-level gain that span <25% of the chromosome arm. Recurrent homozygous deletions of PTEN and CDKN2A/B were called from SNP6.0 data using ABSOLUTE and ASCAT algorithms.

Sample ID	Type of alteration	Chromosome	Cytoband	Start bp	End bp	Genes
META30, META52	Focal amplification	1	q31.2	193156267	193213223	CDC73
META30, META52	Focal amplification	1	q32.1	205220075	205669107	TMCC2, NUAK2, KLHDC8A, LEMD1, PCTK3, MFSD4, ELK4, SLC45A3
META41, META52	Focal amplification	1	q41-q42.12	222458616	226397014	HHIPL2, TAF1A, MIA3, AIDA, C1orf58, FAM177B, DISP1, TLR5, SUSD4, C1orf65, CAPN8, CAPN2, TP53BP2, FBXO28, DEGS1, NVL, CNIH4, WDR26, CNIH3, DNAH14, LBR, C1orf197, ENAH, SRP9, EPHX1, TMEM63A, LEFTY1, LEFTY2, C1orf55, H3F3A, ACBD3
META41, META52	Focal amplification	1	q42.12-q42.2	226880320	232350999	ITPKB, PSEN2, CABC1, CDC42BPA, ZNF678, SNAP47, JMJD4, WNT9A, WNT3A, ARF1, C1orf35, MRPL55, GUK1, GJC2, C1orf148, C1orf69, C1orf145, OBSCN, TRIM11, TRIM17, HIST3H3, HIST3H2A, HIST3H2BB, RNF187, RHOU, TMEM78, RAB4A, SPHAR, C1orf96, ACTA1, NUP133, ABCB10, TAF5L, URB2, GALNT2, PGBD5, COG2, AGT, CAPN9, C1orf198, TTC13, ARV1, FAM89A, TRIM67, C1orf131, GNPAT, EXOC8, C1orf124, EGLN1, TSNAX, DISC1
META42, META52	Focal amplification	2	p25.3	12772	1895349	FAM110C, SH3YL1, ACP1, FAM150B, TMEM18, SNTG2, TPO, PXDN, MYT1L
META39, META42	Focal amplification	3	q26.2-q26.31	169200472	172102741	MYNN, LRRC34, LRRIQ4, LRRC31, SAMD7, SEC62, GPR160, PHC3, PRKCI, SKIL, CLDN11, SLC7A14, RPL22L1, EIF5A2, SLC2A2, TNIK, PLD1, TMEM212, FNDC3B
META37, META40	Focal amplification	6	q13	70039768	70186645	BAI3
META37, META40	Focal amplification	6	q13	71633091	73227808	B3GAT2, OGFRL1, RIMS1
META37, META40	Focal amplification	6	q13-q14.1	73231410	77369597	KCNQ5, KHDC1, DPPA5, C6orf221, OOEP, DDX43, C6orf150, MTO1, EEF1A1, SLC17A5, CD109, COL12A1, COX7A2, TMEM30A, FILIP1, SENP6, MYO6, IMPG1
META39, META41, META52	Focal amplification	7	q21.2-q22.1	92641588	98097063	SAMD9, SAMD9L, HEPACAM2, CCDC132, CALCR, GNGT1, TFPI2, GNG11, BET1, COL1A2, CASD1, SGCE, PPP1R9A, PON1, PON3, PON2, ASB4, PDK4, DYNC1I1, SLC25A13, SHFM1, DLX6, DLX5, ACN9, TAC1, ASNS, OCM2, LMTK2, BHLHA15, TECPR1, BRI3, BAIAP2L1
META31, META39	Focal amplification	8	p11.23-p11.22	37167287	38919419	ZNF703, ERLIN2, PROSC, GPR124, BRF2, RAB11FIP1, GOT1L1, ADRB3, EIF4EBP1, ASH2L, STAR, LSM1, BAG4, DDHD2, PPAPDC1B, WHSC1L1, LETM2, FGFR1, C8orf86, TACC1, PLEKHA2, HTRA4, TM2D2, ADAM9
META39, META42	Focal amplification	10	q22.3-q23.1	78914585	82140647	KCNMA1, DLG5, POLR3A, RPS24, ZMIZ1, PPIF, ZCCHC24, SFTPA2, SFTPA1, FAM22B, FAM22E, SFTPD, PLAC9, ANXA11, MAT1A, DYDC1, DYDC2
META39, META42	Focal amplification	10	q23.1	82160570	83608753	C10orf57, TSPAN14, SH2D4B
META41, META52	Focal amplification	12	q12	42789633	43514990	PPHLN1, PRICKLE1
META42, META49	Homozygous deletion	9	p21.3	21601230	22193708	MTAP, CDKN2A, CDKN2B
META32, META41	Homozygous deletion	10	q23.31	89626442	89921485	PTEN
