# Supplementary material for: Genomic and transcriptomic heterogeneity in metaplastic carcinomas of the breast
Source: NPJ Breast Cancer. 2017 Dec 1;3:48. doi: 10.1038/s41523-017-0048-0 (PMC5711926; doi:10.1038/s41523-017-0048-0)
Supplement: Supplementary file 17 — Supplementary Table 5 [file 41523_2017_48_MOESM17_ESM.pdf]

Supplementary Table 5: Summary of chromosomal regions differentially gained, lost, amplified or deleted between copy number cluster 1 (CN1) and copy number cluster 2 (CN2). Protein coding genes and microRNAs mapping to these regions are listed. Chrom: chromosome; MB: megabase.

Regions differentially gained in CN1 and CN2

Chrom	Start	End	Start MB	End MB	Start Probe	End Probe	BACs	Length MB	maxM	Max.Overlap	Cases	Gains in CN1	Gains in CN2	Genes	miRNAs	Cytobands
1	48911418	51981979	48.91	51.98	SNP_A-2011647	CN_476023	1448	3.070561	2.48	5	META37, META39, META40, META53, META62	1	4	SPATA6, AGBL4, BEND5, ELAVL4, DMRTA2, FAF1, CDKN2C, C1orf185, RNF11, TTC39A, EPS15		p33-p32.3
1	58482304	58484289	58.48	58.48	SNP_A-8677436	SNP_A-4260133	7	0.001985	0.617	5	META37, META39, META40, META53, META62	1	4	DAB1		p32.2
1	65412038	66588176	65.41	66.59	CN_515474	CN_522085	748	1.176138	2.01	5	META37, META39, META40, META53, META62	1	4	JAK1, AK3L1, DNAJC6, LEPR, LEPROT, PDE4B	hsa-mir-101-1	p31.3
1	66649676	75985788	66.65	75.99	SNP_A-8393631	CN_502582	5312	9.336112	6.48	5	META37, META39, META40, META53, META62	1	4	PDE4B, SGIP1, TCTEX1D1, INSL5, WDR78, MIER1, SLC35D1, C1orf141, IL23R, IL12RB2, SERBP1, GADD45A, GNG12, DIRAS3, GPR177, RPE65, DEPDC1, LRRC7, LRRC40, SFRS11, ANKRD13C, HHLA3, CTH, PTGER3, ZRANB2, NEGR1, LRRIQ3, TNNI3K, LRRC53, C1orf173, CRYZ, TYW3, LHX8, SLC44A5	hsa-mir-1262, hsa-mir-186	p31.3-p31.1
1	87614742	88480108	87.61	88.48	SNP_A-8333222	SNP_A-8299537	679	0.865366	4.84	5	META37, META39, META40, META53, META62	1	4	HS2ST1, LMO4		p22.3-p22.2
1	94577411	99001190	94.58	99	SNP_A-8382611	SNP_A-8680203	2262	4.423779	4.73	5	META37, META39, META40, META53, META62	1	4	ABCA4, ARHGAP29, ABCD3, F3, SLC44A3, CNN3, ALG14, TMEM56, RWDD3, PTBP2, DPYD	hsa-mir-137	p22.1-p21.3
1	103014793	107372413	103.01	107.37	CN_451610	CN_444901	1473	4.35762	2.43	5	META37, META40, META52, META53, META62	1	4	COL11A1, RNPC3, AMY2B, AMY1A, AMY1B, AMY1C		p21.1-p13.3
3	133512047	133538027	133.51	133.54	CN_1002049	SNP_A-8332142	20	0.02598	0.954	5	META37, META40, META52, META53, META62	1	4	SRPRB		q22.1
3	189759194	189759440	189.76	189.76	SNP_A-4300293	CN_1009912	3	0.000246	0.623	5	META37, META40, META52, META53, META62	1	4	LEPREL1		q28
13	97758621	112523977	97.76	112.52	CN_644347	SNP_A-8559562	8820	14.765356	6.53	5	META32, META37, META40, META53, META62	1	4	MBNL2, RAP2A, IPO5, FARP1, RNF113B, STK24, SLC15A1, DOCK9, UBAC2, GPR18, GPR183, TM9SF2, CLYBL, ZIC5, ZIC2, PCCA, A2LD1, TMTC4, NALCN, ITGBL1, FGF14, TPP2, C13orf39, C13orf27, KDELC1, BIVM, ERCC5, SLC10A2, DAOA, EFNB2, ARGLU1, FAM155A, LIG4, ABHD13, TNFSF13B, MYO16, IRS2, COL4A1, COL4A2, RAB20, CARKD, CARS2, ING1, C13orf29, ANKRD10, ARHGEF7, C13orf16	hsa-mir-623, hsa-mir-1267	q32.1-q34
X	95557035	96271505	95.56	96.27	SNP_A-8557073	SNP_A-8543944	461	0.71447	2.39	5	META37, META40, META49, META53, META62	1	4	DIAPH2, RPA4		q21.33

Regions differentially lost in CN1 and CN2

Chrom	Start	End	Start MB	End MB	Start Probe	End Probe	BACs	Length MB	maxM	Max.Overlap	Cases	Losses in CN1	Losses in CN2	Genes	miRNAs	Cytobands
1	65396403	65406593	65.4	65.41	CN_515459	CN_515463	6	0.01019	-3.59	6	META36, META37, META40, META42, META53, META62	2	4	JAK1		p31.3
1	159186548	159186793	159.19	159.19	CN_453661	SNP_A-2081184	3	0.000245	-1.81	6	META30, META32, META37, META40, META53, META62	2	4			q23.2
2	10510364	10521138	10.51	10.52	CN_801053	SNP_A-8478959	14	0.010774	-0.957	3	META40, META53, META62	0	3	HPCAL1		p25.1
2	12745408	14038445	12.75	14.04	SNP_A-1918294	CN_808443	454	1.293037	-3.91	3	META40, META53, META62	0	3	TRIB2		p24.3
2	14046577	20710425	14.05	20.71	SNP_A-2060147	CN_838317	3353	6.663848	-4.47	3	META40, META53, META62	0	3	FAM84A, NBAS, DDX1, MYCN, FAM49A, RAD51AP2, VSNL1, SMC6, GEN1, MSGN1, KCNS3, NT5C1B, OSR1, TTC32, WDR35, MATN3, LAPTM4A, SDC1, PUM2, RHOB		p24.3-p24.1
2	20714553	36240162	20.71	36.24	CN_838332	SNP_A-1962709	5266	15.525609	-4.43	4	META37, META40, META53, META62	0	4	HS1BP3, GDF7, C2orf43, APOB, KLHL29, ATAD2B, UBXN2A, C2orf44, FKBP1B, TP53I3, PFN4, C2orf84, ITSN2, NCOA1, C2orf79, CENPO, ADCY3, DNAJC27, EFR3B, POMC, DNMT3A, DTNB, ASXL2, KIF3C, RAB10, FAM59B, HADHA, HADHB, GPR113, C2orf39, OTOF, C2orf70, CIB4, KCNK3, C2orf18, CENPA, DPYSL5, MAPRE3, TMEM214, AGBL5, EMILIN1, KHK, CGREF1, ABHD1, PREB, C2orf53, TCF23, SLC5A6, C2orf28, CAD, SLC30A3, DNAJC5G, TRIM54, UCN, MPV17, GTF3C2, EIF2B4, SNX17, ZNF513, PPM1G, NRBP1, KRTCAP3, IFT172, FNDC4, GCKR, C2orf16, GPN1, CCDC121, SUPT7L, SLC4A1AP, MRPL33, RBKS, FOSL2, PLB1, PPP1CB, SPDYA, TRMT61B, WDR43, FAM179A, C2orf71, CLIP4, ALK, YPEL5, LBH, LCLAT1, CAPN13, GALNT14, CAPN14, EHD3, XDH, SRD5A2, DPY30, MEMO1, SPAST, SLC30A6, NLRC4, YIPF4, BIRC6, TTC27, LTBP1, RASGRP3, FAM98A, MYADML	hsa-mir-1301, hsa-mir-558	p24.1-p22.3
2	56906248	57144898	56.91	57.14	SNP_A-2093454	CN_862236	154	0.23865	-4.71	3	META37, META53, META62	0	3			p16.1
2	57687297	58388708	57.69	58.39	CN_866658	SNP_A-2053863	320	0.701411	-2.84	3	META40, META53, META62	0	3	VRK2, FANCL		p16.1
2	63841101	65333977	63.84	65.33	SNP_A-1897196	CN_837799	605	1.492876	-3.63	3	META40, META53, META62	0	3	UGP2, VPS54, PELI1, AFTPH, SERTAD2, SLC1A4, CEP68, RAB1A		p15-p14
2	66298357	70655625	66.3	70.66	CN_842319	SNP_A-2083173	2241	4.357268	-3.74	3	META40, META53, META62	0	3	MEIS1, ETAA1, C1D, WDR92, PNO1, CNRIP1, PLEK, FBXO48, APLF, PROKR1, ARHGAP25, BMP10, GKN2, GKN1, ANTXR1, GFPT1, NFU1, AAK1, ANXA4, GMCL1, SNRNP27, MXD1, ASPRV1, PCBP1, C2orf42, TIA1, PCYOX1, SNRPG, FAM136A	hsa-mir-1285-2	p14-p13.3
2	70668879	71125453	70.67	71.13	CN_866950	SNP_A-2205632	372	0.456574	-1.96	3	META40, META53, META62	0	3	TGFA, ADD2, FIGLA, CLEC4F, CD207		p13.3
2	73542673	75434258	73.54	75.43	SNP_A-4282997	CN_855908	428	1.891585	-3.11	3	META40, META53, META62	0	3	ALMS1, NAT8, TPRKB, DUSP11, C2orf78, STAMBP, ACTG2, DGUOK, TET3, BOLA3, MOBKL1B, MTHFD2, SLC4A5, DCTN1, C2orf81, WDR54, RTKN, INO80B, MOGS, MRPL53, CCDC142, TTC31, LBX2, PCGF1, TLX2, DQX1, AUP1, HTRA2, LOXL3, DOK1, C2orf65, SEMA4F, HK2, POLE4, TACR1		p13.1-p12
2	75586908	77216439	75.59	77.22	CN_858047	CN_867123	543	1.629531	-2.21	3	META40, META53, META62	0	3	FAM176A, MRPL19, C2orf3, LRRTM4		p12
2	77226075	79544348	77.23	79.54	SNP_A-8455990	SNP_A-8546240	923	2.318273	-4.98	3	META40, META53, META62	0	3	LRRTM4, REG3G, REG1B, REG1A, REG3A		p12
2	79722285	86835981	79.72	86.84	SNP_A-4201790	SNP_A-8469950	2069	7.113696	-3.66	3	META40, META53, META62	0	3	CTNNA2, LRRTM1, SUCLG1, DNAH6, TMSB10, KCMF1, TCF7L1, TGOLN2, RETSAT, ELMOD3, CAPG, SH2D6, MAT2A, GGCX, VAMP8, VAMP5, RNF181, TMEM150, C2orf68, USP39, SFTPB, GNLY, ATOH8, ST3GAL5, POLR1A, PTCD3, IMMT, MRPL35, REEP1, KDM3A, VPS24		p12-p11.2
2	98350334	98575543	98.35	98.58	SNP_A-8317608	SNP_A-8591538	109	0.225209	-2.35	3	META40, META53, META62	0	3	ZAP70, TMEM131		q11.2
2	99980851	100097909	99.98	100.1	CN_891709	CN_893800	64	0.117058	-1.39	3	META40, META53, META62	0	3	EIF5B, REV1		q11.2
2	106108994	110290172	106.11	110.29	CN_803418	SNP_A-2282351	1690	4.181178	-3.45	6	META30, META37, META40, META52, META53, META62	2	4	NCK2, C2orf40, UXS1, PLGLA1, RGPD3, CD8BP, ST6GAL2, RGPD4, SLC5A7, SULT1C3, SULT1C2, SULT1C4, GCC2, LIMS1, RANBP2, CCDC138, EDAR, SH3RF3		q12.2-q13
2	111892369	111897818	111.89	111.9	CN_831809	CN_831812	9	0.005449	-0.636	6	META30, META37, META40, META41, META53, META62	2	4	BCL2L11		q13
2	112814006	114074622	112.81	114.07	CN_836166	SNP_A-2133596	774	1.260616	-3.48	6	META30, META37, META40, META41, META53, META62	2	4	TMEM87B, FBLN7, ZC3H8, ZC3H6, RGPD8, TTL, POLR1B, CHCHD5, SLC20A1, NT5DC4, CKAP2L, IL1A, IL1B, IL1F7, IL1F9, IL1F6, IL1F8, IL1F5, IL1F10, IL1RN, PSD4, PAX8		q13
2	114436294	118522235	114.44	118.52	CN_807886	SNP_A-8367745	1502	4.085941	-3.23	6	META30, META37, META40, META41, META53, META62	2	4	SLC35F5, ACTR3, DPP10		q14.1
2	118530084	121073474	118.53	121.07	SNP_A-2271273	SNP_A-8466013	1468	2.54339	-2.53	6	META30, META37, META40, META41, META53, META62	2	4	DDX18, CCDC93, INSIG2, EN1, MARCO, C1QL2, STEAP3, C2orf76, DBI, TMEM37, SCTR, TMEM177, PTPN4, EPB41L5, RALB		q14.1-q14.2
2	122934639	128970075	122.93	128.97	SNP_A-4230757	CN_819107	3415	6.035436	-3.64	6	META30, META37, META40, META41, META53, META62	2	4	CNTNAP5, GYPC, BIN1, CYP27C1, ERCC3, MAP3K2, PROC, IWS1, MYO7B, LIMS2, GPR17, SFT2D3, WDR33, POLR2D, AMMECR1L, SAP130, UGCGL1		q14.3
2	133198519	133253940	133.2	133.25	CN_804038	SNP_A-8313201	43	0.055421	-4.07	6	META30, META37, META40, META41, META53, META62	2	4	GPR39		q21.2
2	133262787	133515811	133.26	133.52	CN_806097	CN_806216	159	0.253024	-2.98	6	META30, META37, META40, META41, META53, META62	2	4	GPR39, LYPD1		q21.2
2	146956830	147014597	146.96	147.01	CN_812960	SNP_A-2149920	36	0.057767	-1.21	6	META37, META40, META41, META53, META55, META62	2	4			q22.3
2	157539287	160862726	157.54	160.86	CN_804552	SNP_A-2218060	1828	3.323439	-3.07	6	META30, META37, META40, META53, META55, META62	2	4	GALNT5, ERMN, CYTIP, ACVR1C, ACVR1, UPP2, CCDC148, PKP4, DAPL1, TANC1, WDSUB1, BAZ2B, Mar-07, LY75, PLA2R1		q24.1-q24.2
2	175471595	175522288	175.47	175.52	SNP_A-8311874	CN_835334	33	0.050693	-1.71	6	META30, META37, META39, META40, META53, META62	2	4	WIPF1		q31.1
2	183053632	187276422	183.05	187.28	SNP_A-8576904	CN_835542	1203	4.22279	-4.27	6	META30, META37, META40, META53, META55, META62	2	4	PDE1A, DNAJC10, FRZB, NCKAP1, DUSP19, NUP35, ZNF804A, FSIP2		q32.1
2	187682917	188907407	187.68	188.91	CN_802974	SNP_A-8641640	411	1.22449	-2.96	6	META30, META37, META40, META53, META55, META62	2	4	ZSWIM2, CALCRL, TFPI		q32.1
2	188935819	197189193	188.94	197.19	CN_185937	SNP_A-8545940	3946	8.253374	-9.3	6	META30, META37, META40, META53, META55, META62	2	4	GULP1, DIRC1, COL3A1, COL5A2, WDR75, SLC40A1, ASNSD1, ANKAR, OSGEPL1, ORMDL1, PMS1, MSTN, C2orf88, HIBCH, INPP1, MFSD6, TMEM194B, NAB1, GLS, STAT1, STAT4, MYO1B, OBFC2A, SDPR, TMEFF2, SLC39A10, DNAH7, STK17B, HECW2	hsa-mir-561, hsa-mir-1245	q32.1-q32.3
2	197195087	201543508	197.2	201.54	SNP_A-2191649	SNP_A-8567200	2547	4.348421	-4.4	6	META30, META37, META40, META53, META55, META62	2	4	HECW2, CCDC150, GTF3C3, C2orf66, PGAP1, ANKRD44, SF3B1, COQ10B, HSPD1, HSPE1, MOBKL3, RFTN2, MARS2, BOLL, PLCL1, SATB2, C2orf69, C2orf60, C2orf47, KCTD18, SGOL2, AOX1		q32.3-q33.1
2	205843291	206240447	205.84	206.24	SNP_A-8555693	CN_805467	130	0.397156	-2.46	6	META37, META40, META52, META53, META55, META62	2	4	PARD3B		q33.3
2	208153004	216838943	208.15	216.84	CN_847260	SNP_A-1904332	4219	8.685939	-4.63	6	META30, META37, META40, META53, META55, META62	2	4	CREB1, FAM119A, CCNYL1, FZD5, PLEKHM3, CRYGD, CRYGC, CRYGB, CRYGA, C2orf80, IDH1, PIKFYVE, PTH2R, MAP2, C2orf21, RPE, C2orf67, ACADL, MYL1, LANCL1, CPS1, ERBB4, IKZF2, SPAG16, BARD1, ABCA12, ATIC, FN1, MREG	hsa-mir-548f-2	q33.3-q35
2	216840441	217246449	216.84	217.25	SNP_A-4262533	CN_860973	292	0.406008	-2.13	6	META30, META31, META37, META40, META53, META62	2	4	MREG, PECR, TMEM169, XRCC5, Mar-04		q35
2	220747466	225246060	220.75	225.25	CN_845293	SNP_A-8374617	2664	4.498594	-4.28	6	META30, META37, META40, META41, META53, META62	2	4	EPHA4, PAX3, CCDC140, SGPP2, FARSB, MOGAT1, ACSL3, KCNE4, SCG2, AP1S3, WDFY1, MRPL44, SERPINE2, FAM124B		q35-q36.2
2	226205732	227021979	226.21	227.02	CN_838816	SNP_A-8605192	483	0.816247	-2.21	6	META30, META37, META40, META49, META53, META62	2	4			q36.3
2	227821077	228431059	227.82	228.43	CN_847804	CN_852255	359	0.609982	-1.95	6	META30, META37, META40, META41, META53, META62	2	4	RHBDD1, COL4A4, COL4A3, MFF, TM4SF20, AGFG1		q36.3
2	228437778	231499779	228.44	231.5	SNP_A-1851308	CN_870148	1145	3.062001	-3	6	META30, META37, META40, META41, META53, META62	2	4	C2orf83, SLC19A3, CCL20, WDR69, SPHKAP, PID1, DNER, TRIP12, FBXO36, SLC16A14, SP110, SP140, SP140L, SP100		q36.3-q37.1
2	231509917	235046140	231.51	235.05	CN_192450	CN_852400	1546	3.536223	-2.67	6	META30, META37, META40, META41, META53, META62	2	4	CAB39, ITM2C, GPR55, SPATA3, C2orf72, PSMD1, HTR2B, ARMC9, B3GNT7, NCL, NMUR1, C2orf57, PTMA, PDE6D, COPS7B, NPPC, DIS3L2, ALPP, ALPPL2, ALPI, ECEL1, CHRND, CHRNG, TIGD1, EIF4E2, EFHD1, GIGYF2, KCNJ13, C2orf82, NGEF, NEU2, INPP5D, ATG16L1, SAG, DGKD, USP40, UGT1A6, HEATR7B1, HJURP, TRPM8, SPP2	hsa-mir-1471, hsa-mir-562	q37.1
2	242167476	242419678	242.17	242.42	SNP_A-8517838	CN_856898	128	0.252202	-2.84	6	META30, META37, META40, META42, META53, META62	2	4	HDLBP, Sep-02, FARP2		q37.3
4	37489400	37509342	37.49	37.51	CN_1058198	SNP_A-8648687	8	0.019942	-1.07	6	META37, META40, META41, META53, META62, META64	2	4	C4orf19		p14
4	37527693	37989776	37.53	37.99	SNP_A-8554965	CN_1060422	375	0.462083	-3.08	6	META37, META40, META41, META53, META62, META64	2	4	C4orf19, RELL1, PGM2, TBC1D1		p14
4	38016045	38259463	38.02	38.26	CN_1060429	SNP_A-8447038	186	0.243418	-2.74	6	META37, META40, META41, META53, META62, META64	2	4	TBC1D1		p14
4	38263237	38787228	38.26	38.79	SNP_A-8699169	SNP_A-2100978	337	0.523991	-2.29	6	META37, META40, META41, META53, META62, META64	2	4	KLF3, TLR10		p14
4	38791728	49054692	38.79	49.05	CN_1064799	SNP_A-8586957	4834	10.262964	-4.76	6	META37, META40, META41, META53, META62, META64	2	4	TLR1, TLR6, FAM114A1, TMEM156, KLHL5, WDR19, RFC1, KLB, RPL9, LIAS, UGDH, C4orf34, UBE2K, PDS5A, N4BP2, RHOH, CHRNA9, RBM47, NSUN7, APBB2, UCHL1, LIMCH1, PHOX2B, TMEM33, WDR21B, SLC30A9, BEND4, SHISA3, ATP8A1, GRXCR1, KCTD8, YIPF7, GUF1, GNPDA2, GABRG1, GABRA2, COX7B2, GABRA4, GABRB1, COMMD8, ATP10D, CORIN, NFXL1, CNGA1, NIPAL1, TXK, TEC, SLAIN2, SLC10A4, ZAR1, FRYL, OCIAD1, OCIAD2	hsa-mir-574	p14-p11
4	53092664	54954443	53.09	54.95	CN_1054095	SNP_A-8386812	918	1.861779	-3.53	3	META40, META53, META62	0	3	USP46, RASL11B, SCFD2, FIP1L1, LNX1, CHIC2		q12
4	56025604	59156627	56.03	59.16	SNP_A-8467738	CN_1056459	1351	3.131023	-3.93	3	META40, META53, META62	0	3	SRD5A3, TMEM165, CLOCK, PDCL2, NMU, EXOC1, CEP135, KIAA1211, AASDH, PPAT, PAICS, SRP72, ARL9, HOPX, SPINK2, REST, C4orf14, POLR2B, IGFBP7		q12
4	59186665	60353189	59.19	60.35	CN_1056464	CN_1063036	616	1.166524	-2.53	3	META40, META53, META62	0	3			q12-q13.1
4	108600544	108602299	108.6	108.6	CN_1038693	SNP_A-8413775	3	0.001755	-2.6	6	META37, META40, META41, META42, META53, META62	2	4	PAPSS1		q25
4	118132117	120434416	118.13	120.43	CN_1023439	SNP_A-2178353	601	2.302299	-3.61	6	META37, META40, META41, META52, META53, META62	2	4	NDST3, PRSS12, CEP170L, METTL14, SEC24D, SYNPO2, MYOZ2, USP53, FABP2, PDE5A		q26
4	165227300	165437409	165.23	165.44	CN_1055026	SNP_A-8303236	142	0.210109	-2.72	6	META37, META40, META42, META53, META55, META62	2	4			q32.3
4	165496854	165518314	165.5	165.52	SNP_A-8663365	SNP_A-4286586	23	0.02146	-1.15	6	META37, META40, META42, META53, META55, META62	2	4			q32.3
7	130414706	133506967	130.41	133.51	CN_1221152	SNP_A-8321378	1664	3.092261	-2.98	3	META37, META40, META62	0	3	KLF14, MKLN1, PODXL, PLXNA4, CHCHD3, EXOC4	hsa-mir-29a, hsa-mir-29b-1	q32.3-q33
7	141026965	141709706	141.03	141.71	CN_1216971	SNP_A-1991522	100	0.682741	-1.98	3	META37, META40, META62	0	3	AGK, KIAA1147, WEE2, SSBP1, TAS2R3, TAS2R4, TAS2R5, OR9A4, CLEC5A, TAS2R38, MGAM		q34
9	71949456	75280623	71.95	75.28	SNP_A-2296478	SNP_A-1921719	2280	3.331167	-4.04	6	META30, META37, META40, META42, META53, META62	2	4	C9orf61, APBA1, PTAR1, C9orf135, MAMDC2, SMC5, KLF9, TRPM3, C9orf63, TMEM2, FAM108B1, C9orf85, C9orf57, GDA, ZFAND5, TMC1	hsa-mir-204	q21.12-q21.13

9	75285891	75703661	75.29	75.7	SNP_A-8408962	CN_391821	229	0.41777	-2.64	6	META30, META37, META40, META42, META53, META62	2	4	TMC1, ALDH1A1		q21.13
9	75771675	100381046	75.77	100.38	CN_1305057	SNP_A-8299955	12910	24.609371	-4.52	6	ETA30, META37, META39, META40, META42, META53, META6	3	4	ANXA1, RORB, TRPM6, C9orf40, C9orf41, C9orf95, OSTF1, PCSK5, RFK, GCNT1, PRUNE2, FOXB2, VPS13A, GNA14, GNAQ, CEP78, PSAT1, CHCHD9, TLE4, TLE1, FAM75B, RASEF, FRMD3, C9orf103, UBQLN1, GKAP1, KIF27, C9orf64, HNRNPK, RMI1, SLC28A3, NTRK2, AGTPBP1, MAK10, GOLM1, C9orf153, ISCA1, ZCCHC6, GAS1, C9orf170, DAPK1, CTSL1, C9orf79, CCRK, SPIN1, NXNL2, C9orf47, S1PR3, SHC3, C9orf33, CKS2, SECISBP2, SEMA4D, GADD45G, DIRAS2, SYK, AUH, NFIL3, ROR2, SPTLC1, IARS, NOL8, CENPP, OGN, OMD, ASPN, ECM2, IPPK, BICD2, ANKRD19, ZNF484, FGD3, SUSD3, C9orf89, NINJ1, WNK2, C9orf129, FAM120AOS, FAM120A, PHF2, BARX1, PTPDC1, ZNF169, FAM22F, HIATL1, FBP2, FBP1, C9orf118, C9orf3, FANCC, PTCH1, C9orf102, HSD17B3, SLC35D2, ZNF367, HABP4, CDC14B, C9orf21, ZNF510, HIATL2, FAM22G, CTSL2, ZNF322B, KIAA1529, TDRD7, TMOD1, C9orf97	hsa-mir-7-1, hsa-let-7a-1, hsa-let-7f-1, hsa-let-7d, hsa-mir-23b, hsa-mir-27b, hsa-mir-24-1, hsa-mir-1302-8	q21.13-q22.33
9	101836774	103065972	101.84	103.07	SNP_A-8580632	CN_1330031	608	1.229198	-2.54	5	META37, META39, META40, META53, META62	1	4	TGFBR1, ALG2, SEC61B, KRT8P11, NR4A3, STX17, ERP44, INVS, TEX10		q22.33-q31.1
9	120264452	122009024	120.26	122.01	CN_1296788	CN_1308006	1188	1.744572	-4.03	6	META37, META39, META40, META52, META53, META62	2	4	TLR4, DBC1		q33.1
14	22172426	22207967	22.17	22.21	CN_105962	SNP_A-8331514	20	0.035541	-1.24	5	META37, META40, META42, META53, META62	1	4			q11.2
14	24601306	35780690	24.6	35.78	SNP_A-2082623	CN_696883	4568	11.179384	-4.7	6	ETA36, META37, META40, META42, META53, META55, META6	3	4	FITM1, PSME1, FAM158A, PSME2, RNF31, IRF9, REC8, IPO4, TM9SF1, TSSK4, CHMP4A, NEDD8, GMPR2, TINF2, TGM1, DHRS1, C14orf21, CIDEB, LTB4R2, LTB4R, ADCY4, RIPK3, NFATC4, KIAA1305, CBLN3, KIAA0323, SDR39U1, CMA1, CTSG, GZMH, GZMB, STXBP6, NOVA1, FOXG1, C14orf23, PRKD1, G2E3, SCFD1, COCH, STRN3, AP4S1, HECTD1, C14orf126, GPR33, NUBPL, C14orf128, ARHGAP5, AKAP6, NPAS3, EGLN3, C14orf147, EAPP, SNX6, CFL2, BAZ1A, SRP54, FAM177A1, PPP2R3C, KIAA0391, PSMA6	hsa-mir-624	q12-q13.2
14	38182269	38635746	38.18	38.64	SNP_A-8631588	SNP_A-4296098	250	0.453477	-1.9	6	META37, META40, META53, META55, META62, META64	2	4	TTC6		q21.1
14	38683735	43365114	38.68	43.37	CN_675191	SNP_A-2156712	1722	4.681379	-4.06	6	META37, META40, META53, META55, META62, META64	2	4	CLEC14A, SEC23A, SIP1, TRAPPC6B, PNN, MIA2, CTAGE5, FBXO33, LRFN5		q21.1
17	45278778	46597956	45.28	46.6	SNP_A-8600367	CN_750162	520	1.319178	-2.21	4	META37, META40, META53, META62	0	4	MYL4, ITGB3, C17orf57, NPEPPS, KPNB1, TBKBP1, TBX21, OSBPL7, MRPL10, LRRC46, SCRN2, SP6, SP2, PNPO, ATAD4, CDK5RAP3, COPZ2, NFE2L1, CBX1, SNX11, SKAP1	hsa-mir-152, hsa-mir-1203	q21.32
17	47938461	48274772	47.94	48.27	CN_756725	SNP_A-1857873	145	0.336311	-2.5	5	META37, META40, META53, META59, META62	1	4	DLX4, DLX3, ITGA3, PDK2, SAMD14, PPP1R9B, SGCA, HILS1, COL1A1		q21.33
17	48768474	49696874	48.77	49.7	SNP_A-1902997	SNP_A-8529705	389	0.9284	-3.92	3	META40, META53, META62	0	3	ABCC3, ANKRD40, WFIKKN2, TOB1, SPAG9, NME1, MBTD1, UTP18		q21.33

Regions differentially amplified in CN1 and CN2

chrom	start	end	start.MB	end.MB	start.probe	end.probe	BACs	length MB	maxM	max.overlap	amplified cases	amplifications in 1	amplifications in 2	genes	mirnas	cytobands

Regions differentially deleted in CN1 and CN2

Chrom	Start	End	Start MB	End MB	Start Probe	End Probe	BACs	Length MB	maxM	Max.Overlap	Cases	Deletions in CN1	Deletions inCN2	Genes	miRNAs	Cytobands
