# Supplementary material for: Genomic and transcriptomic heterogeneity in metaplastic carcinomas of the breast
Source: NPJ Breast Cancer. 2017 Dec 1;3:48. doi: 10.1038/s41523-017-0048-0 (PMC5711926; doi:10.1038/s41523-017-0048-0)
Supplement: Supplementary file 18 — Supplementary Table 6 [file 41523_2017_48_MOESM18_ESM.pdf]

Supplementary Table 6: Summary of chromosomal regions differentially gained, lost, amplified or deleted between chondroid and non-chondroid tumors. Protein coding genes and microRNAs mapping to these regions are listed. Chrom: chromosome; MB: megabase.

Regions differentially gained in chondroid and non-chondroid groups

Chromosome	Start	End	Start MB	End MB	Start Probe	End Probe	BACs	Length MB	maxM	Max.Overlap	Cases	Gains in Chondroid	Gains in Non-chondroid	Genes	miRNAs	Cytobands
21	14369115	15402520	14.37	15.4	CN_890660	SNP_A-8441067	2	1.033405	1.12	6	META30, META31, META52, META53, META55, META64	6	0	POTED		q11.2

Regions differentially lost in chondroid and non-chondroid groups

Chromosome	start	end	start.MB	end.MB	start.probe	end.probe	BACs	length MB	minM	max.overlap	loss cases	Gains in Chondroid	Gains in Non-chondroid	genes	mirnas	cytobands

Regions differentially amplified in chondroid and non-chondroid groups

Chromosome	Start	End	Start MB	End MB	Start Probe	End Probe	BACs	Length MB	maxM	Max.Overlap	Cases	Gains in Chondroid	Gains in Non-chondroid	Genes	miRNAs	Cytobands
8	78179269	82376711	78.18	82.38	SNP_A-1993930	SNP_A-8671860	1956	4.197442	3.97	7	META30, META31, META52, META53, META55, META59, META64	6	1	PKIA, FAM164A, IL7, STMN2, HEY1, MRPS28, TPD52, ZBTB10, ZNF704, PAG1, FABP5, PMP2, FABP9		q21.11-q21.13
8	82383836	102670463	82.38	102.67	SNP_A-8638900	CN_1252945	7882	20.286627	6	8	META30, META31, META41, META52, META53, META55, META59, META64	6	2	FABP4, FABP12, IMPA1, SLC10A5, ZFAND1, CHMP4C, SNX16, HNRNPA1P4, RALYL, LRRCC1, E2F5, CA13, CA1, CA3, CA2, REXO1L1, PSKH2, ATP6V0D2, SLC7A13, WWP1, FAM82B, CPNE3, CNGB3, CNBD1, WDR21C, MMP16, RIPK2, OSGIN2, NBN, DECR1, CALB1, TMEM64, TMEM55A, OTUD6B, LRRC69, SLC26A7, RUNX1T1, C8orf83, FAM92A1, RBM12B, C8orf39, TMEM67, PPM2C, CDH17, GEM, RAD54B, KIAA1429, DPY19L4, INTS8, CCNE2, C8orf38, TP53INP1, PLEKHF2, C8orf37, GDF6, UQCRB, MTERFD1, PTDSS1, SDC2, TSPYL5, MTDH, LAPTM4B, MATN2, RPL30, C8orf47, HRSP12, POP1, NIPAL2, KCNS2, STK3, VPS13B, COX6C, RGS22, FBXO43, POLR2K, SPAG1, RNF19A, ANKRD46, SNX31, PABPC1, RPS26P6, YWHAZ, ZNF706, NACAP1, GRHL2	hsa-mir-599, hsa-mir-875, hsa-mir-1273	q21.13-q22.3
8	102673474	110616365	102.67	110.62	CN_1252946	CN_1226116	4014	7.942891	5.4	8	META30, META31, META41, META52, META53, META55, META59, META64	6	2	GRHL2, NCALD, RRM2B, UBR5, ODF1, KLF10, AZIN1, ATP6V1C1, C8orf56, BAALC, FZD6, CTHRC1, SLC25A32, WDSOF1, RIMS2, TM7SF4, DPYS, LRP12, ZFPM2, OXR1, ABRA, ANGPT1, RSPO2, EIF3E, TTC35, TMEM74, TRHR, NUDCD1, ENY2, PKHD1L1, EBAG9	hsa-mir-548a-3	q22.3-q23.2
8	110617562	113753034	110.62	113.75	CN_1226117	CN_1244131	1551	3.135472	3.16	8	META30, META31, META41, META52, META53, META55, META59, META64	6	2	KCNV1, CSMD3	hsa-mir-2053	q23.2-q23.3
8	116597180	116597647	116.6	116.6	CN_1259926	SNP_A-2271181	3	0.000467	1.58	8	META30, META31, META39, META52, META53, META55, META59, META64	6	2	TRPS1		q23.3
8	119537210	119539332	119.54	119.54	SNP_A-8410904	AFFX-SNP_11157952	7	0.002122	1.36	8	META30, META31, META39, META52, META53, META55, META59, META64	6	2	SAMD12		q24.12
8	121493820	135516925	121.49	135.52	SNP_A-2236170	CN_1262658	8772	14.023105	7.04	8	META30, META31, META39, META52, META53, META55, META59, META64	6	2	MTBP, SNTB1, HAS2, ZHX2, DERL1, WDR67, FAM83A, C8orf76, ZHX1, ATAD2, WDYHV1, FBXO32, KLHL38, ANXA13, FAM91A1, FER1L6, C8orf54, C8orf78, TMEM65, TRMT12, RNF139, TATDN1, NDUFB9, MTSS1, ZNF572, SQLE, KIAA0196, NSMCE2, TRIB1, FAM84B, POU5F1B, MYC, TMEM75, CCDC26, GSDMC, FAM49B, ASAP1, ADCY8, EFR3A, OC90, KCNQ3, LRRC6, TMEM71, PHF20L1, TG, SLA, WISP1, NDRG1, ST3GAL1, ZFAT	hsa-mir-548d-1, hsa-mir-1204, hsa-mir-1205, hsa-mir-1207, hsa-mir-1208	q24.12-q24.22
8	135531226	139226364	135.53	139.23	CN_1262665	CN_1287450	2239	3.695138	6.21	8	META30, META31, META39, META52, META53, META55, META59, META64	6	2	ZFAT, KHDRBS3, FAM135B	hsa-mir-30b, hsa-mir-30d	q24.22-q24.23
8	139227850	140670865	139.23	140.67	SNP_A-8509370	SNP_A-2040859	1201	1.443015	3.11	8	META30, META31, META39, META52, META53, META55, META59, META64	6	2	FAM135B, COL22A1, KCNK9		q24.23-q24.3

8	140678407	146298168	140.68	146.3	SNP_A-2314685	CN_1287576	1486	5.619761	5.8	8	META30, META31, META39, META52, META53, META55, META59, META64	6	2	KCNK9, TRAPPC9, CHRAC1, EIF2C2, PTK2, DENND3, SLC45A4, GPR20, PTP4A3, TSNARE1, BAI1, ARC, JRK, PSCA, LY6K, C8orf55, SLURP1, LYPD2, LYNX1, LY6D, GML, CYP11B1, CYP11B2, LY6E, C8orf31, LY6H, GPIHBP1, ZFP41, ZNF696, TOP1MT, C8orf51, RHPN1, MAFA, ZC3H3, GSDMD, C8orf73, NAPRT1, EEF1D, TIGD5, PYCRL, TSTA3, ZNF623, MAPK15, FAM83H, SCRIB, NRBP2, PLEC1, PARP10, GRINA, SPATC1, OPLAH, EXOSC4, GPAA1, CYC1, SHARPIN, MAF1, KIAA1875, C8orf30A, HEATR7A, SCXB, C8orf30B, BOP1, SCXA, HSF1, DGAT1, SCRT1, FBXL6, GPR172A, ADCK5, CPSF1, SLC39A4, VPS28, NFKBIL2, CYHR1, KIFC2, FOXH1, PPP1R16A, GPT, MFSD3, RECQL4, LRRC14, LRRC24, C8orf82, ZNF251, ZNF34, RPL8, ZNF517, ZNF7, COMMD5, ZNF250, ZNF16, ZNF252, C8orf77, C8orf33	hsa-mir-151, hsa-mir-937, hsa-mir-661, hsa-mir-939, hsa-mir-1234	q24.3

Regions differentially deleted in chondroid and non-chondroid groups

Chromosome	start	end	start.MB	end.MB	start.probe	end.probe	BACs	length MB	minM	max.overlap	deleted cases	Gains in Chondroid	Gains in Non-chondroid	genes	mirnas	cytobands
