# Supplementary material for: Genomic and transcriptomic heterogeneity in metaplastic carcinomas of the breast
Source: NPJ Breast Cancer. 2017 Dec 1;3:48. doi: 10.1038/s41523-017-0048-0 (PMC5711926; doi:10.1038/s41523-017-0048-0)
Supplement: Supplementary file 19 — Supplementary Table 7 [file 41523_2017_48_MOESM19_ESM.pdf]

Supplementary Table 7: Summary of chromosomal regions differentially gained, lost, amplified or deleted between spindle and non-spindle tumors. Protein coding genes and microRNAs mapping to these regions are listed. Chrom: chromosome; MB: megabase.

Regions differentially gained in non-spindle and Spindle groups

Chromosome	Start	End	Start MB	End MB	Start Probe	End Probe	BACs	Length MB	maxM	Max.Overlap	Cases	Gains in Non-spindle	Gains in Spindle	Genes	miRNAs	Cytobands
7	70043404	70803055	70.04	70.8	CN_1247671	CN_1252165	472	0.759651	2.1	10	META30, META37, META40, META41, META42, META52, META53, META55, META62, META64	10	0	AUTS2, WBSCR17		q11.22
7	71283449	73993091	71.28	73.99	CN_1254418	CN_352557	695	2.709642	2.34	10	META30, META37, META40, META41, META42, META52, META53, META55, META62, META64	10	0	CALN1, TYW1B, POM121, NSUN5C, TRIM74, STAG3L3, NSUN5, TRIM50, FKBP6, FZD9, BAZ1B, BCL7B, TBL2, MLXIPL, VPS37D, DNAJC30, WBSCR22, STX1A, ABHD11, CLDN3, CLDN4, WBSCR27, WBSCR28, ELN, LIMK1, EIF4H, LAT2, RFC2, CLIP2, GTF2IRD1	hsa-mir-590	q11.22-q11.23

Regions differentially lost in non-spindle and Spindle groups

Chromosome	start	end	start.MB	end.MB	start.probe	end.probe	BACs	length MB	minM	max.overlap	loss cases	losses in non-spindle	losses in Spindle	genes	mirnas	cytobands

Regions differentially amplified in non-spindle and Spindle groups

Chromosome	start	end	start.MB	end.MB	start.probe	end.probe	BACs	length MB	maxM	max.overlap	amplified cases	amplifications in non-spindle	amplifications in Spindle	genes	mirnas	cytobands

Regions differentially deleted in non-spindle and Spindle groups

Chromosome	start	end	start.MB	end.MB	start.probe	end.probe	BACs	length MB	minM	max.overlap	deleted cases	deletions in non-spindle	deletions in Spindle	genes	mirnas	cytobands
