# Supplementary material for: Genomic and transcriptomic heterogeneity in metaplastic carcinomas of the breast
Source: NPJ Breast Cancer. 2017 Dec 1;3:48. doi: 10.1038/s41523-017-0048-0 (PMC5711926; doi:10.1038/s41523-017-0048-0)
Supplement: Supplementary file 20 — Supplementary Table 8 [file 41523_2017_48_MOESM20_ESM.pdf]

Supplementary Table 8: Summary of chromosomal regions differentially gained, lost, amplified or deleted between squamous and non-squamous tumors. Protein coding genes and microRNAs mapping to these regions are listed. Chrom: chromosome; MB: megabase.

Regions differentially gained in non-squamous and Squamous groups

Chrom	Start	End	Start MB	End MB	Start Probe	End Probe	BACs	Length MB	maxM	Max.Overlap	Cases	Gains in non-squamous	Gains in Squamous	Genes	miRNAs
6	94872298	96846681	94.87	96.85	SNP_A-8482196	CN_1200704	713	1.974383	6.3	6	37, META40, META41, ME	1	5	MANEA, FUT9	
7	49564516	49942330	49.56	49.94	CN_1227178	CN_1229426	269	0.377814	1.72	7	ETA40, META41, META4	2	5	VWC2	
7	50350429	51295775	50.35	51.3	SNP_A-1782005	CN_1238350	681	0.945346	2.56	7	ETA40, META41, META4	2	5	IKZF1, FIGNL1, DDC, GRB10, COBL	
7	52908346	53974862	52.91	53.97	SNP_A-8611835	SNP_A-8709933	540	1.066516	1.73	7	ETA40, META41, META4	2	5	POM121L12	
11	69814918	78904544	69.81	78.9	SNP_A-2248292	SNP_A-8653599	3588	9.089626	4.01	4	37, META40, META41, ME	0	4	ANO1, FADD, PPFIA1, CTTN, SHANK2, DHCR7, NADSYN1, KRTAP5-10, KRTAP5-11, OR7E87P, FAM86C, DEFB108B, RNF121, IL18BP, NUMA1, LRTOMT, C11orf59, C11orf51, FOLR3, FOLR1, FOLR2, INPPL1, PHOX2A, CLPB, PDE2A, ARAP1, STARD10, ATG16L2, FCHSD2, P2RY2, P2RY6, ARHGEF17, RELT, FAM168A, RAB6A, MRPL48, CHCHD8, PAAF1, DNAJB13, UCP2, UCP3, C2CD3, PPME1, P4HA3, PGM2L1, KCNE3, POLD3, CHRDL2, RNF169, XRRA1, SPCS2, NEU3, OR2AT4, SLCO2B1, ARRB1, RPS3, KLHL35, GDPD5, SERPINH1, MAP6, MOGAT2, DGAT2, UVRAG, WNT11, PRKRIR, C11orf30, LRRC32, GUCY2E, TSKU, ACER3, B3GNT6, CAPN5, MYO7A, GDPD4, PAK1, AQP11, CLNS1A, RSF1, C11orf67, INTS4, KCTD14, THRSP, NDUFC2, ALG8, KCTD21, USP35, GAB2, ZNF75C, NARS2, ODZ4	hsa-mir-548k, hsa-mir-139, hsa-mir-326
11	79282968	79967222	79.28	79.97	CN_570684	CN_572953	383	0.684254	2.52	4	37, META40, META41, ME	0	4		
11	121750112	122976011	121.75	122.98	CN_534588	CN_541090	786	1.225899	1.8	4	37, META40, META41, ME	0	4	BLID, UBASH3B, CRTAM, C11orf63, BSX, HSPA8	hsa-mir-125b-1, hsa-let-7a-2, hsa-mir-100
12	310523	3826633	0.31	3.83	CN_595964	CN_594135	1319	3.51611	3.58	4	37, META40, META41, ME	0	4	SLC6A12, SLC6A13, KDM5A, CCDC77, B4GALNT3, NINJ2, WNK1, RAD52, ERC1, FBXL14, WNT5B, ADIPOR2, LRTM2, DCP1B, CACNA1C, FKBP4, ITFG2, NRIP2, FOXM1, C12orf32, TULP3, TEAD4, TSPAN9, RPS27P23, PRMT8, EFCAB4B	
12	3853184	4385617	3.85	4.39	SNP_A-4271640	CN_626612	408	0.532433	2.17	4	37, META40, META41, ME	0	4	EFCAB4B, PARP11, CCND2	
12	4386274	9079615	4.39	9.08	CN_626619	CN_616628	2333	4.693341	4.65	4	37, META40, META41, ME	0	4	CCND2, C12orf5, FGF23, FGF6, RAD51AP1, DYRK4, AKAP3, NDUFA9, GALNT8, KCNA6, KCNA1, KCNA5, NTF3, ANO2, VWF, CD9, PLEKHG6, TNFRSF1A, SCNN1A, LTBR, CD27, TAPBPL, VAMP1, MRPL51, NCAPD2, GAPDH, IFFO1, NOP2, CHD4, LPAR5, ACRBP, ING4, ZNF384, C12orf53, COPS7A, MLF2, PTMS, LAG3, CD4, GPR162, GNB3, CDCA3, USP5, TPI1, LRRC23, ENO2, ATN1, C12orf57, PTPN6, PHB2, EMG1, LPCAT3, C1S, C1R, C1RL, RBP5, CLSTN3, PEX5, ACSM4, CD163L1, CD163, APOBEC1, GDF3, DPPA3, CLEC4C, NANOG, SLC2A14, NANOGP1, SLC2A3, FOXJ2, C3AR1, NECAP1, CLEC4A, ZNF705A, FAM90A1, CLEC6A, CLEC4D, CLEC4E, AICDA, MFAP5, A2ML1, PHC1	hsa-mir-200c, hsa-mir-141

Regions differentially lost in non-squamous and Squamous groups

Chrom	Start	End	Start MB	End MB	Start Probe	End Probe	BACs	Length MB	maxM	Max.Overlap	Cases	Losses in non-squamous	Losses in Squamous	Genes	miRNAs
2	228433677	228437302	228.43	228.44	CN_852256	CN_852257	3	0.003625	-2.96	7	ETA40, META41, META4	2	5		
4	108600544	108602299	108.6	108.6	CN_1038693	SNP_A-8413775	3	0.001755	-2.6	6	40, META41, META42, ME	1	5	PAPSS1	
7	102934903	104158272	102.93	104.16	CN_1196297	CN_1202949	875	1.223369	-3.15	4	37, META40, META41, ME	0	4	PMPCB, DNAJC2, PSMC2, SLC26A5, RELN, ORC5L, LHFPL3	
7	104181695	104214499	104.18	104.21	SNP_A-1892589	SNP_A-1852486	21	0.032804	-1.2	4	37, META40, META41, ME	0	4	LHFPL3	
7	120304355	120312772	120.3	120.31	CN_1196832	CN_1198910	3	0.008417	-3.88	6	39, META40, META41, ME	1	5	KCND2	
7	129518575	130151095	129.52	130.15	CN_1216776	SNP_A-2250896	284	0.63252	-2.41	4	37, META40, META41, ME	0	4	UBE2H, ZC3HC1, KLHDC10, TMEM209, C7orf45, CPA2, CPA4, CPA5, CPA1, TSGA14, MEST	hsa-mir-335
7	133508764	141026577	133.51	141.03	CN_1203721	SNP_A-4223663	3700	7.517813	-3.7	5	ETA40, META41, META4	0	5	EXOC4, LRGUK, SLC35B4, AKR1B1, AKR1B10, BPGM, CALD1, AGBL3, TMEM140, C7orf49, WDR91, STRA8, CNOT4, NUP205, SLC13A4, FAM180A, MTPN, CHRM2, PTN, DGKI, RPL41, CREB3L2, AKR1D1, TRIM24, SVOPL, ATP6V0A4, TMEM213, KIAA1549, ZC3HAV1L, ZC3HAV1, TTC26, UBN2, LUC7L2, C7orf55, KLRG2, CLEC2L, HIPK2, TBXAS1, PARP12, JHDM1D, SLC37A3, RAB19, MKRN1, DENND2A, ADCK2, NDUFB2, BRAF, MRPS33	hsa-mir-490
7	142124303	144550615	142.12	144.55	SNP_A-8283268	CN_1206087	495	2.426312	-2.13	4	37, META40, META42, ME	0	4	TRBV5-4, TRBV6-2, TRBC1, PRSS1, TRBC2, EPHB6, TRPV6, TRPV5, C7orf34, KEL, OR9A2, OR6V1, OR6W1P, PIP, TAS2R39, TAS2R40, GSTK1, TMEM139, CASP2, CLCN1, FAM131B, ZYX, EPHA1, TAS2R60, TAS2R41, FAM115C, FAM115A, OR2F2, OR2F1, OR6B1, OR2A5, OR2A25, OR2A12, OR2A2, OR2A14, ARHGEF5L, OR2A42, OR2A20P, OR2A9P, OR2A1, ARHGEF5, NOBOX, TPK1	
12	62782455	73567461	62.78	73.57	SNP_A-1911416	SNP_A-4242889	5382	10.785006	-3.61	7	ETA40, META41, META4	2	5	USP15, MON2, C12orf61, PPM1H, AVPR1A, DPY19L2, TMEM5, SRGAP1, C12orf66, C12orf56, XPOT, TBK1, RASSF3, GNS, TBC1D30, WIF1, LEMD3, MSRB3, HMGA2, LLPH, TMBIM4, IRAK3, HELB, GRIP1, CAND1, DYRK2, IFNG, IL26, IL22, MDM1, RAP1B, NUP107, SLC35E3, MDM2, CPM, CPSF6, LYZ, YEATS4, FRS2, CCT2, LRRC10, BEST3, RAB3IP, C12orf28, CNOT2, KCNMB4, PTPRB, PTPRR, TSPAN8, LGR5, ZFC3H1, THAP2, TMEM19, RAB21, TBC1D15, TPH2, TRHDE	hsa-let-7i, hsa-mir-548c
21	42123442	42124113	42.12	42.12	SNP_A-2019813	SNP_A-2019814	3	0.000671	-1.51	4	40, META41, META42, ME	0	4	DSCAM	

Regions differentially amplified in non-squamous and Squamous groups

Chrom	Start	End	Start MB	End MB	Start Probe	End Probe	BACs	Length MB	maxM	Max.Overlap	Cases	Amplification in non-squamous	Amplification in Squamous	Genes	miRNAs
8	116597180	116597647	116.6	116.6	CN_1259926	SNP_A-2271181	3	0.000467	1.58	8	39, META52, META53, ME	8	0	TRPS1	
8	119537210	119539332	119.54	119.54	SNP_A-8410904	FFX-SNP_111579	7	0.002122	1.36	8	39, META52, META53, ME	8	0	SAMD12	
8	121493820	135516925	121.49	135.52	SNP_A-2236170	CN_1262658	8772	14.023105	7.04	8	39, META52, META53, ME	8	0	MTBP, SNTB1, HAS2, ZHX2, DERL1, WDR67, FAM83A, C8orf76, ZHX1, ATAD2, WDYHV1, FBXO32, KLHL38, ANXA13, FAM91A1, FER1L6, C8orf54, C8orf78, TMEM65, TRMT12, RNF139, TATDN1, NDUFB9, MTSS1, ZNF572, SQLE, KIAA0196, NSMCE2, TRIB1, FAM84B, POU5F1B, MYC, TMEM75, CCDC26, GSDMC, FAM49B, ASAP1, ADCY8, EFR3A, OC90, KCNQ3, LRRC6, TMEM71, PHF20L1, TG, SLA, WISP1, NDRG1, ST3GAL1, ZFAT	hsa-mir-548d-1, hsa-mir-1204, hsa-mir-1205, hsa-mir-1207, hsa-mir-1208
8	135531226	139226364	135.53	139.23	CN_1262665	CN_1287450	2239	3.695138	6.21	8	39, META52, META53, ME	8	0	ZFAT, KHDRBS3, FAM135B	hsa-mir-30b, hsa-mir-30d
8	139227850	140670865	139.23	140.67	SNP_A-8509370	SNP_A-2040859	1201	1.443015	3.11	8	39, META52, META53, ME	8	0	FAM135B, COL22A1, KCNK9	
8	140678407	146298168	140.68	146.3	SNP_A-2314685	CN_1287576	1486	5.619761	5.8	8	39, META52, META53, ME	8	0	KCNK9, TRAPPC9, CHRAC1, EIF2C2, PTK2, DENND3, SLC45A4, GPR20, PTP4A3, TSNARE1, BAI1, ARC, JRK, PSCA, LY6K, C8orf55, SLURP1, LYPD2, LYNX1, LY6D, GML, CYP11B1, CYP11B2, LY6E, C8orf31, LY6H, GPIHBP1, ZFP41, ZNF696, TOP1MT, C8orf51, RHPN1, MAFA, ZC3H3, GSDMD, C8orf73, NAPRT1, EEF1D, TIGD5, PYCRL, TSTA3, ZNF623, MAPK15, FAM83H, SCRIB, NRBP2, PLEC1, PARP10, GRINA, SPATC1, OPLAH, EXOSC4, GPAA1, CYC1, SHARPIN, MAF1, KIAA1875, C8orf30A, HEATR7A, SCXB, C8orf30B, BOP1, SCXA, HSF1, DGAT1, SCRT1, FBXL6, GPR172A, ADCK5, CPSF1, SLC39A4, VPS28, NFKBIL2, CYHR1, KIFC2, FOXH1, PPP1R16A, GPT, MFSD3, RECQL4, LRRC14, LRRC24, C8orf82, ZNF251, ZNF34, RPL8, ZNF517, ZNF7, COMMD5, ZNF250, ZNF16, ZNF252, C8orf77, C8orf33	hsa-mir-151, hsa-mir-937, hsa-mir-661, hsa-mir-939, hsa-mir-1234
12	11891824	11999455	11.89	12	CN_562839	CN_599804	147	0.107631	1.86	3	ETA40, META41, META4	0	3	ETV6	

Regions differentially deleted in non-squamous and Squamous groups

chrom	start	end	start.MB	end.MB	start.probe	end.probe	BACs	length MB	minM	max.overlap	deleted cases	deletions in non-squamous	deletions in Squamous	genes	mirnas
