# Supplementary material for: Genomic and transcriptomic heterogeneity in metaplastic carcinomas of the breast
Source: NPJ Breast Cancer. 2017 Dec 1;3:48. doi: 10.1038/s41523-017-0048-0 (PMC5711926; doi:10.1038/s41523-017-0048-0)
Supplement: Supplementary file 21 — Supplementary Table 9 [file 41523_2017_48_MOESM21_ESM.pdf]

Supplementary Table 9: List of 190 differentially expressed transcripts in non-spindle and spindle tumors identified by SAM of the gene expression microarrays.

RefSeq Number	HUGO Gene Symbol	Score (d)	Numerator(r)	Denominator(s+s0)	Fold Change	q value	Validated as differentially expressed in RNA-seq (based on adjusted p-value)
NM_024626.2	VTCN1	-3.9284147	-6.427345795	1.636116927	86.0644657	0	Validated
NM_007281.1	SCRG1	-2.5933042	-6.346571185	2.447291466	81.37824064	0	Validated
NM_020659.2	TTYH1	-2.8369674	-5.921577052	2.08729121	60.61391202	0	Validated
NM_005980.2	S100P	-2.5362274	-5.883059271	2.319610361	59.01702413	0	
NM_005555.3	KRT6B	-2.146099	-5.634771132	2.6255877	49.6861252	0	
NM_006941.3	SOX10	-2.287843	-5.139614512	2.24648912	35.25154324	0	Validated
NM_017578.2	ROPN1	-2.6451818	-4.927897226	1.862971059	30.4400163	0	Validated
NM_020770.1	CGN	-2.9754429	-4.922587025	1.654404823	30.32818012	0	
NM_005558.3	LAD1	-3.4549918	-4.873912452	1.410687133	29.32201721	0	Validated
NM_006271.1	S100A1	-2.4466633	-4.741539475	1.937961593	26.7513441	0	Validated
NM_001851.3	COL9A1	-1.8076595	-4.681008231	2.589540848	25.6521569	0	
NM_021220.2	OVOL2	-2.8831114	-4.638776169	1.608947969	24.91212476	0	Validated
NM_004360.2	CDH1	-2.4922293	-4.635048753	1.859800295	24.84784368	0	Validated
NM_173853.2	KRTCAP3	-2.8686688	-4.562089915	1.590316033	23.62250271	0	
NM_002639.3	SERPINB5	-2.1555027	-4.467597571	2.072647606	22.12487764	0	
NM_001446.3	FABP7	-1.8509283	-4.353867431	2.352261584	20.44771078	0	
NM_001276.2	CHI3L1	-2.2798713	-4.350716272	1.908316599	20.40309729	0	Validated
NM_002281.2	KRT81	-1.8941836	-4.264843005	2.251546773	19.22408471	0	
NM_144707.1	PROM2	-2.3189998	-4.26106877	1.837459729	19.17385829	0	Validated
NM_203349.2	SHC4	-2.0292025	-4.212857453	2.076114906	18.54370291	0	
NM_006533.2	MIA	-2.6326464	-4.21117129	1.599596242	18.52204245	0	
NM_001012337.1	ROPN1B	-2.1982297	-4.202580644	1.911802334	18.41207915	0	Validated
NM_020387.2	RAB25	-2.6653826	-4.169609568	1.564356877	17.99606488	0	Validated
NM_006017.1	PROM1	-2.2793115	-4.142137557	1.817275746	17.65662327	0	Validated
NM_002353.1	TACSTD2	-1.9729371	-4.078223024	2.067082095	16.89147051	0	
NM_005979.2	S100A13	-2.1656597	-4.072860455	1.880655792	16.82880062	0	
NM_001002260.1	AIF1L	-2.0202997	-4.061823739	2.010505559	16.70055033	0	
NM_001185.2	AZGP1	-1.9609291	-4.052489686	2.06661709	16.59284868	0	Validated
NM_004000.2	CHI3L2	-1.9430321	-4.048588989	2.083644915	16.54804624	0	
XM_938439.1	ACAN	-1.8472411	-4.039470055	2.186758428	16.44377983	0	Validated
NM_206895.1	C2orf82	-1.8749068	-4.015647027	2.14178485	16.17447552	0	
NM_024889.3	C10orf81	-2.1703786	-3.9846333	1.835916193	15.83048225	0	
NM_002276.3	KRT15	-2.1373428	-3.939539572	1.843195043	15.34332839	0	
NM_002354.1	EPCAM	-2.2684855	-3.85025237	1.697278779	14.42253011	0	
NM_173495.2	PTCHD1	-2.4044958	-3.842173073	1.597912175	14.34198763	0	
NM_001044390.1	MUC1	-1.8895903	-3.79676281	2.009304795	13.8975899	0	
NM_003064.2	SLPI	-1.7090888	-3.759611446	2.199775375	13.5442767	0	
NM_001024912.1	CEACAM1	-1.967633	-3.740806998	1.901171105	13.36888275	0	
NM_001179.3	ART3	-1.9641022	-3.681322364	1.874302892	12.8288715	0	
NM_001076778.1	FAM107A	-1.6877762	-3.640556602	2.157013833	12.47144392	0	
NM_006147.2	IRF6	-2.0787599	-3.588266991	1.726157528	12.02751748	0	Validated
NM_183239.1	GSTO2	-2.232318	-3.580394418	1.603890885	11.96206384	0	Validated
NM_001044391.1	MUC1	-1.8713415	-3.567514795	1.906394349	11.8557481	0	
NM_021200.1	PLEKHB1	-2.0769485	-3.499818238	1.685077081	11.3122832	0	
NM_005434.3	MALL	-2.4775469	-3.476803148	1.403324848	11.13325192	0	Validated
NM_005556.3	KRT7	-1.950869	-3.448518566	1.767683339	10.91710605	0	
NM_032411.1	C2orf40	-1.3722534	-3.434527284	2.502837544	10.81174349	0.8494881	
NM_181505.1	PPP1R1B	-1.8120063	-3.419877858	1.887343284	10.7025143	0	
NM_002354.2	EPCAM	-2.0910313	-3.41103121	1.631267372	10.63708698	0	
NM_001306.2	CLDN3	-1.8424162	-3.410537167	1.851121955	10.63344499	0	Validated
NM_004455.2	EXTL1	-1.6023257	-3.39911316	2.121362148	10.54957636	0	
NM_020775.2	KIAA1324	-1.8346718	-3.391104966	1.848344193	10.49117939	0	Validated
NM_199328.1	CLDN8	-1.6367734	-3.386007829	2.068708934	10.45417876	0	
NM_003710.3	SPINT1	-2.2416136	-3.375795628	1.505966791	10.38043954	0	Validated
NM_024915.1	GRHL2	-2.5373773	-3.375228221	1.33020351	10.37635775	0	Validated
NM_002638.2	PI3	-1.4166775	-3.351545253	2.36577858	10.2074122	0.7936157	
NM_133467.2	CITED4	-2.2094862	-3.333609243	1.508771277	10.08129622	0	
NM_174911.3	FAM84B	-2.026057	-3.329001482	1.643093726	10.04914937	0	
NM_031426.2	AIF1L	-1.7831239	-3.304785548	1.853368445	9.881880078	0	
NM_000096.1	CP	-1.8874075	-3.244951412	1.719263837	9.480422877	0	Validated
NM_001025357.1	C8orf85	-1.8000639	-3.23609993	1.797769503	9.422434948	0	

NM_198993.2	STAC2	-1.8868402	-3.234330594	1.714151832	9.410886265	0	
NM_001885.1	CRYAB	-1.6054309	-3.217707538	2.004264099	9.303074226	0	
NM_022449.1	RAB17	-1.8218003	-3.192477959	1.752375408	9.141798111	0	Validated
NM_005288.1	GPR12	-1.6518984	-3.185399849	1.928326733	9.097056727	0	
NM_003102.2	SOD3	-1.4098967	-3.171352141	2.249350681	9.008907362	0.7936157	
NM_014220.2	TM4SF1	-2.2113941	-3.159191591	1.428597283	8.933289965	0	
NM_001031615.1	ALDH3B2	-1.6569788	-3.156998099	1.905273726	8.919717994	0	
NM_002773.3	PRSS8	-2.1823415	-3.152367247	1.444488511	8.891132836	0	Validated
NM_002456.4	MUC1	-1.7651083	-3.12471669	1.770269071	8.722348839	0	
NM_001854.3	COL11A1	-1.3721087	-3.117637557	2.272150633	8.679654144	0.8494881	
NM_003508.2	FZD9	-1.664316	-3.116128933	1.872318054	8.670582588	0	
NM_001035516.1	DMKN	-1.5991853	-3.09486884	1.9352784	8.54374649	0	
NM_031426.2	AIF1L	-1.6983682	-3.094043888	1.821774549	8.538862462	0	
NM_001002919.1	FAM150B	-1.3965604	-3.041466505	2.177826692	8.233275514	0.7936157	
NM_001005619.1	ITGB4	-1.4575629	-2.972132901	2.039111288	7.846954862	0.7936157	
NM_152332.3	TC2N	-1.7425679	-2.941700708	1.688141237	7.683164847	0	
NM_002474.2	MYH11	-1.4266898	-2.93934019	2.060251744	7.670604037	0.7936157	
NM_080590.1	CAPS	-1.6659917	-2.939167748	1.764215111	7.669687244	0	
NM_006210.1	ZIM2	-1.5589845	-2.924070456	1.875625138	7.589845171	0.4927031	
NM_014587.2	SOX8	-1.4360732	-2.921509231	2.034373543	7.576382829	0.7936157	Validated
NM_001039792.1	HRCT1	-1.5771571	-2.907132828	1.84327407	7.501259373	0.4927031	
NM_001263.2	CDS1	-1.8280267	-2.903820049	1.588499772	7.484054417	0	
NM_001017967.2	MARVELD3	-1.9531153	-2.903137306	1.486413702	7.480513495	0	Validated
NM_144724.1	MARVELD2	-1.9609006	-2.89723177	1.477500604	7.449955305	0	Validated
NM_001852.3	COL9A2	-1.6180505	-2.888719277	1.78530847	7.406126936	0	
NM_144626.1	TMEM125	-1.8978614	-2.887128884	1.521253824	7.397967099	0	Validated
NM_022965.1	FGFR3	-1.4925041	-2.869626366	1.922692442	7.308758508	0.4927031	
NM_005498.3	AP1M2	-1.8126667	-2.860164976	1.577876896	7.260983506	0	Validated
NM_012101.3	TRIM29	-1.6052633	-2.825490552	1.760141472	7.088550027	0	Validated
NM_201525.1	GPR56	-1.779178	-2.797310168	1.572248641	6.951431822	0	
NM_001085.4	SERPINA3	-1.3679003	-2.796489848	2.044366707	6.947480347	0.8494881	
NM_033254.2	BOC	-1.4207197	-2.783019201	1.95887986	6.88291265	0.7936157	
NM_019605.2	SERTAD4	-1.5891753	-2.770058916	1.743079495	6.821357694	0	
NM_153345.1	TMEM139	-1.5411475	-2.762188168	1.792293202	6.784244517	0.4927031	Validated
NM_002447.2	MST1R	-1.6511946	-2.75680056	1.669579449	6.758956646	0	Validated
NM_005309.1	GPT	-1.7341599	-2.753365534	1.587722986	6.742882858	0	
NM_001024912.1	CEACAM1	-1.5827707	-2.742887497	1.732965802	6.694087909	0	
NM_021572.4	ENPP5	-1.750949	-2.725248393	1.556440782	6.61274098	0	
NM_052943.2	FAM46B	-1.4948232	-2.675693477	1.78997319	6.389457649	0.4927031	
NM_032405.1	TMPRSS3	-1.4936834	-2.658035379	1.779517266	6.311729507	0.4927031	Validated
NM_003012.3	SFRP1	-1.4188149	-2.652765153	1.869704938	6.288714556	0.7936157	
NM_139072.3	DNER	-1.4141685	-2.640391298	1.867098031	6.23500751	0.7936157	
NM_003222.3	TFAP2C	-1.419308	-2.639050045	1.85939207	6.229213606	0.7936157	
NM_001038.4	SCNN1A	-1.2998442	-2.626305279	2.020476997	6.174427092	0.8494881	
NM_001982.2	ERBB3	-1.8235524	-2.597647673	1.424498505	6.052988762	0	Validated
NM_000494.3	COL17A1	-1.2909219	-2.582850208	2.000779656	5.991221649	0.8494881	
NM_015888.4	HOOK1	-1.5868822	-2.570105862	1.619594601	5.938530025	0	Validated
NM_001079843.1	CASZ1	-1.6055013	-2.564252427	1.597166223	5.914484486	0	
NM_170769.1	RNF39	-1.6410733	-2.555594233	1.557269967	5.87909558	0	
NM_014428.1	TJP3	-1.5049038	-2.530854675	1.681738534	5.779139421	0.4927031	
NM_001001552.3	LEMD1	-1.5521665	-2.527629049	1.628452247	5.76623266	0.4927031	Validated
NM_005797.2	MPZL2	-1.7644062	-2.52751158	1.432499799	5.765763175	0	Validated
NM_023938.5	C1orf116	-1.6914319	-2.524692211	1.492636039	5.754506506	0	Validated
NM_152321.1	ERP27	-1.3187964	-2.522848076	1.912992912	5.747155465	0.8494881	
NM_020340.3	KIAA1244	-1.6547613	-2.520686115	1.523292918	5.738549474	0	
NM_198148.1	CPXM2	-1.3853093	-2.509413694	1.8114465	5.693886339	0.7936157	
NM_001077186.1	MYH14	-1.6360563	-2.509044475	1.533592966	5.692429328	0	
NM_006829.2	C10orf116	-1.4713814	-2.480379579	1.685748942	5.580442711	0.4927031	
NM_001008844.1	DSP	-1.6955759	-2.479640256	1.462417746	5.57758369	0	
NM_198488.2	FAM83H	-1.577227	-2.468424002	1.56504039	5.534388812	0.4927031	
NM_052858.3	MARVELD3	-1.6049748	-2.457634007	1.53126019	5.493151214	0	Validated
NM_015286.5	SYNM	-1.3691226	-2.449314031	1.788966195	5.461563557	0.8494881	
NM_173570.2	ZDHHC23	-1.8431727	-2.426399969	1.316425732	5.375503787	0	Validated
NM_002006.3	FGF2	-1.3817673	-2.419738208	1.751190808	5.350739185	0.7936157	
NM_002354.1	EPCAM	-1.4349577	-2.409543003	1.679173569	5.313059993	0.7936157	
NM_198381.1	ELF5	-1.3935294	-2.386088254	1.712262594	5.227380779	0.7936157	Validated
NM_201524.1	GPR56	-1.5781224	-2.361922614	1.496666314	5.140549603	0.4927031	
NM_004433.3	ELF3	-1.6948326	-2.35487034	1.389441265	5.115482559	0	Validated
NM_014399.3	TSPAN13	-1.7308777	-2.347551197	1.356277915	5.089596189	0	

NM_004711.3	SYNGR1	-1.4533824	-2.346261785	1.614345788	5.085049383	0.7936157	
NM_005971.2	FXYD3	-1.4713247	-2.342687912	1.592230397	5.072468186	0.4927031	Validated
NM_004058.2	CAPS	-1.3927989	-2.31833517	1.664515388	4.987563365	0.7936157	
XR_017862.1	STRBP	-1.6557506	-2.302578942	1.390655655	4.933388634	0	
NM_020448.3	NIPAL3	-1.4382393	-2.298505481	1.598138435	4.919478819	0.7936157	
NM_005435.3	ARHGEF5	-1.5306999	-2.288990973	1.495388423	4.887141828	0.4927031	
NM_003980.3	MAP7	-1.5433592	-2.286687583	1.481630214	4.8793453	0.4927031	
NM_174881.2	CRB3	-1.4108848	-2.280630349	1.616453976	4.858902049	0.7936157	
NM_145296.1	CADM4	-1.4511951	-2.274006085	1.566988571	4.836643102	0.7936157	
NM_012101.3	TRIM29	-1.6433142	-2.271034831	1.381984587	4.82669221	0	Validated
NM_006558.1	KHDRBS3	-1.4682725	-2.266020151	1.543323986	4.809944179	0.4927031	
NM_020428.2	SLC44A2	-1.7838193	-2.248193109	1.260325566	4.750874539	0	
NM_177417.1	KLC3	-1.4743498	-2.24061411	1.51973029	4.725981921	0.4927031	
NM_206861.1	TACC2	-1.5677319	-2.235368991	1.425861732	4.70883115	0.4927031	Validated
NM_021907.3	DTNB	-1.554866	-2.232089988	1.435551308	4.698140921	0.4927031	
NM_003471.2	KCNAB1	-1.3182129	-2.227728642	1.689961206	4.683959631	0.8494881	
NM_015198.2	COBL	-1.5728705	-2.222721995	1.413162751	4.667732856	0.4927031	Validated
NM_001546.2	ID4	-1.3848591	-2.216775254	1.600722597	4.648532216	0.7936157	
NM_022121.2	PERP	-1.2967934	-2.214519756	1.70768894	4.641270415	0.8494881	
NM_019885.2	CYP26B1	-1.4473843	-2.21056101	1.527279955	4.628552255	0.7936157	
NM_018050.2	MANSC1	-1.4231802	-2.210371366	1.553121194	4.627943867	0.7936157	Validated
NM_024320.2	PRR15L	-1.3733903	-2.206769822	1.606804582	4.616405078	0.7936157	
BX537698	NFIB	-1.3882927	-2.188427883	1.576344788	4.558085173	0.7936157	
NM_014310.3	RASD2	-1.5221118	-2.186367485	1.436403985	4.551580147	0.4927031	
NM_001955.2	EDN1	-1.3802098	-2.17067424	1.572713292	4.502337603	0.7936157	
NM_015238.1	WWC1	-1.4440014	-2.164194404	1.498748166	4.482160791	0.7936157	
NM_144691.3	CAPN12	-1.3945	-2.154521618	1.545013731	4.452209923	0.7936157	
NM_021923.3	FGFRL1	-1.4561946	-2.1452717	1.473204016	4.423755652	0.7936157	
NM_013993.2	DDR1	-1.5351726	-2.143009422	1.395940397	4.416824235	0.4927031	
NM_001003397.1	TPD52L1	-1.5427078	-2.142756789	1.388958331	4.416050864	0.4927031	Validated
NM_006147.2	IRF6	-1.4420013	-2.140440581	1.484354095	4.408966701	0.7936157	Validated
XM_498725.3	AC020922.1	-1.5333446	-2.138349717	1.394565687	4.402581516	0.4927031	
NM_001307.3	CLDN7	-1.3611944	-2.136565207	1.569625313	4.397139209	0.8494881	
NM_006997.2	TACC2	-1.522123	-2.134622979	1.402398442	4.391223541	0.4927031	Validated
NM_001769.2	CD9	-1.4461975	-2.115226557	1.462612525	4.332580505	0.7936157	
NM_001012337.1	ROPN1B	-1.4610564	-2.099320054	1.436850801	4.285073807	0.7936157	Validated
NM_032034.2	SLC4A11	-1.4862266	-2.077485603	1.39782558	4.220709697	0.4927031	
NM_023938.4	C1orf116	-1.4467482	-2.075799686	1.43480369	4.215780303	0.7936157	Validated
NM_001003396.1	TPD52L1	-1.533239	-2.056656135	1.341380002	4.160209367	0.4927031	Validated
NM_005928.1	MFGE8	-1.3966829	-2.046260908	1.465086265	4.130341037	0.7936157	
NM_014214.1	IMPA2	-1.3430646	-2.043597972	1.521593239	4.122724258	0.8494881	
NM_177526.1	PPAP2C	-1.5386293	-2.027099177	1.317470794	4.075844968	0.4927031	Validated
NM_144670.2	A2ML1	-1.2890218	-2.020183374	1.567222069	4.056353469	0.8494881	Validated
NM_001005752.1	GJB3	-1.2913737	-2.011284368	1.557476585	4.031409589	0.8494881	
NM_020902.1	KIAA1543	-1.298766	-2.008931921	1.546800512	4.024841362	0.8494881	
NM_002600.3	PDE4B	-1.2947081	-2.008006734	1.550933948	4.022261096	0.8494881	
NM_015085.3	RAP1GAP2	-1.2865201	-2.001587189	1.555814942	4.004403044	0.8494881	
NM_020211.1	RGMA	-1.3316295	-1.972992196	1.481637523	3.92581502	0.8494881	
NM_005596.2	NFIB	-1.2854643	-1.967350792	1.530459325	3.910493789	0.8494881	
NM_178537.3	B4GALNT4	-1.3129498	-1.946083798	1.482222605	3.853271385	0.8494881	
NM_032323.1	TMEM79	-1.3436574	-1.945834934	1.448162992	3.852606755	0.8494881	
NM_006558.1	KHDRBS3	-1.292907	-1.878130665	1.452641707	3.675984453	0.8494881	
BI836710	AL139008.5	-1.3163818	-1.867455792	1.418627789	3.648885275	0.8494881	
NM_014399.3	TSPAN13	-1.5491467	-1.846214653	1.191762343	3.595555436	0.4927031	
NM_002224.2	ITPR3	-1.2887388	-1.838014514	1.426211825	3.575176616	0.8494881	
NM_033409.2	C20orf54	-1.3413846	-1.776118556	1.324093477	3.425034585	0.8494881	
BC038245	AL139008.5	-1.3183058	-1.76758025	1.340796865	3.404824057	0.8494881	
NM_003740.3	KCNK5	-1.3928935	-1.754654238	1.259718865	3.374454354	0.7936157	Validated
NM_130898.2	CREB3L4	-1.3534424	-1.727731327	1.27654584	3.312065781	0.8494881	
XM_371461.4	KIAA1671	-1.3408227	-1.666353433	1.242784344	3.174112875	0.8494881	
