# Supplementary material for: Genomic and transcriptomic heterogeneity in metaplastic carcinomas of the breast
Source: NPJ Breast Cancer. 2017 Dec 1;3:48. doi: 10.1038/s41523-017-0048-0 (PMC5711926; doi:10.1038/s41523-017-0048-0)
Supplement: Supplementary file 22 — Supplementary Table 10 [file 41523_2017_48_MOESM22_ESM.pdf]

Supplementary Table 10: Pathway analysis of genes differentially expressed between spindle and non-splindle tumors using g:Profiler.

Number of Query Genes	Number of Term Genes	Number of Common Genes	Term ID	Source	Description	Genes in Overlap	*p*-value
139	52	8	KEGG:04530	8	Tight junction	TJP3,MYH14,CRB3,MYH11,CGN,CLDN8,CLDN3,CLDN7	5.38E-04
286	68	11	REAC:1474244	38	Extracellular matrix organization	CDH1,COL9A2,COL11A1,COL17A1,CEACAM1,COL9A1,ITGB4,FGF2,ACAN,CAPN12,DDR1	2.17E-03
384	159	13	GO:0030198	9	extracellular matrix organization	CDH1,COL9A2,COL11A1,COL17A1,COL9A1,ITGB4,MYH11,FGF2,ACAN,ELF3,SPINT1,DDR1,SERPINB5	4.91E-02
257	159	12	GO:0034330	18	cell junction organization	CD9,CDH1,COL17A1,GRHL2,DSP,SFRP1,PERP,CRB3,ITGB4,MARVELD3,MARVELD2,CLDN3	4.06E-03
1137	159	25	GO:0030054	19	Cell junction	CD9,CDH1,COL17A1,FGFR3,GRHL2,DSP,SYNGR1,TJP3,CADM4,PERP,EPCAM,AIF1L,FGFRL1,CRB3,ITGB4,MARVELD3,CGN,MARVELD2,CLDN8,FABP7,CLDN3,CLDN7,SYNM,SHC4,GJB3	1.84E-02
1418	159	29	GO:0022610	34	Biological adhesion	CYP26B1,CD9,CDH1,ERBB3,COL17A1,CEACAM1,GRHL2,DSP,SFRP1,CADM4,PERP,ROPN1B,EPCAM,FGFRL1,ITGB4,VTCN1,MFGE8,BOC,MPZL2,CLDN8,ACAN,AZGP1,CLDN3,TTYH1,CLDN7,TACSTD2,MUC1,DDR1,GPR56	1.27E-02
224	159	10	GO:0045216	18	Cell-cell junction organization	CD9,CDH1,GRHL2,DSP,SFRP1,PERP,CRB3,MARVELD3,MARVELD2,CLDN3	4.53E-02
384	159	18	GO:0005911	19	Cell-cell junction	CDH1,COL17A1,GRHL2,DSP,TJP3,CADM4,PERP,EPCAM,FGFRL1,CRB3,MARVELD3,CGN,MARVELD2,CLDN8,FABP7,CLDN3,CLDN7,GJB3	8.35E-06
88	68	7	REAC:446728	7	Cell junction organization	CDH1,COL17A1,CRB3,ITGB4,CLDN8,CLDN3,CLDN7	1.09E-03
1412	159	29	GO:0007155	34	Cell adhesion	CYP26B1,CD9,CDH1,ERBB3,COL17A1,CEACAM1,GRHL2,DSP,SFRP1,CADM4,PERP,ROPN1B,EPCAM,FGFRL1,ITGB4,VTCN1,MFGE8,BOC,MPZL2,CLDN8,ACAN,AZGP1,CLDN3,TTYH1,CLDN7,TACSTD2,MUC1,DDR1,GPR56	1.17E-02
2724	159	51	GO:0070062	13	Extracellular exosome	PROM1,CD9,CDH1,CP,PRSS8,GSTO2,CEACAM1,FXYD3,DSP,SFRP1,MYH14,CADM4,SOD3,CRYAB,SCNN1A,KIAA1324,IRF6,EPCAM,PI3,SLPI,RAB17,AIF1L,SLC44A2,CRB3,ITGB4,RAB25,CHI3L1,MYH11,KRT7,MFGE8,PROM2,ART3,LAD1,AZGP1,DMKN,S100P,CLDN3,SPINT1,A2ML1,GPT,KRT15,RGMA,C1ORF116,TACSTD2,KRT6B,MUC1,S100A13,DDR1,GPR56,SERPINB5,CAPS	1.78E-05
576	159	17	GO:0030855	11	Epithelial cell differentiation	CYP26B1,SOX8,PROM1,GRHL2,DSP,SOX10,IRF6,OVOL2,RAB25,ELF5,FGF2,NFIB,MARVELD2,ELF3,TMEM79,CLDN3,MUC1	1.55E-02
61	68	5	REAC:421270	7	Cell-cell junction organization	CDH1,CRB3,CLDN8,CLDN3,CLDN7	2.56E-02
30	68	4	REAC:420029	7	Tight junction interactions	CRB3,CLDN8,CLDN3,CLDN7	2.02E-02
112	159	9	GO:0005923	19	Bicellular tight junction	TJP3,EPCAM,CRB3,MARVELD3,CGN,MARVELD2,CLDN8,CLDN3,CLDN7	9.79E-04
575	159	19	GO:0048729	11	Tissue morphogenesis	SOX8,PROM1,COL11A1,EDN1,GRHL2,DSP,SOX10,SFRP1,COBL,OVOL2,ITGB4,FGF2,TMEM79,SPINT1,ID4,RGMA,TACSTD2,DDR1,SERPINB5	7.80E-04
1490	159	30	GO:0042127	21	Regulation of cell proliferation	SOX8,CD9,ERBB3,FGFR3,EDN1,TFAP2C,SOX10,SFRP1,IRF6,EPCAM,OVOL2,FGFRL1,RAB25,VTCN1,FGF2,MFGE8,MARVELD3,NFIB,AZGP1,MST1R,FABP7,SPINT1,ID4,CLDN7,TACSTD2,SHC4,S100A13,DDR1,GPR56,SERPINB5	1.16E-02
