# Supplementary material for: Genomic and transcriptomic heterogeneity in metaplastic carcinomas of the breast
Source: NPJ Breast Cancer. 2017 Dec 1;3:48. doi: 10.1038/s41523-017-0048-0 (PMC5711926; doi:10.1038/s41523-017-0048-0)
Supplement: Supplementary file 23 — Supplementary Table 11 [file 41523_2017_48_MOESM23_ESM.pdf]

**Supplementary Table 11: List of 48 differentially expressed transcripts in non-chondroid vs chondroid tumors identified by SAM of the gene expression microarrays.**

| RefSeq Number | HUGO Gene Symbol | Score (d) | Numerator(r) | Denominator(s+s0) | Fold Change | q value | Validated as differentially expressed in RNA-seq (based on unadjusted p-value) |
|---|---|---|---|---|---|---|---|
| NM_001851.3 | COL9A1 | -3.471130799 | -6.542306955 | 1.884776845 | 93.20315942 | 0 | Validated |
| NM_020659.2 | TTYH1 | -2.819378295 | -5.695976507 | 2.020295225 | 51.83937816 | 0 | Validated |
| NM_007281.1 | SCRG1 | -2.159929238 | -5.478660481 | 2.536499986 | 44.59037666 | 0 | Validated |
| NM_006941.3 | SOX10 | -2.411176281 | -5.146251195 | 2.134332208 | 35.4140809 | 0 | Validated |
| XM_938439.1 | ACAN | -2.497061213 | -4.754816286 | 1.904164888 | 26.99866722 | 0 | Validated |
| NM_004455.2 | EXTL1 | -2.821343363 | -4.681127066 | 1.659183752 | 25.65426995 | 0 | Validated |
| NM_014587.2 | SOX8 | -3.101650335 | -4.479651721 | 1.444280057 | 22.31051206 | 0 | Validated |
| NM_005979.2 | S100A13 | -2.550644971 | -4.344550465 | 1.703314461 | 20.31608436 | 0 | Validated |
| NM_006271.1 | S100A1 | -2.182094414 | -4.323457883 | 1.981334013 | 20.02121861 | 0 | Validated |
| NM_206895.1 | C2orf82 | -2.065901968 | -4.192460107 | 2.029360624 | 18.28336999 | 0 | Validated |
| NM_032411.1 | C2orf40 | -1.714726712 | -4.009012957 | 2.337989447 | 16.10026977 | 0 | Validated |
| NM_203349.2 | SHC4 | -1.910735474 | -3.96106987 | 2.073060308 | 15.57402422 | 0 | Validated |
| NM_181505.1 | PPP1R1B | -2.184573382 | -3.780569274 | 1.730575546 | 13.74246856 | 0 | Validated |
| NM_080681.2 | COL11A2 | -1.900411491 | -3.77257999 | 1.985138486 | 13.66657654 | 0 | Validated |
| NM_001885.1 | CRYAB | -2.02328882 | -3.715003788 | 1.836121344 | 13.13190022 | 0 | Validated |
| NM_003102.2 | SOD3 | -1.720535337 | -3.63671383 | 2.113710629 | 12.4382691 | 0 | Validated |
| NM_001024912.1 | CEACAM1 | -1.928826935 | -3.628235172 | 1.881057915 | 12.3653843 | 0 | Validated |
| NM_017578.2 | ROPN1 | -1.671029666 | -3.604909269 | 2.157298188 | 12.16706482 | 0 | Validated |
| NM_139072.3 | DNER | -2.289942963 | -3.577390278 | 1.562218071 | 11.93718103 | 0 | Validated |
| NM_001076778.1 | FAM107A | -1.642828458 | -3.507299706 | 2.134915359 | 11.37109834 | 0 | Validated |
| NM_001012337.1 | ROPN1B | -1.717003918 | -3.500053587 | 2.038465696 | 11.31412874 | 0 | Validated |
| NM_003508.2 | FZD9 | -1.94057612 | -3.412518358 | 1.758507859 | 10.64805747 | 0 | Validated |
| NM_006533.2 | MIA | -1.826608272 | -3.354123644 | 1.836257777 | 10.22567125 | 0 | |
| NM_001008223.1 | C1QL4 | -1.556612782 | -3.31032566 | 2.126621147 | 9.919900569 | 0 | Validated |
| NM_001276.2 | CHI3L1 | -1.559159945 | -3.297888154 | 2.11516988 | 9.83474846 | 0 | |
| NM_033254.2 | BOC | -1.773593141 | -3.244201643 | 1.829169028 | 9.475497175 | 0 | Validated |
| NM_001852.3 | COL9A2 | -1.863623135 | -3.148507502 | 1.689455042 | 8.867377546 | 0 | Validated |
| NM_022965.1 | FGFR3 | -1.664615865 | -3.081552195 | 1.851209195 | 8.465247183 | 0 | Validated |
| NM_000087.2 | CNGA1 | -1.82748621 | -3.032987756 | 1.659650146 | 8.185030289 | 0 | Validated |
| NM_080590.1 | CAPS | -1.757220935 | -3.021154486 | 1.719279816 | 8.118169632 | 0 | Validated |
| NM_006210.1 | ZIM2 | -1.631154814 | -2.995387513 | 1.836360036 | 7.974463785 | 0 | |
| NM_000826.2 | GRIA2 | -1.699349443 | -2.991088363 | 1.760137314 | 7.950735703 | 0 | Validated |
| NM_005302.2 | GPR37 | -1.544906175 | -2.965628173 | 1.91961701 | 7.811654666 | 0 | Validated |
| NM_201539.1 | NDRG2 | -2.086449196 | -2.883359772 | 1.381945833 | 7.378664769 | 0 | Validated |
| NM_001851.3 | COL9A1 | -1.58276468 | -2.876616799 | 1.81746335 | 7.344258294 | 0 | Validated |
| NM_001024912.1 | CEACAM1 | -1.704462605 | -2.86944624 | 1.683490287 | 7.307846038 | 0 | Validated |
| NM_201539.1 | NDRG2 | -1.964505834 | -2.847120301 | 1.449280654 | 7.195626489 | 0 | Validated |
| NM_001463.2 | FRZB | -1.590848922 | -2.777990782 | 1.746231678 | 6.85896447 | 0 | Validated |
| NM_031461.3 | CRISPLD1 | -1.704161564 | -2.767508409 | 1.623970677 | 6.80930903 | 0 | Validated |
| NM_014729.2 | TOX | -1.642288417 | -2.751088921 | 1.675155772 | 6.732250789 | 0 | Validated |
| NM_152765.2 | C8orf46 | -1.653875444 | -2.703628339 | 1.634723068 | 6.51438208 | 0 | Validated |
| NM_013227.2 | ACAN | -1.567624482 | -2.681111855 | 1.710302363 | 6.413499869 | 0 | Validated |
| NM_001217.3 | CA11 | -1.688859685 | -2.643105854 | 1.565023949 | 6.246750262 | 0 | Validated |
| NM_005309.1 | GPT | -1.590835772 | -2.585918596 | 1.62550946 | 6.003977607 | 0 | |
| NM_020448.3 | NIPAL3 | -1.652338192 | -2.529875199 | 1.531088013 | 5.775217175 | 0 | Validated |
| NM_001005367.1 | TTYH1 | -1.727180255 | -2.418218439 | 1.400096158 | 5.345105558 | 0 | Validated |
| NM_005079.2 | TPD52 | -1.663021837 | -2.418130582 | 1.454058226 | 5.344780063 | 0 | Validated |
| NM_017508.1 | SOX6 | -1.629666168 | -2.175837637 | 1.33514316 | 4.518480314 | 0 | Validated |
