# Supplementary material for: Genomic and transcriptomic heterogeneity in metaplastic carcinomas of the breast
Source: NPJ Breast Cancer. 2017 Dec 1;3:48. doi: 10.1038/s41523-017-0048-0 (PMC5711926; doi:10.1038/s41523-017-0048-0)
Supplement: Supplementary file 24 — Supplementary Table 12 [file 41523_2017_48_MOESM24_ESM.pdf]

Supplementary Table 12: List of 19 differentially expressed transcripts in non-squamous vs squamous tumors identified by SAM of the gene expression microarrays.

RefSeq Number	HUGO Gene Symbol	Score (d)	Numerator(r)	Denominator(s+s0)	Fold Change	q value	Validated as differentially expressed in RNA-seq (based on unadjusted p-value)
NM_005555.3	KRT6B	1.545213965	4.684902207	3.031879283	25.72148812	0	Validated
NM_002423.3	MMP7	1.775718332	4.527280446	2.549548745	23.05935806	0	Validated
NM_005980.2	S100P	1.504359547	4.336924349	2.88290413	20.20897646	0	Validated
NM_002638.2	PI3	1.688970826	4.127116355	2.443568764	17.47373795	0	Validated
NM_017947.1	MOCOS	1.868732233	3.761771372	2.013007164	13.56456965	0	Validated
NM_001038.4	SCNN1A	1.76029966	3.585704588	2.036985332	12.00617409	0	Validated
NM_002423.3	MMP7	1.645133861	3.569257234	2.169584688	11.87007573	0	Validated
NM_145016.2	GLYATL2	1.572465477	3.559514263	2.263651771	11.79018346	0	Validated
NM_016724.1	FOLR1	1.659158296	3.555354385	2.142866291	11.75623652	0	Validated
NM_006103.3	WFDC2	1.94391408	3.288976149	1.691934938	9.774183221	0	Validated
NM_012153.3	EHF	1.578659371	3.230306414	2.046233958	9.38467259	0	Validated
NM_002965.2	S100A9	1.54996798	3.084097773	1.989781603	8.480196958	0	Validated
NM_020980.2	AQP9	1.667967577	2.952306889	1.770002564	7.739856877	0	Validated
NM_001005731.1	ITGB4	1.403168499	2.745568395	1.95669187	6.70653881	0	Validated
NM_198213.1	OASL	1.393368195	2.642036393	1.896150926	6.242121301	0	Validated
NM_002754.3	MAPK13	1.601899787	2.548218509	1.590747767	5.849115628	0	Validated
NM_144649.1	TMEM71	1.575132215	2.543539052	1.614809873	5.830174448	0	Validated
NM_003149.1	STAC	1.455775009	2.363495835	1.623530985	5.146158296	0	Validated
NM_006408.3	AGR2	1.446244993	2.323411528	1.606513101	5.005143832	0	Validated
