# Supplementary material for: Genomic and transcriptomic heterogeneity in metaplastic carcinomas of the breast
Source: NPJ Breast Cancer. 2017 Dec 1;3:48. doi: 10.1038/s41523-017-0048-0 (PMC5711926; doi:10.1038/s41523-017-0048-0)
Supplement: Supplementary file 25 — Supplementary Table 13 [file 41523_2017_48_MOESM25_ESM.pdf]

Supplementary Table 13: Summary of differentially expressed genes between spindle and non-spindle MBCs according to RNA-sequencing. Statistics calculated using the limma package. Genes with p-value <0.05 have been included.

gene_id	gene_name	Fold Change (log2)	Average expression	t-statistic	p value	adjusted p value
ENSG00000104413	ESRP1	-6.097848174	5.533385619	-13.6381239	1.24E-12	1.95E-08
ENSG00000083307	GRHL2	-5.906060953	4.203686018	-12.09584941	1.47E-11	1.16E-07
ENSG00000065361	ERBB3	-5.312797391	5.43637866	-11.38712281	4.98E-11	2.60E-07
ENSG00000159166	LAD1	-6.346727205	4.978823819	-11.20809832	6.82E-11	2.68E-07
ENSG00000167880	EVPL	-5.345281413	4.108936394	-10.02094054	6.08E-10	1.91E-06
ENSG00000089356	FXYD3	-5.773936923	5.751722871	-9.458087835	1.82E-09	4.13E-06
ENSG00000163435	ELF3	-5.363436468	4.495554919	-9.451456947	1.84E-09	4.13E-06
ENSG00000171219	CDC42BPG	-4.684964255	5.583020782	-9.231810786	2.86E-09	5.61E-06
ENSG00000105329	TGFB1	3.325132216	7.016443466	8.98616611	4.70E-09	8.20E-06
ENSG00000117983	MUC5B	-7.17204163	5.264615045	-8.504204431	1.28E-08	2.01E-05
ENSG00000173898	SPTBN2	-3.282263624	5.878875397	-8.223881029	2.32E-08	3.31E-05
ENSG00000047457	CP	-5.651337912	6.182966063	-7.907347986	4.60E-08	5.55E-05
ENSG00000076826	CAMSAP3	-4.634782672	3.969498936	-7.928411807	4.40E-08	5.55E-05
ENSG00000177106	EPS8L2	-3.685649569	7.751319187	-7.790658525	5.94E-08	6.03E-05
ENSG00000171345	KRT19	-5.930377291	4.892507454	-7.746638966	6.55E-08	6.05E-05
ENSG00000164078	MST1R	-4.465303389	3.961209031	-7.775585811	6.15E-08	6.03E-05
ENSG00000118194	TNNT2	-4.377259612	3.124219496	-7.8446998	5.28E-08	5.92E-05
ENSG00000105699	LSR	-5.13596712	6.698847454	-7.624891212	8.58E-08	7.48E-05
ENSG00000119888	EPCAM	-5.195895664	5.605892708	-7.40633122	1.40E-07	0.00010454
ENSG00000142449	FBN3	-5.87781686	4.99407629	-7.419488274	1.36E-07	0.00010454
ENSG00000142273	CBLC	-4.922102735	3.494713408	-7.436894614	1.31E-07	0.00010454
ENSG00000132698	RAB25	-5.005416023	3.902436575	-7.323190929	1.69E-07	0.000120395
ENSG00000167614	TTYH1	-5.688802616	4.988806292	-7.187308256	2.30E-07	0.000156818
ENSG00000166535	A2ML1	-4.849780112	3.658023663	-7.102824337	2.79E-07	0.000182309
ENSG00000111907	TPD52L1	-4.011599803	3.842748566	-7.04178739	3.21E-07	0.000201346
ENSG00000141738	GRB7	-4.059441698	4.039026017	-6.923079903	4.22E-07	0.000254606
ENSG00000117595	IRF6	-5.139650128	5.708287469	-6.832891954	5.20E-07	0.000302257
ENSG00000096696	DSP	-3.916769328	6.802622821	-6.791988411	5.72E-07	0.000320592
ENSG00000106333	PCOLCE	3.052987528	7.681261794	6.722860121	6.72E-07	0.00036378
ENSG00000096433	ITPR3	-2.821997349	7.381412952	-6.577553695	9.46E-07	0.00046379
ENSG00000189334	S100A14	-4.896240561	3.37589422	-6.603180457	8.90E-07	0.00046379
ENSG00000134317	GRHL1	-3.897209425	4.66517238	-6.539747198	1.03E-06	0.000491709
ENSG00000130701	RBBP8NL	-3.39808627	2.436541699	-6.588362129	9.22E-07	0.00046379
ENSG00000157992	KRTCAP3	-3.98888947	3.849213235	-6.483971846	1.18E-06	0.000544561
ENSG00000169908	TM4SF1	-3.776147428	6.64978359	-6.401313288	1.44E-06	0.000643688
ENSG00000135525	MAP7	-2.914815317	5.189510156	-6.356290186	1.60E-06	0.000696633
ENSG00000157613	CREB3L1	4.225425845	5.54220793	6.208419431	2.28E-06	0.000940067
ENSG00000162069	CCDC64B	-4.540658504	3.663348998	-6.234931651	2.14E-06	0.000905975
ENSG00000129353	SLC44A2	-2.290144116	8.981477769	-6.158311829	2.57E-06	0.001033191
ENSG00000106078	COBL	-3.93829313	3.478448723	-6.147477198	2.64E-06	0.001033975
ENSG00000143375	CGN	-4.421278775	4.459119592	-6.09985453	2.96E-06	0.001104498
ENSG00000103067	ESRP2	-3.68300287	3.587736969	-6.123189763	2.79E-06	0.001069531
ENSG00000073350	LLGL2	-3.132072998	6.368754852	-6.057625598	3.27E-06	0.001194628
ENSG00000129354	AP1M2	-5.456436809	4.476203314	-6.005323511	3.72E-06	0.001295549
ENSG00000183111	ARHGEF37	-3.662010274	3.430320024	-6.01612361	3.62E-06	0.001290784
ENSG00000141934	PPAP2C	-3.846498005	5.507642152	-5.96289432	4.12E-06	0.00140283
ENSG00000039068	CDH1	-4.748859849	5.936661309	-5.9545887	4.20E-06	0.00140283
ENSG00000138080	EMILIN1	3.062871423	7.126575956	5.944805568	4.30E-06	0.001406627
ENSG00000164106	SCRG1	-5.846472687	5.113284818	-5.920258677	4.57E-06	0.001433363
ENSG00000132849	INADL	-2.113976681	6.480095313	-5.922098608	4.55E-06	0.001433363
ENSG00000172197	MBOAT1	-3.046009443	5.42298694	-5.902116752	4.77E-06	0.001447339
ENSG00000163701	IL17RE	-4.268877595	4.804690306	-5.900144292	4.80E-06	0.001447339
ENSG00000119699	TGFB3	2.704158922	4.568860424	5.79404781	6.21E-06	0.001801273
ENSG00000119042	SATB2	2.871115556	3.49381298	5.806861444	6.02E-06	0.001782643
ENSG00000185101	ANO9	-3.37649618	4.239548062	-5.780076499	6.43E-06	0.001801273
ENSG00000137699	TRIM29	-4.029628181	6.174167317	-5.780437322	6.42E-06	0.001801273
ENSG00000135480	KRT7	-5.428933052	6.763855502	-5.757548474	6.79E-06	0.001869929
ENSG00000133048	CHI3L1	-6.404444991	7.332063101	-5.749727117	6.92E-06	0.001873207
ENSG00000100146	SOX10	-5.756634996	3.702498185	-5.722719765	7.40E-06	0.001967381
ENSG00000141750	STAC2	-5.336089527	5.258392535	-5.690753422	8.00E-06	0.002066995

ENSG00000105472	CLEC11A	3.874819359	5.230747121	5.688144287	8.05E-06	0.002066995
ENSG00000148735	PLEKHS1	-4.771069945	3.830358196	-5.677184934	8.27E-06	0.002066995
ENSG00000088386	SLC15A1	-4.303324309	2.971470494	-5.675834724	8.30E-06	0.002066995
ENSG00000164626	KCNK5	-3.089538219	3.850629971	-5.641572687	9.03E-06	0.002179292
ENSG00000105357	MYH14	-4.586637194	7.124239129	-5.652283909	8.79E-06	0.002155847
ENSG00000111261	MANSC1	-3.216091119	4.130637269	-5.605317411	9.87E-06	0.002335228
ENSG00000149573	MPZL2	-3.322948356	4.946369454	-5.601153137	9.97E-06	0.002335228
ENSG00000143217	PVRL4	-3.930044342	3.649904717	-5.532600644	1.18E-05	0.00272434
ENSG00000197857	ZNF44	-2.651777463	4.08536265	-5.505351682	1.26E-05	0.002786476
ENSG00000240038	AMY2B	-3.594582623	4.432629988	-5.491784454	1.31E-05	0.002786476
ENSG00000181143	MUC16	-7.057189005	4.628930034	-5.489217999	1.31E-05	0.002786476
ENSG00000147676	MAL2	-4.788476898	3.397660436	-5.499680929	1.28E-05	0.002786476
ENSG00000113140	SPARC	2.538603424	11.58055022	5.525046892	1.20E-05	0.002735368
ENSG00000188112	C6orf132	-3.197759129	3.995534681	-5.459675824	1.41E-05	0.002945942
ENSG00000143196	DPT	3.247326269	3.831986813	5.455917855	1.43E-05	0.002945942
ENSG00000167306	MYO5B	-4.152970977	4.616220857	-5.441234915	1.48E-05	0.003015246
ENSG00000108821	COL1A1	3.458465496	13.95750562	5.495754143	1.29E-05	0.002786476
ENSG00000007062	PROM1	-4.943593659	5.636671117	-5.430052088	1.52E-05	0.003060122
ENSG00000121068	TBX2	3.010132223	5.027847732	5.402328847	1.63E-05	0.003198721
ENSG00000106236	NPTX2	3.509538074	1.149095126	5.401941682	1.63E-05	0.003198721
ENSG00000165125	TRPV6	-3.637707466	3.160639834	-5.372947353	1.75E-05	0.003394629
ENSG00000073605	GSDMB	-1.989154789	5.197486322	-5.34073873	1.90E-05	0.003545689
ENSG00000155066	PROM2	-3.754884156	5.47104338	-5.330581744	1.95E-05	0.003593446
ENSG00000204580	DDR1	-2.56276264	8.139461942	-5.358363682	1.82E-05	0.00343559
ENSG00000142949	PTPRF	-2.532217785	8.952228492	-5.358277169	1.82E-05	0.00343559
ENSG00000135127	CCDC64	-3.043030639	2.836990462	-5.310215822	2.05E-05	0.003735921
ENSG00000146904	EPHA1	-3.325669295	4.180191762	-5.287475885	2.17E-05	0.003819861
ENSG00000134258	VTCN1	-3.9766801	3.148565923	-5.290972724	2.15E-05	0.003819861
ENSG00000117385	LEPRE1	2.260374287	7.795555592	5.303770389	2.08E-05	0.003752592
ENSG00000122121	XPNPEP2	3.028663291	1.528863379	5.248720816	2.39E-05	0.00412202
ENSG00000183160	TMEM119	3.281633558	4.52609468	5.207944415	2.64E-05	0.004374663
ENSG00000167578	RAB4B	-3.479779591	3.423872372	-5.206761742	2.65E-05	0.004374663
ENSG00000102265	TIMP1	1.917628928	8.700139057	5.24793135	2.39E-05	0.00412202
ENSG00000185008	ROBO2	3.984296378	2.128446629	5.221330812	2.55E-05	0.004356337
ENSG00000059588	TARBP1	-1.884191822	6.664019752	-5.207913578	2.64E-05	0.004374663
ENSG00000085552	IGSF9	-2.670151466	5.331437826	-5.134739985	3.17E-05	0.00512725
ENSG00000196187	TMEM63A	-2.004174427	7.726661567	-5.142045583	3.11E-05	0.005087074
ENSG00000185634	SHC4	-4.473473047	4.199860577	-5.076455255	3.67E-05	0.00580837
ENSG00000106541	AGR2	-3.796597966	2.656697222	-5.081296198	3.62E-05	0.005799446
ENSG00000167680	SEMA6B	2.17623609	4.781909274	5.06861405	3.74E-05	0.00580837
ENSG00000038295	TLL1	2.927130042	1.689427265	5.053317216	3.88E-05	0.005811194
ENSG00000158769	F11R	-2.201150808	4.221067323	-5.041976174	4.00E-05	0.005860196
ENSG00000167642	SPINT2	-3.369689173	6.187618756	-5.072522052	3.70E-05	0.00580837
ENSG00000120332	TNN	3.105711506	1.710754916	5.049089767	3.93E-05	0.005811194
ENSG00000138162	TACC2	-1.948171452	5.924448211	-5.064660891	3.78E-05	0.005808531
ENSG00000020181	GPR124	2.648856717	6.023078049	5.056856465	3.85E-05	0.005811194
ENSG00000156219	ART3	-4.221190171	3.231887102	-5.001363021	4.42E-05	0.006367848
ENSG00000181392	SYNE4	-2.833249874	3.14099195	-4.994500549	4.50E-05	0.0063819
ENSG00000079385	CEACAM1	-3.184408505	3.937776995	-4.983371305	4.63E-05	0.006425377
ENSG00000164692	COL1A2	2.768176758	13.77255545	5.051103086	3.91E-05	0.005811194
ENSG00000107672	NSMCE4A	-1.447269871	5.219463719	-4.988670429	4.57E-05	0.006397318
ENSG00000111728	ST8SIA1	-2.932889164	2.884678353	-4.966526362	4.83E-05	0.006552941
ENSG00000163354	DCST2	-2.492561072	3.055809586	-4.960615806	4.90E-05	0.006552941
ENSG00000115232	ITGA4	2.930086238	4.806394696	4.95973874	4.91E-05	0.006552941
ENSG00000152926	ZNF117	-2.366556778	5.07784742	-4.958229644	4.93E-05	0.006552941
ENSG00000092758	COL9A3	-5.119885189	7.762538312	-4.993217508	4.51E-05	0.0063819
ENSG00000130635	COL5A1	2.993919855	10.09709301	5.002744112	4.41E-05	0.006367848
ENSG00000215018	COL28A1	-3.108464996	2.954822108	-4.936996501	5.20E-05	0.006852857
ENSG00000186832	KRT16	-5.067673771	6.465541382	-4.958910835	4.92E-05	0.006552941
ENSG00000163624	CDS1	-3.14827707	3.026027617	-4.921961674	5.40E-05	0.007048377
ENSG00000164683	HEY1	3.351829204	3.579274374	4.909934382	5.56E-05	0.007048377
ENSG00000166415	WDR72	2.183861645	0.780069656	4.907842237	5.59E-05	0.007048377
ENSG00000095637	SORBS1	-3.228328711	4.872503751	-4.914715381	5.50E-05	0.007048377
ENSG00000204396	VWA7	-2.77248219	2.801521247	-4.906144025	5.61E-05	0.007048377
ENSG00000065371	ROPN1	-4.224825693	2.922829484	-4.90072754	5.69E-05	0.007070101

ENSG00000150551	LYPD1	1.822619406	0.658282048	4.890601334	5.84E-05	0.007136891
ENSG00000166689	PLEKHA7	-2.887377409	4.756791835	-4.89858479	5.72E-05	0.007070101
ENSG00000175265	GOLGA8A	-3.378120832	5.210137071	-4.907091464	5.60E-05	0.007048377
ENSG00000101276	SLC52A3	-2.690239383	2.567777455	-4.888602933	5.87E-05	0.007136891
ENSG00000065621	GSTO2	-2.969206066	2.770678948	-4.877465244	6.03E-05	0.007226991
ENSG00000138771	SHROOM3	-2.870807629	3.963175249	-4.861973968	6.27E-05	0.007456409
ENSG00000149380	P4HA3	2.949075259	3.592760181	4.857833483	6.34E-05	0.007474
ENSG00000143882	ATP6V1C2	-4.268032255	3.455570568	-4.843068567	6.58E-05	0.007574432
ENSG00000162745	OLFML2B	2.967769779	6.663747396	4.885242776	5.92E-05	0.007141916
ENSG00000140511	HAPLN3	-3.098342419	5.155899725	-4.840895752	6.61E-05	0.007574432
ENSG00000162551	ALPL	3.768276643	5.743700532	4.852090686	6.43E-05	0.007474
ENSG00000016391	CHDH	-2.767340712	3.681729983	-4.814737506	7.06E-05	0.008029769
ENSG00000157766	ACAN	-5.167707382	6.965989872	-4.852076747	6.43E-05	0.007474
ENSG00000174292	TNK1	-2.428026821	3.557212853	-4.796655057	7.39E-05	0.008342232
ENSG00000137747	TMPRSS13	-3.88500875	3.292142311	-4.7488984	8.33E-05	0.009142179
ENSG00000043039	BARX2	-3.523899928	2.537683236	-4.755278311	8.20E-05	0.009129172
ENSG00000205426	KRT81	-4.738960978	5.125938797	-4.752258726	8.26E-05	0.009129172
ENSG00000133477	FAM83F	-2.93092129	3.569079603	-4.715546768	9.06E-05	0.009804167
ENSG00000102890	ELMO3	-3.106334364	4.498802985	-4.716048118	9.05E-05	0.009804167
ENSG00000167552	TUBA1A	1.512076116	7.068098698	4.752349132	8.26E-05	0.009129172
ENSG00000176788	BASP1	3.014729462	2.966529317	4.684204396	9.80E-05	0.010460581
ENSG00000141527	CARD14	-2.600501345	4.001278326	-4.681613981	9.87E-05	0.010460581
ENSG00000163898	LIPH	-3.059040211	3.442001557	-4.661480698	0.000103788	0.010856937
ENSG00000168453	HR	-2.944821419	5.406183224	-4.684581135	9.79E-05	0.010460581
ENSG00000142675	CNKSR1	-3.422592322	4.088626566	-4.646644264	0.000107733	0.011048603
ENSG00000157350	ST3GAL2	1.666270791	4.665823959	4.650787328	0.000106616	0.011006041
ENSG00000160862	AZGP1	-5.186967782	5.228347393	-4.656990815	0.000104966	0.010907471
ENSG00000054179	ENTPD2	-2.797597826	3.120059954	-4.62194321	0.000114637	0.011396993
ENSG00000198795	ZNF521	-2.46449817	4.611627991	-4.624953736	0.000113772	0.011396993
ENSG00000166145	SPINT1	-2.883739345	6.771954698	-4.666908249	0.000102382	0.010781677
ENSG00000206075	SERPINB5	-4.369773109	4.918973547	-4.626161341	0.000113427	0.011396993
ENSG00000182795	C1orf116	-3.273593102	2.786461085	-4.600703873	0.000120926	0.011568021
ENSG00000132746	ALDH3B2	-3.345176717	2.736604429	-4.598349797	0.000121644	0.011568021
ENSG00000214944	ARHGEF28	-3.409784938	4.948859218	-4.610829078	0.000117886	0.011510412
ENSG00000185946	RNPC3	-2.003740402	5.200905698	-4.614276068	0.000116868	0.011510412
ENSG00000172201	ID4	-2.862839253	3.659295182	-4.581567081	0.000126889	0.011867843
ENSG00000094755	GABRP	-5.090873457	3.52895116	-4.579137129	0.000127667	0.011867843
ENSG00000150712	MTMR12	-2.162438705	5.793003353	-4.621509672	0.000114762	0.011396993
ENSG00000006016	CRLF1	-4.124853861	4.321843287	-4.575928978	0.000128701	0.011879145
ENSG00000143013	LMO4	-1.505196453	7.075452518	-4.626695897	0.000113275	0.011396993
ENSG00000118898	PPL	-3.625776458	4.927857098	-4.583908235	0.000126144	0.011867843
ENSG00000008441	NFIX	-2.137176239	7.578809855	-4.607773732	0.000118795	0.011510412
ENSG00000162576	MXRA8	2.829153514	7.941864014	4.607630672	0.000118838	0.011510412
ENSG00000123416	TUBA1B	1.555466092	7.987280135	4.602904329	0.000120259	0.011568021
ENSG00000152092	ASTN1	3.175061528	1.499146315	4.523107669	0.000146991	0.013331972
ENSG00000141696	LEPREL4	1.577246746	6.864869595	4.578652948	0.000127823	0.011867843
ENSG00000130545	CRB3	-2.8475272	2.095831439	-4.504373895	0.000154084	0.013678567
ENSG00000052344	PRSS8	-3.319016476	3.944560068	-4.503819776	0.000154299	0.013678567
ENSG00000160211	G6PD	1.534606836	6.197230992	4.539296938	0.000141125	0.012949619
ENSG00000168672	FAM84B	-2.689652235	3.502380498	-4.482317005	0.000162876	0.014155195
ENSG00000162366	PDZK1IP1	-3.807827265	3.330422238	-4.479132244	0.000164186	0.014155195
ENSG00000119280	C1orf198	-1.689071162	5.305129169	-4.505540194	0.000153633	0.013678567
ENSG00000163106	HPGDS	2.610969887	1.724106318	4.46872709	0.000168541	0.014187157
ENSG00000239789	MRPS17	-2.096341672	3.475289845	-4.468191767	0.000168768	0.014187157
ENSG00000182253	SYNM	-3.549465015	3.403060678	-4.466594918	0.000169447	0.014187157
ENSG00000185479	KRT6B	-6.101275855	4.578949113	-4.485767837	0.000161468	0.014154163
ENSG00000139793	MBNL2	-1.879649939	4.459796882	-4.479772202	0.000163922	0.014155195
ENSG00000196724	ZNF418	-2.008077095	3.139035671	-4.464294162	0.000170431	0.014187157
ENSG00000160588	MPZL3	-2.689790621	3.535087041	-4.463234309	0.000170886	0.014187157
ENSG00000008323	PLEKHG6	-3.343597847	3.532198151	-4.444995308	0.00017891	0.014697765
ENSG00000092295	TGM1	-3.11622595	2.801801838	-4.433845599	0.000183999	0.014950808
ENSG00000157227	MMP14	2.109937199	9.261979734	4.523944389	0.000146682	0.013331972
ENSG00000107731	UNC5B	2.089303827	6.477267024	4.495265981	0.000157655	0.013897594
ENSG00000167123	CERCAM	1.934246376	7.472893426	4.506125551	0.000153407	0.013678567
ENSG00000143578	CREB3L4	-2.097672501	4.595298086	-4.445386273	0.000178734	0.014697765

ENSG00000257446	ZNF878	-2.54095704	3.283462147	-4.422218282	0.000189461	0.014950808
ENSG00000086717	PPEF1	3.498145089	2.429693383	4.415008021	0.000192928	0.014950808
ENSG00000079102	RUNX1T1	2.442477321	1.689427265	4.412034794	0.000194377	0.014950808
ENSG00000013588	GPRC5A	-2.878438742	4.647855698	-4.426603181	0.000187382	0.014950808
ENSG00000184454	NCMAP	-2.97269227	3.324784787	-4.400264456	0.000200218	0.015115465
ENSG00000204681	GABBR1	-2.543521704	6.739950752	-4.463564629	0.000170744	0.014187157
ENSG00000108679	LGALS3BP	2.878896875	7.398872276	4.472353328	0.00016701	0.014187157
ENSG00000178078	STAP2	-2.210713374	5.309234904	-4.427420993	0.000186997	0.014950808
ENSG00000197961	ZNF121	-1.642095741	4.898786765	-4.412980567	0.000193915	0.014950808
ENSG00000165929	TC2N	-3.062217644	4.388384567	-4.39996107	0.000200371	0.015115465
ENSG00000141349	G6PC3	1.293542344	5.600325966	4.430684352	0.000185468	0.014950808
ENSG00000101213	PTK6	-3.257842988	3.109830619	-4.378549101	0.000211458	0.015650894
ENSG00000113645	WWC1	-2.115917862	5.666088413	-4.421138139	0.000189976	0.014950808
ENSG00000131771	PPP1R1B	-4.566207613	4.645474159	-4.387584872	0.000206706	0.015371713
ENSG00000116299	KIAA1324	-3.597557272	5.684441448	-4.41680121	0.00019206	0.014950808
ENSG00000161791	FMNL3	1.811015172	6.535810743	4.434382301	0.000183751	0.014950808
ENSG00000006047	YBX2	-3.155052332	2.745624742	-4.356978354	0.000223247	0.016279027
ENSG00000131746	TNS4	-4.627663955	3.66017769	-4.358595176	0.000222341	0.016279027
ENSG00000129910	CDH15	3.980708852	2.462425164	4.355471433	0.000224095	0.016279027
ENSG00000148090	AUH	-2.718490402	4.157608575	-4.360610827	0.000221217	0.016279027
ENSG00000134755	DSC2	-2.997709485	6.055961027	-4.408529122	0.000196098	0.015009659
ENSG00000128641	MYO1B	1.869132149	7.243455429	4.424363101	0.000188441	0.014950808
ENSG00000109846	CRYAB	-3.683848756	6.853768762	-4.400736122	0.00019998	0.015115465
ENSG00000141736	ERBB2	-2.019696939	7.883644722	-4.414747758	0.000193055	0.014950808
ENSG00000182541	LIMK2	-2.300373209	6.750921594	-4.392100079	0.000204372	0.015314721
ENSG00000064270	ATP2C2	-3.23753289	2.303415003	-4.316661345	0.000247067	0.017441544
ENSG00000128606	LRRC17	3.008810582	2.82763352	4.315357239	0.000247879	0.017441544
ENSG00000125355	TMEM255A	3.029342564	1.646352217	4.312957255	0.000249379	0.017452531
ENSG00000110693	SOX6	-2.618185388	3.091695085	-4.310593243	0.000250866	0.017452531
ENSG00000136542	GALNT5	2.6790063	2.977052887	4.309791631	0.000251372	0.017452531
ENSG00000170801	HTRA3	2.618300886	5.609759035	4.348215866	0.000228221	0.016426693
ENSG00000010278	CD9	-1.956287606	8.830145135	-4.390950263	0.000204964	0.015314721
ENSG00000170374	SP7	3.434535866	1.069292004	4.291814696	0.000262992	0.018020154
ENSG00000147144	CCDC120	-1.84752418	3.970276457	-4.29482308	0.000261011	0.017962832
ENSG00000112379	KIAA1244	-2.856844043	3.074804325	-4.283291196	0.000268688	0.018286568
ENSG00000169239	CA5B	-2.489158837	3.571549081	-4.282516806	0.000269211	0.018286568
ENSG00000166963	MAP1A	2.538859187	3.006794631	4.27795336	0.000272317	0.018417796
ENSG00000185499	MUC1	-3.870419145	5.774658931	-4.330226546	0.000238782	0.017079052
ENSG00000153291	SLC25A27	-2.716275794	3.322164736	-4.266134894	0.000280527	0.018810911
ENSG00000141756	FKBP10	2.061955507	7.662570441	4.353083301	0.000225445	0.016301621
ENSG00000121310	ECHDC2	-1.670939469	6.27084977	-4.329096877	0.000239462	0.017079052
ENSG00000165323	FAT3	2.655371604	2.096527053	4.257304936	0.000286822	0.019151151
ENSG00000066735	KIF26A	3.196098863	2.666455957	4.253182018	0.000289809	0.019268602
ENSG00000143369	ECM1	2.168400998	5.915191409	4.303684763	0.000255261	0.017644476
ENSG00000150961	SEC24D	1.358534417	7.188517331	4.318714629	0.000245795	0.017441544
ENSG00000139055	ERP27	-3.579111897	3.016179545	-4.229090977	0.000307892	0.020293984
ENSG00000239665	RP11-295P9.3	-2.094962402	2.808814908	-4.221639783	0.00031371	0.020293984
ENSG00000163993	S100P	-4.428251671	3.496233524	-4.225300185	0.000310838	0.020293984
ENSG00000227184	EPPK1	-3.181191052	2.927863321	-4.220910981	0.000314285	0.020293984
ENSG00000158106	RHPN1	-3.101072559	5.33221868	-4.246590966	0.000294648	0.019507719
ENSG00000047648	ARHGAP6	2.327107027	2.140748203	4.194880585	0.000335517	0.02141803
ENSG00000197647	ZNF433	-2.245238135	2.739558897	-4.189451252	0.000340123	0.02141803
ENSG00000105855	ITGB8	-2.783459322	3.625650066	-4.191660088	0.000338242	0.02141803
ENSG00000149257	SERPINH1	1.91087859	7.64248363	4.26627282	0.00028043	0.018810911
ENSG00000197261	C6orf141	-2.677097903	2.25715371	-4.160944411	0.000365355	0.022481525
ENSG00000151690	MFSD6	-2.223516658	3.759818363	-4.168252745	0.000358714	0.022307217
ENSG00000135374	ELF5	-3.666209709	2.621006099	-4.159062512	0.000367085	0.022499727
ENSG00000189143	CLDN4	-3.232561755	2.688758987	-4.155923962	0.000369988	0.022589415
ENSG00000110330	BIRC2	-1.328578227	6.29748608	-4.223766306	0.000312038	0.020293984
ENSG00000124813	RUNX2	2.782354596	3.755445527	4.151961374	0.000373686	0.022726745
ENSG00000106479	ZNF862	-2.189006825	3.565052581	-4.146905583	0.000378457	0.022928048
ENSG00000112378	PERP	-3.132091724	5.339152303	-4.195746781	0.000334788	0.02141803
ENSG00000102854	MSLN	-5.05488347	4.541955842	-4.16718258	0.000359679	0.022307217
ENSG00000121057	AKAP1	-1.974663021	5.809936965	-4.193251965	0.000336892	0.02141803
ENSG00000164828	SUN1	-1.548873955	7.859936883	-4.223316282	0.000312391	0.020293984

ENSG00000105289	TJP3	-3.393011355	3.308433631	-4.118774749	0.00040613	0.024395047
ENSG00000198429	ZNF69	-2.412339154	3.094969717	-4.114559318	0.000410447	0.024395169
ENSG00000164823	OSGIN2	-2.111031813	4.06622474	-4.117593427	0.000407336	0.024395047
ENSG00000197822	OCLN	-2.487325452	3.533025918	-4.098410225	0.000427409	0.025117902
ENSG00000042445	RETSAT	-1.525906862	7.279570939	-4.188137072	0.000341247	0.02141803
ENSG00000161544	CYGB	2.168531313	3.425115268	4.093379512	0.000432834	0.025247587
ENSG00000035115	SH3YL1	-1.967074008	6.276187968	-4.162106973	0.000364291	0.022481525
ENSG00000117335	CD46	-1.54150729	6.891457091	-4.167902383	0.00035903	0.022307217
ENSG00000011028	MRC2	1.892165137	9.29151833	4.1899619	0.000339687	0.02141803
ENSG00000154027	AK5	2.796660502	3.153441409	4.072507034	0.000456081	0.026118148
ENSG00000050165	DKK3	2.475500951	5.806774137	4.137406229	0.000387586	0.023390791
ENSG00000157703	SVOPL	-2.410993096	1.829390783	-4.063626696	0.000466344	0.026414752
ENSG00000163909	HEYL	2.549478781	3.03221243	4.059918706	0.000470696	0.026472031
ENSG00000066923	STAG3	-1.965646868	4.456383326	-4.081735629	0.000445654	0.025803521
ENSG00000160307	S100B	-4.962285833	4.346149283	-4.069697784	0.000459303	0.026207015
ENSG00000115290	GRB14	-2.981345777	2.91482501	-4.044353406	0.000489411	0.02713552
ENSG00000115339	GALNT3	-3.149010945	4.682464989	-4.078603154	0.000449166	0.02591129
ENSG00000186007	LEMD1	-3.251683961	2.412035627	-4.03764075	0.000497708	0.027498377
ENSG00000115295	CLIP4	-2.212327824	4.75478651	-4.075334603	0.000452861	0.026028724
ENSG00000134285	FKBP11	1.319594695	5.579874473	4.099495946	0.000426247	0.025117902
ENSG00000136490	LIMD2	2.1248815	4.43841542	4.063139355	0.000466914	0.026414752
ENSG00000095970	TREM2	2.802554383	4.238846448	4.055744649	0.000475644	0.026654763
ENSG00000125731	SH2D3A	-2.073605532	4.725394841	-4.062216458	0.000467994	0.026414752
ENSG00000065618	COL17A1	-5.368970615	4.326055938	-4.034806516	0.000501253	0.027551025
ENSG00000134569	LRP4	2.369660951	4.811671507	4.048397743	0.000484479	0.026957293
ENSG00000100201	DDX17	-1.196479939	8.606979475	-4.115548665	0.00040943	0.024395169
ENSG00000163132	MSX1	2.760972635	2.600550435	3.996354586	0.000551892	0.029355049
ENSG00000213445	SIPA1	1.676015736	6.284881265	4.082133175	0.00044521	0.025803521
ENSG00000175707	C1orf172	-3.09238034	2.538891654	-3.993517566	0.000555823	0.029365049
ENSG00000034510	TMSB10	1.52240069	7.58871239	4.099516405	0.000426226	0.025117902
ENSG00000180921	FAM83H	-2.08192873	5.266212419	-4.049440341	0.000483215	0.026957293
ENSG00000119411	BSPRY	-3.359192377	3.099690541	-3.989115346	0.000561978	0.029590573
ENSG00000133800	LYVE1	2.305037231	1.91659213	3.980629234	0.000574033	0.030023826
ENSG00000019991	HGF	3.323504395	2.845991817	3.977945278	0.000577899	0.030028391
ENSG00000134709	HOOK1	-3.168515993	2.573596517	-3.976448689	0.000580065	0.030038958
ENSG00000163466	ARPC2	1.073657753	8.95451901	4.096304054	0.000429672	0.025156669
ENSG00000006555	TTC22	-2.540234415	2.758893515	-3.967666008	0.000592944	0.030504532
ENSG00000119865	CNRIP1	2.498287448	2.716996259	3.95829137	0.000607003	0.031024368
ENSG00000104332	SFRP1	-3.149107023	4.739036269	-3.99498845	0.000553782	0.029356036
ENSG00000177694	NAALADL2	-2.494624933	2.985537536	-3.952088582	0.000616486	0.031406748
ENSG00000196517	SLC6A9	-2.197265133	5.0104731	-3.999299786	0.00054784	0.029291924
ENSG00000046604	DSG2	-2.367331266	6.304232382	-4.0340744	0.000502173	0.027551025
ENSG00000090776	EFNB1	1.985798061	5.381466929	4.008541356	0.000535317	0.028864821
ENSG00000112232	KHDRBS2	1.766508507	0.521698043	3.962605925	0.000600492	0.030791888
ENSG00000162813	BPNT1	-1.233456547	6.349772533	-4.02924565	0.000508281	0.027788984
ENSG00000168300	PCMTD1	-1.670205011	5.064142452	-3.985933299	0.000566468	0.02972727
ENSG00000204175	GPRIN2	-2.344454089	2.206653568	-3.927844486	0.000654977	0.031884909
ENSG00000034677	RNF19A	-1.484779362	5.483347585	-3.99857227	0.000548839	0.029291924
ENSG00000154736	ADAMTS5	2.499083727	1.696654132	3.927004652	0.000656352	0.031884909
ENSG00000184060	ADAP2	2.058543047	4.055649265	3.942910577	0.000630787	0.031884909
ENSG00000008735	MAPK8IP2	-3.237644296	3.887892512	-3.935475343	0.000642613	0.031884909
ENSG00000178522	AMBN	4.424334031	1.630373654	3.917016158	0.000672928	0.032389297
ENSG00000213064	SFT2D2	-1.718309226	5.27546891	-3.977911394	0.000577947	0.030028391
ENSG00000178538	CA8	-3.144279864	3.272349717	-3.919411045	0.000668916	0.032324482
ENSG00000139329	LUM	3.173019252	7.95029641	4.026390247	0.000511928	0.027891172
ENSG00000143178	TBX19	-2.121239861	2.721857089	-3.908545321	0.000687309	0.032708848
ENSG00000198929	NOS1AP	-2.179208383	2.223998083	-3.905956274	0.000691764	0.032708848
ENSG00000168487	BMP1	1.798100577	7.230676476	4.01172519	0.000531069	0.028833916
ENSG00000105519	CAPS	-2.391678439	4.801973783	-3.945750523	0.000626327	0.031804842
ENSG00000100167	SEPT3	-2.846337209	3.643249285	-3.905776708	0.000692074	0.032708848
ENSG00000166033	HTRA1	1.669951952	7.457130657	4.003959224	0.000541491	0.029097704
ENSG00000082781	ITGB5	1.776919562	8.229501549	4.009525196	0.000534001	0.028864821
ENSG00000149485	FADS1	1.590997638	5.73335325	3.968425962	0.000591818	0.030504532
ENSG00000105707	HPN	-4.056855439	2.854762516	-3.887836096	0.000723757	0.033698743
ENSG00000087916	SLC6A14	-3.48088104	2.392306134	-3.880410112	0.000737287	0.034227134

ENSG00000128714	HOXD13	1.934462371	0.85248671	3.896419777	0.000708424	0.033181721
ENSG00000167785	ZNF558	-1.598218337	4.304901614	-3.908665897	0.000687102	0.032708848
ENSG00000102230	PCYT1B	2.18523155	1.974119994	3.86848901	0.000759531	0.034785013
ENSG00000174428	GTF2IRD2B	-1.630086856	5.160332578	-3.929408415	0.000652424	0.031884909
ENSG00000104231	ZFAND1	-1.746456681	5.403939161	-3.936413715	0.000641108	0.031884909
ENSG00000142444	C19orf52	-1.833597915	3.262650703	-3.870035884	0.000756607	0.034785013
ENSG00000108244	KRT23	-5.250939315	4.941162286	-3.912926162	0.000679834	0.032621627
ENSG00000084070	SMAP2	1.456311324	5.451522355	3.931211368	0.000649493	0.031884909
ENSG00000054598	FOXC1	-2.060174141	2.470047187	-3.855709597	0.000784112	0.035559255
ENSG00000164695	CHMP4C	-2.5824594	3.727939667	-3.870548932	0.00075564	0.034785013
ENSG00000130940	CASZ1	-2.691046324	3.37101066	-3.859223279	0.000777276	0.035351425
ENSG00000154143	PANX3	2.889677328	0.902552877	3.868036141	0.000760389	0.034785013
ENSG00000081277	PKP1	-3.787091104	5.050024023	-3.902095797	0.000698461	0.032911566
ENSG00000155130	MARCKS	2.04596558	5.847092075	3.928862862	0.000653314	0.031884909
ENSG00000205336	GPR56	-3.037928038	6.213740427	-3.932936373	0.000646701	0.031884909
ENSG00000169242	EFNA1	-1.552147137	6.403745931	-3.934622251	0.000643983	0.031884909
ENSG00000104892	KLC3	-2.384939589	2.964408732	-3.827337731	0.000841527	0.037406215
ENSG00000117602	RCAN3	-1.860181877	3.749755816	-3.840822636	0.000813739	0.036585593
ENSG00000170421	KRT8	-2.03250566	4.428606681	-3.861200468	0.000773456	0.035279917
ENSG00000136717	BIN1	1.796778827	6.239019887	3.919049044	0.000669521	0.032324482
ENSG00000155511	GRIA1	2.173626463	0.612666058	3.848129803	0.000799061	0.03613276
ENSG00000161249	DMKN	-3.694843533	5.870086219	-3.905850562	0.000691947	0.032708848
ENSG00000171346	KRT15	-4.990947378	6.868122133	-3.927410541	0.000655687	0.031884909
ENSG00000164050	PLXNB1	-1.87137545	7.971168981	-3.940924566	0.000633924	0.031884909
ENSG00000102226	USP11	1.224137377	7.664339691	3.936326189	0.000641248	0.031884909
ENSG00000102554	KLF5	-2.219421814	4.890257892	-3.87139973	0.000754039	0.034785013
ENSG00000104368	PLAT	2.306228253	5.631971792	3.894550402	0.000711736	0.033237626
ENSG00000125850	OVOL2	-2.539170416	1.86266289	-3.812246316	0.000873736	0.038295497
ENSG00000133574	GIMAP4	1.9530085	1.356497986	3.818463371	0.000860322	0.037919428
ENSG00000164318	EGFLAM	1.840829522	3.667081638	3.822590783	0.00085153	0.037743927
ENSG00000160183	TMPRSS3	-3.600052361	4.065911312	-3.833919448	0.000827849	0.037113645
ENSG00000204262	COL5A2	2.52205152	9.875793977	3.94052499	0.000634557	0.031884909
ENSG00000166450	PRTG	1.88676253	0.730187485	3.821267344	0.000854339	0.037761793
ENSG00000044524	EPHA3	2.878338583	2.581059459	3.794136249	0.000913995	0.039657336
ENSG00000104140	RHOV	-2.963409602	2.476166568	-3.784493808	0.000936171	0.040135114
ENSG00000135414	GDF11	2.405723873	2.361502094	3.781435091	0.000943315	0.040187422
ENSG00000203930	LINC00632	1.95560633	0.782721884	3.79871836	0.000903639	0.039495833
ENSG00000176293	ZNF135	-1.94014121	2.191145713	-3.775036798	0.000958434	0.04042686
ENSG00000188522	FAM83G	-1.795492934	3.041977042	-3.779489826	0.000947887	0.040198081
ENSG00000170558	CDH2	3.619955607	3.424294441	3.783126781	0.000939357	0.040161991
ENSG00000215252	GOLGA8B	-2.503747177	2.136741746	-3.767006745	0.000977747	0.040911558
ENSG00000166428	PLD4	2.931248237	2.475121703	3.761610023	0.000990942	0.040959921
ENSG00000117115	PADI2	-2.803557809	5.218474707	-3.827461091	0.000841268	0.037406215
ENSG00000167741	GGT6	-2.968403394	2.455361954	-3.755364024	0.001006432	0.040959921
ENSG00000145020	AMT	-2.597228712	3.686779802	-3.771564192	0.00096674	0.040667862
ENSG00000269343	ZNF587B	-2.096593844	3.377596032	-3.759952581	0.000995029	0.040959921
ENSG00000196839	ADA	1.838357808	4.23145477	3.786788962	0.000930845	0.040040822
ENSG00000147697	GSDMC	-3.519449007	3.175792436	-3.755132601	0.00100701	0.040959921
ENSG00000143126	CELSR2	-2.474148125	5.974312673	-3.84290835	0.000809522	0.036500612
ENSG00000038210	PI4K2B	-1.369169582	4.574855355	-3.792621508	0.000917444	0.039657336
ENSG00000106066	CPVL	2.362096273	5.726228871	3.832338376	0.000831114	0.03715389
ENSG00000151790	TDO2	3.767140557	3.255757651	3.753025715	0.001012292	0.040959921
ENSG00000168542	COL3A1	2.948251036	12.84084138	3.896806465	0.000707741	0.033181721
ENSG00000135315	KIAA1009	-1.570738994	4.018982614	-3.761635944	0.000990878	0.040959921
ENSG00000103647	CORO2B	2.368575326	2.165425829	3.732109236	0.001066226	0.04184893
ENSG00000114805	PLCH1	-2.438786663	2.499713099	-3.729601086	0.001072881	0.041856643
ENSG00000198807	PAX9	1.808754533	0.668903945	3.754792594	0.001007861	0.040959921
ENSG00000184363	PKP3	-3.62174555	5.033731431	-3.793285691	0.00091593	0.039657336
ENSG00000150394	CDH8	2.199228463	1.029947745	3.738399381	0.001049716	0.041804816
ENSG00000005379	BZRAP1	-2.762998598	5.233836608	-3.786540673	0.000931419	0.040040822
ENSG00000198797	BRINP2	1.803371313	0.901857263	3.731848666	0.001066916	0.04184893
ENSG00000204370	SDHD	-1.905073083	2.062979069	-3.710131045	0.001125945	0.042469223
ENSG00000196189	SEMA4A	-2.18200147	4.645768427	-3.758151291	0.00099949	0.040959921
ENSG00000059377	TBXAS1	2.67747911	4.499230369	3.751777039	0.001015435	0.040959921
ENSG00000164855	TMEM184A	-2.635530949	2.999109202	-3.706004482	0.001137518	0.042802875

ENSG00000008226	DLEC1	-2.546463267	1.85886364	-3.704392451	0.001142071	0.042807368
ENSG00000105894	PTN	3.084518991	4.488244312	3.746813842	0.001028023	0.04131946
ENSG00000198598	MMP17	1.888053674	2.813459871	3.698424327	0.001159084	0.043097585
ENSG00000184371	CSF1	2.527374509	5.160901998	3.768444748	0.000974261	0.040874672
ENSG00000157734	SNX22	-3.566111755	4.80424293	-3.746151669	0.001029714	0.04131946
ENSG00000103150	MLYCD	1.654870331	0.888099142	3.710635332	0.001124538	0.042469223
ENSG00000215845	TSTD1	-1.800073651	4.083794069	-3.717481727	0.001105614	0.042209723
ENSG00000076554	TPD52	-1.949786022	6.633948938	-3.797478972	0.000906429	0.039507709
ENSG00000137203	TFAP2A	-1.362592309	5.915925086	-3.780685475	0.000945074	0.040187422
ENSG00000157404	KIT	-2.912514083	4.110520594	-3.714077447	0.001114985	0.042258992
ENSG00000170275	CRTAP	1.267143324	8.128976009	3.812951347	0.000872204	0.038295497
ENSG00000189180	ZNF33A	-1.557745087	4.720114022	-3.732847056	0.001064277	0.04184893
ENSG00000198515	CNGA1	-3.025036955	2.443037927	-3.678444307	0.00121787	0.044753157
ENSG00000105852	PON3	-2.757594182	2.607388868	-3.671727949	0.001238281	0.045217819
ENSG00000154079	C6orf57	-1.842410967	2.94709333	-3.674906441	0.00122858	0.045041246
ENSG00000169855	ROBO1	1.803106459	5.706174955	3.762825948	0.000987954	0.040959921
ENSG00000064886	CHI3L2	-4.123787591	5.305080396	-3.745155792	0.001032262	0.04131946
ENSG00000088836	SLC4A11	-2.978358371	4.700468925	-3.718858771	0.001101846	0.042168462
ENSG00000120088	CRHR1	-2.920152731	2.134581453	-3.664690447	0.001260029	0.04587267
ENSG00000135164	DMTF1	-1.404015667	6.518164431	-3.776219124	0.000955623	0.040416909
ENSG00000173715	C11orf80	-1.48701107	5.359429943	-3.741709438	0.001041129	0.041568348
ENSG00000166016	ABTB2	-1.577121672	5.015306666	-3.726704885	0.001080615	0.041866513
ENSG00000132359	RAP1GAP2	-2.560049621	4.004276231	-3.686316517	0.001194366	0.044200001
ENSG00000145934	TENM2	3.433323885	2.238851634	3.655826309	0.001287956	0.046375097
ENSG00000124191	TOX2	3.089921154	2.075109887	3.655267669	0.001289737	0.046375097
ENSG00000130787	HIP1R	-1.579921733	5.913432445	-3.751884592	0.001015164	0.040959921
ENSG00000151150	ANK3	-1.706307705	4.443323387	-3.698807682	0.001157983	0.043097585
ENSG00000164930	FZD6	-2.429393538	5.104810406	-3.724059388	0.001087729	0.04193501
ENSG00000106537	TSPAN13	-1.707194565	5.492756323	-3.735599048	0.001057035	0.04184893
ENSG00000184224	C11orf72	-2.293820795	3.427090139	-3.658777811	0.001278591	0.046333408
ENSG00000112414	GPR126	-2.694509574	3.224887014	-3.652389542	0.001298947	0.046533729
ENSG00000198189	HSD17B11	1.680110594	3.496201742	3.656794172	0.001284878	0.046375097
ENSG00000116132	PRRX1	1.62758297	5.127445604	3.714361028	0.001114201	0.042258992
ENSG00000198087	CD2AP	-1.597936903	5.40374469	-3.722969567	0.001090672	0.041945435
ENSG00000138758	SEPT11	1.473659874	6.52798224	3.751771425	0.001015449	0.040959921
ENSG00000134686	PHC2	1.200157668	6.700267604	3.753101824	0.0010121	0.040959921
ENSG00000185864	NPIPB4	-1.858910892	3.738279218	-3.65469527	0.001291563	0.046375097
ENSG00000112183	RBM24	1.409418066	0.664813302	3.660080106	0.001274479	0.046291337
ENSG00000113721	PDGFRB	1.717769364	7.521583399	3.757332177	0.001001525	0.040959921
ENSG00000104412	EMC2	-1.207591035	6.160107187	-3.732317318	0.001065676	0.04184893
ENSG00000164330	EBF1	2.296396098	3.831912112	3.646858514	0.001316828	0.047066851
ENSG00000108819	PPP1R9B	1.253400493	6.338797724	3.73327026	0.00106316	0.04184893
ENSG00000140022	STON2	2.023720987	1.771243468	3.624768689	0.001390682	0.04925776
ENSG00000181991	MRPS11	1.259746517	5.048934445	3.691747488	0.001178412	0.04371266
ENSG00000168575	SLC20A2	1.341412351	6.072729603	3.725104153	0.001084914	0.04192952
ENSG00000104313	EYA1	2.956079341	2.282945125	3.618103786	0.001413752	0.049856908
ENSG00000215012	C22orf29	-1.982366786	2.388757643	-3.613125056	0.00143123	0.049905404
ENSG00000162413	KLHL21	-1.849484313	4.725905653	-3.671441266	0.00123916	0.045217819
ENSG00000092929	UNC13D	-1.557727359	6.516103904	-3.728572135	0.001075622	0.041856643
ENSG00000128487	SPECC1	1.739613544	4.946555793	3.681244707	0.001209457	0.044653165
ENSG00000184347	SLIT3	1.857493878	6.374363735	3.719990742	0.001098758	0.042153089
ENSG00000143110	C1orf162	2.267279223	2.809938231	3.607007567	0.001452996	0.050525724
ENSG00000137460	FHDC1	-2.18927221	2.828078112	-3.606320665	0.00145546	0.050525724
ENSG00000011600	TYROBP	2.474895916	7.106059719	3.730875941	0.001069493	0.04184893
ENSG00000101298	SNPH	1.612070575	0.500370391	3.632503438	0.001364371	0.048435168
ENSG00000215915	ATAD3C	-2.632919668	4.324832548	-3.633852234	0.001359833	0.048435168
ENSG00000082458	DLG3	-2.014436986	4.383910697	-3.632866946	0.001363146	0.048435168
ENSG00000105991	HOXA1	1.75788893	0.758742004	3.614915528	0.00142492	0.049856908
ENSG00000100979	PLTP	2.162245943	8.139074443	3.727796724	0.001077693	0.041856643
ENSG00000085563	ABCB1	2.362504112	2.176316587	3.57472792	0.00157333	0.053480056
ENSG00000182134	TDRKH	-1.585781665	4.248469064	-3.614796585	0.001425339	0.049856908
ENSG00000011347	SYT7	-2.883369568	3.59817579	-3.593178038	0.001503404	0.051748514
ENSG00000049089	COL9A2	-3.394052131	7.378265287	-3.70083204	0.001152191	0.04304529
ENSG00000114993	RTKN	-1.841914818	6.34749544	-3.678915353	0.001216451	0.044753157
ENSG00000132470	ITGB4	-3.415881448	7.873610011	-3.704031192	0.001143094	0.042807368

ENSG00000074706	IPCEF1	1.709969769	1.003355496	3.582845808	0.001542179	0.05283479
ENSG00000003249	DBNDD1	-3.114948553	2.877732965	-3.564382408	0.001613922	0.053995853
ENSG00000116774	OLFML3	2.170482917	3.521936875	3.572634113	0.001581464	0.053480056
ENSG00000272325	NUDT3	-1.535237767	3.624087818	-3.573313838	0.001578819	0.053480056
ENSG00000162105	SHANK2	-3.124690244	2.896203334	-3.555978111	0.001647651	0.054291152
ENSG00000135404	CD63	1.007101346	10.93100826	3.715313229	0.001111574	0.042258992
ENSG00000119718	EIF2B2	1.394031605	3.983560904	3.579623576	0.001554471	0.05302435
ENSG00000139330	KERA	2.853265151	1.015110824	3.560223569	0.001630528	0.054090084
ENSG00000169884	WNT10B	2.234771542	1.096588584	3.554442353	0.001653889	0.054291152
ENSG00000001461	NIPAL3	-2.149137781	5.285814241	-3.616142616	0.001420612	0.049856908
ENSG00000180071	ANKRD18A	-1.948570337	2.097038671	-3.530954806	0.001752214	0.056645398
ENSG00000154556	SORBS2	-2.939573855	5.334221817	-3.614420558	0.001426662	0.049856908
ENSG00000185950	IRS2	-2.503459019	2.483679106	-3.526201966	0.001772795	0.056797817
ENSG00000170442	KRT86	-3.135725927	3.924670383	-3.55701063	0.001643471	0.054291152
ENSG00000174607	UGT8	-2.266530021	1.668375483	-3.525377534	0.001776389	0.056797817
ENSG00000140403	DNAJA4	-1.881492244	4.95535028	-3.59305069	0.001503876	0.051748514
ENSG00000180758	GPR157	-1.704348127	4.290293644	-3.561273249	0.001626321	0.05406483
ENSG00000139926	FRMD6	1.702515352	5.757944377	3.618016452	0.001414057	0.049856908
ENSG00000167861	HID1	-2.160766233	4.144806024	-3.553399169	0.001658139	0.054317035
ENSG00000169851	PCDH7	2.406671361	2.10364889	3.507696063	0.001855205	0.058453856
ENSG00000101236	RNF24	-1.492529632	5.416892872	-3.59761725	0.00148704	0.051394575
ENSG00000072954	TMEM38A	-1.849291979	3.724880975	-3.526058075	0.001773421	0.056797817
ENSG00000165238	WNK2	-3.184049868	4.815388009	-3.568466005	0.001597778	0.053799876
ENSG00000151617	EDNRA	2.259956027	3.948300385	3.530427021	0.001754488	0.056645398
ENSG00000134668	SPOCD1	2.565734987	2.546692242	3.498238561	0.001898755	0.059349322
ENSG00000029534	ANK1	2.191522531	1.73001812	3.499473962	0.00189301	0.059287855
ENSG00000005249	PRKAR2B	-2.206556026	4.060093286	-3.529187092	0.001759841	0.056701566
ENSG00000087510	TFAP2C	-2.516388359	4.506897969	-3.544105484	0.001696478	0.055108683
ENSG00000143850	PLEKHA6	-2.280370023	3.881001112	-3.51241292	0.001833851	0.058014028
ENSG00000127129	EDN2	-2.724057408	2.459294269	-3.480690738	0.001982219	0.061012495
ENSG00000104447	TRPS1	-1.975671188	5.204562849	-3.562135781	0.001622872	0.054064721
ENSG00000164830	OXR1	-1.612617245	5.151216042	-3.555524549	0.001649491	0.054291152
ENSG00000174684	B3GNT1	2.419826562	2.070775498	3.471527279	0.002027217	0.062039888
ENSG00000116729	WLS	2.025675021	6.552317194	3.597815486	0.001486313	0.051394575
ENSG00000070159	PTPN3	-1.526301063	5.442443524	-3.562547973	0.001621226	0.054064721
ENSG00000165912	PACSIN3	-1.211447512	5.949338819	-3.572843783	0.001580647	0.053480056
ENSG00000105767	CADM4	-1.783524679	5.376301991	-3.550961103	0.001668114	0.054416581
ENSG00000128833	MYO5C	-2.476379662	4.766918358	-3.52337281	0.001785158	0.056932739
ENSG00000182272	B4GALNT4	-2.47239984	6.170356072	-3.568639873	0.001597095	0.053799876
ENSG00000088448	ANKRD10	-1.777486404	5.578308351	-3.54970926	0.001673258	0.054471156
ENSG00000115112	TFCP2L1	-3.437393455	3.267134027	-3.463002344	0.002069974	0.062702618
ENSG00000168952	STXBP6	2.495538383	2.341215016	3.444690622	0.002164811	0.064948481
ENSG00000250565	ATP6V1E2	-1.760071776	2.637373509	-3.445335504	0.002161401	0.064948481
ENSG00000006453	BAIAP2L1	-2.114966104	5.942210532	-3.554609715	0.001653208	0.054291152
ENSG00000131037	EPS8L1	-2.425420322	4.293010481	-3.488277403	0.001945703	0.060217021
ENSG00000143387	CTSK	2.564067617	7.666368949	3.586417337	0.001528666	0.052486442
ENSG00000186648	LRRC16B	-2.872858731	2.752151308	-3.438348549	0.002198635	0.065711967
ENSG00000087303	NID2	1.797151115	6.625461571	3.564928736	0.001611753	0.053995853
ENSG00000205138	SDHAF1	1.54420956	0.686836567	3.466834632	0.002050645	0.062600529
ENSG00000070018	LRP6	-2.01844	5.223548658	-3.519193899	0.001803573	0.057171444
ENSG00000140545	MFGE8	-2.162848788	8.755678812	-3.581951512	0.001545581	0.052835973
ENSG00000187554	TLR5	-1.866251332	2.45604858	-3.421026042	0.002293666	0.066647994
ENSG00000049283	EPN3	-2.841587244	2.708376124	-3.423810147	0.002278127	0.066481509
ENSG00000095066	HOOK2	-1.53385853	7.485318361	-3.564913343	0.001611814	0.053995853
ENSG00000115457	IGFBP2	2.121493984	6.459546702	3.543294049	0.001699866	0.055108683
ENSG00000148848	ADAM12	2.228436957	5.588876146	3.519251076	0.00180332	0.057171444
ENSG00000135362	PRR5L	2.484458573	2.907542447	3.418720823	0.002306611	0.066649596
ENSG00000185189	NRBP2	-2.127487577	7.065582524	-3.551839875	0.001664512	0.054412199
ENSG00000136883	KIF12	-3.576385404	3.10497081	-3.424956695	0.002271758	0.066481509
ENSG00000187595	ZNF385C	-3.470375069	3.010524633	-3.423570105	0.002279463	0.066481509
ENSG00000139835	GRTP1	-1.920396095	3.295860275	-3.426327237	0.002264167	0.066481509
ENSG00000068976	PYGM	2.304784415	2.843282232	3.41219102	0.002343664	0.067197445
ENSG00000153558	FBXL2	-2.051309828	4.461961791	-3.464340506	0.002063204	0.062702618
ENSG00000054690	PLEKHH1	-2.020632032	3.434699257	-3.423808094	0.002278139	0.066481509
ENSG00000198947	DMD	-2.644178307	5.118705492	-3.490808609	0.001933667	0.06008152

ENSG00000119139	TJP2	-1.328560111	6.040188955	-3.521891671	0.001791663	0.057024324
ENSG00000116039	ATP6V1B1	-1.929892839	4.336432391	-3.452878689	0.002121892	0.063905203
ENSG00000254206	NPIPB11	-1.79934987	1.332936932	-3.411636835	0.002346836	0.067197445
ENSG00000174807	CD248	2.528418761	2.422063426	3.395356495	0.002441887	0.068543208
ENSG00000160678	S100A1	-2.184249819	1.715748381	-3.403149845	0.002395928	0.068052664
ENSG00000254470	AP5B1	-1.986671421	2.948486259	-3.396469907	0.002435269	0.068479939
ENSG00000127824	TUBA4A	-1.361841086	5.461936714	-3.491529843	0.001930251	0.06008152
ENSG00000099250	NRP1	1.554735376	6.117099957	3.509047708	0.001849061	0.058377502
ENSG00000147119	CHST7	1.805652349	1.31160928	3.401987929	0.002402726	0.068052664
ENSG00000165175	MID1IP1	1.667883436	3.537918723	3.404512122	0.002387981	0.068003286
ENSG00000165209	STRBP	-1.543411927	5.676898942	-3.491612108	0.001929861	0.06008152
ENSG00000114841	DNAH1	-1.923749978	5.176021621	-3.471303964	0.002028326	0.062039888
ENSG00000102547	CAB39L	-1.95406019	3.946214547	-3.417361964	0.002314274	0.066649596
ENSG00000174093	RP11-1407O15.2	-1.40187084	4.235641648	-3.428631784	0.002251458	0.066481509
ENSG00000135702	CHST5	-1.785871616	1.31160928	-3.396757946	0.00243356	0.068479939
ENSG00000135063	FAM189A2	-2.490077699	3.145499862	-3.389619473	0.002476267	0.069260445
ENSG00000163293	NIPAL1	-1.877875852	1.386786396	-3.393557976	0.002452615	0.068721393
ENSG00000167889	MGAT5B	2.582029743	2.854700366	3.383792066	0.002511671	0.06965558
ENSG00000139631	CSAD	-2.14963005	6.253452566	-3.50050082	0.001888247	0.059256982
ENSG00000152939	MARVELD2	-1.639404555	4.566590886	-3.43481888	0.002217682	0.065904641
ENSG00000164093	PITX2	2.165689576	1.375276041	3.38505887	0.002503933	0.06965558
ENSG00000140931	CMTM3	1.956369836	5.093102748	3.454641718	0.00211276	0.063752539
ENSG00000197978	GOLGA6L9	-1.920648417	4.819536705	-3.439319303	0.002193425	0.065681347
ENSG00000182054	IDH2	0.921204348	8.44855496	3.52516706	0.001777307	0.056797817
ENSG00000164294	GPX8	2.058203439	4.146481874	3.406926953	0.002373957	0.067850208
ENSG00000071991	CDH19	-2.92675874	3.012946423	-3.367897756	0.002610757	0.071244151
ENSG00000068323	TFE3	1.084925096	6.225320418	3.485960074	0.001956787	0.060440827
ENSG00000125869	LAMP5	2.507630281	2.415421255	3.357707121	0.002676275	0.071975353
ENSG00000129657	SEC14L1	1.222276288	5.385150836	3.459114198	0.002089766	0.063180174
ENSG00000142549	IGLON5	1.803870339	0.550252562	3.399187975	0.002419186	0.068385199
ENSG00000091622	PITPNM3	-2.172046986	3.003907704	-3.360655256	0.002657158	0.071791294
ENSG00000139083	ETV6	-1.347866705	5.713728043	-3.463731643	0.002066282	0.062702618
ENSG00000177707	PVRL3	2.197608412	2.157238328	3.346829298	0.002747974	0.073446975
ENSG00000003147	ICA1	-2.117853535	4.635016849	-3.412838432	0.002339965	0.067197445
ENSG00000150076	C10ORF68	-1.785735282	1.760414363	-3.344432639	0.002764021	0.073508893
ENSG00000011465	DCN	2.29304858	8.347622459	3.502381565	0.001879555	0.059102415
ENSG00000137709	POU2F3	-2.397184413	2.0414374	-3.340116464	0.002793148	0.073842538
ENSG00000135205	CCDC146	-1.730017902	4.112300844	-3.383641775	0.002512591	0.06965558
ENSG00000114948	ADAM23	3.390681375	2.690592962	3.341087649	0.002786569	0.073842538
ENSG00000109436	TBC1D9	1.805778604	4.871293649	3.41835705	0.00230866	0.066649596
ENSG00000103653	CSK	1.22409683	6.901651578	3.480515302	0.001983071	0.061012495
ENSG00000140937	CDH11	1.612166927	7.529283146	3.488727273	0.001943559	0.060217021
ENSG00000133606	MKRN1	-1.345823467	4.110793375	-3.378877256	0.002541913	0.070220341
ENSG00000175318	GRAMD2	-2.935090758	3.18518493	-3.346177279	0.002752331	0.073446975
ENSG00000135905	DOCK10	1.383296457	5.500300186	3.437082388	0.002205449	0.06579031
ENSG00000005471	ABCB4	2.466827158	2.957314853	3.335620501	0.002823807	0.074320368
ENSG00000149089	APIP	-1.903567075	2.674220559	-3.330056114	0.002862203	0.074726846
ENSG00000244754	N4BP2L2	-1.370042178	6.526282	-3.463392283	0.002067999	0.062702618
ENSG00000138792	ENPEP	2.089236427	4.158576103	3.372366069	0.002582523	0.070719677
ENSG00000111319	SCNN1A	-3.042576587	3.479685328	-3.352597122	0.002709727	0.072680906
ENSG00000114796	KLHL24	-1.129036218	5.213661886	-3.418999382	0.002305043	0.066649596
ENSG00000241935	HOGA1	-1.522877049	1.146470755	-3.346294456	0.002751547	0.073446975
ENSG00000115216	NRBP1	0.889307923	7.79484953	3.477923499	0.001995704	0.061281003
ENSG00000164989	CCDC171	-1.804728832	2.626436572	-3.319279468	0.002938014	0.075327415
ENSG00000158042	MRPL17	1.24548277	5.571059007	3.427965707	0.002255124	0.066481509
ENSG00000177685	EFCAB4A	-2.445789306	4.43246079	-3.379119947	0.002540411	0.070220341
ENSG00000123836	PFKFB2	-1.870948552	4.111581523	-3.360842365	0.002655949	0.071791294
ENSG00000172159	FRMD3	-2.418511933	3.091479135	-3.327116144	0.002882695	0.075066743
ENSG00000184381	PLA2G6	-1.900109538	5.137042036	-3.404769671	0.002386482	0.068003286
ENSG00000172058	SERF1A	2.020143831	1.484468071	3.323666232	0.002906922	0.075268174
ENSG00000132793	LPIN3	-1.787501939	5.768785152	-3.423628844	0.002279136	0.066481509
ENSG00000134253	TRIM45	-2.176959583	4.606438173	-3.372885014	0.002579264	0.070719677
ENSG00000078070	MCCC1	-1.400931508	5.944102155	-3.427706901	0.00225655	0.066481509
ENSG00000171150	SOCS5	-1.670094247	1.722899086	-3.311039771	0.002997289	0.076348157
ENSG00000185842	DNAH14	-1.842258226	4.373727058	-3.362117643	0.002647725	0.071791294

ENSG00000139637	C12orf10	1.261692028	4.874677486	3.385091642	0.002503733	0.06965558
ENSG00000095203	EPB41L4B	-2.606662455	3.807635299	-3.335054641	0.002827688	0.074320368
ENSG00000183208	GDPGP1	-1.901471841	1.715748381	-3.30506827	0.003040971	0.077210153
ENSG00000007866	TEAD3	-1.266093951	5.871825837	-3.417240663	0.00231496	0.066649596
ENSG00000165105	RASEF	-2.634671777	2.585815259	-3.295171594	0.003114729	0.078072222
ENSG00000105835	NAMPT	-2.242156742	3.659550995	-3.321025939	0.002925597	0.075327415
ENSG00000104687	GSR	1.318118627	6.653908919	3.434945622	0.002216995	0.065904641
ENSG00000162444	RBP7	-2.023689537	2.910487774	-3.299146511	0.003084898	0.077696856
ENSG00000017373	SRCIN1	-2.227584831	3.355738711	-3.308873731	0.003013063	0.076625562
ENSG00000183421	RIPK4	-2.350412343	3.728494504	-3.319835118	0.002934058	0.075327415
ENSG00000154358	OBSCN	-2.530186041	6.419678281	-3.421609631	0.0022904	0.066647994
ENSG00000039139	DNAH5	-2.151985149	1.55346271	-3.292465972	0.003135194	0.078210368
ENSG00000117480	FAAH	-1.96109642	4.478640047	-3.345049774	0.00275988	0.073508893
ENSG00000175426	PCSK1	2.400305402	1.588623728	3.287547917	0.003172727	0.079021041
ENSG00000213214	ARHGEF35	-2.579885435	3.495414211	-3.293661204	0.003126137	0.078210368
ENSG00000204103	MAFB	2.258723604	2.394386075	3.272447755	0.003290709	0.080678934
ENSG00000064692	SNCAIP	2.253697817	2.376273714	3.273828426	0.003279747	0.080678934
ENSG00000050327	ARHGEF5	-2.207890839	2.973775092	-3.273714441	0.003280651	0.080678934
ENSG00000050820	BCAR1	1.089574512	6.228772588	3.398510735	0.002423183	0.068385199
ENSG00000005001	PRSS22	-2.710358108	2.28406791	-3.267207801	0.003332636	0.081199359
ENSG00000171291	ZNF439	-1.428198043	3.163610843	-3.273640908	0.003281234	0.080678934
ENSG00000171466	ZNF562	-1.348226948	4.769444457	-3.339786103	0.00279539	0.073842538
ENSG00000132749	MTL5	-1.768341171	2.921169631	-3.264469132	0.003354754	0.081280867
ENSG00000177700	POLR2L	1.119862046	6.719244441	3.402064039	0.00240228	0.068052664
ENSG00000215218	UBE2QL1	1.874341348	1.031910015	3.283028307	0.003207603	0.079511059
ENSG00000114812	VIPR1	-2.379054186	2.188202644	-3.254731503	0.003434556	0.082045418
ENSG00000107104	KANK1	-1.706078091	6.317463229	-3.386362634	0.002495994	0.06965558
ENSG00000105619	TFPT	1.240424892	5.463587445	3.360484921	0.002658259	0.071791294
ENSG00000214021	TTLL3	-1.978825679	3.747082757	-3.279964484	0.003231457	0.079976004
ENSG00000184012	TMPRSS2	-2.600746443	2.846746897	-3.254637551	0.003435335	0.082045418
ENSG00000137571	SLCO5A1	1.991653837	1.336248103	3.264228866	0.003356701	0.081280867
ENSG00000112182	BACH2	-1.659126335	1.276523507	-3.269016054	0.003318109	0.081199359
ENSG00000120708	TGFBI	2.255657663	9.465356837	3.426138861	0.002265209	0.066481509
ENSG00000074855	ANO8	-1.774594975	5.511393057	-3.357313808	0.002678835	0.071975353
ENSG00000114547	ROPN1B	-3.674937648	3.141839037	-3.261015308	0.003382851	0.081618943
ENSG00000172183	ISG20	-1.399515105	4.282195405	-3.297547635	0.003096864	0.077873226
ENSG00000117643	MAN1C1	2.097018398	3.46396291	3.2517773	0.003459128	0.082260618
ENSG00000129646	QRICH2	-2.458571947	5.097852409	-3.322379981	0.002916005	0.075327415
ENSG00000100601	ALKBH1	1.911496924	2.188656694	3.226678226	0.003674868	0.084642274
ENSG00000141447	OSBPL1A	-1.413932145	6.805577816	-3.374989879	0.002566083	0.070639319
ENSG00000099204	ABLIM1	-1.556284086	4.998330824	-3.317479458	0.002950865	0.075533481
ENSG00000082397	EPB41L3	1.715582317	5.312892343	3.331369429	0.002853096	0.074726846
ENSG00000088808	PPP1R13B	-1.167494063	5.121552553	-3.319702837	0.002934999	0.075327415
ENSG00000182578	CSF1R	2.771791578	6.755104314	3.370382225	0.002595022	0.070938128
ENSG00000159720	ATP6V0D1	0.995939852	6.725304236	3.365724509	0.002624597	0.071373575
ENSG00000158008	EXTL1	-4.212381646	3.939506608	-3.259715243	0.003393486	0.081618943
ENSG00000172296	SPTLC3	-2.06372146	3.90016467	-3.254686706	0.003434927	0.082045418
ENSG00000101400	SNTA1	1.246394992	4.838806173	3.299178977	0.003084656	0.077696856
ENSG00000042753	AP2S1	1.259847122	7.359825399	3.372738062	0.002580186	0.070719677
ENSG00000138764	CCNG2	-1.496433547	5.436219215	-3.321104581	0.002925039	0.075327415
ENSG00000145103	ILDR1	-1.920116935	1.375385028	-3.226219845	0.003678926	0.084642274
ENSG00000173889	PHC3	-1.43691466	5.538609427	-3.32503825	0.002897263	0.075142082
ENSG00000184949	FAM227A	-1.525081231	4.209052779	-3.260585321	0.003386365	0.081618943
ENSG00000141542	RAB40B	-1.602642433	4.331971158	-3.26545473	0.003346778	0.081280867
ENSG00000105467	SYNGR4	1.32435084	0.407137303	3.259326663	0.003396671	0.081618943
ENSG00000196132	MYT1	1.66962414	0.658282048	3.24698234	0.003499373	0.082445427
ENSG00000084623	EIF3I	1.052458125	8.65374211	3.376815334	0.002554705	0.070449707
ENSG00000081665	ZNF506	-1.137656461	6.339950968	-3.340350898	0.002791559	0.073842538
ENSG00000026508	CD44	-1.645297423	7.893081896	-3.367036597	0.002616233	0.071269629
ENSG00000241058	NSUN6	-1.558602976	3.642313015	-3.226318586	0.003678052	0.084642274
ENSG00000115109	EPB41L5	-1.78307718	3.594994975	-3.222191523	0.003714781	0.085342056
ENSG00000183801	OLFML1	1.898374068	2.009596838	3.194891272	0.003966827	0.088792407
ENSG00000137962	ARHGAP29	-2.202729471	5.631637085	-3.31199157	0.002990383	0.076296103
ENSG00000130052	STARD8	1.881505142	3.113439778	3.203271129	0.003887753	0.087900198
ENSG00000135750	KCNK1	-2.361719379	2.061039216	-3.190084942	0.004012879	0.089211585

ENSG00000106714	CNTNAP3	-2.349953273	3.061600841	-3.19976675	0.003920634	0.088135628
ENSG00000129295	LRRC6	-2.222310802	2.624301717	-3.189970511	0.004013981	0.089211585
ENSG00000196177	ACADSB	-2.031610698	2.934169504	-3.195658513	0.003959523	0.088755531
ENSG00000163617	KIAA1407	-1.869276368	3.614073543	-3.21268397	0.003800749	0.086369001
ENSG00000013619	MAMLD1	-2.052842483	2.090857515	-3.185536467	0.004056935	0.089911535
ENSG00000147459	DOCK5	-1.370777841	5.975670495	-3.313674874	0.002978208	0.076109209
ENSG00000110900	TSPAN11	2.15056969	2.196803013	3.179367221	0.004117437	0.090340082
ENSG00000205002	AARD	-3.856125644	3.83853757	-3.216552928	0.003765536	0.086106116
ENSG00000159450	TCHH	1.570777818	0.588610015	3.228769922	0.003656402	0.084620364
ENSG00000173041	ZNF680	-1.899428274	1.946532221	-3.178978231	0.004121281	0.090340082
ENSG00000008394	MGST1	-2.225315256	4.110039318	-3.232606879	0.003622763	0.084159097
ENSG00000182544	MFSD5	1.568555028	1.153002009	3.201338629	0.003905852	0.088015072
ENSG00000204634	TBC1D8	-1.404139304	5.838144787	-3.301720847	0.003065727	0.077587615
ENSG00000119431	HDHD3	-1.477137354	3.903131572	-3.215457132	0.003775477	0.086106116
ENSG00000237441	RGL2	-1.137674205	7.381627951	-3.338022609	0.002807386	0.074034784
ENSG00000175874	CREG2	1.567069244	0.768544937	3.215747654	0.003772839	0.086106116
ENSG00000175906	ARL4D	1.818151827	0.80746163	3.214824922	0.003781224	0.086112023
ENSG00000181885	CLDN7	-2.359542308	4.392987744	-3.240610756	0.003553552	0.083531007
ENSG00000073331	ALPK1	-1.464057644	4.812282916	-3.257673919	0.003410251	0.08181994
ENSG00000145029	NICN1	-1.450522271	4.306186312	-3.232722224	0.003621756	0.084159097
ENSG00000075826	SEC31B	-1.898750028	3.87408637	-3.210961397	0.003816529	0.086414364
ENSG00000115756	HPCAL1	1.699391887	5.029454178	3.267670194	0.003328915	0.081199359
ENSG00000161551	ZNF577	-1.69045918	4.262574133	-3.226357728	0.003677705	0.084642274
ENSG00000163359	COL6A3	1.914141941	9.952902805	3.357976429	0.002674523	0.071975353
ENSG00000182158	CREB3L2	-1.433914402	6.054877618	-3.300364365	0.003075814	0.077696856
ENSG00000082684	SEMA5B	-2.273626899	2.76670756	-3.171907438	0.00419176	0.090972197
ENSG00000125637	PSD4	-1.319311736	4.756238134	-3.250338374	0.003471158	0.082274823
ENSG00000189319	FAM53B	-1.673456865	1.562754025	-3.178796843	0.004123075	0.090340082
ENSG00000130396	MLLT4	-1.113567403	7.144381768	-3.325303881	0.002895397	0.075142082
ENSG00000126878	AIF1L	-2.967387063	4.18426961	-3.21796839	0.003752732	0.085962215
ENSG00000157423	HYDIN	-2.155724165	1.696577969	-3.17577001	0.004153116	0.090340082
ENSG00000072682	P4HA2	1.611725263	7.719893092	3.330684368	0.002857843	0.074726846
ENSG00000177025	C19orf18	-1.955373641	1.484768743	-3.178186453	0.004129116	0.090340082
ENSG00000147174	ACRC	-1.649648052	2.311966574	-3.159197582	0.00432138	0.093013417
ENSG00000160691	SHC1	1.238722032	7.989450409	3.326816624	0.00288479	0.075066743
ENSG00000147162	OGT	-1.215586273	8.445497886	-3.330390077	0.002859885	0.074726846
ENSG00000121413	ZSCAN18	-2.002009147	4.872597904	-3.240313831	0.003556097	0.083531007
ENSG00000175106	TVP23C	-1.547317521	1.476052191	-3.162442573	0.004287924	0.092547195
ENSG00000169247	SH3TC2	-2.083914491	1.847717887	-3.152515663	0.004391068	0.093741828
ENSG00000104879	CKM	1.534639251	0.708164219	3.191228793	0.004001872	0.089195136
ENSG00000157368	IL34	-3.115064293	3.704320233	-3.179364913	0.00411746	0.090340082
ENSG00000154589	LY96	1.815636328	3.223251157	3.157170566	0.004342407	0.093190796
ENSG00000198915	RASGEF1A	-2.929378244	3.796970043	-3.176111819	0.004149713	0.090340082
ENSG00000125354	SEPT6	1.432741232	5.702200321	3.265524562	0.003346213	0.081280867
ENSG00000054654	SYNE2	-1.337572945	7.271552722	-3.302073084	0.003063113	0.077587615
ENSG00000183072	NKX2-5	1.905616311	1.055889896	3.166767028	0.004243726	0.091845927
ENSG00000154096	THY1	1.980236421	6.580423802	3.286159586	0.003183401	0.079161238
ENSG00000079691	LRRC16A	-1.490787162	5.322273932	-3.247103632	0.003498349	0.082445427
ENSG00000067606	PRKCZ	-2.053805793	4.672943457	-3.212199591	0.00380518	0.086369001
ENSG00000126822	PLEKHG3	-1.507479786	5.520169704	-3.251036528	0.003465316	0.082260618
ENSG00000102181	CD99L2	1.42714334	5.556719186	3.248582977	0.003485888	0.082445427
ENSG00000138101	DTNB	-1.320592981	5.980781125	-3.261404242	0.003379676	0.081618943
ENSG00000204514	ZNF814	-1.039088511	6.651811106	-3.278276195	0.003244674	0.080176669
ENSG00000162384	C1orf123	0.890928633	6.412004967	3.27291618	0.003286986	0.080678934
ENSG00000117122	MFAP2	1.655984442	7.502451109	3.293099263	0.003130392	0.078210368
ENSG00000112624	GLTSCR1L	-1.893180007	3.457050129	-3.146428995	0.004455489	0.094346937
ENSG00000149292	TTC12	-1.644847232	4.158059918	-3.176824129	0.00414263	0.090340082
ENSG00000154856	APCDD1	2.096661807	4.573892863	3.197935659	0.003937921	0.088397604
ENSG00000132481	TRIM47	-1.380427205	5.521669564	-3.238124879	0.00357491	0.083847396
ENSG00000116871	MAP7D1	1.467553212	8.232058119	3.295492781	0.003112308	0.078072222
ENSG00000125398	SOX9	-1.464013782	5.085935038	-3.21964922	0.003737582	0.085740361
ENSG00000167566	NCKAP5L	1.382206759	4.968461323	3.211780101	0.003809021	0.086369001
ENSG00000088538	DOCK3	-2.955879128	2.810320303	-3.114757384	0.004805676	0.098828138
ENSG00000091409	ITGA6	-1.616566995	6.928068522	-3.267619907	0.00332932	0.081199359
ENSG00000132356	PRKAA1	-1.034098717	5.608944742	-3.231990929	0.003628143	0.084159097

ENSG00000174586	ZNF497	-1.516384883	1.556222772	-3.120215496	0.004743493	0.098192813
ENSG00000033170	FUT8	1.143881911	4.646314741	3.180971454	0.004101621	0.090340082
ENSG00000154016	GRAP	1.617755124	1.706564899	3.105807374	0.004909344	0.10004223
ENSG00000142227	EMP3	1.533383102	6.442898213	3.247904189	0.0034916	0.082445427
ENSG00000188322	SBK1	-2.266623685	2.412234839	-3.098023391	0.005001252	0.101388439
ENSG00000100097	LGALS1	1.223349763	9.053656457	3.284282018	0.003197892	0.079395754
ENSG00000132554	RGS22	-1.74375795	1.31160928	-3.115677766	0.004795136	0.098740786
ENSG00000178860	MSC	1.771149297	0.960004579	3.127086885	0.00466631	0.097107518
ENSG00000165078	CPA6	1.707022533	0.491876867	3.149069433	0.004427432	0.094133915
ENSG00000186814	ZSCAN30	-1.683827075	1.91659213	-3.094080547	0.005048436	0.10178345
ENSG00000158270	COLEC12	1.624904148	5.259624633	3.200932973	0.003909662	0.088015072
ENSG00000105514	RAB3D	-1.466106964	4.3694975	-3.156450324	0.004349902	0.093190796
ENSG00000145147	SLIT2	1.886695518	4.222837838	3.149793316	0.004419769	0.094098508
ENSG00000105499	PLA2G4C	-2.101487726	4.180384096	-3.144528679	0.004475788	0.094521657
ENSG00000198408	MGEA5	-1.154596967	7.093615681	-3.252318046	0.003454617	0.082260618
ENSG00000121350	PYROXD1	-1.891824282	1.697273583	-3.093938201	0.005050148	0.10178345
ENSG00000171714	ANO5	2.259299327	1.161718561	3.107611556	0.004888275	0.099742414
ENSG00000132199	ENOSF1	-1.199726342	6.521825446	-3.233145343	0.003618066	0.084159097
ENSG00000157106	SMG1	-1.127830809	6.801131116	-3.23546288	0.003597918	0.08413552
ENSG00000156253	RWDD2B	-1.55038647	3.815010325	-3.112173032	0.004835393	0.099179273
ENSG00000167779	IGFBP6	1.769308133	4.209977583	3.131842657	0.0046136	0.096522668
ENSG00000085465	OVGP1	-2.036819792	2.243332044	-3.072878185	0.005309595	0.104957557
ENSG00000196923	PDLIM7	1.176319064	8.046919151	3.251490934	0.003461519	0.082260618
ENSG00000087495	PHACTR3	1.88393218	0.693732901	3.119049029	0.004756717	0.098207432
ENSG00000161681	SHANK1	2.68668215	2.098041254	3.069872971	0.005347647	0.105219238
ENSG00000129450	SIGLEC9	2.121100774	2.445536147	3.068876831	0.005360317	0.105238679
ENSG00000182199	SHMT2	0.953973794	7.047685827	3.231651857	0.003631108	0.084159097
ENSG00000155659	VSIG4	2.884553701	3.818924052	3.110457989	0.00485521	0.099383804
ENSG00000167701	GPT	-2.691671054	2.655171134	-3.069597029	0.005351154	0.105219238
ENSG00000081087	OSTM1	1.342879911	4.668853959	3.144804523	0.004472836	0.094521657
ENSG00000164142	FAM160A1	-1.781557856	2.951351891	-3.067982186	0.005371721	0.105238679
ENSG00000151006	PRSS53	-1.622222239	1.486550738	-3.074354194	0.005291002	0.104824638
ENSG00000152661	GJA1	1.939483033	3.253324214	3.081177591	0.005205857	0.104137256
ENSG00000215193	PEX26	-1.051716066	5.838159362	-3.192175223	0.003992787	0.089119242
ENSG00000183386	FHL3	1.497996965	4.887705772	3.152788251	0.004388204	0.093741828
ENSG00000227051	C14orf132	1.923029447	1.194288478	3.080319976	0.005216486	0.104137256
ENSG00000141522	ARHGDIA	1.043544331	8.459950059	3.237325734	0.003581802	0.083883663
ENSG00000131773	KHDRBS3	-1.682959482	5.661175757	-3.177645621	0.004134476	0.090340082
ENSG00000174514	MFSD4	-2.421457595	3.111653338	-3.063023446	0.005435355	0.106209408
ENSG00000164181	ELOVL7	-2.092499307	2.306178121	-3.047439895	0.005640079	0.108587092
ENSG00000100632	ERH	1.1898433	5.471180982	3.170245782	0.00420849	0.091209143
ENSG00000077942	FBLN1	1.874515188	8.471134748	3.232912354	0.003620098	0.084159097
ENSG00000108021	FAM208B	-1.364166334	6.111733456	-3.188854571	0.00402475	0.089324404
ENSG00000125814	NAPB	-1.226233022	3.821112997	-3.083665626	0.00517514	0.103840301
ENSG00000169116	PARM1	1.514970534	2.742627417	3.046538902	0.005652139	0.108685925
ENSG00000131089	ARHGEF9	-1.477058879	6.069000656	-3.183697239	0.004074882	0.090181909
ENSG00000187957	DNER	-4.360407837	3.39789288	-3.070499222	0.005339696	0.105219238
ENSG00000156374	PCGF6	-1.479604127	1.86517217	-3.042950549	0.005700417	0.109480094
ENSG00000196150	ZNF250	-1.37342305	4.830863176	-3.127656272	0.004659969	0.097104343
ENSG00000213694	S1PR3	1.941178318	2.445266662	3.033786278	0.005825514	0.110062823
ENSG00000162511	LAPTM5	2.163829288	7.153903064	3.201849552	0.003901059	0.088015072
ENSG00000166337	TAF10	1.131089412	6.693056068	3.192576494	0.003988942	0.089119242
ENSG00000106511	MEOX2	1.743644342	1.280094074	3.054741973	0.005543245	0.107117068
ENSG00000105383	CD33	1.835502004	2.6337501	3.032664766	0.005841003	0.110062823
ENSG00000112964	GHR	1.731217115	2.3817314	3.029436941	0.005885803	0.110167276
ENSG00000145451	GLRA3	1.529772043	0.601323746	3.076874266	0.005259401	0.104462356
ENSG00000137491	SLCO2B1	2.062796322	5.262047889	3.136520681	0.004562312	0.095961447
ENSG00000253313	C1orf210	-1.984198016	1.492748732	-3.035204409	0.005805985	0.110062823
ENSG00000165215	CLDN3	-1.841781943	1.345668882	-3.039672758	0.005744861	0.109796126
ENSG00000243156	MICAL3	-1.131655682	5.945119428	-3.159485355	0.004318403	0.093013417
ENSG00000178695	KCTD12	2.056604633	3.759583076	3.057009455	0.005513502	0.106937402
ENSG00000188487	INSC	2.787986984	1.596616752	3.024817024	0.005950497	0.110235236
ENSG00000178038	ALS2CL	-2.421647131	5.575171848	-3.140259299	0.004521719	0.095363291
ENSG00000152894	PTPRK	-1.701273939	7.014646334	-3.17979966	0.004113168	0.090340082
ENSG00000198930	CSAG1	1.523132542	0.457019473	3.071360881	0.005328775	0.105174592

ENSG00000102174	PHEX	2.347677106	2.335702701	3.010053803	0.006161832	0.11190429
ENSG00000154553	PDLIM3	-2.095169644	5.343214314	-3.123520232	0.00470622	0.097678973
ENSG00000076685	NT5C2	-1.021654327	6.411891794	-3.162548796	0.004286833	0.092547195
ENSG00000119714	GPR68	2.058472796	1.416878705	3.02488337	0.005949563	0.110235236
ENSG00000171222	SCAND1	1.537881415	4.358908255	3.07761041	0.005250204	0.104411856
ENSG00000135373	EHF	-3.627964525	3.253279891	-3.040485096	0.005733816	0.109718661
ENSG00000021300	PLEKHB1	-2.72533212	3.350722377	-3.0260904	0.005932598	0.110235236
ENSG00000198732	SMOC1	-3.109897045	4.362869389	-3.078582069	0.005238089	0.10430311
ENSG00000143452	HORMAD1	-2.516809239	3.24135245	-3.021862205	0.005992231	0.110486599
ENSG00000121380	BCL2L14	-1.54509577	1.126680892	-3.032014433	0.005850003	0.110062823
ENSG00000143184	XCL1	1.717879066	1.041093498	3.033352771	0.005831497	0.110062823
ENSG00000102886	GDPD3	-1.730902281	3.378811814	-3.022330323	0.005985601	0.110486599
ENSG00000121900	TMEM54	-1.501634881	5.410774377	-3.120519839	0.004740049	0.098192813
ENSG00000161638	ITGA5	1.864521862	7.472409207	3.175392696	0.004156876	0.090340082
ENSG00000131711	MAP1B	1.381270776	5.693994128	3.131049247	0.004622354	0.096577035
ENSG00000181555	SETD2	-0.897181502	6.559594631	-3.156116717	0.004353378	0.093190796
ENSG00000102302	FGD1	1.446978429	5.768057105	3.133224872	0.004598389	0.096461652
ENSG00000085719	CPNE3	-1.323925347	6.262254404	-3.14713926	0.004447925	0.094346937
ENSG00000164434	FABP7	-3.637026879	2.919636194	-3.004570474	0.006242142	0.112739238
ENSG00000174876	AMY1B	-2.096501499	2.173392694	-2.991562828	0.006436686	0.114676509
ENSG00000218336	TENM3	2.833531088	2.770451173	2.996722718	0.006358829	0.113899988
ENSG00000142583	SLC2A5	1.695407738	3.604947488	3.024944418	0.005948704	0.110235236
ENSG00000075223	SEMA3C	-3.035908731	4.623546949	-3.079181145	0.005230632	0.104286979
ENSG00000135723	FHOD1	1.241979055	6.0958373	3.13939469	0.004531075	0.095432356
ENSG00000124216	SNAI1	1.935240859	1.540159717	3.002931097	0.006266347	0.112758321
ENSG00000196275	GTF2IRD2	-1.111165021	6.094431471	-3.134673703	0.004582496	0.096256949
ENSG00000145545	SRD5A1	-1.707396611	2.067069712	-2.987086897	0.00650496	0.11468464
ENSG00000124466	LYPD3	-2.72907113	2.885693798	-2.9939521	0.006400522	0.114352082
ENSG00000136068	FLNB	-1.264080311	8.691483601	-3.177597187	0.004134956	0.090340082
ENSG00000103742	IGDCC4	1.869125542	3.704670306	3.018838622	0.006035227	0.110888457
ENSG00000133460	SLC2A11	-1.782223472	3.597894262	-3.01309404	0.006117731	0.111640319
ENSG00000182685	BRICD5	-1.634239879	3.254922885	-2.999501857	0.00631727	0.113544424
ENSG00000115221	ITGB6	-2.817512842	2.534879295	-2.990155715	0.006458075	0.114676509
ENSG00000101443	WFDC2	-3.335195133	3.640149448	-3.032265601	0.005846525	0.110062823
ENSG00000006625	GGCT	-1.182396706	5.247555293	-3.095177155	0.00503527	0.10178345
ENSG00000140443	IGF1R	-1.872633772	5.912658987	-3.118390235	0.004764201	0.098232698
ENSG00000102935	ZNF423	1.924124828	2.58925781	2.977886178	0.006647488	0.116282871
ENSG00000171522	PTGER4	1.743583334	1.596936977	2.988772159	0.006479173	0.114676509
ENSG00000259518	RP11-597K23.2	1.300401694	0.378582783	3.041004274	0.005726767	0.109717581
ENSG00000204149	AGAP6	-1.33373956	4.363457759	-3.042371317	0.005708247	0.10949645
ENSG00000161798	AQP5	-3.361690091	3.000634547	-2.983371818	0.006562155	0.115058952
ENSG00000116649	SRM	1.447944455	7.592031635	3.151192659	0.004404994	0.093911351
ENSG00000088280	ASAP3	-1.431744922	6.581876139	-3.129657972	0.004637742	0.096769697
ENSG00000081181	ARG2	1.939388428	1.29591006	2.990776503	0.00644863	0.114676509
ENSG00000146802	TMEM168	-1.503919747	4.035862124	-3.017094456	0.006060163	0.111095686
ENSG00000010327	STAB1	1.725205954	8.655645839	3.156755498	0.004346725	0.093190796
ENSG00000135049	AGTPBP1	-1.235899035	4.90884125	-3.061546935	0.005454442	0.106276326
ENSG00000042980	ADAM28	1.977395008	4.284989075	3.029088386	0.00589066	0.110167276
ENSG00000166405	RIC3	-3.368466481	3.144086695	-2.979451823	0.006623026	0.115984258
ENSG00000123572	NRK	2.328935619	1.404422246	2.977055396	0.006660504	0.116380807
ENSG00000197969	VPS13A	-1.642042394	4.480310153	-3.034976391	0.005809121	0.110062823
ENSG00000150938	CRIM1	-1.901642991	5.670982435	-3.089678188	0.005101625	0.102496289
ENSG00000064547	LPAR2	-1.752750749	3.208967805	-2.970790592	0.006759441	0.117861932
ENSG00000196739	COL27A1	-1.735256455	8.742391769	-3.146537139	0.004454337	0.094346937
ENSG00000169413	RNASE6	1.703837346	1.070267468	2.983326506	0.006562855	0.115058952
ENSG00000137693	YAP1	-1.533106126	4.530889153	-3.032403197	0.005844621	0.110062823
ENSG00000204956	PCDHGA1	1.272679162	0.378582783	3.017059379	0.006060666	0.111095686
ENSG00000162782	TDRD5	-2.307698954	2.181657839	-2.947509887	0.007139556	0.121907389
ENSG00000160014	CALM3	1.037654535	7.664481756	3.131993508	0.004611938	0.096522668
ENSG00000003137	CYP26B1	-2.301192318	3.883393449	-2.993593824	0.006405932	0.114352082
ENSG00000144857	BOC	-2.461443504	6.876689321	-3.113842538	0.004816176	0.098914413
ENSG00000151773	CCDC122	-1.673472316	2.711004378	-2.950931187	0.007082442	0.121587091
ENSG00000165272	AQP3	-2.898914614	4.262358513	-3.011361085	0.006142832	0.111689655
ENSG00000133134	BEX2	-2.137604479	2.701281523	-2.94818359	0.007128275	0.121907389
ENSG00000115548	KDM3A	-1.078703406	6.446508911	-3.103902827	0.00493168	0.100237043

ENSG00000141526	SLC16A3	1.719258441	5.522558087	3.07502025	0.005282632	0.104791127
ENSG00000117155	SSX2IP	-1.380342885	4.671886699	-3.032291833	0.005846162	0.110062823
ENSG00000181350	FAM211A	1.376613792	0.608399877	2.998620838	0.006330416	0.113650531
ENSG00000160282	FTCD	-2.019903591	1.823435204	-2.950221556	0.007094253	0.12165674
ENSG00000152454	ZNF256	-1.785305074	1.797456751	-2.944797159	0.007185154	0.122412871
ENSG00000186399	GOLGA8R	-1.817723541	1.863209899	-2.941650546	0.007238394	0.12278663
ENSG00000126460	PRRG2	-1.996321026	1.476747805	-2.952451578	0.007057202	0.121286474
ENSG00000131368	MRPS25	-1.261948715	4.468459287	-3.01301755	0.006118837	0.111640319
ENSG00000109472	CPE	2.353624925	7.581672175	3.119438581	0.004752297	0.098207432
ENSG00000198373	WWP2	-1.602737789	8.048438417	-3.124651514	0.004693526	0.097544524
ENSG00000161835	GRASP	1.668057122	3.722382851	2.970737086	0.006760292	0.117861932
ENSG00000177963	RIC8A	0.914529556	6.556791878	3.095872695	0.005026937	0.101777635
ENSG00000148459	PDSS1	-1.475180907	3.032752403	-2.94001359	0.007266239	0.123125869
ENSG00000127980	PEX1	-1.326694863	4.863480915	-3.027927286	0.005906868	0.110235236
ENSG00000130203	APOE	1.900503681	7.202280054	3.104844307	0.004920627	0.100142091
ENSG00000173511	VEGFB	1.148692626	6.976679945	3.099963825	0.004978187	0.101051406
ENSG00000106034	CPED1	2.087111947	3.02834764	2.936576857	0.007325032	0.123721294
ENSG00000113194	FAF2	1.085020315	4.962389753	3.030674821	0.005868583	0.11014825
ENSG00000244486	SCARF2	1.60053098	4.67143967	3.014720942	0.006094256	0.111451023
ENSG00000076864	RAP1GAP	-2.148456217	4.558363817	-3.005828568	0.006223628	0.112739238
ENSG00000147862	NFIB	-2.338506015	6.733659326	-3.090905552	0.005086742	0.102328301
ENSG00000108179	PPIF	1.263964347	5.825811947	3.066088708	0.005395935	0.105570588
ENSG00000124155	PIGT	0.974665975	8.010961824	3.110152644	0.004858747	0.099383804
ENSG00000150764	DIXDC1	-1.637591203	4.361629317	-2.987261213	0.006502288	0.11468464
ENSG00000105479	CCDC114	-1.9025855	1.418116494	-2.9371689	0.007314872	0.123721294
ENSG00000160801	PTH1R	2.894395798	3.963387607	2.966617299	0.006826126	0.11848312
ENSG00000168646	AXIN2	1.930437396	2.440968812	2.917595419	0.007658021	0.127155566
ENSG00000114353	GNAI2	1.136793846	8.301108968	3.109667967	0.004864366	0.099383804
ENSG00000168734	PKIG	1.171663191	4.355050177	2.988071499	0.006489882	0.114676509
ENSG00000151532	VTI1A	-1.608596586	3.762878549	-2.955089545	0.007013611	0.121036113
ENSG00000002834	LASP1	1.130086795	6.312159187	3.072759824	0.005311089	0.104957557
ENSG00000139190	VAMP1	-1.573387791	2.102155485	-2.914151046	0.007719973	0.127509573
ENSG00000113916	BCL6	-1.684395149	5.942003876	-3.055298395	0.005535932	0.107117068
ENSG00000173868	PHOSPHO1	2.664100389	1.385052506	2.929785743	0.007442548	0.124899492
ENSG00000147799	ARHGAP39	-2.048817435	4.973662562	-3.006985401	0.00620665	0.112587913
ENSG00000197372	ZNF675	-1.410504405	3.501693474	-2.931103245	0.00741961	0.124781458
ENSG00000180776	ZDHHC20	-2.089349783	3.127147117	-2.916519029	0.00767733	0.12720696
ENSG00000101955	SRPX	1.921793498	5.738057986	3.036081916	0.005793932	0.110062823
ENSG00000040731	CDH10	2.114063227	1.307542039	2.917875529	0.007653004	0.127155566
ENSG00000167702	KIFC2	-2.788569982	6.636429086	-3.060821002	0.00546385	0.106276326
ENSG00000002586	CD99	1.482392196	8.614795966	3.093686856	0.005053171	0.10178345
ENSG00000146112	PPP1R18	1.253333927	5.014974596	3.003947713	0.006251327	0.112739238
ENSG00000167693	NXN	1.437375725	6.388606907	3.054981519	0.005540096	0.107117068
ENSG00000134324	LPIN1	-1.509640204	6.165384192	-3.047473959	0.005639624	0.108587092
ENSG00000177830	CHID1	0.866110109	7.85175999	3.080341625	0.005216218	0.104137256
ENSG00000103449	SALL1	1.501999878	0.604225376	2.948082854	0.007129961	0.121907389
ENSG00000125970	RALY	0.879636801	8.167596033	3.08248697	0.00518967	0.10399886
ENSG00000155111	CDK19	-1.379403756	4.213174522	-2.952912824	0.007049561	0.121286474
ENSG00000149043	SYT8	-3.500301187	3.678786661	-2.926160124	0.007506022	0.125561822
ENSG00000113971	NPHP3	-1.337070796	5.729171342	-3.026945181	0.005920611	0.110235236
ENSG00000136235	GPNMB	1.690743176	7.318836204	3.067939727	0.005372263	0.105238679
ENSG00000187650	VMAC	-1.646418578	2.699030813	-2.886183883	0.00824099	0.132625002
ENSG00000142507	PSMB6	0.850902518	6.750250186	3.053849099	0.005554999	0.107212172
ENSG00000115507	OTX1	-1.815538528	1.629665997	-2.893840581	0.008095112	0.131627361
ENSG00000162723	SLAMF9	1.496645335	0.72519402	2.930392449	0.007431977	0.12485562
ENSG00000101639	CEP192	-1.049726723	6.087436669	-3.033127054	0.005834614	0.110062823
ENSG00000050555	LAMC3	2.290642254	4.085539923	2.936911604	0.007319286	0.123721294
ENSG00000198838	RYR3	-2.432547184	2.651376888	-2.879339152	0.008373514	0.134366413
ENSG00000147614	ATP6V0D2	1.950674577	1.640287894	2.890939771	0.008150088	0.132247181
ENSG00000116962	NID1	1.806519942	7.41932239	3.061537632	0.005454562	0.106276326
ENSG00000186567	CEACAM19	-1.53798075	3.963751677	-2.927982519	0.007474053	0.125160475
ENSG00000239998	LILRA2	1.750367036	1.153002009	2.904047737	0.007904463	0.130009363
ENSG00000102098	SCML2	-1.524026359	2.374627882	-2.872273406	0.008512444	0.135465269
ENSG00000198089	SFI1	-1.202427002	6.548875543	-3.038502102	0.005760815	0.109967093
ENSG00000102879	CORO1A	1.851615644	6.004698849	3.023965814	0.005962491	0.110327171

ENSG00000136280	CCM2	0.864556175	6.293577938	3.031053393	0.005863327	0.11014825
ENSG00000080573	COL5A3	1.974795675	7.931688022	3.060665119	0.005465872	0.106276326
ENSG00000221994	ZNF630	-1.431339066	2.036146334	-2.867111	0.008615333	0.136548671
ENSG00000204618	RNF39	-1.340065241	1.009886749	-2.903001816	0.0079238	0.130054761
ENSG00000188883	KLRG2	-2.464505689	2.085532221	-2.864065442	0.008676585	0.136691066
ENSG00000204323	SMIM5	-1.413018004	1.103119838	-2.900594278	0.007968483	0.130379002
ENSG00000182983	ZNF662	-1.904000197	1.960709168	-2.864602872	0.008665747	0.136691066
ENSG00000130528	HRC	1.805040578	0.75024848	2.914307747	0.007717144	0.127509573
ENSG00000131446	MGAT1	1.268749579	5.322529184	2.988484781	0.006483563	0.114676509
ENSG00000215045	GRID2IP	-1.915343524	2.031743453	-2.864878173	0.008660199	0.136691066
ENSG00000136630	HLX	1.516312422	3.101209653	2.87592327	0.008440407	0.134925654
ENSG00000142694	EVA1B	1.681684646	2.165046864	2.858942998	0.008780544	0.137193335
ENSG00000139597	N4BP2L1	-1.434150179	3.564958665	-2.895397145	0.008065758	0.131422442
ENSG00000243364	EFNA4	-1.578322466	3.46837822	-2.88753112	0.008215142	0.132444654
ENSG00000166863	TAC3	2.848523229	1.157472147	2.888850289	0.008189907	0.132354143
ENSG00000126870	WDR60	-0.985578014	6.166103172	-3.015144278	0.006088162	0.111451023
ENSG00000121753	BAI2	1.575967012	5.572692661	2.995070221	0.006383665	0.114214462
ENSG00000120756	PLS1	-2.023955439	2.123538127	-2.855585723	0.00884932	0.137616139
ENSG00000035664	DAPK2	-1.332572431	4.782908429	-2.954732703	0.007019493	0.121036113
ENSG00000197070	ARRDC1	-1.419860249	4.801423127	-2.953998666	0.007031606	0.121111888
ENSG00000077549	CAPZB	0.967324327	8.836824071	3.057695239	0.005504536	0.106895646
ENSG00000173918	C1QTNF1	1.90746018	4.050224751	2.911692533	0.007764485	0.128109914
ENSG00000164932	CTHRC1	2.293515523	6.788939922	3.022645473	0.005981141	0.110486599
ENSG00000147488	ST18	1.463795737	0.780069656	2.897275343	0.008030474	0.131065279
ENSG00000142700	DMRTA2	1.13088181	0.335231866	2.917711941	0.007655934	0.127155566
ENSG00000100364	KIAA0930	1.240302309	6.832968468	3.020581832	0.006010402	0.110561804
ENSG00000160255	ITGB2	1.767565325	7.189783262	3.025590266	0.005939622	0.110235236
ENSG00000150625	GPM6A	1.943612611	1.022482326	2.878993899	0.008380252	0.134366413
ENSG00000163141	BNIPL	-1.940147439	4.097627888	-2.901241843	0.007956441	0.130379002
ENSG00000067177	PHKA1	-1.347678627	4.82987119	-2.943209364	0.007211972	0.122603526
ENSG00000198182	ZNF607	-1.647430091	1.999815077	-2.841915274	0.009134692	0.140936535
ENSG00000186469	GNG2	1.725807849	2.552676698	2.841157717	0.009150759	0.141045741
ENSG00000138801	PAPSS1	-1.162392677	7.950737267	-3.034860353	0.005810717	0.110062823
ENSG00000139174	PRICKLE1	-2.055675376	3.250680025	-2.858619039	0.008787158	0.137193335
ENSG00000163462	TRIM46	-1.803321581	3.190836042	-2.85719627	0.008816263	0.137388984
ENSG00000179832	MROH1	-1.320795378	6.361927441	-3.003488874	0.006258102	0.112739238
ENSG00000158246	FAM46B	-1.803133349	1.392698199	-2.862818186	0.00870179	0.136781937
ENSG00000171962	LRRC48	-1.644888856	2.467089303	-2.834491678	0.009293301	0.141763776
ENSG00000103037	SETD6	-1.208408731	4.194644213	-2.900986209	0.007961192	0.130379002
ENSG00000100985	MMP9	3.323454132	7.656131586	3.025550227	0.005940184	0.110235236
ENSG00000106991	ENG	1.545082626	6.954404522	3.011966089	0.006134058	0.111689655
ENSG00000112280	COL9A1	-4.867345968	3.672248479	-2.875161081	0.008455402	0.134968174
ENSG00000105717	PBX4	-1.672065541	2.566678107	-2.831093485	0.009366775	0.142416728
ENSG00000228146	CASP16	-1.626387857	1.168494021	-2.860789807	0.008742928	0.136911457
ENSG00000173801	JUP	-1.543332554	7.659360659	-3.02124258	0.006001018	0.11051875
ENSG00000124145	SDC4	-1.509384229	7.145448733	-3.011356702	0.006142895	0.111689655
ENSG00000150593	PDCD4	-1.246961989	6.850178846	-3.003541667	0.006257322	0.112739238
ENSG00000161664	ASB16	-1.383977385	1.971467765	-2.825980768	0.009478358	0.142867357
ENSG00000198018	ENTPD7	1.840863992	3.263695649	2.844755056	0.009074701	0.14014876
ENSG00000108798	ABI3	1.69239901	3.60699825	2.860033594	0.008758312	0.137015625
ENSG00000111653	ING4	-1.187735668	6.358931874	-2.988379162	0.006485177	0.114676509
ENSG00000169084	DHRSX	1.297439665	4.64376687	2.916041412	0.007685913	0.127214834
ENSG00000102218	RP2	1.361295417	3.513068236	2.853393574	0.008894504	0.138076029
ENSG00000144791	LIMD1	-1.087401439	5.28692983	-2.946082848	0.007163509	0.122176755
ENSG00000120149	MSX2	1.788520288	0.769363901	2.86899853	0.008577577	0.136274304
ENSG00000087245	MMP2	1.422765843	9.19809306	3.029452358	0.005885588	0.110167276
ENSG00000149357	LAMTOR1	0.961344249	6.367247536	2.985599099	0.006527808	0.114958283
ENSG00000108602	ALDH3A1	-1.578169383	1.47175434	-2.835138179	0.009279385	0.141763776
ENSG00000180353	HCLS1	1.677106371	6.366530035	2.984627734	0.006542766	0.114963651
ENSG00000159200	RCAN1	-1.541668971	4.857036359	-2.921587325	0.007586814	0.126495746
ENSG00000163499	CRYBA2	2.148635544	1.469550148	2.835248725	0.009277008	0.141763776
ENSG00000151692	RNF144A	1.843260554	3.623041065	2.852871733	0.008905292	0.138076029
ENSG00000154511	FAM69A	1.704944862	2.168432489	2.815243182	0.009716822	0.144930286
ENSG00000171700	RGS19	1.429274982	4.314608588	2.887207547	0.008221343	0.132444654
ENSG00000197381	ADARB1	-1.52799644	5.43948472	-2.944192594	0.007195354	0.122453688

ENSG00000051523	CYBA	1.71495208	7.673994876	3.003997259	0.006250596	0.112739238
ENSG00000110328	GALNT18	-1.685395686	4.972373708	-2.916748094	0.007673217	0.12720696
ENSG00000188783	PRELP	-2.795508207	4.821022406	-2.904585772	0.007894533	0.129982292
ENSG00000144283	PKP4	-0.881057966	5.601988798	-2.947486642	0.007139946	0.121907389
ENSG00000204019	CT83	-1.835855574	1.435146295	-2.828757918	0.009417592	0.142686548
ENSG00000137573	SULF1	2.052897807	6.995103177	2.984931686	0.006538082	0.114963651
ENSG00000134762	DSC3	-3.116944523	5.153184104	-2.923671473	0.007549889	0.12616114
ENSG00000153012	LGI2	1.734925558	1.7235947	2.809322789	0.009850727	0.145956336
ENSG00000119630	PGF	1.557656838	4.802391704	2.903275692	0.007918733	0.130054761
ENSG00000064666	CNN2	0.965923024	6.378803432	2.96832046	0.006798835	0.118271093
ENSG00000162664	ZNF326	-1.31923093	5.423571818	-2.933027399	0.007386231	0.124486946
ENSG00000115423	DNAH6	-1.690575612	1.249657513	-2.826627946	0.009464164	0.142867357
ENSG00000146242	TPBG	-1.595577804	1.525934362	-2.812753651	0.009772918	0.145435702
ENSG00000077809	GTF2I	-0.831447174	7.983411336	-2.996776536	0.006358022	0.113899988
ENSG00000183287	CCBE1	1.770847786	2.184766676	2.79669504	0.010142204	0.148278
ENSG00000011454	RABGAP1	-1.093546133	6.554481678	-2.970056495	0.006771126	0.117919791
ENSG00000213203	GIMAP1	1.43490306	1.189821672	2.823388271	0.00953542	0.143451839
ENSG00000151689	INPP1	1.460178553	3.938761619	2.848966788	0.008986417	0.139059037
ENSG00000145349	CAMK2D	1.211721478	5.497773734	2.931750555	0.007408365	0.124726021
ENSG00000127526	SLC35E1	-0.957972499	6.435814488	-2.961157527	0.006914318	0.119873997
ENSG00000198121	LPAR1	1.919523873	3.210846464	2.814305518	0.009737914	0.145106947
ENSG00000182600	C2orf82	-3.10776981	3.668754355	-2.829784845	0.009395216	0.142641438
ENSG00000181467	RAP2B	1.761614646	2.328043665	2.786876585	0.010374463	0.150350243
ENSG00000271605	MILR1	1.523864889	3.264964185	2.808673922	0.009865509	0.146037453
ENSG00000185345	PARK2	-1.416217674	1.388324185	-2.806206875	0.009921902	0.146361708
ENSG00000168795	ZBTB5	-1.635048021	2.027969459	-2.785808856	0.010400021	0.150350243
ENSG00000055070	SZRD1	1.262606785	5.205722541	2.909351772	0.007807092	0.128677601
ENSG00000163660	CCNL1	-1.07097889	6.997547497	-2.967541939	0.006811297	0.118356657
ENSG00000184047	DIABLO	-1.765286507	2.645807608	-2.784761433	0.010425152	0.150350243
ENSG00000155660	PDIA4	1.165768016	8.531196529	2.990407563	0.006454242	0.114676509
ENSG00000105778	AVL9	-0.992698808	4.901504746	-2.892287223	0.008124507	0.131968568
ENSG00000055163	CYFIP2	1.453314187	4.58078689	2.876947865	0.008420289	0.134819142
ENSG00000170315	UBB	1.396791146	4.862429316	2.887321546	0.008219158	0.132444654
ENSG00000163694	RBM47	-1.790338095	3.44032769	-2.810071149	0.009833705	0.145956336
ENSG00000198556	ZNF789	-1.125968711	4.501477645	-2.864560749	0.008666596	0.136691066
ENSG00000181896	ZNF101	-1.245061844	4.549688272	-2.867727008	0.008602993	0.136490971
ENSG00000140682	TGFB1I1	1.177418142	6.193736139	2.941663372	0.007238176	0.12278663
ENSG00000183549	ACSM5	1.952318354	2.013808028	2.783190515	0.010462951	0.150390404
ENSG00000186188	FFAR4	1.472928396	1.218376192	2.804247652	0.009966905	0.146707983
ENSG00000149328	GLB1L2	-2.272250764	4.040815426	-2.832309923	0.009340411	0.142153621
ENSG00000134504	KCTD1	-1.452493601	3.87249001	-2.823717415	0.009528157	0.143451839
ENSG00000111371	SLC38A1	-1.585643905	4.519817178	-2.861203335	0.008734526	0.136911457
ENSG00000163817	SLC6A20	-2.752174391	2.596185548	-2.777687826	0.01059638	0.15115255
ENSG00000170485	NPAS2	-1.353238371	6.183060778	-2.933056146	0.007385733	0.124486946
ENSG00000178726	THBD	1.656326564	1.256733644	2.795863416	0.010161683	0.148322767
ENSG00000125848	FLRT3	1.57862991	0.808624175	2.819636126	0.009618582	0.144150121
ENSG00000137872	SEMA6D	1.980956844	2.412318697	2.769465925	0.010798749	0.153203593
ENSG00000122254	HS3ST2	1.19362951	0.478347125	2.836004844	0.009260761	0.141763776
ENSG00000104870	FCGRT	1.182757758	7.208579966	2.956056418	0.006997699	0.1209261
ENSG00000159792	PSKH1	1.362604756	2.691370757	2.767978153	0.010835757	0.153451137
ENSG00000184009	ACTG1	1.251540113	10.43686029	2.988794092	0.006478838	0.114676509
ENSG00000103489	XYLT1	-2.065704183	4.032419193	-2.820100444	0.009608254	0.144132996
ENSG00000204472	AIF1	1.747763487	4.908471563	2.873592224	0.008486347	0.135187081
ENSG00000250038	RP11-180C1.1	1.267828133	0.363786385	2.833913637	0.009305761	0.141763776
ENSG00000115234	SNX17	0.88697847	7.921497634	2.960713526	0.006921537	0.119873997
ENSG00000163814	CDCP1	-2.222696885	3.847117831	-2.812506029	0.009778514	0.145435702
ENSG00000180739	S1PR5	1.512165337	0.99774258	2.797065713	0.010133532	0.148278
ENSG00000256771	ZNF253	-1.591375746	4.137362091	-2.822654727	0.009551624	0.143557981
ENSG00000062038	CDH3	-2.884790051	6.140450014	-2.92155649	0.007587362	0.126495746
ENSG00000242866	STRC	-1.886049061	1.429592436	-2.777717789	0.01059565	0.15115255
ENSG00000165084	C8orf34	1.327076639	0.844748225	2.801578934	0.010028516	0.147200608
ENSG00000174652	ZNF266	-1.204750166	4.800158529	-2.855938191	0.008842076	0.137616139
ENSG00000134775	FHOD3	1.897756452	4.443321743	2.837631823	0.009225894	0.141763776
ENSG00000162819	BROX	-1.144641095	5.602165639	-2.889433957	0.008178765	0.132354143
ENSG00000162148	PPP1R32	-1.67217626	2.451403434	-2.744326601	0.011440425	0.159424256

ENSG00000085365	SCAMP1	-1.138432794	4.195035718	-2.809617677	0.009844016	0.145956336
ENSG00000111341	MGP	-2.466750702	9.583900783	-2.958577363	0.006956373	0.120344487
ENSG00000147669	POLR2K	-1.467678723	5.16155969	-2.862486451	0.008708505	0.136781937
ENSG00000103855	CD276	1.112422734	6.803964159	2.921177359	0.007594098	0.126495746
ENSG00000138336	TET1	-1.614957745	1.554700499	-2.751271375	0.011259649	0.158044558
ENSG00000147687	TATDN1	-1.788539872	3.647438886	-2.77406381	0.010685133	0.15214195
ENSG00000129538	RNASE1	1.753969682	7.255588202	2.928197024	0.007470299	0.125160475
ENSG00000135929	CYP27A1	-1.475499686	6.04775341	-2.897008109	0.008035485	0.131065279
ENSG00000105851	PIK3CG	1.593387054	1.807283659	2.739746299	0.01156115	0.160536285
ENSG00000162522	KIAA1522	-1.488729701	5.199618925	-2.861998587	0.00871839	0.136800258
ENSG00000255524	NPIPB8	-1.230179048	5.288157564	-2.865134146	0.008655044	0.136691066
ENSG00000235531	RP11-383H13.1	1.749039441	1.102217016	2.76841038	0.010824993	0.153437187
ENSG00000166295	ANAPC16	-1.167113228	5.893363573	-2.88882346	0.00819042	0.132354143
ENSG00000149295	DRD2	-2.049399579	1.577864991	-2.74811732	0.011341413	0.158749436
ENSG00000214046	SMIM7	-1.253475154	5.633184766	-2.875733435	0.008444139	0.134925654
ENSG00000268674	FAM231B	1.141000072	0.335231866	2.804613325	0.009958491	0.146707983
ENSG00000160190	SLC37A1	-1.500116559	4.564935905	-2.818999364	0.009632764	0.144224901
ENSG00000170145	SIK2	-1.224144775	4.834776075	-2.834056279	0.009302685	0.141763776
ENSG00000185324	CDK10	-1.086698657	6.415575857	-2.900092863	0.007977819	0.130395788
ENSG00000062282	DGAT2	-2.766039157	3.959820321	-2.780718393	0.010522697	0.150786888
ENSG00000161642	ZNF385A	1.467382403	5.292676638	2.85715049	0.008817201	0.137388984
ENSG00000111266	DUSP16	-1.89509201	3.796712321	-2.77535705	0.010653381	0.151827616
ENSG00000170458	CD14	2.267982634	3.95838321	2.783468829	0.010456245	0.150390404
ENSG00000197124	ZNF682	-1.190586432	3.177889408	-2.738546091	0.011592982	0.160805518
ENSG00000197933	ZNF823	-1.566931156	1.559993964	-2.734504284	0.011700791	0.161617181
ENSG00000136250	AOAH	2.153970729	4.386523852	2.803814919	0.009976871	0.146715601
ENSG00000183023	SLC8A1	1.725740431	1.146470755	2.750579542	0.011277536	0.158137454
ENSG00000138380	CARF	-1.624228013	3.408473736	-2.743860277	0.011452662	0.159453162
ENSG00000185760	KCNQ5	-1.490142416	1.141477291	-2.749027696	0.011317756	0.158559735
ENSG00000186812	ZNF397	-1.522538738	2.897159562	-2.723296095	0.012004724	0.164511902
ENSG00000053371	AKR7A2	1.29011434	4.351897556	2.796397607	0.010149167	0.148278
ENSG00000134444	KIAA1468	-1.428341013	4.901169457	-2.827606783	0.009442735	0.142867357
ENSG00000133627	ACTR3B	-1.302467237	4.92675504	-2.826128191	0.009475123	0.142867357
ENSG00000002016	RAD52	-1.322353513	4.243929052	-2.785584284	0.010405405	0.150350243
ENSG00000170734	POLH	-1.197183661	5.792682459	-2.864395149	0.008669934	0.136691066
ENSG00000050438	SLC4A8	1.330416402	2.289943309	2.705277917	0.01250898	0.169059786
ENSG00000170190	SLC16A5	-1.270538012	3.344571937	-2.734744884	0.011694347	0.161617181
ENSG00000117318	ID3	1.784946482	6.59748934	2.888997706	0.008187092	0.132354143
ENSG00000125703	ATG4C	1.087170643	4.261473422	2.780973622	0.010516514	0.150786888
ENSG00000175294	CATSPER1	1.534361484	1.124447489	2.738242958	0.011601035	0.160805518
ENSG00000168350	DEGS2	-1.69348127	1.239703843	-2.732784237	0.011746957	0.161969692
ENSG00000138395	CDK15	1.153521462	0.335231866	2.778563672	0.010575036	0.151122846
ENSG00000167972	ABCA3	-1.561603163	5.224759294	-2.829577253	0.009399735	0.142641438
ENSG00000039523	FAM65A	0.851057621	6.356431472	2.874619825	0.008466066	0.135001059
ENSG00000136026	CKAP4	1.320102656	5.349115223	2.834849677	0.009285593	0.141763776
ENSG00000169306	IL1RAPL1	1.13454335	0.335231866	2.77359639	0.010696632	0.152167587
ENSG00000163481	RNF25	0.929863813	5.551847711	2.840037045	0.009174577	0.141274079
ENSG00000166348	USP54	-1.332160571	5.531123475	-2.836566083	0.009248719	0.141763776
ENSG00000067840	PDZD4	1.760561386	2.548622001	2.689843186	0.012956742	0.171949395
ENSG00000184588	PDE4B	-1.561288658	4.67801555	-2.792624762	0.010237883	0.149296122
ENSG00000250722	SEPP1	2.002554477	5.935979499	2.852959444	0.008903478	0.138076029
ENSG00000175029	CTBP2	-1.050729661	4.631149506	-2.785950292	0.010396632	0.150350243
ENSG00000183688	FAM101B	1.300783631	3.642814938	2.72890769	0.011851635	0.162852804
ENSG00000171940	ZNF217	-1.298130798	4.277922292	-2.76441294	0.010924928	0.154574438
ENSG00000132485	ZRANB2	-1.023227799	6.556227155	-2.868844495	0.008580652	0.136274304
ENSG00000135378	PRRG4	-1.514583972	3.980078593	-2.747708038	0.011352065	0.158756903
ENSG00000033100	CHPF2	1.130899294	5.415955963	2.828606535	0.009420895	0.142686548
ENSG00000186635	ARAP1	0.909424226	7.923191734	2.894068235	0.008090813	0.131627361
ENSG00000125089	SH3TC1	1.363317402	4.682458738	2.788564592	0.010334177	0.150350243
ENSG00000198113	TOR4A	1.773527426	2.273341268	2.681426634	0.013207221	0.172781115
ENSG00000173209	AHSA2	-1.191369089	6.070428433	-2.84932491	0.008978948	0.139059037
ENSG00000198300	PEG3	-1.951727797	1.486873417	-2.704493893	0.012531369	0.169071118
ENSG00000072210	ALDH3A2	-1.073456393	6.008210564	-2.847976598	0.009007099	0.139241764
ENSG00000164976	KIAA1161	-1.673543218	3.467000839	-2.714117089	0.012259163	0.16683464
ENSG00000183779	ZNF703	1.527601159	2.349905201	2.68283268	0.013165063	0.172781115

ENSG00000117620	SLC35A3	-1.115960498	4.74163113	-2.787451983	0.010360713	0.150350243
ENSG00000182704	TSKU	1.703921796	2.060126215	2.6795501	0.013263685	0.172781115
ENSG00000184194	GPR173	1.847406432	1.984741891	2.678400436	0.01329839	0.172781115
ENSG00000019144	PHLDB1	0.990548547	7.635808626	2.881850445	0.008324658	0.13383423
ENSG00000106012	IQCE	-1.14271832	5.275572659	-2.81057008	0.009822372	0.145949656
ENSG00000196511	TPK1	1.676729829	1.562646193	2.690970964	0.012923521	0.171704461
ENSG00000147443	DOK2	1.952686199	2.954220495	2.691720067	0.012901499	0.171557134
ENSG00000157322	CLEC18A	-2.221603132	2.92393203	-2.686569474	0.013053633	0.172247198
ENSG00000157349	DDX19B	-1.778261064	2.206170491	-2.675760909	0.013378393	0.173256777
ENSG00000172888	ZNF621	-1.458240196	2.874808542	-2.681618225	0.013201469	0.172781115
ENSG00000155850	SLC26A2	-2.039753025	2.708790069	-2.681787171	0.013196399	0.172781115
ENSG00000126264	HCST	1.561611425	2.563204112	2.675790599	0.013377491	0.173256777
ENSG00000254901	MEF2BNB	-1.152528519	3.907717528	-2.726978401	0.011904059	0.163417836
ENSG00000162585	C1orf86	1.109109765	5.93030242	2.835527536	0.009271014	0.141763776
ENSG00000088387	DOCK9	-1.323308834	6.912309168	-2.86249141	0.008708405	0.136781937
ENSG00000196961	AP2A1	0.947553404	7.848376931	2.878830128	0.00838345	0.134366413
ENSG00000188039	NWD1	-2.070325071	3.500803952	-2.711479431	0.012333213	0.167144566
ENSG00000135074	ADAM19	1.700858099	4.418758295	2.759908532	0.011038583	0.155901358
ENSG00000074047	GLI2	1.847418407	2.789855935	2.678226267	0.013303655	0.172781115
ENSG00000165507	C10orf10	-1.839539776	3.779484255	-2.716225636	0.012200269	0.166320087
ENSG00000136943	CTSV	-2.779962039	4.483042453	-2.755793585	0.011143387	0.156957711
ENSG00000175489	LRRC25	1.786125763	2.987209239	2.678555884	0.013293693	0.172781115
ENSG00000188295	ZNF669	-1.28998732	3.469020506	-2.697747281	0.012725592	0.170227843
ENSG00000144504	ANKMY1	-1.450489162	4.204282771	-2.737497362	0.011620865	0.160938214
ENSG00000145949	MYLK4	1.729545759	2.380207974	2.66375253	0.013748109	0.17581221
ENSG00000197629	MPEG1	1.614238002	1.550609856	2.679349174	0.013269745	0.172781115
ENSG00000135363	LMO2	1.242796326	3.318229089	2.68823966	0.013004116	0.172192058
ENSG00000143119	CD53	1.734294573	4.339010155	2.745939569	0.011398196	0.159118408
ENSG00000171160	MORN4	-1.553081272	2.825381885	-2.666050841	0.013676613	0.175326586
ENSG00000106004	HOXA5	1.708405104	1.262417067	2.686962842	0.013041955	0.172247198
ENSG00000204822	MRPL53	1.429968415	0.86677149	2.70795464	0.012432827	0.168175427
ENSG00000187527	ATP13A5	-2.242449892	1.933153065	-2.656631383	0.01397187	0.177086115
ENSG00000182318	ZSCAN22	-1.526491338	2.649705322	-2.655588881	0.014004913	0.177108472
ENSG00000197565	COL4A6	2.408803102	1.492827621	2.671459664	0.013509733	0.174040408
ENSG00000160087	UBE2J2	1.264004776	6.009678626	2.821520468	0.009576732	0.14379761
ENSG00000182175	RGMA	-2.396429134	3.568011833	-2.689601465	0.012963873	0.171949395
ENSG00000163297	ANTXR2	1.339636009	5.130570188	2.783052631	0.010466275	0.150390404
ENSG00000140398	NEIL1	-1.471855251	4.309146754	-2.732347773	0.011758699	0.161989248
ENSG00000124107	SLPI	-3.583014449	6.632717507	-2.838156526	0.009214676	0.14175243
ENSG00000114013	CD86	1.78026802	3.338450173	2.677921344	0.013312878	0.172781115
ENSG00000196646	ZNF136	-1.374146831	2.993728255	-2.661517533	0.013817972	0.176131342
ENSG00000112799	LY86	1.856397975	2.471029075	2.647904622	0.014250748	0.179173461
ENSG00000109814	UGDH	1.009389792	6.291157624	2.826571345	0.009465405	0.142867357
ENSG00000150471	LPHN3	1.90802314	1.336248103	2.67364926	0.013442722	0.173461969
ENSG00000133937	GSC	1.342809207	0.910122408	2.695604139	0.01278788	0.170624681
ENSG00000233276	GPX1	1.564850548	3.587147282	2.688943188	0.012983311	0.172061774
ENSG00000117411	B4GALT2	1.16253763	6.089965319	2.817928084	0.009656667	0.144444961
ENSG00000066336	SPI1	1.955959253	5.396901508	2.789620219	0.010309059	0.150194467
ENSG00000102595	UGGT2	-1.045469557	6.151140148	-2.816946506	0.009678618	0.144635429
ENSG00000134152	KATNBL1	-1.582565482	3.521072206	-2.678821079	0.013285683	0.172781115
ENSG00000174950	CD164L2	-1.97644861	1.458267352	-2.664722366	0.013717897	0.175712261
ENSG00000140279	DUOX2	-2.00958889	2.353932563	-2.640189772	0.014501632	0.180513772
ENSG00000187840	EIF4EBP1	1.131182239	5.397186523	2.784839178	0.010423285	0.150350243
ENSG00000106536	POU6F2	1.214406085	0.471815872	2.711635642	0.012328816	0.167144566
ENSG00000101004	NINL	-1.466034911	5.898958177	-2.803413535	0.009986123	0.146715601
ENSG00000061337	LZTS1	1.732471014	2.631158075	2.645190357	0.014338546	0.179414777
ENSG00000154447	SH3RF1	-1.344192821	4.776348309	-2.746531923	0.011382724	0.159043925
ENSG00000166681	NGFRAP1	2.296075681	4.015293326	2.704549512	0.012529779	0.169071118
ENSG00000181085	MAPK15	-3.398939058	4.079476354	-2.703063663	0.012572308	0.169332261
ENSG00000120498	TEX11	1.685039911	0.691722199	2.698143809	0.012714098	0.170219214
ENSG00000142621	FHAD1	1.808035028	2.050039911	2.632904097	0.01474236	0.182228752
ENSG00000153253	SCN3A	2.103866505	1.364787815	2.657453646	0.013945859	0.176899333
ENSG00000106392	C1GALT1	-1.469312383	3.277240988	-2.654494227	0.014039688	0.177111095
ENSG00000124664	SPDEF	-1.998595834	1.457576097	-2.651053445	0.014149525	0.178186348
ENSG00000065717	TLE2	-1.472311084	6.563810893	-2.815838852	0.009703446	0.144868476

ENSG00000102034	ELF4	1.314139728	4.393080962	2.716976149	0.012179371	0.16617957
ENSG00000122691	TWIST1	1.686258898	2.827286903	2.633250482	0.01473083	0.182228752
ENSG00000024422	EHD2	1.180957085	5.630894231	2.781594919	0.010501477	0.15075816
ENSG00000186862	PDZD7	-1.505809908	2.648898044	-2.626360111	0.014961772	0.182908913
ENSG00000165030	NFIL3	-1.434925522	2.177387592	-2.620259538	0.01516907	0.18393963
ENSG00000105792	C7orf63	-1.523294937	2.959883224	-2.632535637	0.014754633	0.182228752
ENSG00000172061	LRRC15	1.843479949	2.958130864	2.631823958	0.014778366	0.182301373
ENSG00000130224	LRCH2	1.662586653	1.643195739	2.628681202	0.0148836	0.182908913
ENSG00000168779	SHOX2	2.646407867	2.321028832	2.615674056	0.015326655	0.184934705
ENSG00000168275	COA6	-1.353648235	2.402340226	-2.612000152	0.015454017	0.185673031
ENSG00000099849	RASSF7	-1.707780016	2.989041952	-2.626190464	0.0149675	0.182908913
ENSG00000078900	TP73	2.329841638	1.797408479	2.619695354	0.015188377	0.184031521
ENSG00000094975	SUCO	-1.213322861	5.277237493	-2.751225321	0.011260838	0.158044558
ENSG00000133055	MYBPH	-1.713356846	1.309268044	-2.6435099	0.014393159	0.179794167
ENSG00000184232	OAF	1.439000822	5.074491464	2.740330262	0.011545691	0.160463636
ENSG00000203697	CAPN8	-2.196304742	1.632394389	-2.62539542	0.014994374	0.183094728
ENSG00000015532	XYLT2	0.958721148	5.382700153	2.754138732	0.0111858	0.157413795
ENSG00000167766	ZNF83	-1.146534781	6.114961391	-2.783125379	0.010464521	0.150390404
ENSG00000204385	SLC44A4	-1.691669572	2.187418905	-2.60603575	0.015662896	0.186450486
ENSG00000097021	ACOT7	1.184418519	4.995812038	2.733231083	0.011734948	0.161946405
ENSG00000198695	MT-ND6	-1.104475275	7.019575112	-2.80608375	0.009924725	0.146361708
ENSG00000129595	EPB41L4A	-1.903541112	2.619328947	-2.609140473	0.015553838	0.185679876
ENSG00000139946	PELI2	1.646040041	1.300084562	2.636256956	0.014631115	0.181483659
ENSG00000130338	TULP4	-1.096508088	4.237314554	-2.684577254	0.013112929	0.172468542
ENSG00000149090	PAMR1	2.132406035	3.045545502	2.620675655	0.015154845	0.183927613
ENSG00000127418	FGFRL1	-1.830312969	5.492793333	-2.751613935	0.011250802	0.158044558
ENSG00000070731	ST6GALNAC2	-1.804815488	4.331604999	-2.685957078	0.013071833	0.172247198
ENSG00000114923	SLC4A3	-1.65027039	5.614005797	-2.756476424	0.011125931	0.156852637
ENSG00000147145	LPAR4	1.718490809	1.125833062	2.638907037	0.014543746	0.180828777
ENSG00000189043	NDUFA4	-1.52101756	2.692914201	-2.603016532	0.015769637	0.186686591
ENSG00000156162	DPY19L4	-1.048743256	5.090149755	-2.728999599	0.011849143	0.162852804
ENSG00000135318	NT5E	-2.031827013	3.911193582	-2.660133967	0.013861388	0.176140249
ENSG00000092010	PSME1	0.838065913	7.486411386	2.807635125	0.009889217	0.146225121
ENSG00000068438	FTSJ1	0.755766228	6.525227664	2.787394966	0.010362075	0.150350243
ENSG00000091137	SLC26A4	-1.435004849	1.383514716	-2.622867552	0.015080122	0.183712885
ENSG00000135930	EIF4E2	1.096831717	4.95867452	2.720397951	0.012084517	0.165172606
ENSG00000105088	OLFM2	2.286981089	4.931554941	2.717025475	0.012177998	0.16617957
ENSG00000267508	ZNF285	-1.503887016	2.048258834	-2.597530966	0.015965317	0.18696929
ENSG00000175592	FOSL1	1.367000442	1.327943467	2.623285021	0.01506593	0.183682595
ENSG00000121933	ADORA3	1.704481316	2.46250803	2.598818629	0.015919181	0.186907667
ENSG00000167553	TUBA1C	1.132624527	5.058731808	2.725237682	0.011951546	0.163926322
ENSG00000179178	TMEM125	-1.615551281	1.225419064	-2.627983507	0.014907057	0.182908913
ENSG00000183258	DDX41	0.826835367	7.330895597	2.799779363	0.010070266	0.147546525
ENSG00000172638	EFEMP2	1.110916645	7.363735721	2.799752516	0.01007089	0.147546525
ENSG00000173264	GPR137	1.044284316	4.441519997	2.685879344	0.013074145	0.172247198
ENSG00000181817	LSM10	1.117808514	3.968517402	2.6555075	0.014007496	0.177108472
ENSG00000185917	SETD4	-1.081443383	4.705864534	-2.699104641	0.012686289	0.169991944
ENSG00000127328	RAB3IP	-1.621566631	3.147544376	-2.608933436	0.015561088	0.185679876
ENSG00000105523	FAM83E	-2.092796666	1.483279058	-2.609276612	0.015549072	0.185679876
ENSG00000064607	SUGP2	-1.00581338	6.561779411	-2.780223251	0.010534703	0.150821184
ENSG00000101000	PROCR	1.497070581	2.913257674	2.600806225	0.015848211	0.186686591
ENSG00000122970	IFT81	-1.151287499	4.986964996	-2.713290096	0.012282335	0.167003569
ENSG00000126821	SGPP1	1.787011599	2.288621662	2.585239727	0.016412046	0.189353983
ENSG00000114480	GBE1	1.180577354	6.006218667	2.760967281	0.011011769	0.155662761
ENSG00000167615	LENG8	-1.288675136	8.196854917	-2.807302296	0.009896825	0.146225121
ENSG00000179862	CITED4	-1.554665819	1.1882994	-2.620938171	0.015145878	0.183927613
ENSG00000243716	NPIPB5	-1.007718129	4.48207983	-2.679323465	0.01327052	0.172781115
ENSG00000204946	ZNF783	-1.141069293	4.896517214	-2.703764086	0.012552243	0.169207259
ENSG00000196757	ZNF700	-1.478977721	2.669199708	-2.58717027	0.016341115	0.188813278
ENSG00000168792	ABHD15	1.280588597	1.606104943	2.600901022	0.015844834	0.186686591
ENSG00000100139	MICALL1	-1.135143506	7.069302271	-2.784976748	0.010419982	0.150350243
ENSG00000180628	PCGF5	-1.17165219	3.634576753	-2.624571425	0.015022275	0.183292777
ENSG00000175183	CSRP2	1.46383774	4.280052772	2.667346041	0.013636477	0.175241567
ENSG00000134533	RERG	-2.048810132	2.709921871	-2.587205591	0.01633982	0.188813278
ENSG00000008283	CYB561	-1.294818528	6.445168078	-2.769524656	0.010797291	0.153203593

ENSG00000150636	CCDC102B	1.898648153	2.986323506	2.592808308	0.016135601	0.187771048
ENSG00000180113	TDRD6	-1.625438593	1.289586014	-2.615471986	0.015333634	0.184934705
ENSG00000164125	FAM198B	1.897028843	3.222365954	2.602977377	0.015771026	0.186686591
ENSG00000122335	SERAC1	-1.334367479	4.19368721	-2.65564121	0.014003253	0.177108472
ENSG00000214595	EML6	-1.473453818	2.207332117	-2.5760875	0.016752249	0.191170569
ENSG00000136155	SCEL	-2.148334574	1.563781519	-2.591574748	0.016180358	0.188063704
ENSG00000188747	NOXA1	-1.799381239	3.978686824	-2.63631876	0.014629072	0.181483659
ENSG00000173947	PIFO	-1.780110358	1.626316674	-2.587593722	0.016325595	0.188813278
ENSG00000213654	GPSM3	1.782237694	3.663709544	2.621532088	0.015125608	0.183927613
ENSG00000111344	RASAL1	-2.808901169	3.066382449	-2.587885462	0.01631491	0.188813278
ENSG00000143553	SNAPIN	1.067347901	4.776946779	2.686422301	0.013058005	0.172247198
ENSG00000107282	APBA1	-2.34154849	3.167189868	-2.595924396	0.016023056	0.187485283
ENSG00000127585	FBXL16	-2.012402264	1.753326362	-2.579234272	0.016634542	0.190421721
ENSG00000258227	CLEC5A	1.650802406	1.253233585	2.600676013	0.015852852	0.186686591
ENSG00000100599	RIN3	1.094929059	4.715191488	2.680518347	0.013234523	0.172781115
ENSG00000171451	DSEL	1.621522937	2.235914023	2.565742171	0.017144729	0.193816957
ENSG00000088002	SULT2B1	-1.564474704	1.177677503	-2.601310162	0.015830265	0.186686591
ENSG00000145242	EPHA5	1.267060082	0.587072225	2.635437995	0.014658215	0.181633274
ENSG00000172785	CBWD1	-0.936683382	5.561135018	-2.720644182	0.012077718	0.165172606
ENSG00000169223	LMAN2	0.944082171	8.112827495	2.785268822	0.010412971	0.150350243
ENSG00000173681	CXorf23	-1.404664912	3.664684482	-2.604675528	0.015710901	0.186616012
ENSG00000148429	USP6NL	-1.205269191	4.278726922	-2.642060499	0.014440419	0.180114951
ENSG00000169994	MYO7B	-1.444970559	1.074565319	-2.602530847	0.015786871	0.186686591
ENSG00000127124	HIVEP3	1.071071319	3.783388693	2.609523763	0.015540424	0.185679876
ENSG00000124422	USP22	0.761083481	7.624068488	2.771563982	0.010746764	0.152742281
ENSG00000173372	C1QA	2.013295097	4.150700631	2.636512487	0.014622669	0.181483659
ENSG00000149231	CCDC82	-0.959347537	5.152663707	-2.69302917	0.012863099	0.171236839
ENSG00000013016	EHD3	1.65534558	3.028545698	2.569450931	0.017003049	0.192631655
ENSG00000075643	MOCOS	-2.18746644	4.480391282	-2.660271677	0.013857061	0.176140249
ENSG00000117868	ESYT2	-1.027571922	8.245862867	-2.778617693	0.01057372	0.151122846
ENSG00000104903	LYL1	1.478001925	2.654729433	2.556310908	0.017509996	0.196109452
ENSG00000100583	SAMD15	1.191602743	0.658282048	2.620631407	0.015156357	0.183927613
ENSG00000101098	RIMS4	1.289330272	0.658282048	2.618692584	0.015222748	0.18429737
ENSG00000184635	ZNF93	-1.13552594	4.148772966	-2.627168594	0.014934499	0.182908913
ENSG00000197077	KIAA1671	-1.002010433	4.68655901	-2.660064662	0.013863566	0.176140249
ENSG00000124570	SERPINB6	0.921817202	6.65180968	2.744876621	0.011426008	0.159364888
ENSG00000164776	PHKG1	-1.441513762	3.494493301	-2.582519093	0.016512496	0.189676114
ENSG00000148985	PGAP2	-1.151509265	3.591254745	-2.587989492	0.016311102	0.188813278
ENSG00000144026	ZNF514	-1.409725441	3.763172587	-2.598753557	0.01592151	0.186907667
ENSG00000174498	IGDCC3	1.359480956	0.838216971	2.599647783	0.015889539	0.186898618
ENSG00000186665	C17orf58	1.375996047	2.681913439	2.547366242	0.017863114	0.197903296
ENSG00000105810	CDK6	-1.582328686	3.12703561	-2.56172798	0.017299318	0.195002582
ENSG00000167613	LAIR1	1.657828451	5.841763408	2.714112964	0.012259279	0.16683464
ENSG00000118322	ATP10B	-1.620685946	1.506356117	-2.56309275	0.017246614	0.194688217
ENSG00000048707	VPS13D	-1.007284477	6.534484808	-2.735115143	0.011684437	0.161617181
ENSG00000175066	GK5	-1.340639982	4.548729921	-2.646444801	0.014297906	0.179214115
ENSG00000129255	MPDU1	0.999713878	4.992909608	2.672711216	0.013471392	0.173689079
ENSG00000104361	NIPAL2	-1.623075829	4.864136612	-2.66268061	0.013781574	0.175977978
ENSG00000007402	CACNA2D2	2.228422312	1.954836623	2.544123142	0.017992778	0.198820193
ENSG00000172830	SSH3	-1.040077005	5.056264633	-2.674977543	0.013402224	0.173367101
ENSG00000166426	CRABP1	-3.237935407	3.36826288	-2.570396252	0.016967112	0.192631655
ENSG00000017797	RALBP1	-1.063540819	4.660365137	-2.646988422	0.014280327	0.179214115
ENSG00000165115	KIF27	-1.402184883	3.012178251	-2.550527597	0.017737558	0.196961129
ENSG00000137269	LRRC1	-1.84670335	4.606824607	-2.640028932	0.014506906	0.180513772
ENSG00000038945	MSR1	1.425962986	4.961980712	2.662618932	0.013783502	0.175977978
ENSG00000166532	RIMKLB	-1.178900768	3.555737582	-2.574270863	0.016820555	0.19167126
ENSG00000010810	FYN	1.13312136	6.520746444	2.728875032	0.011852521	0.162852804
ENSG00000139209	SLC38A4	1.398059281	0.658282048	2.60356652	0.015750142	0.186686591
ENSG00000187742	SECISBP2	-0.909904451	5.986183386	-2.709743171	0.012382187	0.167634945
ENSG00000101017	CD40	1.668761056	4.404232724	2.626663131	0.014951544	0.182908913
ENSG00000198520	C1orf228	-1.739555763	1.91758925	-2.534638033	0.018377058	0.201381423
ENSG00000253379	RP11-1102P16.1	1.157497871	0.478347125	2.609344778	0.015546686	0.185679876
ENSG00000232560	C21orf37	1.178126406	0.500370391	2.607866196	0.015598513	0.185985002
ENSG00000100365	NCF4	1.478407771	4.059340805	2.604288055	0.015724601	0.186637457
ENSG00000146648	EGFR	-2.10834309	4.433415805	-2.629867236	0.014843804	0.182821138

ENSG00000213865	C8orf44	-1.392974937	3.182362429	-2.550233039	0.017749222	0.196961129
ENSG00000165813	C10orf118	-1.549795887	4.092536731	-2.60048628	0.015859615	0.186686591
ENSG00000132196	HSD17B7	-1.410625262	3.018540449	-2.541944331	0.018080382	0.19936702
ENSG00000115468	EFHD1	-1.701513182	3.996488823	-2.593638248	0.016105554	0.187771048
ENSG00000177054	ZDHHC13	-1.153776304	5.172773057	-2.6663244	0.013668127	0.175326586
ENSG00000167994	RAB3IL1	1.109378083	5.089465013	2.661159663	0.01382919	0.176131342
ENSG00000030582	GRN	1.160757588	8.895936918	2.759176958	0.011057147	0.156023111
ENSG00000113722	CDX1	1.079753259	0.428464954	2.605743446	0.015673201	0.186450486
ENSG00000091640	SPAG7	0.876356262	5.796962879	2.693948144	0.012836206	0.17112397
ENSG00000211455	STK38L	-1.561600077	5.7863799	-2.692913101	0.012866499	0.171236839
ENSG00000124839	RAB17	-1.72024039	2.189445101	-2.519135339	0.019021632	0.205274026
ENSG00000224877	C17orf89	1.340114476	2.275559154	2.521213366	0.018934027	0.205004974
ENSG00000130755	GMFG	1.398274768	5.158372369	2.662083582	0.013800247	0.176048513
ENSG00000111052	LIN7A	1.469898922	1.185647171	2.555552331	0.017539688	0.196139981
ENSG00000083750	RRAGB	-1.032768788	4.625090532	-2.62680756	0.014946672	0.182908913
ENSG00000135454	B4GALNT1	2.179419586	3.679785608	2.568495276	0.017039451	0.192904784
ENSG00000137673	MMP7	-3.688984423	4.463580776	-2.627611298	0.014919585	0.182908913
ENSG00000077420	APBB1IP	1.758285109	4.206221631	2.598578634	0.015927771	0.186907667
ENSG00000011258	MBTD1	-1.274545614	4.153232533	-2.59300823	0.016128358	0.187771048
ENSG00000205220	PSMB10	1.584996268	2.398144553	2.520655571	0.018957505	0.205004974
ENSG00000141480	ARRB2	1.443129744	6.112222366	2.699790203	0.012666482	0.169991944
ENSG00000168495	POLR3D	0.982557161	5.514940101	2.675620774	0.013382653	0.173256777
ENSG00000184863	RBM33	-0.854071667	5.671827226	-2.680063032	0.013248229	0.172781115
ENSG00000206052	DOK6	1.683552477	1.198586329	2.551070424	0.017716082	0.196961129
ENSG00000164031	DNAJB14	-1.37001839	2.37957416	-2.513172877	0.019275096	0.206166008
ENSG00000108826	MRPL27	0.752162096	6.096822275	2.696771977	0.012753902	0.170461229
ENSG00000203883	SOX18	1.416399765	2.723951994	2.518078089	0.019066349	0.205350808
ENSG00000178665	ZNF713	-1.171563632	3.858868547	-2.571626034	0.016920467	0.192435276
ENSG00000173726	TOMM20	-1.614611456	3.961810914	-2.575747365	0.016765018	0.191177251
ENSG00000222038	POTEJ	0.981815541	0.291880949	2.598294218	0.015937955	0.186907667
ENSG00000183020	AP2A2	0.751248694	7.249701458	2.721343135	0.012058439	0.165103808
ENSG00000160781	PAQR6	-1.835709755	3.34633303	-2.537328867	0.018267271	0.200863179
ENSG00000151640	DPYSL4	2.074186545	3.393251993	2.547010977	0.017877275	0.197903296
ENSG00000050767	COL23A1	2.062146964	2.469354376	2.506404834	0.01956661	0.207381752
ENSG00000142634	EFHD2	1.402517616	5.251302657	2.65461819	0.014035746	0.177111095
ENSG00000104321	TRPA1	1.906500691	1.225130473	2.540600344	0.018134618	0.199634577
ENSG00000171115	GIMAP8	1.550667185	2.29431074	2.503718783	0.019683436	0.208049749
ENSG00000117222	RBBP5	-0.728968994	5.615967768	-2.669080209	0.01358291	0.174839572
ENSG00000185046	ANKS1B	1.451939604	2.516795936	2.506369524	0.019568142	0.207381752
ENSG00000117616	C1orf63	-0.877678255	6.532499976	-2.700710728	0.012639933	0.16980581
ENSG00000015171	ZMYND11	-1.003061791	6.046621561	-2.68546098	0.013086595	0.172266576
ENSG00000152763	WDR78	-1.550236821	1.804064168	-2.514013755	0.019239161	0.20604833
ENSG00000172081	MOB3A	0.892080157	5.997988034	2.683658483	0.013140361	0.172684588
ENSG00000146414	SHPRH	-1.419851151	3.819262198	-2.560185599	0.017359061	0.195425777
ENSG00000158089	GALNT14	1.962287575	3.57510685	2.552671591	0.017652876	0.196613562
ENSG00000125746	EML2	-1.243419068	6.750226255	-2.702039219	0.012601709	0.169460292
ENSG00000155093	PTPRN2	1.590509043	1.50787839	2.520677349	0.018956588	0.205004974
ENSG00000128710	HOXD10	1.265252299	0.500370391	2.57659466	0.016733225	0.191170569
ENSG00000204624	PTCHD2	1.297862691	0.500370391	2.576344039	0.016742624	0.191170569
ENSG00000204947	ZNF425	-1.476514477	1.823359042	-2.508947667	0.019456607	0.207381752
ENSG00000164111	ANXA5	0.713615399	9.952969139	2.741135143	0.011524417	0.160309954
ENSG00000068078	FGFR3	-2.38186624	5.415139681	-2.643567721	0.014391276	0.179794167
ENSG00000185885	IFITM1	-1.677438377	2.174115912	-2.490074007	0.020286971	0.212089553
ENSG00000033867	SLC4A7	-1.036225366	4.511209105	-2.592854184	0.016133939	0.187771048
ENSG00000186335	SLC36A2	1.813407437	1.573211416	2.502417668	0.019740259	0.208049749
ENSG00000134369	NAV1	1.186922067	6.53128934	2.682626009	0.013171251	0.172781115
ENSG00000164167	LSM6	1.106160337	4.590186908	2.592598308	0.016143212	0.187771048
ENSG00000177189	RPS6KA3	0.964601247	5.698055642	2.651899406	0.014122445	0.177988184
ENSG00000110841	PPFIBP1	-0.997540462	7.428415084	-2.699176098	0.012684224	0.169991944
ENSG00000087086	FTL	1.090939368	6.310261413	2.673726068	0.013440377	0.173461969
ENSG00000158869	FCER1G	1.873165451	5.036064157	2.617646422	0.015258685	0.184314112
ENSG00000175093	SPSB4	-1.05899355	0.844748225	-2.536342245	0.018307455	0.201164055
ENSG00000101294	HM13	0.952332897	8.484837103	2.712183939	0.012313394	0.167144566
ENSG00000056736	IL17RB	-2.168841459	2.82994258	-2.484068578	0.020558011	0.213343752
ENSG00000198753	PLXNB3	-1.499346537	5.7441532	-2.646184931	0.014306316	0.179214115

ENSG00000184792	OSBP2	-1.826104085	2.789437237	-2.483086466	0.020602655	0.213524608
ENSG00000160803	UBQLN4	-1.210980945	3.297759433	-2.502751099	0.019725683	0.208049749
ENSG00000204305	AGER	-1.596440567	3.873933719	-2.535351315	0.018347896	0.201326461
ENSG00000122862	SRGN	1.614094326	5.453918079	2.635193068	0.014666328	0.181633274
ENSG00000095564	BTAF1	-1.206278589	6.064363217	-2.658769158	0.01390434	0.176515369
ENSG00000137434	C6orf52	-1.387191135	1.068952403	-2.518038879	0.019068009	0.205350808
ENSG00000163083	INHBB	-1.75672151	2.213008958	-2.473423286	0.021046734	0.215251342
ENSG00000198856	OSTC	1.312789044	3.368570036	2.507209459	0.01953174	0.207381752
ENSG00000163257	DCAF16	-0.982594582	4.274363922	-2.559690381	0.017378284	0.19544952
ENSG00000006606	CCL26	1.323253935	0.689488796	2.540397547	0.018142815	0.199634577
ENSG00000083123	BCKDHB	-1.081900308	4.451993197	-2.571522342	0.016924395	0.192435276
ENSG00000183426	NPIPA1	-1.05938587	7.727683791	-2.695699247	0.01278511	0.170624681
ENSG00000064999	ANKS1A	-0.820204191	5.734255125	-2.643200284	0.014403242	0.179794167
ENSG00000157765	SLC34A2	-2.464083742	2.558964205	-2.474303218	0.021005932	0.215251342
ENSG00000169604	ANTXR1	1.164795177	7.328495642	2.687307972	0.013031717	0.172247198
ENSG00000169100	SLC25A6	1.067740049	8.309016674	2.701979902	0.012603413	0.169460292
ENSG00000168772	CXXC4	1.538856332	0.94520818	2.520020878	0.018984254	0.205011649
ENSG00000070669	ASNS	-1.336601697	6.502494732	-2.666770051	0.013654312	0.175326586
ENSG00000138674	SEC31A	0.665270215	9.305147436	2.711404833	0.012335314	0.167144566
ENSG00000106069	CHN2	1.828071471	2.041201356	2.465530009	0.021416045	0.216380658
ENSG00000197599	CCDC154	-1.755363672	2.6878753	-2.471487243	0.021136767	0.21563228
ENSG00000075213	SEMA3A	2.456018753	2.335003773	2.46737246	0.021329307	0.216110133
ENSG00000205592	MUC19	1.002839173	0.291880949	2.552611385	0.017655249	0.196613562
ENSG00000156968	MPV17L	-1.36459181	4.161435049	-2.540986436	0.018119022	0.199634577
ENSG00000223547	ZNF844	-1.125688467	4.753435793	-2.579419487	0.016627638	0.190421721
ENSG00000198046	ZNF667	-1.356297456	3.382990104	-2.492339009	0.02018561	0.211436856
ENSG00000135077	HAVCR2	1.338415079	4.289117325	2.551613277	0.017694629	0.196912359
ENSG00000188868	ZNF563	-1.551684302	2.635616223	-2.463695452	0.021502736	0.216977129
ENSG00000151468	CCDC3	1.954088878	3.44684769	2.495363343	0.020051002	0.210448341
ENSG00000168447	SCNN1B	-2.019708683	1.970076841	-2.462605659	0.021554388	0.217358547
ENSG00000099889	ARVCF	-1.299356965	4.99304114	-2.593040916	0.016127174	0.187771048
ENSG00000164663	USP49	-1.065224605	4.370375041	-2.55353327	0.017618948	0.196488215
ENSG00000137166	FOXP4	-1.270358288	7.108548453	-2.67374634	0.013439758	0.173461969
ENSG00000107242	PIP5K1B	1.551172731	1.554700499	2.476263144	0.020915313	0.214778915
ENSG00000141446	ESCO1	-1.189784281	3.89931734	-2.521274684	0.018931447	0.205004974
ENSG00000148346	LCN2	-3.079058373	3.140384587	-2.479547443	0.020764275	0.213732777
ENSG00000112936	C7	2.39028806	2.532601015	2.456360624	0.0218526	0.218400726
ENSG00000103035	PSMD7	0.995413444	4.444610666	2.55514952	0.017555474	0.196139981
ENSG00000081059	TCF7	1.265907815	4.522069038	2.563603795	0.017226918	0.194605878
ENSG00000147394	ZNF185	-1.808478001	4.478561132	-2.557913315	0.017447428	0.195688054
ENSG00000166851	PLK1	1.336466169	5.398549844	2.611599047	0.015467982	0.185679876
ENSG00000166311	SMPD1	0.990245488	5.149998813	2.597482916	0.015967042	0.18696929
ENSG00000196141	SPATS2L	1.075391524	7.285278183	2.66810653	0.013612961	0.175082769
ENSG00000168685	IL7R	1.894480328	2.302356541	2.453082827	0.022010645	0.219003825
ENSG00000214491	SEC14L6	-1.409889656	1.700951983	-2.460807694	0.021639855	0.217655215
ENSG00000099985	OSM	1.23066753	0.894630396	2.502693783	0.019728188	0.208049749
ENSG00000181804	SLC9A9	1.767536286	2.609812205	2.447056011	0.022304005	0.220370597
ENSG00000155085	AK9	-1.482953622	3.264602652	-2.471260971	0.021147313	0.21563228
ENSG00000197808	ZNF461	-1.371338841	3.458829874	-2.479785149	0.020753382	0.213732777
ENSG00000146197	SCUBE3	-2.435306418	4.987897268	-2.570774916	0.016952736	0.192617948
ENSG00000152402	GUCY1A2	1.561117719	1.180393984	2.480670874	0.020712843	0.213732777
ENSG00000178252	WDR6	-0.904168374	6.637448001	-2.64603753	0.014311088	0.179214115
ENSG00000248712	CCDC153	-1.405696285	1.36414368	-2.471101176	0.021154763	0.21563228
ENSG00000124731	TREM1	1.648900358	1.949911734	2.446513526	0.022330588	0.220370597
ENSG00000183580	FBXL7	1.933395684	2.336743472	2.437851825	0.022759015	0.222146161
ENSG00000169035	KLK7	-2.713618915	2.330815815	-2.436850455	0.022809032	0.222157989
ENSG00000166340	TPP1	0.943696463	7.084061141	2.654435211	0.014041565	0.177111095
ENSG00000168763	CNNM3	-0.982036577	4.411087265	-2.53460099	0.018378574	0.201381423
ENSG00000138193	PLCE1	-1.525741256	6.066681454	-2.620978893	0.015144487	0.183927613
ENSG00000165633	VSTM4	1.439182099	3.5037123	2.473511496	0.02104264	0.215251342
ENSG00000211456	SACM1L	-0.952668005	5.436577489	-2.594596738	0.016070917	0.187771048
ENSG00000166927	MS4A7	1.768661514	3.743119154	2.490561639	0.020265109	0.212089553
ENSG00000110169	HPX	-1.495172933	2.525245501	-2.434083358	0.022947774	0.222575506
ENSG00000166046	TCP11L2	-1.63707486	2.68357728	-2.435773505	0.022862938	0.222269119
ENSG00000169918	OTUD7A	1.495516293	1.012120152	2.479855828	0.020750145	0.213732777

ENSG00000071246	VASH1	1.410166767	3.976172941	2.502356942	0.019742915	0.208049749
ENSG00000175287	PHYHD1	-1.851778144	2.899744463	-2.439472962	0.022678256	0.222146161
ENSG00000054356	PTPRN	2.395523299	1.793421714	2.43881775	0.022710864	0.222146161
ENSG00000112297	AIM1	-1.557515283	5.300045665	-2.583506584	0.01647597	0.189534051
ENSG00000119397	CNTRL	-1.075049572	5.12282174	-2.570025787	0.016981187	0.192631655
ENSG00000132164	SLC6A11	-1.746359702	2.017258048	-2.428211448	0.02324478	0.223737422
ENSG00000183569	SERHL2	-1.426047513	2.90750319	-2.436231275	0.02284001	0.222227695
ENSG00000189350	FAM179A	-1.535050516	2.236988787	-2.427347958	0.023288754	0.223774551
ENSG00000092068	SLC7A8	1.919632861	4.404059917	2.522603277	0.018875641	0.204684879
ENSG00000143153	ATP1B1	-1.059073583	6.124750826	-2.614834883	0.015355659	0.185058099
ENSG00000139178	C1RL	-1.522323309	4.408759213	-2.526524948	0.018711805	0.203784336
ENSG00000151552	QDPR	0.850200439	5.400449013	2.584503491	0.016439173	0.189388441
ENSG00000154146	NRGN	1.49556588	1.09159512	2.467268792	0.021334179	0.216110133
ENSG00000111666	CHPT1	-1.240612952	6.091176922	-2.610996189	0.015488993	0.185679876
ENSG00000143344	RGL1	1.59062467	4.871865042	2.554964693	0.017562722	0.196139981
ENSG00000198786	MT-ND5	-0.978542767	8.674673015	-2.663904186	0.013743381	0.17581221
ENSG00000178295	GEN1	-1.226036857	2.872224018	-2.432325809	0.023036302	0.222575506
ENSG00000097096	SYDE2	-1.46068107	1.715329555	-2.43284171	0.023010284	0.222575506
ENSG00000122643	NT5C3A	-1.329889	3.137141943	-2.44076082	0.022614289	0.222146161
ENSG00000135903	PAX3	1.4734203	1.17003181	2.458912166	0.0217303	0.217855793
ENSG00000163923	RPL39L	-1.634085085	4.168876847	-2.502427495	0.019739829	0.208049749
ENSG00000169519	METTL15	1.598303262	1.825216282	2.424504652	0.023434097	0.224489008
ENSG00000118733	OLFM3	1.462530713	0.861158575	2.47635721	0.020910973	0.214778915
ENSG00000108984	MAP2K6	1.165190307	3.467752253	2.45684213	0.021829471	0.218400726
ENSG00000092969	TGFB2	-1.653277317	3.27587274	-2.442895852	0.02250861	0.221987809
ENSG00000160961	ZNF333	-1.022080979	4.481627516	-2.520057904	0.018982692	0.205011649
ENSG00000073910	FRY	-1.718232523	5.23804457	-2.562316067	0.017276589	0.194886383
ENSG00000162676	GFI1	1.367846146	0.854627321	2.47318564	0.021057766	0.215251342
ENSG00000100151	PICK1	-1.032329285	5.548936521	-2.582034872	0.016530434	0.189743263
ENSG00000112769	LAMA4	1.147797175	8.159616387	2.649893746	0.014186726	0.178511559
ENSG00000105438	KDELR1	0.703857639	7.902316323	2.646195845	0.014305963	0.179214115
ENSG00000174705	SH3PXD2B	0.968955105	5.735037923	2.589550214	0.016254066	0.188780569
ENSG00000178826	TMEM139	-1.56488478	1.149122984	-2.454367016	0.0219486	0.218663795
ENSG00000106772	PRUNE2	-1.880773995	3.294161856	-2.440439765	0.02263022	0.222146161
ENSG00000258986	TMEM179	1.55874902	0.811276404	2.47561054	0.020945446	0.214947679
ENSG00000169902	TPST1	1.287043735	5.13216309	2.556870394	0.017488126	0.196004419
ENSG00000099974	DDTL	-1.525472173	2.368172791	-2.412756376	0.024043568	0.228232075
ENSG00000182621	PLCB1	-1.572737726	4.248801695	-2.504020057	0.0196703	0.208049749
ENSG00000127083	OMD	1.527284007	1.475844983	2.432522403	0.023026384	0.222575506
ENSG00000119541	VPS4B	-1.166823187	5.692833371	-2.584843522	0.016426639	0.189383096
ENSG00000130706	ADRM1	1.028198677	5.108030772	2.554541275	0.017579337	0.196185896
ENSG00000123505	AMD1	-1.103360712	5.65434175	-2.581434371	0.016552705	0.189860013
ENSG00000119787	ATL2	-1.391497681	6.102497616	-2.600747952	0.015850288	0.186686591
ENSG00000105971	CAV2	-2.01520506	3.723637224	-2.4604501	0.021656891	0.217655215
ENSG00000187778	MCRS1	0.839918136	6.567166704	2.613706264	0.015394749	0.185102683
ENSG00000167754	KLK5	-2.719852917	1.991602038	-2.409376277	0.02422161	0.228400188
ENSG00000132122	SPATA6	-1.045010191	3.874750291	-2.468912555	0.021257053	0.216077398
ENSG00000123159	GIPC1	-0.911202886	7.458623622	-2.631121207	0.014801837	0.182447465
ENSG00000134590	FAM127A	1.584047954	3.121939971	2.425626905	0.023376631	0.224207035
ENSG00000133216	EPHB2	2.207443677	4.150080845	2.496715057	0.019991109	0.210100799
ENSG00000160404	TOR2A	1.487794956	3.165633299	2.426950302	0.023309031	0.223832319
ENSG00000196586	MYO6	-1.24791036	7.270465627	-2.627533287	0.014922212	0.182908913
ENSG00000106701	FSD1L	-1.407534733	1.031910015	-2.450477588	0.022137016	0.219443237
ENSG00000144730	IL17RD	-1.684701807	3.590164344	-2.447008671	0.022306323	0.220370597
ENSG00000128340	RAC2	1.648285729	3.986851243	2.479087422	0.020785369	0.213732777
ENSG00000027847	B4GALT7	1.049536615	4.775905105	2.525584866	0.018750957	0.204036944
ENSG00000186522	SEPT10	-1.358266192	2.299403579	-2.39791138	0.024834606	0.229764032
ENSG00000242265	PEG10	-1.891591282	2.836064862	-2.40885351	0.024249254	0.228400188
ENSG00000121898	CPXM2	-1.969023447	5.397640292	-2.560115779	0.01736177	0.195425777
ENSG00000110077	MS4A6A	1.519965245	6.806231355	2.613880795	0.015388698	0.185102683
ENSG00000101336	HCK	1.893382507	4.610249273	2.516244786	0.01914412	0.205606012
ENSG00000177556	ATOX1	0.720180072	6.556058896	2.606623209	0.015642206	0.186364355
ENSG00000142188	TMEM50B	-1.0025335	5.862188875	-2.57881393	0.01665022	0.190421721
ENSG00000125508	SRMS	-1.502051287	1.155007032	-2.437564398	0.022773361	0.222146161
ENSG00000149929	HIRIP3	1.305923167	3.852039126	2.458695538	0.021740659	0.217855793

ENSG00000258839	MC1R	-1.451883278	1.416878705	-2.424453985	0.023436695	0.224489008
ENSG00000011198	ABHD5	-1.207900782	3.686746272	-2.446728267	0.022320061	0.220370597
ENSG00000197329	PELI1	-1.35786982	4.374786414	-2.489716163	0.020303028	0.212089553
ENSG00000117906	RCN2	-0.977269239	7.056242161	-2.614026226	0.015383657	0.185102683
ENSG00000027644	INSRR	1.605223712	1.692957514	2.406753378	0.024360605	0.228613784
ENSG00000165973	NELL1	2.081251311	1.276463137	2.428063036	0.023252332	0.223737422
ENSG00000125337	KIF25	1.482230197	0.794866054	2.458656235	0.021742539	0.217855793
ENSG00000173369	C1QB	2.125990775	4.928925486	2.53198756	0.018485798	0.202273822
ENSG00000135443	KRT85	-1.389625267	1.663213981	-2.408283394	0.024279436	0.228416339
ENSG00000164309	CMYA5	-2.157433719	1.954114744	-2.400934833	0.024671576	0.229075308
ENSG00000156453	PCDH1	-1.60633371	3.822744847	-2.451953436	0.022065346	0.219270005
ENSG00000175455	CCDC14	-1.330459566	6.275848397	-2.588808095	0.016281163	0.188813278
ENSG00000169062	UPF3A	-0.907495945	5.933322662	-2.578825337	0.016649794	0.190421721
ENSG00000136144	RCBTB1	-1.015163886	4.497014607	-2.497585915	0.019952611	0.20991778
ENSG00000165923	AGBL2	-1.406247559	1.699338031	-2.405555109	0.024424349	0.228794202
ENSG00000102243	VGLL1	-2.442969255	1.813032606	-2.400916377	0.024672568	0.229075308
ENSG00000078401	EDN1	-1.666800667	2.127314975	-2.390325448	0.025248032	0.231302424
ENSG00000175787	ZNF169	-1.076872389	4.067627393	-2.467702258	0.021313815	0.216110133
ENSG00000086159	AQP6	-1.513269375	1.195450105	-2.434677845	0.022917901	0.222527713
ENSG00000179240	RP11-111M22.2	-1.292614365	1.584777291	-2.409177089	0.02423214	0.228400188
ENSG00000067798	NAV3	1.55212639	2.052947756	2.388601871	0.025342845	0.231329021
ENSG00000068615	REEP1	1.383010483	1.623134744	2.404986792	0.024454635	0.228794202
ENSG00000029559	IBSP	2.920413161	2.552487809	2.390188944	0.025255529	0.231302424
ENSG00000185697	MYBL1	-1.926011657	3.679172803	-2.439361151	0.022683817	0.222146161
ENSG00000100823	APEX1	1.023425609	7.138299219	2.609587238	0.015538203	0.185679876
ENSG00000172986	GXYLT2	1.378575205	4.332302057	2.483142803	0.020600091	0.213524608
ENSG00000119844	AFTPH	-0.809386318	5.836646249	-2.569720064	0.01699281	0.192631655
ENSG00000132965	ALOX5AP	1.621768699	3.927829871	2.454697175	0.021932674	0.218643959
ENSG00000204790	CBWD6	-1.162812615	3.73046813	-2.437555582	0.022773801	0.222146161
ENSG00000103187	COTL1	1.438567196	6.253955382	2.582595915	0.016509652	0.189676114
ENSG00000111252	SH2B3	1.147838792	4.890879887	2.518324857	0.019055903	0.205350808
ENSG00000128872	TMOD2	-1.299227645	4.241203182	-2.469999982	0.021206173	0.215928652
ENSG00000162909	CAPN2	-0.938817157	9.029866242	-2.63234864	0.014760866	0.182228752
ENSG00000067225	PKM	0.905302377	9.920719377	2.64133051	0.014464276	0.180269227
ENSG00000182580	EPHB3	-1.068114575	7.95295444	-2.61837037	0.015233808	0.18429737
ENSG00000137817	PARP6	-0.887754192	7.1968463	-2.605088229	0.015696322	0.186584079
ENSG00000164197	RNF180	1.469138736	1.572633122	2.398567607	0.024799137	0.229671739
ENSG00000119729	RHOQ	1.339824758	2.493608242	2.379773306	0.025833663	0.234852838
ENSG00000132341	RAN	1.029388912	5.152563673	2.530059503	0.018565277	0.202811855
ENSG00000133962	CATSPERB	-1.97363814	1.742650719	-2.39036487	0.025245867	0.231302424
ENSG00000259529	RP11-468E2.4	-1.445440097	1.026916551	-2.42795784	0.023257687	0.223737422
ENSG00000184162	NR2C2AP	-1.855140297	3.366623825	-2.410129335	0.024181838	0.228400188
ENSG00000144674	GOLGA4	-0.904056893	7.25197723	-2.601976894	0.01580655	0.186686591
ENSG00000148225	WDR31	-1.649418051	2.948156172	-2.389522749	0.025292148	0.231302424
ENSG00000215749	GOLGA6L18	-1.405988678	3.240319147	-2.401116816	0.024661795	0.229075308
ENSG00000166181	API5	-1.053154045	3.868433405	-2.438731978	0.022715136	0.222146161
ENSG00000106483	SFRP4	2.677973607	5.820071813	2.558779226	0.017413704	0.19544952
ENSG00000163516	ANKZF1	-1.099680756	6.221396867	-2.572346131	0.016893209	0.192359467
ENSG00000182141	ZNF708	-1.018295804	4.468900612	-2.479172143	0.020781483	0.213732777
ENSG00000060718	COL11A1	-2.785397347	7.227051999	-2.594209926	0.016084887	0.187771048
ENSG00000104881	PPP1R13L	-1.202115768	6.81421585	-2.588292089	0.016300029	0.188813278
ENSG00000109066	TMEM104	1.147090479	4.175169205	2.456609126	0.02184066	0.218400726
ENSG00000148218	ALAD	-1.073961716	5.891099351	-2.555434361	0.01754431	0.196139981
ENSG00000144063	MALL	-1.788215967	2.197421351	-2.369501903	0.026415696	0.237665531
ENSG00000176845	METRNL	1.185828951	5.391446178	2.535578664	0.01833861	0.201326461
ENSG00000102359	SRPX2	1.137777371	5.187640478	2.523422695	0.018841298	0.204594327
ENSG00000087250	MT3	1.107174851	0.478347125	2.456422157	0.021849643	0.218400726
ENSG00000140538	NTRK3	-2.453595358	3.248495892	-2.399882191	0.024728224	0.229320664
ENSG00000051382	PIK3CB	-1.099146492	5.769794234	-2.55053274	0.017737354	0.196961129
ENSG00000109654	TRIM2	-1.454441323	4.343169651	-2.465952109	0.021396145	0.216380658
ENSG00000004399	PLXND1	1.285152429	8.772955514	2.617753439	0.015255005	0.184314112
ENSG00000047188	YTHDC2	-0.938614467	5.30110505	-2.527323369	0.018678612	0.203673449
ENSG00000122863	CHST3	-1.54923314	3.896206076	-2.433386703	0.022982827	0.222575506
ENSG00000168356	SCN11A	-1.237016714	0.987863484	-2.423998024	0.023460083	0.224489008
ENSG00000167851	CD300A	1.185956709	3.662447738	2.420280955	0.02365155	0.22576924

ENSG00000022976	ZNF839	-1.056284156	4.239684945	-2.456068841	0.021866626	0.218401797
ENSG00000158470	B4GALT5	0.909683693	5.712007203	2.546466693	0.017898992	0.197923241
ENSG00000125851	PCSK2	2.178466231	1.248596694	2.404320091	0.024490209	0.228794202
ENSG00000144445	KANSL1L	-1.274188479	3.267486798	-2.392536775	0.025126867	0.23124086
ENSG00000148834	GSTO1	0.809079906	5.037293812	2.507611934	0.019514319	0.207381752
ENSG00000173432	SAA1	-2.865503064	4.588586375	-2.479130411	0.020783397	0.213732777
ENSG00000143457	GOLPH3L	-1.155743622	4.779646892	-2.48943827	0.020315506	0.212089553
ENSG00000164733	CTSB	1.122498731	10.16319205	2.626638839	0.014952364	0.182908913
ENSG00000103056	SMPD3	2.106162322	2.067062451	2.361825342	0.02685854	0.240272149
ENSG00000123358	NR4A1	-1.449147782	5.925193163	-2.547030518	0.017876496	0.197903296
ENSG00000170624	SGCD	1.873376155	2.511076736	2.360593438	0.026930238	0.24042876
ENSG00000147454	SLC25A37	-1.210517412	5.206315698	-2.516829736	0.019119273	0.205606012
ENSG00000180879	SSR4	0.844045969	8.699860024	2.609782166	0.015531386	0.185679876
ENSG00000085741	WNT11	-1.634691398	2.017545485	-2.363045846	0.026787679	0.239774937
ENSG00000163406	SLC15A2	-1.399065889	3.003431762	-2.376606885	0.026011819	0.235294563
ENSG00000137834	SMAD6	1.227316601	4.047050461	2.437157993	0.02279366	0.222146161
ENSG00000171408	PDE7B	1.585151114	2.776033045	2.363851019	0.026741025	0.239630739
ENSG00000163220	S100A9	-2.5133462	3.079196558	-2.388800149	0.025331922	0.231329021
ENSG00000179909	ZNF154	-1.441880872	2.408865824	-2.352092702	0.027429801	0.242616127
ENSG00000165410	CFL2	1.441809168	4.02417461	2.427703644	0.023270631	0.223737422
ENSG00000168818	STX18	1.062490285	4.158618017	2.438145187	0.022744381	0.222146161
ENSG00000165804	ZNF219	1.194856571	3.772930108	2.409467229	0.024216803	0.228400188
ENSG00000184939	ZFP90	-1.275328308	3.188542321	-2.375227423	0.026089786	0.235679234
ENSG00000228696	ARL17B	-1.494400351	2.77067768	-2.357223554	0.027127267	0.241300427
ENSG00000108799	EZH1	-0.874605798	5.785488979	-2.532756166	0.018454203	0.202069019
ENSG00000129282	MRM1	1.382722355	1.877007881	2.357301945	0.027122669	0.241300427
ENSG00000127249	ATP13A4	-1.615781673	1.155654238	-2.391675459	0.025173997	0.231302424
ENSG00000170523	KRT83	-1.622782539	2.311442718	-2.345814424	0.027804205	0.244686362
ENSG00000163347	CLDN1	-2.261507389	3.612153466	-2.407360488	0.024328367	0.228499341
ENSG00000168393	DTYMK	1.104213377	5.464115595	2.51646661	0.019134694	0.205606012
ENSG00000177374	HIC1	1.376733568	1.024683148	2.397573158	0.024852905	0.229797837
ENSG00000196683	TOMM7	-1.224548361	3.976772881	-2.416059847	0.02387073	0.227278895
ENSG00000137877	SPTBN5	-2.351960945	4.818743484	-2.474231981	0.021009232	0.215251342
ENSG00000120675	DNAJC15	1.483555803	5.313235311	2.507307728	0.019527485	0.207381752
ENSG00000168005	C11orf84	1.193870026	5.13459477	2.496121995	0.020017366	0.210235942
ENSG00000204397	CARD16	1.755453123	1.835730346	2.352610917	0.027399105	0.242616127
ENSG00000126860	EVI2A	1.451637337	0.854627321	2.405284566	0.024438762	0.228794202
ENSG00000172354	GNB2	0.83737438	8.176713589	2.586227421	0.016375721	0.189073903
ENSG00000196544	C17orf59	1.018466114	0.428464954	2.428542889	0.023227921	0.223737422
ENSG00000064225	ST3GAL6	-1.510157181	4.653048643	-2.455386009	0.021899482	0.218590826
ENSG00000150630	VEGFC	1.483659779	3.360323896	2.376691173	0.026007062	0.235294563
ENSG00000118849	RARRES1	-2.429176067	4.574652016	-2.460237293	0.021667035	0.217655215
ENSG00000111684	LPCAT3	-1.514350505	5.485538517	-2.508258617	0.019486359	0.207381752
ENSG00000232653	GOLGA8N	-1.358794307	0.98133223	-2.389458017	0.025295708	0.231302424
ENSG00000103507	BCKDK	0.838139756	5.861810975	2.524484037	0.018796902	0.204395142
ENSG00000168476	REEP4	1.061377486	5.128307296	2.488704647	0.02034848	0.212231054
ENSG00000189007	ADAT2	-1.081984493	3.58784205	-2.377379723	0.025968231	0.235294563
ENSG00000171067	C11orf24	0.931416828	5.139560087	2.484574011	0.020535071	0.213343752
ENSG00000137648	TMPRSS4	-1.509478002	1.245814975	-2.367950756	0.026504633	0.238329056
ENSG00000177042	TMEM80	-1.248028491	3.211509012	-2.35472702	0.027274087	0.242057525
ENSG00000110921	MVK	0.982124016	3.940254656	2.401676266	0.024631748	0.229075308
ENSG00000164151	KIAA0947	-1.165296044	4.879873501	-2.467546131	0.021321148	0.216110133
ENSG00000078808	SDF4	0.942757852	7.753897209	2.566478239	0.017116522	0.193637601
ENSG00000185567	AHNAK2	-1.902395812	3.87166029	-2.391043398	0.025208635	0.231302424
ENSG00000106617	PRKAG2	-1.285522741	5.060075708	-2.472899349	0.021071064	0.215251342
ENSG00000151883	PARP8	1.132981718	4.941212065	2.468568365	0.021273181	0.216077398
ENSG00000163219	ARHGAP25	1.233272229	3.338040683	2.359784984	0.026977386	0.240512593
ENSG00000175604	RP11-276H1.3	1.220952154	0.931450059	2.381564455	0.025733383	0.234076819
ENSG00000134817	APLNR	1.444249259	1.205536409	2.363473769	0.026762874	0.239689647
ENSG00000175877	WBSCR28	-1.305227962	0.955011114	-2.378591254	0.025900038	0.235047713
ENSG00000186847	KRT14	-4.033398234	7.66889722	-2.559092898	0.017401503	0.19544952
ENSG00000198185	ZNF334	-1.885985954	1.720634013	-2.337107551	0.028331202	0.247106668
ENSG00000176393	RNPEP	0.848557739	6.812359851	2.541993403	0.018078404	0.19936702
ENSG00000164161	HHIP	2.082815701	1.311989877	2.352263586	0.027419676	0.242616127
ENSG00000119147	C2orf40	-3.63553128	3.021095456	-2.345510502	0.027822448	0.244709658

ENSG00000100055	CYTH4	1.459055617	4.158551663	2.408034291	0.024292634	0.228416339
ENSG00000110076	NRXN2	2.008034706	2.743170571	2.324256117	0.029125807	0.250418104
ENSG00000174827	PDZK1	-1.062320529	2.379621347	-2.314925018	0.029715486	0.253130128
ENSG00000196368	NUDT11	1.754020026	1.775132712	2.327185558	0.028942904	0.249392147
ENSG00000138796	HADH	0.833799302	6.053033078	2.514958037	0.019198882	0.20604833
ENSG00000100170	SLC5A1	-2.262235666	1.987728358	-2.322042707	0.029264706	0.250554613
ENSG00000143365	RORC	-1.964174797	1.443275984	-2.341389621	0.028070889	0.245578987
ENSG00000066185	ZMYND12	-1.377749496	1.404842368	-2.343231152	0.027959616	0.245091803
ENSG00000120071	KANSL1	-0.761929445	5.74114919	-2.500009942	0.019845813	0.208993723
ENSG00000181007	ZFP82	-0.98340374	3.54247602	-2.360208799	0.02695266	0.24042876
ENSG00000198887	SMC5	-1.027917645	5.890500059	-2.505309765	0.019614161	0.207669226
ENSG00000185504	C17orf70	0.888700801	6.094029048	2.514057494	0.019237294	0.20604833
ENSG00000164897	TMUB1	1.086025757	5.523166602	2.488278354	0.020367664	0.212231054
ENSG00000213366	GSTM2	-1.382238609	4.959276091	-2.453932898	0.021969556	0.218733694
ENSG00000019582	CD74	1.925277582	10.43012788	2.583835013	0.016463839	0.18953345
ENSG00000059728	MXD1	-1.517851309	2.918075601	-2.326442988	0.028989167	0.249653689
ENSG00000163681	SLMAP	-1.039923041	5.770084337	-2.497411476	0.019960317	0.20991778
ENSG00000185133	INPP5J	-1.378335427	2.475582827	-2.31055782	0.029995208	0.253996123
ENSG00000075914	EXOSC7	-0.893219133	4.615531885	-2.431855448	0.023060048	0.222667825
ENSG00000117528	ABCD3	-0.79736803	6.288039395	-2.517590787	0.019086992	0.205414259
ENSG00000187730	GABRD	1.624956066	1.677902547	2.329201844	0.028817626	0.24899635
ENSG00000166997	CNPY4	0.85099082	4.675948088	2.433664786	0.022968829	0.222575506
ENSG00000108292	MLLT6	-0.914551093	7.370715529	-2.543368699	0.018023067	0.199014738
ENSG00000257743	RP11-1220K2.2	-1.68426608	1.33558916	-2.346850989	0.027742068	0.244442106
ENSG00000100994	PYGB	-0.976469447	8.346933365	-2.559167346	0.017398608	0.19544952
ENSG00000128709	HOXD9	1.38358383	1.189821672	2.35151682	0.02746395	0.242781318
ENSG00000170322	NFRKB	-0.817887317	6.028322743	-2.506573123	0.019559312	0.207381752
ENSG00000105854	PON2	-0.975155973	6.813992844	-2.530657045	0.018540611	0.202732211
ENSG00000143933	CALM2	-1.209667103	5.492082165	-2.480616634	0.020715323	0.213732777
ENSG00000141384	TAF4B	-1.477879323	2.27084906	-2.303277659	0.030466868	0.255721448
ENSG00000064309	CDON	-1.372584672	4.33585633	-2.403323285	0.024543485	0.228824616
ENSG00000143294	PRCC	0.843193391	6.901548986	2.529854516	0.018573745	0.202811855
ENSG00000113396	SLC27A6	-2.347733136	2.050197409	-2.305372223	0.030330477	0.255319482
ENSG00000167755	KLK6	-1.955574724	1.451726035	-2.327721225	0.028909573	0.249392147
ENSG00000090659	CD209	1.579176023	3.043510082	2.323074842	0.029199861	0.250554613
ENSG00000131409	LRRC4B	1.46715773	0.578935275	2.385817009	0.025496733	0.232463237
ENSG00000023445	BIRC3	-1.812163251	4.319301931	-2.404054638	0.024504386	0.228794202
ENSG00000164754	RAD21	-1.107987522	6.968851211	-2.526454033	0.018714755	0.203784336
ENSG00000126603	GLIS2	1.032037912	4.169271018	2.389919284	0.025270346	0.231302424
ENSG00000015285	WAS	1.535547408	4.357310344	2.405523465	0.024426034	0.228794202
ENSG00000134326	CMPK2	-1.729929539	3.445383866	-2.342621575	0.027996404	0.245277261
ENSG00000160113	NR2F6	-1.109193169	4.327260101	-2.401102207	0.02466258	0.229075308
ENSG00000135617	PRADC1	1.134691156	3.748743524	2.356211169	0.027186718	0.241418667
ENSG00000147996	CBWD5	-1.287016179	2.530427905	-2.298008955	0.03081243	0.256895768
ENSG00000177425	PAWR	-1.63788474	4.610264157	-2.417589325	0.023791096	0.226658221
ENSG00000014824	SLC30A9	-0.788240293	6.572965787	-2.514654095	0.019211838	0.20604833
ENSG00000006282	SPATA20	0.863476038	6.440325731	2.509035694	0.01945281	0.207381752
ENSG00000138035	PNPT1	-0.862704289	5.835780165	-2.486449405	0.020450159	0.212787427
ENSG00000168906	MAT2A	-0.730239502	6.630464858	-2.513737444	0.019250962	0.20604833
ENSG00000075426	FOSL2	0.943845818	5.386004422	2.464572063	0.021461272	0.21669808
ENSG00000148604	RGR	-2.237232305	1.753461581	-2.319597201	0.029418875	0.251239482
ENSG00000139719	VPS33A	1.011680646	3.825676349	2.356300666	0.027181457	0.241418667
ENSG00000152229	PSTPIP2	-1.817849248	4.290782401	-2.392898416	0.025107102	0.23124086
ENSG00000127463	EMC1	0.873394351	7.154101681	2.524133807	0.018811541	0.204412669
ENSG00000167797	CDK2AP2	0.992731031	4.377127681	2.39616239	0.024929367	0.230233487
ENSG00000197046	SIGLEC15	2.028494306	1.807577269	2.299252867	0.030730523	0.256621945
ENSG00000173166	RAPH1	-1.332830181	3.67484769	-2.343780731	0.027926486	0.245003487
ENSG00000170365	SMAD1	0.975978401	4.284142391	2.388921505	0.025325238	0.231329021
ENSG00000144451	SPAG16	-1.409451119	3.548445584	-2.335917543	0.028403937	0.247529538
ENSG00000108465	CDK5RAP3	-1.411380222	5.999570267	-2.487271144	0.020413056	0.212542307
ENSG00000101160	CTSZ	0.973310788	8.712041633	2.546827945	0.017884575	0.197903296
ENSG00000130513	GDF15	1.329167702	2.587639121	2.290925853	0.031282639	0.259300519
ENSG00000110047	EHD1	0.878174593	5.216687432	2.450442568	0.022138719	0.219443237
ENSG00000002746	HECW1	1.353887445	1.800493601	2.297396928	0.030852803	0.257095764
ENSG00000006652	IFRD1	-1.119458196	6.674442716	-2.509004617	0.01945415	0.207381752

ENSG00000242485	MRPL20	0.795543329	6.062006937	2.488234532	0.020369637	0.212231054
ENSG00000135976	ANKRD36	-1.276827418	6.17745183	-2.489860776	0.020296538	0.212089553
ENSG00000197363	ZNF517	-1.324414608	3.819843971	-2.349299815	0.027595779	0.243589862
ENSG00000175756	AURKAIP1	1.293802877	5.853709269	2.479344444	0.020773581	0.213732777
ENSG00000188897	CTD-3088G3.8	-1.473197058	3.040020427	-2.303946156	0.030423277	0.255721448
ENSG00000072786	STK10	0.805927924	6.271227167	2.494847063	0.020073921	0.210548063
ENSG00000156689	GLYATL2	-2.803361649	2.696580768	-2.311651335	0.029924943	0.253985812
ENSG00000130830	MPP1	1.205722618	5.465094772	2.461714108	0.021596729	0.217525859
ENSG00000143641	GALNT2	0.839590772	7.387178699	2.522601237	0.018875726	0.204684879
ENSG00000155966	AFF2	1.56434676	1.812376499	2.294838641	0.031022086	0.257923223
ENSG00000076716	GPC4	1.822750999	5.912542565	2.480389375	0.020725719	0.213732777
ENSG00000087116	ADAMTS2	1.574117075	5.965370833	2.480316806	0.02072904	0.213732777
ENSG00000159189	C1QC	2.353159891	5.342260868	2.452658659	0.022031174	0.219069174
ENSG00000198563	DDX39B	-1.486793201	5.907834875	-2.479069373	0.020786197	0.213732777
ENSG00000139908	TSSK4	-1.161578298	0.960004579	-2.344042099	0.027910743	0.245003487
ENSG00000179532	DNHD1	-1.402326883	4.482914994	-2.391015955	0.02521014	0.231302424
ENSG00000172922	RNASEH2C	0.9528622	4.605246371	2.403912312	0.024511991	0.228794202
ENSG00000111181	SLC6A12	-1.810613127	2.291988025	-2.277643962	0.032182055	0.263405624
ENSG00000141040	ZNF287	1.387031961	2.400482986	2.280594378	0.031980246	0.262962548
ENSG00000163975	MFI2	-1.619055663	7.0571308	-2.50953865	0.019431124	0.207381752
ENSG00000148942	SLC5A12	1.575436078	0.629878265	2.360237694	0.026950975	0.24042876
ENSG00000145782	ATG12	-1.198727292	2.474964489	-2.27717336	0.032214352	0.263405624
ENSG00000130558	OLFM1	1.648601299	2.088473527	2.278207694	0.032143406	0.26331568
ENSG00000155016	CYP2U1	1.262821916	2.100617696	2.278074727	0.032152518	0.26331568
ENSG00000124615	MOCS1	1.257971039	3.043098807	2.295077438	0.031006248	0.257923223
ENSG00000161381	PLXDC1	1.169847464	5.376041155	2.447127876	0.022300486	0.220370597
ENSG00000132639	SNAP25	1.379239204	1.184208757	2.318466289	0.029490421	0.251485973
ENSG00000136237	RAPGEF5	-1.448307126	3.229744647	-2.300729778	0.030633532	0.25629046
ENSG00000137103	TMEM8B	-1.09181341	5.171136762	-2.432567547	0.023024107	0.222575506
ENSG00000183682	BMP8A	1.362965656	0.90957753	2.333607291	0.028545631	0.24787465
ENSG00000143458	GABPB2	-1.033079483	6.648172019	-2.492741099	0.020167666	0.211390007
ENSG00000168546	GFRA2	1.421874922	1.440966418	2.301009845	0.030615172	0.25629046
ENSG00000186716	BCR	-0.729968593	7.266940678	-2.508369857	0.019481553	0.207381752
ENSG00000112902	SEMA5A	1.784107976	3.663661637	2.327404693	0.028929264	0.249392147
ENSG00000060709	RIMBP2	1.185247908	0.478347125	2.363916039	0.026737261	0.239630739
ENSG00000136378	ADAMTS7	1.178250543	5.50379113	2.450775886	0.022122513	0.219443237
ENSG00000197380	DACT3	1.141855793	3.734941821	2.3293815	0.028806488	0.24899635
ENSG00000100302	RASD2	-1.326646786	1.924980624	-2.274758613	0.032380537	0.264351197
ENSG00000257704	PRR24	1.840568184	1.908266643	2.278641802	0.032113673	0.26331568
ENSG00000075884	ARHGAP15	1.026569761	3.43659121	2.310821025	0.029978282	0.253989861
ENSG00000164841	TMEM74	0.886254328	0.385114037	2.366188801	0.026605992	0.23896658
ENSG00000137955	RABGGTB	-0.803378158	6.066254663	-2.470917639	0.021163324	0.21563228
ENSG00000119986	AVPI1	-1.663514187	3.49819068	-2.311074368	0.029961998	0.253989039
ENSG00000144747	TMF1	-1.076442757	5.085203857	-2.420221595	0.023654619	0.22576924
ENSG00000184903	IMMP2L	-1.39411391	2.695049156	-2.271726981	0.032590283	0.265495749
ENSG00000071127	WDR1	0.770690699	8.947828513	2.528887338	0.018613751	0.203107346
ENSG00000117479	SLC19A2	-1.491957599	2.6172032	-2.269292964	0.032759578	0.266337069
ENSG00000165449	SLC16A9	1.309211031	0.773538402	2.337119916	0.028330447	0.247106668
ENSG00000109819	PPARGC1A	-1.952705756	2.368034348	-2.269458906	0.032748011	0.266337069
ENSG00000023902	PLEKHO1	1.362553221	5.88111929	2.46167253	0.021598706	0.217525859
ENSG00000172137	CALB2	-2.472278016	3.216129161	-2.294407051	0.031050728	0.257923223
ENSG00000184730	APOBR	1.592078445	2.323388287	2.261384415	0.033315196	0.268076279
ENSG00000234906	APOC2	-1.699250086	1.906197368	-2.265246739	0.033042784	0.267254803
ENSG00000169499	PLEKHA2	1.200503386	4.3037188	2.36475876	0.026688518	0.239433697
ENSG00000101353	MROH8	-1.328440397	1.931160148	-2.265708376	0.03301036	0.267254803
ENSG00000196074	SYCP2	-1.974396466	5.021387458	-2.409631294	0.024208135	0.228400188
ENSG00000105127	AKAP8	-0.744867009	6.187739127	-2.468511129	0.021275864	0.216077398
ENSG00000099219	ERMP1	-1.045594924	4.960237995	-2.406277467	0.024385903	0.22871441
ENSG00000135914	HTR2B	1.353596413	1.089019054	2.307231338	0.030209886	0.254988339
ENSG00000186204	CYP4F12	-2.673874729	1.971736592	-2.265859559	0.032999748	0.267254803
ENSG00000107719	PALD1	1.065353835	4.891301498	2.400041163	0.024719661	0.229320664
ENSG00000248540	RP11-247C2.2	-1.21271897	0.960004579	-2.319429209	0.029429493	0.251239482
ENSG00000125505	MBOAT7	0.937818456	6.200322251	2.466780483	0.02135714	0.216203152
ENSG00000104047	DTWD1	-1.667519845	2.97336726	-2.272499246	0.032536736	0.265350271
ENSG00000135643	KCNMB4	1.567714983	1.117188952	2.302756774	0.030500873	0.255775996

ENSG00000053918	KCNQ1	1.263166415	3.435952522	2.299803376	0.030694338	0.256456257
ENSG00000154645	CHODL	-1.653940587	1.617521828	-2.278493499	0.032123828	0.26331568
ENSG00000164211	STARD4	1.424006934	1.974739445	2.25953802	0.033446145	0.26857905
ENSG00000122986	HVCN1	1.288290536	2.672335932	2.258747662	0.033502342	0.268755237
ENSG00000173120	KDM2A	-0.706248332	6.97326345	-2.486123741	0.02046488	0.212799495
ENSG00000188483	IER5L	1.358058842	1.246235097	2.293500479	0.03111097	0.258286895
ENSG00000090104	RGS1	1.617297227	3.006872498	2.271510108	0.032605335	0.265495749
ENSG00000116863	ADPRHL2	1.05609139	5.235205166	2.418514994	0.02374302	0.226475212
ENSG00000196358	NTNG2	1.354048308	0.823420573	2.320169202	0.029382748	0.251180672
ENSG00000178814	OPLAH	-2.168467615	6.247233326	-2.460977351	0.021631777	0.217655215
ENSG00000167608	TMC4	-2.239230176	5.137343911	-2.408974928	0.024242831	0.228400188
ENSG00000133083	DCLK1	1.567845745	3.046298867	2.275374656	0.032338066	0.264154923
ENSG00000172893	DHCR7	-1.157915825	5.910660143	-2.450496141	0.022136114	0.219443237
ENSG00000182481	KPNA2	1.351222033	6.411826916	2.469130431	0.021246849	0.216077398
ENSG00000031691	CENPQ	-1.461225496	2.848630651	-2.26393403	0.033135141	0.2677258
ENSG00000130032	PRRG3	-1.247909231	0.901857263	-2.312826715	0.029849585	0.253985812
ENSG00000108479	GALK1	1.187281428	5.210015764	2.413935603	0.023981738	0.227920929
ENSG00000146085	MUT	-0.772725336	6.07311009	-2.454944373	0.021920758	0.218643959
ENSG00000127081	ZNF484	1.024521555	0.708164219	2.325655624	0.029038295	0.249939601
ENSG00000158856	DMTN	-1.22226931	4.548096886	-2.37100992	0.026329495	0.237161943
ENSG00000131187	F12	-1.374583101	1.674630867	-2.26678437	0.032934899	0.267163396
ENSG00000020577	SAMD4A	-1.043926399	4.910345211	-2.391355432	0.025191529	0.231302424
ENSG00000188257	PLA2G2A	-3.595699241	3.673112175	-2.302538777	0.030515115	0.255775996
ENSG00000132507	EIF5A	0.93744418	5.147084919	2.407257225	0.024333847	0.228499341
ENSG00000037757	MRI1	-0.969134434	4.882717884	-2.387894834	0.025381834	0.231550207
ENSG00000228300	C19orf24	1.065250271	4.730284459	2.376705115	0.026006275	0.235294563
ENSG00000102316	MAGED2	1.044133222	8.732255969	2.506239273	0.019573792	0.207381752
ENSG00000082497	SERTAD4	-1.710813011	3.837986148	-2.31133225	0.029945431	0.253985812
ENSG00000104722	NEFM	1.541050224	0.718043315	2.321967619	0.029269429	0.250554613
ENSG00000155974	GRIP1	-1.546267705	3.046692944	-2.265314673	0.03303801	0.267254803
ENSG00000185015	CA13	-1.433727589	3.522137562	-2.291400743	0.031250909	0.259174429
ENSG00000164761	TNFRSF11B	-1.924093071	2.2315504	-2.241197964	0.034772521	0.274040996
ENSG00000083828	ZNF586	-1.144894393	3.310221454	-2.275351519	0.03233966	0.264154923
ENSG00000146856	AGBL3	-1.414326819	1.862211483	-2.251670123	0.034009417	0.27102561
ENSG00000130005	GAMT	1.71323843	4.0204151	2.320705503	0.029348914	0.25109804
ENSG00000174370	C11orf45	1.221345934	0.873302744	2.305586543	0.030316553	0.255319482
ENSG00000162927	PUS10	-0.999698506	3.833314758	-2.307943919	0.030163781	0.254784704
ENSG00000256087	ZNF432	-0.996145834	4.086771429	-2.325164072	0.029069005	0.250066752
ENSG00000101346	POFUT1	0.750741222	5.240829256	2.403656915	0.024525642	0.228794202
ENSG00000141469	SLC14A1	-1.439449837	1.622589866	-2.255609256	0.03372634	0.26955384
ENSG00000102313	ITIH6	-2.435782614	1.908566335	-2.262115411	0.033263482	0.268076279
ENSG00000125775	SDCBP2	-1.515526918	2.637819721	-2.241729217	0.034733437	0.274040996
ENSG00000133392	MYH11	-1.991874535	4.72993409	-2.366316293	0.026598646	0.23896658
ENSG00000007384	RHBDF1	-1.157486519	6.056941326	-2.441053732	0.022599763	0.222146161
ENSG00000130720	FIBCD1	1.279172376	1.17003181	2.280413631	0.031992576	0.262962548
ENSG00000138759	FRAS1	2.25671504	2.867029024	2.248315757	0.034252174	0.271714291
ENSG00000110675	ELMOD1	1.17742614	0.543721308	2.322579749	0.029230949	0.250554613
ENSG00000176490	DIRAS1	-1.423856987	1.809493088	-2.251399075	0.034028975	0.271026783
ENSG00000198431	TXNRD1	0.836898189	6.281991031	2.448381396	0.022239182	0.220160887
ENSG00000141665	FBXO15	-1.101134869	1.267562749	-2.272984162	0.032503154	0.265214242
ENSG00000129675	ARHGEF6	1.433857984	5.396538455	2.409220835	0.024229827	0.228400188
ENSG00000185513	L3MBTL1	-1.034322823	4.291064476	-2.332003142	0.028644398	0.2484573
ENSG00000142347	MYO1F	1.411232811	6.13399785	2.441907194	0.022557489	0.222146161
ENSG00000089199	CHGB	1.296355559	0.99774258	2.288172101	0.031467208	0.260280422
ENSG00000130779	CLIP1	-0.838254013	6.803528435	-2.459706222	0.021692369	0.217770291
ENSG00000105246	EBI3	1.497179024	0.964270759	2.285442929	0.03165111	0.261014593
ENSG00000136928	GABBR2	-2.882189035	2.32294347	-2.233424301	0.035349016	0.277053648
ENSG00000164118	CEP44	-1.177688386	3.303349392	-2.257725095	0.033575176	0.268875078
ENSG00000171163	ZNF692	-1.473874493	6.037118383	-2.429419293	0.023183397	0.223721211
ENSG00000153037	SRP19	-1.225113986	1.434435164	-2.255427909	0.033739325	0.26955384
ENSG00000185624	P4HB	0.941355757	10.64254204	2.507714795	0.019509869	0.207381752
ENSG00000163064	EN1	-1.560843853	3.953807664	-2.298241827	0.030797081	0.256895768
ENSG00000160685	ZBTB7B	-1.17068928	4.536406333	-2.342272935	0.028017465	0.245324797
ENSG00000166342	NETO1	1.49724055	0.652021927	2.303137531	0.030476012	0.255721448
ENSG00000180155	LYNX1	-1.715271301	3.940992352	-2.29551065	0.030977536	0.257910196

ENSG00000071054	MAP4K4	0.791982602	8.53514299	2.484125619	0.020555421	0.213343752
ENSG00000188321	ZNF559	-1.229273057	1.268258363	-2.262209709	0.033256816	0.268076279
ENSG00000101138	CSTF1	0.889718797	4.695431462	2.352752509	0.027390724	0.242616127
ENSG00000165124	SVEP1	1.341921866	4.420307019	2.33218003	0.028633492	0.2484573
ENSG00000141012	GALNS	0.816616503	5.957648531	2.42393616	0.023463258	0.224489008
ENSG00000183960	KCNH8	-1.766741825	1.525363319	-2.255171971	0.033757658	0.26956306
ENSG00000146233	CYP39A1	-1.429062035	3.512991411	-2.26159845	0.033300047	0.268076279
ENSG00000184292	TACSTD2	-2.220900407	2.97916839	-2.236204329	0.035141859	0.276257968
ENSG00000160219	GAB3	1.497753967	1.767635575	2.22752262	0.035792467	0.27903468
ENSG00000198125	MB	-1.895962847	1.726989729	-2.228710254	0.035702824	0.27903468
ENSG00000108344	PSMD3	0.700968662	7.625171784	2.46572148	0.021407016	0.216380658
ENSG00000135605	TEC	1.555193108	1.616255172	2.234563038	0.035264028	0.276663928
ENSG00000106683	LIMK1	0.749406748	6.518408942	2.439234493	0.022690119	0.222146161
ENSG00000130988	RGN	-1.605604795	1.523493751	-2.241404932	0.034757289	0.274040996
ENSG00000007129	CEACAM21	1.264836072	1.117916236	2.262513533	0.033235348	0.268076279
ENSG00000126088	UROD	0.756127708	6.594861142	2.440512827	0.022626593	0.222146161
ENSG00000170006	TMEM154	-1.48396849	2.585920289	-2.214677877	0.036775122	0.282723392
ENSG00000124313	IQSEC2	0.954614577	4.560633217	2.335798281	0.028411236	0.247529538
ENSG00000135218	CD36	2.134780207	5.193716355	2.376511346	0.026017212	0.235294563
ENSG00000058866	DGKG	1.108596755	0.794866054	2.283331957	0.031794028	0.262016856
ENSG00000124126	PREX1	1.492560417	6.656050232	2.438116754	0.022745799	0.222146161
ENSG00000108829	LRRC59	0.853246587	7.13346728	2.44951713	0.022183774	0.219751005
ENSG00000163565	IFI16	1.263667714	6.164973648	2.422354192	0.02354458	0.225102669
ENSG00000166926	MS4A6E	1.801433915	2.605893319	2.205736759	0.037473513	0.285020304
ENSG00000196329	GIMAP5	1.650971693	2.236979502	2.201210443	0.037831625	0.286356017
ENSG00000163479	SSR2	0.71310147	9.134003549	2.473199819	0.021057108	0.215251342
ENSG00000137404	NRM	1.59827255	3.321541983	2.248766815	0.03421944	0.271639363
ENSG00000134138	MEIS2	1.138347413	4.006626328	2.282256055	0.031867096	0.262481153
ENSG00000069493	CLEC2D	-1.265330609	2.848231809	-2.212413752	0.036950848	0.283796262
ENSG00000187773	FAM69C	1.866967918	2.036062476	2.207289128	0.037351402	0.285020304
ENSG00000139910	NOVA1	1.787828319	2.212132844	2.199270617	0.037986046	0.286800124
ENSG00000130881	LRP3	1.08275998	5.647144163	2.392568405	0.025125138	0.23124086
ENSG00000187609	EXD3	-1.091915928	4.668717378	-2.327697678	0.028911037	0.249392147
ENSG00000173928	SWSAP1	-1.303880202	2.315530559	-2.198516072	0.038046266	0.286866365
ENSG00000171747	LGALS4	-1.3264739	1.670256853	-2.217015744	0.036594468	0.282025442
ENSG00000019169	MARCO	-1.806019221	2.303815721	-2.195556275	0.038283322	0.287280537
ENSG00000177854	TMEM187	-1.382650428	1.500727685	-2.224550649	0.036017687	0.280334092
ENSG00000205403	CFI	1.683945572	5.416477651	2.378968167	0.025878856	0.234991397
ENSG00000203827	NBPF16	-1.118653522	1.843148904	-2.204192647	0.037595333	0.285433685
ENSG00000189184	PCDH18	1.311159224	3.980736033	2.278067589	0.032153008	0.26331568
ENSG00000137161	CNPY3	0.881695277	6.278426278	2.415141055	0.023918685	0.227508519
ENSG00000177098	SCN4B	1.673364418	2.106805368	2.201511175	0.037807736	0.286313311
ENSG00000164764	SBSPON	-1.970939094	3.70897589	-2.258280357	0.033535609	0.268875078
ENSG00000140527	WDR93	-1.224028055	1.305078026	-2.235471429	0.035196364	0.276271211
ENSG00000106003	LFNG	1.356185896	4.362388619	2.301989791	0.030551007	0.255940124
ENSG00000107897	ACBD5	-1.142642724	4.814138066	-2.334281598	0.028504207	0.247789202
ENSG00000184258	CDR1	1.217972501	1.965429911	2.194613503	0.038359111	0.287436869
ENSG00000099812	MISP	-1.55420703	1.109443884	-2.242930488	0.034645207	0.273781946
ENSG00000185651	UBE2L3	-1.348200241	2.34345011	-2.190250689	0.038711604	0.288888928
ENSG00000011243	AKAP8L	-0.948222304	6.842735807	-2.4260182	0.023356624	0.224152166
ENSG00000174886	NDUFA11	-1.46528138	1.903652972	-2.197850044	0.038099493	0.286866365
ENSG00000169282	KCNAB1	-1.256554077	0.967231446	-2.250943488	0.034061871	0.271026783
ENSG00000156976	EIF4A2	-0.809007735	7.011894522	-2.42785902	0.023262718	0.223737422
ENSG00000198517	MAFK	-1.486705422	3.323783658	-2.222133822	0.036201785	0.280356868
ENSG00000136295	TTYH3	1.188319864	7.415043425	2.436141314	0.022844514	0.222227695
ENSG00000120656	TAF12	1.040238776	4.971233489	2.341017104	0.028093447	0.245578987
ENSG00000170448	NFXL1	-0.990861049	4.808334977	-2.327271926	0.028937527	0.249392147
ENSG00000198216	CACNA1E	1.436735367	1.194707304	2.232373073	0.035427638	0.277392745
ENSG00000134884	ARGLU1	-1.074678659	6.518752586	-2.411214789	0.024124618	0.228400188
ENSG00000165795	NDRG2	-1.70908047	7.92550981	-2.43968985	0.022667471	0.222146161
ENSG00000154059	IMPACT	-1.210170354	4.738848137	-2.32277863	0.029218457	0.250554613
ENSG00000133026	MYH10	1.128258622	7.348018057	2.432438297	0.023030627	0.222575506
ENSG00000141026	MED9	1.372776085	2.293814455	2.181423823	0.039433745	0.290631699
ENSG00000133739	LRRCC1	-0.92642011	6.118525037	-2.396728662	0.024898649	0.230085223
ENSG00000055917	PUM2	-0.7669551	6.666313468	-2.415042297	0.023923845	0.227508519

ENSG00000108733	PEX12	-1.181175399	1.189821672	-2.228081675	0.035750244	0.27903468
ENSG00000182504	CEP97	-1.042037512	3.581100559	-2.233523174	0.035341629	0.277053648
ENSG00000163444	TMEM183A	-1.064529395	4.047689626	-2.266378055	0.032963376	0.267163396
ENSG00000072422	RHOBTB1	1.218212404	3.795462253	2.253647644	0.033867038	0.270161509
ENSG00000118873	RAB3GAP2	-0.764347002	6.513161282	-2.407972778	0.024295894	0.228416339
ENSG00000140254	DUOXA1	-1.522187568	2.239537282	-2.182348072	0.039357564	0.290479554
ENSG00000080503	SMARCA2	-0.817674875	7.826651606	-2.437309642	0.022786084	0.222146161
ENSG00000164056	SPRY1	1.231137195	2.467194333	2.183254712	0.039282963	0.290338662
ENSG00000154479	CCDC173	-1.268518825	0.94847986	-2.243096895	0.034633001	0.273781946
ENSG00000141380	SS18	-0.839432848	6.659820173	-2.411403431	0.024114687	0.228400188
ENSG00000124299	PEPD	0.756350507	6.447014609	2.404538112	0.024478571	0.228794202
ENSG00000139517	LNX2	-1.140680918	3.591752955	-2.228460569	0.035721654	0.27903468
ENSG00000132970	WASF3	-1.685522988	3.056368826	-2.196339548	0.038220458	0.287220887
ENSG00000111817	DSE	1.365801115	2.705807753	2.187553054	0.038931023	0.28935369
ENSG00000114251	WNT5A	1.545419997	2.199555379	2.177173305	0.039785811	0.291513443
ENSG00000127528	KLF2	1.376291284	3.272744877	2.206990735	0.037374846	0.285020304
ENSG00000047932	GOPC	-1.088191312	4.021056377	-2.259049869	0.033480844	0.268720164
ENSG00000140379	BCL2A1	-1.381879669	1.376287849	-2.206540005	0.037410283	0.285020304
ENSG00000116685	KIAA2013	-1.075758818	2.866447802	-2.185079552	0.039133199	0.290095758
ENSG00000101146	RAE1	0.826536116	5.783997971	2.373252456	0.026201785	0.236418755
ENSG00000118292	C1orf54	1.338436556	3.538607644	2.222681832	0.036159967	0.280342685
ENSG00000132680	KIAA0907	-0.940846386	7.219888647	-2.417746961	0.023782903	0.226658221
ENSG00000170382	LRRN2	-1.395618658	2.07785163	-2.17051036	0.040343411	0.293885083
ENSG00000183520	UTP11L	0.811532113	5.789978109	2.371469566	0.026303272	0.237138085
ENSG00000185043	CIB1	0.782471639	6.913016192	2.408829821	0.024250507	0.228400188
ENSG00000149150	SLC43A1	1.306981258	4.148290774	2.264036678	0.033127911	0.2677258
ENSG00000196262	PPIA	-0.701834294	6.093184716	-2.382432694	0.025684903	0.233907027
ENSG00000144199	FAHD2B	-1.378216613	4.773915258	-2.306272066	0.030272054	0.255238475
ENSG00000107738	C10orf54	1.318176198	5.17280841	2.33498815	0.028460861	0.247609358
ENSG00000183742	MACC1	-1.405539426	0.998362031	-2.227457506	0.035797388	0.27903468
ENSG00000042062	FAM65C	1.447932138	2.667541797	2.173333936	0.040106259	0.292701078
ENSG00000110844	PRPF40B	-1.037716241	4.38468005	-2.278227404	0.032142056	0.26331568
ENSG00000135094	SDS	1.45021281	3.180988797	2.199820887	0.037942183	0.286777842
ENSG00000176853	FAM91A1	-1.098367183	4.546480506	-2.289798609	0.031358072	0.259788546
ENSG00000175550	DRAP1	0.73570858	7.137860387	2.412461343	0.02405906	0.228241057
ENSG00000196456	ZNF775	-1.161853741	1.807367517	-2.176844579	0.039813157	0.291513443
ENSG00000116525	TRIM62	1.276101102	2.446739046	2.167617906	0.04058766	0.294843045
ENSG00000164749	HNF4G	-1.386768017	1.604838286	-2.187485272	0.038936551	0.28935369
ENSG00000171729	TMEM51	1.134950868	2.727075981	2.17264284	0.040164188	0.292921992
ENSG00000110492	MDK	1.359745271	7.180308865	2.411168931	0.024127033	0.228400188
ENSG00000138593	SECISBP2L	-1.424707844	4.790758296	-2.305684657	0.03031018	0.255319482
ENSG00000198862	LTN1	-1.06654879	4.904202947	-2.311864225	0.029911281	0.253985812
ENSG00000116985	BMP8B	1.48291841	2.262355536	2.160972271	0.041153902	0.296077893
ENSG00000196418	ZNF124	-0.928786035	4.096731524	-2.251663355	0.034009905	0.27102561
ENSG00000090661	CERS4	-1.266103858	5.000472778	-2.318946399	0.029460028	0.2513634
ENSG00000160796	NBEAL2	-1.183472891	6.875593192	-2.401334855	0.02465008	0.229075308
ENSG00000172269	DPAGT1	0.7228859	5.277610925	2.337551733	0.028304097	0.247106668
ENSG00000107669	ATE1	-1.271875465	3.426539642	-2.202083123	0.03776234	0.28614523
ENSG00000141985	SH3GL1	1.010935956	6.12724657	2.375706132	0.026062705	0.23557022
ENSG00000159111	MRPL10	0.822892584	4.892283186	2.308175053	0.03014884	0.254784704
ENSG00000158806	NPM2	-1.617698357	1.793901701	-2.169730772	0.040409112	0.293954275
ENSG00000128011	LRFN1	1.108207856	1.17003181	2.209837025	0.037151763	0.284226381
ENSG00000159733	ZFYVE28	-1.291068441	3.334692608	-2.194683864	0.03835345	0.287436869
ENSG00000005243	COPZ2	1.003946642	6.966771712	2.399163074	0.024766992	0.229544521
ENSG00000131242	RAB11FIP4	-1.240303399	4.527744451	-2.279339052	0.032065969	0.26331568
ENSG00000249242	TMEM150C	1.505987057	2.604397789	2.15868561	0.041350379	0.296404202
ENSG00000166578	IQCD	-1.268784456	1.436049116	-2.187518791	0.038933817	0.28935369
ENSG00000174501	ANKRD36C	-1.277614883	5.930029899	-2.361080406	0.026901875	0.240385716
ENSG00000081019	RSBN1	-0.995028905	3.412056541	-2.196380941	0.038217139	0.287220887
ENSG00000170445	HARS	0.670463673	6.944805719	2.395450584	0.024968028	0.2304549
ENSG00000169554	ZEB2	1.265107006	3.788983937	2.221594875	0.036242955	0.280418247
ENSG00000101782	RIOK3	-0.746566097	7.226478205	-2.401548623	0.0246386	0.229075308
ENSG00000005513	SOX8	-2.626035185	2.241349383	-2.179131569	0.039623258	0.290930879
ENSG00000242372	EIF6	0.757940913	6.486454847	2.381661501	0.02572796	0.234076819
ENSG00000159086	PAXBP1	-0.944379266	5.775137876	-2.352123466	0.027427978	0.242616127

ENSG00000087237	CETP	1.390039176	1.847338922	2.157947897	0.041413946	0.296588873
ENSG00000155858	LSM11	1.231603714	2.039418014	2.153885782	0.041765547	0.297827488
ENSG00000078369	GNB1	0.817969505	8.669460357	2.422130719	0.023556088	0.225102669
ENSG00000213190	MLLT11	1.270276414	1.25032574	2.192189373	0.038554608	0.288213604
ENSG00000163357	DCST1	-1.245886486	1.263264898	-2.193387302	0.038457887	0.287902054
ENSG00000124177	CHD6	-0.830849824	6.017036592	-2.361530766	0.026875668	0.240288384
ENSG00000100603	SNW1	0.905106377	4.896069947	2.300475293	0.030650225	0.25629046
ENSG00000106077	ABHD11	-1.215630992	5.447494902	-2.335106168	0.028453627	0.247609358
ENSG00000125122	LRRC29	1.300750302	1.484317335	2.176423855	0.039848181	0.291513443
ENSG00000115935	WIPF1	0.991197394	5.001296825	2.308214895	0.030146265	0.254784704
ENSG00000134278	SPIRE1	-1.102780794	4.635466039	-2.281304357	0.031931856	0.262876573
ENSG00000171004	HS6ST2	1.342358178	1.192055075	2.193006899	0.038488577	0.287994402
ENSG00000111860	CEP85L	-1.297951193	2.075889359	-2.146616856	0.042401437	0.300018939
ENSG00000205302	SNX2	0.700310833	7.144444971	2.394228913	0.025034511	0.230932697
ENSG00000186260	MKL2	-1.032006031	3.527827315	-2.196099962	0.038239677	0.287227751
ENSG00000120963	ZNF706	-1.127169279	3.889265069	-2.218989927	0.036442544	0.281546017
ENSG00000240184	PCDHGC3	1.213879181	2.632847278	2.148073816	0.042273287	0.300018939
ENSG00000180901	KCTD2	0.958419271	4.168805797	2.241615286	0.034741815	0.274040996
ENSG00000167468	GPX4	0.830457928	8.396039377	2.413367867	0.024011487	0.228065527
ENSG00000009844	VTA1	-1.010389244	4.930500532	-2.298802371	0.030760163	0.256732831
ENSG00000118785	SPP1	2.016804126	7.238661747	2.393606434	0.025068449	0.231109893
ENSG00000117154	IGSF21	1.709310907	2.977937791	2.161030544	0.041148907	0.296077893
ENSG00000128203	ASPHD2	1.015752092	0.550252562	2.232577042	0.03541237	0.277392745
ENSG00000166037	CEP57	-0.963345192	5.202994392	-2.313104123	0.029831825	0.253983266
ENSG00000188735	TMEM120B	-0.933708388	5.055084638	-2.300354355	0.03065816	0.25629046
ENSG00000175984	DENND2C	1.103550327	3.906358453	2.218089732	0.036511748	0.281803166
ENSG00000107186	MPDZ	-1.106820058	5.984380491	-2.35061899	0.027517268	0.243115679
ENSG00000232810	TNF	-1.162916435	1.203579794	-2.184781092	0.039157658	0.290095758
ENSG00000010671	BTK	1.719816258	3.389283269	2.183856808	0.039233492	0.290246454
ENSG00000268891	AC006014.1	-1.245147758	1.27548523	-2.182623921	0.039334853	0.290479554
ENSG00000142173	COL6A2	1.288694175	11.59173289	2.438430774	0.022730143	0.222146161
ENSG00000123415	SMUG1	0.870532967	4.180868661	2.235548581	0.035190623	0.276271211
ENSG00000173548	SNX33	-1.068342059	2.358170346	-2.136248404	0.043323561	0.302814704
ENSG00000215114	UBXN2B	-0.959210022	4.019337842	-2.223647771	0.036086363	0.280342685
ENSG00000178573	MAF	1.379540575	2.969495561	2.154311782	0.041728548	0.297754729
ENSG00000163864	NMNAT3	-1.328080089	1.450426688	-2.165875754	0.040735417	0.295183138
ENSG00000147894	C9orf72	-1.216720479	2.707057738	-2.143463548	0.042679991	0.300940454
ENSG00000169047	IRS1	1.584344028	2.605472369	2.141427141	0.042860756	0.301358608
ENSG00000142156	COL6A1	1.420527056	11.41647994	2.435393839	0.02288197	0.222316401
ENSG00000116266	STXBP3	-0.722225178	6.219948691	-2.357264714	0.027124853	0.241300427
ENSG00000162627	SNX7	0.971566103	4.838826561	2.285375698	0.031655653	0.261014593
ENSG00000257950	P2RX5-TAX1BP3	-1.138938822	1.831624186	-2.143230165	0.042700673	0.300940454
ENSG00000198246	SLC29A3	1.202971286	2.719109115	2.143102097	0.042712025	0.300940454
ENSG00000054967	RELT	1.610086864	4.143858785	2.230812964	0.035544612	0.278169828
ENSG00000169951	ZNF764	-1.250820829	2.68145999	-2.13771863	0.043191718	0.302814704
ENSG00000111696	NT5DC3	-1.066993829	3.882610226	-2.206345754	0.037425565	0.285020304
ENSG00000173064	HECTD4	-0.713390586	6.730959765	-2.370234085	0.026373811	0.237424822
ENSG00000121690	DEPDC7	1.450912623	2.558813469	2.13728206	0.04323083	0.302814704
ENSG00000130193	THEM6	-1.510014669	2.418131125	-2.134865619	0.043447894	0.302814704
ENSG00000175879	HOXD8	1.30608757	0.983565633	2.196762095	0.038186584	0.287220887
ENSG00000164929	BAALC	1.821641484	2.686959609	2.135421723	0.043397854	0.302814704
ENSG00000111676	ATN1	-0.858046525	8.217309372	-2.398366662	0.024809993	0.229671739
ENSG00000063660	GPC1	1.113862269	7.103767162	2.377054115	0.025986587	0.235294563
ENSG00000112701	SENP6	-0.792586623	7.344429306	-2.382601971	0.02567546	0.233907027
ENSG00000127995	CASD1	-1.042914352	4.48920007	-2.250390597	0.034101832	0.27106983
ENSG00000100060	MFNG	1.248637034	3.248722294	2.158921059	0.04133011	0.296404202
ENSG00000140873	ADAMTS18	1.428581969	1.683638812	2.143675997	0.042661172	0.300940454
ENSG00000011295	TTC19	-0.977458008	5.334683963	-2.306793467	0.030238249	0.255090518
ENSG00000138411	HECW2	1.351197863	4.120982728	2.222424615	0.036179589	0.280342685
ENSG00000213523	SRA1	0.878487939	5.563057512	2.320045024	0.029390588	0.251180672
ENSG00000115419	GLS	-1.263939982	6.366640593	-2.352284779	0.02741842	0.242616127
ENSG00000162733	DDR2	1.585050471	5.285551351	2.303715472	0.030438313	0.255721448
ENSG00000188549	C15orf52	-1.877508373	4.062919674	-2.216251704	0.036653419	0.282166226
ENSG00000134339	SAA2	-1.460537397	1.424354669	-2.154590781	0.041704333	0.297723684
ENSG00000158220	ESYT3	-1.237748697	3.418595424	-2.165130402	0.04079878	0.29540122

ENSG00000214530	STARD10	-1.050339634	5.911903738	-2.334875985	0.028467738	0.247609358
ENSG00000168032	ENTPD3	1.303478237	1.797835716	2.132262128	0.043682857	0.302814704
ENSG00000169682	SPNS1	-1.196876822	3.575676709	-2.175274924	0.039943967	0.291652296
ENSG00000080031	PTPRH	1.406880069	1.005588899	2.180143298	0.039539512	0.290864735
ENSG00000167757	KLK11	-1.963179617	1.897192945	-2.130751807	0.043819686	0.303297172
ENSG00000087191	PSMC5	0.72053919	7.695151397	2.37910112	0.025871388	0.234991397
ENSG00000169203	RP11-231C14.4	-0.902208621	4.940530549	-2.271839827	0.032582453	0.265495749
ENSG00000172508	CARNS1	-1.330478401	1.026297099	-2.177876194	0.039727396	0.2913173
ENSG00000112796	ENPP5	-1.723700439	2.184566051	-2.11469968	0.045297915	0.308359904
ENSG00000109971	HSPA8	0.735516387	7.488884745	2.374207222	0.026147586	0.236065461
ENSG00000134061	CD180	1.115378685	1.076798721	2.170051102	0.040382104	0.293954275
ENSG00000056050	C4orf27	0.901653387	4.714958047	2.255931365	0.033703287	0.269540407
ENSG00000147234	FRMPD3	-1.206543918	1.189821672	-2.160285289	0.041212841	0.296230276
ENSG00000198908	BHLHB9	1.105060022	0.780069656	2.190523067	0.038689512	0.288888928
ENSG00000263053	RP11-1055B8.6	0.899812687	0.521698043	2.209848623	0.037150856	0.284226381
ENSG00000024862	CCDC28A	-1.135793578	4.1980832	-2.210325819	0.037113574	0.284211367
ENSG00000126217	MCF2L	-1.157425631	5.646222685	-2.312046698	0.029899575	0.253985812
ENSG00000181104	F2R	1.129455783	2.896515309	2.124507595	0.044389481	0.305489188
ENSG00000065485	PDIA5	0.98361048	6.558953373	2.347024613	0.027731673	0.244442106
ENSG00000119632	IFI27L2	1.373786737	5.578362021	2.307855125	0.030169523	0.254784704
ENSG00000151498	ACAD8	-0.80114755	4.871017276	-2.262104145	0.033264279	0.268076279
ENSG00000128346	C22orf23	-1.74939326	2.825406646	-2.123520402	0.044480171	0.30578086
ENSG00000158717	RNF166	1.095839506	3.796211655	2.181554733	0.039422946	0.290631699
ENSG00000164241	C5orf63	-1.509875824	2.036657953	-2.111772365	0.045572264	0.30966457
ENSG00000090975	PITPNM2	-0.951181409	4.639690507	-2.242439199	0.034681266	0.27387204
ENSG00000165655	ZNF503	1.294055295	2.032886761	2.107855637	0.045941673	0.310051956
ENSG00000060140	STYK1	-1.722931138	2.151313175	-2.115361383	0.045236105	0.308165684
ENSG00000196104	SPOCK3	1.253525555	0.474049275	2.208013902	0.037294514	0.285020304
ENSG00000177946	CENPBD1	-1.067587345	1.448193285	-2.13611438	0.043335598	0.302814704
ENSG00000166086	JAM3	1.245127713	4.213853655	2.206199111	0.037437106	0.285020304
ENSG00000183617	MRPL54	1.083353867	3.242607609	2.137940074	0.043171891	0.302814704
ENSG00000135119	RNFT2	-1.439317446	2.545948885	-2.105747062	0.046141655	0.310501005
ENSG00000146090	RASGEF1C	-1.535966164	1.152099187	-2.158042571	0.041405783	0.296588873
ENSG00000112679	DUSP22	-1.194448738	3.929382431	-2.18591313	0.039064959	0.289956612
ENSG00000168918	INPP5D	1.3842816	5.016670271	2.267941515	0.032853923	0.266827588
ENSG00000128815	WDFY4	1.524928617	3.954062712	2.190519873	0.038689771	0.288888928
ENSG00000174529	TMEM81	-1.025909282	0.794866054	-2.179241254	0.039614171	0.290930879
ENSG00000224689	ZNF812	1.40925729	0.736718738	2.180455317	0.039513717	0.290864735
ENSG00000063180	CA11	-1.883503324	3.904555776	-2.178914956	0.03964121	0.290930879
ENSG00000107679	PLEKHA1	-0.762487761	5.094463556	-2.268084285	0.032843944	0.266827588
ENSG00000140319	SRP14	0.818066447	6.856313333	2.346057022	0.027789651	0.244686362
ENSG00000144061	NPHP1	-1.299115438	3.664841707	-2.159064559	0.04131776	0.296404202
ENSG00000173531	MST1	-1.548304847	3.062090879	-2.121858859	0.044633187	0.306494239
ENSG00000141034	GID4	1.095660103	2.836990462	2.112497317	0.045504183	0.309495506
ENSG00000110042	DTX4	1.637344398	3.208476664	2.135997371	0.043346109	0.302814704
ENSG00000214694	ARHGEF33	1.186307862	0.955011114	2.164263855	0.040872558	0.29540122
ENSG00000052841	TTC17	-0.741806029	6.864871177	-2.343657255	0.027933926	0.245003487
ENSG00000160305	DIP2A	-0.793953865	6.71954601	-2.338659001	0.028236633	0.246693209
ENSG00000140832	MARVELD3	-1.20532196	2.780365419	-2.107946983	0.045933028	0.310051956
ENSG00000107485	GATA3	-1.892145327	3.2046481	-2.133078029	0.0436091	0.302814704
ENSG00000149925	ALDOA	0.986279749	10.06461883	2.389717601	0.025281432	0.231302424
ENSG00000196151	WDSUB1	-1.341213529	3.358756513	-2.133794147	0.043544456	0.302814704
ENSG00000132170	PPARG	1.334294929	3.083025484	2.118204568	0.044971385	0.307203307
ENSG00000110013	SIAE	-1.050720811	4.669299716	-2.235671218	0.035181498	0.276271211
ENSG00000115525	ST3GAL5	1.145622409	4.210427039	2.199946064	0.037932212	0.286777842
ENSG00000108523	RNF167	0.704432591	6.856119751	2.341120674	0.028087174	0.245578987
ENSG00000131979	GCH1	-1.725712239	2.427857896	-2.09333943	0.047334302	0.314910221
ENSG00000078295	ADCY2	-2.054354514	5.066059934	-2.257236061	0.03361006	0.268931896
ENSG00000119723	COQ6	-1.285971529	2.225852521	-2.09077746	0.047583975	0.31597129
ENSG00000150337	FCGR1A	1.485012235	2.29590352	2.093137931	0.047353897	0.314910221
ENSG00000182909	LENG9	0.902884226	0.385114037	2.198015241	0.038086285	0.286866365
ENSG00000196557	CACNA1H	1.441015566	3.088020755	2.116041357	0.045172668	0.308066256
ENSG00000196126	HLA-DRB1	1.650841528	8.55317884	2.37132121	0.026311733	0.237138085
ENSG00000197119	SLC25A29	-0.980881279	4.251322872	-2.19821264	0.038070507	0.286866365
ENSG00000145741	BTF3	0.720588127	5.953779567	2.308699449	0.030114967	0.254784704

ENSG00000164975	SNAPC3	-0.801807951	5.576599482	-2.288806059	0.031424629	0.260065325
ENSG00000181830	SLC35C1	1.125821308	2.988462504	2.113631163	0.045397884	0.30890642
ENSG00000205246	RPSAP58	-1.196135052	2.226231486	-2.089165819	0.047741639	0.316307405
ENSG00000170917	NUDT6	-1.155651109	1.354960197	-2.127785572	0.044089535	0.304359391
ENSG00000160813	PPP1R35	1.25450974	1.927509503	2.095571933	0.047117694	0.314322457
ENSG00000173214	KIAA1919	1.305275167	3.192034644	2.124585551	0.044382327	0.305489188
ENSG00000114127	XRN1	-1.086728828	4.477334534	-2.216302213	0.036649519	0.282166226
ENSG00000231389	HLA-DPA1	1.613939627	8.208110507	2.364974514	0.026676052	0.239433697
ENSG00000099817	POLR2E	0.932097848	7.132927284	2.343721493	0.027930055	0.245003487
ENSG00000143514	TP53BP2	-1.234937493	6.706682174	-2.33073757	0.028722541	0.24885996
ENSG00000104894	CD37	1.154537182	4.894259187	2.247941103	0.034279386	0.271792746
ENSG00000107821	KAZALD1	1.376376869	3.304378743	2.125087744	0.044336262	0.305489188
ENSG00000014914	MTMR11	-1.556882434	4.67448905	-2.225988341	0.035908577	0.279623562
ENSG00000124766	SOX4	-0.849708395	5.563298045	-2.285900152	0.031620232	0.261014593
ENSG00000130653	PNPLA7	-1.66155257	4.739712164	-2.226988058	0.035832882	0.27917267
ENSG00000143341	HMCN1	1.717177038	5.64077815	2.289119109	0.031403623	0.260028628
ENSG00000149418	ST14	-1.763861135	7.448266811	-2.346797143	0.027745293	0.244442106
ENSG00000023171	GRAMD1B	1.828696373	2.618845869	2.103174618	0.046386687	0.311223889
ENSG00000171227	TMEM37	1.11596525	0.736718738	2.166955487	0.040643785	0.294977625
ENSG00000112877	CEP72	-1.31368265	3.061238173	-2.111898065	0.045560453	0.30966457
ENSG00000103355	PRSS33	-1.64780263	1.260204836	-2.129643219	0.043920364	0.303458577
ENSG00000128165	ADM2	-1.401348105	2.838440286	-2.095374282	0.047136835	0.314322457
ENSG00000198873	GRK5	-1.404821436	5.035632308	-2.24625351	0.034402201	0.272491137
ENSG00000086730	LAT2	1.010741145	4.66768175	2.223488719	0.036098473	0.280342685
ENSG00000171862	PTEN	-1.275221246	2.804421462	-2.092943962	0.047372765	0.314910221
ENSG00000120210	INSL6	1.357696014	0.740809381	2.162496537	0.041023401	0.295946515
ENSG00000164073	MFSD8	-1.189464001	3.669114085	-2.143896828	0.042641619	0.300940454
ENSG00000169246	NPIPB3	-1.058824271	2.074503787	-2.080417396	0.04860566	0.319101763
ENSG00000136770	DNAJC1	0.800004998	6.469797308	2.317671721	0.029540783	0.251778614
ENSG00000137757	CASP5	1.1718457	0.651750794	2.169820283	0.040401563	0.293954275
ENSG00000167618	LAIR2	1.440860068	1.582017229	2.103108432	0.046393007	0.311223889
ENSG00000123338	NCKAP1L	1.630847568	5.263035935	2.263241864	0.033183935	0.267982048
ENSG00000173065	FAM222B	-1.092868185	3.156613944	-2.106951099	0.046027366	0.310495576
ENSG00000241484	ARHGAP8	-1.211048113	1.862954283	-2.089750092	0.047684427	0.316256796
ENSG00000126233	SLURP1	-1.494265426	1.150880288	-2.13379356	0.043544509	0.302814704
ENSG00000130021	HDHD1	1.082303692	2.742595747	2.086102514	0.048042604	0.317204501
ENSG00000105219	CNTD2	-1.124638311	1.146470755	-2.130765077	0.043818482	0.303297172
ENSG00000110429	FBXO3	-0.880235394	5.141327577	-2.24961355	0.034158066	0.271379349
ENSG00000090621	PABPC4	0.716123955	7.751673903	2.344339287	0.027892852	0.245003487
ENSG00000253719	ATXN7L3B	1.286316241	1.91139804	2.08163523	0.048484551	0.318503349
ENSG00000155367	PPM1J	-1.536507136	2.257290107	-2.087899863	0.047865812	0.316370032
ENSG00000213096	ZNF254	-1.202562542	2.255645942	-2.074412384	0.049206797	0.321442067
ENSG00000165457	FOLR2	1.727957952	3.576636111	2.133766537	0.043546946	0.302814704
ENSG00000174567	GOLT1A	-1.568348244	1.128538133	-2.129951606	0.043892336	0.303398524
ENSG00000126247	CAPNS1	0.70658791	8.715562573	2.356222827	0.027186032	0.241418667
ENSG00000167641	PPP1R14A	-1.463023544	3.957913042	-2.155174909	0.041653675	0.297698399
ENSG00000072506	HSD17B10	0.727988744	7.207459763	2.329469582	0.028801028	0.24899635
ENSG00000198502	HLA-DRB5	1.629118163	6.530761503	2.31137855	0.029942457	0.253985812
ENSG00000152475	ZNF837	-1.093866113	0.916653661	-2.144528354	0.042585745	0.300725891
ENSG00000177225	PDDC1	-0.834573643	7.014894794	-2.323594367	0.029167271	0.250532677
ENSG00000120251	GRIA2	-1.697184369	1.445232599	-2.116002996	0.045176245	0.308066256
ENSG00000145730	PAM	-1.290144554	9.330936837	-2.359344526	0.027003105	0.24060518
ENSG00000183060	LYSMD4	-1.191130025	1.957908973	-2.072003108	0.04944984	0.322571504
ENSG00000179698	KIAA1875	-1.390170972	3.339391341	-2.108200359	0.045909053	0.310051956
ENSG00000139219	COL2A1	-3.537777911	5.378091729	-2.248684419	0.034225417	0.271639363
ENSG00000123104	ITPR2	-1.259064453	6.414681009	-2.303151841	0.030475078	0.255721448
ENSG00000082126	MPP4	-1.159417621	0.966535832	-2.137605481	0.043201851	0.302814704
ENSG00000132424	PNISR	-0.623558927	7.497441775	-2.329441114	0.028802793	0.24899635
ENSG00000162337	LRP5	-1.340689427	7.2454955	-2.323533835	0.029171066	0.250532677
ENSG00000145649	GZMA	1.598630421	1.453980047	2.092828055	0.047384044	0.314910221
ENSG00000146250	PRSS35	1.056585159	0.680305314	2.153050894	0.041838144	0.297994698
ENSG00000132535	DLG4	0.945677317	5.634573272	2.266466905	0.032957147	0.267163396
ENSG00000176049	JAKMIP2	1.6156784	1.595101999	2.08330408	0.048319029	0.31829298
ENSG00000173276	ZBTB21	-1.245687495	1.213382727	-2.107947132	0.045933013	0.310051956
ENSG00000182871	COL18A1	1.311490824	9.010442074	2.34919566	0.027601987	0.243589862

ENSG00000088827	SIGLEC1	1.540882221	5.004369322	2.227875348	0.035765822	0.27903468
ENSG00000004799	PDK4	1.307852618	3.123250509	2.085999936	0.048052712	0.317204501
ENSG00000153879	CEBPG	-1.271501106	2.787903279	-2.069951771	0.04965762	0.323176157
ENSG00000138166	DUSP5	1.214747988	1.462989684	2.089023905	0.047755544	0.316307405
ENSG00000106665	CLIP2	1.068530331	6.157312968	2.286773607	0.031561322	0.260921337
ENSG00000103522	IL21R	1.302632203	1.78042695	2.06916794	0.04973722	0.323291928
ENSG00000138795	LEF1	1.499546885	4.839607539	2.210572886	0.037094285	0.284202355
ENSG00000142208	AKT1	0.739846577	7.952292405	2.331420533	0.028680347	0.248631673
ENSG00000132688	NES	2.01047092	4.988946182	2.218648226	0.036468799	0.281610198
ENSG00000136908	DPM2	0.953513646	5.006871426	2.222472294	0.036175951	0.280342685
ENSG00000165905	GYLTL1B	-1.949602078	4.235406253	-2.161440743	0.041113754	0.296060539
ENSG00000141425	RPRD1A	-1.045789014	4.953527058	-2.219748145	0.036384347	0.281234875
ENSG00000158186	MRAS	-1.020668603	5.674311327	-2.260272861	0.033393973	0.268530104
ENSG00000198625	MDM4	-0.947447382	5.726646471	-2.261593698	0.033300383	0.268076279
ENSG00000128534	NAA38	-1.308555873	4.038988078	-2.141783028	0.042829115	0.301358608
ENSG00000137801	THBS1	-1.561811819	7.773690475	-2.322178379	0.029256175	0.250554613
ENSG00000168675	LDLRAD4	-1.39518618	3.168151815	-2.079473199	0.048699742	0.319101763
ENSG00000144736	SHQ1	-0.883849121	3.56206855	-2.104399834	0.046269837	0.311195892
ENSG00000103485	QPRT	1.192736179	4.586259562	2.186146189	0.039045899	0.289952299
ENSG00000077092	RARB	1.556711019	3.216061183	2.082144825	0.048433954	0.318503349
ENSG00000164736	SOX17	1.131939343	0.708164219	2.136334051	0.043315871	0.302814704
ENSG00000156103	MMP16	-1.397690218	1.539085138	-2.078578293	0.048789064	0.319378055
ENSG00000170412	GPRC5C	1.630944153	4.374680863	2.164758599	0.040830421	0.29540122
ENSG00000047346	FAM214A	-1.088830861	3.516548877	-2.098511703	0.046833817	0.313116051
ENSG00000063601	MTMR1	-0.945979119	4.597110186	-2.183312739	0.039278193	0.290338662
ENSG00000124253	PCK1	1.384140361	1.083529304	2.106685802	0.046052527	0.310495576
ENSG00000143632	ACTA1	-1.186423531	1.232134313	-2.094321994	0.04723886	0.3147452
ENSG00000073711	PPP2R3A	-1.058662369	3.966296758	-2.133776695	0.04354603	0.302814704
ENSG00000123064	DDX54	0.717884697	6.764414072	2.295421968	0.030983412	0.257910196
ENSG00000155008	APOOL	1.163588119	1.081792186	2.103273215	0.046377274	0.311223889
ENSG00000114770	ABCC5	-0.79064324	6.744235424	-2.294489268	0.03104527	0.257923223
ENSG00000009950	MLXIPL	-1.197867121	3.31152897	-2.082936643	0.048355429	0.318399092
ENSG00000179222	MAGED1	1.076128124	8.953816876	2.333917203	0.028526585	0.24784643
ENSG00000123684	LPGAT1	-1.48907647	4.09613177	-2.141352407	0.042867402	0.301358608
ENSG00000122735	DNAI1	-1.100235292	0.844748225	-2.120247935	0.044781992	0.307065891
ENSG00000123989	CHPF	0.885908017	6.387490437	2.280511987	0.031985866	0.262962548
ENSG00000147533	GOLGA7	1.094008036	4.523532539	2.175786653	0.039901278	0.291611998
ENSG00000153208	MERTK	1.03074314	5.175402996	2.22193256	0.036217154	0.280356868
ENSG00000267114	CTB-129P6.11	0.980556598	0.428464954	2.148165408	0.042265242	0.300018939
ENSG00000110888	CAPRIN2	-0.827513058	5.302164624	-2.227478794	0.035795779	0.27903468
ENSG00000089723	OTUB2	1.135449734	0.916653661	2.109361781	0.045799304	0.310051956
ENSG00000198783	ZNF830	0.91271658	0.521698043	2.141510839	0.042853312	0.301358608
ENSG00000141401	IMPA2	-1.45068345	5.649550442	-2.246486058	0.034385254	0.272491137
ENSG00000017621	MAGIX	-1.078772149	3.244948009	-2.070841782	0.049567375	0.322856656
ENSG00000163807	KIAA1143	-0.958225246	3.41873838	-2.081549644	0.048493053	0.318503349
ENSG00000198825	INPP5F	-1.04550545	4.368463077	-2.155058841	0.041663736	0.297698399
ENSG00000128928	IVD	0.961377817	6.005112126	2.259864789	0.033422936	0.268530104
ENSG00000101445	PPP1R16B	1.347782274	1.348428944	2.074525965	0.049195365	0.321442067
ENSG00000123342	MMP19	1.548373967	3.199251047	2.069533527	0.049700079	0.323184998
ENSG00000144891	AGTR1	1.193988633	0.686836567	2.123409977	0.044490326	0.30578086
ENSG00000163803	PLB1	1.282338348	3.271152609	2.070960718	0.049555327	0.322856656
ENSG00000083223	ZCCHC6	-0.788447899	5.875182942	-2.251038704	0.034054993	0.271026783
ENSG00000101230	ISM1	-1.630560855	3.692561322	-2.096673061	0.047011184	0.314028307
ENSG00000041982	TNC	1.531434355	9.55455065	2.329801303	0.028780476	0.24899635
ENSG00000170043	TRAPPC1	0.775500918	5.699089231	2.242891218	0.034648088	0.273781946
ENSG00000131238	PPT1	0.648047481	7.180381463	2.293128978	0.031135687	0.258355405
ENSG00000141579	ZNF750	-1.327510313	1.278061296	-2.074796785	0.049168118	0.321442067
ENSG00000181626	ANKRD62	1.002910342	0.801397307	2.111392437	0.04560798	0.30966457
ENSG00000173848	NET1	-1.327950256	6.181310749	-2.259990216	0.033414032	0.268530104
ENSG00000112941	PAPD7	-0.912400223	5.260745865	-2.215098423	0.036742566	0.282611569
ENSG00000085063	CD59	-1.068570383	8.417421303	-2.312109689	0.029895535	0.253985812
ENSG00000172725	CORO1B	0.816235147	7.023523642	2.285798314	0.031627108	0.261014593
ENSG00000064601	CTSA	0.910095457	7.945998641	2.304316619	0.030399144	0.255721448
ENSG00000155366	RHOC	0.928499939	6.796890709	2.277386438	0.032199725	0.263405624
ENSG00000197020	ZNF100	-1.07721148	3.398339024	-2.06825297	0.049830281	0.323494803

ENSG00000255423	EBLN2	-1.063680967	0.794866054	-2.105923832	0.04612486	0.310501005
ENSG00000131373	HACL1	-0.90606516	4.88491706	-2.184466723	0.039183436	0.290149739
ENSG00000109113	RAB34	0.764664185	7.172292257	2.285373886	0.031655775	0.261014593
ENSG00000059804	SLC2A3	1.09245504	4.96667596	2.188862108	0.038824408	0.289279168
ENSG00000180855	ZNF443	-1.343246757	1.250202391	-2.066972918	0.049960736	0.323774874
ENSG00000125170	DOK4	0.807803318	4.830300085	2.176168627	0.039869441	0.291515097
ENSG00000115194	SLC30A3	1.162015545	0.715391087	2.106535667	0.046066771	0.310495576
ENSG00000002726	AOC1	-1.234216653	0.933683462	-2.088164198	0.04783986	0.316370032
ENSG00000174326	SLC16A11	-1.236593295	1.009886749	-2.088491975	0.047807698	0.316370032
ENSG00000157870	FAM213B	0.996698348	3.990438968	2.108327161	0.04589706	0.310051956
ENSG00000167977	KCTD5	0.955461647	4.937274386	2.180358795	0.039521695	0.290864735
ENSG00000163945	UVSSA	-1.136922856	4.174304325	-2.118589474	0.044935654	0.307203307
ENSG00000116830	TTF2	-0.996043915	5.354389666	-2.20701256	0.03737313	0.285020304
ENSG00000010610	CD4	1.602558852	5.28261936	2.205323424	0.037506087	0.285048531
ENSG00000131503	ANKHD1	-1.034631924	1.124447489	-2.071365001	0.049514391	0.32277952
ENSG00000173588	CCDC41	-0.864210849	4.354465251	-2.13219817	0.043688644	0.302814704
ENSG00000049246	PER3	-1.468405296	4.050277538	-2.105693656	0.04614673	0.310501005
ENSG00000179294	C17orf96	0.859748812	0.385114037	2.12305205	0.044523255	0.305873203
ENSG00000148737	TCF7L2	-0.950963076	5.183145499	-2.192634392	0.038518651	0.288082059
ENSG00000197265	GTF2E2	0.736763114	5.451110574	2.211563868	0.037017007	0.284147189
ENSG00000122912	SLC25A16	-0.718063441	5.710565671	-2.223162317	0.036123337	0.280342685
ENSG00000205238	SPDYE2	-0.999869444	4.121814832	-2.107924483	0.045935157	0.310051956
ENSG00000106477	CEP41	0.837101696	3.973521275	2.097136792	0.046966393	0.313862724
ENSG00000181666	HKR1	-1.042174499	4.829054124	-2.164054371	0.040890412	0.29540122
ENSG00000150867	PIP4K2A	0.936463792	4.825046809	2.164666732	0.040838242	0.29540122
ENSG00000167232	ZNF91	-0.978036092	3.868981945	-2.087975982	0.047858338	0.316370032
ENSG00000112335	SNX3	0.772201972	7.135090648	2.269822838	0.032722655	0.266312856
ENSG00000167083	GNGT2	1.110036423	0.801397307	2.083721204	0.048277736	0.318154537
ENSG00000213339	QTRT1	-0.955667706	6.532502471	-2.250580721	0.034088086	0.27106983
ENSG00000162437	RAVER2	-1.02249433	4.362205412	-2.126260703	0.044228839	0.304918588
ENSG00000118640	VAMP8	-1.335498527	5.140762513	-2.185113279	0.039130436	0.290095758
ENSG00000100815	TRIP11	-0.737458809	5.068593243	-2.177832876	0.039730994	0.2913173
ENSG00000100207	TCF20	-0.887297825	4.670449568	-2.14630053	0.042429306	0.300026245
ENSG00000158019	BRE	0.920943574	5.140767047	2.182034233	0.039383417	0.290533707
ENSG00000164808	SPIDR	-0.744293727	6.770541021	-2.254867861	0.033779454	0.269599905
ENSG00000075303	SLC25A40	-0.822771395	4.594745779	-2.140339625	0.042957572	0.301857264
ENSG00000196123	KIAA0895L	-1.172761938	4.074281806	-2.09518286	0.04715538	0.314322457
ENSG00000115977	AAK1	0.813317747	6.342968061	2.240521839	0.034822321	0.2742957
ENSG00000121671	CRY2	-1.008548317	4.551776568	-2.134713683	0.043461575	0.302814704
ENSG00000080709	KCNN2	1.093145496	0.780069656	2.08184348	0.048463868	0.318503349
ENSG00000188643	S100A16	-1.584366345	5.717355829	-2.213549247	0.036862624	0.283257313
ENSG00000145283	SLC10A6	-1.111681149	0.794866054	-2.079539533	0.048693127	0.319101763
ENSG00000162302	RPS6KA4	0.702637984	6.288206756	2.236992711	0.035083313	0.275935969
ENSG00000104783	KCNN4	-1.267223654	5.79190583	-2.21607994	0.036666684	0.282166226
ENSG00000186106	ANKRD46	-0.885091382	4.363242204	-2.116652018	0.045115765	0.307921475
ENSG00000101825	MXRA5	1.638504724	5.912834241	2.220908656	0.036295436	0.280685899
ENSG00000137710	RDX	-0.847431167	5.440684236	-2.19380507	0.038424208	0.28778723
ENSG00000174775	HRAS	0.762067666	5.890737207	2.217739373	0.036538714	0.281812744
ENSG00000171109	MFN1	-0.673828926	5.507750131	-2.197613225	0.038118436	0.286866365
ENSG00000185627	PSMD13	0.581034503	7.516862522	2.266655297	0.032943943	0.267163396
ENSG00000196116	TDRD7	-0.883480194	4.160374733	-2.096313925	0.047045899	0.314126469
ENSG00000173950	XXYLT1	1.025298027	3.793384985	2.070038726	0.049648796	0.323176157
ENSG00000136104	RNASEH2B	0.941965486	5.587135244	2.19916962	0.037994101	0.286800124
ENSG00000144810	COL8A1	-2.04957893	4.642968504	-2.130309806	0.043859802	0.30330725
ENSG00000171045	TSNARE1	-1.22077542	5.135970386	-2.164335636	0.040866442	0.29540122
ENSG00000117143	UAP1	-0.84702136	6.755191781	-2.242832329	0.034652409	0.273781946
ENSG00000155158	TTC39B	-0.801000839	4.854714992	-2.149574907	0.04214162	0.299476521
ENSG00000213983	AP1G2	-0.94038623	5.518557323	-2.188840058	0.038826202	0.289279168
ENSG00000160439	RDH13	-1.020287821	4.71546191	-2.134008382	0.043525133	0.302814704
ENSG00000138964	PARVG	1.424692286	5.107257894	2.167332396	0.040611842	0.29488219
ENSG00000204764	RANBP17	-1.273270369	3.929203353	-2.07226141	0.049423732	0.322571504
ENSG00000067955	CBFB	0.855617503	5.099645337	2.162286787	0.041041337	0.295946515
ENSG00000141013	GAS8	-1.17997746	4.143846993	-2.081953407	0.048452954	0.318503349
ENSG00000181135	ZNF707	-1.038035322	4.364612076	-2.099690008	0.046720466	0.313102489
ENSG00000137486	ARRB1	0.885570731	4.740089787	2.132443961	0.04366641	0.302814704

ENSG00000163539	CLASP2	-0.745415191	5.725718563	-2.195042863	0.038324578	0.287436869
ENSG00000116786	PLEKHM2	0.742781238	6.891869867	2.238183482	0.034995053	0.275379825
ENSG00000184465	WDR27	-1.37475799	5.088907212	-2.156882811	0.041505876	0.297111637
ENSG00000121390	PSPC1	-0.772099212	5.835690199	-2.199096111	0.037999966	0.286800124
ENSG00000204564	C6orf136	-0.928002407	4.284864138	-2.093411186	0.047327326	0.314910221
ENSG00000056972	TRAF3IP2	-0.943846535	5.143143708	-2.158741364	0.041345578	0.296404202
ENSG00000157514	TSC22D3	1.037187634	4.723051065	2.127788241	0.044089292	0.304359391
ENSG00000009780	FAM76A	-0.877829454	4.387516104	-2.098502473	0.046834706	0.313116051
ENSG00000128191	DGCR8	-0.589840086	6.140278375	-2.210649117	0.037088335	0.284202355
ENSG00000121210	KIAA0922	-0.796231018	6.019481005	-2.205737129	0.037473484	0.285020304
ENSG00000173875	ZNF791	-0.824477272	5.985872207	-2.202937331	0.037694633	0.285870705
ENSG00000010295	IFFO1	1.129801792	4.385351402	2.098988754	0.046787896	0.313116051
ENSG00000187955	COL14A1	2.17871002	6.711710193	2.228650466	0.035707332	0.27903468
ENSG00000197283	SYNGAP1	-1.044262729	5.133863281	-2.150768148	0.04203722	0.298869965
ENSG00000225485	ARHGAP23	0.752469851	6.145138382	2.20674063	0.037394506	0.285020304
ENSG00000129933	MAU2	-0.657761397	6.45893355	-2.21760698	0.036548909	0.281812744
ENSG00000047578	KIAA0556	-0.825890089	4.762541842	-2.121375751	0.044677766	0.306666156
ENSG00000128591	FLNC	1.655375214	5.618889576	2.176394367	0.039850636	0.291513443
ENSG00000206560	ANKRD28	-1.165707549	5.752033775	-2.184781604	0.039157616	0.290095758
ENSG00000068079	IFI35	0.933638416	5.113777047	2.145022485	0.042542074	0.300552758
ENSG00000181019	NQO1	1.159880992	5.686131722	2.179153498	0.039621442	0.290930879
ENSG00000127955	GNAI1	1.087076238	5.296605163	2.154580518	0.041705223	0.297723684
ENSG00000164638	SLC29A4	1.288436352	4.208936531	2.067830336	0.049873319	0.323593153
ENSG00000102710	SUPT20H	-0.673119262	6.3731561	-2.206490643	0.037414166	0.285020304
ENSG00000175274	TP53I11	0.990890957	6.352138238	2.205228688	0.037513557	0.285048531
ENSG00000108666	C17orf75	-0.906783365	4.391751197	-2.083843294	0.048265656	0.318154537
ENSG00000172270	BSG	0.785345323	8.938711386	2.257575785	0.033585823	0.268875078
ENSG00000104774	MAN2B1	0.844729806	7.951267756	2.243092042	0.034633357	0.273781946
ENSG00000152558	TMEM123	-1.119065517	6.603388799	-2.211361735	0.037032758	0.284147189
ENSG00000096093	EFHC1	-1.075636179	4.474852389	-2.08683407	0.047970576	0.316928971
ENSG00000092330	TINF2	0.71523341	5.297323047	2.14729814	0.042341469	0.300018939
ENSG00000091986	CCDC80	1.725101273	7.166588053	2.22388839	0.036068049	0.280342685
ENSG00000022267	FHL1	1.624286929	4.694962917	2.103305066	0.046374234	0.311223889
ENSG00000169896	ITGAM	1.533302179	4.687486917	2.103621742	0.046344014	0.311223889
ENSG00000023191	RNH1	0.629976365	7.987423126	2.239321334	0.034910904	0.274855489
ENSG00000205268	PDE7A	-0.968447089	5.435586277	-2.155341164	0.041639267	0.297698399
ENSG00000123106	CCDC91	-1.011934607	5.087043522	-2.132160149	0.043692084	0.302814704
ENSG00000167771	RCOR2	1.839060911	4.448497562	2.080035422	0.048643701	0.319101763
ENSG00000000971	CFH	1.285147158	6.33867352	2.195791214	0.038264456	0.287276357
ENSG00000121741	ZMYM2	-0.884026327	6.602260859	-2.202020548	0.037767304	0.28614523
ENSG00000156467	UQCRB	-1.080588052	4.894120625	-2.110976708	0.045647091	0.309796066
ENSG00000142910	TINAGL1	-1.84719468	5.133085415	-2.131673941	0.043736099	0.302985927
ENSG00000135446	CDK4	0.689362481	5.73583527	2.166455359	0.040686205	0.29514898
ENSG00000122678	POLM	-0.741232144	5.117442156	-2.127074719	0.044154426	0.304539381
ENSG00000183853	KIRREL	1.04800615	5.799359305	2.165813713	0.040740688	0.295183138
ENSG00000080189	SLC35C2	0.737605221	6.695772857	2.200596579	0.03788043	0.28658719
ENSG00000175054	ATR	-0.844310731	5.973637066	-2.171609685	0.040250929	0.293347576
ENSG00000141753	IGFBP4	1.247225685	6.939012096	2.205886869	0.037461689	0.285020304
ENSG00000102878	HSF4	-1.411658096	5.577704018	-2.147133786	0.042355929	0.300018939
ENSG00000134996	OSTF1	0.639089812	6.46908681	2.189498564	0.038772667	0.289154907
ENSG00000197170	PSMD12	0.732141985	5.612166663	2.150928807	0.042023181	0.298869965
ENSG00000164933	SLC25A32	-0.935662736	4.506982544	-2.068698033	0.049784994	0.323468467
ENSG00000156970	BUB1B	1.08772628	4.752163938	2.093008084	0.047366527	0.314910221
ENSG00000130723	PRRC2B	-0.787972398	8.021384844	-2.222495496	0.036174181	0.280342685
ENSG00000077097	TOP2B	-0.668119932	8.112430596	-2.22306719	0.036130586	0.280342685
ENSG00000101997	CCDC22	0.633745528	5.380545332	2.133496686	0.043571297	0.302814704
ENSG00000149923	PPP4C	0.804831486	7.186216194	2.204125654	0.037600626	0.285433685
ENSG00000196924	FLNA	0.808336897	11.58400955	2.257750355	0.033573375	0.268875078
ENSG00000077721	UBE2A	0.718292683	5.917025745	2.161517935	0.041107142	0.296060539
ENSG00000142279	WTIP	-0.928309753	5.009359163	-2.106021684	0.046115565	0.310501005
ENSG00000116288	PARK7	0.762252132	6.771834385	2.190164574	0.038718591	0.288888928
ENSG00000166226	CCT2	0.695373971	7.860271054	2.210592343	0.037092766	0.284202355
ENSG00000143367	TUFT1	-1.027971784	5.034197673	-2.09894071	0.046792519	0.313116051
ENSG00000212907	MT-ND4L	-1.135965421	5.94166888	-2.151132197	0.042005414	0.298869965
ENSG00000102393	GLA	1.014199878	5.108361827	2.104056414	0.046302563	0.311223889

ENSG00000168090	COPS6	0.738644291	7.636888507	2.203040648	0.037686451	0.285870705
ENSG00000159842	ABR	0.888573118	6.416985817	2.168380481	0.040523137	0.29451067
ENSG00000088367	EPB41L1	-0.940553914	6.281694264	-2.161967132	0.041068684	0.296007683
ENSG00000180448	HMHA1	1.110216967	5.386979406	2.118537078	0.044940517	0.307203307
ENSG00000087842	PIR	1.149731798	5.482286948	2.124558626	0.044384798	0.305489188
ENSG00000071564	TCF3	0.782270602	7.151768548	2.187359026	0.038946848	0.28935369
ENSG00000109184	DCUN1D4	-1.015124509	4.952082073	-2.085702886	0.048081992	0.317264313
ENSG00000071794	HLTF	-0.980939834	5.677617708	-2.132996929	0.043616427	0.302814704
ENSG00000137992	DBT	-0.599836929	5.413248411	-2.117978	0.044992429	0.307213315
ENSG00000115392	FANCL	-0.999382157	5.009535388	-2.08987908	0.047671804	0.316256796
ENSG00000167114	SLC27A4	0.781888297	5.493141029	2.120782447	0.044732569	0.306908062
ENSG00000078269	SYNJ2	-0.92142075	5.392694883	-2.115215301	0.045249744	0.308165684
ENSG00000003402	CFLAR	-0.795296948	6.329941943	-2.159664985	0.041266125	0.296404202
ENSG00000160299	PCNT	-0.859980928	6.99499491	-2.178951241	0.039638202	0.290930879
ENSG00000130177	CDC16	-0.774404416	7.28891103	-2.187702425	0.038918844	0.28935369
ENSG00000108528	SLC25A11	0.61804878	6.193804344	2.153190924	0.04182596	0.297994698
ENSG00000125912	NCLN	0.824425797	6.77309466	2.172527471	0.040173866	0.292921992
ENSG00000101199	ARFGAP1	0.765542266	6.949086853	2.176713589	0.039824058	0.291513443
ENSG00000166441	RPL27A	0.684174254	5.5361103	2.118542609	0.044940003	0.307203307
ENSG00000142627	EPHA2	1.517097438	5.77659465	2.130480924	0.043844267	0.30330725
ENSG00000152583	SPARCL1	1.263115688	7.416112625	2.183887277	0.03923099	0.290246454
ENSG00000100242	SUN2	-0.891150852	7.365874087	-2.182370861	0.039355687	0.290479554
ENSG00000006744	ELAC2	0.620633378	7.134367221	2.175379538	0.039935237	0.291652296
ENSG00000175467	SART1	0.628276246	7.71572325	2.188266954	0.038872847	0.28935369
ENSG00000158941	CCAR2	0.787947972	6.195842978	2.146600663	0.042402863	0.300018939
ENSG00000166825	ANPEP	1.568404679	6.244049474	2.147240751	0.042346518	0.300018939
ENSG00000112159	MDN1	-0.799094653	6.993215202	-2.169475318	0.040430661	0.293974747
ENSG00000010270	STARD3NL	0.797729975	5.798871215	2.118973758	0.044900007	0.307203307
ENSG00000071205	ARHGAP10	1.037336743	5.018154957	2.068429471	0.049812317	0.323494803
ENSG00000143624	INTS3	-0.690214836	8.030765403	-2.18023592	0.039531853	0.290864735
ENSG00000143515	ATP8B2	1.137654745	7.227905431	2.162406158	0.041031129	0.295946515
ENSG00000105355	PLIN3	0.917148432	6.97062367	2.156352173	0.041551746	0.297304354
ENSG00000084636	COL16A1	0.985575922	9.597684006	2.197704922	0.0381111	0.286866365
ENSG00000108828	VAT1	0.645585012	6.414272098	2.13607355	0.043339266	0.302814704
ENSG00000131981	LGALS3	-0.912674472	7.625065929	-2.165735295	0.04074735	0.295183138
ENSG00000047579	DTNBP1	0.647262679	5.46342283	2.089513436	0.047707593	0.316256796
ENSG00000100485	SOS2	-0.882130532	5.896263821	-2.109055088	0.045828262	0.310051956
ENSG00000101940	WDR13	0.753682491	6.702406817	2.13897825	0.043079047	0.302439968
ENSG00000113758	DBN1	0.699127925	7.705738914	2.163203434	0.040963006	0.295789477
ENSG00000143442	POGZ	-0.675261698	7.080124078	-2.146529263	0.042409152	0.300018939
ENSG00000058085	LAMC2	-2.893381336	6.22522429	-2.120030128	0.044802146	0.307065891
ENSG00000113719	ERGIC1	0.6201209	6.811231991	2.139592194	0.043024227	0.302190307
ENSG00000169764	UGP2	-0.728016155	7.309435207	-2.147999058	0.042279854	0.300018939
ENSG00000051108	HERPUD1	0.681999586	6.800701705	2.135353483	0.043403991	0.302814704
ENSG00000013441	CLK1	-0.84136588	7.556550202	-2.153757359	0.041776707	0.297827488
ENSG00000104517	UBR5	-0.839936343	7.924016476	-2.16031966	0.041209891	0.296230276
ENSG00000116641	DOCK7	-0.677320149	6.741188039	-2.132224262	0.043686283	0.302814704
ENSG00000141140	MYO19	-0.81295295	6.481752921	-2.123500154	0.044482033	0.30578086
ENSG00000028528	SNX1	-0.894597648	7.335897	-2.147131413	0.042356138	0.300018939
ENSG00000146067	FAM193B	-0.768762725	6.299892163	-2.115668324	0.04520746	0.3081452
ENSG00000136636	KCTD3	-0.885040616	6.167664317	-2.107980941	0.045929814	0.310051956
ENSG00000102317	RBM3	0.532905628	8.113065543	2.159294727	0.041297959	0.296404202
ENSG00000086015	MAST2	-0.692671253	7.578695673	-2.145422114	0.042506784	0.300438712
ENSG00000111199	TRPV4	-1.530425987	5.849544482	-2.079408617	0.048706183	0.319101763
ENSG00000110048	OSBP	-0.637975608	5.993025209	-2.095307458	0.047143308	0.314322457
ENSG00000058668	ATP2B4	-1.088983977	6.487172135	-2.11040146	0.045701258	0.309895609
ENSG00000101596	SMCHD1	-0.95280615	6.152780063	-2.098616448	0.046823731	0.313116051
ENSG00000163520	FBLN2	-1.215954628	7.850787646	-2.142156094	0.04279597	0.301358608
ENSG00000077235	GTF3C1	0.636035151	7.597989568	2.13663692	0.043288686	0.302814704
ENSG00000136830	FAM129B	-0.769124084	8.650641752	-2.152765886	0.041862953	0.298036113
ENSG00000088882	CPXM1	1.602338293	6.145921461	2.091744251	0.04748962	0.315478251
ENSG00000125656	CLPP	0.761856311	6.101948567	2.089545929	0.047704411	0.316256796
ENSG00000118523	CTGF	-1.351767922	7.176141137	-2.119899018	0.044814282	0.307065891
ENSG00000266714	MYO15B	-1.322637	7.199512317	-2.118496284	0.044944303	0.307203307
ENSG00000155463	OXA1L	0.745322516	7.210476204	2.118405244	0.044952753	0.307203307

ENSG00000008869	HEATR5B	-0.78604292	5.884778116	-2.069532615	0.049700172	0.323184998
ENSG00000152767	FARP1	-0.823575513	8.316828204	-2.133739394	0.043549395	0.302814704
ENSG00000064932	SBNO2	-0.771747056	8.023913012	-2.127168766	0.044145835	0.304539381
ENSG00000105738	SIPA1L3	-0.710881585	6.405246745	-2.081992467	0.048449076	0.318503349
ENSG00000130429	ARPC1B	0.769478104	7.519971661	2.109699112	0.045767471	0.310051956
ENSG00000174243	DDX23	0.687357999	6.462392569	2.078919091	0.048755031	0.319288477
ENSG00000182809	CRIP2	-0.862140253	7.656812905	-2.109270323	0.045807938	0.310051956
ENSG00000164919	COX6C	-0.809908688	7.764022941	-2.110538192	0.045688378	0.309895609
ENSG00000153071	DAB2	0.832391347	6.353603194	2.071883606	0.049461923	0.322571504
ENSG00000163399	ATP1A1	-0.787859669	9.807498922	-2.137414958	0.04321892	0.302814704
ENSG00000013275	PSMC4	0.67311129	7.352964763	2.099560564	0.046732906	0.313102489
ENSG00000140575	IQGAP1	-0.84874551	8.331800377	-2.111420273	0.045605363	0.30966457
ENSG00000165801	ARHGEF40	1.027534324	7.277297866	2.077906111	0.048856251	0.319684501
ENSG00000143753	DEGS1	0.750972484	7.029998228	2.072643336	0.049385151	0.322472909
ENSG00000108883	EFTUD2	0.584380412	7.812814475	2.088263493	0.047830115	0.316370032
ENSG00000163430	FSTL1	1.145309652	7.727523711	2.085225496	0.048129082	0.317441541
ENSG00000197043	ANXA6	0.803923651	8.866393437	2.102383813	0.046462248	0.311555184
ENSG00000111328	CDK2AP1	-0.61252685	7.067662633	-2.066817509	0.049976595	0.323774874
ENSG00000152818	UTRN	-0.79563747	7.136761982	-2.067333222	0.049923985	0.323701342
ENSG00000004534	RBM6	-0.692642502	8.12074581	-2.079478284	0.048699235	0.319101763
ENSG00000223865	HLA-DPB1	1.428713767	7.573757773	2.067699239	0.049886676	0.323593153
ENSG00000101182	PSMA7	0.749834515	8.736744023	2.076829639	0.048964021	0.320256129
