# Supplementary material for: Genomic and transcriptomic heterogeneity in metaplastic carcinomas of the breast
Source: NPJ Breast Cancer. 2017 Dec 1;3:48. doi: 10.1038/s41523-017-0048-0 (PMC5711926; doi:10.1038/s41523-017-0048-0)
Supplement: Supplementary file 26 — Supplementary Table 14 [file 41523_2017_48_MOESM26_ESM.pdf]

Supplementary Table 14: Summary of differentially expressed genes between chondroid and non-chondroid MBCs according to RNA-sequencing. Statistics calculated using the limma package. Genes with p-value <0.05 have been included.

gene_id	gene_name	Fold Change (log2)	Average expression	t-statistic	p value	adjusted p value
ENSG00000075035	WSCD2	4.260780087	2.046062446	8.976662311	5.77E-09	9.05E-05
ENSG00000005513	SOX8	4.394355318	2.241349383	8.072089363	3.75E-08	0.000294488
ENSG00000167614	TTYH1	5.204656986	4.988806292	7.522370458	1.24E-07	0.000646203
ENSG00000158008	EXTL1	5.084354352	3.939506608	6.893464193	5.07E-07	0.001989132
ENSG00000157766	ACAN	5.473323561	6.965989872	6.697189888	7.96E-07	0.002498477
ENSG00000107104	KANK1	2.167351359	6.317463229	6.412280091	1.55E-06	0.00349175
ENSG00000109846	CRYAB	3.986506469	6.853768762	6.292983571	2.05E-06	0.003749031
ENSG00000100146	SOX10	5.091025627	3.702498185	6.408934044	1.56E-06	0.00349175
ENSG00000112280	COL9A1	5.831986951	3.672248479	6.271967264	2.15E-06	0.003749031
ENSG00000165795	NDRG2	2.725079565	7.92550981	6.064413264	3.52E-06	0.005522301
ENSG00000114547	ROPN1B	4.165761179	3.141839037	5.978019649	4.33E-06	0.006171551
ENSG00000155130	MARCKS	-2.460030248	5.847092075	-5.857290742	5.78E-06	0.006977818
ENSG00000131771	PPP1R1B	4.68406549	4.645474159	5.877760149	5.50E-06	0.006977818
ENSG00000205403	CFI	-3.004866281	5.416477651	-5.647107909	9.61E-06	0.010769367
ENSG00000103489	XYLT1	2.70939903	4.032419193	5.540440533	1.25E-05	0.013028584
ENSG00000164106	SCRG1	5.12132131	5.113284818	5.460999117	1.51E-05	0.014667278
ENSG00000140545	MFGE8	2.50157764	8.755678812	5.440636735	1.59E-05	0.014667278
ENSG00000148735	PLEKHS1	4.041989524	3.830358196	5.287306103	2.31E-05	0.020172657
ENSG00000121316	PLBD1	-2.12269481	5.759750762	-5.223166868	2.71E-05	0.022376417
ENSG00000185634	SHC4	3.993120173	4.199860577	5.184313786	2.98E-05	0.023392358
ENSG00000166405	RIC3	3.831574576	3.144086695	5.128430423	3.42E-05	0.024408061
ENSG00000187595	ZNF385C	3.607995369	3.010524633	5.134257066	3.37E-05	0.024408061
ENSG00000160310	PRMT2	1.203844059	7.349792468	5.01590502	4.52E-05	0.029928051
ENSG00000065361	ERBB3	3.77641014	5.43637866	5.01072475	4.58E-05	0.029928051
ENSG00000092758	COL9A3	4.68790262	7.762538312	4.942819536	5.42E-05	0.030717159
ENSG00000141526	SLC16A3	-2.268909756	5.522558087	-4.937972698	5.48E-05	0.030717159
ENSG00000182158	CREB3L2	1.654911457	6.054877618	4.921215613	5.71E-05	0.030915799
ENSG00000136099	PCDH8	1.986865833	0.816889319	4.948330905	5.34E-05	0.030717159
ENSG00000111199	TRPV4	2.512497013	5.849544482	4.86835504	6.51E-05	0.03297303
ENSG00000155367	PPM1J	2.412581574	2.257290107	4.948682156	5.34E-05	0.030717159
ENSG00000196839	ADA	-2.0976125	4.23145477	-4.903161588	5.98E-05	0.031253535
ENSG00000078053	AMPH	-3.387902285	3.229454857	-4.851446021	6.79E-05	0.033311659
ENSG00000131089	ARHGEF9	1.724479728	6.069000656	4.786346372	7.99E-05	0.033887951
ENSG00000160307	S100B	4.793247365	4.346149283	4.796078843	7.79E-05	0.033887951
ENSG00000147614	ATP6V0D2	-2.321650989	1.640287894	-4.8340258	7.09E-05	0.033729842
ENSG00000171094	ALK	3.504191014	1.381992444	4.812621171	7.48E-05	0.033887951
ENSG00000136883	KIF12	3.659651883	3.10497081	4.783552071	8.04E-05	0.033887951
ENSG00000172201	ID4	2.582982609	3.659295182	4.772667053	8.26E-05	0.033887951
ENSG00000162413	KLHL21	1.920403109	4.725905653	4.721632084	9.38E-05	0.035895521
ENSG00000146476	C6orf211	-2.060585358	2.21962407	-4.764897304	8.42E-05	0.033887951
ENSG00000169085	C8orf46	3.288694695	1.853637576	4.749572866	8.75E-05	0.034323617
ENSG00000142449	FBN3	4.410299068	4.99407629	4.682717811	0.000103326	0.037704451
ENSG00000122863	CHST3	2.113896549	3.896206076	4.688676772	0.000101806	0.037704451
ENSG00000240038	AMY2B	2.960435565	4.432629988	4.614300603	0.000122506	0.042349794
ENSG00000063180	CA11	2.754750005	3.904555776	4.600296732	0.000126852	0.042349794
ENSG00000144857	BOC	2.869673864	6.876689321	4.579455076	0.000133609	0.043676192
ENSG00000167578	RAB4B	2.889605981	3.423872372	4.607243444	0.000124677	0.042349794
ENSG00000140022	STON2	-2.043166093	1.771243468	-4.600479285	0.000126795	0.042349794
ENSG00000154358	OBSCN	2.743086549	6.419678281	4.526424268	0.000152473	0.048825489
ENSG00000066923	STAG3	1.866224911	4.456383326	4.512848564	0.000157717	0.049494633
ENSG00000181143	MUC16	5.723906116	4.628930034	4.476906766	0.000172489	0.053069004
ENSG00000187957	DNER	4.516508427	3.39789288	4.461284435	0.000179334	0.053092971
ENSG00000107282	APBA1	2.88161828	3.167189868	4.467330943	0.000176653	0.053092971
ENSG00000049089	COL9A2	3.463723966	7.378265287	4.417477706	0.000200013	0.057061887
ENSG00000113916	BCL6	1.937971778	5.942003876	4.403051774	0.000207332	0.057074461
ENSG00000182287	AP1S2	-2.336446743	2.750984241	-4.424713305	0.00019644	0.057061887
ENSG00000175318	GRAMD2	2.981782068	3.18518493	4.406330499	0.000205645	0.057074461
ENSG00000089356	FXYD3	3.973868981	5.751722871	4.377320203	0.000221057	0.059803658

ENSG00000141013	GAS8	1.775068213	4.143846993	4.367262928	0.000226666	0.060281539
ENSG00000205542	TMSB4X	-1.496854073	5.588017902	-4.350223441	0.000236494	0.060833136
ENSG00000108679	LGALS3BP	-2.695589419	7.398872276	-4.352199036	0.000235333	0.060833136
ENSG00000146197	SCUBE3	3.122848443	4.987897268	4.313922166	0.000258873	0.062625334
ENSG00000138801	PAPSS1	1.342872361	7.950737267	4.330637047	0.000248317	0.061846781
ENSG00000065371	ROPN1	3.432130856	2.922829484	4.336939809	0.00024445	0.061846781
ENSG00000079385	CEACAM1	2.627421696	3.937776995	4.313066187	0.000259426	0.062625334
ENSG00000164830	OXR1	1.63535059	5.151216042	4.290410171	0.000274484	0.064282414
ENSG00000198373	WWP2	1.806890975	8.048438417	4.291725238	0.000273586	0.064282414
ENSG00000117983	MUC5B	4.953555488	5.264615045	4.261720734	0.000294809	0.065709525
ENSG00000214871	AC005082.1	1.663092847	0.86677149	4.279138397	0.000282297	0.065140001
ENSG00000143882	ATP6V1C2	3.489733541	3.455570568	4.262079431	0.000294546	0.065709525
ENSG00000157734	SNX22	3.458633137	4.80424293	4.245821356	0.000306712	0.065709525
ENSG00000145020	AMT	2.461000714	3.686779802	4.242599852	0.000309181	0.065709525
ENSG00000164050	PLXNB1	1.801040668	7.971168981	4.240638358	0.000310695	0.065709525
ENSG00000186832	KRT16	4.277096376	6.465541382	4.227919638	0.000320688	0.065709525
ENSG00000166016	ABTB2	1.534595046	5.015306666	4.21348934	0.000332415	0.065709525
ENSG00000128641	MYO1B	-1.712364926	7.243455429	-4.225598028	0.000322547	0.065709525
ENSG00000176845	METRNL	-1.626762311	5.391446178	-4.208301298	0.000336734	0.065709525
ENSG00000162909	CAPN2	1.19210144	9.029866242	4.235097056	0.00031501	0.065709525
ENSG00000140511	HAPLN3	2.604636696	5.155899725	4.205362985	0.000339205	0.065709525
ENSG00000064692	SNCAIP	-2.336745133	2.376273714	-4.233097201	0.000316582	0.065709525
ENSG00000155850	SLC26A2	2.395320234	2.708790069	4.216019988	0.000330328	0.065709525
ENSG00000123358	NR4A1	1.857324159	5.925193163	4.18502395	0.000356813	0.067420985
ENSG00000115295	CLIP4	2.01879088	4.75478651	4.16083376	0.000378943	0.067420985
ENSG00000147799	ARHGAP39	2.284601548	4.973662562	4.160119014	0.000379617	0.067420985
ENSG00000213064	SFT2D2	1.601807614	5.27546891	4.161664868	0.00037816	0.067420985
ENSG00000062282	DGAT2	3.173463438	3.959820321	4.155187753	0.000384301	0.067420985
ENSG00000173209	AHSA2	1.397715942	6.070428433	4.159759883	0.000379956	0.067420985
ENSG00000172159	FRMD3	2.447721444	3.091479135	4.166866531	0.000373299	0.067420985
ENSG00000166535	A2ML1	3.405268568	3.658023663	4.145913043	0.000393268	0.06781065
ENSG00000149257	SERPINH1	-1.766755363	7.64248363	-4.152673973	0.000386711	0.067420985
ENSG00000179218	CALR	-1.233595481	10.66644126	-4.157361609	0.000382229	0.067420985
ENSG00000067992	PDK3	-1.819277009	4.118841402	-4.122172709	0.000417179	0.070384462
ENSG00000153902	LGI4	2.966010883	3.012389533	4.117773182	0.000421766	0.070384462
ENSG00000185189	NRBP2	2.143796219	7.065582524	4.123511327	0.000415793	0.070384462
ENSG00000104892	KLC3	2.205086808	2.964408732	4.113294534	0.000426488	0.070384462
ENSG00000197329	PELI1	1.710645418	4.374786414	4.083452652	0.000459316	0.072071209
ENSG00000146904	EPHA1	2.606586486	4.180191762	4.083684186	0.000459051	0.072071209
ENSG00000169891	REPS2	-1.742910936	2.658641736	-4.109411906	0.000430623	0.070384462
ENSG00000003249	DBNDD1	2.950989827	2.877732965	4.098241209	0.000442745	0.07088889
ENSG00000137404	NRM	-2.365473374	3.321541983	-4.102796804	0.000437761	0.070813498
ENSG00000134775	FHOD3	-2.442497108	4.443321743	-4.04175068	0.000509434	0.07789171
ENSG00000073910	FRY	2.133206408	5.23804457	4.042962662	0.000507903	0.07789171
ENSG00000015475	BID	-2.036653776	4.449475893	-4.023711303	0.000532761	0.080380354
ENSG00000206503	HLA-A	-2.018138602	9.7686692	-4.04027565	0.000511302	0.07789171
ENSG00000059588	TARBP1	1.509641163	6.664019752	4.015786085	0.000543342	0.080429976
ENSG00000163617	KIAA1407	1.907974434	3.614073543	3.996837088	0.000569492	0.082318518
ENSG00000168300	PCMTD1	1.515034571	5.064142452	3.995179238	0.000571839	0.082318518
ENSG00000143458	GABPB2	1.319441767	6.648172019	4.011325615	0.000549388	0.080564962
ENSG00000117385	LEPRE1	-1.832388643	7.795555592	-4.016318391	0.000542625	0.080429976
ENSG00000137745	MMP13	-3.761737168	6.077141083	-3.968790322	0.000610504	0.085530473
ENSG00000154127	UBASH3B	-2.150652062	2.826443138	-3.972511233	0.000604899	0.085530473
ENSG00000121005	CRISPLD1	2.372006368	6.997444444	3.971305113	0.00060671	0.085530473
ENSG00000181085	MAPK15	3.84809448	4.079476354	3.940453818	0.000654909	0.0909396
ENSG00000158106	RHPN1	2.696180776	5.33221868	3.934778301	0.00066418	0.091417946
ENSG00000148218	ALAD	1.321567286	5.891099351	3.924766127	0.000680853	0.092897892
ENSG00000120251	GRIA2	2.553740584	1.445232599	3.912081069	0.000702572	0.094222741
ENSG00000150764	DIXDC1	1.749821121	4.361629317	3.894618576	0.000733597	0.096285684
ENSG00000104413	ESRP1	3.71058339	5.533385619	3.89847074	0.000726639	0.096285684
ENSG00000157379	DHRS1	1.309151396	5.387988058	3.893097087	0.000736364	0.096285684
ENSG00000133048	CHI3L1	4.844764518	7.332063101	3.913451357	0.000700193	0.094222741
ENSG00000152661	GJA1	-2.160415623	3.253324214	-3.886373906	0.000748712	0.096309967

ENSG00000185946	RNPC3	1.64870285	5.200905698	3.871649111	0.000776477	0.09905452
ENSG00000167850	CD300C	-1.782804026	1.911598665	-3.868296944	0.000782939	0.099073358
ENSG00000198515	CNGA1	2.727061408	2.443037927	3.859329142	0.000800488	0.099283749
ENSG00000185697	MYBL1	2.341343207	3.679172803	3.84776968	0.000823683	0.099283749
ENSG00000129646	QRICH2	2.450977885	5.097852409	3.854577154	0.000809944	0.099283749
ENSG00000102313	ITIH6	3.197117497	1.908566335	3.85215192	0.000814812	0.099283749
ENSG00000141522	ARHGDIA	-1.09254317	8.459950059	-3.886312938	0.000748825	0.096309967
ENSG00000154553	PDLIM3	2.184399722	5.343214314	3.849185067	0.000820807	0.099283749
ENSG00000197748	WDR96	2.259171052	1.551380043	3.843925392	0.000831543	0.099283749
ENSG00000064225	ST3GAL6	1.860885861	4.653048643	3.834018119	0.000852142	0.099593237
ENSG00000117595	IRF6	3.577313365	5.708287469	3.839083085	0.000841549	0.099283749
ENSG00000113532	ST8SIA4	-2.350309535	2.660585014	-3.824710707	0.000871952	0.100601515
ENSG00000128656	CHN1	-1.748801346	4.567631464	-3.812882651	0.000897785	0.100665976
ENSG00000198804	MT-CO1	1.092047442	8.698783688	3.857519374	0.000804076	0.099283749
ENSG00000188783	PRELP	3.028759049	4.821022406	3.813298755	0.000896864	0.100665976
ENSG00000114993	RTKN	1.732898001	6.34749544	3.831779288	0.000856866	0.099593237
ENSG00000196187	TMEM63A	1.579430947	7.726661567	3.84209953	0.000835302	0.099283749
ENSG00000164976	KIAA1161	1.873799372	3.467000839	3.801098747	0.000924272	0.100665976
ENSG00000139631	CSAD	2.082023941	6.253452566	3.818582565	0.000885244	0.100665976
ENSG00000099284	H2AFY2	-2.006126373	4.864343474	-3.790926312	0.000947756	0.100665976
ENSG00000157368	IL34	3.068803427	3.704320233	3.787228052	0.000956439	0.100665976
ENSG00000165959	CLMN	1.442289617	5.065629848	3.79643972	0.000934956	0.100665976
ENSG00000254470	AP5B1	1.898616744	2.948486259	3.794428369	0.000939606	0.100665976
ENSG00000161249	DMKN	3.323613346	5.870086219	3.80131268	0.000923785	0.100665976
ENSG00000243244	STON1	1.981189948	1.860178705	3.781885608	0.000969121	0.100665976
ENSG00000196090	PTPRT	2.043928531	1.003355496	3.779757875	0.000974218	0.100665976
ENSG00000183960	KCNH8	2.443848686	1.525363319	3.780361661	0.000972769	0.100665976
ENSG00000204634	TBC1D8	1.403626617	5.838144787	3.792693368	0.000943635	0.100665976
ENSG00000073282	TP63	-2.751629202	1.900264226	-3.771282775	0.000994783	0.100665976
ENSG00000182600	C2orf82	3.269030978	3.668754355	3.766734793	0.001005994	0.100665976
ENSG00000162004	CCDC78	2.733838635	2.311818654	3.77028773	0.000997225	0.100665976
ENSG00000042445	RETSAT	1.327013763	7.279570939	3.803967864	0.000917753	0.100665976
ENSG00000213865	C8orf44	1.628902142	3.182362429	3.762302012	0.001017041	0.100665976
ENSG00000106078	COBL	2.750606424	3.478448723	3.758316093	0.001027077	0.100665976
ENSG00000141736	ERBB2	1.709027053	7.883644722	3.801243703	0.000923942	0.100665976
ENSG00000184454	NCMAP	2.408940229	3.324784787	3.757284716	0.00102969	0.100665976
ENSG00000092295	TGM1	2.506569497	2.801801838	3.757722603	0.00102858	0.100665976
ENSG00000143184	XCL1	-1.775651587	1.041093498	-3.743238558	0.00106593	0.101974147
ENSG00000102890	ELMO3	2.452104677	4.498802985	3.737963918	0.00107986	0.101974147
ENSG00000253276	CCDC71L	1.80038492	0.904509492	3.735916774	0.001085315	0.101974147
ENSG00000169908	TM4SF1	2.673536774	6.64978359	3.763379959	0.001014344	0.100665976
ENSG00000204947	ZNF425	1.813436834	1.823359042	3.736387404	0.001084059	0.101974147
ENSG00000198786	MT-ND5	1.151317154	8.674673015	3.777777012	0.000978986	0.100665976
ENSG00000105519	CAPS	2.08750829	4.801973783	3.728178856	0.00110618	0.102294558
ENSG00000170775	GPR37	2.048372826	1.209627052	3.727406872	0.001108283	0.102294558
ENSG00000051523	CYBA	-1.879277939	7.673994876	-3.756021253	0.001032899	0.100665976
ENSG00000154330	PGM5	2.552838189	2.057989802	3.708933787	0.001159801	0.104583062
ENSG00000011105	TSPAN9	-1.753473549	5.234961293	-3.712574342	0.001149465	0.104255854
ENSG00000156587	UBE2L6	-1.790530658	5.796287595	-3.722158152	0.001122688	0.10301807
ENSG00000160678	S100A1	2.163770688	1.715748381	3.715036855	0.001142526	0.10422889
ENSG00000162998	FRZB	3.223392358	4.139549239	3.69759504	0.001192583	0.104583062
ENSG00000163702	IL17RC	1.68051604	7.369835442	3.741068007	0.001071641	0.101974147
ENSG00000179698	KIAA1875	1.862534539	3.339391341	3.68952782	0.001216459	0.104632035
ENSG00000205238	SPDYE2	1.355198801	4.121814832	3.688245993	0.001220296	0.104632035
ENSG00000185624	P4HB	-1.165976246	10.64254204	-3.752305689	0.001042395	0.100964306
ENSG00000184863	RBM33	0.974504668	5.671827226	3.706489232	0.001166793	0.104583062
ENSG00000180921	FAM83H	1.79168821	5.266212419	3.697879949	0.001191748	0.104583062
ENSG00000077942	FBLN1	-1.934539714	8.471134748	-3.731997899	0.001095833	0.102294558
ENSG00000141738	GRB7	2.699759444	4.039026017	3.677357857	0.00125337	0.105249547
ENSG00000165140	FBP1	-1.9681924	2.930210158	-3.681636886	0.001240268	0.105249547
ENSG00000171492	LRRC8D	1.698425334	1.520098722	3.677046698	0.001254328	0.105249547
ENSG00000188895	MSL1	0.986354023	6.138139972	3.697430849	0.001193064	0.104583062
ENSG00000168487	BMP1	-1.608554611	7.230676476	-3.703527063	0.001175321	0.104583062

ENSG00000168453	HR	2.334262187	5.406183224	3.678445538	0.001250027	0.105249547
ENSG00000182253	SYNM	2.800711525	3.403060678	3.656091898	0.00132053	0.108170124
ENSG00000139192	TAPBPL	-1.647617046	5.181494205	-3.656776304	0.001318315	0.108170124
ENSG00000108950	FAM20A	-2.03181134	4.988163332	-3.655144364	0.001323604	0.108170124
ENSG00000188486	H2AFX	-1.898681807	2.660058978	-3.651847169	0.001334353	0.108483578
ENSG00000198712	MT-CO2	1.049621177	7.563366577	3.693518915	0.001204589	0.104632035
ENSG00000108799	EZH1	1.038032554	5.785488979	3.669888422	0.001276568	0.105982154
ENSG00000106333	PCOLCE	-2.142125847	7.681261794	-3.690732437	0.001212864	0.104632035
ENSG00000067606	PRKCZ	2.014686203	4.672943457	3.644261301	0.00135941	0.109951075
ENSG00000160323	ADAMTS13	2.100673502	3.72375659	3.630682526	0.001405423	0.110010671
ENSG00000147724	FAM135B	3.281341498	1.839763111	3.630753693	0.001405178	0.110010671
ENSG00000065618	COL17A1	4.472019907	4.326055938	3.627554861	0.001416236	0.110010671
ENSG00000157703	SVOPL	2.087682288	1.829390783	3.628546383	0.001412799	0.110010671
ENSG00000204248	COL11A2	3.939388652	8.024094722	3.672359684	0.001268847	0.10590145
ENSG00000154556	SORBS2	2.684292282	5.334221817	3.638573372	0.001378501	0.110010671
ENSG00000163462	TRIM46	1.883241442	3.190836042	3.612307703	0.001470129	0.110383856
ENSG00000139438	FAM222A	2.034767362	1.227559674	3.614479119	0.001462333	0.110383856
ENSG00000103404	USP31	-1.939143332	3.553314614	-3.609956059	0.001478618	0.110383856
ENSG00000197930	ERO1L	-1.926783237	6.195721678	-3.632644469	0.001398681	0.110010671
ENSG00000055163	CYFIP2	-1.660446608	4.58078689	-3.608465807	0.001484023	0.110383856
ENSG00000109861	CTSC	-1.939561301	6.524531938	-3.639821501	0.00137429	0.110010671
ENSG00000115232	ITGA4	-2.427622197	4.806394696	-3.610121721	0.001478019	0.110383856
ENSG00000126803	HSPA2	1.825359617	0.730187485	3.601558946	0.001509326	0.111704752
ENSG00000187609	EXD3	1.355355236	4.668717378	3.60837482	0.001484354	0.110383856
ENSG00000135773	CAPN9	2.249216515	1.244664048	3.599660744	0.001516354	0.111704752
ENSG00000110328	GALNT18	1.765392984	4.972373708	3.608553216	0.001483706	0.110383856
ENSG00000176788	BASP1	-2.427765483	2.966529317	-3.595751664	0.001530928	0.11172928
ENSG00000198695	MT-ND6	1.214658655	7.019575112	3.631748533	0.001401756	0.110010671
ENSG00000121057	AKAP1	1.651455138	5.809936965	3.613712291	0.001465081	0.110383856
ENSG00000158805	ZNF276	1.349828327	5.048603668	3.582447936	0.001581565	0.113316606
ENSG00000049860	HEXB	-0.956554535	8.092194327	-3.620353305	0.001441445	0.110383856
ENSG00000179344	HLA-DQB1	-2.002097067	6.572167263	-3.597196111	0.001525527	0.11172928
ENSG00000214944	ARHGEF28	2.66086007	4.948859218	3.574772826	0.001611521	0.114938091
ENSG00000168539	CHRM1	1.704016382	0.844748225	3.553682672	0.001696729	0.11832614
ENSG00000125740	FOSB	2.030681604	2.531947475	3.551477151	0.001705891	0.118438656
ENSG00000175455	CCDC14	1.539515041	6.275848397	3.589953256	0.0015528	0.112800834
ENSG00000104765	BNIP3L	-1.042291277	5.48172486	-3.568863455	0.001634964	0.116082444
ENSG00000160094	ZNF362	-1.064597792	5.205979226	-3.560968032	0.001666808	0.117132899
ENSG00000174514	MFSD4	2.359843963	3.111653338	3.544096699	0.001736903	0.11944394
ENSG00000130287	NCAN	4.41649207	1.975292379	3.542612128	0.001743207	0.11944394
ENSG00000157423	HYDIN	2.178138262	1.696577969	3.542749657	0.001742622	0.11944394
ENSG00000128266	GNAZ	1.652640566	1.239703843	3.54006889	0.001754059	0.119484305
ENSG00000076554	TPD52	1.728476743	6.633948938	3.58436448	0.00157417	0.113316606
ENSG00000123572	NRK	-2.308783876	1.404422246	-3.523999902	0.001824172	0.121800328
ENSG00000069702	TGFBR3	1.916284932	3.872742594	3.524538882	0.001821776	0.121800328
ENSG00000177106	EPS8L2	2.387494192	7.751319187	3.582596502	0.001580991	0.113316606
ENSG00000163050	ADCK3	1.263273946	5.961211806	3.559656942	0.001672154	0.117132899
ENSG00000018510	AGPS	-1.528293353	5.120878926	-3.530229037	0.001796673	0.121515495
ENSG00000157927	RADIL	2.709688643	2.694097559	3.514121191	0.001868628	0.122121939
ENSG00000117020	AKT3	2.388567355	3.521748411	3.512804008	0.001874635	0.122121939
ENSG00000213214	ARHGEF35	2.38322141	3.495414211	3.511176662	0.001882083	0.122121939
ENSG00000142675	CNKSR1	2.600567355	4.088626566	3.510534675	0.001885029	0.122121939
ENSG00000163354	DCST2	1.83679693	3.055809586	3.502990885	0.001919987	0.122121939
ENSG00000081277	PKP1	3.214141804	5.050024023	3.519098117	0.0018461	0.122121939
ENSG00000143375	CGN	2.98724853	4.459119592	3.507044299	0.001901126	0.122121939
ENSG00000158006	PAFAH2	1.493806886	4.586328688	3.504973307	0.00191074	0.122121939
ENSG00000002586	CD99	-1.525422349	8.614795966	-3.560423132	0.001669028	0.117132899
ENSG00000104687	GSR	-1.26192524	6.653908919	-3.538910082	0.001759026	0.119484305
ENSG00000120509	PDZD11	-1.234674601	4.783166477	-3.496263129	0.001951698	0.122496401
ENSG00000151790	TDO2	-3.438937646	3.255757651	-3.484876089	0.002006541	0.124340787
ENSG00000174501	ANKRD36C	1.567577439	5.930029899	3.524040006	0.001823993	0.121800328
ENSG00000167701	GPT	2.558558991	2.655171134	3.482820265	0.002016602	0.124340787
ENSG00000108244	KRT23	4.421110458	4.941162286	3.493666429	0.001964074	0.122782036

ENSG00000162745	OLFML2B	-2.337053741	6.663747396	-3.519748069	0.001843178	0.122121939
ENSG00000172818	OVOL1	1.918956711	1.140210634	3.468508067	0.002088021	0.124340787
ENSG00000149357	LAMTOR1	-1.005916494	6.367247536	-3.511137686	0.001882262	0.122121939
ENSG00000132793	LPIN3	1.658547158	5.768785152	3.501714483	0.001925965	0.122121939
ENSG00000204655	MOG	2.740630402	1.711274005	3.459911943	0.002132098	0.124831135
ENSG00000119147	C2orf40	3.979603446	3.021095456	3.461035733	0.002126284	0.124831135
ENSG00000104412	EMC2	1.062066808	6.160107187	3.503447479	0.001917854	0.122121939
ENSG00000138193	PLCE1	1.717261995	6.066681454	3.496382417	0.001951132	0.122496401
ENSG00000006747	SCIN	2.632255167	4.637592402	3.465260373	0.002104568	0.124340787
ENSG00000160305	DIP2A	0.980125351	6.71954601	3.500819563	0.001930166	0.122121939
ENSG00000204516	MICB	-1.609459801	3.328185561	-3.44568612	0.002207044	0.128261968
ENSG00000001461	NIPAL3	1.904813613	5.285814241	3.474163986	0.002059507	0.124340787
ENSG00000141934	PPAP2C	2.641699734	5.507642152	3.476581021	0.002047438	0.124340787
ENSG00000166091	CMTM5	1.70895037	0.882486226	3.440435536	0.002235351	0.128389343
ENSG00000117115	PADI2	2.391894005	5.218474707	3.468525451	0.002087933	0.124340787
ENSG00000171045	TSNARE1	1.576661254	5.135970386	3.464614498	0.002107874	0.124340787
ENSG00000163817	SLC6A20	2.762439749	2.596185548	3.437506095	0.002251298	0.128454943
ENSG00000156299	TIAM1	1.301983514	5.527156401	3.472025815	0.002070241	0.124340787
ENSG00000157578	LCA5L	1.651974894	0.988559098	3.433814584	0.002271551	0.12914095
ENSG00000110492	MDK	-1.684830685	7.180308865	-3.488243546	0.001990168	0.12391954
ENSG00000196150	ZNF250	1.329484126	4.830863176	3.448577794	0.002191605	0.127838168
ENSG00000159899	NPR2	2.000295457	6.143080296	3.473020418	0.002065241	0.124340787
ENSG00000100644	HIF1A	-1.133318129	7.22698352	-3.481549291	0.002022846	0.124340787
ENSG00000204149	AGAP6	1.302208334	4.363457759	3.425919048	0.002315467	0.130690647
ENSG00000143514	TP53BP2	1.523660646	6.706682174	3.47065631	0.002077146	0.124340787
ENSG00000105825	TFPI2	-2.100946336	1.750150479	-3.409596145	0.002408901	0.134512662
ENSG00000182795	C1orf116	2.440137604	2.786461085	3.405983687	0.00243007	0.134672184
ENSG00000100225	FBXO7	0.962560149	6.954995415	3.46561463	0.002102757	0.124340787
ENSG00000182771	GRID1	2.144637489	2.191320193	3.40310291	0.002447081	0.134726849
ENSG00000092853	CLSPN	-1.877961602	5.613413925	-3.429885731	0.002293301	0.129906828
ENSG00000060718	COL11A1	3.159971949	7.227051999	3.466641571	0.002097515	0.124340787
ENSG00000136542	GALNT5	-2.175067445	2.977052887	-3.396604355	0.002485884	0.13572409
ENSG00000181135	ZNF707	1.346310641	4.364612076	3.404798764	0.002437053	0.134672184
ENSG00000151651	ADAM8	-1.810730388	5.416092946	-3.417093549	0.002365537	0.133038156
ENSG00000140443	IGF1R	1.83262558	5.912658987	3.439217949	0.002241965	0.128389343
ENSG00000061337	LZTS1	-1.895072589	2.631158075	-3.392608296	0.002510041	0.13572409
ENSG00000148604	RGR	2.74142067	1.753461581	3.390885527	0.002520526	0.13572409
ENSG00000118194	TNNT2	2.665642791	3.124219496	3.38815339	0.002537241	0.13572409
ENSG00000198626	RYR2	2.637489706	2.017742044	3.387461648	0.00254149	0.13572409
ENSG00000179532	DNHD1	1.632171986	4.482914994	3.393133587	0.002506852	0.13572409
ENSG00000140538	NTRK3	2.733228764	3.248495892	3.376269995	0.00261121	0.13572409
ENSG00000160299	PCNT	1.114940485	6.99499491	3.440265576	0.002236273	0.128389343
ENSG00000119431	HDHD3	1.37083238	3.903131572	3.378614365	0.002596453	0.13572409
ENSG00000151883	PARP8	-1.380374929	4.941212065	-3.390476955	0.002523018	0.13572409
ENSG00000117899	MESDC2	-1.251072262	4.984549998	-3.390137142	0.002525094	0.13572409
ENSG00000162148	PPP1R32	1.734815814	2.451403434	3.373778558	0.002626983	0.13572409
ENSG00000154511	FAM69A	-1.727054787	2.168432489	-3.367465817	0.002667362	0.13572409
ENSG00000178538	CA8	2.540249571	3.272349717	3.365151275	0.002682318	0.13572409
ENSG00000172716	SLFN11	-1.53549244	3.889850564	-3.365618524	0.002679293	0.13572409
ENSG00000144504	ANKMY1	1.509749152	4.204282771	3.371390002	0.002642191	0.13572409
ENSG00000111913	FAM65B	-1.917797405	2.714191964	-3.362377844	0.002700347	0.13572409
ENSG00000176490	DIRAS1	1.789578763	1.809493088	3.361741164	0.002704502	0.13572409
ENSG00000038295	TLL1	-2.078658793	1.689427265	-3.359978549	0.002716039	0.13572409
ENSG00000129353	SLC44A2	1.563875478	8.981477769	3.442416988	0.002224627	0.128389343
ENSG00000147416	ATP6V1B2	-1.155894476	6.484392341	-3.404722076	0.002437506	0.134672184
ENSG00000169302	STK32A	2.134138615	1.611065148	3.347134499	0.002801561	0.139111695
ENSG00000070081	NUCB2	-1.197939384	7.480931463	-3.415542839	0.002374443	0.133062109
ENSG00000105767	CADM4	1.574433545	5.376301991	3.382939931	0.002569438	0.13572409
ENSG00000144199	FAHD2B	1.650708011	4.773915258	3.367536402	0.002666908	0.13572409
ENSG00000181240	SLC25A41	1.680012775	0.855370121	3.341533062	0.002839673	0.139380529
ENSG00000073605	GSDMB	1.401809353	5.197486322	3.374346352	0.00262338	0.13572409
ENSG00000073331	ALPK1	1.355388229	4.812282916	3.36449552	0.00268657	0.13572409
ENSG00000158089	GALNT14	-2.336896762	3.57510685	-3.337660206	0.002866319	0.139380529

ENSG00000138606	SHF	1.706677123	3.816632057	3.342900518	0.002830323	0.139380529
ENSG00000134575	ACP2	-1.070382859	5.932416827	-3.382563602	0.002571777	0.13572409
ENSG00000071991	CDH19	2.55388341	3.012946423	3.337554444	0.00286705	0.139380529
ENSG00000197599	CCDC154	1.935009881	2.6878753	3.3357199	0.002879759	0.139380529
ENSG00000114923	SLC4A3	1.739287958	5.614005797	3.37266256	0.002634078	0.13572409
ENSG00000198121	LPAR1	-2.061465014	3.210846464	-3.328165315	0.002932677	0.139868168
ENSG00000188211	NCR3LG1	1.671778176	1.254500241	3.323820115	0.00296354	0.14048612
ENSG00000149269	PAK1	-0.918124609	6.487348381	-3.380749345	0.002583085	0.13572409
ENSG00000243317	C7orf73	1.648380283	2.884456706	3.316780905	0.00301421	0.141181981
ENSG00000108387	SEPT4	1.85156238	6.179266896	3.370674779	0.002646761	0.13572409
ENSG00000133106	EPSTI1	-2.041323639	4.477915043	-3.321056658	0.002983332	0.140772643
ENSG00000158941	CCAR2	-1.042438417	6.195842978	-3.361007925	0.002709296	0.13572409
ENSG00000143178	TBX19	1.711652882	2.721857089	3.303344054	0.003113278	0.143847244
ENSG00000137726	FXYD6	1.825031006	2.457591106	3.302853984	0.00311695	0.143847244
ENSG00000159166	LAD1	3.580088932	4.978823819	3.331426083	0.002909721	0.139868168
ENSG00000204257	HLA-DMA	-1.816846335	6.416997219	-3.353347707	0.002759868	0.137476483
ENSG00000095739	BAMBI	2.086664873	4.950338028	3.328790279	0.002928263	0.139868168
ENSG00000101160	CTSZ	-1.115638207	8.712041633	-3.383919604	0.002563358	0.13572409
ENSG00000140563	MCTP2	-2.462861472	2.774257818	-3.297243064	0.003159296	0.144526301
ENSG00000120708	TGFBI	-2.095607249	9.465356837	-3.381680754	0.002577274	0.13572409
ENSG00000166348	USP54	1.363119551	5.531123475	3.334818048	0.002886027	0.139380529
ENSG00000103490	PYCARD	-1.749267203	4.328334714	-3.293072268	0.003191135	0.145410578
ENSG00000106829	TLE4	-1.470160285	4.780632985	-3.303670045	0.003110837	0.143847244
ENSG00000187650	VMAC	1.616017716	2.699030813	3.286429931	0.003242486	0.147045789
ENSG00000076864	RAP1GAP	2.066921027	4.558363817	3.301448	0.003127509	0.14391128
ENSG00000157322	CLEC18A	2.264273435	2.92393203	3.279911561	0.003293657	0.147613538
ENSG00000156219	ART3	2.949907262	3.231887102	3.279453055	0.003297285	0.147613538
ENSG00000133216	EPHB2	-2.750129235	4.150080845	-3.280973682	0.003285266	0.147613538
ENSG00000135976	ANKRD36	1.450967113	6.17745183	3.334689683	0.002886921	0.139380529
ENSG00000198863	RUNDC1	0.93730872	4.535164057	3.292287527	0.003197161	0.145410578
ENSG00000180155	LYNX1	1.998080917	3.940992352	3.280712	0.003287331	0.147613538
ENSG00000206075	SERPINB5	3.226777836	4.918973547	3.29999728	0.003138441	0.143992026
ENSG00000184949	FAM227A	1.378856836	4.209052779	3.277842167	0.003310065	0.147613538
ENSG00000100985	MMP9	-3.416480483	7.656131586	-3.338139444	0.002863009	0.139380529
ENSG00000106278	PTPRZ1	2.614508867	3.892763457	3.27221207	0.003355109	0.148916886
ENSG00000164398	ACSL6	1.904282091	2.215504559	3.260605738	0.003449851	0.152095283
ENSG00000023171	GRAMD1B	-2.303214016	2.618845869	-3.259325825	0.003460456	0.152095283
ENSG00000084710	EFR3B	1.956221975	3.0621094	3.259776519	0.003456718	0.152095283
ENSG00000120088	CRHR1	2.443402887	2.134581453	3.249800177	0.003540384	0.154311552
ENSG00000104218	CSPP1	1.194534859	6.646350661	3.320473259	0.002987527	0.140772643
ENSG00000146416	AIG1	-1.347726764	5.015854586	-3.271646168	0.00335967	0.148916886
ENSG00000176623	RMDN1	1.068495003	6.338936974	3.313156994	0.003040623	0.141995261
ENSG00000184787	UBE2G2	0.89254439	7.289390572	3.325927327	0.002948533	0.140198299
ENSG00000165055	METTL2B	0.999537014	6.157722377	3.305946499	0.003093847	0.143847244
ENSG00000164733	CTSB	-1.246664735	10.16319205	-3.343629311	0.002825352	0.139380529
ENSG00000017373	SRCIN1	1.960925409	3.355738711	3.241822084	0.0036087	0.155322281
ENSG00000100201	DDX17	0.965861182	8.606979475	3.328971086	0.002926988	0.139868168
ENSG00000174038	C9orf131	1.187211636	0.478347125	3.241317098	0.003613067	0.155322281
ENSG00000111962	UST	-1.893472422	2.609231233	-3.232220962	0.003692609	0.156633899
ENSG00000168672	FAM84B	1.971785706	3.502380498	3.234339269	0.003673935	0.156633899
ENSG00000164418	GRIK2	-2.004889998	1.901567352	-3.231696724	0.003697244	0.156633899
ENSG00000105835	NAMPT	1.96512842	3.659550995	3.234168368	0.003675438	0.156633899
ENSG00000185332	TMEM105	1.755800202	1.769025581	3.228063334	0.003729527	0.156890119
ENSG00000155660	PDIA4	-1.169348911	8.531196529	-3.31889341	0.002998916	0.140886184
ENSG00000081803	CADPS2	-1.528840626	5.165658757	-3.255474165	0.003492562	0.153077608
ENSG00000088836	SLC4A11	2.464034875	4.700468925	3.252275001	0.003519448	0.153826327
ENSG00000081181	ARG2	-1.834389841	1.29591006	-3.224059703	0.003765416	0.157608495
ENSG00000130653	PNPLA7	1.995688735	4.739712164	3.247620274	0.003558925	0.154689991
ENSG00000205220	PSMB10	-1.709239757	2.398144553	-3.217054938	0.003829014	0.158364423
ENSG00000177791	MYOZ1	2.019815712	1.225294601	3.215335594	0.003844782	0.158364423
ENSG00000168476	REEP4	-1.240043823	5.128307296	-3.246069871	0.003572169	0.154721975
ENSG00000135063	FAM189A2	2.138386681	3.145499862	3.215079897	0.003847132	0.158364423
ENSG00000147789	ZNF7	1.450757415	4.199345833	3.2298685	0.003713454	0.156633899

ENSG00000085719	CPNE3	1.249510183	6.262254404	3.277667944	0.00331145	0.147613538
ENSG00000175294	CATSPER1	-1.550449095	1.124447489	-3.212700113	0.003869074	0.158364423
ENSG00000141506	PIK3R5	-2.076478623	3.283367048	-3.206388499	0.003927857	0.159563631
ENSG00000153291	SLC25A27	2.034538958	3.322164736	3.201867026	0.003970497	0.16015698
ENSG00000141655	TNFRSF11A	-2.170807697	1.776819058	-3.196754861	0.004019247	0.160305783
ENSG00000082126	MPP4	1.533001142	0.966535832	3.196127633	0.004025268	0.160305783
ENSG00000189350	FAM179A	1.701020294	2.236988787	3.1906055	0.004078655	0.161611549
ENSG00000125354	SEPT6	-1.352858954	5.702200321	-3.239125713	0.003632076	0.155712873
ENSG00000145386	CCNA2	-2.139523455	4.491787077	-3.196633744	0.004020409	0.160305783
ENSG00000088053	GP6	1.428776233	0.730187485	3.17937719	0.004189327	0.164592552
ENSG00000128872	TMOD2	1.411122282	4.241203182	3.193924312	0.004046488	0.160742882
ENSG00000173950	XXYLT1	-1.3696201	3.793384985	-3.178724756	0.004195846	0.164592552
ENSG00000153404	PLEKHG4B	-2.958628143	3.956441829	-3.177357131	0.004209543	0.164718065
ENSG00000196230	TUBB	-1.537884934	5.499084262	-3.213456813	0.003862084	0.158364423
ENSG00000185274	WBSCR17	2.431882866	2.423305389	3.170269883	0.004281219	0.164843483
ENSG00000165816	VWA2	-1.620311298	1.046161535	-3.166432227	0.004320522	0.164843483
ENSG00000204323	SMIM5	1.440672739	1.103119838	3.161864934	0.00436775	0.164843483
ENSG00000205002	AARD	3.380327762	3.83853757	3.171044209	0.004273331	0.164843483
ENSG00000147443	DOK2	-2.059985111	2.954220495	-3.158359275	0.004404339	0.164843483
ENSG00000183837	PNMA3	1.294358406	0.701632965	3.162722936	0.00435884	0.164843483
ENSG00000188735	TMEM120B	1.087392585	5.055084638	3.200167928	0.003986636	0.160305783
ENSG00000181856	SLC2A4	1.692378517	1.541426374	3.157594984	0.004412355	0.164843483
ENSG00000110693	SOX6	1.936876662	3.091695085	3.157875539	0.004409411	0.164843483
ENSG00000012660	ELOVL5	-0.976555224	6.318710125	-3.223917932	0.003766693	0.157608495
ENSG00000183833	MAATS1	1.996473112	1.591308545	3.155201294	0.004437552	0.164999119
ENSG00000076826	CAMSAP3	2.699600747	3.969498936	3.166560907	0.004319198	0.164843483
ENSG00000168140	VASN	-1.688323826	2.853961165	-3.153273597	0.004457945	0.165315604
ENSG00000003137	CYP26B1	2.136353628	3.883393449	3.163786329	0.004347821	0.164843483
ENSG00000153707	PTPRD	-1.935828173	4.27470837	-3.159447612	0.004392948	0.164843483
ENSG00000011600	TYROBP	-2.132367397	7.106059719	-3.230218751	0.003710343	0.156633899
ENSG00000113368	LMNB1	-1.663566451	6.276498436	-3.211995573	0.003875594	0.158364423
ENSG00000003056	M6PR	-1.13506631	6.447588264	-3.215722275	0.00384123	0.158364423
ENSG00000175426	PCSK1	-2.073352778	1.588623728	-3.146276367	0.004532729	0.166201704
ENSG00000108518	PFN1	-1.004265216	5.326616765	-3.187685833	0.004107158	0.162047316
ENSG00000196739	COL27A1	1.640562488	8.742391769	3.245227848	0.003579382	0.154721975
ENSG00000052126	PLEKHA5	-1.228965897	6.3342602	-3.207532132	0.003917142	0.159563631
ENSG00000198840	MT-ND3	1.344833114	6.022678446	3.205579989	0.003935449	0.159563631
ENSG00000131368	MRPS25	1.178391025	4.468459287	3.160714651	0.004379723	0.164843483
ENSG00000013810	TACC3	-1.464928337	5.794616973	-3.187364402	0.004110307	0.162047316
ENSG00000135903	PAX3	-1.588206218	1.17003181	-3.135080425	0.004654913	0.1673955
ENSG00000143851	PTPN7	-1.816082905	3.022136236	-3.129505154	0.004716936	0.168595548
ENSG00000166794	PPIB	-1.216601509	7.346746094	-3.216432003	0.003834719	0.158364423
ENSG00000100139	MICALL1	1.159850274	7.069302271	3.21698043	0.003829696	0.158364423
ENSG00000169299	PGM2	1.005339497	5.956801284	3.197103045	0.004015908	0.160305783
ENSG00000148737	TCF7L2	1.147508473	5.183145499	3.175717102	0.004226026	0.164764895
ENSG00000182575	NXPH3	1.234671684	0.686836567	3.130546835	0.004705288	0.168563169
ENSG00000143452	HORMAD1	2.287248596	3.24135245	3.121106114	0.004811879	0.169125787
ENSG00000105499	PLA2G4C	1.884882001	4.180384096	3.134437521	0.004662025	0.1673955
ENSG00000153558	FBXL2	1.732404283	4.461961791	3.141680649	0.004582504	0.1673955
ENSG00000165025	SYK	-1.704464366	4.31799132	-3.12607046	0.004755542	0.169125787
ENSG00000088386	SLC15A1	2.770338603	2.971470494	3.11201294	0.004916745	0.170306078
ENSG00000160588	MPZL3	1.934467324	3.535087041	3.11651561	0.004864547	0.169125787
ENSG00000106686	SPATA6L	1.719376525	1.440966418	3.108286885	0.004960349	0.171061172
ENSG00000186635	ARAP1	-0.909047996	7.923191734	-3.203617484	0.003953936	0.159900029
ENSG00000155093	PTPRN2	-1.657309865	1.50787839	-3.104335227	0.005006999	0.171438413
ENSG00000107242	PIP5K1B	-1.639261774	1.554700499	-3.103661774	0.005014992	0.171438413
ENSG00000139278	GLIPR1	-1.467829296	4.340465066	-3.11591912	0.004871431	0.169125787
ENSG00000065621	GSTO2	2.038773799	2.770678948	3.099950548	0.005059256	0.171941268
ENSG00000182134	TDRKH	1.294792839	4.248469064	3.120458585	0.004819275	0.169125787
ENSG00000167972	ABCA3	1.534449127	5.224759294	3.14764089	0.004518051	0.166201704
ENSG00000129451	KLK10	2.983699066	2.881676859	3.099673532	0.005062575	0.171941268
ENSG00000179832	MROH1	1.260897364	6.361927441	3.170607167	0.004277782	0.164843483
ENSG00000117425	PTCH2	1.706977352	3.692244568	3.101193267	0.005044392	0.171941268

ENSG00000186007	LEMD1	2.431320697	2.412035627	3.089415203	0.005186973	0.174280057
ENSG00000162851	TFB2M	0.993145708	4.843057878	3.127417615	0.004740364	0.169047837
ENSG00000140950	TLDC1	1.284980541	4.634528662	3.12032646	0.004820786	0.169125787
ENSG00000101057	MYBL2	-2.081058882	5.926944074	-3.139232464	0.004609235	0.1673955
ENSG00000134253	TRIM45	1.863391603	4.606438173	3.116948227	0.00485956	0.169125787
ENSG00000198795	ZNF521	1.751939253	4.611627991	3.115879201	0.004871892	0.169125787
ENSG00000106012	IQCE	1.126152533	5.275572659	3.136219557	0.004642338	0.1673955
ENSG00000182541	LIMK2	1.72838284	6.750921594	3.167137161	0.004313276	0.164843483
ENSG00000204397	CARD16	-1.904101972	1.835730346	-3.078598412	0.005321327	0.174813374
ENSG00000143847	PPFIA4	1.651784713	4.860353538	3.119366278	0.004831776	0.169125787
ENSG00000067182	TNFRSF1A	-0.915006629	7.709308809	-3.175149837	0.004231741	0.164764895
ENSG00000094755	GABRP	3.541635274	3.52895116	3.084264277	0.00525054	0.174813374
ENSG00000112182	BACH2	1.515652358	1.276523507	3.076825659	0.005343664	0.175003045
ENSG00000081059	TCF7	-1.408238025	4.522069038	-3.097378132	0.005090158	0.172325775
ENSG00000102575	ACP5	-3.499911156	4.987616155	-3.094067426	0.005130197	0.173113811
ENSG00000168685	IL7R	-2.002442001	2.302356541	-3.074328577	0.005375279	0.175003045
ENSG00000029534	ANK1	-1.802444839	1.73001812	-3.074692119	0.005370665	0.175003045
ENSG00000185324	CDK10	1.060037826	6.415575857	3.155460561	0.004434816	0.164999119
ENSG00000172137	CALB2	2.684364667	3.216129161	3.079121862	0.005314749	0.174813374
ENSG00000133460	SLC2A11	1.621430864	3.597894262	3.07827494	0.005325396	0.174813374
ENSG00000110200	ANAPC15	-1.627882943	2.917758298	-3.067249235	0.005465892	0.176573237
ENSG00000087258	GNAO1	2.155460533	1.953622661	3.067241819	0.005465988	0.176573237
ENSG00000034510	TMSB10	-1.20362869	7.58871239	-3.164582386	0.00433959	0.164843483
ENSG00000113328	CCNG1	-1.149513635	5.035148961	-3.104229974	0.005008247	0.171438413
ENSG00000215193	PEX26	0.94943433	5.838159362	3.135094115	0.004654762	0.1673955
ENSG00000145632	PLK2	1.237723421	7.160339826	3.159755072	0.004389736	0.164843483
ENSG00000171004	HS6ST2	-1.559442198	1.192055075	-3.069607669	0.005435543	0.176573237
ENSG00000150782	IL18	-1.797595241	3.036150202	-3.06280429	0.005523534	0.177623974
ENSG00000244045	TMEM199	1.304467996	0.851279478	3.067005881	0.005469033	0.176573237
ENSG00000171219	CDC42BPG	2.637760122	5.583020782	3.123842844	0.004780743	0.169125787
ENSG00000102265	TIMP1	-1.332050803	8.700139057	-3.168132948	0.004303061	0.164843483
ENSG00000102804	TSC22D1	1.008042428	6.361045386	3.14037112	0.004596784	0.1673955
ENSG00000139908	TSSK4	1.367079124	0.960004579	3.060399762	0.005554959	0.178247174
ENSG00000073060	SCARB1	1.470380073	6.932076432	3.147931352	0.004514933	0.166201704
ENSG00000136295	TTYH3	-1.352553768	7.415043425	-3.146209687	0.004533448	0.166201704
ENSG00000204103	MAFB	-1.941909103	2.394386075	-3.048934228	0.005707183	0.181645848
ENSG00000182481	KPNA2	-1.532861148	6.411826916	-3.1231618	0.004788473	0.169125787
ENSG00000134317	GRHL1	2.372013443	4.66517238	3.080312557	0.005299815	0.174813374
ENSG00000197363	ZNF517	1.448830177	3.819843971	3.055342338	0.005621618	0.180017961
ENSG00000156395	SORCS3	2.496957867	1.698394053	3.037105679	0.005868422	0.184902433
ENSG00000136104	RNASEH2B	-1.164285382	5.587135244	-3.093131824	0.005141567	0.173125161
ENSG00000125746	EML2	1.27311975	6.750226255	3.124012925	0.004778814	0.169125787
ENSG00000188234	AGAP4	1.046245706	5.230889379	3.087865259	0.005206022	0.174546347
ENSG00000126562	WNK4	2.008772003	2.597310041	3.028852219	0.00598351	0.187774498
ENSG00000167676	PLIN4	2.353410278	2.325179693	3.027188368	0.006006972	0.187948647
ENSG00000187840	EIF4EBP1	-1.155370933	5.397186523	-3.079428514	0.005310899	0.174813374
ENSG00000035687	ADSS	1.03285271	6.04989178	3.1039797	0.005011217	0.171438413
ENSG00000234745	HLA-B	-1.411686744	11.02539669	-3.152407615	0.004467135	0.165315604
ENSG00000160285	LSS	1.137584347	6.334556597	3.109701873	0.004943747	0.170864156
ENSG00000116157	GPX7	-1.750147887	3.45123765	-3.024829934	0.006040379	0.187948647
ENSG00000111640	GAPDH	-0.923480296	9.219434474	-3.139676284	0.004604378	0.1673955
ENSG00000135917	SLC19A3	1.889108423	1.862954283	3.019969706	0.006109788	0.188717887
ENSG00000104231	ZFAND1	1.359306754	5.403939161	3.082453196	0.005273068	0.174813374
ENSG00000221818	EBF2	1.534009975	1.313842683	3.018095535	0.006136757	0.188845072
ENSG00000158869	FCER1G	-2.094313317	5.036064157	-3.049849141	0.00569489	0.181623007
ENSG00000064309	CDON	1.475012107	4.33585633	3.043018627	0.005787281	0.183121442
ENSG00000159733	ZFYVE28	1.476383426	3.334692608	3.018011001	0.006137976	0.188845072
ENSG00000145569	FAM105A	-1.674516927	3.224566861	-3.012188874	0.006222502	0.189167736
ENSG00000197635	DPP4	-2.512066043	3.007293081	-3.01275751	0.006214198	0.189167736
ENSG00000130635	COL5A1	-2.118550753	10.09709301	-3.135566877	0.004649539	0.1673955
ENSG00000083814	ZNF671	1.703122171	1.370674933	3.010279246	0.006250469	0.189167736
ENSG00000165698	C9orf9	1.616144163	2.58851501	3.009910424	0.006255884	0.189167736
ENSG00000124731	TREM1	-1.713241753	1.949911734	-3.008962393	0.006269824	0.189167736

ENSG00000134668	SPOCD1	-2.111372939	2.546692242	-3.007226626	0.006295425	0.189167736
ENSG00000103351	CLUAP1	1.346598522	4.651819633	3.046564009	0.005739147	0.18229344
ENSG00000163132	MSX1	-2.097958348	2.600550435	-3.002993575	0.006358278	0.189386518
ENSG00000164823	OSGIN2	1.559415325	4.06622474	3.024791255	0.006040928	0.187948647
ENSG00000123836	PFKFB2	1.563624747	4.111581523	3.024226917	0.00604895	0.187948647
ENSG00000175029	CTBP2	1.012266006	4.631149506	3.039159579	0.005840114	0.18438075
ENSG00000149927	DOC2A	1.898860708	2.969759694	3.005920592	0.006314753	0.189167736
ENSG00000163507	KIAA1524	-1.317783138	4.457881509	-3.021104638	0.006093512	0.188623134
ENSG00000198408	MGEA5	1.027486918	7.093615681	3.096905036	0.005095861	0.172325775
ENSG00000196236	XPNPEP3	0.782236731	6.325498145	3.083388798	0.005261418	0.174813374
ENSG00000099974	DDTL	1.617422498	2.368172791	2.993797097	0.00649691	0.190592569
ENSG00000174844	DNAH12	1.602791797	0.923880529	2.998707999	0.006422524	0.190502507
ENSG00000109805	NCAPG	-2.086569249	4.347910207	-3.008132568	0.006282051	0.189167736
ENSG00000214021	TTLL3	1.669779511	3.747082757	3.004602021	0.006334325	0.18931789
ENSG00000203685	C1orf95	1.821510175	1.3851807	2.989667856	0.006560093	0.190592569
ENSG00000119535	CSF3R	-1.97171272	4.389686562	-3.005752483	0.006317245	0.189167736
ENSG00000173705	SUSD5	2.070223817	2.235447962	2.989549463	0.006561914	0.190592569
ENSG00000133800	LYVE1	-1.716046701	1.91659213	-2.985624835	0.006622528	0.190592569
ENSG00000186862	PDZD7	1.488982062	2.648898044	2.985468395	0.006624955	0.190592569
ENSG00000167880	EVPL	2.860137801	4.108936394	3.007710881	0.006288272	0.189167736
ENSG00000171522	PTGER4	-1.566626731	1.596936977	-2.983773436	0.006651308	0.190592569
ENSG00000146802	TMEM168	1.349029766	4.035862124	3.00201923	0.00637283	0.189386518
ENSG00000048707	VPS13D	1.007991338	6.534484808	3.074288612	0.005375787	0.175003045
ENSG00000076555	ACACB	1.239127828	5.627414894	3.049919915	0.00569394	0.181623007
ENSG00000164961	KIAA0196	0.938918794	7.260316036	3.079737052	0.005307028	0.174813374
ENSG00000090857	PDPR	1.038470725	5.303860122	3.035799269	0.005886496	0.185100225
ENSG00000148225	WDR31	1.736068334	2.948156172	2.970319712	0.006864073	0.193712545
ENSG00000100167	SEPT3	2.13401202	3.643249285	2.978466017	0.006734478	0.191267263
ENSG00000105609	LILRB5	-1.896771965	2.793916332	-2.963018388	0.006982252	0.196341436
ENSG00000163466	ARPC2	-0.827316318	8.95451901	-3.083177423	0.005264048	0.174813374
ENSG00000158109	TPRG1L	1.200868155	4.357229268	2.992510356	0.006516536	0.190592569
ENSG00000006016	CRLF1	2.844966446	4.321843287	2.986916121	0.006602526	0.190592569
ENSG00000163884	KLF15	1.510285873	0.974256099	2.957901435	0.007066233	0.197037386
ENSG00000109046	WSB1	0.905833751	7.656008431	3.062751857	0.005524218	0.177623974
ENSG00000068078	FGFR3	2.394312901	5.415139681	3.016990053	0.006152718	0.18892819
ENSG00000198522	GPN1	-1.038275212	4.763525841	-2.983450136	0.006656346	0.190592569
ENSG00000118508	RAB32	-1.301438334	4.598036371	-2.976920931	0.006758876	0.191267263
ENSG00000167702	KIFC2	2.554326219	6.636429086	3.042925097	0.005788556	0.183121442
ENSG00000130545	CRB3	1.988106881	2.095831439	2.940342401	0.007361822	0.199640978
ENSG00000049283	EPN3	2.290853045	2.708376124	2.942231613	0.00732946	0.199640978
ENSG00000177025	C19orf18	1.764936134	1.484768743	2.941989131	0.007333606	0.199640978
ENSG00000196312	HIATL2	1.146037382	4.9920758	2.997426945	0.006441849	0.190592569
ENSG00000139364	TMEM132B	1.472421941	0.923184915	2.947605416	0.00723815	0.199640978
ENSG00000176731	C8orf59	1.284022204	4.424083818	2.977830357	0.006744505	0.191267263
ENSG00000185567	AHNAK2	1.994540033	3.87166029	2.960089843	0.007030199	0.196983655
ENSG00000255823	MTRNR2L8	1.559270489	1.591308545	2.93827699	0.007397358	0.199640978
ENSG00000136999	NOV	-1.939137388	2.800323318	-2.939681287	0.007373179	0.199640978
ENSG00000173083	HPSE	-1.25509242	3.82982224	-2.943072774	0.007315095	0.199640978
ENSG00000114841	DNAH1	1.582864105	5.176021621	2.994620463	0.006484381	0.190592569
ENSG00000196954	CASP4	-1.421999298	5.4738213	-2.990442545	0.006548195	0.190592569
ENSG00000106772	PRUNE2	1.925023333	3.294161856	2.938079348	0.007400767	0.199640978
ENSG00000166819	PLIN1	2.77055954	2.282471361	2.93636533	0.007430395	0.199640978
ENSG00000134779	TPGS2	-1.218444201	6.219063146	-3.006183983	0.006310851	0.189167736
ENSG00000180438	TPRXL	1.631102038	1.242356072	2.925171191	0.007626697	0.202146129
ENSG00000163444	TMEM183A	1.17121071	4.047689626	2.945929018	0.007266518	0.199640978
ENSG00000203666	EFCAB2	1.267229321	3.025814939	2.922879703	0.007667487	0.202884563
ENSG00000089057	SLC23A2	1.345759483	5.446933395	2.990576721	0.006546136	0.190592569
ENSG00000198938	MT-CO3	1.051737166	7.421435888	3.025365797	0.006032773	0.187948647
ENSG00000131746	TNS4	3.203133121	3.66017769	2.925191033	0.007626345	0.202146129
ENSG00000050327	ARHGEF5	1.825152612	2.973775092	2.910994081	0.007882424	0.205795524
ENSG00000001036	FUCA2	-1.142979842	6.103626138	-2.992478324	0.006517025	0.190592569
ENSG00000107077	KDM4C	1.008174656	5.307380672	2.976519011	0.006765237	0.191267263
ENSG00000133026	MYH10	-1.245874376	7.348018057	-3.015714218	0.006171189	0.189125239

ENSG00000198910	L1CAM	-2.088656332	2.907032378	-2.906863754	0.007958453	0.206634589
ENSG00000146411	SLC2A12	-1.873234908	1.726321502	-2.911134209	0.007879856	0.205795524
ENSG00000160255	ITGB2	-1.660231124	7.189783262	-3.009721989	0.006258652	0.189167736
ENSG00000110844	PRPF40B	1.138591107	4.38468005	2.937682952	0.007407609	0.199640978
ENSG00000164828	SUN1	1.153280117	7.859936883	3.021021743	0.006094699	0.188623134
ENSG00000138964	PARVG	-1.788417363	5.107257894	-2.947250461	0.007244148	0.199640978
ENSG00000108176	DNAJC12	-1.394581674	1.885804208	-2.901074219	0.008066202	0.20815316
ENSG00000183779	ZNF703	-1.465811267	2.349905201	-2.897337339	0.008136487	0.208713002
ENSG00000112183	RBM24	-1.101036629	0.664813302	-2.918132935	0.007752647	0.20410534
ENSG00000160282	FTCD	1.856904756	1.823435204	2.894432068	0.008191533	0.209337698
ENSG00000143951	WDPCP	1.191848553	4.378587102	2.932094887	0.007504706	0.200607056
ENSG00000126602	TRAP1	1.014587808	6.087180006	2.987617767	0.006591681	0.190592569
ENSG00000174428	GTF2IRD2B	1.231393146	5.160332578	2.958776722	0.007051799	0.197037386
ENSG00000196141	SPATS2L	-1.098005098	7.285278183	-3.002217038	0.006369873	0.189386518
ENSG00000140368	PSTPIP1	-2.018884745	4.059660188	-2.901664388	0.008055155	0.20815316
ENSG00000092470	WDR76	-1.443488866	4.624491963	-2.919276409	0.00773205	0.20390521
ENSG00000095637	SORBS1	2.141595174	4.872503751	2.941914221	0.007334888	0.199640978
ENSG00000108924	HLF	1.881930823	1.301593483	2.888191592	0.008310975	0.211357391
ENSG00000176853	FAM91A1	1.199539645	4.546480506	2.928408771	0.007569419	0.201404143
ENSG00000147862	NFIB	2.103421823	6.733659326	2.991221958	0.006536244	0.190592569
ENSG00000148600	CDHR1	1.969734434	2.106480722	2.88721378	0.00832984	0.211494371
ENSG00000111817	DSE	-1.566613592	2.705807753	-2.881561815	0.008439686	0.211635496
ENSG00000163823	CCR1	-2.046554819	2.04957298	-2.88557284	0.008361591	0.211538892
ENSG00000138600	SPPL2A	-1.207897561	4.762826829	-2.920436735	0.007711203	0.203697799
ENSG00000008196	TFAP2B	2.3055202	1.802961834	2.883356582	0.008404656	0.211635496
ENSG00000112984	KIF20A	-2.055609231	5.367309654	-2.931051797	0.007522965	0.200753125
ENSG00000111206	FOXM1	-1.779324654	5.296921707	-2.928203877	0.007573032	0.201404143
ENSG00000065357	DGKA	1.221244222	5.987909293	2.969514732	0.006877008	0.193729149
ENSG00000071539	TRIP13	-1.878982759	4.692875468	-2.90638893	0.007967238	0.206634589
ENSG00000100558	PLEK2	-1.416156109	3.237350909	-2.879092917	0.008488101	0.211635496
ENSG00000065413	ANKRD44	-1.157441856	3.866034835	-2.89046025	0.008267362	0.210816857
ENSG00000135775	COG2	0.869182635	5.538854095	2.956094512	0.007096119	0.197420563
ENSG00000130414	NDUFA10	0.742905786	6.963733547	2.98781459	0.006588642	0.190592569
ENSG00000128536	CDHR3	1.623064237	2.560427421	2.876668331	0.008535904	0.211635496
ENSG00000141448	GATA6	2.167271283	1.99555901	2.873625895	0.008596252	0.212081439
ENSG00000088325	TPX2	-1.640934224	7.000109496	-2.976931772	0.006758705	0.191267263
ENSG00000172987	HPSE2	2.137748988	0.853349445	2.877780191	0.008513951	0.211635496
ENSG00000110169	HPX	1.526494362	2.525245501	2.870418859	0.008660305	0.212985948
ENSG00000166851	PLK1	-1.404850593	5.398549844	-2.934251596	0.007467088	0.200003473
ENSG00000184381	PLA2G6	1.561596625	5.137042036	2.936659878	0.007425296	0.199640978
ENSG00000160953	MUM1	0.764759857	5.82817525	2.957686087	0.007069788	0.197037386
ENSG00000090520	DNAJB11	-0.802162292	7.710558621	-2.987454985	0.006594196	0.190592569
ENSG00000180113	TDRD6	1.676751536	1.289586014	2.869754235	0.008673636	0.212985948
ENSG00000135047	CTSL	-0.786571345	7.24544267	-2.980018236	0.006710051	0.191267263
ENSG00000121933	ADORA3	-1.668255413	2.46250803	-2.863827988	0.008793371	0.214582864
ENSG00000165891	E2F7	-1.705058891	1.952792342	-2.864599638	0.008777692	0.214533893
ENSG00000107165	TYRP1	1.132614112	0.471815872	2.876999194	0.008529366	0.211635496
ENSG00000183479	TREX2	1.159098066	0.471815872	2.876863982	0.008532037	0.211635496
ENSG00000085117	CD82	-1.386475616	6.503032027	-2.949582207	0.007204836	0.199384616
ENSG00000136156	ITM2B	-1.278931224	8.661725126	-2.98484132	0.006634693	0.190592569
ENSG00000150712	MTMR12	1.494131964	5.793003353	2.941172616	0.007347584	0.199640978
ENSG00000196724	ZNF418	1.362891457	3.139035671	2.854473251	0.008985583	0.216743807
ENSG00000149573	MPZL2	2.068080961	4.946369454	2.91110902	0.007880318	0.205795524
ENSG00000123560	PLP1	2.141889153	1.223600266	2.851027987	0.009057375	0.216975987
ENSG00000128607	KLHDC10	1.130924086	4.17770148	2.880967024	0.008451326	0.211635496
ENSG00000012779	ALOX5	-1.688463924	4.540681574	-2.877180513	0.008525785	0.211635496
ENSG00000151474	FRMD4A	-1.206066409	5.540896711	-2.912892695	0.007847708	0.205795524
ENSG00000170485	NPAS2	1.244138613	6.183060778	2.937524818	0.00741034	0.199640978
ENSG00000118705	RPN2	-0.801739379	9.903540202	-2.9796196	0.006716316	0.191267263
ENSG00000124145	SDC4	1.372838187	7.145448733	2.952981836	0.007147885	0.198508778
ENSG00000128159	TUBGCP6	1.025111074	7.058019601	2.95071652	0.007185785	0.199208764
ENSG00000189180	ZNF33A	1.187359414	4.720114022	2.885961257	0.008354065	0.211538892
ENSG00000156162	DPY19L4	0.996673102	5.090149755	2.899043455	0.008104325	0.208467158

ENSG00000106299	WASL	1.153864375	4.896866868	2.889995202	0.008276285	0.210816857
ENSG00000111728	ST8SIA1	1.874782144	2.884678353	2.828251611	0.00954589	0.222604416
ENSG00000090889	KIF4A	-1.840549277	3.188475774	-2.827551473	0.009561296	0.222604416
ENSG00000151490	PTPRO	-1.462118183	2.555057794	-2.824322578	0.009632653	0.222604416
ENSG00000065717	TLE2	1.397177164	6.563810893	2.934119938	0.00746938	0.200003473
ENSG00000071127	WDR1	-0.805640448	8.947828513	-2.962032017	0.006998366	0.196442512
ENSG00000239665	RP11-295P9.3	1.454704038	2.808814908	2.822408783	0.009675186	0.222604416
ENSG00000135914	HTR2B	-1.431665267	1.089019054	-2.835216982	0.009393897	0.222604416
ENSG00000109674	NEIL3	-1.676521129	2.133491797	-2.821314925	0.009699576	0.222604416
ENSG00000128346	C22orf23	1.936018323	2.825406646	2.824300739	0.009633138	0.222604416
ENSG00000060558	GNA15	-1.884573996	3.173352721	-2.821907732	0.009686351	0.222604416
ENSG00000197381	ADARB1	1.379928091	5.43948472	2.900399333	0.008078852	0.20815316
ENSG00000144847	IGSF11	1.51906667	0.780069656	2.828809303	0.009533635	0.222604416
ENSG00000164574	GALNT10	-1.242652518	5.14432911	-2.876576808	0.008537714	0.211635496
ENSG00000155096	AZIN1	0.863733247	7.505920689	2.94140456	0.007343611	0.199640978
ENSG00000198846	TOX	1.816030848	3.921408689	2.843245051	0.009221569	0.219555976
ENSG00000107186	MPDZ	1.196813469	5.984380491	2.911306763	0.007876696	0.205795524
ENSG00000126368	NR1D1	1.266945383	4.321676	2.85432131	0.008988738	0.216743807
ENSG00000156345	CDK20	1.412253297	2.95882411	2.81669123	0.009803319	0.223096051
ENSG00000181392	SYNE4	1.798210185	3.14099195	2.816040055	0.009818014	0.223096051
ENSG00000157349	DDX19B	1.672522522	2.206170491	2.808355001	0.009993028	0.224251118
ENSG00000188282	RUFY4	-2.172027866	2.103281334	-2.817307085	0.00978944	0.223096051
ENSG00000047578	KIAA0556	0.946208031	4.762541842	2.861447597	0.008841906	0.215289893
ENSG00000067191	CACNB1	1.81342373	4.454980708	2.848734316	0.009105473	0.217795685
ENSG00000138771	SHROOM3	1.854970487	3.963175249	2.827723043	0.009557518	0.222604416
ENSG00000135074	ADAM19	-1.680984733	4.418758295	-2.833664227	0.009427579	0.222604416
ENSG00000106479	ZNF862	1.529678214	3.565052581	2.814601439	0.009850553	0.223096051
ENSG00000183484	GPR132	-1.743901211	2.230427159	-2.803672933	0.010101101	0.225262867
ENSG00000213937	CLDN9	1.235605328	0.471815872	2.821452158	0.009696513	0.222604416
ENSG00000128340	RAC2	-1.776480329	3.986851243	-2.816906467	0.009798466	0.223096051
ENSG00000132792	CTNNBL1	-0.826340433	6.728201163	-2.91000559	0.007900556	0.205926285
ENSG00000101198	NKAIN4	2.014422324	1.112475078	2.80268122	0.010124134	0.225262867
ENSG00000212997	AC023632.1	1.001391663	0.428464954	2.82112834	0.009703742	0.222604416
ENSG00000086300	SNX10	-1.785769344	2.616993366	-2.795223474	0.010298946	0.226967372
ENSG00000154736	ADAMTS5	-1.794188854	1.696654132	-2.796474594	0.010269421	0.22695421
ENSG00000163701	IL17RE	2.554341046	4.804690306	2.851966227	0.00903777	0.216975987
ENSG00000130940	CASZ1	1.949753575	3.37101066	2.802194132	0.010135465	0.225262867
ENSG00000169242	EFNA1	1.165084669	6.403745931	2.901142776	0.008064918	0.20815316
ENSG00000185101	ANO9	2.02373629	4.239548062	2.826759119	0.00957876	0.222604416
ENSG00000154274	C4orf19	1.337063408	0.873302744	2.802644239	0.010124994	0.225262867
ENSG00000171435	KSR2	1.148861673	0.64348565	2.807869103	0.010004192	0.224251118
ENSG00000186897	C1QL4	1.91749123	1.413123029	2.791393548	0.01038983	0.227691095
ENSG00000144115	THNSL2	1.537992578	4.559755235	2.838393463	0.009325349	0.221367696
ENSG00000016391	CHDH	1.779490674	3.681729983	2.803147096	0.010113308	0.225262867
ENSG00000214046	SMIM7	1.146535981	5.633184766	2.874658959	0.008575716	0.211918617
ENSG00000101115	SALL4	-1.776151151	1.829559762	-2.785724526	0.010525752	0.227793619
ENSG00000164434	FABP7	3.035385494	2.919636194	2.786295367	0.010511989	0.227793619
ENSG00000170801	HTRA3	-1.914080736	5.609759035	-2.858429	0.008903821	0.215601632
ENSG00000132383	RPA1	-0.843064825	7.009436248	-2.897125655	0.008140485	0.208713002
ENSG00000119121	TRPM6	1.998129783	2.103058306	2.782997498	0.010591735	0.228055323
ENSG00000139329	LUM	-2.4080921	7.95029641	-2.907535626	0.007946038	0.206634589
ENSG00000155858	LSM11	-1.350047551	2.039418014	-2.776043821	0.010761763	0.228055323
ENSG00000196357	ZNF565	1.306399453	2.950161196	2.779389312	0.01067964	0.228055323
ENSG00000123146	CD97	-1.249233083	6.192695474	-2.87194981	0.008629671	0.212571704
ENSG00000147421	HMBOX1	-1.153762464	3.410734893	-2.779339977	0.010680847	0.228055323
ENSG00000267106	C19orf82	1.345301254	2.743757373	2.774498214	0.010799905	0.228055323
ENSG00000106714	CNTNAP3	1.90010751	3.061600841	2.776350675	0.010754206	0.228055323
ENSG00000198089	SFI1	1.065486186	6.548875543	2.885034181	0.008372038	0.211538892
ENSG00000169764	UGP2	0.84765629	7.309435207	2.89622094	0.008157597	0.208810537
ENSG00000109099	PMP22	1.032951103	6.468701092	2.881564286	0.008439638	0.211635496
ENSG00000100253	MIOX	1.493743476	1.683274976	2.765397791	0.011027092	0.229449642
ENSG00000149548	CCDC15	-1.499684585	2.656766277	-2.764723935	0.011044093	0.229449642
ENSG00000132000	PODNL1	-1.280736462	4.646881317	-2.809073876	0.009976532	0.224251118

ENSG00000213983	AP1G2	1.057154178	5.518557323	2.851602685	0.009045362	0.216975987
ENSG00000124733	MEA1	-0.814396564	6.899462128	-2.881295134	0.008444903	0.211635496
ENSG00000140044	JDP2	-1.358096274	4.39736568	-2.795794759	0.010285455	0.226967372
ENSG00000058091	CDK14	-1.522404017	4.527941858	-2.8004989	0.010174995	0.225821566
ENSG00000141480	ARRB2	-1.433636991	6.112222366	-2.858732356	0.00889758	0.215601632
ENSG00000157335	CLEC18C	1.749285295	1.607718895	2.762381259	0.011103391	0.229601667
ENSG00000143369	ECM1	-1.570145227	5.915191409	-2.85414354	0.00899243	0.216743807
ENSG00000206052	DOK6	-1.615894206	1.198586329	-2.772725668	0.010843805	0.228055323
ENSG00000185551	NR2F2	1.326983416	5.044523007	2.830750405	0.009491096	0.222604416
ENSG00000143344	RGL1	-1.668462825	4.871865042	-2.807870748	0.010004155	0.224251118
ENSG00000165702	GFI1B	1.043724116	0.428464954	2.780175228	0.010660434	0.228055323
ENSG00000171659	GPR34	-1.30996803	0.811895855	-2.775615947	0.010772309	0.228055323
ENSG00000133606	MKRN1	1.059615222	4.110793375	2.785665767	0.01052717	0.227793619
ENSG00000171596	NMUR1	1.698616943	1.586884642	2.751783046	0.01137544	0.232714501
ENSG00000124570	SERPINB6	-0.887566165	6.65180968	-2.864602261	0.008777638	0.214533893
ENSG00000119632	IFI27L2	-1.549685277	5.578362021	-2.828831711	0.009533142	0.222604416
ENSG00000143196	DPT	-2.077774033	3.831986813	-2.764553485	0.011048398	0.229449642
ENSG00000184602	SNN	-1.345306787	2.077156016	-2.749129259	0.011444541	0.233823296
ENSG00000236882	C5orf27	1.858418665	1.226293018	2.753227942	0.011337982	0.232251022
ENSG00000172269	DPAGT1	-0.782933601	5.277610925	-2.822221375	0.00967936	0.222604416
ENSG00000120053	GOT1	0.690359864	5.760886406	2.842612566	0.009235036	0.219555976
ENSG00000155792	DEPTOR	1.444386551	3.565201564	2.761310785	0.011130588	0.229802695
ENSG00000215915	ATAD3C	1.977068827	4.324832548	2.785399124	0.010533605	0.227793619
ENSG00000196757	ZNF700	1.3913355	2.669199708	2.738381323	0.011728484	0.23562562
ENSG00000110876	SELPLG	-1.536443561	1.821125639	-2.740702642	0.0116666	0.23562562
ENSG00000182175	RGMA	2.185139795	3.568011833	2.755975522	0.011267076	0.2320101
ENSG00000124191	TOX2	-2.260080756	2.075109887	-2.737757073	0.011745178	0.23562562
ENSG00000149380	P4HA3	-1.960836719	3.592760181	-2.747228053	0.01149429	0.234362835
ENSG00000009950	MLXIPL	1.338941903	3.31152897	2.746889153	0.011503179	0.234362835
ENSG00000188931	C1orf192	1.0169804	0.428464954	2.762269886	0.011106218	0.229601667
ENSG00000157992	KRTCAP3	2.247898039	3.849213235	2.760157992	0.011159946	0.230106052
ENSG00000127418	FGFRL1	1.705170662	5.492793333	2.825877961	0.009598217	0.222604416
ENSG00000204946	ZNF783	1.064851369	4.896517214	2.802837895	0.010120492	0.225262867
ENSG00000107438	PDLIM1	-1.382856998	5.585324363	-2.814943618	0.009842804	0.223096051
ENSG00000130193	THEM6	1.650499014	2.418131125	2.734405985	0.011835183	0.23562562
ENSG00000131242	RAB11FIP4	1.313803934	4.527744451	2.785147412	0.010539683	0.227793619
ENSG00000196511	TPK1	-1.534545748	1.562646193	-2.737480495	0.011752582	0.23562562
ENSG00000168495	POLR3D	-0.961357821	5.514940101	-2.813315533	0.009879725	0.223375735
ENSG00000185155	MIXL1	1.328513523	1.175025274	2.735880582	0.011795498	0.23562562
ENSG00000213390	ARHGAP19	1.018718943	0.428464954	2.754530902	0.011304305	0.232251022
ENSG00000197629	MPEG1	-1.478035515	1.550609856	-2.733370644	0.011863122	0.23562562
ENSG00000155307	SAMSN1	-1.915090609	2.617064635	-2.724454254	0.012106325	0.236720555
ENSG00000021355	SERPINB1	-1.097943818	5.678455945	-2.808327606	0.009993657	0.224251118
ENSG00000160145	KALRN	1.365077375	5.639814673	2.818138838	0.009770726	0.223096051
ENSG00000066777	ARFGEF1	1.078888193	6.820436721	2.845667736	0.009170157	0.219009036
ENSG00000145730	PAM	1.392681492	9.330936837	2.87463724	0.008576147	0.211918617
ENSG00000204019	CT83	1.706489021	1.435146295	2.721549662	0.012186562	0.236720555
ENSG00000026508	CD44	1.355574043	7.893081896	2.8587164	0.008897909	0.215601632
ENSG00000183421	RIPK4	1.84234964	3.728494504	2.739576604	0.01169658	0.23562562
ENSG00000165138	ANKS6	1.11157388	4.957075304	2.787933623	0.010472587	0.227793619
ENSG00000168961	LGALS9	-1.398117579	4.754951349	-2.765474671	0.011025154	0.229449642
ENSG00000162755	KLHDC9	1.520420323	1.024683148	2.722385053	0.012163434	0.236720555
ENSG00000124813	RUNX2	-2.006620093	3.755445527	-2.728490471	0.011995655	0.236672314
ENSG00000124787	RPP40	-1.16276978	3.692161097	-2.724180462	0.012113867	0.236720555
ENSG00000143870	PDIA6	-0.890047543	9.090806806	-2.861062345	0.008849785	0.215289893
ENSG00000124225	PMEPA1	-1.265976273	6.41013358	-2.819194856	0.009747014	0.223096051
ENSG00000187288	CIDEC	2.212780909	1.57989648	2.714066907	0.012395586	0.23879045
ENSG00000217442	SYCE3	1.376613493	0.848438563	2.722552537	0.012158802	0.236720555
ENSG00000103855	CD276	-1.016239541	6.803964159	-2.830376877	0.009499268	0.222604416
ENSG00000197442	MAP3K5	-1.30411035	5.399667648	-2.780456943	0.010653558	0.228055323
ENSG00000035115	SH3YL1	1.400875833	6.276187968	2.822861806	0.009665102	0.222604416
ENSG00000137872	SEMA6D	-1.770019856	2.412318697	-2.706180881	0.012619535	0.240119332
ENSG00000152926	ZNF117	1.508501814	5.07784742	2.784156711	0.010563639	0.227997326

ENSG00000215529	EFCAB8	1.053503832	0.428464954	2.734892898	0.011822065	0.23562562
ENSG00000142273	CBLC	2.630304305	3.494713408	2.720980602	0.012202341	0.236720555
ENSG00000215045	GRID2IP	1.683405224	2.031743453	2.704594019	0.012665058	0.240119332
ENSG00000167553	TUBA1C	-1.090966207	5.058731808	-2.772550764	0.010848146	0.228055323
ENSG00000196966	HIST1H3E	1.424530866	1.074565319	2.713806543	0.01240292	0.23879045
ENSG00000154978	VOPP1	-1.377613255	2.582139694	-2.703458384	0.012697731	0.240119332
ENSG00000095203	EPB41L4B	2.029777584	3.807635299	2.730504213	0.011940798	0.236399468
ENSG00000166352	C11orf74	-1.558301693	2.606378379	-2.701555427	0.01275266	0.240218464
ENSG00000175782	SLC35E3	1.138121588	4.927900606	2.773301537	0.010829524	0.228055323
ENSG00000154027	AK5	-2.001564801	3.153441409	-2.702903255	0.012713732	0.240119332
ENSG00000156253	RWDD2B	1.268504247	3.815010325	2.725048768	0.012089963	0.236720555
ENSG00000175874	CREG2	-1.264234026	0.768544937	-2.722944667	0.012147964	0.236720555
ENSG00000024862	CCDC28A	1.21135379	4.1980832	2.734994038	0.011819342	0.23562562
ENSG00000197261	C6orf141	1.824781606	2.25715371	2.689651664	0.013101375	0.242136245
ENSG00000164695	CHMP4C	1.824946535	3.727939667	2.7166265	0.012323708	0.238242118
ENSG00000179456	ZBTB18	1.327526794	0.844748225	2.705804563	0.012630317	0.240119332
ENSG00000186684	CYP27C1	1.776276985	2.281242577	2.695181306	0.012938283	0.241908226
ENSG00000168542	COL3A1	-2.23796089	12.84084138	-2.866447792	0.008740247	0.214286276
ENSG00000183423	LRIT3	1.171733502	0.651750794	2.710588996	0.012493886	0.239152111
ENSG00000117280	RAB7L1	1.095694939	5.151567202	2.772850494	0.010840708	0.228055323
ENSG00000198753	PLXNB3	1.430139308	5.7441532	2.792607484	0.010360942	0.227375572
ENSG00000124216	SNAI1	-1.611734083	1.540159717	-2.696448061	0.012901192	0.241854961
ENSG00000148090	AUH	1.808209387	4.157608575	2.727699962	0.012017255	0.236672314
ENSG00000104356	POP1	1.114731722	4.461791013	2.741276808	0.011651341	0.23562562
ENSG00000167920	TMEM99	1.084668556	0.428464954	2.715698182	0.012349732	0.23835134
ENSG00000138162	TACC2	1.24062631	5.924448211	2.793332626	0.010343721	0.227315592
ENSG00000177575	CD163	-1.854253011	6.226147454	-2.788673006	0.010454849	0.227793619
ENSG00000124762	CDKN1A	-1.206989952	4.865789893	-2.743733839	0.011586257	0.235187521
ENSG00000080854	IGSF9B	1.926971406	1.061535393	2.691352313	0.013051011	0.242136245
ENSG00000000971	CFH	-1.478220259	6.33867352	-2.79452389	0.010315491	0.227013141
ENSG00000198888	MT-ND1	1.06039621	8.062853977	2.830382533	0.009499144	0.222604416
ENSG00000204525	HLA-C	-1.189768798	11.32801727	-2.852530925	0.00902599	0.216975987
ENSG00000172197	MBOAT1	1.80119371	5.42298694	2.774276222	0.010805394	0.228055323
ENSG00000204592	HLA-E	-1.09107865	8.777120152	-2.833581796	0.009429371	0.222604416
ENSG00000104972	LILRB1	-1.9383821	3.98690254	-2.700833675	0.012773551	0.240323492
ENSG00000147113	CXorf36	-1.372157655	3.105931129	-2.681064541	0.013358489	0.242859904
ENSG00000088538	DOCK3	2.368304571	2.810320303	2.681929346	0.013332381	0.242859904
ENSG00000180879	SSR4	-0.833652333	8.699860024	-2.830016404	0.00950716	0.222604416
ENSG00000196503	ARL9	1.706587769	1.627934011	2.677295944	0.013472821	0.243273341
ENSG00000169862	CTNND2	2.129847426	1.916691853	2.681459747	0.013346552	0.242859904
ENSG00000157823	AP3S2	1.244844945	2.778017601	2.680789035	0.013366816	0.242859904
ENSG00000086159	AQP6	1.565075886	1.195450105	2.682829219	0.013305266	0.242859904
ENSG00000251322	SHANK3	0.933184262	5.288847482	2.762782484	0.011093214	0.229601667
ENSG00000186648	LRRC16B	2.171658528	2.752151308	2.675233396	0.013535785	0.243273341
ENSG00000187514	PTMA	0.755519115	6.59812813	2.79944969	0.010199534	0.226046452
ENSG00000136636	KCTD3	1.014989008	6.167664317	2.789605745	0.010432514	0.227793619
ENSG00000160781	PAQR6	1.714739977	3.34633303	2.686311499	0.013200826	0.242734786
ENSG00000115138	POMC	1.734534651	0.744256599	2.683577098	0.01328277	0.242859904
ENSG00000072682	P4HA2	-1.336191395	7.719893092	-2.809281412	0.009971775	0.224251118
ENSG00000083307	GRHL2	2.852719202	4.203686018	2.708433941	0.012555166	0.239371939
ENSG00000164742	ADCY1	-2.163668908	2.817799381	-2.670218368	0.013690036	0.243273341
ENSG00000164975	SNAPC3	0.849087223	5.576599482	2.766215197	0.011006502	0.229449642
ENSG00000182827	ACBD3	0.81612853	6.252583023	2.785149776	0.010539626	0.227793619
ENSG00000164692	COL1A2	-1.811815615	13.77255545	-2.844414332	0.009196721	0.21930966
ENSG00000185917	SETD4	0.988455754	4.705864534	2.728159458	0.012004695	0.236672314
ENSG00000126351	THRA	0.994961231	5.319768412	2.754207633	0.011312652	0.232251022
ENSG00000180902	D2HGDH	1.225032182	4.992531223	2.741290945	0.011650966	0.23562562
ENSG00000196177	ACADSB	1.607742204	2.934169504	2.666438813	0.013807379	0.243428747
ENSG00000165323	FAT3	-1.755853032	2.096527053	-2.660799947	0.013984208	0.245168941
ENSG00000117602	RCAN3	1.314771419	3.749755816	2.686014538	0.013209702	0.242734786
ENSG00000169398	PTK2	0.935097674	8.371553604	2.814487415	0.009853136	0.223096051
ENSG00000243660	ZNF487	1.422681561	1.134946037	2.670630142	0.013677309	0.243273341
ENSG00000162913	C1orf145	1.341869803	1.268258363	2.666812535	0.013795734	0.243428747

ENSG00000182568	SATB1	-1.668386153	3.625814973	-2.671115404	0.013662324	0.243273341
ENSG00000162627	SNX7	-1.05706549	4.838826561	-2.72088872	0.01220489	0.236720555
ENSG00000111186	WNT5B	-1.9051401	2.757350389	-2.663682539	0.013893548	0.244398729
ENSG00000105991	HOXA1	-1.302340543	0.758742004	-2.688483045	0.013136089	0.242207256
ENSG00000182628	SKA2	-1.09856123	5.363368967	-2.739679646	0.011693834	0.23562562
ENSG00000147588	PMP2	1.617794822	0.66316768	2.674721842	0.013551444	0.243273341
ENSG00000198887	SMC5	1.0160195	5.890500059	2.765460563	0.01102551	0.229449642
ENSG00000164465	DCBLD1	-1.577865681	4.005096056	-2.67442474	0.013560546	0.243273341
ENSG00000134824	FADS2	-1.322886363	6.791311204	-2.77734399	0.010729776	0.228055323
ENSG00000138380	CARF	1.428277982	3.408473736	2.667923134	0.013761183	0.243428747
ENSG00000142207	URB1	0.925277797	6.828337717	2.785474342	0.010531789	0.227793619
ENSG00000103653	CSK	-0.975105845	6.901651578	-2.782844015	0.01059546	0.228055323
ENSG00000100626	GALNT16	-1.667873368	2.57261348	-2.657584936	0.014085978	0.246071908
ENSG00000058673	ZC3H11A	0.766369137	5.23348517	2.739179999	0.011707157	0.23562562
ENSG00000198056	PRIM1	-1.323310518	4.02831579	-2.676597858	0.013494101	0.243273341
ENSG00000162739	SLAMF6	-1.256884127	0.823420573	-2.676448542	0.013498656	0.243273341
ENSG00000123975	CKS2	-1.154656347	1.146470755	-2.668745886	0.01373564	0.243428747
ENSG00000124155	PIGT	-0.838333373	8.010961824	-2.797871308	0.010236555	0.226546946
ENSG00000162444	RBP7	1.56198718	2.910487774	2.652142774	0.014259837	0.24791533
ENSG00000185100	ADSSL1	-1.787660654	3.837403968	-2.666573939	0.013803168	0.243428747
ENSG00000155465	SLC7A7	-1.52680542	4.799542666	-2.702797199	0.012716791	0.240119332
ENSG00000172819	RARG	0.858012357	6.200926629	2.766786783	0.010992126	0.229449642
ENSG00000104447	TRPS1	1.497792202	5.204562849	2.734138712	0.011842389	0.23562562
ENSG00000151690	MFSD6	1.490669252	3.759818363	2.670324871	0.013686743	0.243273341
ENSG00000185022	MAFF	1.003695463	5.21208315	2.732286964	0.011892432	0.23590916
ENSG00000109610	SOD3	2.528504359	3.805461848	2.676618087	0.013493484	0.243273341
ENSG00000165548	TMEM63C	1.646617624	2.061456796	2.64490776	0.014494097	0.249371574
ENSG00000106683	LIMK1	-0.768529756	6.518408942	-2.764539234	0.011048757	0.229449642
ENSG00000151640	DPYSL4	-2.061090804	3.393251993	-2.647353444	0.014414507	0.249060408
ENSG00000169851	PCDH7	-1.771427442	2.10364889	-2.639456465	0.014672986	0.25079937
ENSG00000165863	C10orf82	1.223758059	0.64348565	2.661582869	0.013959529	0.245104601
ENSG00000143578	CREB3L4	1.377644282	4.595298086	2.699934822	0.012799614	0.240525449
ENSG00000003756	RBM5	0.819345949	6.939234362	2.774596755	0.010797469	0.228055323
ENSG00000117525	F3	-1.61600553	4.255465018	-2.670801892	0.013672004	0.243273341
ENSG00000175970	UNC119B	1.080454997	3.876456403	2.671217243	0.013659182	0.243273341
ENSG00000163956	LRPAP1	-0.750956952	7.431347872	-2.778413448	0.010703533	0.228055323
ENSG00000162407	PPAP2B	1.448510623	5.033062147	2.718647701	0.012267228	0.237635888
ENSG00000163898	LIPH	1.918935075	3.442001557	2.651502328	0.01428043	0.24791533
ENSG00000132749	MTL5	1.370237407	2.921169631	2.638228765	0.014713559	0.250819327
ENSG00000173614	NMNAT1	0.826680425	4.797750096	2.705219411	0.012647099	0.240119332
ENSG00000099866	MADCAM1	1.082838327	0.614931131	2.658626607	0.014052928	0.24582441
ENSG00000006047	YBX2	2.044042134	2.745624742	2.631962164	0.014922305	0.253172451
ENSG00000107968	MAP3K8	1.415547334	3.132909016	2.63957213	0.014669169	0.25079937
ENSG00000198721	ECI2	-1.509273365	3.990929499	-2.651599444	0.014277305	0.24791533
ENSG00000138764	CCNG2	1.187815999	5.436219215	2.726540009	0.012049015	0.236720555
ENSG00000160211	G6PD	-1.034806089	6.197230992	-2.745039556	0.011551809	0.234792022
ENSG00000164048	ZNF589	1.239501466	2.137536734	2.62343231	0.015210912	0.253762255
ENSG00000158246	FAM46B	1.611492105	1.392698199	2.630162344	0.01498277	0.253334753
ENSG00000136490	LIMD2	-1.532380309	4.43841542	-2.668424534	0.013745611	0.243428747
ENSG00000129657	SEC14L1	-0.966283908	5.385150836	-2.711771347	0.012460386	0.239152111
ENSG00000164520	RAET1E	1.42239991	1.596813628	2.623049083	0.015224001	0.253762255
ENSG00000122085	MTERFD2	0.997929645	4.303636339	2.672759941	0.013611657	0.243273341
ENSG00000105137	SYDE1	-1.400475014	4.818739553	-2.67995027	0.013392198	0.242932926
ENSG00000010292	NCAPD2	-1.082178425	7.83877274	-2.764292341	0.011054995	0.229449642
ENSG00000105329	TGFB1	-1.746408416	7.016443466	-2.753473616	0.011331625	0.232251022
ENSG00000167766	ZNF83	1.038920507	6.114961391	2.733899844	0.011848833	0.23562562
ENSG00000159763	PIP	1.518883924	0.583304261	2.634931355	0.014823054	0.252265233
ENSG00000079102	RUNX1T1	-1.571909433	1.689427265	-2.613538812	0.015552216	0.25472841
ENSG00000143614	GATAD2B	0.743229207	5.292670068	2.704266154	0.012674483	0.240119332
ENSG00000213901	SLC23A3	1.474302021	2.518770534	2.611563291	0.015621221	0.255059922
ENSG00000161203	AP2M1	-0.834261322	9.13344569	-2.772263736	0.010855273	0.228055323
ENSG00000008516	MMP25	1.157817715	1.124447489	2.617781218	0.015404992	0.25411309
ENSG00000164292	RHOBTB3	1.035485476	5.535525958	2.709486541	0.0125252	0.239252544

ENSG00000157388	CACNA1D	1.495464643	3.277731483	2.619020146	0.015362245	0.25379187
ENSG00000166509	CLEC3A	1.440708446	0.745255015	2.625324341	0.015146447	0.253762255
ENSG00000186710	CCDC42B	1.328529181	0.730187485	2.623633178	0.015204056	0.253762255
ENSG00000197283	SYNGAP1	1.136400661	5.133863281	2.690573863	0.013074041	0.242136245
ENSG00000166689	PLEKHA7	1.790993631	4.756791835	2.671648947	0.013645867	0.243273341
ENSG00000259494	MRPL46	0.921525588	0.385114037	2.633936637	0.014856235	0.252556
ENSG00000198670	LPA	1.025920041	0.571580214	2.624772472	0.015165223	0.253762255
ENSG00000248540	RP11-247C2.2	1.274824023	0.960004579	2.613747576	0.01554494	0.25472841
ENSG00000008853	RHOBTB2	-1.147744805	4.099077682	-2.626493076	0.015106754	0.253762255
ENSG00000132963	POMP	-0.821367995	6.841641676	-2.730265807	0.01194728	0.236399468
ENSG00000165312	OTUD1	-1.05945431	0.894630396	-2.622977341	0.015226452	0.253762255
ENSG00000100632	ERH	-0.992765723	5.471180982	-2.688641331	0.013131382	0.242207256
ENSG00000168078	PBK	-1.780346882	3.881461558	-2.61266996	0.01558253	0.254958785
ENSG00000122970	IFT81	1.036106382	4.986964996	2.675306114	0.01353356	0.243273341
ENSG00000166845	C18orf54	-1.213100777	1.529901656	-2.602331085	0.015947527	0.257344834
ENSG00000205670	SMIM11	1.084473983	3.774500221	2.620918821	0.01529695	0.253762255
ENSG00000147174	ACRC	1.297937433	2.311966574	2.587417712	0.016488151	0.259758883
ENSG00000198625	MDM4	0.997837872	5.726646471	2.701771145	0.012746422	0.240218464
ENSG00000151276	MAGI1	1.246976783	4.614263105	2.657192709	0.014098441	0.246071908
ENSG00000196275	GTF2IRD2	0.917322323	6.094431471	2.711117568	0.0124789	0.239152111
ENSG00000203852	HIST2H3A	1.323204249	0.744439005	2.608544714	0.015727216	0.255726162
ENSG00000204775	KM-PA-2	1.082838327	0.614931131	2.614337209	0.015524409	0.25472841
ENSG00000169239	CA5B	1.615305569	3.571549081	2.60666421	0.015793589	0.256274249
ENSG00000082684	SEMA5B	1.772978111	2.76670756	2.586454503	0.016523652	0.259758883
ENSG00000174326	SLC16A11	1.425672946	1.009886749	2.599611622	0.016044856	0.257686628
ENSG00000224877	C17orf89	-1.237436279	2.275559154	-2.582331868	0.016676408	0.259758883
ENSG00000122952	ZWINT	-1.235880149	5.711436209	-2.684666887	0.013250054	0.242859904
ENSG00000151917	BEND6	-1.545423826	2.394593282	-2.58247082	0.016671238	0.259758883
ENSG00000108821	COL1A1	-2.137286081	13.95750562	-2.77219387	0.010857009	0.228055323
ENSG00000197880	MDS2	1.163608441	1.274789616	2.587552467	0.01648319	0.259758883
ENSG00000138032	PPM1B	0.888348988	4.701224613	2.650350621	0.014317531	0.24823909
ENSG00000244731	C4A	-2.127731632	3.035889591	-2.588645163	0.016443015	0.259758883
ENSG00000084072	PPIE	-0.78925207	5.64612727	-2.680594626	0.013372695	0.242859904
ENSG00000105855	ITGB8	1.818138626	3.625650066	2.602354335	0.015946698	0.257344834
ENSG00000116199	FAM20B	1.023480621	5.166926184	2.669077411	0.01372536	0.243428747
ENSG00000163565	IFI16	-1.300518316	6.164973648	-2.69461735	0.012954829	0.241908226
ENSG00000165272	AQP3	2.358196274	4.262358513	2.628766224	0.015029832	0.253729392
ENSG00000105497	ZNF175	1.309613434	1.326405678	2.58260565	0.016666223	0.259758883
ENSG00000189320	FAM180A	1.895939549	2.125555801	2.578833732	0.016807059	0.260335211
ENSG00000124006	OBSL1	0.9907259	7.464391473	2.727548302	0.012021403	0.236672314
ENSG00000158406	HIST1H4H	1.594410919	2.255480701	2.576545076	0.016893056	0.261409208
ENSG00000196482	ESRRG	1.363204792	1.189821672	2.584319614	0.016602592	0.259758883
ENSG00000074181	NOTCH3	-1.191686547	8.534438626	-2.736074492	0.011790288	0.23562562
ENSG00000187091	PLCD1	1.29739963	5.587729678	2.682220096	0.013323615	0.242859904
ENSG00000152475	ZNF837	1.221716313	0.916653661	2.592180417	0.016313661	0.259758883
ENSG00000091622	PITPNM3	1.616475503	3.003907704	2.579687131	0.016775098	0.260146406
ENSG00000187742	SECISBP2	0.830047946	5.986183386	2.69424712	0.012965701	0.241908226
ENSG00000070731	ST6GALNAC2	1.605735213	4.331604999	2.62803247	0.015054621	0.253729392
ENSG00000266967	AARSD1	1.26178408	3.038147232	2.580360334	0.016749925	0.260146406
ENSG00000159063	ALG8	-0.817993225	6.077123158	-2.689994171	0.013091217	0.242136245
ENSG00000175691	ZNF77	1.424693199	1.512871855	2.574511556	0.01696981	0.262080014
ENSG00000139793	MBNL2	1.203223226	4.459796882	2.630499219	0.014971436	0.253334753
ENSG00000169084	DHRSX	-1.139155769	4.64376687	-2.630166632	0.014982626	0.253334753
ENSG00000132259	CNGA4	0.95141214	0.385114037	2.607711412	0.015756595	0.255938649
ENSG00000067225	PKM	-0.866699238	9.920719377	-2.746410056	0.011515757	0.234362835
ENSG00000119714	GPR68	-1.653757335	1.416878705	-2.582082044	0.016685707	0.259758883
ENSG00000107331	ABCA2	1.126026993	7.333905645	2.720904192	0.012204461	0.236720555
ENSG00000172243	CLEC7A	-1.525629842	4.600339846	-2.617344506	0.015420086	0.25411309
ENSG00000054282	SDCCAG8	0.755157993	6.145882318	2.695633822	0.012925022	0.241908226
ENSG00000162511	LAPTM5	-1.808777007	7.153903064	-2.710447133	0.012497912	0.239152111
ENSG00000213445	SIPA1	-1.186560234	6.284881265	-2.692884571	0.01300579	0.242136245
ENSG00000072210	ALDH3A2	0.943772133	6.008210564	2.690293588	0.013082343	0.242136245
ENSG00000116580	GON4L	0.680954666	6.89352118	2.709189269	0.012533656	0.239252544

ENSG00000000938	FGR	-1.503909661	3.149675624	-2.570040564	0.017139715	0.262859727
ENSG00000074047	GLI2	-1.663169802	2.789855935	-2.564435902	0.017354947	0.264015838
ENSG00000135049	AGTPBP1	1.00959016	4.90884125	2.645573015	0.014472407	0.24927172
ENSG00000186854	TRABD2A	-1.18729762	1.816132174	-2.566111851	0.017290323	0.263656429
ENSG00000146112	PPP1R18	-1.077168047	5.014974596	-2.640508149	0.014638313	0.250753029
ENSG00000077809	GTF2I	0.713107554	7.983411336	2.723026511	0.012145703	0.236720555
ENSG00000168646	AXIN2	-1.597079558	2.440968812	-2.560503945	0.017507448	0.264364464
ENSG00000169413	RNASE6	-1.386961057	1.070267468	-2.585108936	0.016573365	0.259758883
ENSG00000197857	ZNF44	1.527205232	4.08536265	2.603770011	0.015896253	0.257344834
ENSG00000172531	PPP1CA	-0.812282386	7.540217523	-2.712690244	0.012434409	0.239103321
ENSG00000213203	GIMAP1	-1.213449669	1.189821672	-2.579601804	0.016778291	0.260146406
ENSG00000147687	TATDN1	1.527324929	3.647438886	2.586677738	0.016515418	0.259758883
ENSG00000104879	CKM	-1.204281568	0.708164219	-2.595666702	0.016187033	0.259704224
ENSG00000067704	IARS2	0.718945543	7.679014378	2.716441541	0.012328889	0.238242118
ENSG00000204472	AIF1	-1.582904711	4.908471563	-2.627408281	0.015075739	0.253762255
ENSG00000150977	RILPL2	-1.32232715	3.446198346	-2.568782226	0.01718782	0.262859727
ENSG00000135114	OASL	-1.772097477	3.726596362	-2.572723555	0.017037567	0.262508881
ENSG00000137474	MYO7A	-1.51916193	3.730381668	-2.574512692	0.016969767	0.262080014
ENSG00000165794	SLC39A2	1.181542847	0.52892491	2.584722847	0.016587655	0.259758883
ENSG00000119138	KLF9	1.639928817	1.889423183	2.560285414	0.017515961	0.264364464
ENSG00000104549	SQLE	1.345472403	6.261893855	2.685967505	0.013211109	0.242734786
ENSG00000120008	WDR11	0.883184306	6.455973475	2.690175882	0.013085831	0.242136245
ENSG00000166979	EVA1C	1.358539728	4.095438748	2.600603165	0.016009305	0.2573791
ENSG00000185019	UBOX5	0.997513454	3.751189843	2.585818275	0.01654714	0.259758883
ENSG00000132549	VPS13B	0.935475943	5.784573832	2.671306162	0.013656438	0.243273341
ENSG00000138028	CGREF1	1.733553031	3.990020033	2.601269033	0.015985472	0.257344834
ENSG00000110719	TCIRG1	-1.366831871	7.240914195	-2.698718905	0.012834951	0.240900976
ENSG00000196890	HIST3H2BB	0.986891631	0.385114037	2.589774073	0.016401605	0.259758883
ENSG00000188626	GOLGA8M	1.303477938	1.191359461	2.562496066	0.017430028	0.264364464
ENSG00000106236	NPTX2	-2.025842142	1.149095126	-2.569395077	0.017164375	0.262859727
ENSG00000204175	GPRIN2	1.587723691	2.206653568	2.546655691	0.018054578	0.268485152
ENSG00000112981	NME5	1.574881783	0.921539293	2.564183655	0.017364693	0.264015838
ENSG00000074706	IPCEF1	-1.231962142	1.003355496	-2.572454914	0.017047769	0.262508881
ENSG00000129277	CCL4	-1.524981564	1.500727685	-2.563756805	0.017381197	0.264015838
ENSG00000130303	BST2	-1.86021775	6.735677586	-2.678446012	0.013437833	0.243273341
ENSG00000149554	CHEK1	-1.102775732	4.960160163	-2.615552505	0.015482171	0.254377742
ENSG00000184922	FMNL1	-1.140701732	5.78836355	-2.652798334	0.014238788	0.24791533
ENSG00000136943	CTSV	2.403751897	4.483042453	2.610158325	0.015670472	0.255332684
ENSG00000185905	C16orf54	-1.247760465	1.526629976	-2.55429862	0.017750671	0.266601259
ENSG00000139734	DIAPH3	-1.373760421	4.428814693	-2.587704532	0.016477594	0.259758883
ENSG00000102218	RP2	-1.178616133	3.513068236	-2.55603012	0.017682488	0.266272472
ENSG00000188112	C6orf132	1.830553625	3.995534681	2.581600804	0.016703633	0.259758883
ENSG00000104369	JPH1	1.411018879	2.387983541	2.539659853	0.018337005	0.271695889
ENSG00000253320	KB-1507C5.2	1.583453284	1.223261823	2.549059343	0.017958482	0.267720573
ENSG00000183520	UTP11L	-0.829622477	5.789978109	-2.649828184	0.014334391	0.248257088
ENSG00000198682	PAPSS2	1.253989548	5.448429387	2.642632034	0.014568526	0.25010366
ENSG00000180758	GPR157	1.232142742	4.290293644	2.589619443	0.016407271	0.259758883
ENSG00000159905	ZNF221	1.132595471	1.05976892	2.549956151	0.01792275	0.267680294
ENSG00000180739	S1PR5	-1.282645896	0.99774258	-2.561196489	0.017480497	0.264364464
ENSG00000167552	TUBA1A	-0.974185943	7.068098698	-2.68186945	0.013334188	0.242859904
ENSG00000169203	RP11-231C14.4	0.917950508	4.940530549	2.61941692	0.015348578	0.25379187
ENSG00000073670	ADAM11	1.485020506	2.186152249	2.536110285	0.018481875	0.272811944
ENSG00000131773	KHDRBS3	1.350026775	5.661175757	2.647950591	0.014395137	0.249034284
ENSG00000140450	ARRDC4	1.058348332	3.686344919	2.559696139	0.017538934	0.264364464
ENSG00000239305	RNF103	1.378788389	1.268258363	2.539224966	0.018354697	0.271701465
ENSG00000105011	ASF1B	-1.798841631	3.866864375	-2.549786287	0.017929513	0.267680294
ENSG00000168795	ZBTB5	1.395709694	2.027969459	2.525988824	0.018900847	0.275402745
ENSG00000172915	NBEA	-1.441273404	2.560351258	-2.532843645	0.01861614	0.2737328
ENSG00000167094	TTC16	1.354695461	0.845443839	2.547192882	0.01803306	0.268459908
ENSG00000116898	MRPS15	-0.900386895	6.753406327	-2.664462849	0.013869102	0.244242519
ENSG00000108381	ASPA	1.513131921	1.728582509	2.525403312	0.018925353	0.275402745
ENSG00000198853	RUSC2	0.99942805	5.632673265	2.63797048	0.014722108	0.250819327
ENSG00000148926	ADM	-1.270827986	3.958259032	-2.549929559	0.017923809	0.267680294

ENSG00000039068	CDH1	2.706495828	5.936661309	2.646624661	0.014438181	0.249060408
ENSG00000173531	MST1	1.60058335	3.062090879	2.531678912	0.018664232	0.2737328
ENSG00000030582	GRN	-1.06224154	8.895936918	-2.691929184	0.013033968	0.242136245
ENSG00000163681	SLMAP	0.994423871	5.770084337	2.63879287	0.014694903	0.250819327
ENSG00000122585	NPY	2.129989443	1.475974547	2.524719418	0.018954014	0.275402745
ENSG00000170631	ZNF16	1.097623193	3.327075205	2.536427862	0.01846887	0.272811944
ENSG00000156170	NDUFAF6	0.770907263	5.381323622	2.622372632	0.01524713	0.253762255
ENSG00000169504	CLIC4	1.132897988	6.13097473	2.646437534	0.014444266	0.249060408
ENSG00000155659	VSIG4	-2.444935061	3.818924052	-2.532413894	0.018633871	0.2737328
ENSG00000102547	CAB39L	1.429615702	3.946214547	2.551962792	0.01784304	0.267151856
ENSG00000156966	B3GNT7	1.450391385	1.120149639	2.524646915	0.018957054	0.275402745
ENSG00000053918	KCNQ1	-1.2887853	3.435952522	-2.523247465	0.019015838	0.275402745
ENSG00000126870	WDR60	0.819904508	6.166103172	2.643522282	0.014539367	0.249876467
ENSG00000172803	SNX32	1.980109711	1.702976598	2.514378845	0.019392328	0.278492975
ENSG00000101276	SLC52A3	1.594087518	2.567777455	2.506748464	0.019721795	0.278718352
ENSG00000109819	PPARGC1A	1.899544013	2.368034348	2.511387979	0.019520853	0.278673807
ENSG00000169231	THBS3	1.117781527	7.029944761	2.661411833	0.013964917	0.245104601
ENSG00000148848	ADAM12	-1.721919192	5.588876146	-2.612151064	0.01560066	0.254989543
ENSG00000185739	SRL	1.057282708	0.751515136	2.533913782	0.018572055	0.273371592
ENSG00000085465	OVGP1	1.596995632	2.243332044	2.504353137	0.019826291	0.278718352
ENSG00000114378	HYAL1	1.700097105	2.360277697	2.510547812	0.0195571	0.278673807
ENSG00000165389	SPTSSA	1.074706637	4.687227946	2.58167053	0.016701035	0.259758883
ENSG00000150630	VEGFC	-1.472761281	3.360323896	-2.513650803	0.019423541	0.278492975
ENSG00000150551	LYPD1	-1.095632578	0.658282048	-2.543212227	0.018193082	0.269818195
ENSG00000171817	ZNF540	1.352861789	1.715748381	2.503201051	0.019876734	0.278718352
ENSG00000129450	SIGLEC9	-1.661775748	2.445536147	-2.500167674	0.020010119	0.27934055
ENSG00000163683	SMIM14	0.897082419	5.913525875	2.628434589	0.015041031	0.253729392
ENSG00000169217	CD2BP2	-0.868156194	6.57324797	-2.640532748	0.014637503	0.250753029
ENSG00000119280	C1orf198	1.077852236	5.305129169	2.604823304	0.015858818	0.25706685
ENSG00000117122	MFAP2	-1.328286671	7.502451109	-2.65870789	0.014050352	0.24582441
ENSG00000122122	SASH3	-1.663661052	2.820400314	-2.504278765	0.019829544	0.278718352
ENSG00000054179	ENTPD2	1.696284453	3.120059954	2.509289459	0.019611505	0.278718352
ENSG00000100399	CHADL	2.024934357	2.019496822	2.501610832	0.019946556	0.279038377
ENSG00000166825	ANPEP	-1.763733971	6.244049474	-2.620383558	0.015315331	0.253762255
ENSG00000176871	WSB2	-0.88493287	5.979033164	-2.616484034	0.015449869	0.25411309
ENSG00000065978	YBX1	-0.950011454	5.404853389	-2.592248212	0.01631119	0.259758883
ENSG00000122390	NAA60	1.083782145	0.75804639	2.516666663	0.019294547	0.278049106
ENSG00000256618	MTRNR2L1	1.24501674	1.298769497	2.498612658	0.020078819	0.279928073
ENSG00000100385	IL2RB	-1.757635937	2.712259232	-2.490596931	0.020436444	0.282483296
ENSG00000007384	RHBDF1	1.124452511	6.056941326	2.621709133	0.015269848	0.253762255
ENSG00000072422	RHOBTB1	-1.267830388	3.795462253	-2.512977616	0.019452444	0.278492975
ENSG00000054523	KIF1B	0.758334101	6.508859386	2.631888745	0.014924767	0.253172451
ENSG00000164707	SLC13A4	1.628180709	1.712629222	2.490275442	0.02045091	0.282483296
ENSG00000165168	CYBB	-1.805313941	4.486998883	-2.535145386	0.01852144	0.273139012
ENSG00000154645	CHODL	1.681880212	1.617521828	2.486902219	0.020603271	0.283809756
ENSG00000214106	PAXIP1-AS2	-1.086826401	1.670783512	-2.49047955	0.020441725	0.282483296
ENSG00000197497	ZNF665	0.965100063	3.413450672	2.503247267	0.019874708	0.278718352
ENSG00000176593	CTD-2368P22.1	1.203624926	3.452104397	2.504156602	0.019834888	0.278718352
ENSG00000146859	TMEM140	1.106801099	0.780069656	2.504991057	0.019798412	0.278718352
ENSG00000157613	CREB3L1	-2.409919017	5.54220793	-2.585578344	0.016556006	0.259758883
ENSG00000165695	AK8	1.281274626	1.867128785	2.47803848	0.021008667	0.285161758
ENSG00000139219	COL2A1	3.613056723	5.378091729	2.585825474	0.016546874	0.259758883
ENSG00000135643	KCNMB4	-1.512914005	1.117188952	-2.501236418	0.019963029	0.279038377
ENSG00000135250	SRPK2	0.974403037	5.035612994	2.57028879	0.01713024	0.262859727
ENSG00000177700	POLR2L	-0.864302343	6.719244441	-2.621413507	0.01527998	0.253762255
ENSG00000084623	EIF3I	-0.820705102	8.65374211	-2.651420653	0.014283058	0.24791533
ENSG00000214113	LYRM4	-1.066585841	4.587728014	-2.530597504	0.018708987	0.274101511
ENSG00000114529	C3orf52	1.489735501	1.25174018	2.484550866	0.020710099	0.283809756
ENSG00000224963	U82695.9	1.579520595	0.598319382	2.501062387	0.01997069	0.279038377
ENSG00000145934	TENM2	-2.340013464	2.238851634	-2.473602213	0.021214336	0.286492423
ENSG00000141232	TOB1	1.160943029	3.197160307	2.48605464	0.02064172	0.283809756
ENSG00000101213	PTK6	2.018036763	3.109830619	2.48149483	0.020849713	0.283971801
ENSG00000224474	AL355490.1	1.027339078	0.571580214	2.505829297	0.019761833	0.278718352

ENSG00000112414	GPR126	1.84694526	3.224887014	2.481759428	0.02083759	0.283971801
ENSG00000198429	ZNF69	1.542006098	3.094969717	2.476505984	0.021079504	0.285875977
ENSG00000175265	GOLGA8A	2.041356461	5.210137071	2.571190685	0.017095857	0.262733689
ENSG00000008226	DLEC1	1.774832568	1.85886364	2.465211485	0.021608456	0.287600111
ENSG00000141527	CARD14	1.573102982	4.001278326	2.509635964	0.01959651	0.278718352
ENSG00000150593	PDCD4	1.038578973	6.850178846	2.620426932	0.015313841	0.253762255
ENSG00000145536	ADAMTS16	2.108512097	3.177207575	2.485737064	0.020656143	0.283809756
ENSG00000090776	EFNB1	-1.383464189	5.381466929	-2.568128316	0.017212868	0.262986473
ENSG00000159648	TEPP	-1.109944513	0.715391087	-2.503734306	0.019853371	0.278718352
ENSG00000111666	CHPT1	1.136490133	6.091176922	2.601590163	0.01597399	0.257344834
ENSG00000245680	ZNF585B	1.159375979	2.586949163	2.463998919	0.02166597	0.287614838
ENSG00000184674	GSTT1	2.491666871	3.048996157	2.482598078	0.020799211	0.283971801
ENSG00000198435	NRARP	1.130867102	0.794866054	2.483551917	0.02075564	0.283971801
ENSG00000100307	CBX7	1.30943661	3.778749527	2.498004988	0.020105725	0.279928073
ENSG00000188785	ZNF548	1.263661904	2.758824904	2.467959535	0.021478636	0.287600111
ENSG00000136928	GABBR2	2.77567411	2.32294347	2.465163938	0.021610709	0.287600111
ENSG00000071553	ATP6AP1	-0.701730343	7.475557813	-2.621079322	0.015291442	0.253762255
ENSG00000166523	CLEC4E	-1.639982055	2.126195183	-2.47155707	0.021309779	0.28652031
ENSG00000164045	CDC25A	-1.398761583	3.032551778	-2.46063266	0.021826382	0.289010772
ENSG00000110801	PSMD9	0.882153456	3.799346229	2.492696694	0.020342194	0.282218719
ENSG00000100239	PPP6R2	0.714677009	7.362326614	2.618921583	0.015365641	0.25379187
ENSG00000002834	LASP1	-0.930945208	6.312159187	-2.591787567	0.016327988	0.259758883
ENSG00000181830	SLC35C1	-1.187029238	2.988462504	-2.454752077	0.022109254	0.291820833
ENSG00000173198	CYSLTR1	-1.307925451	1.685237247	-2.465247137	0.021606767	0.287600111
ENSG00000110077	MS4A6A	-1.428538085	6.806231355	-2.601120855	0.015990773	0.257344834
ENSG00000198502	HLA-DRB5	-1.69907508	6.530761503	-2.591335169	0.016344502	0.259758883
ENSG00000175193	PARL	-0.74315557	6.098491106	-2.583582183	0.016629941	0.259758883
ENSG00000175063	UBE2C	-1.770691485	4.8093148	-2.513141621	0.019445399	0.278492975
ENSG00000026950	BTN3A1	-1.165463775	5.128958857	-2.534049274	0.018566481	0.273371592
ENSG00000013275	PSMC4	-0.746056923	7.352964763	-2.609622578	0.015689291	0.255374128
ENSG00000132481	TRIM47	1.06633386	5.521669564	2.565451412	0.017315763	0.263787993
ENSG00000215845	TSTD1	1.231605208	4.083794069	2.495219202	0.020229503	0.280903663
ENSG00000102878	HSF4	1.49202769	5.577704018	2.567316924	0.017243996	0.263205773
ENSG00000197459	HIST1H2BH	0.995334392	0.445494755	2.484949159	0.020691968	0.283809756
ENSG00000167900	TK1	-1.732274761	5.553257795	-2.545870136	0.018086088	0.268485152
ENSG00000111716	LDHB	-1.3486232	7.738842299	-2.610589523	0.015655341	0.255332684
ENSG00000142208	AKT1	-0.752920234	7.952292405	-2.616573161	0.015446781	0.25411309
ENSG00000131042	LILRB2	-1.648574545	4.343087472	-2.491138308	0.020412105	0.282483296
ENSG00000105173	CCNE1	-1.625030172	4.182069154	-2.48227467	0.020814003	0.283971801
ENSG00000034677	RNF19A	1.000201086	5.483347585	2.56004719	0.017525244	0.264364464
ENSG00000072571	HMMR	-1.397991619	4.140529047	-2.481124149	0.020866706	0.283971801
ENSG00000102174	PHEX	-1.821140716	2.335702701	-2.44218524	0.022725179	0.295672287
ENSG00000181722	ZBTB20	1.342298931	3.982881578	2.490267245	0.020451279	0.282483296
ENSG00000168356	SCN11A	1.194850493	0.987863484	2.462937969	0.021716409	0.288040725
ENSG00000017797	RALBP1	0.92943845	4.660365137	2.52048185	0.019132507	0.276434773
ENSG00000130720	FIBCD1	-1.23336538	1.17003181	-2.464408405	0.021646532	0.287600111
ENSG00000023287	RB1CC1	0.893690821	7.032280837	2.601202637	0.015987847	0.257344834
ENSG00000149295	DRD2	1.775971382	1.577864991	2.44293167	0.022688155	0.295672287
ENSG00000132196	HSD17B7	1.236296709	3.018540449	2.446684253	0.02250287	0.294647798
ENSG00000120963	ZNF706	1.111320622	3.889265069	2.479626321	0.020935504	0.284661177
ENSG00000166426	CRABP1	2.786916943	3.36826288	2.461808258	0.021770237	0.2885108
ENSG00000101400	SNTA1	-0.960367304	4.838806173	-2.515937847	0.019325646	0.278049106
ENSG00000178209	PLEC	1.216801901	9.169747085	2.62433997	0.015179954	0.253762255
ENSG00000186868	MAPT	1.583149358	1.990454181	2.440635661	0.022802217	0.296183438
ENSG00000092208	GEMIN2	-0.98098661	3.991474919	-2.470780208	0.021346137	0.28652031
ENSG00000064270	ATP2C2	2.003564512	2.303415003	2.430520142	0.02331109	0.298598986
ENSG00000182111	ZNF716	1.088597423	0.751515136	2.464936595	0.021621482	0.287600111
ENSG00000180071	ANKRD18A	1.364939107	2.097038671	2.429322219	0.023372044	0.298640667
ENSG00000085871	MGST2	-1.326724862	5.034855082	-2.51576945	0.019332839	0.278049106
ENSG00000133392	MYH11	1.895183341	4.72993409	2.517822533	0.01924532	0.277808936
ENSG00000198298	ZNF485	1.144226903	1.103119838	2.448028415	0.022436843	0.294607951
ENSG00000164211	STARD4	-1.357032336	1.974739445	-2.434406156	0.023114369	0.298508285
ENSG00000147144	CCDC120	1.153873866	3.970276457	2.474942029	0.021152025	0.28636447

ENSG00000136141	LRCH1	-1.170588121	4.433657343	-2.484934998	0.020692612	0.283809756
ENSG00000008735	MAPK8IP2	2.116040874	3.887892512	2.470932831	0.02133899	0.28652031
ENSG00000257093	KIAA1147	1.063688956	3.885011019	2.471829962	0.02129702	0.28652031
ENSG00000115919	KYNU	-2.405673558	3.380205196	-2.442310937	0.02271894	0.295672287
ENSG00000142961	MOB3C	-1.13548831	2.53616417	-2.424737259	0.023606704	0.299929392
ENSG00000161638	ITGA5	-1.506607466	7.472409207	-2.591098079	0.016353162	0.259758883
ENSG00000118298	CA14	1.718767668	1.735809376	2.43205741	0.023233085	0.298554465
ENSG00000105357	MYH14	2.638030812	7.124239129	2.588860205	0.016435119	0.259758883
ENSG00000176533	GNG7	1.410084064	4.309981861	2.488899738	0.020512921	0.282836768
ENSG00000104763	ASAH1	-0.858066794	7.620177972	-2.592262933	0.016310653	0.259758883
ENSG00000133055	MYBPH	1.5365722	1.309268044	2.432327551	0.023219402	0.298554465
ENSG00000148400	NOTCH1	1.187815753	6.906275512	2.582803833	0.016658853	0.259758883
ENSG00000150938	CRIM1	1.510748735	5.670982435	2.548862455	0.017966335	0.267720573
ENSG00000171862	PTEN	1.295846598	2.804421462	2.424061318	0.023641483	0.300128246
ENSG00000104880	ARHGEF18	1.272183187	1.19704854	2.431701269	0.023251135	0.298554465
ENSG00000166682	TMPRSS5	1.542812707	1.886499821	2.418882734	0.023909509	0.300919502
ENSG00000183072	NKX2-5	-1.428912578	1.055889896	-2.445587276	0.022556888	0.294950109
ENSG00000102054	RBBP7	-0.876393353	8.199945684	-2.594919665	0.016214089	0.259758883
ENSG00000099968	BCL2L13	0.984863537	4.487509794	2.48933054	0.020493484	0.282817282
ENSG00000233822	HIST1H2BN	1.459660229	1.672933057	2.417615032	0.023975548	0.300960257
ENSG00000214595	EML6	1.276809185	2.207332117	2.409643928	0.024394664	0.303309572
ENSG00000007264	MATK	-1.452680411	3.482734015	-2.427429809	0.023468636	0.299387285
ENSG00000083223	ZCCHC6	0.796822828	5.875182942	2.546030387	0.018079656	0.268485152
ENSG00000115590	IL1R2	-1.535321261	1.68629104	-2.431830482	0.023244584	0.298554465
ENSG00000106066	CPVL	-1.679349382	5.726228871	-2.52886498	0.018780898	0.274642196
ENSG00000197183	C20orf112	0.899098618	4.773634359	2.497340106	0.020135203	0.28008996
ENSG00000184702	SEPT5	1.174611087	6.849174959	2.569002532	0.017179389	0.262859727
ENSG00000140279	DUOX2	1.696392699	2.353932563	2.405541596	0.024612991	0.304136669
ENSG00000160193	WDR4	1.121983058	4.907324052	2.502255081	0.019918242	0.279038377
ENSG00000110042	DTX4	-1.721080373	3.208476664	-2.41327928	0.024202687	0.30211962
ENSG00000167487	KLHL26	1.233844182	1.654720457	2.405480624	0.024616249	0.304136669
ENSG00000242866	STRC	1.608269196	1.429592436	2.408394075	0.024460992	0.303412979
ENSG00000164056	SPRY1	-1.215920795	2.467194333	-2.398605268	0.024986251	0.307738826
ENSG00000198455	ZXDB	-0.985888468	0.614931131	-2.446437908	0.02251499	0.294647798
ENSG00000109881	CCDC34	-1.105397909	4.832583182	-2.478896874	0.020969085	0.284870926
ENSG00000178999	AURKB	-1.515079684	5.094397688	-2.484829186	0.020697428	0.283809756
ENSG00000013619	MAMLD1	1.525550063	2.090857515	2.396363411	0.025108003	0.308511885
ENSG00000183741	CBX6	1.139669909	5.064122956	2.503463705	0.019865224	0.278718352
ENSG00000196126	HLA-DRB1	-1.658248982	8.55317884	-2.582822406	0.016658163	0.259758883
ENSG00000052795	FNIP2	-1.043047753	3.902615844	-2.430929071	0.023290316	0.298598986
ENSG00000123473	STIL	-1.053581528	4.712796681	-2.469188401	0.021420817	0.287276963
ENSG00000250673	RP11-6L6.2	1.024055447	0.421933701	2.440644721	0.022801766	0.296183438
ENSG00000090905	TNRC6A	0.759778762	7.043757107	2.560751307	0.017497817	0.264364464
ENSG00000162576	MXRA8	-1.804366582	7.941864014	-2.571761172	0.017074142	0.262657215
ENSG00000104368	PLAT	-1.612544072	5.631971792	-2.511462658	0.019517635	0.278673807
ENSG00000006756	ARSD	-1.049903842	5.512987985	-2.503545187	0.019861654	0.278718352
ENSG00000174080	CTSF	0.99757114	6.775741123	2.552912853	0.017805416	0.266843161
ENSG00000198400	NTRK1	-1.274998374	1.131674357	-2.421065007	0.023796222	0.30085063
ENSG00000124159	MATN4	2.299335425	2.425671956	2.407487718	0.024509194	0.303530991
ENSG00000184207	PGP	-1.249902014	2.861447755	-2.390932186	0.025405238	0.30911855
ENSG00000203930	LINC00632	-1.301847026	0.782721884	-2.429908174	0.02334221	0.298598986
ENSG00000115137	DNAJC27	1.263942802	1.592004159	2.391397975	0.02537962	0.30911855
ENSG00000087510	TFAP2C	1.75648001	4.506897969	2.464462637	0.021643959	0.287600111
ENSG00000131236	CAP1	-0.729795209	6.131956716	-2.526422813	0.018882702	0.275402745
ENSG00000215218	UBE2QL1	-1.358797914	1.031910015	-2.41633978	0.02404215	0.301314195
ENSG00000124357	NAGK	-0.713767353	7.329365214	-2.55502598	0.017721999	0.266601259
ENSG00000128815	WDFY4	-1.604940976	3.954062712	-2.418413271	0.023933945	0.300919502
ENSG00000189283	FHIT	1.135048629	2.919802278	2.394788512	0.025193861	0.308962875
ENSG00000162819	BROX	0.942064479	5.602165639	2.510995671	0.019537771	0.278673807
ENSG00000172935	MRGPRF	-1.234624352	0.782721884	-2.421665214	0.02376515	0.30085063
ENSG00000149273	RPS3	-1.043480184	7.972040002	-2.559898082	0.017531058	0.264364464
ENSG00000196549	MME	-2.638891461	4.393819085	-2.425884753	0.023547772	0.299748964
ENSG00000156469	MTERFD1	0.896402615	6.027711061	2.52251133	0.019046827	0.275450476

ENSG00000087245	MMP2	-1.174701253	9.19809306	-2.57278133	0.017035374	0.262508881
ENSG00000163644	PPM1K	1.068863009	5.713179755	2.510211292	0.019571636	0.278673807
ENSG00000071073	MGAT4A	-1.151129839	4.382401944	-2.43205222	0.023233348	0.298554465
ENSG00000090006	LTBP4	1.580179241	7.477460985	2.553127326	0.017796933	0.266843161
ENSG00000007062	PROM1	2.801725854	5.636671117	2.506533525	0.019731151	0.278718352
ENSG00000009780	FAM76A	0.89957816	4.387516104	2.444895687	0.022591006	0.295150265
ENSG00000117222	RBBP5	0.631727204	5.615967768	2.503453212	0.019865683	0.278718352
ENSG00000058668	ATP2B4	1.162331543	6.487172135	2.531627063	0.018666375	0.2737328
ENSG00000136826	KLF4	1.009477563	4.25879445	2.439781278	0.022844797	0.296246044
ENSG00000156467	UQCRB	1.117643503	4.894120625	2.471217638	0.021325658	0.28652031
ENSG00000137522	RNF121	-0.875660025	4.585855304	-2.442843077	0.022692547	0.295672287
ENSG00000123416	TUBA1B	-0.980964871	7.987280135	-2.554181446	0.017755294	0.266601259
ENSG00000133083	DCLK1	-1.528187402	3.046298867	-2.374917598	0.026300659	0.315232739
ENSG00000185008	ROBO2	-2.182121916	2.128446629	-2.368229318	0.026683153	0.315768956
ENSG00000187554	TLR5	1.296604589	2.45604858	2.365480035	0.026841861	0.315768956
ENSG00000127252	HRASLS	1.506058021	1.793956692	2.371369932	0.026502912	0.315768956
ENSG00000162735	PEX19	1.352546602	1.590612931	2.37218743	0.02645618	0.315768956
ENSG00000151491	EPS8	-1.055316384	6.050676066	-2.505098793	0.019793707	0.278718352
ENSG00000137496	IL18BP	-1.099533377	3.414079842	-2.381740717	0.025915662	0.312206131
ENSG00000197993	KEL	1.586125462	1.429481191	2.375340025	0.026276671	0.315232739
ENSG00000012822	CALCOCO1	0.697637907	6.444114591	2.523006539	0.019025975	0.275402745
ENSG00000185650	ZFP36L1	-0.831853854	6.447258783	-2.516593464	0.019297668	0.278049106
ENSG00000134917	ADAMTS8	1.33513859	1.031214401	2.385072999	0.02572953	0.311067838
ENSG00000091129	NRCAM	1.849984707	3.409878367	2.394246563	0.025223468	0.308962875
ENSG00000185950	IRS2	1.694392236	2.483679106	2.361755332	0.027058263	0.315768956
ENSG00000130429	ARPC1B	-0.833265719	7.519971661	-2.537753247	0.018414688	0.272332579
ENSG00000198189	HSD17B11	-1.151347797	3.496201742	-2.381589753	0.025924124	0.312206131
ENSG00000154099	DNAAF1	1.2589882	1.679548168	2.362522342	0.02701357	0.315768956
ENSG00000161798	AQP5	2.526481011	3.000634547	2.374613394	0.026317946	0.315232739
ENSG00000132517	SLC52A1	1.827315853	2.804631892	2.370864805	0.026531826	0.315768956
ENSG00000136108	CKAP2	-1.101697644	4.715473819	-2.433079492	0.023181355	0.298554465
ENSG00000172348	RCAN2	-1.345808657	2.423304043	-2.359874146	0.027168167	0.315768956
ENSG00000145029	NICN1	1.062892975	4.306186312	2.426150497	0.023534143	0.299748964
ENSG00000166450	PRTG	-1.242126678	0.730187485	-2.404572654	0.024664821	0.304497013
ENSG00000162813	BPNT1	0.818254049	6.349772533	2.513542579	0.019428185	0.278492975
ENSG00000126264	HCST	-1.302020265	2.563204112	-2.35631327	0.027377328	0.316331118
ENSG00000163171	CDC42EP3	1.200184067	2.884220404	2.368536306	0.026665486	0.315768956
ENSG00000119929	CUTC	0.898679901	4.332292396	2.425059359	0.023590148	0.299929392
ENSG00000128487	SPECC1	-1.228376604	4.946555793	-2.446580655	0.022507966	0.294647798
ENSG00000143442	POGZ	0.708508698	7.080124078	2.526265001	0.018889298	0.275402745
ENSG00000198900	TOP1	-0.666684009	7.474861738	-2.530126782	0.0187285	0.274131428
ENSG00000204790	CBWD6	1.032738238	3.73046813	2.391340581	0.025382775	0.30911855
ENSG00000168615	ADAM9	-0.890541174	7.292530624	-2.525704649	0.018912737	0.275402745
ENSG00000186160	CYP4Z1	1.164836532	0.445494755	2.394561219	0.025206274	0.308962875
ENSG00000257743	RP11-1220K2.2	1.621327043	1.33558916	2.360617753	0.027124675	0.315768956
ENSG00000183955	SETD8	1.179990253	2.651297198	2.35886143	0.027227503	0.315768956
ENSG00000204498	NFKBIL1	-1.175332983	2.839100515	-2.350029842	0.027750024	0.318857312
ENSG00000087086	FTL	-0.970618119	6.310261413	-2.498130463	0.020100167	0.279928073
ENSG00000018625	ATP1A2	1.793215275	1.548545408	2.359497442	0.027190225	0.315768956
ENSG00000116299	KIAA1324	2.238690236	5.684441448	2.482986639	0.020781451	0.283971801
ENSG00000154263	ABCA10	1.475589708	1.529901656	2.352939518	0.027576863	0.318402176
ENSG00000089847	ANKRD24	1.392728662	1.64638351	2.350707586	0.027709601	0.318857312
ENSG00000129467	ADCY4	1.185913792	4.510007008	2.427787717	0.023450339	0.299387285
ENSG00000075213	SEMA3A	-2.1304689	2.335003773	-2.350006071	0.027751442	0.318857312
ENSG00000114544	SLC41A3	0.885192542	6.403848975	2.50358734	0.019859808	0.278718352
ENSG00000106605	BLVRA	-1.036237514	5.544428433	-2.464579828	0.021638399	0.287600111
ENSG00000230510	PPP5D1	1.364975179	2.079290044	2.343390465	0.028148893	0.321458722
ENSG00000149480	MTA2	-0.627350642	7.806940622	-2.523863036	0.01898996	0.275402745
ENSG00000159251	ACTC1	1.401624037	1.031214401	2.361409325	0.027078447	0.315768956
ENSG00000137269	LRRC1	1.56579338	4.606824607	2.425757566	0.023554297	0.299748964
ENSG00000156970	BUB1B	-1.175808963	4.752163938	-2.418435418	0.023932792	0.300919502
ENSG00000188820	FAM26F	-1.146975955	1.17003181	-2.365948026	0.026814784	0.315768956
ENSG00000106537	TSPAN13	1.164021246	5.492756323	2.465150628	0.02161134	0.287600111

ENSG00000184224	C11orf72	1.516713987	3.427090139	2.359259402	0.027204171	0.315768956
ENSG00000178726	THBD	-1.317190612	1.256733644	-2.358932052	0.027223361	0.315768956
ENSG00000197093	GAL3ST4	-1.08812665	4.816922636	-2.416702692	0.024023179	0.301314195
ENSG00000132359	RAP1GAP2	1.697914152	4.004276231	2.386016196	0.02567707	0.311067838
ENSG00000198727	MT-CYB	0.841716251	8.10893368	2.523253203	0.019015597	0.275402745
ENSG00000130479	MAP1S	-0.890065054	4.787582261	-2.411703621	0.024285723	0.302855048
ENSG00000203392	AC105020.1	1.097282414	2.027893296	2.328999655	0.029031576	0.324917592
ENSG00000143772	ITPKB	1.462993623	5.238295931	2.449876959	0.022346333	0.293665255
ENSG00000138078	PREPL	0.804248596	5.354331144	2.453982727	0.022146513	0.292017588
ENSG00000126759	CFP	-1.271568553	1.797532914	-2.340925544	0.028298312	0.321844263
ENSG00000204264	PSMB8	-1.232627821	6.193078715	-2.473581367	0.021215307	0.286492423
ENSG00000177103	DSCAML1	1.448975927	0.828306205	2.359502916	0.027189904	0.315768956
ENSG00000147036	LANCL3	-1.257039161	0.998362031	-2.36262587	0.027007542	0.315768956
ENSG00000198873	GRK5	1.364839526	5.035632308	2.434965581	0.023086176	0.29838978
ENSG00000205930	C21orf49	1.016524565	0.751515136	2.358133357	0.027270235	0.316024557
ENSG00000078328	RBFOX1	1.244681151	0.459671702	2.36822945	0.026683146	0.315768956
ENSG00000132256	TRIM5	-1.020482302	4.584819674	-2.394964431	0.025184257	0.308962875
ENSG00000169223	LMAN2	-0.81102418	8.112827495	-2.512540738	0.019471223	0.278507707
ENSG00000075826	SEC31B	1.367948961	3.87408637	2.368233477	0.026682914	0.315768956
ENSG00000134532	SOX5	1.206507174	1.131674357	2.340655866	0.028314704	0.321844263
ENSG00000183287	CCBE1	-1.390113284	2.184766676	-2.319170606	0.029648975	0.327558889
ENSG00000198246	SLC29A3	-1.189530437	2.719109115	-2.319454418	0.02963098	0.327558889
ENSG00000086548	CEACAM6	1.205293555	0.631960932	2.358899509	0.027225269	0.315768956
ENSG00000181378	CCDC108	1.173604411	0.823420573	2.348540644	0.027839035	0.318857312
ENSG00000143751	SDE2	0.733830845	5.18767746	2.43577796	0.023045292	0.298352873
ENSG00000118965	WDR35	0.972738695	4.696568851	2.409668534	0.02439336	0.303309572
ENSG00000135248	FAM71F1	0.902423283	0.416320785	2.364765599	0.026883246	0.315768956
ENSG00000163293	NIPAL1	1.338486753	1.386786396	2.325940112	0.029222482	0.326355844
ENSG00000104998	IL27RA	-1.31399998	4.626334573	-2.38120182	0.02594588	0.312206131
ENSG00000203747	FCGR3A	-1.580709267	2.730833589	-2.315592868	0.029876677	0.327882214
ENSG00000147604	RPL7	0.877696729	4.841035175	2.413680406	0.02418159	0.302096597
ENSG00000164307	ERAP1	-1.120817807	5.768951118	-2.44267156	0.022701051	0.295672287
ENSG00000115756	HPCAL1	-1.292275689	5.029454178	-2.408724525	0.024443439	0.303412979
ENSG00000181781	ODF3L2	0.923419585	0.416320785	2.358852028	0.027228054	0.315768956
ENSG00000178814	OPLAH	1.998585431	6.247233326	2.467888134	0.021482	0.287600111
ENSG00000101347	SAMHD1	-0.965015797	6.679839435	-2.473000179	0.02124239	0.28652031
ENSG00000134443	GRP	1.406819163	1.318836147	2.320493127	0.029565207	0.327502506
ENSG00000186409	CCDC30	0.961308234	3.484677023	2.33739202	0.028513779	0.321844263
ENSG00000060982	BCAT1	-1.696280479	3.237730907	-2.318930375	0.029664214	0.327558889
ENSG00000162723	SLAMF9	-1.156521966	0.72519402	-2.35676714	0.027350586	0.316331118
ENSG00000138294	MSMB	1.058349785	0.407137303	2.357361103	0.027315627	0.316316974
ENSG00000164933	SLC25A32	0.954460579	4.506982544	2.39025583	0.025442479	0.30911855
ENSG00000117640	MTFR1L	1.107506358	4.048131816	2.367245388	0.026739853	0.315768956
ENSG00000196517	SLC6A9	1.41276417	5.0104731	2.41573435	0.024073829	0.301470432
ENSG00000166598	HSP90B1	-0.760599541	7.902162139	-2.495308997	0.020225503	0.280903663
ENSG00000125505	MBOAT7	-0.875624672	6.200322251	-2.458078922	0.021948809	0.29014218
ENSG00000196683	TOMM7	1.085260764	3.976772881	2.359082198	0.027214558	0.315768956
ENSG00000135929	CYP27A1	1.197393933	6.04775341	2.458617082	0.021922957	0.290044787
ENSG00000142507	PSMB6	-0.672881888	6.750250186	-2.473561092	0.021216251	0.286492423
ENSG00000044574	HSPA5	-0.798285504	7.359966013	-2.482155419	0.02081946	0.283971801
ENSG00000109911	ELP4	-0.93970358	4.383494826	-2.363846688	0.02693656	0.315768956
ENSG00000117114	LPHN2	-1.351256915	5.062959966	-2.393045262	0.025289212	0.30911855
ENSG00000169499	PLEKHA2	-1.14246399	4.3037188	-2.360635115	0.02712366	0.315768956
ENSG00000122756	CNTFR	2.225620999	3.943717403	2.363445419	0.026959872	0.315768956
ENSG00000116525	TRIM62	-1.22594325	2.446739046	-2.298167135	0.031008795	0.332803697
ENSG00000007255	TRAPPC6A	1.11446634	4.737629526	2.39744441	0.025049227	0.308272492
ENSG00000130881	LRP3	-1.037119099	5.647144163	-2.429759986	0.023349752	0.298598986
ENSG00000144619	CNTN4	-1.562164603	2.248637108	-2.306325076	0.030473988	0.330015644
ENSG00000151726	ACSL1	1.076721523	6.902042061	2.475469474	0.021127541	0.286280011
ENSG00000079101	CLUL1	1.411714526	2.027969459	2.30086152	0.030831218	0.331955912
ENSG00000204538	PSORS1C2	0.887068079	0.402143838	2.348627649	0.027833827	0.318857312
ENSG00000168246	UBTD2	-0.964633117	3.98629625	-2.339460713	0.028387453	0.321844263
ENSG00000171208	NETO2	-1.652241354	4.016915926	-2.337494519	0.028507508	0.321844263

ENSG00000078295	ADCY2	1.970728787	5.066059934	2.411429344	0.024300204	0.302855048
ENSG00000100802	C14orf93	0.977041434	3.16532623	2.311836394	0.030117478	0.328775307
ENSG00000115840	SLC25A12	0.824706155	5.714237833	2.437414674	0.022963126	0.297534608
ENSG00000102934	PLLP	1.02092083	0.751515136	2.32969223	0.028988521	0.324667298
ENSG00000174370	C11orf45	-1.124297902	0.873302744	-2.337216955	0.028524493	0.321844263
ENSG00000079974	RABL2B	0.785328404	5.311849589	2.4199937	0.023851774	0.30085063
ENSG00000188372	ZP3	1.11956405	5.066317612	2.408400573	0.024460646	0.303412979
ENSG00000008277	ADAM22	-1.798728013	2.830665642	-2.301821123	0.030768199	0.331939256
ENSG00000166681	NGFRAP1	-1.97446532	4.015293326	-2.334543556	0.028688567	0.322304955
ENSG00000121281	ADCY7	-0.860361929	5.026748504	-2.390512306	0.025428351	0.30911855
ENSG00000007129	CEACAM21	-1.172520005	1.117916236	-2.321347381	0.029511215	0.327502506
ENSG00000243716	NPIPB5	0.830419611	4.48207983	2.371135526	0.026516326	0.315768956
ENSG00000169247	SH3TC2	1.524357704	1.847717887	2.288205381	0.031673492	0.334358662
ENSG00000174684	B3GNT1	-1.612543306	2.070775498	-2.288654562	0.031643242	0.334358662
ENSG00000198894	CIPC	1.121567592	1.577550424	2.291896659	0.031425688	0.334358662
ENSG00000116266	STXBP3	0.68189852	6.219948691	2.446683624	0.022502901	0.294647798
ENSG00000129538	RNASE1	-1.445820085	7.255588202	-2.465443036	0.02159749	0.287600111
ENSG00000133740	E2F5	1.275497139	4.218204747	2.356584636	0.027361337	0.316331118
ENSG00000161010	C5orf45	0.939816484	4.873899921	2.390067073	0.025452882	0.30911855
ENSG00000110841	PPFIBP1	0.860079147	7.428415084	2.471923438	0.021292652	0.28652031
ENSG00000101265	RASSF2	-1.228345127	4.444430385	-2.350620759	0.027714776	0.318857312
ENSG00000088280	ASAP3	1.100809885	6.581876139	2.454674827	0.022112993	0.291820833
ENSG00000165125	TRPV6	1.927353884	3.160639834	2.296966527	0.031088224	0.333200356
ENSG00000124243	BCAS4	1.445461473	2.945731078	2.303870699	0.030633994	0.331045454
ENSG00000171502	COL24A1	-1.448107157	4.79559616	-2.363481017	0.026957804	0.315768956
ENSG00000167700	MFSD3	1.149795247	4.750696085	2.381203446	0.025945788	0.312206131
ENSG00000163219	ARHGAP25	-1.132723575	3.338040683	-2.297165405	0.031075054	0.333200356
ENSG00000151322	NPAS3	1.402810198	2.365365567	2.286217817	0.031807661	0.334664234
ENSG00000040731	CDH10	-1.599011564	1.307542039	-2.30521752	0.030546097	0.330323092
ENSG00000140104	C14orf79	0.928408027	3.109539022	2.295337233	0.031196311	0.33413059
ENSG00000128165	ADM2	1.359721975	2.838440286	2.28892783	0.031624852	0.334358662
ENSG00000174136	RGMB	-1.366084353	2.017563704	-2.2937049	0.031304944	0.334153657
ENSG00000143776	CDC42BPA	0.904184339	6.559534528	2.450565937	0.022312686	0.29346886
ENSG00000184937	WT1	-1.187889361	0.952928447	-2.320419856	0.029569842	0.327502506
ENSG00000129354	AP1M2	2.815636881	4.476203314	2.360068668	0.027156784	0.315768956
ENSG00000184661	CDCA2	-1.627646077	3.264095261	-2.290401802	0.031525827	0.334358662
ENSG00000185513	L3MBTL1	0.940681472	4.291064476	2.349941377	0.027755304	0.318857312
ENSG00000137161	CNPY3	-0.829583333	6.278426278	-2.435283637	0.023070162	0.29838978
ENSG00000167618	LAIR2	-1.39791421	1.582017229	-2.290538103	0.031516684	0.334358662
ENSG00000163064	EN1	1.426371436	3.953807664	2.329928635	0.028973838	0.324667298
ENSG00000138061	CYP1B1	-1.695085847	2.527529095	-2.282262234	0.032076225	0.336423295
ENSG00000157326	DHRS4	1.195813408	3.097161135	2.298451203	0.030990029	0.332803697
ENSG00000215012	C22orf29	1.288735881	2.388757643	2.270802302	0.032866031	0.339723905
ENSG00000215788	TNFRSF25	0.876744704	4.951883123	2.383524303	0.025815882	0.311597697
ENSG00000143119	CD53	-1.466926328	4.339010155	-2.33522651	0.02864657	0.322217445
ENSG00000180353	HCLS1	-1.353717005	6.366530035	-2.43187123	0.023242519	0.298554465
ENSG00000188375	H3F3C	1.054724455	0.56504896	2.317418155	0.029760308	0.327697544
ENSG00000223547	ZNF844	0.956958573	4.753435793	2.368748651	0.026653271	0.315768956
ENSG00000269866	FKSG63	1.201054759	3.681316611	2.313284808	0.03002442	0.328646526
ENSG00000138435	CHRNA1	-1.331330047	0.820682774	-2.31503506	0.029912322	0.327990389
ENSG00000214160	ALG3	-0.718485642	6.427458695	-2.430134451	0.023330699	0.298598986
ENSG00000204386	NEU1	-0.74891835	6.131218519	-2.420412285	0.023830054	0.30085063
ENSG00000104299	INTS9	-0.877531926	5.006463328	-2.369066149	0.026635017	0.315768956
ENSG00000161509	GRIN2C	1.724451852	1.551229307	2.274280507	0.03262446	0.338789152
ENSG00000105675	ATP4A	0.889321519	0.378582783	2.320303429	0.029577209	0.327502506
ENSG00000185818	NAT8L	1.610586024	1.567639657	2.273339167	0.032689678	0.338793755
ENSG00000173372	C1QA	-1.772963389	4.150700631	-2.312384241	0.030082249	0.328766596
ENSG00000171357	LURAP1	-1.006880927	1.304382412	-2.287941304	0.031691289	0.334358662
ENSG00000233276	GPX1	-1.304070723	3.587147282	-2.287463871	0.031723487	0.334358662
ENSG00000117625	RCOR3	0.765058386	5.262296057	2.388372129	0.025546465	0.310003169
ENSG00000163297	ANTXR2	-1.119906408	5.130570188	-2.371431245	0.026499405	0.315768956
ENSG00000152763	WDR78	1.331017047	1.804064168	2.261362903	0.033529868	0.341241014
ENSG00000175183	CSRP2	-1.248259366	4.280052772	-2.322198376	0.029457518	0.327502506

ENSG00000131037	EPS8L1	1.639869507	4.293010481	2.334432013	0.028695432	0.322304955
ENSG00000184599	FAM19A3	1.725850737	1.906815665	2.262576091	0.03344387	0.341241014
ENSG00000244165	P2RY11	1.289982514	1.507954553	2.267866646	0.033071188	0.340344316
ENSG00000146830	GIGYF1	0.901158794	7.459173331	2.451157517	0.022283832	0.29346886
ENSG00000178773	CPNE7	2.051098296	3.432197697	2.291436385	0.03145649	0.334358662
ENSG00000182685	BRICD5	1.198915895	3.254922885	2.277239663	0.032420217	0.33823271
ENSG00000117758	STX12	0.800585677	5.414551084	2.392113715	0.025340302	0.30911855
ENSG00000221819	C16orf3	1.109052081	0.780069656	2.29483492	0.031229703	0.334153657
ENSG00000141510	TP53	-0.918073489	6.567675379	-2.423208195	0.023685446	0.300279204
ENSG00000154016	GRAP	-1.155458936	1.706564899	-2.263946061	0.033347	0.341241014
ENSG00000104870	FCGRT	-0.953817095	7.208579966	-2.439787377	0.022844493	0.296246044
ENSG00000198019	FCGR1B	-1.134015431	0.686836567	-2.307075011	0.030425251	0.330015644
ENSG00000164620	RELL2	1.297595333	1.340657198	2.266520997	0.033165619	0.340800081
ENSG00000174744	BRMS1	-0.624046958	6.512512958	-2.420934649	0.023802975	0.30085063
ENSG00000116151	MORN1	1.310623365	2.851497598	2.267760955	0.033078596	0.340344316
ENSG00000108984	MAP2K6	-1.028120517	3.467752253	-2.273870195	0.032652873	0.338793755
ENSG00000110442	COMMD9	-0.624214856	5.694225682	-2.392095365	0.025341309	0.30911855
ENSG00000110148	CCKBR	1.419132895	0.970626475	2.279230661	0.032283454	0.337423144
ENSG00000102172	SMS	-1.06646985	2.897777532	-2.254782852	0.033999825	0.341762496
ENSG00000134398	ERN2	1.284532776	0.739370967	2.284269002	0.031939718	0.335228167
ENSG00000101049	SGK2	1.456901309	2.350128229	2.250339011	0.034320598	0.342732818
ENSG00000182704	TSKU	-1.342267756	2.060126215	-2.251064592	0.034268035	0.342702192
ENSG00000065923	SLC9A7	-1.298771817	3.287763661	-2.262154051	0.033473764	0.341241014
ENSG00000123131	PRDX4	-0.960095253	8.36618795	-2.450689436	0.022306659	0.29346886
ENSG00000088726	TMEM40	-1.506107226	1.956406518	-2.257638751	0.03379512	0.341241014
ENSG00000069956	MAPK6	-1.406742195	2.152680428	-2.251668594	0.034224336	0.342483453
ENSG00000112624	GLTSCR1L	1.339057858	3.457050129	2.274573277	0.032604201	0.338789152
ENSG00000119139	TJP2	0.924643895	6.040188955	2.403036201	0.024747214	0.305274008
ENSG00000162669	HFM1	1.16986503	1.300084562	2.256052511	0.03390868	0.341241014
ENSG00000164430	MB21D1	-1.242257447	4.17251876	-2.294209599	0.031271319	0.334153657
ENSG00000106144	CASP2	0.745792567	5.61583036	2.38540304	0.025711162	0.311067838
ENSG00000159167	STC1	-1.856576513	2.714768512	-2.252127216	0.034191189	0.342370096
ENSG00000019995	ZRANB1	0.817936813	5.207811923	2.36577637	0.026824713	0.315768956
ENSG00000105926	MPP6	1.002575901	4.418607017	2.323147304	0.029397748	0.327437672
ENSG00000130592	LSP1	-1.419466616	6.845766726	-2.413951208	0.024167357	0.302096597
ENSG00000179241	LDLRAD3	0.929225603	4.164985461	2.306408636	0.030468554	0.330015644
ENSG00000167244	IGF2	2.554581681	4.648927807	2.336435401	0.02857237	0.321844263
ENSG00000119866	BCL11A	1.191819401	3.783779602	2.287917114	0.031692919	0.334358662
ENSG00000116221	MRPL37	-0.869372338	6.57172992	-2.407541417	0.024506336	0.303530991
ENSG00000171067	C11orf24	-0.838484833	5.139560087	-2.351401021	0.027668298	0.318857312
ENSG00000090382	LYZ	-1.885916577	5.406120283	-2.348943927	0.027814904	0.318857312
ENSG00000126878	AIF1L	2.085812743	4.18426961	2.306650127	0.030452855	0.330015644
ENSG00000169403	PTAFR	-1.480639654	3.736853939	-2.263249412	0.033396227	0.341241014
ENSG00000267545	AC005779.2	0.97332055	0.378582783	2.290102922	0.031545883	0.334358662
ENSG00000129518	EAPP	0.730452592	5.360159702	2.36789555	0.026702375	0.315768956
ENSG00000135702	CHST5	1.236283183	1.31160928	2.249482067	0.034382771	0.342757347
ENSG00000157456	CCNB2	-1.198748959	5.201381111	-2.342638984	0.02819437	0.321510069
ENSG00000100365	NCF4	-1.274529888	4.059340805	-2.281345545	0.032138758	0.336423295
ENSG00000198948	MFAP3L	1.56695896	2.002297577	2.234618052	0.035477635	0.345978605
ENSG00000164309	CMYA5	1.882394483	1.954114744	2.233775321	0.03554065	0.346104242
ENSG00000101251	SEL1L2	0.80914639	0.363786385	2.289775509	0.031567868	0.334358662
ENSG00000198899	MT-ATP6	0.834242195	7.06629964	2.420120525	0.023845191	0.30085063
ENSG00000171119	NRTN	1.389278966	1.517757486	2.240438109	0.035045214	0.345008771
ENSG00000147684	NDUFB9	0.82239752	6.151611476	2.396921584	0.025077638	0.30838027
ENSG00000111145	ELK3	-1.172090653	3.085528584	-2.238928992	0.035156876	0.345567417
ENSG00000180264	GPR144	1.038105355	0.378582783	2.28738187	0.03172902	0.334358662
ENSG00000215375	MYL5	1.053043698	3.296912847	2.256239296	0.033895289	0.341241014
ENSG00000198929	NOS1AP	1.334437799	2.223998083	2.225059389	0.036198367	0.346212821
ENSG00000165819	METTL3	0.953949378	5.810222238	2.384153074	0.025780791	0.311413697
ENSG00000111328	CDK2AP1	0.640318064	7.067662633	2.417971655	0.023956953	0.300960257
ENSG00000164674	SYTL3	-1.163470403	3.00843186	-2.235773214	0.035391424	0.345567417
ENSG00000033627	ATP6V0A1	0.64801268	5.957427329	2.388032859	0.025565235	0.310003169
ENSG00000168876	ANKRD49	1.27843844	1.39004597	2.240395387	0.03504837	0.345008771

ENSG00000160439	RDH13	0.997036024	4.71546191	2.329701656	0.028987936	0.324667298
ENSG00000204482	LST1	-1.604700179	3.090353078	-2.236421876	0.035343098	0.345567417
ENSG00000160877	NACC1	-0.991153417	5.396453055	-2.349459646	0.027784074	0.318857312
ENSG00000171940	ZNF217	1.021568178	4.277922292	2.301638729	0.030780168	0.331939256
ENSG00000144959	NCEH1	-1.388386681	1.720634013	-2.25254776	0.034160819	0.342370096
ENSG00000047634	SCML1	-1.451279674	2.765731863	-2.228429627	0.035942743	0.346212821
ENSG00000188747	NOXA1	1.453176948	3.978686824	2.284484569	0.031925086	0.335228167
ENSG00000172785	CBWD1	0.76884294	5.561135018	2.367521062	0.026723956	0.315768956
ENSG00000128294	TPST2	-1.179335276	4.846484705	-2.315745181	0.02986695	0.327882214
ENSG00000214078	CPNE1	-0.842423683	7.878990188	-2.423087952	0.023691648	0.300279204
ENSG00000181873	IBA57	1.047423578	3.383192676	2.25324075	0.034110829	0.342370096
ENSG00000205129	C4orf47	1.351051203	1.770611779	2.225154354	0.036191142	0.346212821
ENSG00000167889	MGAT5B	-1.759972282	2.854700366	-2.222498861	0.03639368	0.347279205
ENSG00000173421	CCDC36	1.133994236	1.860178705	2.219997959	0.036585368	0.347856598
ENSG00000183426	NPIPA1	0.900199097	7.727683791	2.419592628	0.023872602	0.300871486
ENSG00000125648	SLC25A23	1.250651986	4.821021644	2.327621056	0.029117454	0.325646455
ENSG00000137825	ITPKA	-1.094392554	1.175025274	-2.252546634	0.034160901	0.342370096
ENSG00000125355	TMEM255A	-1.745914583	1.646352217	-2.226500507	0.036088861	0.346212821
ENSG00000231256	C17orf105	1.073198909	0.624810227	2.258362809	0.0337434	0.341241014
ENSG00000133119	RFC3	-1.013414313	3.753153637	-2.249816873	0.034358468	0.342732818
ENSG00000187688	TRPV2	-1.086698017	4.188210071	-2.274483372	0.032610421	0.338789152
ENSG00000020633	RUNX3	-1.51144028	4.400295321	-2.281336902	0.032139349	0.336423295
ENSG00000188641	DPYD	-1.450120186	3.977257417	-2.257940553	0.033773553	0.341241014
ENSG00000173898	SPTBN2	1.554095789	5.878875397	2.371001297	0.02652401	0.315768956
ENSG00000161956	SENP3	-0.977501719	3.584658792	-2.240708442	0.035025245	0.345008771
ENSG00000159184	HOXB13	1.262659424	1.266991706	2.22995298	0.03582774	0.346212821
ENSG00000111252	SH2B3	-1.018761258	4.890879887	-2.31312	0.030034996	0.328646526
ENSG00000164626	KCNK5	1.577908467	3.850629971	2.26124681	0.033538108	0.341241014
ENSG00000166343	MSS51	0.925001377	3.237781352	2.235938601	0.035379097	0.345567417
ENSG00000101447	FAM83D	-1.252838632	3.444951664	-2.231557127	0.035706999	0.346146062
ENSG00000161640	SIGLEC11	-1.112023543	1.781742015	-2.218088827	0.036732315	0.348435972
ENSG00000171792	RHNO1	-1.10186231	4.266925284	-2.268356048	0.033036906	0.340344316
ENSG00000125675	GRIA3	1.233356219	0.940214716	2.237842272	0.035237485	0.345567417
ENSG00000171345	KRT19	2.787356685	4.892507454	2.319998803	0.029596492	0.327502506
ENSG00000175497	DPP10	1.154117221	0.46473974	2.259059796	0.033693682	0.341241014
ENSG00000128228	SDF2L1	-0.969190507	4.652723181	-2.293985748	0.031286229	0.334153657
ENSG00000140873	ADAMTS18	-1.331016364	1.683638812	-2.219790429	0.036601316	0.347856598
ENSG00000111247	RAD51AP1	-1.690975194	3.20686316	-2.22275124	0.036374386	0.347279205
ENSG00000125538	IL1B	-1.462706524	2.057007056	-2.214830705	0.036984332	0.349190098
ENSG00000110318	KIAA1377	1.376292661	3.206893649	2.232616722	0.035627449	0.346146062
ENSG00000171100	MTM1	1.17057499	3.628465183	2.248256252	0.034471886	0.342991983
ENSG00000136378	ADAMTS7	-1.076752844	5.50379113	-2.33964095	0.028376471	0.321844263
ENSG00000121690	DEPDC7	-1.373248198	2.558813469	-2.205400379	0.03772264	0.351696939
ENSG00000105889	STEAP1B	-1.690859163	2.339320379	-2.210694861	0.037306501	0.350314968
ENSG00000254901	MEF2BNB	0.899154655	3.907717528	2.259815204	0.033639871	0.341241014
ENSG00000153885	KCTD15	0.798485713	5.2329706	2.337211663	0.028524817	0.321844263
ENSG00000130830	MPP1	-1.097019246	5.465094772	-2.33653553	0.028566232	0.321844263
ENSG00000156831	NSMCE2	0.965988915	5.235543028	2.335644184	0.028620914	0.322159801
ENSG00000130787	HIP1R	1.042358408	5.913432445	2.3621326	0.027036271	0.315768956
ENSG00000147162	OGT	0.886541213	8.445497886	2.414900191	0.02411754	0.301776968
ENSG00000078900	TP73	-1.830828616	1.797408479	-2.21244825	0.037169608	0.349878336
ENSG00000105246	EBI3	-1.342164734	0.964270759	-2.244271997	0.034762987	0.343925622
ENSG00000183891	TTC32	1.139258753	1.577550424	2.206895602	0.037604693	0.35153641
ENSG00000080503	SMARCA2	0.746827735	7.826651606	2.405561453	0.02461193	0.304136669
ENSG00000136235	GPNMB	-1.312760445	7.318836204	-2.39308524	0.025287022	0.30911855
ENSG00000123329	ARHGAP9	-1.413377634	4.411310508	-2.267486696	0.033097826	0.340344316
ENSG00000136531	SCN2A	-1.542131083	1.255830823	-2.233063979	0.035593919	0.346146062
ENSG00000048740	CELF2	1.458577137	3.865309103	2.257642942	0.033794821	0.341241014
ENSG00000110852	CLEC2B	-1.164420139	4.032952523	-2.246357237	0.034610356	0.343345171
ENSG00000085999	RAD54L	-1.366913426	4.177225058	-2.250629829	0.034299521	0.342732818
ENSG00000120725	SIL1	-0.916197127	5.513942313	-2.333195233	0.028771646	0.322929827
ENSG00000146374	RSPO3	-1.205293807	1.50787839	-2.217984609	0.036740352	0.348435972
ENSG00000234719	RP11-166B2.1	1.225040552	2.333294226	2.198213682	0.038294239	0.353663863

ENSG00000169918	OTUD7A	-1.251955663	1.012120152	-2.238553363	0.03518472	0.345567417
ENSG00000115935	WIPF1	-0.935914084	5.001296825	-2.306580134	0.030457404	0.330015644
ENSG00000183111	ARHGEF37	1.798502262	3.430320024	2.224936572	0.036207714	0.346212821
ENSG00000156697	UTP14A	-1.015470403	3.058590819	-2.2038581	0.03784465	0.352114194
ENSG00000164924	YWHAZ	0.8364659	6.543130003	2.376769345	0.026195656	0.314728971
ENSG00000162551	ALPL	-2.206540496	5.743700532	-2.34028023	0.02833755	0.321844263
ENSG00000131080	EDA2R	1.289651617	1.654720457	2.205105574	0.037745935	0.351705144
ENSG00000005249	PRKAR2B	1.440623792	4.060093286	2.258089697	0.0337629	0.341241014
ENSG00000129682	FGF13	1.592605991	2.641666919	2.202086105	0.037985275	0.352114194
ENSG00000163106	HPGDS	-1.463009303	1.724106318	-2.199382095	0.038200777	0.353216498
ENSG00000136830	FAM129B	0.779788856	8.650641752	2.410036279	0.024373877	0.303309572
ENSG00000149923	PPP4C	-0.800184084	7.186216194	-2.385022636	0.025732334	0.311067838
ENSG00000143457	GOLPH3L	0.988149565	4.779646892	2.300420297	0.030860233	0.331955912
ENSG00000233232	NPIPB7	1.190790632	3.284264422	2.21698061	0.03681786	0.348647583
ENSG00000121552	CSTA	-1.437708679	1.398066908	-2.221966088	0.036434439	0.347279205
ENSG00000189159	HN1	-1.576225874	5.955302066	-2.337614492	0.028500169	0.321844263
ENSG00000085552	IGSF9	1.466118196	5.331437826	2.326598741	0.029181289	0.326127919
ENSG00000173214	KIAA1919	-1.263045899	3.192034644	-2.198253994	0.038291011	0.353663863
ENSG00000146409	SLC18B1	-1.012300824	4.498380553	-2.260449476	0.033594751	0.341241014
ENSG00000152767	FARP1	0.838485113	8.316828204	2.39917636	0.024955323	0.30759935
ENSG00000166803	KIAA0101	-1.413069738	3.703977874	-2.215735773	0.036914167	0.349138156
ENSG00000159263	SIM2	1.804456633	3.635002202	2.232112268	0.035665301	0.346146062
ENSG00000144741	SLC25A26	0.790107677	4.724739233	2.289706441	0.031572508	0.334358662
ENSG00000142632	ARHGEF19	1.118541208	5.700684327	2.338053187	0.028473348	0.321844263
ENSG00000102384	CENPI	-1.189011801	3.657011076	-2.211476903	0.037245388	0.350075752
ENSG00000234284	ZNF879	-1.048172706	1.103119838	-2.220582863	0.036540454	0.347699376
ENSG00000163909	HEYL	-1.531365685	3.03221243	-2.188550672	0.039075162	0.358011153
ENSG00000198300	PEG3	1.566296105	1.486873417	2.19119393	0.038860125	0.356998959
ENSG00000146386	ABRACL	-1.354053376	4.210991644	-2.235152176	0.035437749	0.345804555
ENSG00000121064	SCPEP1	-1.060108165	7.6474783	-2.380105384	0.02600746	0.312707324
ENSG00000089693	MLF2	-0.680105848	8.297785071	-2.392278387	0.025331263	0.30911855
ENSG00000197608	ZNF841	0.803178129	4.172767536	2.252219471	0.034184524	0.342370096
ENSG00000172426	RSPH9	0.85261567	0.521698043	2.230564736	0.035781651	0.346212821
ENSG00000105479	CCDC114	1.433977877	1.418116494	2.191504164	0.038834957	0.356976748
ENSG00000163319	MRPS18C	-1.353824426	2.328376924	-2.189795788	0.038973734	0.357833157
ENSG00000140990	NDUFB10	-0.824508046	6.191188476	-2.344286012	0.028094786	0.321308523
ENSG00000151689	INPP1	-1.135604087	3.938761619	-2.226484137	0.036090103	0.346212821
ENSG00000186487	MYT1L	0.857147173	0.521698043	2.229901259	0.035831639	0.346212821
ENSG00000163141	BNIPL	1.440610555	4.097627888	2.246663421	0.034587995	0.343345171
ENSG00000169895	SYAP1	-0.899587167	7.391899433	-2.373017694	0.026408796	0.315768956
ENSG00000146263	MMS22L	-0.949880623	4.593667316	-2.256217091	0.033896881	0.341241014
ENSG00000095585	BLNK	-1.102519615	3.571830035	-2.202595786	0.037944779	0.352114194
ENSG00000104205	SGK3	0.928838267	4.031178656	2.239195897	0.035137104	0.345567417
ENSG00000171316	CHD7	1.233231161	7.055723614	2.369800109	0.026592865	0.315768956
ENSG00000143768	LEFTY2	1.708536534	1.319118441	2.188100165	0.03911192	0.358054335
ENSG00000178409	BEND3	1.062188286	1.866709959	2.172625598	0.040393659	0.360866344
ENSG00000167528	ZNF641	1.106384024	2.870917228	2.182040445	0.03960939	0.359254874
ENSG00000173575	CHD2	0.586868215	7.43240752	2.375174128	0.026286089	0.315232739
ENSG00000121022	COPS5	0.823629225	6.265697	2.348527628	0.027839814	0.318857312
ENSG00000101977	MCF2	-1.205182242	0.753748539	-2.227281942	0.036029608	0.346212821
ENSG00000179387	ELMOD2	-0.907672884	4.083060554	-2.225503269	0.036164606	0.346212821
ENSG00000179833	SERTAD2	1.316952134	2.591729764	2.176892769	0.040036475	0.36038212
ENSG00000146963	C7orf55-LUC7L2	1.12480598	2.32703976	2.177888161	0.039953567	0.36038212
ENSG00000225614	ZNF469	1.564023698	2.377356274	2.184153056	0.039435309	0.358641419
ENSG00000241935	HOGA1	1.039932473	1.146470755	2.195831476	0.038485434	0.354178856
ENSG00000182272	B4GALNT4	1.674980172	6.170356072	2.343644208	0.028133553	0.321458722
ENSG00000198223	CSF2RA	-1.164697501	4.741727253	-2.25965445	0.033651316	0.341241014
ENSG00000133477	FAM83F	1.592411109	3.569079603	2.205965677	0.037678009	0.35153641
ENSG00000101236	RNF24	0.98961046	5.416892872	2.310734638	0.03018844	0.329177773
ENSG00000147576	ADHFE1	1.288661577	2.101412684	2.167608797	0.040817273	0.361005094
ENSG00000160200	CBS	1.644752391	6.243564711	2.342784673	0.028185548	0.321510069
ENSG00000159445	THEM4	0.785208186	4.019309584	2.231261651	0.035729211	0.346146062
ENSG00000107863	ARHGAP21	1.090451253	4.239099454	2.245969746	0.034638672	0.343345171

ENSG00000104938	CLEC4M	-1.017423051	1.103119838	-2.202530008	0.037950003	0.352114194
ENSG00000118985	ELL2	-1.342576376	2.819101837	-2.168609441	0.04073246	0.360872756
ENSG00000187145	MRPS21	0.789048819	4.560340653	2.259000022	0.033697943	0.341241014
ENSG00000189319	FAM53B	1.168406371	1.562754025	2.168358511	0.040753714	0.360872756
ENSG00000147145	LPAR4	-1.349654219	1.125833062	-2.196830594	0.03840514	0.354062901
ENSG00000008441	NFIX	1.262871651	7.578809855	2.365817847	0.026822314	0.315768956
ENSG00000197860	SGTB	-0.853373344	3.542183866	-2.187176875	0.03918735	0.3580839
ENSG00000111729	CLEC4A	-1.509017959	1.953535143	-2.172338471	0.040417796	0.360866344
ENSG00000213020	ZNF611	0.686504952	5.885326796	2.323253389	0.029391073	0.327437672
ENSG00000071246	VASH1	-1.213158629	3.976172941	-2.207538162	0.037554109	0.35153641
ENSG00000110911	SLC11A2	0.932542117	6.299973529	2.336969425	0.028539649	0.321844263
ENSG00000168785	TSPAN5	1.38494412	4.380739723	2.248426216	0.034459517	0.342991983
ENSG00000198353	HOXC4	0.967629302	0.550252562	2.211306257	0.037258716	0.350075752
ENSG00000141750	STAC2	2.76122666	5.258392535	2.293009741	0.031351312	0.334193914
ENSG00000116819	TFAP2E	1.284462205	1.199005155	2.175978023	0.040112802	0.360460451
ENSG00000212907	MT-ND4L	1.111606422	5.94166888	2.322131759	0.029461719	0.327502506
ENSG00000116478	HDAC1	-0.694170195	6.917691482	-2.344601994	0.028075718	0.321308523
ENSG00000134825	TMEM258	-0.839330335	6.052003291	-2.316756521	0.029802442	0.327882214
ENSG00000148143	ZNF462	0.879469122	5.86105907	2.317646234	0.029745797	0.327697544
ENSG00000215252	GOLGA8B	1.525140887	2.136741746	2.14565182	0.042718888	0.363701616
ENSG00000142188	TMEM50B	0.844493928	5.862188875	2.314363117	0.029955313	0.328232409
ENSG00000179954	SSC5D	-1.261475741	4.090371715	-2.204269435	0.037812075	0.352112323
ENSG00000213366	GSTM2	1.183849686	4.959276091	2.271299347	0.03283141	0.339589751
ENSG00000100600	LGMN	-0.807319012	6.952486527	-2.34088785	0.028300603	0.321844263
ENSG00000116132	PRRX1	-1.065694815	5.127445604	-2.272037814	0.032780035	0.339505958
ENSG00000186235	AC016757.3	0.853528813	0.521698043	2.200852878	0.038083423	0.35260695
ENSG00000168255	POLR2J3	0.994206578	5.555861508	2.30032755	0.030866336	0.331955912
ENSG00000164087	POC1A	-0.718841437	4.428563	-2.225661275	0.036152595	0.346212821
ENSG00000185046	ANKS1B	-1.177846682	2.516795936	-2.144092531	0.042856924	0.363851129
ENSG00000164972	C9orf24	1.579940487	1.068874759	2.171018271	0.040528946	0.360866344
ENSG00000174945	AMZ1	-1.182573118	1.572633122	-2.168617851	0.040731748	0.360872756
ENSG00000066468	FGFR2	1.482176603	6.084187125	2.3182133	0.029709745	0.327697544
ENSG00000084070	SMAP2	-0.924035572	5.451522355	-2.286108729	0.03181504	0.334664234
ENSG00000100526	CDKN3	-1.454834214	4.693602904	-2.226233891	0.036109098	0.346212821
ENSG00000102879	CORO1A	-1.426133726	6.004698849	-2.305581072	0.03052241	0.330294578
ENSG00000115252	PDE1A	-1.431254491	1.73238973	-2.161591048	0.041330697	0.362387043
ENSG00000131126	TEX101	0.767765415	0.335231866	2.208271588	0.037496448	0.35153641
ENSG00000175344	CHRNA7	-1.201940507	1.406380157	-2.169454057	0.040660997	0.360866344
ENSG00000112335	SNX3	-0.736519972	7.135090648	-2.339960629	0.028357002	0.321844263
ENSG00000184271	POU6F1	1.090392384	4.089119173	2.216262677	0.036873375	0.348962686
ENSG00000198856	OSTC	-1.093195263	3.368570036	-2.161133069	0.041370009	0.362387043
ENSG00000112541	PDE10A	-1.560182886	2.179324223	-2.153103311	0.042064768	0.362636645
ENSG00000077721	UBE2A	-0.705952561	5.917025745	-2.301924286	0.030761431	0.331939256
ENSG00000160219	GAB3	-1.316798039	1.767635575	-2.149320483	0.042395702	0.362636645
ENSG00000169914	OTUD3	1.13564592	3.009290081	2.156078274	0.041806147	0.362636645
ENSG00000136250	AOAH	-1.73577544	4.386523852	-2.210010955	0.03736002	0.3506077
ENSG00000155313	USP25	0.687537064	6.151338133	2.315516407	0.029881561	0.327882214
ENSG00000144791	LIMD1	0.81550094	5.28692983	2.278998422	0.032299379	0.337423144
ENSG00000107611	CUBN	1.08226596	3.654288936	2.188952104	0.039042435	0.358011153
ENSG00000138152	BTBD16	1.094952856	0.775076191	2.178870442	0.039871904	0.360254047
ENSG00000089597	GANAB	-0.589787994	9.793318381	-2.372750627	0.026424029	0.315768956
ENSG00000162069	CCDC64B	2.168229467	3.663348998	2.178883284	0.039870837	0.360254047
ENSG00000070159	PTPN3	1.010578843	5.442443524	2.284870082	0.031898934	0.335228167
ENSG00000166866	MYO1A	1.094762373	0.588610015	2.187618018	0.039151293	0.3580839
ENSG00000232774	RP11-47I22.3	-1.283073298	1.238181571	-2.168457275	0.040745347	0.360872756
ENSG00000011454	RABGAP1	0.834186208	6.554481678	2.323066682	0.029402822	0.327437672
ENSG00000138468	SENP7	1.132163131	4.477741938	2.23115185	0.035737469	0.346146062
ENSG00000100714	MTHFD1	-0.802051197	5.889286716	-2.29329228	0.031332459	0.334193914
ENSG00000215784	FAM72D	1.243504205	1.860070872	2.135678029	0.04360877	0.364118884
ENSG00000185112	FAM43A	-0.978463505	0.98133223	-2.181313079	0.039669486	0.359384469
ENSG00000151835	SACS	-1.009361757	1.327943467	-2.162215672	0.041277135	0.362387043
ENSG00000121101	TEX14	1.37301299	2.030305039	2.137809573	0.043417199	0.364118884
ENSG00000101098	RIMS4	-1.025197239	0.658282048	-2.194437116	0.038597746	0.355004241

ENSG00000198252	STYX	-0.781504468	4.381374083	-2.208005277	0.037517376	0.35153641
ENSG00000213132	AC022498.1	1.404815459	1.561231753	2.140885565	0.043142082	0.364118884
ENSG00000156103	MMP16	1.356398469	1.539085138	2.139704675	0.043247515	0.364118884
ENSG00000166396	SERPINB7	-1.086789924	0.708164219	-2.186184233	0.039268592	0.3580839
ENSG00000126787	DLGAP5	-1.303721451	5.084299127	-2.238370797	0.03519826	0.345567417
ENSG00000177885	GRB2	-0.681677444	6.807285471	-2.321713093	0.029488128	0.327502506
ENSG00000172543	CTSW	-1.560020391	2.065803055	-2.141777626	0.04306259	0.364118884
ENSG00000167769	ACER1	1.030864912	0.730187485	2.173779356	0.040296801	0.36083805
ENSG00000170373	CST1	1.247490717	0.647576293	2.176668003	0.040055217	0.36038212
ENSG00000044524	EPHA3	-1.74168906	2.581059459	-2.125773818	0.044508924	0.366597905
ENSG00000136634	IL10	-0.954540924	1.038441269	-2.171294801	0.040505641	0.360866344
ENSG00000135164	DMTF1	0.909805629	6.518164431	2.316199979	0.029837925	0.327882214
ENSG00000179066	AC020907.1	1.302725634	0.664813302	2.169901348	0.040623197	0.360866344
ENSG00000166896	XRCC6BP1	-1.005716771	3.039402189	-2.135618472	0.043614134	0.364118884
ENSG00000133019	CHRM3	1.406427211	1.815712052	2.13213572	0.043928817	0.364316632
ENSG00000196781	TLE1	0.910812649	6.031042262	2.300927845	0.030826858	0.331955912
ENSG00000187713	TMEM203	-1.071311843	1.759099298	-2.137674008	0.04342936	0.364118884
ENSG00000214189	ZNF788	1.161103221	1.217680578	2.146986045	0.042601095	0.363290101
ENSG00000132970	WASF3	1.489836916	3.056368826	2.144219954	0.042845628	0.363851129
ENSG00000164116	GUCY1A3	-1.825366482	4.260007019	-2.186515474	0.039241465	0.3580839
ENSG00000256060	TRAPPC2P1	1.02021986	1.261031495	2.143882119	0.042875581	0.363851129
ENSG00000205176	REXO1L1P	-1.011519802	0.664813302	-2.183415076	0.039496041	0.358641419
ENSG00000198719	DLL1	-1.312368794	3.363798489	-2.143501544	0.042909345	0.36394083
ENSG00000203546	RP11-176H8.1	1.162022519	1.440966418	2.135578073	0.043617773	0.364118884
ENSG00000188921	PTPLAD2	-0.932804038	4.030009202	-2.176934766	0.040032974	0.36038212
ENSG00000168894	RNF181	-0.824705596	4.507783912	-2.205902195	0.037683018	0.35153641
ENSG00000161326	DUSP14	1.125673692	0.916653661	2.153823301	0.042002045	0.362636645
ENSG00000147316	MCPH1	-1.089098851	3.445974022	-2.14498434	0.042777928	0.363809464
ENSG00000146376	ARHGAP18	-0.738059418	5.303526898	-2.25668004	0.033863713	0.341241014
ENSG00000101443	WFDC2	-2.550590489	3.640149448	-2.158275694	0.041616042	0.362636645
ENSG00000157796	WDR19	0.875400049	5.183389489	2.259822916	0.033639322	0.341241014
ENSG00000178860	MSC	-1.218836023	0.960004579	-2.167273989	0.040845686	0.361005094
ENSG00000141040	ZNF287	-1.194270903	2.400482986	-2.118303926	0.045198849	0.369575375
ENSG00000166337	TAF10	-0.825128539	6.693056068	-2.310340464	0.030213865	0.329226224
ENSG00000184678	HIST2H2BE	1.388746638	1.47175434	2.130030432	0.044120029	0.365130473
ENSG00000267060	PTGES3L	1.214403262	1.31160928	2.136211664	0.043560739	0.364118884
ENSG00000104889	RNASEH2A	-1.19327967	5.991691219	-2.276929907	0.032441542	0.33823271
ENSG00000130427	EPO	0.921596893	0.521698043	2.173039902	0.040358854	0.36083805
ENSG00000187535	IFT140	0.732503815	5.834659424	2.286005818	0.031822002	0.334664234
ENSG00000011465	DCN	-1.594402842	8.347622459	-2.339243359	0.028400701	0.321844263
ENSG00000163807	KIAA1143	0.88866143	3.41873838	2.149842123	0.042349928	0.362636645
ENSG00000144843	ADPRH	-1.203187572	2.025191178	-2.1367787	0.043509753	0.364118884
ENSG00000186470	BTN3A2	-1.126331801	5.559036411	-2.256441704	0.033880785	0.341241014
ENSG00000166321	NUDT13	1.041026332	0.98133223	2.151209174	0.04223018	0.362636645
ENSG00000255112	CHMP1B	1.260472175	2.892194155	2.128164861	0.044290094	0.366151671
ENSG00000065328	MCM10	-1.050417925	3.787713908	-2.154845397	0.041913149	0.362636645
ENSG00000137968	SLC44A5	-1.522557565	1.996833218	-2.125453539	0.04453831	0.366597905
ENSG00000204681	GABBR1	1.49806481	6.739950752	2.311632832	0.030130577	0.328775307
ENSG00000135336	ORC3	-0.931529536	4.929476521	-2.225684592	0.036150823	0.346212821
ENSG00000135334	AKIRIN2	-0.735974893	5.000224533	-2.23263892	0.035625784	0.346146062
ENSG00000137767	SQRDL	-1.415240725	3.476077541	-2.137754211	0.043422165	0.364118884
ENSG00000151327	FAM177A1	0.755114508	6.590407487	2.307242189	0.030414396	0.330015644
ENSG00000140848	CPNE2	-0.907629951	6.318400601	-2.292597793	0.03137882	0.334260053
ENSG00000101974	ATP11C	-1.166030061	4.076618509	-2.17039924	0.040581158	0.360866344
ENSG00000186185	KIF18B	-1.339885659	5.193492358	-2.228664653	0.035924979	0.346212821
ENSG00000144747	TMF1	0.924296073	5.085203857	2.246130427	0.034626928	0.343345171
ENSG00000123892	RAB38	-1.263008548	1.233443722	-2.148815133	0.042440089	0.362636645
ENSG00000188257	PLA2G2A	3.04363718	3.673112175	2.165744373	0.040975723	0.361005094
ENSG00000135094	SDS	-1.328830649	3.180988797	-2.123764468	0.044693572	0.367358218
ENSG00000130779	CLIP1	0.733547276	6.803528435	2.308376457	0.030340842	0.330015644
ENSG00000143387	CTSK	-1.748094896	7.666368949	-2.320944083	0.029536694	0.327502506
ENSG00000165434	PGM2L1	-1.444840809	3.311226167	-2.126385788	0.044452824	0.366525797
ENSG00000116793	PHTF1	-0.71601509	5.28169471	-2.243742898	0.034801813	0.344079576

ENSG00000011258	MBTD1	1.007193173	4.153232533	2.184399247	0.039415068	0.358641419
ENSG00000010072	SPRTN	1.451383605	2.485021925	2.121016566	0.044947204	0.368750171
ENSG00000118276	B4GALT6	-1.352659859	1.646495173	-2.132986077	0.043851795	0.364126158
ENSG00000133937	GSC	-1.027777663	0.910122408	-2.156848734	0.041739403	0.362636645
ENSG00000174233	ADCY6	0.640479467	6.158505946	2.288557684	0.031649764	0.334358662
ENSG00000134013	LOXL2	-1.293654152	7.336233539	-2.312296724	0.030087874	0.328766596
ENSG00000138767	CNOT6L	0.815123795	2.97544969	2.120117302	0.045030487	0.368775243
ENSG00000198807	PAX9	-1.111315045	0.668903945	-2.169585754	0.040649864	0.360866344
ENSG00000165868	HSPA12A	1.734613941	2.764910765	2.137118347	0.04347924	0.364118884
ENSG00000148356	LRSAM1	0.707747491	5.7449847	2.271632713	0.032808209	0.339573615
ENSG00000153832	FBXO36	1.158876778	1.009886749	2.134450052	0.043719479	0.364118884
ENSG00000143367	TUFT1	0.988392321	5.034197673	2.236047808	0.035370959	0.345567417
ENSG00000100949	RABGGTA	0.665504936	6.248207356	2.28874898	0.031636887	0.334358662
ENSG00000142765	SYTL1	1.293549546	5.338773469	2.252415036	0.034170401	0.342370096
ENSG00000119705	SLIRP	-0.905898837	5.267822689	-2.235776742	0.035391161	0.345567417
ENSG00000158477	CD1A	-1.547018449	1.852910681	-2.126334297	0.044457541	0.366525797
ENSG00000165934	CPSF2	-0.61732328	5.937210705	-2.270231622	0.032905821	0.339911286
ENSG00000068489	PRR11	-1.182796146	5.518607131	-2.243323065	0.034832648	0.344079576
ENSG00000189337	KAZN	0.963324801	4.927866102	2.22894112	0.035904092	0.346212821
ENSG00000138080	EMILIN1	-1.588213004	7.126575956	-2.306300263	0.030475602	0.330015644
ENSG00000171202	TMEM126A	-1.119714971	4.024525016	-2.152930473	0.042079837	0.362636645
ENSG00000130595	TNNT3	1.569251478	2.718231246	2.115705804	0.045441057	0.370578675
ENSG00000152782	PANK1	1.055726869	2.614324072	2.105906955	0.046365072	0.374235775
ENSG00000145365	TIFA	1.365449903	2.21051675	2.114908218	0.045515645	0.370620649
ENSG00000167513	CDT1	-1.171627807	4.887483762	-2.206121528	0.037665712	0.35153641
ENSG00000095564	BTAF1	0.982013441	6.064363217	2.278261513	0.032349959	0.337726688
ENSG00000203791	METTL10	0.776519412	0.335231866	2.167913496	0.04079143	0.361003009
ENSG00000138604	GLCE	1.14125823	2.365036567	2.103762675	0.046569509	0.374662991
ENSG00000185811	IKZF1	-1.195575505	0.960004579	-2.148903277	0.042432344	0.362636645
ENSG00000131187	F12	1.214853126	1.674630867	2.10333844	0.046610052	0.374662991
ENSG00000049249	TNFRSF9	-1.45379271	1.532694299	-2.133472218	0.043807817	0.364118884
ENSG00000171291	ZNF439	0.928420272	3.163610843	2.116860298	0.045333287	0.36990359
ENSG00000080166	DCT	1.297294197	0.828136476	2.136197546	0.043562009	0.364118884
ENSG00000167083	GNGT2	-1.041299442	0.801397307	-2.155059953	0.04189451	0.362636645
ENSG00000168309	FAM107A	1.902449431	2.032593754	2.111769151	0.045810271	0.372054328
ENSG00000176194	CIDEA	1.551033117	1.07177639	2.12028949	0.045014529	0.368775243
ENSG00000101294	HM13	-0.784298783	8.484837103	-2.324471946	0.029314499	0.327150639
ENSG00000100055	CYTH4	-1.275621579	4.158551663	-2.161274375	0.041357876	0.362387043
ENSG00000185728	YTHDF3	0.829332649	4.502003558	2.197095994	0.038383837	0.354062901
ENSG00000171163	ZNF692	1.286203853	6.037118383	2.274866001	0.032583956	0.338789152
ENSG00000171714	ANO5	-1.54076419	1.161718561	-2.13290164	0.043859438	0.364126158
ENSG00000108523	RNF167	-0.643093526	6.856119751	-2.294352842	0.031261782	0.334153657
ENSG00000100330	MTMR3	0.677973794	5.455824004	2.250053741	0.034341284	0.342732818
ENSG00000164754	RAD21	0.949244691	6.968851211	2.299992305	0.030888403	0.331965705
ENSG00000018236	CNTN1	1.923368767	3.983442377	2.169801062	0.040631669	0.360866344
ENSG00000230055	CISD3	-1.099743913	3.047710236	-2.103932248	0.046553313	0.374662991
ENSG00000047579	DTNBP1	-0.639582344	5.46342283	-2.242037671	0.034927212	0.344464412
ENSG00000128191	DGCR8	0.551794044	6.140278375	2.275874848	0.032514272	0.338541103
ENSG00000109971	HSPA8	-0.666860402	7.488884745	-2.306441296	0.03046643	0.330015644
ENSG00000104894	CD37	-1.083518152	4.894259187	-2.202760983	0.037931661	0.352114194
ENSG00000006555	TTC22	1.459564487	2.758893515	2.095770498	0.047338631	0.376920467
ENSG00000081665	ZNF506	0.786501788	6.339950968	2.279808794	0.03224384	0.337292067
ENSG00000171385	KCND3	1.234818422	1.381552446	2.106541497	0.046304729	0.374235775
ENSG00000122733	KIAA1045	0.912850202	0.550252562	2.149546695	0.042375846	0.362636645
ENSG00000170458	CD14	-1.782805127	3.95838321	-2.13989543	0.043230468	0.364118884
ENSG00000197647	ZNF433	1.24736169	2.739558897	2.093570227	0.047552362	0.376997816
ENSG00000183780	SLC35F3	1.183333042	0.780765269	2.133799142	0.043778265	0.364118884
ENSG00000203760	CENPW	-1.379089925	3.578953405	-2.118482483	0.045182246	0.369575375
ENSG00000256061	DYX1C1	0.865610896	0.335231866	2.158368418	0.041608037	0.362636645
ENSG00000167759	KLK13	0.857216777	0.335231866	2.157578035	0.041676313	0.362636645
ENSG00000106633	GCK	0.867304408	0.335231866	2.157169082	0.04171168	0.362636645
ENSG00000133574	GIMAP4	-1.158021787	1.356497986	-2.110655686	0.045915189	0.372385831
ENSG00000105664	COMP	2.505253178	6.117174649	2.267461768	0.033099575	0.340344316

ENSG00000185651	UBE2L3	1.181573338	2.34345011	2.083605577	0.048531178	0.379702023
ENSG00000156172	C8orf37	1.150231618	2.256446686	2.086582499	0.048236885	0.378821305
ENSG00000241484	ARHGAP8	1.124702145	1.862954283	2.084872195	0.048405767	0.379388053
ENSG00000254667	AP000783.1	-0.957935061	0.614931131	-2.149293468	0.042398073	0.362636645
ENSG00000198797	BRINP2	-1.093179241	0.901857263	-2.134407388	0.04372333	0.364118884
ENSG00000175643	RMI2	-1.040855789	1.634659462	-2.098322184	0.047091843	0.376039746
ENSG00000174175	SELP	1.53228479	1.532574771	2.094142124	0.047496726	0.376997816
ENSG00000104953	TLE6	1.371355565	1.926366196	2.084936798	0.048399378	0.379388053
ENSG00000107669	ATE1	1.113064782	3.426539642	2.117222465	0.045299526	0.369850998
ENSG00000227051	C14orf132	-1.311532662	1.194288478	-2.117182287	0.045303271	0.369850998
ENSG00000204152	TIMM23B	0.907072071	0.335231866	2.151514105	0.042203512	0.362636645
ENSG00000152939	MARVELD2	1.065518827	4.566590886	2.184825747	0.039380025	0.358625635
ENSG00000254553	RP1-27O5.3	0.886070909	0.335231866	2.150623367	0.042281457	0.362636645
ENSG00000165621	OXGR1	0.886070909	0.335231866	2.150623367	0.042281457	0.362636645
ENSG00000204952	FBXO47	0.886070909	0.335231866	2.150623367	0.042281457	0.362636645
ENSG00000107672	NSMCE4A	0.773293036	5.219463719	2.22173628	0.036452033	0.347279205
ENSG00000204176	SYT15	1.509484855	2.591340092	2.097489425	0.047172256	0.376490273
ENSG00000130701	RBBP8NL	1.535857388	2.436541699	2.074492179	0.049442125	0.380705578
ENSG00000163435	ELF3	2.290887702	4.495554919	2.17599112	0.040111709	0.360460451
ENSG00000173264	GPR137	-0.823340747	4.441519997	-2.165432288	0.0410023	0.361036529
ENSG00000156502	SUPV3L1	0.783700409	5.251517162	2.22373773	0.036299062	0.346874898
ENSG00000080986	NDC80	-1.467252709	4.890284188	-2.174035669	0.040275312	0.36083805
ENSG00000165973	NELL1	-1.680562054	1.276463137	-2.112687637	0.045723887	0.371545062
ENSG00000235531	RP11-383H13.1	-1.287290722	1.102217016	-2.118840741	0.04514895	0.369552516
ENSG00000177842	ZNF620	1.138361383	1.959943047	2.078216747	0.049067996	0.38026669
ENSG00000108561	C1QBP	-0.864803771	6.749229816	-2.273402331	0.032685299	0.338793755
ENSG00000173369	C1QB	-1.844838373	4.928925486	-2.183889059	0.039457025	0.358641419
ENSG00000111665	CDCA3	-1.207358816	4.498791285	-2.154227585	0.041966862	0.362636645
ENSG00000131508	UBE2D2	-0.653416904	6.26572207	-2.2592678	0.033678857	0.341241014
ENSG00000100842	EFS	1.02432144	5.822891676	2.249038054	0.034415026	0.342861061
ENSG00000153130	SCOC	-1.250431573	3.495237077	-2.100519908	0.046880213	0.375387595
ENSG00000114251	WNT5A	-1.346861906	2.199555379	-2.076383813	0.049251795	0.38026669
ENSG00000118193	KIF14	-1.511347688	3.546128478	-2.099986976	0.046931453	0.375387595
ENSG00000143740	SNAP47	1.073226037	4.224486818	2.1609992	0.041381506	0.362387043
ENSG00000198169	ZNF251	0.945132187	4.620582771	2.18236323	0.039582747	0.35922087
ENSG00000198707	CEP290	0.723430695	5.516559551	2.231949698	0.035677508	0.346146062
ENSG00000173409	ARV1	0.895937103	4.299186292	2.160670796	0.041409723	0.362387043
ENSG00000185480	PARPBP	-1.107454584	2.350724165	-2.070387422	0.049857396	0.381988474
ENSG00000129455	KLK8	1.434952918	0.907362346	2.107593821	0.046204811	0.374235775
ENSG00000071994	PDCD2	-0.883061565	3.665775319	-2.10601369	0.046354917	0.374235775
ENSG00000075142	SRI	-0.774100049	6.732753375	-2.265777879	0.033217872	0.340968683
ENSG00000198791	CNOT7	-1.128369541	3.048387748	-2.080251701	0.048864659	0.379757002
ENSG00000245017	RP11-181C3.1	0.86977702	0.521698043	2.131004153	0.044031498	0.364881364
ENSG00000108798	ABI3	-1.255523846	3.60699825	-2.102222357	0.046716863	0.374705512
ENSG00000175084	DES	1.79141604	2.188813796	2.075728896	0.049317616	0.38026669
ENSG00000110321	EIF4G2	-0.633805032	10.47698493	-2.317915957	0.029728644	0.327697544
ENSG00000173064	HECTD4	0.632648606	6.730959765	2.26849451	0.033027212	0.340344316
ENSG00000145431	PDGFC	-1.001050147	4.38689895	-2.146383599	0.042654246	0.363348416
ENSG00000087303	NID2	-1.202690055	6.625461571	-2.261292896	0.033534837	0.341241014
ENSG00000174292	TNK1	1.262274547	3.557212853	2.103405135	0.046603676	0.374662991
ENSG00000267281	RP11-793H13.10	1.178321911	1.117916236	2.093373518	0.047571512	0.376997816
ENSG00000119943	PYROXD2	1.362685896	3.98775374	2.134935655	0.043675669	0.364118884
ENSG00000178695	KCTD12	-1.455835237	3.759583076	-2.107507481	0.046213002	0.374235775
ENSG00000000460	C1orf112	-1.19856554	4.311117675	-2.134992475	0.043670546	0.364118884
ENSG00000117616	C1orf63	0.701647397	6.532499976	2.261488731	0.033520939	0.341241014
ENSG00000177189	RPS6KA3	-0.783808401	5.698055642	-2.225986289	0.0361279	0.346212821
ENSG00000142634	EFHD2	-1.147191862	5.251302657	-2.197810545	0.038326534	0.353753909
ENSG00000105227	PRX	1.275329662	1.378521252	2.077384505	0.049151374	0.38026669
ENSG00000132199	ENOSF1	0.842763009	6.521825446	2.258907091	0.033704569	0.341241014
ENSG00000172554	SNTG2	1.28391023	0.759888838	2.10309951	0.046632899	0.374662991
ENSG00000122679	RAMP3	-1.357394221	1.941902592	-2.079680107	0.048921698	0.379826998
ENSG00000141429	GALNT1	-1.132144418	6.826488567	-2.256816959	0.033853909	0.341241014
ENSG00000148572	NRBF2	-1.004900024	1.777774721	-2.074016404	0.049490099	0.380848036

ENSG00000184708	EIF4ENIF1	0.567315194	5.680157212	2.227458713	0.036016216	0.346212821
ENSG00000146731	CCT6A	-0.529994091	7.859268559	-2.281367316	0.032137272	0.336423295
ENSG00000100591	AHSA1	-0.688762288	6.737016929	-2.25693983	0.033845113	0.341241014
ENSG00000124260	MAGEA10	0.903695505	0.335231866	2.128220929	0.044284975	0.366151671
ENSG00000163357	DCST1	1.127814417	1.263264898	2.078726667	0.049016973	0.380190472
ENSG00000162923	WDR26	0.618193378	7.460959792	2.276024701	0.032503933	0.338541103
ENSG00000114796	KLHL24	0.741619025	5.213661886	2.200771468	0.03808991	0.35260695
ENSG00000169855	ROBO1	-1.1489886	5.706174955	-2.219407962	0.036630723	0.347925348
ENSG00000160883	HK3	-1.342651631	3.658044257	-2.089845929	0.047916106	0.378575844
ENSG00000100077	ADRBK2	-0.744820245	4.930882794	-2.173273202	0.040339267	0.36083805
ENSG00000124743	KLHL31	0.897376977	0.335231866	2.126303573	0.044460357	0.366525797
ENSG00000130208	APOC1	-1.63132103	4.969788835	-2.164909359	0.041046868	0.361226249
ENSG00000129951	LPPR3	1.202040987	1.081792186	2.081920221	0.0486985	0.379702023
ENSG00000170558	CDH2	-2.19793287	3.424294441	-2.078762385	0.049013401	0.380190472
ENSG00000187123	LYPD6	-0.923297014	1.074565319	-2.099914748	0.046938402	0.375387595
ENSG00000143545	RAB13	0.916867926	3.979494727	2.127140649	0.044383712	0.366525797
ENSG00000197312	DDI2	0.664444162	4.847758528	2.17721267	0.040009813	0.36038212
ENSG00000173166	RAPH1	1.100752829	3.67484769	2.100267482	0.046904477	0.375387595
ENSG00000224689	ZNF812	-1.25522522	0.736718738	-2.113364891	0.045660285	0.371220484
ENSG00000160208	RRP1B	0.717485975	5.327456241	2.202742598	0.037933121	0.352114194
ENSG00000166394	CYB5R2	1.125589617	4.361028146	2.14662922	0.042632569	0.363348416
ENSG00000109685	WHSC1	-0.87046945	6.174209795	-2.226294599	0.036104489	0.346212821
ENSG00000197448	GSTK1	0.759547757	7.378112442	2.268004547	0.033061525	0.340344316
ENSG00000102886	GDPD3	1.173415369	3.378811814	2.080043235	0.048885455	0.379757002
ENSG00000254521	SIGLEC12	-1.03135799	1.183290419	-2.091997828	0.047705632	0.377673599
ENSG00000158717	RNF166	-0.99784131	3.796211655	-2.093119573	0.047596245	0.376997816
ENSG00000169432	SCN9A	-0.986759423	0.895326009	-2.102188219	0.046720133	0.374705512
ENSG00000171466	ZNF562	0.885932189	4.769444457	2.165784716	0.040972289	0.361005094
ENSG00000168824	NSG1	-1.481285366	1.675250318	-2.069200642	0.04997804	0.382352715
ENSG00000136144	RCBTB1	0.815496941	4.497014607	2.147845986	0.04252533	0.363038603
ENSG00000123159	GIPC1	0.746752246	7.458623622	2.265181167	0.033259885	0.341098598
ENSG00000143194	MAEL	-1.515644432	1.214276045	-2.090562448	0.047845933	0.378211852
ENSG00000114757	PEX5L	0.993989507	0.751515136	2.09353023	0.047556255	0.376997816
ENSG00000167183	PRR15L	1.158825358	0.873302744	2.085566303	0.048337164	0.379229222
ENSG00000151338	MIPOL1	0.940974915	3.869646446	2.106053337	0.046351145	0.374235775
ENSG00000149948	HMGA2	-1.261028157	0.776461763	-2.104628379	0.046486875	0.374662991
ENSG00000175054	ATR	0.790827663	5.973637066	2.224950687	0.03620664	0.346212821
ENSG00000027075	PRKCH	-0.852530697	3.930032187	-2.094092701	0.047501532	0.376997816
ENSG00000158470	B4GALT5	-0.758130668	5.712007203	-2.207312002	0.037571906	0.35153641
ENSG00000170043	TRAPPC1	-0.71481501	5.699089231	-2.20613292	0.037664814	0.35153641
ENSG00000173486	FKBP2	-0.605837223	6.905666929	-2.246938224	0.034567938	0.343345171
ENSG00000162384	C1orf123	-0.617456316	6.412004967	-2.233561005	0.035556691	0.346104242
ENSG00000137834	SMAD6	-1.025606276	4.047050461	-2.102046499	0.046733713	0.374705512
ENSG00000023697	DERA	-0.972063371	4.994514596	-2.154322095	0.041958642	0.362636645
ENSG00000167315	ACAA2	-0.905194388	4.185754904	-2.106532546	0.04630558	0.374235775
ENSG00000105971	CAV2	1.595274574	3.723637224	2.094324878	0.047478959	0.376997816
ENSG00000125124	BBS2	0.754739355	5.956713908	2.221294155	0.036485904	0.347390966
ENSG00000198763	MT-ND2	0.745765965	7.571053359	2.26340752	0.033385049	0.341241014
ENSG00000126749	EMG1	-0.800253437	5.804132779	-2.202627916	0.037942227	0.352114194
ENSG00000146143	PRIM2	-0.859633098	5.073036673	-2.159936411	0.041472886	0.362636645
ENSG00000163945	UVSSA	1.028981992	4.174304325	2.120139809	0.045028401	0.368775243
ENSG00000118200	CAMSAP2	0.851968605	5.231330346	2.186107293	0.039274896	0.3580839
ENSG00000156804	FBXO32	1.566080615	6.816689987	2.245991507	0.034637081	0.343345171
ENSG00000151552	QDPR	-0.693314954	5.400449013	-2.186993942	0.03920231	0.3580839
ENSG00000243156	MICAL3	0.793671679	5.945119428	2.217845248	0.036751102	0.348435972
ENSG00000169126	ARMC4	-0.974556113	0.873302744	-2.093712332	0.047538532	0.376997816
ENSG00000134686	PHC2	-0.764461431	6.700267604	-2.237435881	0.035267672	0.345567417
ENSG00000100567	PSMA3	-0.79610055	7.074393101	-2.244626495	0.034736996	0.343885306
ENSG00000175166	PSMD2	-0.808943454	7.130884074	-2.245066703	0.034704746	0.343782933
ENSG00000164548	TRA2A	0.586432213	6.585632235	2.237059685	0.035295638	0.345567417
ENSG00000198055	GRK6	-0.667309775	4.93778309	-2.154696038	0.041926129	0.362636645
ENSG00000117289	TXNIP	1.040276243	7.49337501	2.255808229	0.033926199	0.341241014
ENSG00000188368	PRR19	1.017283766	0.901857263	2.07225778	0.04966779	0.381470373

ENSG00000057468	MSH4	0.966796861	0.550252562	2.088143211	0.048083236	0.378821105
ENSG00000149428	HYOU1	-0.624332727	8.419232727	-2.265669673	0.033225487	0.340968683
ENSG00000168575	SLC20A2	-0.853024804	6.072729603	-2.212921856	0.03713271	0.349878336
ENSG00000185842	DNAH14	1.184953345	4.373727058	2.125009901	0.044579043	0.366608888
ENSG00000100207	TCF20	0.805746067	4.670449568	2.142544545	0.042994355	0.364118884
ENSG00000174498	IGDCC3	-1.039082317	0.838216971	-2.084456394	0.048446904	0.379520904
ENSG00000123815	ADCK4	0.822551368	5.879230875	2.207714064	0.037540273	0.35153641
ENSG00000120149	MSX2	-1.273436161	0.769363901	-2.088548719	0.048043386	0.378821105
ENSG00000137804	NUSAP1	-1.068339613	5.636077366	-2.178603506	0.039894081	0.360254047
ENSG00000126822	PLEKHG3	1.024326403	5.520169704	2.189061582	0.039033514	0.358011153
ENSG00000162676	GFI1	-1.08562189	0.854627321	-2.080012914	0.04888848	0.379757002
ENSG00000120742	SERP1	-0.676220586	7.468348506	-2.243164052	0.034844334	0.344079576
ENSG00000161791	FMNL3	-1.04475979	6.535810743	-2.221837513	0.036444282	0.347279205
ENSG00000135048	TMEM2	-0.909671869	6.349645339	-2.212994949	0.037127019	0.349878336
ENSG00000152904	GGPS1	0.701758917	5.282357262	2.174757204	0.040214874	0.360660621
ENSG00000066336	SPI1	-1.533139096	5.396901508	-2.165761459	0.040974269	0.361005094
ENSG00000137965	IFI44	-1.147387884	6.134167712	-2.195913757	0.038478816	0.354178856
ENSG00000120210	INSL6	-1.202736922	0.740809381	-2.082253469	0.048665374	0.379702023
ENSG00000080802	CNOT4	0.70691223	4.802719256	2.138370399	0.043366921	0.364118884
ENSG00000133398	MED10	-0.677355575	5.09858729	-2.144314466	0.042837252	0.363851129
ENSG00000147854	UHRF2	0.833612629	5.087977814	2.15398462	0.041988003	0.362636645
ENSG00000076003	MCM6	-1.005142236	6.699849132	-2.211717108	0.037226635	0.350075752
ENSG00000158042	MRPL17	-0.824515443	5.571059007	-2.171918824	0.040453097	0.360866344
ENSG00000125246	CLYBL	0.867607303	3.856814388	2.071071719	0.049787951	0.381988474
ENSG00000182580	EPHB3	0.873523868	7.95295444	2.242098035	0.034922766	0.344464412
ENSG00000101825	MXRA5	-1.536229753	5.912834241	-2.179357551	0.039831463	0.360254047
ENSG00000122912	SLC25A16	0.646979355	5.710565671	2.181701965	0.039637345	0.359300741
ENSG00000166275	C10orf32	0.750275905	4.404680059	2.105915663	0.046364243	0.374235775
ENSG00000142892	PIGK	-0.713070602	5.300467017	-2.149124585	0.042412903	0.362636645
ENSG00000080573	COL5A3	-1.45278264	7.931688022	-2.236506077	0.035336829	0.345567417
ENSG00000111271	ACAD10	0.698869829	5.135926158	2.150646958	0.042279391	0.362636645
ENSG00000100031	GGT1	-1.255020452	4.388036628	-2.076367364	0.049253447	0.38026669
ENSG00000128203	ASPHD2	-0.879111773	0.550252562	-2.07787618	0.0491021	0.38026669
ENSG00000100228	RAB36	0.974488292	4.058207991	2.081444424	0.048745831	0.379702023
ENSG00000158769	F11R	1.107778216	4.221067323	2.086676941	0.048227575	0.378821305
ENSG00000167543	TP53I13	-0.642396678	5.41733605	-2.154339725	0.041957108	0.362636645
ENSG00000134697	GNL2	-0.761880608	7.042651478	-2.21480377	0.036986422	0.349190098
ENSG00000135480	KRT7	2.737041844	6.763855502	2.212432824	0.03717081	0.349878336
ENSG00000169683	LRRC45	-0.84392172	5.710597465	-2.166166482	0.040939801	0.361005094
ENSG00000172006	ZNF554	0.809618483	4.462397801	2.103180687	0.046625136	0.374662991
ENSG00000157800	SLC37A3	0.646443901	5.916473345	2.183658635	0.039475988	0.358641419
ENSG00000174606	ANGEL2	0.735561957	4.950531776	2.133949604	0.04376467	0.364118884
ENSG00000129255	MPDU1	-0.779845854	4.992909608	-2.126680409	0.044425838	0.366525797
ENSG00000136895	GARNL3	1.085929123	4.163200075	2.080308841	0.048858961	0.379757002
ENSG00000154447	SH3RF1	0.998229518	4.776348309	2.120911545	0.044956923	0.368750171
ENSG00000164944	KIAA1429	0.683813073	7.154731836	2.217154989	0.036804388	0.348647583
ENSG00000125089	SH3TC1	-1.028966171	4.682458738	-2.102598535	0.046680837	0.374705512
ENSG00000149084	HSD17B12	-0.901327591	5.382301109	-2.141167353	0.043116958	0.364118884
ENSG00000167291	TBC1D16	-0.70471101	5.795519892	-2.166530476	0.040908848	0.361005094
ENSG00000014216	CAPN1	-0.640590185	8.104973907	-2.229645775	0.035850905	0.346212821
ENSG00000010610	CD4	-1.502813793	5.28261936	-2.132354349	0.043909003	0.364316632
ENSG00000116039	ATP6V1B1	1.202421894	4.336432391	2.087242231	0.048171882	0.378821305
ENSG00000152601	MBNL1	0.966822342	5.366224857	2.153346443	0.042043577	0.362636645
ENSG00000011295	TTC19	0.844634982	5.334683963	2.149743552	0.042358574	0.362636645
ENSG00000073169	SELO	0.829495412	5.666932263	2.164424246	0.041088251	0.361387753
ENSG00000069188	SDK2	1.447959105	6.164704628	2.179269194	0.039838796	0.360254047
ENSG00000146282	RARS2	-0.71947528	6.603179314	-2.188437583	0.039084386	0.358011153
ENSG00000166295	ANAPC16	0.858334495	5.893363573	2.171834105	0.040460227	0.360866344
ENSG00000121152	NCAPH	-1.255557584	4.439034493	-2.069404146	0.049957334	0.382352715
ENSG00000196262	PPIA	0.597381946	6.093184716	2.177419202	0.039992608	0.36038212
ENSG00000126767	ELK1	-0.677158559	5.259301489	-2.130326983	0.04409305	0.365099759
ENSG00000174231	PRPF8	-0.619588678	9.086740681	-2.23393936	0.035528376	0.346104242
ENSG00000116830	TTF2	0.890000901	5.354389666	2.143035646	0.042950712	0.364094879

ENSG00000173230	GOLGB1	0.729736599	7.005229513	2.202004035	0.0379918	0.352114194
ENSG00000143924	EML4	-0.869598912	6.308718842	-2.175738133	0.040132841	0.360460451
ENSG00000109436	TBC1D9	-1.177984068	4.871293649	-2.103519755	0.04659272	0.374662991
ENSG00000120594	PLXDC2	-1.087724284	5.804677219	-2.151351626	0.04221772	0.362636645
ENSG00000177963	RIC8A	-0.648152434	6.556791878	-2.185706203	0.039307771	0.358175516
ENSG00000058063	ATP11B	-1.039135166	5.496917578	-2.126138306	0.044475503	0.366525797
ENSG00000105887	MTPN	0.761908067	5.583211207	2.152354025	0.042130132	0.362636645
ENSG00000115504	EHBP1	0.616879269	6.180763746	2.174647201	0.040224083	0.360660621
ENSG00000066027	PPP2R5A	0.720135092	5.85724255	2.162472852	0.0412551	0.362387043
ENSG00000197070	ARRDC1	0.994712979	4.801423127	2.104286374	0.046519505	0.374662991
ENSG00000121900	TMEM54	1.030851485	5.410774377	2.140195261	0.043203686	0.364118884
ENSG00000146909	NOM1	0.721351011	5.339474802	2.136451881	0.043539133	0.364118884
ENSG00000113615	SEC24A	-0.73061984	5.51328068	-2.136786927	0.043509014	0.364118884
ENSG00000115902	SLC1A4	-0.959582564	4.533954902	-2.0732326	0.049569224	0.381083143
ENSG00000100979	PLTP	-1.378411659	8.139074443	-2.21302658	0.037124556	0.349878336
ENSG00000158286	RNF207	1.02779117	4.549150637	2.086735938	0.04822176	0.378821305
ENSG00000145901	TNIP1	-0.624496855	7.510598842	-2.200193765	0.038135973	0.352825212
ENSG00000162849	KIF26B	1.458404369	4.293728051	2.07051794	0.049844143	0.381988474
ENSG00000117984	CTSD	-0.669068761	4.766371925	-2.086710887	0.048224229	0.378821305
ENSG00000094804	CDC6	-1.338316798	4.914658241	-2.082020154	0.048688564	0.379702023
ENSG00000014138	POLA2	-0.84221061	5.696718464	-2.139228946	0.043290055	0.364118884
ENSG00000106483	SFRP4	-2.248844052	5.820071813	-2.133424299	0.04381215	0.364118884
ENSG00000164751	PEX2	0.789854449	5.638960578	2.145338456	0.042746597	0.363740156
ENSG00000005059	CCDC109B	-0.875821558	5.072200029	-2.095691758	0.047346265	0.376920467
ENSG00000113648	H2AFY	-0.654290959	6.64682235	-2.173234991	0.040342474	0.36083805
ENSG00000081870	HSPB11	-0.975256286	5.425248342	-2.113918864	0.04560832	0.370990225
ENSG00000101811	CSTF2	-0.9769506	5.367935296	-2.110580456	0.045922286	0.372385831
ENSG00000115355	CCDC88A	-1.014197004	6.620445291	-2.166187621	0.040938003	0.361005094
ENSG00000125991	ERGIC3	-0.528684881	9.233727519	-2.215270566	0.036950217	0.349190098
ENSG00000115457	IGFBP2	-1.377868654	6.459546702	-2.161862049	0.041307451	0.362387043
ENSG00000087842	PIR	-1.090543573	5.482286948	-2.115265578	0.045482212	0.370578675
ENSG00000099622	CIRBP	0.661044879	7.969353458	2.196279845	0.038449382	0.354178856
ENSG00000155324	GRAMD3	1.148458746	4.626237107	2.074440593	0.049447324	0.380705578
ENSG00000147471	PROSC	-0.70707289	5.504749826	-2.115215744	0.045486873	0.370578675
ENSG00000064666	CNN2	-0.699030983	6.378803432	-2.154274162	0.041962811	0.362636645
ENSG00000076706	MCAM	-0.763674456	7.834587723	-2.186920945	0.039208282	0.3580839
ENSG00000076685	NT5C2	0.700328023	6.411891794	2.156648618	0.041756729	0.362636645
ENSG00000100324	TAB1	0.69967934	4.981034553	2.091232104	0.047780431	0.377884451
ENSG00000113407	TARS	-0.75948424	7.667902495	-2.17849346	0.039903227	0.360254047
ENSG00000156711	MAPK13	-1.1666314	5.406000472	-2.081139652	0.04877617	0.379702023
ENSG00000188229	TUBB4B	-1.02250518	6.43101446	-2.142472773	0.043000737	0.364118884
ENSG00000115459	ELMOD3	0.73298302	4.943791373	2.081512633	0.048739043	0.379702023
ENSG00000163382	APOA1BP	-0.671833389	6.935614045	-2.157565453	0.041677401	0.362636645
ENSG00000104881	PPP1R13L	0.959777253	6.81421585	2.158698064	0.041579591	0.362636645
ENSG00000120071	KANSL1	0.610227717	5.74114919	2.121422967	0.044909612	0.368747627
ENSG00000088298	EDEM2	-0.619865167	5.776110535	-2.113977892	0.045602786	0.370990225
ENSG00000165609	NUDT5	-0.775670666	6.367149264	-2.136825273	0.043505568	0.364118884
ENSG00000102595	UGGT2	0.772485685	6.151140148	2.135521785	0.043622843	0.364118884
ENSG00000126067	PSMB2	-0.735281065	7.963557571	-2.174802232	0.040211105	0.360660621
ENSG00000105699	LSR	2.300080238	6.698847454	2.150268232	0.04231257	0.362636645
ENSG00000164930	FZD6	1.447067569	5.104810406	2.08105492	0.048784608	0.379702023
ENSG00000101052	IFT52	-0.747373988	5.879253566	-2.111082211	0.045874974	0.372385831
ENSG00000164808	SPIDR	0.658085415	6.770541021	2.147158223	0.042585916	0.363290101
ENSG00000168090	COPS6	-0.675646807	7.636888507	-2.160917532	0.041388522	0.362387043
ENSG00000125970	RALY	-0.620894488	8.167596033	-2.170196191	0.040598297	0.360866344
ENSG00000228474	OST4	-0.776436724	7.51393113	-2.156242537	0.041791909	0.362636645
ENSG00000108292	MLLT6	0.738095484	7.370715529	2.152558132	0.042112317	0.362636645
ENSG00000096696	DSP	1.808487624	6.802622821	2.138289082	0.043374208	0.364118884
ENSG00000108848	LUC7L3	0.657021279	8.644145589	2.17135156	0.04050086	0.360866344
ENSG00000173715	C11orf80	0.884039211	5.359429943	2.081386498	0.048751596	0.379702023
ENSG00000028310	BRD9	-0.687522046	6.47602821	-2.121541506	0.044898652	0.368747627
ENSG00000075426	FOSL2	-0.767371543	5.386004422	-2.073535901	0.049538592	0.38103434
ENSG00000104517	UBR5	0.775761533	7.924016476	2.156583592	0.041762361	0.362636645

ENSG00000072195	SPEG	1.71631606	5.865863794	2.099607036	0.046968014	0.375433068
ENSG00000142186	SCYL1	-0.63677992	7.411319325	-2.14026397	0.043197551	0.364118884
ENSG00000064961	HMG20B	0.732952925	6.639466003	2.125278864	0.044554344	0.366597905
ENSG00000105856	HBP1	0.707742357	5.804341167	2.094789605	0.047433807	0.376997816
ENSG00000144136	SLC20A1	-0.682491487	5.896969223	-2.088731341	0.048025449	0.378821105
ENSG00000159086	PAXBP1	0.786393856	5.775137876	2.088949909	0.04800399	0.378821105
ENSG00000153147	SMARCA5	-0.804884712	7.68358269	-2.136468823	0.04353761	0.364118884
ENSG00000183963	SMTN	1.053922104	7.666801094	2.138826403	0.043326079	0.364118884
ENSG00000123066	MED13L	0.748164943	6.196632109	2.097113112	0.047208635	0.376589064
ENSG00000177830	CHID1	-0.602905527	7.85175999	-2.133845017	0.04377412	0.364118884
ENSG00000175662	TOM1L2	0.651750973	5.748182383	2.076373823	0.049252798	0.38026669
ENSG00000173039	RELA	-0.632562568	6.788105756	-2.10717989	0.046244091	0.374235775
ENSG00000108528	SLC25A11	-0.55828931	6.193804344	-2.088054024	0.048092004	0.378821105
ENSG00000101191	DIDO1	0.769193024	6.395986928	2.0989803	0.04702838	0.375724189
ENSG00000160194	NDUFV3	0.668504024	5.69227873	2.070449732	0.049851069	0.381988474
ENSG00000047249	ATP6V1H	0.653488082	7.153832705	2.117745071	0.04525085	0.369807858
ENSG00000133059	DSTYK	0.796778135	6.112375191	2.087292238	0.048166958	0.378821305
ENSG00000234127	TRIM26	-0.6485953	6.088752278	-2.074631732	0.049428061	0.380705578
ENSG00000129038	LOXL1	-1.26271541	6.153596358	-2.072013398	0.049692527	0.381470373
ENSG00000054793	ATP9A	1.0293807	6.173282623	2.082672216	0.048623778	0.379702023
ENSG00000166710	B2M	-1.028353726	10.6441639	-2.1528783	0.042084387	0.362636645
ENSG00000215021	PHB2	-0.728332701	8.579323033	-2.130872928	0.04404342	0.364881364
ENSG00000172725	CORO1B	-0.708626445	7.023523642	-2.100523834	0.046879836	0.375387595
ENSG00000019582	CD74	-1.552278367	10.43012788	-2.14864394	0.042455135	0.362636645
ENSG00000137073	UBAP2	0.667983876	7.089883015	2.096643625	0.047254056	0.376759851
ENSG00000171346	KRT15	2.907800098	6.868122133	2.091593451	0.04774512	0.377795604
ENSG00000171793	CTPS1	-0.810923875	6.606934492	-2.076532075	0.049236905	0.38026669
ENSG00000145592	RPL37	0.815135242	6.678406726	2.081469039	0.048743381	0.379702023
ENSG00000082641	NFE2L1	0.713820627	6.689354713	2.081502313	0.04874007	0.379702023
ENSG00000178252	WDR6	0.685225162	6.637448001	2.076497498	0.049240377	0.38026669
ENSG00000177600	RPLP2	-0.800070434	7.582560733	-2.093135205	0.047594722	0.376997816
ENSG00000136861	CDK5RAP2	0.669946621	7.183113923	2.082512458	0.048639644	0.379702023
ENSG00000054267	ARID4B	0.584505154	7.559943547	2.088174904	0.04808012	0.378821105
ENSG00000047410	TPR	0.607361253	8.447093997	2.095694291	0.047346019	0.376920467
ENSG00000005483	KMT2E	0.778769495	7.861649723	2.086077872	0.048286659	0.379022494
ENSG00000161202	DVL3	-0.538452303	7.612216062	-2.075905467	0.049299862	0.38026669
ENSG00000133422	MORC2	0.578209096	7.489178851	2.072153227	0.049678372	0.381470373
ENSG00000104852	SNRNP70	0.731515415	8.774525929	2.078042087	0.049085484	0.38026669
ENSG00000167615	LENG8	0.93688599	8.196854917	2.069963656	0.049900444	0.382131709
ENSG00000068697	LAPTM4A	0.531833442	9.303673296	2.076138538	0.049276437	0.38026669
