# Supplementary material for: Genomic and transcriptomic heterogeneity in metaplastic carcinomas of the breast
Source: NPJ Breast Cancer. 2017 Dec 1;3:48. doi: 10.1038/s41523-017-0048-0 (PMC5711926; doi:10.1038/s41523-017-0048-0)
Supplement: Supplementary file 27 — Supplementary Table 15 [file 41523_2017_48_MOESM27_ESM.pdf]

Supplementary Table 15: Summary of differentially expressed genes between squmous and non-squamous MBCs according to RNA-sequencing. Statistics calculated using the limma package. Genes with p-value <0.05 have been included.

gene_id	gene_name	Fold Change (log2)	Average expression	t-statistic	p value	adjusted p value
ENSG00000101443	WFDC2	4.515328087	3.640149448	6.956069668	4.90E-07	0.007683082
ENSG00000075643	MOCOS	3.129218049	4.480391282	5.389540992	1.92E-05	0.15048159
ENSG00000100968	NFATC4	-1.896907642	6.044750167	-5.046099914	4.43E-05	0.173366852
ENSG00000135373	EHF	4.313240059	3.253279891	5.139445241	3.52E-05	0.173366852
ENSG00000008394	MGST1	2.654593562	4.110039318	4.955762265	5.52E-05	0.173366852
ENSG00000111319	SCNN1A	3.416232773	3.479685328	4.865157629	6.90E-05	0.180460537
ENSG00000156711	MAPK13	1.975904135	5.406000472	4.626169822	0.00012428	0.232355633
ENSG00000139055	ERP27	3.42962995	3.016179545	4.611677406	0.000128801	0.232355633
ENSG00000139192	TAPBPL	1.72584989	5.181494205	4.351210102	0.000244969	0.263895636
ENSG00000092929	UNC13D	1.670752079	6.516103904	4.308428894	0.000272258	0.263895636
ENSG00000183549	ACSM5	-2.51809847	2.013808028	-4.597836659	0.000133274	0.232355633
ENSG00000069188	SDK2	-2.931721667	6.164704628	-4.342060242	0.000250565	0.263895636
ENSG00000168269	FOXI1	2.409645632	0.753748539	4.502459598	0.000168635	0.263895636
ENSG00000121316	PLBD1	1.802259609	5.759750762	4.144902347	0.000407585	0.283036162
ENSG00000114805	PLCH1	2.533093675	2.499713099	4.422156295	0.000205607	0.263895636
ENSG00000188039	NWD1	2.55828608	3.500803952	4.252238712	0.000312764	0.272642955
ENSG00000087916	SLC6A14	3.402506376	2.392306134	4.312377643	0.000269616	0.263895636
ENSG00000135525	MAP7	2.297565452	5.189510156	4.089835425	0.000466839	0.283036162
ENSG00000178882	FAM101A	-2.734671714	2.176730215	-4.288607304	0.000285911	0.263895636
ENSG00000006747	SCIN	-4.223142712	4.637592402	-4.317947055	0.000265935	0.263895636
ENSG00000079691	LRRC16A	1.663865258	5.322273932	4.070640624	0.000489447	0.283036162
ENSG00000156689	GLYATL2	3.688477817	2.696580768	4.163443697	0.000389368	0.283036162
ENSG00000153404	PLEKHG4B	2.995138754	3.956441829	4.081146123	0.000476942	0.283036162
ENSG00000118849	RARRES1	3.222499729	4.574652016	4.059739986	0.000502766	0.283036162
ENSG00000137673	MMP7	4.603194126	4.463580776	4.057885832	0.000505067	0.283036162
ENSG00000115221	ITGB6	3.18548853	2.534879295	4.174649774	0.000378754	0.283036162
ENSG00000163220	S100A9	3.27251268	3.079196558	4.068522142	0.000492007	0.283036162
ENSG00000163347	CLDN1	2.955139153	3.612153466	4.020485461	0.000553784	0.29963551
ENSG00000143546	S100A8	4.024492319	2.805910866	3.988216927	0.000599547	0.303189791
ENSG00000144681	STAC	2.332728555	2.037173828	4.100259068	0.000455001	0.283036162
ENSG00000114209	PDCD10	1.529601222	6.191874676	3.84920934	0.000843513	0.330457532
ENSG00000143556	S100A7	3.889364968	1.754574342	4.001398527	0.000580416	0.303189791
ENSG00000139182	CLSTN3	1.93181163	6.402670292	3.820862626	0.000904195	0.330457532
ENSG00000110195	FOLR1	3.079899215	3.731826642	3.878223875	0.000785572	0.330457532
ENSG00000010292	NCAPD2	1.418500979	7.83877274	3.803817348	0.000942739	0.330457532
ENSG00000165801	ARHGEF40	-1.648911267	7.277297866	-3.790874781	0.000973086	0.330457532
ENSG00000106541	AGR2	3.134667297	2.656697222	3.975680532	0.000618321	0.303189791
ENSG00000146648	EGFR	2.56757236	4.433415805	3.817692413	0.000911244	0.330457532
ENSG00000119900	OGFRL1	1.843870951	4.515783226	3.810789067	0.000926782	0.330457532
ENSG00000105270	CLIP3	-2.650973035	4.169370774	-3.955231738	0.000650203	0.3091616
ENSG00000189334	S100A14	3.581624627	3.37589422	3.895783186	0.000752438	0.330457532
ENSG00000155066	PROM2	3.029920075	5.47104338	3.770839665	0.001021974	0.330457532
ENSG00000049540	ELN	3.526430208	8.195301722	3.741688783	0.001097463	0.339471698
ENSG00000115112	TFCP2L1	3.389140791	3.267134027	3.859731287	0.000822028	0.330457532
ENSG00000131080	EDA2R	-1.906321973	1.654720457	-3.872225392	0.000797218	0.330457532
ENSG00000162366	PDZK1IP1	3.254857429	3.330422238	3.846545511	0.000849039	0.330457532
ENSG00000139083	ETV6	1.37789315	5.713728043	3.713116686	0.001176779	0.355093162
ENSG00000005243	COPZ2	-1.401012595	6.966771712	-3.694300337	0.00123207	0.36476252
ENSG00000243244	STON1	-1.984350358	1.860178705	-3.817992157	0.000910575	0.330457532
ENSG00000111266	DUSP16	2.18959844	3.796712321	3.766865644	0.001031956	0.330457532
ENSG00000137747	TMPRSS13	3.195781143	3.292142311	3.786042504	0.000984662	0.330457532
ENSG00000058063	ATP11B	1.537316836	5.496917578	3.635979224	0.001420197	0.39095271
ENSG00000060140	STYK1	2.496381081	2.151313175	3.768424123	0.00102803	0.330457532
ENSG00000160183	TMPRSS3	3.267925487	4.065911312	3.625663456	0.001456294	0.393977647
ENSG00000144843	ADPRH	1.992716201	2.025191178	3.739490069	0.001103375	0.339471698
ENSG00000157927	RADIL	-3.048934755	2.694097559	-3.653106575	0.001362197	0.39095271
ENSG00000104689	TNFRSF10A	2.191536277	3.148999921	3.61742288	0.001485776	0.395140738
ENSG00000181333	HEPHL1	1.777637313	0.708859833	3.642053289	0.001399355	0.39095271

ENSG00000196586	MYO6	1.51885587	7.270465627	3.491956074	0.002014068	0.480354688
ENSG00000006074	CCL18	2.826721252	1.625277255	3.643073807	0.001395883	0.39095271
ENSG00000182621	PLCB1	1.893160003	4.248801695	3.511108252	0.001922907	0.478925993
ENSG00000111711	GOLT1B	1.626009336	4.444997807	3.488175868	0.002032555	0.480354688
ENSG00000130005	GAMT	-2.611748218	4.0204151	-3.572587502	0.001656747	0.426164162
ENSG00000147676	MAL2	3.572102644	3.397660436	3.527900049	0.001846314	0.467266296
ENSG00000135847	ACBD6	-0.892728402	6.195960368	-3.432526172	0.002324685	0.493334438
ENSG00000135114	OASL	2.009691582	3.726596362	3.467928998	0.002134429	0.492519556
ENSG00000137648	TMPRSS4	2.200464645	1.245814975	3.59298898	0.00157668	0.412328131
ENSG00000060138	YBX3	1.597724232	6.175090536	3.426611713	0.002358045	0.493334438
ENSG00000165929	TC2N	2.515820321	4.388384567	3.426890787	0.00235646	0.493334438
ENSG00000075035	WSCD2	-2.931389538	2.046062446	-3.438147874	0.002293404	0.493334438
ENSG00000198682	PAPSS2	-1.856707652	5.448429387	-3.410594725	0.00245075	0.499411931
ENSG00000153902	LGI4	-3.030744153	3.012389533	-3.459980575	0.002175773	0.493334438
ENSG00000198732	SMOC1	3.128438699	4.362869389	3.388465322	0.002584706	0.517882696
ENSG00000188643	S100A16	2.11380029	5.717355829	3.364224233	0.002739639	0.53734595
ENSG00000163710	PCOLCE2	-3.28556836	4.897724799	-3.44585951	0.002251162	0.493334438
ENSG00000133985	TTC9	1.780585242	0.960004579	3.492611993	0.002010877	0.480354688
ENSG00000128383	APOBEC3A	2.110855848	1.523493751	3.484417872	0.002051097	0.480354688
ENSG00000052344	PRSS8	2.635346383	3.944560068	3.3848272	0.002607401	0.517882696
ENSG00000138028	CGREF1	-2.767682722	3.990020033	-3.442186491	0.002271186	0.493334438
ENSG00000178776	C5orf46	2.387458115	1.991769246	3.420323916	0.002394023	0.4942712
ENSG00000196218	RYR1	1.492065843	5.745320312	3.294145909	0.003240138	0.579579831
ENSG00000112297	AIM1	1.802247431	5.300045665	3.292813963	0.003250464	0.579579831
ENSG00000068650	ATP11A	1.590283284	4.940970591	3.279293939	0.003357096	0.585291058
ENSG00000136444	RSAD1	-1.140355013	4.668158177	-3.298820258	0.003204154	0.579579831
ENSG00000181856	SLC2A4	-1.698699238	1.541426374	-3.325624401	0.003005191	0.575054281
ENSG00000166523	CLEC4E	2.214322277	2.126195183	3.354930255	0.002801409	0.542677827
ENSG00000117461	PIK3R3	1.274422165	4.924691044	3.245668836	0.003637235	0.600402038
ENSG00000112367	FIG4	1.329103983	4.596601546	3.231992636	0.003757548	0.600402038
ENSG00000133424	LARGE	-1.686451602	4.597452005	-3.266245655	0.003463225	0.587035913
ENSG00000132470	ITGB4	3.108099443	7.873610011	3.2254828	0.003816164	0.600402038
ENSG00000146282	RARS2	1.009119489	6.603179314	3.215106064	0.003911433	0.603275791
ENSG00000256060	TRAPPC2P1	-1.377207236	1.261031495	-3.264397545	0.003478517	0.587035913
ENSG00000106400	ZNHIT1	-1.220843951	6.372355427	-3.204433577	0.00401182	0.604084742
ENSG00000018625	ATP1A2	-2.207598262	1.548545408	-3.249743999	0.003602112	0.600402038
ENSG00000029153	ARNTL2	2.008423453	3.835490482	3.224354752	0.00382641	0.600402038
ENSG00000005001	PRSS22	2.60692017	2.28406791	3.298502377	0.003206589	0.579579831
ENSG00000163814	CDCP1	2.302613271	3.847117831	3.224377752	0.003826201	0.600402038
ENSG00000134709	HOOK1	2.690045381	2.573596517	3.293922005	0.003241872	0.579579831
ENSG00000197977	ELOVL2	2.267090108	1.37059729	3.308037662	0.003134332	0.579579831
ENSG00000132698	RAB25	3.220745478	3.902436575	3.227836498	0.003794869	0.600402038
ENSG00000173598	NUDT4	-1.318762149	4.889159526	-3.214011099	0.003921619	0.603275791
ENSG00000177694	NAALADL2	2.113973157	2.985537536	3.264298238	0.003479341	0.587035913
ENSG00000103175	WFDC1	-2.158486384	3.078362532	-3.285248527	0.00330972	0.583514804
ENSG00000185479	KRT6B	4.706418041	4.578949113	3.181908522	0.004231946	0.612143989
ENSG00000139178	C1RL	1.714085192	4.408759213	3.172224913	0.004330129	0.612143989
ENSG00000047621	C12orf4	1.302500469	4.728767927	3.166731235	0.004386806	0.612143989
ENSG00000004478	FKBP4	1.281198506	5.878471994	3.157472441	0.004483953	0.612143989
ENSG00000184702	SEPT5	-1.561060339	6.849174959	-3.156439927	0.004494915	0.612143989
ENSG00000113532	ST8SIA4	2.095349545	2.660585014	3.197241305	0.004080873	0.604084742
ENSG00000164151	KIAA0947	1.349579637	4.879873501	3.154810902	0.004512262	0.612143989
ENSG00000005513	SOX8	-2.977658499	2.241349383	-3.178797179	0.004263255	0.612143989
ENSG00000182287	AP1S2	1.895097083	2.750984241	3.192988803	0.004122242	0.604505538
ENSG00000084463	WBP11	1.068082296	5.519058401	3.148636346	0.004578602	0.612143989
ENSG00000147454	SLC25A37	1.38590819	5.206315698	3.139346342	0.004680192	0.612143989
ENSG00000105929	ATP6V0A4	2.813104658	1.823558371	3.198999254	0.00406389	0.604084742
ENSG00000155463	OXA1L	-1.019785498	7.210476204	-3.125334946	0.004837541	0.612143989
ENSG00000102287	GABRE	1.857608781	3.503922008	3.132274002	0.004758987	0.612143989
ENSG00000183955	SETD8	-1.655808724	2.651297198	-3.202986428	0.004025622	0.604084742
ENSG00000075223	SEMA3C	2.899507526	4.623546949	3.085469568	0.005313649	0.648439713
ENSG00000023697	DERA	1.262450865	4.994514596	3.078045231	0.00540717	0.652645368
ENSG00000112237	CCNC	1.133094962	6.281804054	3.072800945	0.005474182	0.653714866

ENSG00000234127	TRIM26	0.913874233	6.088752278	3.070847031	0.005499354	0.653714866
ENSG00000197933	ZNF823	1.814729225	1.559993964	3.166318173	0.004391097	0.612143989
ENSG00000148488	ST8SIA6	2.17525957	1.472573303	3.144612529	0.00462234	0.612143989
ENSG00000145365	TIFA	-1.945159679	2.21051675	-3.12907511	0.004795046	0.612143989
ENSG00000168792	ABHD15	-1.415739352	1.606104943	-3.128706068	0.004799223	0.612143989
ENSG00000133488	SEC14L4	2.098323379	1.463260816	3.149183071	0.00457269	0.612143989
ENSG00000126217	MCF2L	1.402580615	5.646222685	3.062036311	0.005614249	0.657411837
ENSG00000115339	GALNT3	2.51923669	4.682464989	3.062280177	0.005611038	0.657411837
ENSG00000198756	COLGALT2	-1.825492508	1.27548523	-3.084082632	0.005331	0.648439713
ENSG00000143545	RAB13	-1.500254998	3.979494727	-3.102563299	0.005104214	0.638548513
ENSG00000205667	ARSH	1.178342213	0.385114037	3.085359846	0.005315019	0.648439713
ENSG00000150787	PTS	1.29867901	5.19192685	3.043363613	0.005865431	0.666916543
ENSG00000157326	DHRS4	-1.779459644	3.097161135	-3.125384367	0.004836977	0.612143989
ENSG00000153132	CLGN	2.001011724	2.166617138	3.100621662	0.005127596	0.638548513
ENSG00000143217	PVRL4	2.695238404	3.649904717	3.05866444	0.005658828	0.657723505
ENSG00000085117	CD82	1.445060754	6.503032027	3.02357259	0.00614344	0.693501544
ENSG00000132423	COQ3	1.23261309	3.507752928	3.043978912	0.005856985	0.666916543
ENSG00000111912	NCOA7	0.950660974	4.911252793	3.010386218	0.006335651	0.698959773
ENSG00000135316	SYNCRIP	1.039509321	4.204391538	3.008072112	0.006369973	0.698959773
ENSG00000109099	PMP22	-1.20727174	6.468701092	-2.993217638	0.006594574	0.710270921
ENSG00000075651	PLD1	1.178897683	5.338845846	2.973700167	0.006901246	0.710270921
ENSG00000173821	RNF213	0.795437104	8.26058481	2.988143712	0.006673018	0.710270921
ENSG00000077092	RARB	-2.211687727	3.216061183	-3.048900526	0.005789844	0.666916543
ENSG00000019549	SNAI2	-1.861494006	5.248265477	-2.970382422	0.006954718	0.710270921
ENSG00000114346	ECT2	1.651755009	5.534046004	2.966016701	0.007025685	0.710270921
ENSG00000160352	ZNF714	1.340241356	4.37418594	2.963761521	0.007062615	0.710270921
ENSG00000085831	TTC39A	2.021680212	3.069226434	2.991630235	0.00661902	0.710270921
ENSG00000105141	CASP14	3.347233902	1.435496658	3.009868742	0.006343311	0.698959773
ENSG00000176956	LY6H	-1.222311586	0.844748225	-2.971382031	0.006938566	0.710270921
ENSG00000175066	GK5	1.403834847	4.548729921	2.955417772	0.007200873	0.710622045
ENSG00000140853	NLRC5	1.348210738	6.219396247	2.957704046	0.007162734	0.710622045
ENSG00000109182	CWH43	1.931856329	0.951511054	3.010497471	0.006334006	0.698959773
ENSG00000011347	SYT7	2.390487048	3.59817579	2.963874686	0.007060758	0.710270921
ENSG00000163993	S100P	3.348761111	3.496233524	2.961079164	0.007106783	0.710270921
ENSG00000101955	SRPX	-1.976773721	5.738057986	-2.938916796	0.007481965	0.724398982
ENSG00000100626	GALNT16	1.8541118	2.57261348	2.973730697	0.006900756	0.710270921
ENSG00000146476	C6orf211	1.643046347	2.21962407	2.97692707	0.006849617	0.710270921
ENSG00000143257	NR1I3	-1.319490577	0.916653661	-2.948294879	0.007320948	0.717458259
ENSG00000026559	KCNG1	-2.280553714	3.34651603	-2.999305391	0.006501623	0.708451117
ENSG00000162669	HFM1	-1.398795432	1.300084562	-2.936434725	0.007525144	0.724398982
ENSG00000153292	GPR110	1.753794185	1.285288164	2.978125367	0.006830539	0.710270921
ENSG00000141424	SLC39A6	1.589376354	6.625528323	2.916013163	0.007889566	0.735110272
ENSG00000146263	MMS22L	1.125839975	4.593667316	2.897805011	0.008228656	0.736281602
ENSG00000170775	GPR37	-1.654437511	1.209627052	-2.913170529	0.007941611	0.735110272
ENSG00000115590	IL1R2	2.038178963	1.68629104	2.945909028	0.007361595	0.717458259
ENSG00000118046	STK11	-1.16568365	6.449135841	-2.896232156	0.008258592	0.736281602
ENSG00000007237	GAS7	-2.726376543	4.757002378	-2.910537453	0.007990111	0.735110272
ENSG00000105281	SLC1A5	-1.096060542	7.664066568	-2.901148851	0.008165359	0.736281602
ENSG00000116791	CRYZ	1.287222521	4.741289208	2.885225557	0.008471001	0.738958136
ENSG00000060237	WNK1	1.134279458	7.354051169	2.90052629	0.008177108	0.736281602
ENSG00000124102	PI3	2.740485408	1.981472441	2.92366308	0.00775112	0.733835394
ENSG00000184012	TMPRSS2	2.300132901	2.846746897	2.918840793	0.00783812	0.735110272
ENSG00000166086	JAM3	-1.655567539	4.213853655	-2.898616284	0.008213256	0.736281602
ENSG00000171617	ENC1	-1.969260113	2.82646416	-2.932238928	0.007598679	0.727017491
ENSG00000111247	RAD51AP1	1.967145623	3.20686316	2.881930735	0.008535593	0.738958136
ENSG00000162078	ZG16B	2.13849003	1.360588629	2.922975175	0.007763474	0.733835394
ENSG00000214113	LYRM4	1.131641207	4.587728014	2.864485765	0.008885485	0.739854509
ENSG00000064115	TM7SF3	1.276251037	6.383749267	2.874915137	0.008674695	0.738958136
ENSG00000154175	ABI3BP	-2.119372143	4.342426886	-2.883378932	0.008507145	0.738958136
ENSG00000003056	M6PR	1.081419401	6.447588264	2.869000794	0.008793639	0.738958136
ENSG00000099814	CEP170B	0.863615152	6.294876481	2.86320829	0.008911637	0.739854509
ENSG00000103067	ESRP2	2.330204081	3.587736969	2.868862484	0.008796439	0.738958136
ENSG00000085741	WNT11	1.895962593	2.017545485	2.909396887	0.008011207	0.735110272

ENSG00000048028	USP28	1.019111868	5.53403531	2.852370032	0.009136486	0.741221825
ENSG00000105289	TJP3	2.569841492	3.308433631	2.870238613	0.008768617	0.738958136
ENSG00000125398	SOX9	1.302283404	5.085935038	2.843550981	0.00932342	0.741221825
ENSG00000134759	ELP2	1.148128299	6.802278963	2.850651901	0.009172622	0.741221825
ENSG00000178222	RNF212	-2.753111687	2.944333411	-2.874400527	0.008684984	0.738958136
ENSG00000204525	HLA-C	1.284526532	11.32801727	2.868469804	0.008804393	0.738958136
ENSG00000007866	TEAD3	1.094777469	5.871825837	2.837096927	0.009462525	0.741221825
ENSG00000139190	VAMP1	1.544663725	2.102155485	2.88870681	0.008403262	0.738958136
ENSG00000054282	SDCCAG8	-0.880523825	6.145882318	-2.828792569	0.009644416	0.741221825
ENSG00000154127	UBASH3B	1.695017401	2.826443138	2.838310588	0.009436217	0.741221825
ENSG00000092330	TINF2	-0.902684126	5.297323047	-2.818373805	0.009877324	0.741221825
ENSG00000165475	CRYL1	-1.493693255	4.606502995	-2.824384256	0.009742318	0.741221825
ENSG00000184838	PRR16	1.608588519	1.343435479	2.868358374	0.008806652	0.738958136
ENSG00000244045	TMEM199	-1.201255792	0.851279478	-2.822025568	0.009795088	0.741221825
ENSG00000136986	DERL1	-0.96895734	6.888546353	-2.822870263	0.009776159	0.741221825
ENSG00000161911	TREML1	-1.351306336	0.911660197	-2.816945052	0.009909677	0.741221825
ENSG00000197226	TBC1D9B	-0.764898428	7.849425862	-2.827823267	0.009665862	0.741221825
ENSG00000221818	EBF2	-1.405278715	1.313842683	-2.817202441	0.009903842	0.741221825
ENSG00000008323	PLEKHG6	2.394425122	3.532198151	2.816484894	0.009920119	0.741221825
ENSG00000168952	STXBP6	-2.15318797	2.341215016	-2.853200543	0.009119067	0.741221825
ENSG00000111371	SLC38A1	1.505618714	4.519817178	2.798195206	0.010343694	0.748100537
ENSG00000164638	SLC29A4	-1.773351295	4.208936531	-2.813008298	0.009999344	0.743600518
ENSG00000169891	REPS2	1.377456619	2.658641736	2.819710674	0.009847143	0.741221825
ENSG00000183844	FAM3B	2.123741016	1.318505614	2.833296595	0.009545355	0.741221825
ENSG00000134779	TPGS2	1.181090332	6.219063146	2.802190088	0.010249735	0.748100537
ENSG00000129946	SHC2	-3.12234412	3.927180544	-2.824298971	0.009744222	0.741221825
ENSG00000143486	EIF2D	-2.007220862	6.375395688	-2.7907157	0.010521814	0.748100537
ENSG00000181374	CCL13	1.942856879	1.110346705	2.830087268	0.009615841	0.741221825
ENSG00000197217	ENTPD4	0.983656631	5.913291762	2.79421769	0.010438057	0.748100537
ENSG00000248835	AL357673.1	-1.477963343	1.785697203	-2.810370906	0.010059846	0.744570978
ENSG00000121653	MAPK8IP1	1.61653238	1.747155754	2.826682761	0.009691155	0.741221825
ENSG00000102230	PCYT1B	-1.731697991	1.974119994	-2.8263232	0.009699141	0.741221825
ENSG00000105974	CAV1	-2.241935997	4.201764789	-2.797184754	0.010367589	0.748100537
ENSG00000147421	HMBOX1	1.124531088	3.410734893	2.77996238	0.010783005	0.748100537
ENSG00000161326	DUSP14	-1.348628562	0.916653661	-2.776297188	0.010873426	0.748100537
ENSG00000154330	PGM5	-2.125918377	2.057989802	-2.7793204	0.010798791	0.748100537
ENSG00000112699	GMDS	1.14018506	6.527001269	2.787389105	0.010601967	0.748100537
ENSG00000177098	SCN4B	-1.953330049	2.106805368	-2.807367479	0.010129169	0.746182094
ENSG00000112763	BTN2A1	0.913006663	4.885828836	2.770067137	0.011028776	0.748100537
ENSG00000167468	GPX4	-0.938170066	8.396039377	-2.794145486	0.010439777	0.748100537
ENSG00000112079	STK38	0.816926033	6.370797405	2.78133051	0.010749436	0.748100537
ENSG00000128011	LRFN1	-1.282875982	1.17003181	-2.778569533	0.010817283	0.748100537
ENSG00000175879	HOXD8	-1.510165502	0.983565633	-2.764948903	0.011157973	0.748100537
ENSG00000152944	MED21	1.821404219	2.596408515	2.786846675	0.010615091	0.748100537
ENSG00000108387	SEPT4	-1.946287643	6.179266896	-2.757456629	0.011349685	0.748100537
ENSG00000006831	ADIPOR2	1.197769624	4.613525859	2.75525041	0.01140673	0.748100537
ENSG00000123552	USP45	1.421065787	3.596433318	2.76106139	0.01125706	0.748100537
ENSG00000100439	ABHD4	-1.098422613	4.819171102	-2.749649205	0.011552778	0.748100537
ENSG00000158006	PAFAH2	-1.580532614	4.586328688	-2.755876459	0.011390515	0.748100537
ENSG00000177565	TBL1XR1	1.311335984	5.624751786	2.755597636	0.011397734	0.748100537
ENSG00000125813	PAX1	-1.899930614	1.31136775	-2.752863725	0.011468746	0.748100537
ENSG00000187730	GABRD	-1.791352531	1.677902547	-2.76755446	0.011092024	0.748100537
ENSG00000150401	DCUN1D2	1.091299741	4.694957364	2.74043485	0.011796896	0.749295141
ENSG00000100031	GGT1	1.478795922	4.388036628	2.739190342	0.011830239	0.749295141
ENSG00000083844	ZNF264	-1.470413602	0.983565633	-2.741822261	0.011759829	0.749295141
ENSG00000140092	FBLN5	2.621620692	5.254437609	2.743184087	0.011723554	0.749295141
ENSG00000180776	ZDHHC20	1.920776298	3.127147117	2.754347102	0.011430164	0.748100537
ENSG00000187951	ARHGAP11B	1.543048418	3.097123168	2.750042702	0.011542461	0.748100537
ENSG00000162739	SLAMF6	1.583231466	0.823420573	2.766772491	0.011111777	0.748100537
ENSG00000198598	MMP17	-1.574548727	2.813459871	-2.781631337	0.010742068	0.748100537
ENSG00000104549	SQLE	-1.600371969	6.261893855	-2.73872278	0.011842789	0.749295141
ENSG00000125378	BMP4	-2.259151894	3.297317211	-2.771287876	0.010998171	0.748100537
ENSG00000165633	VSTM4	-1.642195573	3.5037123	-2.757826973	0.011340136	0.748100537

ENSG00000109107	ALDOC	-2.06287448	4.610973423	-2.726879573	0.012164904	0.751494141
ENSG00000186432	KPNA4	0.913761571	6.00283247	2.734756408	0.011949758	0.749444239
ENSG00000152556	PFKM	-1.28020024	6.42843253	-2.727913241	0.012136463	0.751494141
ENSG00000146386	ABRACL	1.501864093	4.210991644	2.715751248	0.012475109	0.752199834
ENSG00000112877	CEP72	1.532427641	3.061238173	2.732447547	0.012012446	0.749444239
ENSG00000167741	GGT6	2.322497395	2.455361954	2.748402629	0.011585522	0.748100537
ENSG00000060749	QSER1	1.625994475	4.036189496	2.708065943	0.012693685	0.752199834
ENSG00000181885	CLDN7	2.000533152	4.392987744	2.706069302	0.012751061	0.752199834
ENSG00000178425	NT5DC1	1.446131836	3.759736531	2.706308543	0.012744173	0.752199834
ENSG00000144821	MYH15	-1.316743236	0.931450059	-2.705319139	0.01277268	0.752199834
ENSG00000198003	CCDC151	1.529925385	1.17003181	2.731575798	0.012036196	0.749444239
ENSG00000188505	NCCRP1	1.652138121	1.627432595	2.734298852	0.011962157	0.749444239
ENSG00000141540	TTYH2	-1.643206254	4.21569502	-2.7058841	0.012756395	0.752199834
ENSG00000135144	DTX1	-1.775188417	1.845875706	-2.705627051	0.012763802	0.752199834
ENSG00000165810	BTNL9	-1.600199542	1.893073828	-2.70837339	0.012684872	0.752199834
ENSG00000159753	RLTPR	2.037243582	3.380054295	2.687726825	0.013289722	0.764133944
ENSG00000143013	LMO4	1.063002269	7.075452518	2.710952213	0.012611177	0.752199834
ENSG00000151468	CCDC3	-2.217709027	3.44684769	-2.710413651	0.012626534	0.752199834
ENSG00000109685	WHSC1	1.047740665	6.174209795	2.701083512	0.012895402	0.752199834
ENSG00000118495	PLAGL1	1.528565365	4.026104005	2.677772001	0.013590978	0.764168493
ENSG00000073614	KDM5A	0.941892138	6.783281942	2.703432666	0.012827202	0.752199834
ENSG00000091490	SEL1L3	1.573252765	5.560420746	2.690418284	0.013209361	0.764133944
ENSG00000107105	ELAVL2	1.622623488	1.016418003	2.70199994	0.012868756	0.752199834
ENSG00000170631	ZNF16	-1.368545695	3.327075205	-2.702617851	0.012850819	0.752199834
ENSG00000204140	CLPSL1	1.127070185	0.378582783	2.678378708	0.013572435	0.764168493
ENSG00000123453	SARDH	-1.970921726	2.983282174	-2.70830578	0.01268681	0.752199834
ENSG00000197380	DACT3	-1.323526641	3.734941821	-2.678273277	0.013575656	0.764168493
ENSG00000146963	C7orf55-LUC7L2	-1.427084532	2.32703976	-2.687557584	0.013294791	0.764133944
ENSG00000145632	PLK2	-1.238778573	7.160339826	-2.683545192	0.013415493	0.764168493
ENSG00000137166	FOXP4	1.272495244	7.108548453	2.690562604	0.013205064	0.764133944
ENSG00000007372	PAX6	1.877836932	3.330076287	2.661249584	0.014105233	0.764168493
ENSG00000118257	NRP2	-1.501118432	5.775493419	-2.664347377	0.01400744	0.764168493
ENSG00000167306	MYO5B	2.622080798	4.616220857	2.65788535	0.014212164	0.764168493
ENSG00000197150	ABCB8	-1.005997079	5.908939095	-2.666681874	0.013934166	0.764168493
ENSG00000144959	NCEH1	1.82185674	1.720634013	2.679180113	0.013547978	0.764168493
ENSG00000177311	ZBTB38	1.481908847	3.354681197	2.659656258	0.014155781	0.764168493
ENSG00000139597	N4BP2L1	1.290512379	3.564958665	2.658163197	0.014203304	0.764168493
ENSG00000160360	GPSM1	-1.332020353	5.744686734	-2.65535494	0.014293095	0.764168493
ENSG00000100461	RBM23	-0.7772415	7.013485379	-2.670322685	0.01382061	0.764168493
ENSG00000047365	ARAP2	1.648727456	3.476098294	2.643884212	0.014665436	0.764168493
ENSG00000102531	FNDC3A	1.432594753	5.055598142	2.650028531	0.014464871	0.764168493
ENSG00000164330	EBF1	-1.944655038	3.831912112	-2.651097204	0.014430251	0.764168493
ENSG00000133119	RFC3	1.122450924	3.753153637	2.63875158	0.014834979	0.764168493
ENSG00000111907	TPD52L1	2.294192546	3.842748566	2.639413907	0.014812998	0.764168493
ENSG00000141380	SS18	0.89908379	6.659820173	2.663851667	0.014023046	0.764168493
ENSG00000050165	DKK3	-1.901018698	5.806774137	-2.646330255	0.01458528	0.764168493
ENSG00000237441	RGL2	0.964924755	7.381627951	2.669119618	0.013858036	0.764168493
ENSG00000165084	C8orf34	-1.235607341	0.844748225	-2.633814056	0.014999814	0.764168493
ENSG00000111276	CDKN1B	1.001460997	4.731358361	2.635163173	0.014954605	0.764168493
ENSG00000171121	KCNMB3	1.437803712	1.91986381	2.659817155	0.014150669	0.764168493
ENSG00000159111	MRPL10	-0.930675764	4.892283186	-2.628808677	0.015168671	0.765310671
ENSG00000152137	HSPB8	-2.494951048	2.916722635	-2.650547066	0.014448063	0.764168493
ENSG00000167617	CDC42EP5	-1.976566119	3.771976571	-2.639966521	0.014794681	0.764168493
ENSG00000175970	UNC119B	-1.29882612	3.876456403	-2.63719797	0.01488666	0.764168493
ENSG00000112378	PERP	2.249040039	5.339152303	2.635370384	0.014947673	0.764168493
ENSG00000174502	SLC26A9	1.841033844	0.838758373	2.644215137	0.014654567	0.764168493
ENSG00000004776	HSPB6	-2.377115117	2.265555721	-2.631978937	0.015061516	0.764822814
ENSG00000158856	DMTN	1.271795539	4.548096886	2.620037798	0.015468871	0.766758365
ENSG00000112697	TMEM30A	1.185155352	6.054601087	2.639985027	0.014794068	0.764168493
ENSG00000135424	ITGA7	-1.360375348	5.526301903	-2.620648855	0.015447777	0.766758365
ENSG00000091831	ESR1	-1.34633352	0.895326009	-2.616463785	0.015592791	0.766758365
ENSG00000125611	CHCHD5	-1.458043228	3.216549663	-2.639958186	0.014794957	0.764168493
ENSG00000100234	TIMP3	-1.672825289	6.871862201	-2.633810623	0.01499993	0.764168493

ENSG00000158805	ZNF276	-1.317746204	5.048603668	-2.610735096	0.015793364	0.766758365
ENSG00000121858	TNFSF10	1.52489283	5.679037361	2.629612755	0.015141426	0.765310671
ENSG00000243943	ZNF512	-1.415814617	1.239703843	-2.613695905	0.015689399	0.766758365
ENSG00000214253	FIS1	-0.920948671	7.128201761	-2.637911872	0.014862891	0.764168493
ENSG00000138600	SPPL2A	1.084021009	4.762826829	2.613392889	0.01570001	0.766758365
ENSG00000126351	THRA	-1.124204202	5.319768412	-2.608897064	0.015858228	0.766758365
ENSG00000205664	RP11-706O15.1	1.482200928	3.217697298	2.606934858	0.015927751	0.766758365
ENSG00000111664	GNB3	1.878077242	2.271384653	2.620888521	0.015439511	0.766758365
ENSG00000185104	FAF1	0.78440043	7.013586226	2.635068441	0.014957775	0.764168493
ENSG00000082014	SMARCD3	-1.432575206	6.155299094	-2.612369789	0.015735884	0.766758365
ENSG00000133636	NTS	1.335638176	0.543721308	2.606861467	0.015930357	0.766758365
ENSG00000099622	CIRBP	-0.840337858	7.969353458	-2.634550277	0.014975128	0.764168493
ENSG00000172819	RARG	-0.926956508	6.200926629	-2.607910273	0.015893155	0.766758365
ENSG00000165816	VWA2	1.726278189	1.046161535	2.610846889	0.015789427	0.766758365
ENSG00000184343	SRPK3	1.919729815	3.067231586	2.591024372	0.016502121	0.77639638
ENSG00000114315	HES1	1.064367649	5.381222788	2.602820042	0.016074472	0.771328847
ENSG00000085382	HACE1	0.990151791	4.783552423	2.591895458	0.016470179	0.77639638
ENSG00000152705	CATSPER3	1.396462128	0.567282363	2.590361279	0.016526475	0.77639638
ENSG00000163406	SLC15A2	1.44247161	3.003431762	2.592851282	0.016435196	0.77639638
ENSG00000141542	RAB40B	1.303347781	4.331971158	2.580774188	0.016882374	0.77639638
ENSG00000213853	EMP2	1.075998692	6.906304505	2.615696945	0.0156195	0.766758365
ENSG00000141446	ESCO1	1.164595086	3.89931734	2.578785598	0.016957089	0.77639638
ENSG00000119138	KLF9	-1.67364579	1.889423183	-2.582962513	0.01680051	0.77639638
ENSG00000177675	CD163L1	1.875556968	4.181476848	2.578379088	0.0169724	0.77639638
ENSG00000198840	MT-ND3	-1.347538266	6.022678446	-2.58290345	0.016802715	0.77639638
ENSG00000139220	PPFIA2	-1.376397948	1.277441845	-2.574127842	0.017133303	0.77639638
ENSG00000124523	SIRT5	0.788733045	4.969134045	2.573628385	0.0171523	0.77639638
ENSG00000167157	PRRX2	-1.641740586	3.37419401	-2.582093683	0.016832968	0.77639638
ENSG00000185090	MANEAL	1.54810962	1.277441845	2.579641401	0.016924897	0.77639638
ENSG00000117899	MESDC2	1.002093571	4.984549998	2.570438949	0.017274077	0.77639638
ENSG00000131480	AOC2	-1.839505424	1.60458267	-2.56414991	0.017516576	0.77639638
ENSG00000161940	BCL6B	-1.377007391	3.309937571	-2.575418474	0.017084304	0.77639638
ENSG00000110680	CALCA	1.897827639	0.739370967	2.568562918	0.017346084	0.77639638
ENSG00000198712	MT-CO2	-0.908583325	7.563366577	-2.592380464	0.016452419	0.77639638
ENSG00000132561	MATN2	-1.343778457	6.948375119	-2.583602423	0.016776642	0.77639638
ENSG00000161057	PSMC2	-0.90304275	5.740662341	-2.569197391	0.0173217	0.77639638
ENSG00000164136	IL15	1.48948516	2.903810754	2.560313326	0.017666071	0.778646956
ENSG00000175387	SMAD2	0.930494785	6.214781966	2.583606887	0.016776476	0.77639638
ENSG00000198435	NRARP	-1.138928053	0.794866054	-2.55126602	0.018023341	0.784798375
ENSG00000189057	FAM111B	1.474672428	0.998362031	2.565388309	0.017468574	0.77639638
ENSG00000119121	TRPM6	-1.935590863	2.103058306	-2.555138342	0.017869607	0.784798375
ENSG00000088320	REM1	-1.309134101	0.875954973	-2.549536821	0.018092389	0.784798375
ENSG00000085563	ABCB1	-1.847122711	2.176316587	-2.572608629	0.017191148	0.77639638
ENSG00000171097	CCBL1	-1.526868631	3.966975402	-2.551914366	0.017997515	0.784798375
ENSG00000229676	ZNF492	1.335879126	1.547957627	2.566648035	0.017419872	0.77639638
ENSG00000138587	MNS1	1.723229529	2.92810626	2.549221134	0.018105021	0.784798375
ENSG00000179218	CALR	0.965248742	10.66644126	2.607395465	0.015911406	0.766758365
ENSG00000116157	GPX7	1.445521857	3.45123765	2.542894812	0.018359915	0.785807558
ENSG00000178750	STX19	1.150793147	0.363786385	2.543232909	0.018346209	0.785807558
ENSG00000159348	CYB5R1	-0.871246461	6.883187984	-2.572125665	0.017209576	0.77639638
ENSG00000149418	ST14	1.899631784	7.448266811	2.583078239	0.016796192	0.77639638
ENSG00000078142	PIK3C3	1.010994766	5.860859846	2.56289024	0.017565529	0.77639638
ENSG00000119042	SATB2	-1.775068964	3.49381298	-2.54357421	0.018332382	0.785807558
ENSG00000180104	EXOC3	0.742777609	6.303679852	2.565690482	0.017456881	0.77639638
ENSG00000165868	HSPA12A	-2.247470515	2.764910765	-2.541958404	0.018397928	0.785807558
ENSG00000214919	AC104472.1	1.095894259	0.614931131	2.533307724	0.018752583	0.785807558
ENSG00000164465	DCBLD1	1.414440388	4.005096056	2.531411827	0.018831158	0.785807558
ENSG00000101197	BIRC7	-1.441561189	0.975719315	-2.52674018	0.019026086	0.785807558
ENSG00000144034	TPRKB	0.871250878	4.940011097	2.535770817	0.018650958	0.785807558
ENSG00000165804	ZNF219	-1.283341748	3.772930108	-2.523164089	0.019176571	0.785807558
ENSG00000111639	MRPL51	0.976076097	6.034978438	2.54920343	0.01810573	0.784798375
ENSG00000135414	GDF11	-1.818691707	2.361502094	-2.53951104	0.018497623	0.785807558
ENSG00000145919	BOD1	-0.920799603	4.937166292	-2.519344293	0.019338537	0.785807558

ENSG00000023445	BIRC3	1.806275228	4.319301931	2.518644665	0.01936834	0.785807558
ENSG00000148600	CDHR1	-1.832062762	2.106480722	-2.51509842	0.019520065	0.785807558
ENSG00000196684	HSH2D	1.634169584	2.33166029	2.527299615	0.019002644	0.785807558
ENSG00000165140	FBP1	1.465383738	2.930210158	2.510142388	0.019733964	0.785807558
ENSG00000158887	MPZ	-1.409086566	0.936714656	-2.509224552	0.019773817	0.785807558
ENSG00000134758	RNF138	1.543755033	2.613960237	2.516861439	0.019444497	0.785807558
ENSG00000112941	PAPD7	0.989150053	5.260745865	2.525925661	0.019060264	0.785807558
ENSG00000162552	WNT4	-1.64406483	1.741385822	-2.511299436	0.019683832	0.785807558
ENSG00000006377	DLX6	-1.346452477	0.873302744	-2.503233981	0.020035772	0.785807558
ENSG00000206384	COL6A6	-1.840468277	1.220483543	-2.502697032	0.020059409	0.785807558
ENSG00000114948	ADAM23	-2.78951036	2.690592962	-2.521133987	0.019262492	0.785807558
ENSG00000165389	SPTSSA	-1.275114213	4.687227946	-2.501372319	0.020117835	0.785807558
ENSG00000184005	ST6GALNAC3	-1.188891352	0.829951827	-2.500269317	0.020166603	0.785807558
ENSG00000161904	LEMD2	0.727321596	6.085256159	2.529908077	0.018893699	0.785807558
ENSG00000131143	COX4I1	-0.758820782	7.097786332	-2.535586726	0.018658536	0.785807558
ENSG00000111726	CMAS	0.96564972	6.328269485	2.529367329	0.018916236	0.785807558
ENSG00000197993	KEL	-1.590898875	1.429481191	-2.490459894	0.020605185	0.785807558
ENSG00000127528	KLF2	-1.580356609	3.272744877	-2.500186926	0.02017025	0.785807558
ENSG00000145247	OCIAD2	1.259631293	5.627088811	2.514099013	0.019563024	0.785807558
ENSG00000179886	TIGD5	-1.150384787	0.780069656	-2.485257356	0.020841377	0.785807558
ENSG00000129355	CDKN2D	1.367502383	2.95592248	2.487129828	0.020756079	0.785807558
ENSG00000197442	MAP3K5	1.187090152	5.399667648	2.509561843	0.019759163	0.785807558
ENSG00000101850	GPR143	1.532616213	2.520655346	2.494819555	0.020409179	0.785807558
ENSG00000182676	PPP1R27	-1.060362319	0.708164219	-2.482524692	0.020966445	0.785807558
ENSG00000183741	CBX6	-1.376907778	5.064122956	-2.481998653	0.0209906	0.785807558
ENSG00000055732	MCOLN3	1.998477141	3.155230895	2.478614465	0.021146621	0.785807558
ENSG00000112280	COL9A1	-3.923074341	3.672248479	-2.47882681	0.0211368	0.785807558
ENSG00000135315	KIAA1009	1.133230232	4.018982614	2.475442325	0.021293844	0.785807558
ENSG00000171365	CLCN5	1.206955685	3.002051343	2.476555245	0.021242084	0.785807558
ENSG00000081138	CDH7	1.316246239	0.575670857	2.475883412	0.021273315	0.785807558
ENSG00000128422	KRT17	2.042094674	2.277721496	2.476154181	0.021260723	0.785807558
ENSG00000143127	ITGA10	-2.523447565	5.666690839	-2.47427794	0.021348123	0.785807558
ENSG00000152558	TMEM123	1.233132885	6.603388799	2.51279393	0.019619255	0.785807558
ENSG00000181577	C6orf223	1.00892862	0.335231866	2.466464217	0.021715706	0.785807558
ENSG00000128394	APOBEC3F	-1.42242892	2.461553663	-2.478839145	0.021136229	0.785807558
ENSG00000196591	HDAC2	0.837101231	8.159345453	2.525507051	0.019077852	0.785807558
ENSG00000160796	NBEAL2	1.222417616	6.875593192	2.513054009	0.019608038	0.785807558
ENSG00000024048	UBR2	0.872327613	6.727338089	2.510586631	0.019714702	0.785807558
ENSG00000262246	CORO7	-1.503226992	1.886391989	-2.471608245	0.021473059	0.785807558
ENSG00000166886	NAB2	-0.78362094	5.467541553	-2.481472865	0.02101477	0.785807558
ENSG00000162174	ASRGL1	-1.544626701	2.644823404	-2.475437762	0.021294056	0.785807558
ENSG00000184436	THAP7	-1.231876406	4.641341089	-2.463321775	0.021865188	0.785807558
ENSG00000120647	CCDC77	1.351914516	3.434696716	2.462096522	0.02192373	0.785807558
ENSG00000166562	SEC11C	1.042792936	5.4705063	2.489378082	0.020654092	0.785807558
ENSG00000139835	GRTP1	1.460136396	3.295860275	2.462466932	0.021906016	0.785807558
ENSG00000171757	LRRC34	1.54962658	1.501470486	2.471398376	0.021482909	0.785807558
ENSG00000188322	SBK1	1.87741709	2.412234839	2.469574213	0.021568703	0.785807558
ENSG00000113205	PCDHB3	1.025149367	0.335231866	2.455351646	0.02224861	0.785807558
ENSG00000198176	TFDP1	1.161124181	5.255290359	2.478383271	0.021157319	0.785807558
ENSG00000137561	TTPA	1.217209324	0.931450059	2.458190418	0.022111334	0.785807558
ENSG00000068697	LAPTM4A	-0.67857645	9.303673296	-2.518531748	0.019373154	0.785807558
ENSG00000162998	FRZB	-3.216556716	4.139549239	-2.457037982	0.022166968	0.785807558
ENSG00000167088	SNRPD1	0.946954839	4.977732783	2.469741376	0.021560828	0.785807558
ENSG00000133657	ATP13A3	1.088843119	5.444495109	2.475644579	0.021284428	0.785807558
ENSG00000187773	FAM69C	-1.956365163	2.036062476	-2.454689704	0.022280733	0.785807558
ENSG00000118418	HMGN3	0.92337005	6.585484142	2.492630526	0.020507377	0.785807558
ENSG00000132746	ALDH3B2	2.167524713	2.736604429	2.453586568	0.022334362	0.785807558
ENSG00000160049	DFFA	-0.901120064	6.31395706	-2.475828092	0.021275889	0.785807558
ENSG00000128602	SMO	-1.172732522	6.289000693	-2.474207361	0.021351417	0.785807558
ENSG00000062524	LTK	1.724836462	1.570492511	2.451436825	0.022439218	0.785807558
ENSG00000129282	MRM1	-1.392568926	1.877007881	-2.449533151	0.022532452	0.785807558
ENSG00000128606	LRRC17	-2.09839505	2.82763352	-2.455289533	0.022251622	0.785807558
ENSG00000162777	DENND2D	1.248835493	4.460744593	2.446250911	0.022694046	0.785807558

ENSG00000181444	ZNF467	-1.618358574	3.015903255	-2.448833336	0.022566816	0.785807558
ENSG00000101670	LIPG	1.608707595	1.278061296	2.445773083	0.022717661	0.785807558
ENSG00000087301	TXNDC16	1.430907145	3.408652076	2.430889086	0.023464734	0.798161458
ENSG00000244731	C4A	1.944127616	3.035889591	2.429116049	0.023555229	0.798161458
ENSG00000132321	IQCA1	-2.09089888	4.040972715	-2.431268681	0.023445401	0.798161458
ENSG00000143515	ATP8B2	-1.299368924	7.227905431	-2.476991088	0.021221845	0.785807558
ENSG00000172915	NBEA	1.437501088	2.560351258	2.429011642	0.023560568	0.798161458
ENSG00000164961	KIAA0196	-0.885870594	7.260316036	-2.475209491	0.021304687	0.785807558
ENSG00000146143	PRIM2	0.952210714	5.073036673	2.448731472	0.022571822	0.785807558
ENSG00000198938	MT-CO3	-1.008159321	7.421435888	-2.475649297	0.021284209	0.785807558
ENSG00000184900	SUMO3	-0.861606706	6.629272681	-2.467297177	0.021676243	0.785807558
ENSG00000174243	DDX23	-0.797856223	6.462392569	-2.465471557	0.021762823	0.785807558
ENSG00000006453	BAIAP2L1	1.607515041	5.942210532	2.463021015	0.021879544	0.785807558
ENSG00000185896	LAMP1	0.768053434	8.830618487	2.493008219	0.020490403	0.785807558
ENSG00000198910	L1CAM	1.777492368	2.907032378	2.423045934	0.023867498	0.798161458
ENSG00000102048	ASB9	1.6509301	1.174122453	2.430914434	0.023463442	0.798161458
ENSG00000184271	POU6F1	-1.435194603	4.089119173	-2.422467359	0.023897461	0.798161458
ENSG00000141150	RASL10B	-1.493909142	2.05620733	-2.428825118	0.023570109	0.798161458
ENSG00000167767	KRT80	2.127296454	2.312779514	2.425170063	0.023757792	0.798161458
ENSG00000169255	B3GALNT1	1.173856123	3.61598119	2.418240019	0.024117445	0.801751761
ENSG00000073803	MAP3K13	0.894530214	5.656806735	2.449119154	0.022552775	0.785807558
ENSG00000053747	LAMA3	1.64349767	5.716651305	2.452696153	0.022377738	0.785807558
ENSG00000179456	ZBTB18	-1.191509573	0.844748225	-2.414848982	0.024295261	0.803529127
ENSG00000149090	PAMR1	-2.087645733	3.045545502	-2.423005639	0.023869584	0.798161458
ENSG00000149927	DOC2A	-1.8672648	2.969759694	-2.417093409	0.024177435	0.80204679
ENSG00000124243	BCAS4	-1.743476156	2.945731078	-2.419558342	0.024048642	0.801161858
ENSG00000167658	EEF2	-0.750488686	11.43787005	-2.493887488	0.020450937	0.785807558
ENSG00000169085	C8orf46	-2.100086904	1.853637576	-2.410225634	0.024539651	0.803529127
ENSG00000068831	RASGRP2	-1.201056536	3.459005915	-2.411787946	0.024456814	0.803529127
ENSG00000188859	FAM78B	1.276734535	0.83064744	2.410637322	0.024517797	0.803529127
ENSG00000158125	XDH	2.242990796	2.462317416	2.411746592	0.024459003	0.803529127
ENSG00000185760	KCNQ5	1.425078182	1.141477291	2.409455378	0.024580586	0.803529127
ENSG00000137185	ZSCAN9	1.36151228	2.343173322	2.410051051	0.024548923	0.803529127
ENSG00000130177	CDC16	0.847876204	7.28891103	2.460036931	0.022022464	0.785807558
ENSG00000197183	C20orf112	-1.036286499	4.773634359	-2.40058576	0.025056538	0.812318463
ENSG00000228049	POLR2J2	-1.360875633	1.730125953	-2.39877821	0.025154571	0.813815205
ENSG00000132182	NUP210	1.504133817	6.339512807	2.44467004	0.02277226	0.785807558
ENSG00000177469	PTRF	-1.251473815	7.622116968	-2.448767145	0.022570069	0.785807558
ENSG00000157800	SLC37A3	-0.791754993	5.916473345	-2.422269609	0.023907711	0.798161458
ENSG00000047457	CP	2.995604661	6.182966063	2.434279564	0.023292581	0.798161458
ENSG00000197457	STMN3	-1.304197899	4.434261819	-2.389559709	0.025660073	0.814092845
ENSG00000064102	ASUN	0.964854701	5.567674181	2.424082542	0.023813901	0.798161458
ENSG00000175315	CST6	1.325833419	0.751515136	2.38656997	0.02582602	0.814092845
ENSG00000089693	MLF2	0.741557395	8.297785071	2.45568769	0.022232318	0.785807558
ENSG00000086205	FOLH1	1.364777326	4.367888771	2.394162898	0.025406494	0.814092845
ENSG00000141425	RPRD1A	1.08597203	4.953527058	2.4053022	0.0248024	0.807415876
ENSG00000078237	C12orf5	1.507017848	2.821727133	2.384254026	0.025955248	0.814092845
ENSG00000004700	RECQL	1.077173037	5.818921676	2.425189847	0.023756772	0.798161458
ENSG00000096433	ITPR3	1.591749137	7.381412952	2.444383447	0.022786466	0.785807558
ENSG00000165410	CFL2	-1.48999327	4.02417461	-2.382424572	0.026057751	0.814092845
ENSG00000213888	AC005003.1	-1.438925115	3.071661517	-2.39086958	0.025587678	0.814092845
ENSG00000254521	SIGLEC12	1.389403958	1.183290419	2.388008161	0.025746069	0.814092845
ENSG00000158008	EXTL1	-3.443081827	3.939506608	-2.381551738	0.026106787	0.814092845
ENSG00000182851	GPIHBP1	-1.141879936	0.775076191	-2.383328671	0.026007048	0.814092845
ENSG00000121064	SCPEP1	1.145261592	7.6474783	2.447585464	0.022628213	0.785807558
ENSG00000140254	DUOXA1	1.609308518	2.239537282	2.38477207	0.02592629	0.814092845
ENSG00000013588	GPRC5A	1.85814986	4.647855698	2.390527072	0.02560659	0.814092845
ENSG00000198763	MT-ND2	-0.874402719	7.571053359	-2.435829837	0.023214255	0.798161458
ENSG00000135378	PRRG4	1.313699412	3.980078593	2.37721005	0.026351971	0.814920926
ENSG00000196409	ZNF658	-1.092611482	0.751515136	-2.375635595	0.026441407	0.814920926
ENSG00000124370	MCEE	-1.405633419	3.678433023	-2.37483244	0.026487137	0.814920926
ENSG00000173210	ABLIM3	-1.785765344	2.9213408	-2.380850984	0.026146217	0.814092845
ENSG00000178295	GEN1	1.16628803	2.872224018	2.375082136	0.026472912	0.814920926

ENSG00000136999	NOV	1.633815562	2.800323318	2.370710551	0.026722981	0.817368988
ENSG00000111199	TRPV4	-2.008004264	5.849544482	-2.380802886	0.026148926	0.814092845
ENSG00000185551	NR2F2	-1.416046232	5.044523007	-2.376576536	0.026387924	0.814920926
ENSG00000154914	USP43	1.706932403	1.901188387	2.374926923	0.026481754	0.814920926
ENSG00000112305	SMAP1	0.883796417	4.743357666	2.385071038	0.025909591	0.814092845
ENSG00000163171	CDC42EP3	-1.380174098	2.884220404	-2.370982259	0.026707375	0.817368988
ENSG00000151778	SERP2	-1.279435538	1.213382727	-2.362978867	0.027170593	0.823038182
ENSG00000121644	DESI2	-1.296068574	2.811376645	-2.367973548	0.026880652	0.818998665
ENSG00000177685	EFCAB4A	1.82779747	4.43246079	2.366913175	0.026941967	0.819275988
ENSG00000107485	GATA3	1.940529279	3.2046481	2.353016636	0.027757519	0.826898049
ENSG00000146411	SLC2A12	1.816951111	1.726321502	2.354413583	0.02767452	0.826898049
ENSG00000119812	FAM98A	-0.802316532	5.83509585	-2.383421987	0.02600182	0.814092845
ENSG00000129932	DOHH	-1.097838464	4.148921818	-2.346360296	0.028156155	0.829563404
ENSG00000013583	HEBP1	0.921984372	5.396238348	2.381179028	0.026127752	0.814092845
ENSG00000184156	KCNQ3	-1.454975564	1.969745981	-2.340690055	0.028499881	0.829563404
ENSG00000117643	MAN1C1	-1.691619891	3.46396291	-2.340639547	0.02850296	0.829563404
ENSG00000113721	PDGFRB	-1.266273095	7.521583399	-2.405943788	0.024768013	0.807415876
ENSG00000153130	SCOC	1.319402352	3.495237077	2.34106909	0.028476784	0.829563404
ENSG00000198168	SVIP	1.242246375	3.710328783	2.34196604	0.028422197	0.829563404
ENSG00000178568	ERBB4	-1.10908978	0.758742004	-2.341116306	0.028473908	0.829563404
ENSG00000047648	ARHGAP6	-1.549549175	2.140748203	-2.345678847	0.028197261	0.829563404
ENSG00000144645	OSBPL10	1.302996001	4.339806095	2.352489454	0.0277889	0.826898049
ENSG00000113716	HMGXB3	-0.728740663	6.470050616	-2.386470293	0.02583157	0.814092845
ENSG00000118503	TNFAIP3	1.287885486	4.093874612	2.345773477	0.02819155	0.829563404
ENSG00000155158	TTC39B	0.838119586	4.854714992	2.356398554	0.027556977	0.826898049
ENSG00000101161	PRPF6	-0.681272297	7.996126898	-2.402858571	0.024933772	0.810012045
ENSG00000126746	ZNF384	0.86333917	5.661697129	2.372251557	0.026634583	0.817368988
ENSG00000146242	TPBG	1.436799332	1.525934362	2.332630015	0.028995115	0.829563404
ENSG00000107331	ABCA2	-1.139307378	7.333905645	-2.387841093	0.025755345	0.814092845
ENSG00000106278	PTPRZ1	-2.644566011	3.892763457	-2.328240815	0.029268113	0.829563404
ENSG00000233701	PRR23C	-1.024123276	0.701632965	-2.33071068	0.029114204	0.829563404
ENSG00000186868	MAPT	-1.565564466	1.990454181	-2.325680213	0.029428465	0.829563404
ENSG00000131477	RAMP2	-1.3485161	2.448625153	-2.330137426	0.02914986	0.829563404
ENSG00000105472	CLEC11A	-2.282413192	5.230747121	-2.347703669	0.028075281	0.829563404
ENSG00000160691	SHC1	-0.981193053	7.989450409	-2.396882385	0.025257772	0.814092845
ENSG00000115919	KYNU	2.132110106	3.380205196	2.328649749	0.029242579	0.829563404
ENSG00000188130	MAPK12	-1.315020057	5.438390946	-2.34414539	0.028289965	0.829563404
ENSG00000111731	C2CD5	1.16866198	5.529695715	2.364430583	0.027086026	0.822063497
ENSG00000121068	TBX2	-1.805652707	5.027847732	-2.340661206	0.028501639	0.829563404
ENSG00000163319	MRPS18C	1.50510239	2.328376924	2.316804521	0.029990547	0.829563404
ENSG00000134326	CMPK2	1.64365952	3.445383866	2.316286031	0.030023684	0.829563404
ENSG00000174844	DNAH12	-1.277568453	0.923880529	-2.318897468	0.029857124	0.829563404
ENSG00000122679	RAMP3	1.651234664	1.941902592	2.316148121	0.030032504	0.829563404
ENSG00000171703	TCEA2	-0.943705433	5.902836897	-2.352979654	0.027759719	0.826898049
ENSG00000115267	IFIH1	1.238932527	5.624628345	2.359451159	0.027377109	0.826898049
ENSG00000177613	CSTF2T	-1.296309179	1.146470755	-2.312570564	0.03026213	0.829563404
ENSG00000139734	DIAPH3	1.206668366	4.428814693	2.332554743	0.028999777	0.829563404
ENSG00000163735	CXCL5	1.439375212	0.94860321	2.312824989	0.030245747	0.829563404
ENSG00000164778	EN2	-1.074568411	0.736718738	-2.314012346	0.030169396	0.829563404
ENSG00000163435	ELF3	2.628672071	4.495554919	2.321125066	0.029715715	0.829563404
ENSG00000100170	SLC5A1	2.227497025	1.987728358	2.312329441	0.030277664	0.829563404
ENSG00000111752	PHC1	1.278705656	2.577999397	2.305782406	0.030702259	0.83357425
ENSG00000112210	RAB23	1.138127967	4.510619572	2.323335496	0.029576005	0.829563404
ENSG00000154229	PRKCA	-1.522792467	5.075786432	-2.312079284	0.030293788	0.829563404
ENSG00000198563	DDX39B	1.410544769	5.907834875	2.352704053	0.027776122	0.826898049
ENSG00000179361	ARID3B	-1.33082715	1.569980893	-2.298983667	0.031148946	0.834446934
ENSG00000129244	ATP1B2	-2.093075003	3.007677067	-2.300684237	0.031036661	0.834446934
ENSG00000166396	SERPINB7	1.375223094	0.708164219	2.298306959	0.031193731	0.834446934
ENSG00000227184	EPPK1	2.043946362	2.927863321	2.297432465	0.031251692	0.834446934
ENSG00000165392	WRN	1.012151413	4.990753029	2.327309933	0.029326315	0.829563404
ENSG00000164683	HEY1	-2.089145528	3.579274374	-2.29547951	0.031381491	0.834446934
ENSG00000064393	HIPK2	-1.119581681	5.007246073	-2.309471924	0.030462316	0.832725097
ENSG00000143740	SNAP47	-1.384206461	4.224486818	-2.294013166	0.031479272	0.834446934

ENSG00000140519	RHCG	1.431770284	1.090975669	2.293964189	0.031482543	0.834446934
ENSG00000162496	DHRS3	-1.272346259	6.171714459	-2.337520426	0.028693698	0.829563404
ENSG00000063180	CA11	-2.239690682	3.904555776	-2.291173054	0.031669455	0.83610408
ENSG00000012660	ELOVL5	0.800401093	6.318710125	2.353323087	0.027739291	0.826898049
ENSG00000173889	PHC3	1.090376308	5.538609427	2.333162086	0.028962181	0.829563404
ENSG00000101463	SYNDIG1	-1.135759908	1.103119838	-2.291168077	0.03166979	0.83610408
ENSG00000130147	SH3BP4	-1.034296218	3.867739187	-2.289001911	0.03181555	0.83610408
ENSG00000072134	EPN2	-1.040920324	4.255686152	-2.288559824	0.031845374	0.83610408
ENSG00000138772	ANXA3	2.532523055	4.320250524	2.304340237	0.030796518	0.834446934
ENSG00000165434	PGM2L1	1.475767152	3.311226167	2.287310499	0.031929792	0.83610408
ENSG00000204580	DDR1	1.531362988	8.139461942	2.368426172	0.026854519	0.818998665
ENSG00000160310	PRMT2	-0.817575523	7.349792468	-2.351883588	0.027825006	0.826898049
ENSG00000170190	SLC16A5	1.06404963	3.344571937	2.282313636	0.032269485	0.83610408
ENSG00000119888	EPCAM	2.70298154	5.605892708	2.32617045	0.029397703	0.829563404
ENSG00000088035	ALG6	0.910445312	4.737654973	2.306806044	0.030635515	0.83357425
ENSG00000122417	ODF2L	1.006394393	5.758943217	2.333650953	0.028931951	0.829563404
ENSG00000188785	ZNF548	-1.343911128	2.758824904	-2.282214095	0.032276285	0.83610408
ENSG00000205922	ONECUT3	-1.068008023	1.031214401	-2.281997846	0.032291063	0.83610408
ENSG00000090432	MUL1	-1.007578773	4.020675936	-2.278389513	0.032538565	0.836435787
ENSG00000116035	VAX2	-1.429037515	1.521636511	-2.278146301	0.032555309	0.836435787
ENSG00000144228	SPOPL	1.099419316	2.855588241	2.277238151	0.032617902	0.836435787
ENSG00000137727	ARHGAP20	-1.135306297	0.768544937	-2.282626089	0.032248147	0.83610408
ENSG00000105722	ERF	-0.958963708	5.111751359	-2.296549858	0.031310292	0.834446934
ENSG00000182307	C8orf33	-1.242895764	5.298591665	-2.29757318	0.031242359	0.834446934
ENSG00000144908	ALDH1L1	-1.973417781	2.930771166	-2.273987199	0.032842874	0.836435787
ENSG00000160791	CCR5	1.189527239	0.509553873	2.272953288	0.032914719	0.836435787
ENSG00000170175	CHRNB1	1.009559953	4.098762482	2.286239659	0.032002312	0.83610408
ENSG00000173705	SUSD5	-1.763278569	2.235447962	-2.270652402	0.03307512	0.836435787
ENSG00000211455	STK38L	1.375319673	5.7863799	2.324206159	0.02952114	0.829563404
ENSG00000167778	SPRYD3	-1.006952739	6.161187885	-2.319908438	0.029792871	0.829563404
ENSG00000083817	ZNF416	1.393700586	1.189821672	2.269896716	0.033127956	0.836435787
ENSG00000165730	STOX1	1.388459766	0.826072802	2.268255688	0.03324296	0.836435787
ENSG00000165689	SDCCAG3	-0.942228066	3.98735822	-2.267974439	0.033262707	0.836435787
ENSG00000111077	TENC1	-1.062378823	7.353140967	-2.338401652	0.028639691	0.829563404
ENSG00000125827	TMX4	1.195816411	5.918896242	2.324782622	0.029484865	0.829563404
ENSG00000186496	ZNF396	1.394266734	1.795623486	2.267853338	0.033271212	0.836435787
ENSG00000117472	TSPAN1	1.480799839	0.942071956	2.264668125	0.033495648	0.836435787
ENSG00000021355	SERPINB1	0.959831898	5.678455945	2.319474962	0.029820405	0.829563404
ENSG00000160447	PKN3	1.130711995	4.555607824	2.289425297	0.031787013	0.83610408
ENSG00000136026	CKAP4	-1.150640916	5.349115223	-2.296193933	0.031333951	0.834446934
ENSG00000118640	VAMP8	1.361893607	5.140762513	2.302288774	0.030931057	0.834446934
ENSG00000181896	ZNF101	1.016092266	4.549688272	2.282320344	0.032269026	0.83610408
ENSG00000176731	C8orf59	-1.267437163	4.424083818	-2.261169124	0.033743786	0.840434524
ENSG00000124279	FASTKD3	1.220931876	2.951162565	2.253083288	0.034323638	0.841011466
ENSG00000134539	KLRD1	1.410774989	1.579164375	2.255224051	0.034169241	0.841011466
ENSG00000136169	SETDB2	1.089385114	3.779134483	2.258729877	0.033917761	0.841011466
ENSG00000112379	KIAA1244	1.799167299	3.074804325	2.252216932	0.034386302	0.841011466
ENSG00000254995	STX16-NPEPL1	0.922654744	0.428464954	2.255113765	0.03417718	0.841011466
ENSG00000132849	INADL	1.18449625	6.480095313	2.319512259	0.029818035	0.829563404
ENSG00000132017	DCAF15	0.719436342	5.924173759	2.312194978	0.03028633	0.829563404
ENSG00000186407	CD300E	1.528419646	1.125143103	2.251185178	0.034461065	0.841011466
ENSG00000164078	MST1R	2.222691478	3.961209031	2.255120978	0.03417666	0.841011466
ENSG00000086475	SEPHS1	1.040411042	3.540841047	2.252622535	0.034356951	0.841011466
ENSG00000169884	WNT10B	-1.576569676	1.096588584	-2.248625578	0.03464718	0.84103712
ENSG00000185163	DDX51	-0.867484191	5.505512209	-2.283583879	0.03218282	0.83610408
ENSG00000065457	ADAT1	-0.7971968	5.020898498	-2.268301523	0.033239743	0.836435787
ENSG00000111261	MANSC1	1.785531531	4.130637269	2.254352682	0.03423201	0.841011466
ENSG00000184787	UBE2G2	-0.755468245	7.289390572	-2.319843816	0.029796974	0.829563404
ENSG00000005893	LAMP2	0.968412096	7.879830385	2.33366499	0.028931083	0.829563404
ENSG00000179632	MAF1	-0.960788859	7.328800528	-2.318968758	0.029852589	0.829563404
ENSG00000145882	PCYOX1L	-1.245139553	2.675683775	-2.244023452	0.034984119	0.844357795
ENSG00000104998	IL27RA	1.211983803	4.626334573	2.274308279	0.032820592	0.836435787
ENSG00000109927	TECTA	1.399087624	1.254500241	2.24088461	0.035215635	0.844357795

ENSG00000184857	TMEM186	1.295250881	1.591308545	2.241737425	0.035152595	0.844357795
ENSG00000139197	PEX5	0.866799577	5.799635067	2.298125058	0.031205779	0.834446934
ENSG00000113140	SPARC	-1.495544647	11.58055022	-2.352074686	0.027813613	0.826898049
ENSG00000235173	FAM203A	-1.407640447	2.280233168	-2.239936571	0.035285835	0.844357795
ENSG00000219438	FAM19A5	-1.674829749	2.006657324	-2.23842418	0.035398087	0.844357795
ENSG00000187288	CIDEC	-1.851047232	1.57989648	-2.242007102	0.035132682	0.844357795
ENSG00000006530	AGK	-1.351962786	4.047135395	-2.237154855	0.035492549	0.844357795
ENSG00000111450	STX2	-0.867202884	5.221176376	-2.266900406	0.033338213	0.836435787
ENSG00000186714	CCDC73	0.885929254	0.291880949	2.240029336	0.03527896	0.844357795
ENSG00000099999	RNF215	-0.80068385	4.905861347	-2.255314993	0.034162696	0.841011466
ENSG00000241322	CDRT1	0.924131714	0.571580214	2.236874297	0.035513459	0.844357795
ENSG00000171847	FAM90A1	1.423251675	1.035305044	2.233405952	0.035772882	0.84766165
ENSG00000110536	PTPMT1	0.86136811	0.428464954	2.236844842	0.035515655	0.844357795
ENSG00000125841	NRSN2	-1.090410183	3.842188089	-2.229633722	0.036056991	0.84766165
ENSG00000111361	EIF2B1	-0.665042665	5.906518485	-2.282649262	0.032246565	0.83610408
ENSG00000124795	DEK	0.835334607	7.956788056	2.320683466	0.029743699	0.829563404
ENSG00000106733	NMRK1	-0.86820293	5.128498755	-2.253662294	0.034281816	0.841011466
ENSG00000108384	RAD51C	-1.243723515	3.93006758	-2.226362212	0.036305049	0.84766165
ENSG00000183709	IFNL2	0.858425188	0.291880949	2.230554456	0.035987456	0.84766165
ENSG00000242259	C22orf39	-1.01264192	3.50587199	-2.223396462	0.036531263	0.84766165
ENSG00000172403	SYNPO2	-1.490666784	1.320792762	-2.227616823	0.036209737	0.84766165
ENSG00000065357	DGKA	-1.154688997	5.987909293	-2.267953499	0.033264177	0.836435787
ENSG00000197930	ERO1L	1.375234699	6.195721678	2.296444958	0.031317263	0.834446934
ENSG00000188522	FAM83G	1.194627195	3.041977042	2.218330977	0.036920601	0.84766165
ENSG00000146409	SLC18B1	0.989298506	4.498380553	2.247491378	0.034729944	0.84103712
ENSG00000188215	DCUN1D3	-1.205655566	1.476747805	-2.217984389	0.036947377	0.84766165
ENSG00000161681	SHANK1	-2.063640314	2.098041254	-2.216829066	0.037036762	0.84766165
ENSG00000104218	CSPP1	-1.021069408	6.646350661	-2.280874564	0.032367926	0.836435787
ENSG00000141293	SKAP1	1.539505371	3.119396172	2.216302089	0.037077598	0.84766165
ENSG00000118242	MREG	1.493599656	1.941191461	2.215376653	0.03714941	0.84766165
ENSG00000198791	CNOT7	1.178076718	3.048387748	2.21151811	0.037450191	0.84766165
ENSG00000257704	PRR24	-1.75017513	1.908266643	-2.208053933	0.037722112	0.84766165
ENSG00000066468	FGFR2	-1.724867181	6.084187125	-2.251467515	0.034440592	0.841011466
ENSG00000111203	ITFG2	0.85935556	4.964779034	2.24745255	0.03473278	0.84103712
ENSG00000134504	KCTD1	1.163939501	3.87249001	2.214838293	0.037191244	0.84766165
ENSG00000102316	MAGED2	-0.988881407	8.732255969	-2.305726973	0.030705877	0.83357425
ENSG00000100097	LGALS1	-0.947544071	9.053656457	-2.307778441	0.030572235	0.83357425
ENSG00000177732	SOX12	-1.351961219	1.866602126	-2.203239595	0.038102991	0.84766165
ENSG00000157077	ZFYVE9	0.827790843	3.914528759	2.210772372	0.037508577	0.84766165
ENSG00000102243	VGLL1	2.301436834	1.813032606	2.196083567	0.03867558	0.84766165
ENSG00000203727	SAMD5	1.345649136	1.189276795	2.194540109	0.038800098	0.84766165
ENSG00000121104	FAM117A	-1.656828142	2.691808052	-2.194164002	0.038830496	0.84766165
ENSG00000111962	UST	1.440085533	2.609231233	2.19574349	0.038702984	0.84766165
ENSG00000011105	TSPAN9	1.17346943	5.234961293	2.250192219	0.034533157	0.84103712
ENSG00000124493	GRM4	-0.928606112	0.64348565	-2.205331121	0.037937096	0.84766165
ENSG00000181092	ADIPOQ	-1.740835568	1.528209654	-2.196429209	0.038647745	0.84766165
ENSG00000196557	CACNA1H	-1.531811135	3.088020755	-2.190477164	0.039129615	0.84766165
ENSG00000251247	ZNF345	-1.008577316	2.173425954	-2.189371844	0.039219697	0.84766165
ENSG00000181649	PHLDA2	1.416098004	2.452351961	2.188848561	0.03926241	0.84766165
ENSG00000108370	RGS9	1.487452595	1.970564944	2.188705113	0.039274126	0.84766165
ENSG00000196993	NPIPB9	1.516649445	1.141477291	2.188258348	0.039310636	0.84766165
ENSG00000168539	CHRM1	-1.162260473	0.844748225	-2.198529606	0.038478988	0.84766165
ENSG00000175471	MCTP1	1.28519208	3.615080583	2.198663339	0.038468266	0.84766165
ENSG00000156299	TIAM1	-1.107695446	5.527156401	-2.222008551	0.036637566	0.84766165
ENSG00000198933	TBKBP1	-1.037460193	4.252549639	-2.19504965	0.038758951	0.84766165
ENSG00000182575	NXPH3	-0.93473672	0.686836567	-2.198419326	0.038487832	0.84766165
ENSG00000156931	VPS8	0.762633364	6.398077715	2.26418333	0.033529929	0.836435787
ENSG00000267270	AC139100.2	-1.039478176	0.701632965	-2.196250591	0.038662127	0.84766165
ENSG00000225614	ZNF469	-1.683107387	2.377356274	-2.183682314	0.039686368	0.84766165
ENSG00000115290	GRB14	1.879612992	2.91482501	2.183422345	0.039707811	0.84766165
ENSG00000140564	FURIN	-0.968503986	6.997131116	-2.265508164	0.033436324	0.836435787
ENSG00000163083	INHBB	1.574797714	2.213008958	2.18319328	0.039726714	0.84766165
ENSG00000168394	TAP1	1.157934696	6.782181741	2.269667091	0.033144027	0.836435787

ENSG00000173040	EVC2	1.6636168	3.541376201	2.191976693	0.039007705	0.84766165
ENSG00000111641	NOP2	0.820849217	6.692810393	2.2649501	0.033475724	0.836435787
ENSG00000049249	TNFRSF9	1.73665425	1.532694299	2.177495767	0.040199508	0.84766165
ENSG00000153113	CAST	0.972723648	7.924905278	2.280007037	0.032427402	0.836435787
ENSG00000160285	LSS	-1.010176405	6.334556597	-2.240572768	0.035238712	0.844357795
ENSG00000179314	WSCD1	1.217528007	0.933683462	2.177657641	0.040186006	0.84766165
ENSG00000181617	FDCSP	2.692542105	2.170491485	2.175501272	0.040366216	0.84766165
ENSG00000149554	CHEK1	0.953961715	4.960160163	2.223875329	0.03649465	0.84766165
ENSG00000160113	NR2F6	0.999378103	4.327260101	2.197118308	0.038592306	0.84766165
ENSG00000113407	TARS	0.836600064	7.667902495	2.273795787	0.032856164	0.836435787
ENSG00000107295	SH3GL2	1.273401063	1.081792186	2.172492398	0.040618894	0.84766165
ENSG00000139318	DUSP6	-1.057578166	3.987895411	-2.177415866	0.040206175	0.84766165
ENSG00000152953	STK32B	-1.866116725	2.532143393	-2.171981443	0.040661945	0.84766165
ENSG00000151575	TEX9	1.19709159	2.660963735	2.171669107	0.040688281	0.84766165
ENSG00000112981	NME5	-1.344343139	0.921539293	-2.182319999	0.039798853	0.84766165
ENSG00000136319	TTC5	-1.036290817	4.041357381	-2.175576448	0.040359922	0.84766165
ENSG00000183837	PNMA3	-0.965955559	0.701632965	-2.183829633	0.039674222	0.84766165
ENSG00000111837	MAK	1.290383696	1.046706413	2.168492667	0.040956997	0.84766165
ENSG00000115514	TXNDC9	1.364046614	1.254500241	2.166736277	0.041106269	0.84766165
ENSG00000133069	TMCC2	-1.535240405	2.728131573	-2.166516252	0.041125004	0.84766165
ENSG00000134258	VTCN1	2.18902922	3.148565923	2.166552978	0.041121876	0.84766165
ENSG00000155016	CYP2U1	-1.194207089	2.100617696	-2.165553503	0.041207068	0.84766165
ENSG00000110013	SIAE	1.00728435	4.669299716	2.201196497	0.03826568	0.84766165
ENSG00000107863	ARHGAP21	-1.291519376	4.239099454	-2.169120913	0.040903722	0.84766165
ENSG00000168062	BATF2	1.227748012	0.660515451	2.165704143	0.041194218	0.84766165
ENSG00000198643	FAM3D	1.271644028	0.811276404	2.165245733	0.041233334	0.84766165
ENSG00000185499	MUC1	2.423213503	5.774658931	2.231697545	0.035901299	0.84766165
ENSG00000156234	CXCL13	1.882130567	1.873241213	2.162563606	0.041462872	0.84766165
ENSG00000157823	AP3S2	-1.190118468	2.778017601	-2.162150892	0.041498294	0.84766165
ENSG00000119787	ATL2	1.226077477	6.102497616	2.239555472	0.03531409	0.844357795
ENSG00000141994	DUS3L	-0.869307885	5.232850342	-2.203179342	0.03810778	0.84766165
ENSG00000176194	CIDEA	-1.524758193	1.07177639	-2.172352522	0.040630675	0.84766165
ENSG00000111716	LDHB	1.269901642	7.738842299	2.266436719	0.03337086	0.836435787
ENSG00000165097	KDM1B	0.817469481	4.991110464	2.209279546	0.037625703	0.84766165
ENSG00000145979	TBC1D7	0.830800725	5.360374977	2.22137288	0.036686347	0.84766165
ENSG00000140623	SEPT12	-0.977086166	0.658282048	-2.173394524	0.040542986	0.84766165
ENSG00000163624	CDS1	1.789431698	3.026027617	2.157284485	0.041918037	0.84766165
ENSG00000137434	C6orf52	1.284767982	1.068952403	2.157227912	0.041922939	0.84766165
ENSG00000213694	S1PR3	-1.498812851	2.445266662	-2.155140306	0.042104194	0.84766165
ENSG00000171119	NRTN	-1.329642573	1.517757486	-2.159440494	0.041731604	0.84766165
ENSG00000111669	TPI1	0.948358042	4.636331031	2.194155283	0.038831201	0.84766165
ENSG00000182010	RTKN2	1.544122169	1.415464265	2.153421883	0.042253925	0.84766165
ENSG00000244752	CRYBB2	1.348957134	0.715391087	2.15475766	0.042137493	0.84766165
ENSG00000171094	ALK	-1.992215115	1.381992444	-2.1638486	0.041352757	0.84766165
ENSG00000077279	DCX	-1.083208678	0.715391087	-2.166659606	0.041112797	0.84766165
ENSG00000185019	UBOX5	-1.021923599	3.751189843	-2.152167892	0.042363492	0.84766165
ENSG00000145495	MARCH6	0.818118887	7.261227923	2.249245265	0.034602037	0.84103712
ENSG00000130540	SULT4A1	1.651155758	1.277977438	2.149920044	0.042560539	0.84766165
ENSG00000189221	MAOA	-1.710002315	3.456814491	-2.150107056	0.042544114	0.84766165
ENSG00000168143	FAM83B	1.287345344	0.969188061	2.150400298	0.04251837	0.84766165
ENSG00000147689	FAM83A	1.198119859	0.703866368	2.150585837	0.042502089	0.84766165
ENSG00000203666	EFCAB2	-1.158039668	3.025814939	-2.14768333	0.04275743	0.84766165
ENSG00000134321	RSAD2	1.456696194	3.129287947	2.151847092	0.042391563	0.84766165
ENSG00000096063	SRPK1	1.011088566	6.175852398	2.228641553	0.036132057	0.84766165
ENSG00000150967	ABCB9	-1.019212978	3.92459528	-2.149037232	0.042638152	0.84766165
ENSG00000135002	RFK	1.352127211	2.079389419	2.144352386	0.043052167	0.84766165
ENSG00000105379	ETFB	-1.161710195	4.900096857	-2.17348606	0.040535291	0.84766165
ENSG00000076321	KLHL20	-0.721997999	4.979917852	-2.176909524	0.040248444	0.84766165
ENSG00000224186	C5orf66	0.997651074	0.658282048	2.148037041	0.042726239	0.84766165
ENSG00000115963	RND3	-1.030570087	5.247349183	-2.182297082	0.039800748	0.84766165
ENSG00000010030	ETV7	1.405352365	2.984494883	2.144962005	0.042998089	0.84766165
ENSG00000184731	FAM110C	1.22056988	0.801397307	2.144036411	0.043080221	0.84766165
ENSG00000099800	TIMM13	-0.857548311	5.668649943	-2.198515673	0.038480106	0.84766165

ENSG00000181240	SLC25A41	-1.171599308	0.855370121	-2.153012008	0.042289709	0.84766165
ENSG00000138039	LHCGR	0.950122358	0.571580214	2.145222334	0.042975014	0.84766165
ENSG00000127824	TUBA4A	0.952972627	5.461936714	2.199379612	0.038410885	0.84766165
ENSG00000184292	TACSTD2	2.064652749	2.97916839	2.136860982	0.043721746	0.851514668
ENSG00000125409	TEKT3	1.005765154	0.780069656	2.138351859	0.043587748	0.851514668
ENSG00000135069	PSAT1	1.494703716	2.795476384	2.137217227	0.043689693	0.851514668
ENSG00000172830	SSH3	0.865618453	5.056264633	2.186255859	0.03947466	0.84766165
ENSG00000157680	DGKI	-1.650296328	2.524117687	-2.133910047	0.043988066	0.851514668
ENSG00000137965	IFI44	1.184891487	6.134167712	2.220514585	0.036752306	0.84766165
ENSG00000165795	NDRG2	-1.682592619	7.92550981	-2.225446029	0.036374795	0.84766165
ENSG00000103489	XYLT1	-1.91217194	4.032419193	-2.133710233	0.044006151	0.851514668
ENSG00000163491	NEK10	-1.127342886	1.074565319	-2.141211695	0.043331747	0.849898052
ENSG00000155974	GRIP1	1.420663673	3.046692944	2.133655415	0.044011114	0.851514668
ENSG00000073282	TP63	2.004156475	1.900264226	2.134997667	0.043889739	0.851514668
ENSG00000176597	B3GNT5	1.37800826	1.482795063	2.130153854	0.044329173	0.852413065
ENSG00000128928	IVD	-0.958768079	6.005112126	-2.201599322	0.038233554	0.84766165
ENSG00000171681	ATF7IP	0.792571803	5.582245136	2.201334748	0.038254651	0.84766165
ENSG00000163694	RBM47	1.4022576	3.44032769	2.134987298	0.043890675	0.851514668
ENSG00000198336	MYL4	-1.013627005	0.658282048	-2.145835487	0.04292071	0.84766165
ENSG00000112742	TTK	1.28229357	4.417600457	2.170157813	0.040815932	0.84766165
ENSG00000112242	E2F3	1.07506105	3.514198591	2.138714453	0.043555215	0.851514668
ENSG00000130234	ACE2	1.347856737	0.829951827	2.130305243	0.04431538	0.852413065
ENSG00000111640	GAPDH	0.76711392	9.219434474	2.251148453	0.034463729	0.841011466
ENSG00000118898	PPL	2.149408787	4.927857098	2.171877752	0.040670686	0.84766165
ENSG00000168876	ANKRD49	-1.204016591	1.39004597	-2.132963323	0.044073816	0.851677644
ENSG00000105088	OLFM2	-2.029882977	4.931554941	-2.154178387	0.042187949	0.84766165
ENSG00000205176	REXO1L1P	1.190524291	0.664813302	2.123971951	0.044895754	0.855191178
ENSG00000132016	C19orf57	1.506818572	2.607500347	2.121455409	0.04512826	0.855191178
ENSG00000047230	CTPS2	1.150228158	5.582712516	2.195140875	0.038751588	0.84766165
ENSG00000171295	ZNF440	0.970651103	3.829778226	2.134918149	0.043896921	0.851514668
ENSG00000184347	SLIT3	-1.286410159	6.374363735	-2.201980114	0.038203207	0.84766165
ENSG00000157911	PEX10	-1.082588501	4.10972892	-2.126410016	0.044671528	0.85480603
ENSG00000226397	C12orf77	0.946770326	0.708164219	2.12161205	0.045113756	0.855191178
ENSG00000117020	AKT3	-2.037688414	3.521748411	-2.116317834	0.045606291	0.855191178
ENSG00000114331	ACAP2	0.703615476	4.900925109	2.166476753	0.041128368	0.84766165
ENSG00000178997	EXD1	-1.020695956	1.024683148	-2.127284736	0.044591327	0.85480603
ENSG00000112419	PHACTR2	1.411975738	3.113959854	2.119196249	0.045337908	0.855191178
ENSG00000015532	XYLT2	-0.799845287	5.382700153	-2.168742133	0.040935835	0.84766165
ENSG00000111206	FOXM1	1.350059443	5.296921707	2.183270644	0.039720329	0.84766165
ENSG00000100304	TTLL12	-0.921706536	6.329625116	-2.189441079	0.039214049	0.84766165
ENSG00000165548	TMEM63C	-1.424982789	2.061456796	-2.113031313	0.045914473	0.855191178
ENSG00000118946	PCDH17	1.182366674	0.758742004	2.11039208	0.046163311	0.856203916
ENSG00000016402	IL20RA	1.677906603	1.541148812	2.107696481	0.046418714	0.858707298
ENSG00000012223	LTF	3.554970346	4.699158047	2.162886243	0.041435199	0.84766165
ENSG00000171792	RHNO1	1.01833695	4.266925284	2.142224755	0.043241387	0.849187246
ENSG00000165304	MELK	1.340889131	5.011744442	2.167138479	0.041072044	0.84766165
ENSG00000140961	OSGIN1	-1.479278264	1.996067097	-2.104066374	0.046764666	0.859232304
ENSG00000131711	MAP1B	-1.059492241	5.693994128	-2.171568556	0.040696763	0.84766165
ENSG00000115850	LCT	1.105260549	0.478347125	2.109600114	0.046238217	0.856580712
ENSG00000142207	URB1	-0.856970662	6.828337717	-2.192705657	0.038948565	0.84766165
ENSG00000205138	SDHAF1	-1.060204238	0.686836567	-2.11660902	0.045579076	0.855191178
ENSG00000166831	RBPMS2	1.112872933	2.964212091	2.100072238	0.047147984	0.859232304
ENSG00000152213	ARL11	-1.050164257	0.686836567	-2.116836163	0.045557857	0.855191178
ENSG00000153956	CACNA2D1	1.598506852	2.479820968	2.098901755	0.047260848	0.859645156
ENSG00000117425	PTCH2	-1.560978451	3.692244568	-2.095686322	0.047572148	0.859645156
ENSG00000121005	CRISPLD1	-1.813164741	6.997444444	-2.173030966	0.040573562	0.84766165
ENSG00000064300	NGFR	-1.711356931	1.819858982	-2.09651991	0.047491268	0.859645156
ENSG00000170275	CRTAP	-0.858114606	8.128976009	-2.211542001	0.037448322	0.84766165
ENSG00000198899	MT-ATP6	-0.856633872	7.06629964	-2.191389299	0.039055418	0.84766165
ENSG00000088053	GP6	-1.030529194	0.730187485	-2.113243878	0.045894484	0.855191178
ENSG00000135318	NT5E	1.635808667	3.911193582	2.112997569	0.045917647	0.855191178
ENSG00000198886	MT-ND4	-0.720865011	9.943159169	-2.224240132	0.036466781	0.84766165
ENSG00000105131	EPHX3	1.234480311	0.888099142	2.095307696	0.047608925	0.859645156

ENSG00000070018	LRP6	1.383736452	5.223548658	2.153851314	0.042216462	0.84766165
ENSG00000185880	TRIM69	1.005228737	3.990663976	2.113126305	0.045905539	0.855191178
ENSG00000147697	GSDMC	2.225208198	3.175792436	2.09418799	0.047717835	0.860621317
ENSG00000135617	PRADC1	-1.05141431	3.748743524	-2.095978215	0.047543813	0.859645156
ENSG00000174136	RGMB	1.393870869	2.017563704	2.089742192	0.048152471	0.862834039
ENSG00000147394	ZNF185	1.514897473	4.478561132	2.128844076	0.044448676	0.853664845
ENSG00000168724	DNAJC21	0.790688711	6.254060185	2.181508052	0.039866032	0.84766165
ENSG00000152242	C18orf25	1.330503641	2.431221255	2.086698496	0.048452076	0.866202896
ENSG00000127129	EDN2	1.839799329	2.459294269	2.085951696	0.048525842	0.866202896
ENSG00000184661	CDCA2	1.453489385	3.264095261	2.101331953	0.047026785	0.859232304
ENSG00000141959	PFKL	-0.685748336	8.742209377	-2.210527875	0.037527737	0.84766165
ENSG00000136273	HUS1	0.801333966	5.018482974	2.146248569	0.04288416	0.84766165
ENSG00000170412	GPRC5C	-1.695563456	4.374680863	-2.101351496	0.047024907	0.859232304
ENSG00000136244	IL6	1.351663366	1.269447376	2.08262795	0.04885537	0.866289095
ENSG00000072858	SIDT1	1.290759546	1.618141279	2.079220424	0.049195285	0.866289095
ENSG00000100811	YY1	0.732204991	5.78605785	2.16256303	0.041462921	0.84766165
ENSG00000065675	PRKCQ	1.403269484	1.701647597	2.077816491	0.049335948	0.866289095
ENSG00000157111	TMEM171	1.08614037	0.736718738	2.082062813	0.048911599	0.866289095
ENSG00000157379	DHRS1	-0.994327381	5.387988058	-2.123726306	0.044918402	0.855191178
ENSG00000017483	SLC38A5	1.489579937	4.138411938	2.113342528	0.04588521	0.855191178
ENSG00000107738	C10orf54	-1.263284324	5.17280841	-2.126873597	0.044629007	0.85480603
ENSG00000183814	LIN9	0.893088807	4.378748362	2.113184879	0.045900031	0.855191178
ENSG00000168913	ENHO	-0.92624926	0.614931131	-2.097532794	0.047393159	0.859645156
ENSG00000197586	ENTPD6	0.642786671	6.886122415	2.181578624	0.039860188	0.84766165
ENSG00000126934	MAP2K2	-0.749547521	6.400301732	-2.162689752	0.04145205	0.84766165
ENSG00000122547	EEPD1	-1.320882879	3.643806069	-2.076356517	0.049482608	0.867521335
ENSG00000187193	MT1X	-0.927431054	0.614931131	-2.09629815	0.047512773	0.859645156
ENSG00000141013	GAS8	-1.314602678	4.143846993	-2.079020455	0.049215298	0.866289095
ENSG00000136099	PCDH8	-1.074553793	0.816889319	-2.093035003	0.047830215	0.86165776
ENSG00000126749	EMG1	0.811217253	5.804132779	2.160772339	0.041616812	0.84766165
ENSG00000148346	LCN2	2.564275742	3.140384587	2.078848306	0.049232533	0.866289095
ENSG00000176623	RMDN1	-0.872544031	6.338936974	-2.154006316	0.042202948	0.84766165
ENSG00000133059	DSTYK	-0.924447142	6.112375191	-2.148300405	0.042703029	0.84766165
ENSG00000145868	FBXO38	-0.717604481	5.653840047	-2.130793487	0.044270919	0.852413065
ENSG00000123243	ITIH5	-1.728595269	4.435736136	-2.075801646	0.049538449	0.867531023
ENSG00000128567	PODXL	1.074710459	5.014558016	2.124086364	0.044885209	0.855191178
ENSG00000198804	MT-CO1	-0.796141002	8.698783688	-2.185899075	0.039503949	0.84766165
ENSG00000117289	TXNIP	-1.125442585	7.49337501	-2.168444405	0.040961092	0.84766165
ENSG00000112118	MCM3	1.066516753	7.770033968	2.183301281	0.039717801	0.84766165
ENSG00000176715	ACSF3	-0.841100477	4.578377824	-2.085410642	0.048579348	0.866202896
ENSG00000114021	NIT2	-0.644381842	6.16399638	-2.143704804	0.043109681	0.84766165
ENSG00000082805	ERC1	0.8681879	5.473104881	2.136725961	0.043733899	0.851514668
ENSG00000054983	GALC	1.000579216	4.84687197	2.113850823	0.045837451	0.855191178
ENSG00000145332	KLHL8	-0.903771161	4.290967664	-2.07406312	0.049713776	0.868662423
ENSG00000177508	IRX3	1.188745614	4.215917983	2.090434468	0.048084559	0.862834039
ENSG00000176485	PLA2G16	1.331872205	4.956677553	2.118584503	0.045394828	0.855191178
ENSG00000125170	DOK4	-0.794017768	4.830300085	-2.09179469	0.047951372	0.862834039
ENSG00000205426	KRT81	2.690142909	5.125938797	2.112047561	0.046007084	0.855191178
ENSG00000231925	TAPBP	0.707884608	8.814463711	2.178233655	0.040137991	0.84766165
ENSG00000008517	IL32	1.642927051	5.591204883	2.130738423	0.044275932	0.852413065
ENSG00000136156	ITM2B	1.06591794	8.661725126	2.177800318	0.040174108	0.84766165
ENSG00000159899	NPR2	-1.657471506	6.143080296	-2.101205112	0.047038976	0.859232304
ENSG00000148180	GSN	-0.985331554	10.19986139	-2.179911923	0.039998391	0.84766165
ENSG00000115318	LOXL3	-1.055428417	5.063503171	-2.078413594	0.049276079	0.866289095
ENSG00000146416	AIG1	0.939169214	5.015854586	2.104852611	0.046689541	0.859232304
ENSG00000006625	GGCT	0.862580047	5.247555293	2.103301442	0.046837859	0.859232304
ENSG00000149547	EI24	0.701022366	5.754029617	2.118285308	0.04542269	0.855191178
ENSG00000197603	C5orf42	1.132228879	5.411792331	2.105102339	0.046665702	0.859232304
ENSG00000102804	TSC22D1	-0.83864389	6.361045386	-2.112436115	0.045970485	0.855191178
ENSG00000108819	PPP1R9B	-0.829904379	6.338797724	-2.121553854	0.045119144	0.855191178
ENSG00000078269	SYNJ2	0.900844322	5.392694883	2.100340016	0.047122197	0.859232304
ENSG00000150753	CCT5	0.899703015	5.334843601	2.100906002	0.047067735	0.859232304
ENSG00000130816	DNMT1	0.831836165	8.42791284	2.156429289	0.041992196	0.84766165

ENSG00000082781	ITGB5	-1.145611359	8.229501549	-2.149465714	0.042600466	0.84766165
ENSG00000111364	DDX55	-0.774222751	6.132276059	-2.107236087	0.046462462	0.858707298
ENSG00000101150	TPD52L2	-0.731006706	6.428209629	-2.115676521	0.045666282	0.855191178
ENSG00000078043	PIAS2	0.688718167	5.177528875	2.089559006	0.048170456	0.862834039
ENSG00000214530	STARD10	0.957197166	5.911903738	2.111547694	0.046054206	0.855191178
ENSG00000078070	MCCC1	0.961544252	5.944102155	2.101906857	0.046971566	0.859232304
ENSG00000117335	CD46	0.951342841	6.891457091	2.121398626	0.045133519	0.855191178
ENSG00000026950	BTN3A1	0.983794853	5.128958857	2.082941191	0.048824229	0.866289095
ENSG00000101439	CST3	-1.295398245	9.28712809	-2.145889412	0.042915937	0.84766165
ENSG00000141504	SAT2	-0.716074227	6.005943513	-2.083018978	0.048816499	0.866289095
ENSG00000134243	SORT1	1.16628592	6.626173751	2.102183224	0.046945042	0.859232304
ENSG00000160948	VPS28	-1.037559215	8.546438104	-2.120976632	0.045172617	0.855191178
ENSG00000120697	ALG5	0.767479296	5.959565341	2.081204678	0.048997091	0.866289095
ENSG00000100823	APEX1	-0.867771091	7.138299219	-2.098552454	0.047294577	0.859645156
ENSG00000101773	RBBP8	0.815879125	6.224340152	2.082003319	0.048917522	0.866289095
ENSG00000184428	TOP1MT	-1.172073399	6.96301798	-2.079874122	0.049129912	0.866289095
ENSG00000070961	ATP2B1	-0.924371503	6.649109858	-2.071926351	0.049930026	0.870328099
ENSG00000151208	DLG5	0.873766113	7.011947178	2.089645722	0.048161941	0.862834039
ENSG00000105699	LSR	2.435602321	6.698847454	2.077759536	0.049341662	0.866289095
ENSG00000135317	SNX14	0.817320447	6.704619235	2.077605561	0.049357112	0.866289095
ENSG00000162337	LRP5	1.227927806	7.2454955	2.085542618	0.048566292	0.866202896
ENSG00000116649	SRM	-1.060210823	7.592031635	-2.074197574	0.049700197	0.868662423
ENSG00000115415	STAT1	0.873683549	8.266155312	2.07343139	0.049777622	0.868810531
