# Supplementary material for: Genomic and transcriptomic heterogeneity in metaplastic carcinomas of the breast
Source: NPJ Breast Cancer. 2017 Dec 1;3:48. doi: 10.1038/s41523-017-0048-0 (PMC5711926; doi:10.1038/s41523-017-0048-0)
Supplement: Supplementary file 28 — Supplementary Table 16 [file 41523_2017_48_MOESM28_ESM.pdf]

Supplementary Table 16: List of transcripts for which gene expression significantly correlates with gene copy number.

RefSeq	HUGO Gene Symbol	Chromosome	Start	End	pearson.p	pearson.adjp
NM_003324.3	TULP3	12	3000033	3050297	9.29E-10	1.64E-05
NM_024551.2	ADIPOR2	12	1800193	1897844	9.60E-10	1.64E-05
NM_015971.2	MRPS7	17	73257749	73262456	3.10E-09	3.43E-05
NM_005057.2	RBBP5	1	205055270	205091143	4.42E-09	3.43E-05
NM_021646.1	ZNF500	16	4800815	4817166	5.02E-09	3.43E-05
NM_017798.2	YTHDF1	20	61826781	61847586	6.52E-09	3.72E-05
NM_001002296.1	GOLGA7	8	41347915	41368499	1.03E-08	5.02E-05
NM_004064.2	CDKN1B	12	12867992	12875305	1.29E-08	5.19E-05
NM_017755.4	NSUN2	5	6599352	6633404	1.36E-08	5.19E-05
NM_003416.1	ZNF7	8	146052849	146072894	2.85E-08	9.75E-05
NM_032272.4	MAF1	8	145159402	145162514	3.51E-08	0.000102993
NM_001083617.1	RB1CC1	8	53535016	53658403	3.61E-08	0.000102993
NM_001089591.1	UQCRH	1	46769303	46782448	5.19E-08	0.000134461
NM_198935.1	SS18L1	20	60718822	60757540	5.50E-08	0.000134461
NM_014462.1	LSM1	8	38020839	38034248	6.19E-08	0.00014122
NM_144584.1	C1orf59	1	109190912	109217815	6.66E-08	0.000142372
NM_032842.3	TMEM209	7	129804555	129847610	7.83E-08	0.000156274
NM_201224.1	DDX47	12	12966280	12982909	8.22E-08	0.000156274
NM_033301.1	RPL8	8	146015150	146017972	8.93E-08	0.000160805
NM_024844.3	NUP85	17	73201597	73231854	9.98E-08	0.000163771
NM_018120.3	ARMC1	8	66514694	66546442	1.01E-07	0.000163771
NM_030782.3	CLPTM1L	5	1317859	1345214	1.18E-07	0.000183817
NM_004047.3	ATP6V0B	1	44440159	44443967	1.52E-07	0.000225859
NM_018657.3	MYNN	3	169490853	169505333	1.90E-07	0.000260292
NM_024790.5	CSPP1	8	67974661	68108498	2.01E-07	0.000260292
NM_004146.4	NDUFB7	19	14676892	14682886	2.09E-07	0.000260292
NM_016023.2	OTUD6B	8	92082424	92099323	2.10E-07	0.000260292
NM_006852.2	TLK2	17	60556386	60692839	2.13E-07	0.000260292
NM_018463.2	ITFG2	12	2921788	2934237	2.57E-07	0.000303371
NM_016027.1	LACTB2	8	71547553	71581409	2.67E-07	0.000304749
NM_001018677.1	FNTA	8	42889337	42940931	3.21E-07	0.000310872
NM_006766.2	MYST3	8	41786997	41909508	3.22E-07	0.000310872
NM_022066.2	UBE2O	17	74385614	74449288	3.28E-07	0.000310872
NM_016208.2	VPS28	8	145649000	145653931	3.32E-07	0.000310872
NM_016312.2	WBP11	12	14939414	14956401	3.36E-07	0.000310872
NM_014740.2	EIF4A3	17	78109019	78120938	3.53E-07	0.000310872
NM_021177.3	VARS	6	31745295	31763730	3.54E-07	0.000310872
NM_014652.2	IPO13	1	44412611	44433694	3.60E-07	0.000310872
NM_002568.3	PABPC1	8	101698044	101735037	3.61E-07	0.000310872
NM_001033002.2	RPAIN	17	5322961	5336196	3.63E-07	0.000310872
NM_015169.3	RRS1	8	67341263	67342966	3.89E-07	0.000320743
NM_006421.3	ARFGEF1	8	68085747	68255912	3.99E-07	0.000320743
NM_014637.2	MTFR1	8	66556124	66683496	4.20E-07	0.000320743
NM_005131.2	THOC1	18	214527	268059	4.28E-07	0.000320743
NM_018686.3	CMAS	12	22199159	22218602	4.31E-07	0.000320743
NM_173685.1	NSMCE2	8	126103921	126379362	4.37E-07	0.000320743
NM_016023.2	OTUD6B	8	92082424	92099323	4.41E-07	0.000320743
NM_005439.1	MLF2	12	6857170	6862266	4.66E-07	0.000332109

BC014384	TMEM33	4	41937137	41962589	4.88E-07	0.000341101
NM_032847.1	C8orf76	8	124232196	124279627	5.37E-07	0.000364427
NM_025078.3	PQLC1	18	77662420	77711664	5.43E-07	0.000364427
NM_032338.2	LLPH	12	66516842	66524533	5.56E-07	0.000366053
NM_004374.2	COX6C	8	100885428	100906290	5.74E-07	0.00037054
NM_003941.2	WASL	7	123321989	123389121	6.32E-07	0.000394963
NM_175573.1	ADRM1	20	60877149	60883918	6.35E-07	0.000394963
NM_178547.2	ZBTB8OS	1	33065854	33116533	6.87E-07	0.000419781
NM_201437.1	TCEA1	8	54879112	54935089	7.10E-07	0.000419788
NM_001031725.3	DDX59	1	200593024	200639126	7.12E-07	0.000419788
NM_000989.2	RPL30	8	99037079	99058697	7.63E-07	0.000442699
NM_176814.3	ZNF800	7	126986844	127071978	8.06E-07	0.000449794
NM_023008.2	KRI1	19	10663761	10676713	8.07E-07	0.000449794
NM_024038.2	C19orf43	19	12841455	12845529	8.15E-07	0.000449794
NM_015117.1	ZC3H3	8	144519825	144623623	8.47E-07	0.000453038
NM_007055.2	POLR3A	10	79729008	79789303	8.47E-07	0.000453038
NM_021174.4	KIAA1967	8	22462145	22479027	9.04E-07	0.00046915
NM_032111.2	MRPL14	6	44081194	44095194	9.05E-07	0.00046915
NM_006304.1	SHFM1	7	96110938	96339203	1.03E-06	0.000521157
NM_001002246.1	ANAPC11	17	79849599	79858363	1.04E-06	0.000521157
NM_001008239.2	C18orf25	18	43753988	43846954	1.06E-06	0.000521157
NM_006708.2	GLO1	6	38643719	38670917	1.07E-06	0.000521157
NM_014885.3	ANAPC10	4	145888264	146019693	1.10E-06	0.000525121
XM_001134346.1	THOC4	17	79845717	79849462	1.10E-06	0.000525121
NM_001923.3	DDB1	11	61066920	61100666	1.24E-06	0.000564763
NM_004792.2	PPIG	2	170440850	170497916	1.24E-06	0.000564763
NM_006958.2	ZNF16	8	146155744	146176274	1.26E-06	0.000564763
NM_018686.3	CMAS	12	22199159	22218602	1.29E-06	0.000564763
NM_001007026.1	ATN1	12	7033626	7051482	1.30E-06	0.000564763
NM_006004.2	UQCRH	1	46769303	46782448	1.31E-06	0.000564763
NM_004674.2	ASH2L	8	37962760	38001594	1.32E-06	0.000564763
NM_003801.3	GPAA1	8	145137493	145141119	1.32E-06	0.000564763
NM_004999.3	MYO6	6	76458909	76629254	1.36E-06	0.000572405
NM_023018.3	NADK	1	1682671	1711896	1.37E-06	0.000572405
NM_017767.1	SLC39A4	8	145635126	145642279	1.40E-06	0.000575611
NM_018998.2	FBXW5	9	139834887	139839148	1.42E-06	0.000576605
NM_002095.4	GTF2E2	8	30435835	30515768	1.43E-06	0.000577328
NM_001002246.1	ANAPC11	17	79849599	79858363	1.48E-06	0.000587147
NM_005494.2	DNAJB6	7	157128075	157210133	1.49E-06	0.000587147
NM_032901.2	C12orf62	12	50505900	50514234	1.61E-06	0.000617937
NM_024947.2	PHC3	3	169804520	169899537	1.63E-06	0.000617937
NM_015545.2	PTCD1	7	99014362	99063820	1.64E-06	0.000617937
NM_014345.1	ZNF318	6	43299513	43337181	1.65E-06	0.000617937
NM_014230.2	SRP68	17	74035191	74068607	1.66E-06	0.000617937
NM_133433.2	NIPBL	5	36876861	37066515	1.71E-06	0.000628469
NM_001010853.1	PM20D2	6	89855769	89875284	1.74E-06	0.000634875
NM_003620.2	PPM1D	17	58677554	58742036	1.79E-06	0.000646241
NM_001014286.2	FAM48A	13	37583449	37633850	1.88E-06	0.000661211
NM_020463.1	SMEK2	2	55774428	55846015	1.89E-06	0.000661211
NM_005034.3	POLR2K	8	101162812	101166230	1.89E-06	0.000661211
NM_021074.1	NDUFV2	18	9102628	9134343	1.93E-06	0.000668529
NM_001916.3	CYC1	8	145149930	145152428	1.97E-06	0.000673224
NM_015476.2	C18orf10	18	34376035	34409158	2.09E-06	0.00070687

NM_198256.2	E2F6	2	11584501	11606297	2.27E-06	0.000761855
NM_012319.2	SLC39A6	18	33688495	33709357	2.34E-06	0.00076757
NM_198401.2	ANKRD46	8	101521980	101572012	2.37E-06	0.00076757
NM_016208.2	VPS28	8	145649000	145653931	2.38E-06	0.00076757
NM_004168.1	SDHA	5	218356	256815	2.38E-06	0.00076757
NM_012062.2	DNM1L	12	32832137	32898584	2.48E-06	0.000783905
NM_002794.3	PSMB2	1	36042938	36107445	2.49E-06	0.000783905
NM_006197.2	PCM1	8	17780349	17887453	2.50E-06	0.000783905
NM_001017416.1	USP1	1	62901968	62917475	2.53E-06	0.000786455
NM_006281.2	STK3	8	99413631	99955055	2.59E-06	0.000797744
NM_014445.3	SERP1	3	150259781	150321015	2.62E-06	0.000800021
NM_017583.3	TRIM44	11	35684353	35829775	2.79E-06	0.00084449
NM_138787.2	C11orf74	11	36616051	36680834	2.97E-06	0.000883171
NM_007051.2	FAF1	1	50905150	51425935	2.97E-06	0.000883171
NM_014319.3	LEMD3	12	65563371	65642107	2.99E-06	0.000883171
NM_032557.4	USP38	4	144106070	144144983	3.05E-06	0.000888741
NM_015397.1	DCAF12	9	34086522	34127397	3.08E-06	0.000888741
NM_006331.5	EMG1	12	7080087	7095921	3.09E-06	0.000888741
NM_006339.1	HMG20B	19	3572775	3579086	3.13E-06	0.000888741
NM_001042369.1	TROVE2	1	193028552	193060907	3.16E-06	0.000888741
NM_005359.3	SMAD4	18	48556583	48611415	3.17E-06	0.000888741
NM_016258.2	YTHDF2	1	29063133	29096287	3.29E-06	0.00091524
NM_003900.3	SQSTM1	5	179233398	179265078	3.35E-06	0.000923693
NM_032730.4	RTN4IP1	6	107019846	107077362	3.44E-06	0.000941934
NM_018979.2	WNK1	12	862089	1020618	3.48E-06	0.000943225
NM_004168.1	SDHA	5	218356	256815	3.50E-06	0.000943225
NM_006640.3	SEPT9	17	75277492	75496674	3.56E-06	0.000950831
NM_002949.2	MRPL12	17	79670407	79674546	3.65E-06	0.000965986
NM_207356.1	C1orf174	1	3805689	3816857	3.67E-06	0.000965986
NM_016271.3	RNF138	18	29671828	29711524	3.71E-06	0.000968734
NM_025188.2	TRIM45	1	117653682	117665209	3.74E-06	0.000969548
NM_022488.3	ATG3	3	112251356	112280893	3.77E-06	0.000969694
NM_018299.3	UBE2W	8	74650409	74791145	3.91E-06	0.000992885
NM_170686.1	ZNF398	7	148787977	148880116	3.93E-06	0.000992885
NM_001105079.1	FBRS	16	30670289	30682131	3.97E-06	0.000992885
NM_002027.2	FNTA	8	42889337	42940931	3.98E-06	0.000992885
NM_031905.2	ARMC10	7	102715328	102740205	4.04E-06	0.001002212
NM_031446.3	C18orf21	18	33552046	33559241	4.10E-06	0.001008456
NM_003410.2	ZFX	23	24167290	24234206	4.15E-06	0.001014585
NM_030621.2	DICER1	14	95552565	95624347	4.26E-06	0.001033222
NM_133368.1	RSPRY1	16	57220209	57274386	4.31E-06	0.001033222
NM_002485.4	NBN	8	90945564	91015456	4.32E-06	0.001033222
XM_001129080.1	MAEA	4	1283639	1333935	4.43E-06	0.001046247
NM_007342.1	NUPL2	7	23221446	23240630	4.44E-06	0.001046247
NM_001040273.1	TYSND1	10	71897737	71906432	4.46E-06	0.001046247
NM_002355.2	M6PR	12	9092976	9102252	4.53E-06	0.001050581
AK056312	ERC1	12	1100404	1605099	4.54E-06	0.001050581
NM_198990.3	NAPEPLD	7	102740023	102790007	4.61E-06	0.00105487
NM_005053.2	RAD23A	19	13056654	13064448	4.65E-06	0.00105487
NM_033224.3	PURB	7	44915896	44924960	4.65E-06	0.00105487
NM_007273.3	PHB2	12	7074848	7079916	4.71E-06	0.001057147
NM_000971.3	RPL7	8	74202506	74213303	4.73E-06	0.001057147
NM_030818.2	CCDC130	19	13858720	13874106	4.89E-06	0.00108663

NM_022156.3	DUS1L	17	80015754	80023680	5.01E-06	0.00110714
NM_014078.4	MRPL13	8	121393000	121457642	5.09E-06	0.001117332
NM_003903.3	CDC16	13	115000362	115038198	5.24E-06	0.001136327
NM_017704.2	ANKRD49	11	94227153	94232737	5.25E-06	0.001136327
NM_019030.2	DHX29	5	54552073	54603550	5.28E-06	0.001136327
NM_014846.3	KIAA0196	8	126036502	126104082	5.40E-06	0.001154286
NM_023080.1	C8orf33	8	146277764	146281416	5.49E-06	0.001167721
NM_015629.2	PRPF31	19	54618837	54635140	5.60E-06	0.001181888
NM_015155.1	LARP4B	10	855484	977564	5.66E-06	0.001188243
NM_198513.1	PHF20L1	8	133787618	133861052	5.80E-06	0.001206829
NM_006349.2	ZNHIT1	7	100860949	100867471	5.83E-06	0.001206829
NM_033414.2	ZNF622	5	16451628	16465901	5.85E-06	0.001206829
NM_031899.2	GORASP1	3	39138150	39149854	6.00E-06	0.001217295
NM_005662.4	VDAC3	8	42249142	42263415	6.02E-06	0.001217295
NM_024818.2	UBA5	3	132373290	132396941	6.04E-06	0.001217295
NM_006590.2	USP39	2	85829979	85876406	6.05E-06	0.001217295
NM_012086.2	GTF3C3	2	197627756	197664449	6.14E-06	0.001222873
NM_001001795.1	C8orf82	8	145751117	145754516	6.15E-06	0.001222873
NM_007055.2	POLR3A	10	79729008	79789303	6.28E-06	0.001242509
NM_032295.2	SLC37A3	7	139993493	140104233	6.44E-06	0.001266821
NM_016355.3	DDX47	12	12966280	12982909	6.53E-06	0.001277229
NM_001040876.1	ABCE1	4	146019084	146050676	6.57E-06	0.001277267
NM_015462.3	NOL11	17	65714059	65740310	6.74E-06	0.001302414
NM_001005368.1	ZNF32	10	44139307	44144304	6.82E-06	0.001303626
NM_005002.3	NDUFA9	12	4758283	4796397	6.82E-06	0.001303626
NM_006374.3	STK25	2	242432089	242449145	7.03E-06	0.001334791
NM_145214.2	TRIM11	1	228581377	228594541	7.06E-06	0.001334791
NM_014574.3	STRN3	14	31363005	31495607	7.16E-06	0.001339121
NM_018047.1	RBM22	5	150070356	150080669	7.16E-06	0.001339121
NM_145859.1	PDCD10	3	167401086	167452727	7.30E-06	0.00135732
NM_001084.4	PLOD3	7	100849258	100861701	7.45E-06	0.001377987
NM_178812.2	MTDH	8	98656407	98740998	7.51E-06	0.001382352
NM_018846.2	KLHL7	7	23145377	23215040	7.56E-06	0.001383228
NM_001039937.1	INTS6	13	51928213	52028400	7.87E-06	0.00142511
NM_014018.2	MRPS28	8	80830952	80942524	7.87E-06	0.00142511
NM_006571.2	DCTN6	8	30013813	30041156	8.14E-06	0.00145647
NM_017956.2	TRMT12	8	125463048	125474391	8.15E-06	0.00145647
NM_006330.2	LYPLA1	8	54958938	55014577	8.20E-06	0.00145647
NM_019042.3	PUS7	7	105080108	105162714	8.25E-06	0.00145647
NM_203497.2	COMMD6	13	76099350	76123575	8.26E-06	0.00145647
NM_006837.2	COPS5	8	67955314	67996018	8.48E-06	0.001486558
NM_001006.3	RPS3A	4	152020725	152025804	8.51E-06	0.001486558
NM_020234.4	DTWD1	15	49913226	49937337	8.71E-06	0.001505107
NM_004064.2	CDKN1B	12	12867992	12875305	8.73E-06	0.001505107
NM_014444.2	TUBGCP4	15	43663293	43698238	8.79E-06	0.001505107
NM_024531.3	GPR172A	8	145577795	145584932	8.80E-06	0.001505107
NM_021131.3	PPP2R4	9	131873229	131911225	8.89E-06	0.001513495
NM_001042370.1	TROVE2	1	193028552	193060907	8.96E-06	0.001517439
NM_018255.1	ELP2	18	33709864	33757909	9.06E-06	0.001521759
NM_003800.3	RNGTT	6	89319985	89673348	9.10E-06	0.001521759
NM_004453.1	ETFDH	4	159593277	159630775	9.15E-06	0.001521759
NM_001005369.1	MTIF2	2	55463731	55496483	9.20E-06	0.001521759
NM_015954.2	DERA	12	16064106	16190220	9.24E-06	0.001521759

NM_005124.2	NUP153	6	17615269	17706818	9.25E-06	0.001521759
NM_022493.1	NARFL	16	779753	791002	9.36E-06	0.00152257
NM_013232.3	PDCD6	5	271736	353971	9.37E-06	0.00152257
NM_182715.1	SYPL1	7	105730949	105753022	9.40E-06	0.00152257
NM_144998.2	STRA13	17	79976579	79980794	9.43E-06	0.00152257
NM_015375.1	DSTYK	1	205111632	205180727	9.48E-06	0.001523501
NM_177974.1	CASC4	15	44580929	44707956	9.69E-06	0.001548697
NM_032941.1	RECQL	12	21621845	21657515	9.83E-06	0.001564539
NM_030895.1	ZNF696	8	144371846	144380231	1.00E-05	0.001578944
NM_019006.2	ZFAND6	15	80351910	80430735	1.00E-05	0.001578944
NM_005089.3	ZRSR2	23	15808595	15841383	1.02E-05	0.001592581
NM_001040436.1	YARS2	12	32899479	32908874	1.03E-05	0.001592581
NM_005880.2	DNAJA2	16	46989274	47007625	1.03E-05	0.001592581
NM_003858.3	CCNK	14	99947739	99977851	1.04E-05	0.001592581
XM_940962.2	C17orf95	17	74722940	74729961	1.04E-05	0.001592581
NM_207113.1	SLC37A3	7	139993493	140104233	1.04E-05	0.001592581
NM_018106.3	ZDHHC4	7	6617065	6629005	1.07E-05	0.001628145
NM_006304.1	SHFM1	7	96110938	96339203	1.07E-05	0.001629105
NM_016621.2	PHF21A	11	45950871	46142985	1.08E-05	0.001629105
NM_006323.2	SEC24B	4	110354928	110461612	1.10E-05	0.00165765
NM_014347.1	ZNF324	19	58978463	58984765	1.11E-05	0.001665292
NM_031412.2	GABARAPL1	12	10365489	10375722	1.12E-05	0.001665392
NM_015695.2	BRPF3	6	36164521	36200567	1.12E-05	0.001665392
NM_006860.2	IFT27	22	37154246	37172300	1.13E-05	0.001669306
NM_003137.3	SRPK1	6	35800743	35889119	1.13E-05	0.001670574
NM_006999.3	PAPD7	5	6714718	6757161	1.15E-05	0.001683787
NM_001826.1	CKS1B	1	154947129	154951725	1.17E-05	0.001694472
NM_203288.1	RP9	7	33134409	33149013	1.17E-05	0.001694472
NM_004127.4	GPS1	17	80009763	80015346	1.17E-05	0.001694472
NM_001008393.2	C4orf46	4	159587831	159593407	1.18E-05	0.001694472
NM_015571.1	SENP6	6	76311225	76427997	1.18E-05	0.001694472
NM_003129.3	SQLE	8	126010739	126034525	1.18E-05	0.001694472
NM_003481.2	USP5	12	6961285	6975793	1.19E-05	0.001696582
NM_015045.1	WAPAL	10	88195013	88281572	1.20E-05	0.001707743
NM_014394.2	GHITM	10	85899196	85913001	1.23E-05	0.001732778
NM_152260.1	RPUSD2	15	40861499	40866905	1.23E-05	0.001734039
NM_002525.1	NRD1	1	52254863	52344477	1.24E-05	0.001739599
NM_016468.4	COX16	14	70791799	70826448	1.32E-05	0.001849383
NM_007159.2	SLMAP	3	57741177	57914895	1.35E-05	0.001862503
NM_018386.1	PCID2	13	113831891	113863029	1.35E-05	0.001862503
NM_020919.2	ALS2	2	202565277	202645912	1.35E-05	0.001862503
NM_003144.2	SSR1	6	7268539	7347679	1.36E-05	0.001862503
NM_152681.1	TMEM192	4	165997256	166129701	1.38E-05	0.001887546
NM_005088.2	AKAP17A	23	1710486	1721407	1.39E-05	0.001887546
NM_032233.2	SETD3	14	99864083	99947216	1.39E-05	0.001887546
NM_013237.2	PRELID1	5	176730775	176733960	1.40E-05	0.001887546
NM_181466.1	EIF6	20	33866714	33872788	1.41E-05	0.001899763
NM_021709.2	SIVA1	14	105219470	105225990	1.42E-05	0.001899763
NM_021824.2	NIF3L1	2	201754050	201768655	1.42E-05	0.001899763
NM_152434.1	CWF19L2	11	107197071	107328572	1.43E-05	0.001899763
NM_003909.2	CPNE3	8	87497059	87573726	1.45E-05	0.001929747
NM_015260.1	SIN3B	19	16940218	16991164	1.47E-05	0.001935291
NM_014140.2	SMARCAL1	2	217277137	217347776	1.48E-05	0.001935291

NM_001642.1	APLP2	11	129939732	130014699	1.48E-05	0.001935291
NM_001662.2	ARF5	7	127228399	127231759	1.50E-05	0.001955473
NM_005089.1	ZRSR2	23	15808595	15841383	1.51E-05	0.001955473
NM_007198.2	PROSC	8	37620111	37637285	1.51E-05	0.001955473
NM_016065.3	DNAJC9	10	74943120	75008620	1.53E-05	0.001961124
NM_004705.2	PRKRIR	11	76061000	76092015	1.53E-05	0.001961124
NM_016039.1	C14orf166	14	52456193	52471414	1.53E-05	0.001961124
NM_018310.2	BRF2	8	37700786	37707422	1.54E-05	0.001961124
NM_019037.2	EXOSC4	8	145133529	145135550	1.54E-05	0.001961417
NM_004592.2	SFSWAP	12	132195635	132284282	1.57E-05	0.001980981
NM_014765.2	TOMM20	1	235272651	235292251	1.57E-05	0.001980981
NM_017818.2	WDR8	1	3547331	3569325	1.58E-05	0.001991519
NM_001031677.2	RAB24	5	176728199	176730745	1.59E-05	0.001993062
NM_181581.1	DUS4L	7	107203929	107218906	1.60E-05	0.001993062
NM_003129.3	SQLE	8	126010739	126034525	1.61E-05	0.001993062
NM_032725.2	BUD13	11	116618886	116643704	1.61E-05	0.001993062
NM_018024.1	WDYHV1	8	124428965	124479470	1.61E-05	0.001993062
NM_002342.1	LTBR	12	6493334	6500733	1.64E-05	0.002018118
NM_002014.2	FKBP4	12	2904119	2913124	1.67E-05	0.002047177
NM_012398.1	PIP5K1C	19	3630182	3700445	1.68E-05	0.002050743
NM_001545.1	ICT1	17	73008776	73017355	1.71E-05	0.002080747
NM_014947.3	FOXJ3	1	42642210	42801548	1.71E-05	0.002080747
NM_003406.2	YWHAZ	8	101930804	101965616	1.75E-05	0.0021141
NM_001031677.2	RAB24	5	176728199	176730745	1.76E-05	0.002116944
NM_002388.3	MCM3	6	52128807	52149607	1.77E-05	0.002119391
NM_003651.3	CSDA	12	10851676	10875953	1.77E-05	0.002123236
NM_006389.2	HYOU1	11	118914896	118927940	1.79E-05	0.002124087
NM_032547.1	SCOC	4	141178440	141303710	1.79E-05	0.002124087
NM_023078.1	PYCRL	8	144686083	144691943	1.79E-05	0.002124087
NM_001008393.1	C4orf46	4	159587831	159593407	1.81E-05	0.002139432
NM_024699.1	ZFAND1	8	82613569	82645138	1.82E-05	0.002141103
NM_022495.3	C14orf135	14	60558629	60635851	1.83E-05	0.00214441
NM_015942.3	MTERFD1	8	97251626	97273838	1.84E-05	0.00214441
NM_015575.2	GIGYF2	2	233562009	233725285	1.85E-05	0.002154095
NM_014183.2	DYNLRB1	20	33104214	33128762	1.86E-05	0.002154095
NM_015902.4	UBR5	8	103264501	103425069	1.86E-05	0.002155828
NM_030969.2	TMEM14B	6	10747973	10930656	1.91E-05	0.002195401
NM_020933.2	ZNF317	19	9251073	9274089	1.91E-05	0.002195401
NM_152699.4	SENP5	3	196594727	196661585	1.93E-05	0.00221203
NM_024295.3	DERL1	8	124025458	124054663	1.95E-05	0.002219772
NM_017941.3	C17orf80	17	71228776	71245091	2.01E-05	0.002279416
NM_003601.2	SMARCA5	4	144434616	144478639	2.02E-05	0.002279416
NM_003689.2	AKR7A2	1	19630459	19638640	2.03E-05	0.002279416
NM_018045.5	BSDC1	1	32830704	32860332	2.03E-05	0.002279416
NM_001568.2	EIF3E	8	109213445	109447562	2.04E-05	0.002279416
NM_014186.2	COMMD9	11	36295051	36310999	2.04E-05	0.002279416
NM_000837.1	GRINA	8	145064226	145067583	2.05E-05	0.002279416
XM_930460.1	RPL22	1	6241329	6269449	2.06E-05	0.002279416
NM_207327.4	C22orf40	22	46639908	46646576	2.06E-05	0.002279416
NM_004997.2	MYBPH	1	203136939	203144941	2.08E-05	0.002295504
NM_005999.2	TSNAX	1	231664287	231702270	2.09E-05	0.002303577
NM_006197.3	PCM1	8	17780349	17887453	2.10E-05	0.002304181
NM_019843.2	EIF4ENIF1	22	31835349	31892094	2.11E-05	0.002305407

NM_032881.1	LSM10	1	36856839	36863493	2.12E-05	0.002305407
NM_003831.3	RIOK3	18	21032787	21063104	2.13E-05	0.002309092
NM_016319.1	COPS7A	12	6833212	6841026	2.16E-05	0.00234085
NM_174922.3	ADCK5	8	145596790	145618457	2.20E-05	0.002373197
NM_017969.1	IWS1	2	128193783	128284462	2.21E-05	0.002373197
NM_001011671.1	CHCHD7	8	57124245	57131357	2.22E-05	0.002380538
NM_052879.3	LARP4	12	50794650	50873787	2.23E-05	0.002380538
NM_012123.2	MTO1	6	74171301	74218959	2.24E-05	0.002380538
NM_001017369.1	SC4MOL	4	166248775	166264312	2.24E-05	0.002380538
NM_001006.3	RPS3A	4	152020725	152025804	2.25E-05	0.002380748
NM_017921.1	NPLOC4	17	79523915	79604138	2.26E-05	0.002380748
NM_019104.1	LIN37	19	36239499	36245417	2.26E-05	0.002380748
NM_001011885.1	BTBD1	15	83685174	83736106	2.27E-05	0.002380748
NM_030803.5	ATG16L1	2	234118697	234204320	2.27E-05	0.002380748
NM_002067.1	GNA11	19	3094408	3121452	2.28E-05	0.002383094
NM_006116.2	TAB1	22	39795746	39913596	2.29E-05	0.002383094
NM_015705.4	SGSM3	22	40766595	40806122	2.32E-05	0.002408877
NM_014789.1	ZNF623	8	144718183	144738588	2.33E-05	0.002409536
NM_053052.2	SNAP47	1	227916240	227968927	2.36E-05	0.002434506
NM_001419.2	ELAVL1	19	8023458	8070529	2.37E-05	0.002436083
NM_001042635.1	NGDN	14	23924714	23947402	2.42E-05	0.002480026
NM_018407.4	LAPTM4B	8	98787285	98865241	2.44E-05	0.002485635
NM_024321.3	RBM42	19	36119980	36128586	2.45E-05	0.002485635
NM_012067.2	AKR7A3	1	19609052	19615744	2.45E-05	0.002485635
NM_006004.1	UQCRH	1	46769303	46782448	2.46E-05	0.002495529
NM_015356.3	SCRIB	8	144873090	144897549	2.49E-05	0.002504038
NM_017866.4	TMEM70	8	74884672	74895018	2.50E-05	0.002504038
NM_016290.3	UIMC1	5	176332006	176449634	2.50E-05	0.002504038
NM_003470.2	USP7	16	8985951	9057341	2.51E-05	0.002504038
NM_001376.2	DYNC1H1	14	102430865	102517135	2.52E-05	0.002504038
NM_138783.2	ZNF653	19	11594246	11616654	2.52E-05	0.002504038
NM_022063.1	C10orf84	10	120065401	120101840	2.53E-05	0.002512581
NM_021254.1	C21orf59	21	33951132	33985176	2.54E-05	0.002512581
NM_020120.2	UGGT1	2	128848774	128953251	2.57E-05	0.002536193
NM_020745.2	AARS2	6	44267391	44281063	2.59E-05	0.002537532
CR933609	INO80D	2	206858445	206951027	2.59E-05	0.002537532
NM_058182.4	FAM165B	21	35747779	35780164	2.61E-05	0.002547155
NM_001031713.2	CCDC90A	6	13791020	13814800	2.63E-05	0.002559315
NM_014066.3	COMMD5	8	146066427	146079121	2.63E-05	0.002559315
NM_005973.4	PRCC	1	156720402	156770607	2.65E-05	0.00256952
NM_031902.3	MRPS5	2	95752952	95815179	2.66E-05	0.002569538
NM_001077199.1	SREK1	5	65435799	65479443	2.69E-05	0.002584749
NM_213674.1	TPM2	9	35681989	35691017	2.70E-05	0.002584749
NM_001014432.1	AKT1	14	105235689	105262080	2.71E-05	0.002584749
NM_001024227.1	ARF1	1	228270361	228286910	2.71E-05	0.002584749
NM_024710.1	ISOC2	19	55964345	55973049	2.72E-05	0.002584749
NM_014672.2	KIAA0391	14	35591769	35743268	2.72E-05	0.002584749
NM_001035521.1	MPV17	2	27532360	27548547	2.74E-05	0.002593839
NM_014665.1	LRRC14	8	145743376	145750557	2.75E-05	0.002596486
NM_005782.2	THOC4	17	79845717	79849462	2.77E-05	0.00260901
NM_015636.2	EIF2B4	2	27587219	27593353	2.79E-05	0.002622395
NM_032026.2	TATDN1	8	125500726	125551699	2.80E-05	0.002625071
NM_004641.2	MLLT10	10	21823094	22032559	2.81E-05	0.002627013

NM_003169.2	SUPT5H	19	39936186	39967310	2.85E-05	0.002657478
NM_005005.2	NDUFB9	8	125551344	125580751	2.89E-05	0.002686644
NM_007099.2	ACP1	2	264140	278283	2.92E-05	0.002696128
NM_007214.3	SEC63	6	108188960	108279482	2.92E-05	0.002696128
NM_020706.1	SFRS15	21	33043313	33104388	2.93E-05	0.002696128
NM_023080.1	C8orf33	8	146277764	146281416	2.93E-05	0.002696128
NM_002690.1	POLB	8	42195972	42229326	2.97E-05	0.002721462
NM_002107.3	H3F3A	1	226249552	226259702	3.00E-05	0.002745554
NM_003910.2	BUD31	7	99006264	99017239	3.02E-05	0.002758083
NM_001035258.1	RPL38	17	72199721	72206676	3.06E-05	0.00278207
NM_001007565.1	TFG	3	100428205	100467810	3.11E-05	0.002807839
NM_015957.1	APIP	11	34874641	34938046	3.11E-05	0.002807839
NM_002805.4	PSMC5	17	61904810	61909386	3.11E-05	0.002807839
NM_016304.2	RSL24D1	15	55473004	55489265	3.12E-05	0.002813384
NM_023948.4	MOSPD3	7	100209725	100213007	3.15E-05	0.00282535
NM_014754.1	PTDSS1	8	97273943	97349223	3.16E-05	0.002832255
NM_032172.1	USP42	7	6144515	6201195	3.18E-05	0.002835688
NM_000318.2	PEX2	8	77892494	77913280	3.19E-05	0.002835688
NM_012430.3	SEC22A	3	122920774	122992977	3.19E-05	0.002835688
NM_014819.3	PJA2	5	108670410	108745695	3.20E-05	0.002836499
NM_002028.3	FNTB	14	65381140	65529368	3.22E-05	0.002845994
NM_016360.2	TACO1	17	61678243	61685724	3.25E-05	0.002865151
NM_031488.4	L3MBTL2	22	41601209	41627275	3.31E-05	0.002909565
XM_001132711.1	RFNG	17	80005778	80009650	3.32E-05	0.002909565
NM_032687.2	CYHR1	8	145674965	145691060	3.34E-05	0.002916415
NM_024627.5	C22orf29	22	19833661	19842419	3.35E-05	0.002916415
NM_020194.4	MFF	2	228189867	228222550	3.35E-05	0.002916415
NM_002807.2	PSMD1	2	231921578	232037541	3.39E-05	0.002941958
NM_080875.1	MIB2	1	1550795	1565990	3.42E-05	0.00296122
NM_003001.2	SDHC	1	161284047	161332984	3.47E-05	0.002988641
NM_001013839.1	EXOC7	17	74077087	74099856	3.47E-05	0.002988641
NM_006427.2	SIVA1	14	105219470	105225990	3.49E-05	0.003001472
NM_001120.3	MFSD10	4	2932288	2936586	3.53E-05	0.003028202
NM_013326.3	C18orf8	18	21083462	21111746	3.54E-05	0.003028202
NM_004280.3	EEF1E1	6	8015959	8102811	3.56E-05	0.003028202
NM_007269.2	STXBP3	1	109289296	109352148	3.56E-05	0.003028202
NM_020474.2	GALNT1	18	33161003	33291798	3.59E-05	0.003043147
NM_003213.2	TEAD4	12	3068496	3149842	3.59E-05	0.003043147
NM_018145.1	FAM82A2	15	41028082	41048049	3.62E-05	0.003050828
NM_001042401.1	FAM165B	21	35747779	35780164	3.63E-05	0.003050828
NM_025115.1	C8orf41	8	33330904	33371119	3.64E-05	0.003050828
NM_004707.2	ATG12	5	115163897	115177555	3.65E-05	0.003050828
NM_022133.2	SNX16	8	82711816	82755101	3.65E-05	0.003050828
NM_014570.3	ARFGAP3	22	43192508	43267429	3.66E-05	0.003055019
NM_004462.3	FDFT1	8	11659748	11696818	3.69E-05	0.003066306
NM_005760.2	CEBPZ	2	37428755	37458856	3.69E-05	0.003066306
NM_100486.1	WAC	10	28821422	28909928	3.72E-05	0.003081561
NM_014023.3	WDR37	10	1095478	1178237	3.73E-05	0.003081561
NM_003845.1	DYRK4	12	4699486	4723030	3.76E-05	0.003091547
NM_004906.3	WTAP	6	160146617	160177351	3.76E-05	0.003091547
NM_182476.1	COQ6	14	74416643	74429811	3.77E-05	0.003091547
NM_015449.2	C1orf43	1	154179196	154193104	3.81E-05	0.003117391
NM_138501.4	TECR	19	14640382	14676791	3.88E-05	0.003170913

NM_178588.1	PPP2R5C	14	102228222	102394075	3.90E-05	0.003180845
NM_015497.2	TMEM87A	15	42502728	42565755	3.93E-05	0.003190359
NM_177989.2	ACTL6A	3	179280668	179306196	3.97E-05	0.003215548
NM_175573.1	ADRM1	20	60877149	60883918	4.01E-05	0.003242811
NM_014793.3	LCMT2	15	43619976	43622891	4.06E-05	0.003274109
NM_015308.1	FNBP4	11	47738070	47789012	4.08E-05	0.003280365
NM_197964.2	C7orf55	7	139024203	139031065	4.09E-05	0.003280365
NM_024555.3	FBXL6	8	145579091	145583036	4.10E-05	0.003280365
NM_000153.2	GALC	14	88399360	88459907	4.10E-05	0.003280365
NM_020466.4	LYRM2	6	90277863	90348472	4.12E-05	0.003282614
NM_032632.3	PAPOLA	14	96968718	97033425	4.16E-05	0.003302452
NM_001042369.1	TROVE2	1	193028552	193060907	4.16E-05	0.003302452
NM_014175.2	MRPL15	8	55047770	55060461	4.18E-05	0.003308717
NM_001014431.1	AKT1	14	105235689	105262080	4.22E-05	0.00332556
NM_000375.1	UROS	10	127477146	127511817	4.22E-05	0.00332556
NM_003035.2	STIL	1	47715811	47779819	4.23E-05	0.00332556
NM_002707.3	PPM1G	2	27604061	27632554	4.24E-05	0.003328802
NM_033657.1	DAP3	1	155657751	155708803	4.26E-05	0.003330795
NM_014949.2	KIAA0907	1	155882834	155904191	4.26E-05	0.003330795
NM_006756.2	TCEA1	8	54879112	54935089	4.27E-05	0.003330795
NM_006445.3	PRPF8	17	1553923	1588154	4.33E-05	0.003365969
NM_019600.1	KIAA1370	15	52873530	52970820	4.34E-05	0.003365969
NM_018407.4	LAPTM4B	8	98787285	98865241	4.36E-05	0.003370555
NM_024558.2	C14orf138	14	50575350	50583318	4.36E-05	0.003370555
NM_000462.2	UBE3A	15	25582396	25684128	4.39E-05	0.003386864
NM_007192.2	SUPT16H	14	21819631	21852425	4.44E-05	0.003399844
NM_005104.2	BRD2	6	32936437	32949282	4.44E-05	0.003399844
NM_001033714.1	IFFO1	12	6648127	6665237	4.45E-05	0.003399844
NM_014281.3	PUF60	8	144898514	144912029	4.46E-05	0.003399844
NM_003443.1	ZBTB17	1	16268364	16302627	4.46E-05	0.003399844
NM_032205.3	PHF20L1	8	133787618	133861052	4.48E-05	0.003399844
NM_005926.2	MFAP1	15	44096690	44117000	4.49E-05	0.003399844
NM_001005849.1	SUMO2	17	73163825	73179098	4.50E-05	0.003399844
NM_032168.1	WDR75	2	190306159	190340291	4.50E-05	0.003399844
NM_020336.2	RALGAPB	20	37101459	37207504	4.51E-05	0.003399844
NM_020312.1	COQ9	16	57481378	57495178	4.52E-05	0.003399844
NM_000535.4	PMS2	7	6012870	6048756	4.58E-05	0.003438112
NM_016287.3	HP1BP3	1	21069154	21113816	4.59E-05	0.003438224
NM_025234.1	WDR61	15	78575577	78592136	4.60E-05	0.003438535
NM_005255.1	GAK	4	843064	926174	4.68E-05	0.003488722
NM_004869.3	VPS4B	18	61056423	61089693	4.73E-05	0.003517909
NM_005081.2	ZNF142	2	219502639	219524378	4.78E-05	0.003541433
NM_001006.3	RPS3A	4	152020725	152025804	4.78E-05	0.003541433
NM_000666.1	RP11-155D18.11	3	52009066	52023211	4.83E-05	0.003569062
NM_001042462.1	TRAPPC5	19	7745707	7747748	4.84E-05	0.003572998
NM_030939.3	C6orf62	6	24705089	24721064	4.87E-05	0.003581236
NM_152766.2	C17orf61	17	7306294	7307456	4.92E-05	0.00359364
NM_014929.2	FASTKD2	2	207630081	207657233	4.92E-05	0.00359364
NM_001212.3	C1QBP	17	5336099	5342471	4.92E-05	0.00359364
NM_018320.3	RNF121	11	71639747	71708643	4.93E-05	0.00359364
NM_021140.1	KDM6A	23	44732423	44971847	4.95E-05	0.003602383
XM_929318.2	TTC28	22	28374004	29075853	4.99E-05	0.003618459
NM_017455.2	NPTN	15	73852355	73925756	4.99E-05	0.003618459

NM_003350.2	UBE2V2	8	48920960	48976511	5.02E-05	0.003627589
NM_016316.2	REV1	2	100016938	100106497	5.02E-05	0.003627589
NM_175921.4	C5orf51	5	41904290	41921738	5.06E-05	0.00364395
NM_007346.2	OGFR	20	61436187	61445352	5.09E-05	0.003656146
NM_004539.3	NARS	18	55267896	55289177	5.10E-05	0.003656146
NM_001042401.1	FAM165B	21	35747779	35780164	5.12E-05	0.003663456
NM_000319.3	PEX5	12	7341759	7371170	5.19E-05	0.003695811
NM_003288.2	TPD52L2	20	62489087	62522898	5.19E-05	0.003695811
NM_001134493.1	TOMM6	6	41755400	41757636	5.19E-05	0.003695811
NM_001659.1	ARF3	12	49329506	49351310	5.21E-05	0.003695884
NM_001521.2	MPV17	2	27532360	27548547	5.23E-05	0.003701491
NM_206840.1	NVL	1	224415036	224518089	5.24E-05	0.003701491
NM_012319.3	SLC39A6	18	33688495	33709357	5.26E-05	0.003707059
NM_002733.3	PRKAG1	12	49396058	49412592	5.26E-05	0.003707059
NM_019852.3	METTL3	14	21966283	21979457	5.31E-05	0.00372143
NM_006858.2	TMED1	19	10943115	10946983	5.31E-05	0.00372143
NM_004462.3	FDFT1	8	11659748	11696818	5.33E-05	0.003725912
NM_005637.2	SS18	18	23596219	23670611	5.34E-05	0.003725912
NM_032620.1	GTPBP3	19	17448356	17453539	5.43E-05	0.003778084
NM_018266.1	TMEM39A	3	119148347	119187677	5.43E-05	0.003778084
XM_001127807.1	HSF1	8	145515270	145538385	5.46E-05	0.003789249
NM_018088.3	FAM90A1	12	8373866	8377428	5.47E-05	0.003789249
XR_019393.1	DNAJB6	7	157128075	157210133	5.48E-05	0.003789302
NM_199324.1	OTUD4	4	146031990	146101313	5.52E-05	0.003808729
NM_022460.3	HS1BP3	2	20760208	20850864	5.54E-05	0.003816378
NM_016497.2	MRPL51	12	6601318	6602471	5.56E-05	0.003823793
NM_207660.2	ZC3H14	14	89029314	89079852	5.59E-05	0.003834949
NM_014802.1	KIAA0528	12	22601541	22697452	5.61E-05	0.00383677
NM_020701.2	ISY1	3	128806418	128880136	5.63E-05	0.003845348
NM_181469.1	EIF6	20	33866714	33872788	5.75E-05	0.003916558
NM_016372.1	TPRA1	3	127291912	127317094	5.79E-05	0.003937865
NM_012224.1	NEK1	4	170314426	170533780	5.80E-05	0.003939824
NM_012470.2	TNPO3	7	128594948	128695198	5.82E-05	0.00394529
NM_001001654.1	MED8	1	43849588	43855483	5.88E-05	0.003974746
NM_002797.2	PSMB5	14	23495061	23504429	5.92E-05	0.003995038
NM_001001794.2	FAM116B	22	50750392	50765489	5.94E-05	0.003999663
NM_005637.2	SS18	18	23596219	23670611	5.95E-05	0.003999685
NM_203457.1	PPIE	1	40157854	40229586	6.02E-05	0.004040561
NM_003348.3	UBE2N	12	93802090	93836026	6.05E-05	0.0040488
NM_004901.2	ENTPD4	8	23243296	23315208	6.08E-05	0.004049404
NM_001033569.1	AMZ2	17	66244145	66265039	6.08E-05	0.004049404
NM_001625.2	AK2	1	33473585	33502593	6.09E-05	0.004049404
NM_019008.4	TAB1	22	39795746	39913596	6.10E-05	0.004049404
NM_002388.3	MCM3	6	52128807	52149607	6.11E-05	0.004049404
NM_005190.3	CCNC	6	99990256	100016849	6.14E-05	0.00406113
NM_005605.3	PPP3CC	8	22298332	22398652	6.16E-05	0.00406668
NM_016265.3	ZNF12	7	6728064	6746566	6.19E-05	0.004080901
NM_016167.3	NOL7	6	13615559	13632971	6.28E-05	0.004123033
NM_052905.3	FMNL2	2	153191751	153506348	6.28E-05	0.004123033
NM_004329.2	BMPR1A	10	88516396	88684945	6.32E-05	0.004144871
NM_203462.1	MRFAP1L1	4	6709428	6711607	6.36E-05	0.004159347
NM_025241.1	UBXN6	19	4445009	4457694	6.40E-05	0.00418053
NM_032182.3	FAM175B	10	126490354	126525239	6.43E-05	0.004190576

NM_002693.1	POLG	15	89859534	89878092	6.44E-05	0.004190576
NM_018975.2	TERF2IP	16	75681684	75691328	6.48E-05	0.004205548
NM_024323.3	C19orf57	19	13993168	14016909	6.49E-05	0.004207178
NM_019048.1	ASNSD1	2	190526146	190535557	6.55E-05	0.004238785
NM_199298.1	THYN1	11	134118173	134123285	6.61E-05	0.00427003
NM_005154.2	USP8	15	50716579	50793280	6.63E-05	0.004270617
NM_001893.3	CSNK1D	17	80202246	80231573	6.64E-05	0.004272489
NM_016072.3	GOLT1B	12	21654699	21671337	6.66E-05	0.004276864
NM_019070.3	DDX49	19	19030494	19039438	6.68E-05	0.004279424
NM_006427.3	SIVA1	14	105219470	105225990	6.71E-05	0.004280245
NM_032320.5	BTBD10	11	13409548	13484844	6.72E-05	0.004280245
NM_005040.2	PRCP	11	82534544	82681626	6.73E-05	0.004280245
NM_019006.2	ZFAND6	15	80351910	80430735	6.73E-05	0.004280245
NM_024839.1	TRIM39	6	30294256	30314631	6.74E-05	0.004280245
NM_006389.2	HYOU1	11	118914896	118927940	6.78E-05	0.004284806
NM_138465.3	ZFP41	8	144328991	144358573	6.79E-05	0.004284806
NM_002647.2	PIK3C3	18	39535171	39667794	6.79E-05	0.004284806
NM_007166.2	PICALM	11	85668486	85780924	6.80E-05	0.004284808
NM_001031723.1	DNAJB14	4	100817405	100867883	6.81E-05	0.004284808
NM_016286.2	DCXR	17	79993757	79995573	6.84E-05	0.004294285
NM_003601.2	SMARCA5	4	144434616	144478639	6.86E-05	0.004301379
NM_001550.2	IFRD1	7	112063023	112121072	6.90E-05	0.0043139
NM_018141.2	MRPS10	6	42174539	42185603	6.92E-05	0.004322035
NM_176800.1	PRPF4B	6	4021501	4065217	7.00E-05	0.004357864
NM_000714.4	TSPO	22	43547520	43559248	7.00E-05	0.004357864
NM_001003785.1	ATP5H	17	73034955	73043074	7.07E-05	0.004389792
NM_015987.3	HEBP1	12	13127803	13153221	7.13E-05	0.004417901
NM_022745.3	ATPAF1	1	47098409	47139539	7.19E-05	0.004435894
NM_005607.3	PTK2	8	141667999	142012315	7.19E-05	0.004435894
NM_003403.3	YY1	14	100705322	100749129	7.19E-05	0.004435894
NM_001826.1	CKS1B	1	154947129	154951725	7.28E-05	0.004473984
NM_018318.3	CCDC91	12	28410133	28703098	7.29E-05	0.004473984
NM_003184.3	TAF2	8	120743015	120845103	7.30E-05	0.004473984
NM_002033.2	FUT4	11	94277017	94283063	7.34E-05	0.004476458
NM_030974.2	SHARPIN	8	145153536	145163027	7.35E-05	0.004476458
NM_024699.1	ZFAND1	8	82613569	82645138	7.35E-05	0.004476458
NM_015942.3	MTERFD1	8	97251626	97273838	7.36E-05	0.004476458
NM_144566.1	ZNF700	19	12035900	12061588	7.38E-05	0.004476458
NM_001921.2	DCTD	4	183811213	183839089	7.38E-05	0.004476458
NM_005836.2	HRSP12	8	99114572	99129469	7.39E-05	0.004476458
NM_007320.1	RANBP3	19	5916154	5978320	7.42E-05	0.004483476
NR_003545.1	C5orf44	5	64920543	64962060	7.44E-05	0.004487297
NM_199133.2	FAM173B	5	10226442	10250009	7.45E-05	0.004487297
NM_004627.2	WRB	21	40752170	40800454	7.50E-05	0.004512238
NM_030580.2	ZNF34	8	145997611	146012730	7.55E-05	0.004530574
NM_007194.3	CHEK2	22	29083731	29138410	7.67E-05	0.004593864
NM_014384.2	ACAD8	11	134123389	134135749	7.73E-05	0.004625477
NM_001895.3	CSNK2A1	20	459307	524482	7.93E-05	0.004728431
NM_032390.3	MKI67IP	2	122484521	122494499	7.95E-05	0.004731235
XM_936354.2	FAM82B	8	87480486	87526586	8.03E-05	0.004769297
NM_133259.2	LRPPRC	2	44113647	44223144	8.09E-05	0.004795126
NM_000098.1	CPT2	1	53662101	53679869	8.12E-05	0.004805344
NM_001642.1	APLP2	11	129939732	130014699	8.17E-05	0.004827695

NM_016308.1	CMPK1	1	47799469	47844511	8.18E-05	0.004828151
NM_198889.1	ANKRD17	4	73939093	74124515	8.22E-05	0.004839849
NM_024313.2	NOL12	22	38077680	38170137	8.26E-05	0.004855711
NM_002808.3	PSMD2	3	184016497	184026842	8.33E-05	0.004888633
NM_001024688.1	NBN	8	90945564	91015456	8.37E-05	0.004894818
NM_020782.1	KLHDC5	12	27933187	27955972	8.38E-05	0.004894818
NM_014758.1	SNX19	11	130745331	130786404	8.38E-05	0.004894818
NM_001984.1	ESD	13	47345391	47371367	8.41E-05	0.004904497
NM_018250.1	INTS9	8	28625178	28747759	8.50E-05	0.004947173
NM_020905.1	RDH14	2	18735989	18741946	8.54E-05	0.004960854
NM_006628.4	ARPP19	15	52839432	52861213	8.60E-05	0.004990087
NM_138325.2	PCSK6	15	101844134	102030187	8.75E-05	0.0050581
NM_001007553.1	CSDE1	1	115259534	115301297	8.75E-05	0.0050581
NM_002796.2	PSMB4	1	151372010	151374420	8.83E-05	0.005096678
NM_004552.1	NDUFS5	1	39491990	39500308	8.87E-05	0.005099668
NM_012143.2	TFIP11	22	26887191	26908471	8.88E-05	0.005099668
NM_015201.3	BOP1	8	145486055	145515082	8.88E-05	0.005099668
NM_022781.4	RNF38	9	36336393	36487545	8.98E-05	0.00514612
NM_138348.4	FAM105B	5	14664773	14699820	9.03E-05	0.005167778
NM_000154.1	GALK1	17	73754018	73761307	9.05E-05	0.005167778
NM_014043.2	CHMP2B	3	87276421	87304698	9.11E-05	0.005197225
NM_002816.3	PSMD12	17	65334032	65362743	9.14E-05	0.005205897
NM_021633.2	KLHL12	1	202860228	202897764	9.16E-05	0.005208783
NM_133476.3	ZNF384	12	6775643	6798676	9.21E-05	0.00522408
NM_021994.2	ZNF277	7	111846643	111983151	9.26E-05	0.005231813
NM_015528.1	RNF167	17	4843423	4848518	9.27E-05	0.005231813
NM_014877.3	HELZ	17	65066555	65241319	9.27E-05	0.005231813
NM_181573.1	RFC4	3	186507669	186524847	9.28E-05	0.005231813
NM_005302.2	GPR37	7	124386051	124405681	9.34E-05	0.005250445
NM_004431.2	EPHA2	1	16450832	16482582	9.39E-05	0.00525856
NM_003594.3	TTF2	1	117602925	117645492	9.41E-05	0.00525856
NM_022826.2	MARCH7	2	160569000	160625359	9.42E-05	0.00525856
NM_032027.2	TM2D1	1	62146717	62191095	9.44E-05	0.00525856
NM_006156.1	NEDD8	14	24686058	24701660	9.47E-05	0.00525856
NM_018714.2	COG1	17	71189173	71204646	9.49E-05	0.00525856
NM_017746.2	TEX10	9	103064366	103115221	9.50E-05	0.00525856
NM_014041.2	SPCS1	3	52738971	52742182	9.51E-05	0.00525856
NM_080605.3	B3GALT6	1	1167629	1170421	9.51E-05	0.00525856
NM_012062.2	DNM1L	12	32832137	32898584	9.51E-05	0.00525856
NM_014820.3	TOMM70A	3	100082275	100120242	9.53E-05	0.005261118
NM_006233.4	POLR2I	19	36604612	36606206	9.55E-05	0.005261118
NM_003257.3	TJP1	15	29991571	30248497	9.61E-05	0.005289068
NM_001005910.1	IP6K2	3	48725436	48777786	9.63E-05	0.005291751
NM_006348.2	COG5	7	106842189	107204959	9.66E-05	0.005293253
NM_021184.3	C6orf47	6	31626075	31628549	9.67E-05	0.005293253
NM_021826.4	FASTKD5	20	3127165	3140543	9.74E-05	0.005325491
NM_019613.2	WDR45L	17	80572438	80606411	9.79E-05	0.005345635
NM_001035260.1	VPS26A	10	70883268	70932617	9.84E-05	0.005362554
NM_175614.2	NDUFA11	19	5891287	5904025	9.90E-05	0.00538505
NM_004104.4	FASN	17	80036215	80056106	9.92E-05	0.005386604
NM_213605.1	ZNF517	8	146024261	146036554	9.96E-05	0.005402166
NM_024540.2	MRPL24	1	156707095	156711382	0.000100055	0.005417509
NM_013296.3	GPSM2	1	109417972	109473044	0.000100453	0.005424851

NM_024523.5	GCC1	7	127220672	127233665	0.000100577	0.005424851
NM_005830.2	MRPS31	13	41303432	41345309	0.000100666	0.005424851
NM_017759.4	INO80D	2	206858445	206951027	0.00010109	0.005439175
NM_020154.2	C15orf24	15	34376218	34394149	0.000101576	0.005454809
NM_032195.1	SON	21	34914924	34949812	0.0001017	0.005454809
NM_018177.2	N4BP2	4	40058446	40159872	0.00010295	0.005513239
NM_017906.2	PAK1IP1	6	10694928	10710015	0.00010353	0.005535605
NM_145791.1	MGST1	12	16500076	16517343	0.000104272	0.005566494
NM_032340.2	C6orf125	6	33665345	33679504	0.000104433	0.005566494
NM_019020.2	TBC1D16	17	77913821	78009647	0.000104774	0.005575989
NM_001040020.1	FAM3C	7	120988905	121036422	0.000105592	0.005607554
NM_014777.2	URB2	1	229761981	229795946	0.000105695	0.005607554
NM_005648.2	TCEB1	8	74857354	74884522	0.000105942	0.005611986
NM_033271.1	BTBD6	14	105715008	105717430	0.00010773	0.005697875
NM_002263.2	KIFC1	6	33359313	33377701	0.000107992	0.005702915
NM_006221.2	PIN1	19	9945999	9960358	0.00010818	0.00570402
NM_003831.2	RIOK3	18	21032787	21063104	0.000109098	0.005743578
NM_138320.1	PCSK6	15	101844134	102030187	0.000109564	0.00575361
NM_020865.1	DHX36	3	153990335	154042286	0.000109625	0.00575361
NM_033549.3	TRIM41	5	180649499	180662809	0.000110032	0.005766124
NM_024824.3	ZC3H14	14	89029314	89079852	0.000110891	0.005802281
NM_022067.2	VIPAR	14	77893018	77923904	0.000111809	0.00584136
NM_018011.3	ARGLU1	13	107194021	107220512	0.000112213	0.005848687
NM_013336.3	SEC61A1	3	127770484	127790526	0.000112332	0.005848687
NM_005648.2	TCEB1	8	74857354	74884522	0.000112462	0.005848687
NM_020680.3	SCYL1	11	65292548	65306175	0.000112927	0.005863992
NM_000918.3	P4HB	17	79801037	79818545	0.000114843	0.005954413
NM_015013.2	KDM1A	1	23345941	23410182	0.000115115	0.005959529
NM_017421.3	COQ3	6	99817276	99842080	0.000115804	0.00598614
NM_012295.2	CABIN1	22	24407642	24574596	0.000116074	0.005991042
NM_004713.3	SDCCAG1	14	50249997	50319506	0.000116427	0.006000206
NM_018285.2	IMP3	15	75931426	75941047	0.000118357	0.006083877
NM_006465.2	ARID3B	15	74833546	74890472	0.000118406	0.006083877
NM_005587.1	MEF2A	15	100106133	100256627	0.000119024	0.006106443
NM_198155.1	C21orf33	21	45553487	45565605	0.000119534	0.006123434
NM_015344.1	LEPROTL1	8	29952914	30034724	0.000119987	0.006137213
NM_002494.2	NDUFC1	4	140188034	140223705	0.000120222	0.006137213
XM_001126750.1	ABI2	2	204192942	204301606	0.000120341	0.006137213
NM_030810.2	TXNDC5	6	7881750	7911047	0.000121007	0.006153954
NM_020461.3	TUBGCP6	22	50656118	50683421	0.000121048	0.006153954
NM_004800.1	TM9SF2	13	100153671	100215645	0.000121209	0.006153954
NM_003756.2	EIF3H	8	117654369	117779164	0.000122741	0.006204287
NM_004634.2	BRPF1	3	9773413	9789702	0.000123054	0.006204287
NM_018056.1	TMEM39B	1	32537632	32568467	0.000123114	0.006204287
NM_005154.2	USP8	15	50716579	50793280	0.000123142	0.006204287
NM_024095.3	ASB8	12	48541574	48551377	0.00012323	0.006204287
NM_001040439.1	MAPK8IP3	16	1756194	1820318	0.000123288	0.006204287
NM_024419.3	PGS1	17	76374735	76420635	0.000123651	0.006213427
NM_001040876.1	ABCE1	4	146019084	146050676	0.000123968	0.006216972
NM_006310.2	NPEPPS	17	45600306	45700642	0.000124086	0.006216972
NM_001012271.1	BIRC5	17	76210277	76221715	0.000124267	0.006216972
NM_000019.2	ACAT1	11	107992243	108018503	0.000124522	0.006220627
NM_005857.3	ZMPSTE24	1	40723779	40759856	0.000124858	0.006226626

NM_002865.1	RAB2A	8	61429416	61536186	0.000125006	0.006226626
NM_020983.2	ADCY6	12	49159975	49177877	0.000125526	0.006243437
AK056642	ATXN7L3B	12	74931607	74935232	0.000126047	0.006260283
NM_014517.3	UBP1	3	33429828	33482863	0.000127075	0.006302164
NM_145729.1	MRPL24	1	156707095	156711382	0.000127696	0.006323815
NM_006353.2	HMGN4	6	26538572	26547160	0.000128561	0.006357465
NM_001168.2	BIRC5	17	76210277	76221715	0.000128783	0.00635923
NM_001002836.2	ZNF787	19	56598732	56632649	0.00012925	0.006373115
NM_018981.1	DNAJC10	2	183580999	183644334	0.000129827	0.006392324
NM_201443.1	TEAD4	12	3068496	3149842	0.000130134	0.00639826
NM_015029.1	POP1	8	99129525	99172062	0.000130371	0.006399857
NM_003779.2	B4GALT3	1	161141100	161147314	0.000130626	0.006399857
NM_024325.4	ZNF343	20	2447858	2505348	0.000130728	0.006399857
NM_002268.3	KPNA4	3	160212783	160283376	0.000131241	0.00641582
NM_025203.1	C2orf44	2	24252210	24272445	0.00013253	0.006469584
NM_018115.2	SDAD1	4	76862103	76912115	0.000132846	0.006470547
NM_016454.2	TMEM85	15	34517200	34522357	0.000132928	0.006470547
NM_006493.1	CLN5	13	77564795	77576652	0.000133346	0.006474211
NM_004712.3	HGS	17	79651020	79669149	0.000133426	0.006474211
NM_004127.4	GPS1	17	80009763	80015346	0.000133571	0.006474211
NM_016308.1	CMPK1	1	47799469	47844511	0.000134296	0.00649686
NM_025054.3	VCPIP1	8	67540722	67579452	0.000134418	0.00649686
NM_005077.3	TLE1	9	84198598	84304220	0.000135572	0.006543396
NM_020213.1	PARP6	15	72533522	72563628	0.000135884	0.006549228
NM_080649.1	APEX1	14	20923290	20925933	0.00013773	0.006628849
NM_001098.2	ACO2	22	41865129	41924993	0.000138198	0.006636845
NM_021253.2	TRIM39	6	30294256	30314631	0.000138446	0.006636845
NM_016176.2	SDF4	1	1152288	1167411	0.000138534	0.006636845
NM_178819.2	AGPAT6	8	41434706	41482520	0.000138837	0.006636845
NM_018023.3	YEATS2	3	183415606	183530413	0.000138866	0.006636845
NM_199229.1	RPE	2	210867289	210886300	0.000139744	0.006665395
NM_006659.2	TUBGCP2	10	135093135	135125841	0.000139937	0.006665395
NM_021709.1	SIVA1	14	105219470	105225990	0.000140047	0.006665395
NM_014173.2	C19orf62	19	17378232	17390162	0.000140672	0.006685817
NM_005414.2	SKIL	3	170075466	170111651	0.000141236	0.006703303
NM_004855.4	PIGB	15	55611133	55647845	0.000141986	0.006727816
BC004287	ZNF704	8	81540686	81787016	0.000142145	0.006727816
NM_138323.1	PCSK6	15	101844134	102030187	0.000142799	0.006749434
NM_014691.2	AQR	15	35147750	35261956	0.000143338	0.006765552
NM_014628.2	MAD2L1BP	6	43597277	43608689	0.000143818	0.00677884
NM_020940.3	FAM160B1	10	116581503	116659591	0.000144386	0.006796289
NM_178136.1	POLDIP3	22	42979727	43010968	0.000145523	0.006817243
NM_001003694.1	BRPF1	3	9773413	9789702	0.000145533	0.006817243
NM_001040614.1	LINS	15	101107119	101142445	0.00014562	0.006817243
NM_198527.2	HDDC3	15	91474148	91475799	0.000145628	0.006817243
NM_015565.1	LTN1	21	30300466	30365270	0.000146427	0.006845241
NM_020347.2	LZTFL1	3	45864808	45957534	0.00014727	0.006852496
NM_032276.2	RHBDD1	2	227700297	227863931	0.000147298	0.006852496
NM_015197.1	PACS2	14	105767171	105864484	0.000147425	0.006852496
NM_031307.2	PUS3	11	125763381	125773116	0.000147679	0.006852496
NM_002451.3	MTAP	9	21802635	22032985	0.000147754	0.006852496
NM_006392.2	NOP56	20	2632791	2639039	0.000147783	0.006852496
NM_022730.1	COPS7B	2	232646381	232674093	0.000148307	0.006864887

NM_004782.2	SNAP29	22	21213271	21245502	0.000148452	0.006864887
NM_015965.4	NDUFA13	19	19626540	19646885	0.000148726	0.006868291
NM_022495.5	C14orf135	14	60558629	60635851	0.00014933	0.006886884
NM_032026.1	TATDN1	8	125500726	125551699	0.000149783	0.006894649
NM_015024.2	XPO7	8	21777180	21864096	0.000149901	0.006894649
NM_001079514.1	UBN1	16	4897912	4932361	0.000150704	0.006922274
NM_138428.3	C1orf212	1	35178338	35325417	0.000152328	0.006985978
NM_001940.3	ATN1	12	7033626	7051482	0.000152499	0.006985978
NM_005561.2	LAMP1	13	113951556	113977987	0.00015277	0.006989018
NM_015092.3	SMG1	16	18816175	18937776	0.000154065	0.007038859
NM_004930.1	CAPZB	1	19665270	19812033	0.000156829	0.007155598
NM_001204.5	BMPR2	2	203241580	203432474	0.000157346	0.007169608
NM_032509.2	MAK16	8	33342268	33358687	0.00015769	0.007171155
NM_018067.2	MAP7D1	1	36621180	36646450	0.000157899	0.007171155
NM_015375.2	DSTYK	1	205111632	205180727	0.000158009	0.007171155
NM_000108.3	DLD	7	107531415	107572175	0.000158726	0.007185418
NM_173797.2	PAPD4	5	78907943	78982471	0.000158743	0.007185418
NM_003795.3	SNX3	6	108532426	108582464	0.000159127	0.007193296
NM_015636.3	EIF2B4	2	27587219	27593353	0.000159364	0.007194496
NM_130781.1	RAB24	5	176728199	176730745	0.000160385	0.007219323
NM_197964.3	C7orf55	7	139024203	139031065	0.000160482	0.007219323
NM_024052.4	C17orf39	17	17942736	17971718	0.000160547	0.007219323
NM_002887.3	RARS	5	167913450	167946304	0.000160895	0.007221817
NM_001037872.1	REV1	2	100016938	100106497	0.000161098	0.007221817
NM_018098.4	ECT2	3	172468472	172539264	0.000161235	0.007221817
NM_024874.3	KIAA0319L	1	35899091	36023551	0.000161843	0.007239554
NM_181354.3	OXR1	8	107282473	107764922	0.000162318	0.00725134
NM_005626.3	SRSF4	1	29474255	29508499	0.000162556	0.0072525
NM_145806.2	ZNF511	10	135121979	135126666	0.000162888	0.007257852
NM_003093.1	SNRPC	6	34725183	34741571	0.000163825	0.007284063
NM_000462.2	UBE3A	15	25582396	25684128	0.000163902	0.007284063
NM_138797.1	ANKRD54	22	38226862	38240438	0.000164767	0.007312998
NM_017743.4	DPP8	15	65734801	65810042	0.00016522	0.00732361
NM_001040057.1	FAM133B	7	92190107	92219708	0.000166507	0.007354427
NM_000987.3	RPL26	17	8280838	8286568	0.000166569	0.007354427
CN291296	GALNT2	1	230193737	230417870	0.000166747	0.007354427
NM_007342.1	NUPL2	7	23221446	23240630	0.000166775	0.007354427
NM_007126.2	VCP	9	35056061	35073246	0.000168137	0.007398321
NM_016547.1	SDF4	1	1152288	1167411	0.000168203	0.007398321
NM_020680.2	SCYL1	11	65292548	65306175	0.000168874	0.007415318
NM_199462.2	DSTYK	1	205111632	205180727	0.000169022	0.007415318
NM_016145.2	C19orf56	19	12778881	12780465	0.000170354	0.007455324
NM_001625.2	AK2	1	33473585	33502593	0.000170649	0.007455324
NM_032926.2	TCEAL3	23	102862379	102884618	0.000170746	0.007455324
NM_199483.1	C20orf24	20	35234137	35240960	0.000170806	0.007455324
NM_006441.1	MTHFS	15	80137318	80216096	0.000171558	0.007472598
NM_015388.2	YIPF3	6	43479565	43484728	0.000171638	0.007472598
NM_017865.2	ZNF692	1	249144205	249153343	0.000172591	0.007488738
NM_199361.1	TPD52L2	20	62489087	62522898	0.000172592	0.007488738
NM_032549.2	IMMP2L	7	110303110	111202573	0.000172666	0.007488738
NM_001079673.1	FNDC3A	13	49550048	49783888	0.000173028	0.007494945
NM_018066.2	GPN2	1	27205773	27216788	0.000173616	0.007502378
NM_001024463.2	PPIP5K1	15	43825660	43982262	0.000173638	0.007502378

NM_017859.2	UCKL1	20	62571186	62587769	0.0001753	0.007564641
NM_024662.1	NAT10	11	34127111	34169217	0.000175833	0.00757811
NM_015325.1	KIAA0947	5	5420777	5490347	0.000176095	0.007579822
NM_001017391.1	SULT1A3	16	30205743	30215649	0.000176692	0.007595999
NM_173469.1	UBE2Q2	15	76135622	76193419	0.000177822	0.007634963
NM_005861.2	STUB1	16	730276	732753	0.000178049	0.007635122
NM_015231.1	NUP160	11	47799639	47870107	0.000179154	0.007672898
NM_017553.1	INO80	15	41271078	41408444	0.000180655	0.007720424
NM_032864.3	PRPF38A	1	52870236	52883992	0.000180715	0.007720424
NM_000310.2	PPT1	1	40538379	40563375	0.000181106	0.00772749
NM_024708.2	ASB7	15	101142739	101191910	0.000181712	0.007734359
NM_030649.1	ACAP3	1	1227756	1244989	0.000181979	0.007734359
NM_003218.2	TERF1	8	73921099	73960357	0.00018212	0.007734359
NM_001014840.1	CUTA	6	33384219	33386094	0.000182171	0.007734359
NM_031466.4	TRAPPC9	8	140742586	141468678	0.000182908	0.007756026
NM_024120.3	C20orf7	20	13765596	13799067	0.000183937	0.007781691
NM_005271.1	GLUD1	10	88810243	88854623	0.000183968	0.007781691
NM_182931.2	MLL5	7	104654626	104754808	0.000184209	0.007782278
NM_021188.1	ZNF410	14	74353574	74398980	0.000185116	0.007810924
NM_004517.2	ILK	11	6624961	6632102	0.00018538	0.007812456
NM_032377.3	ELOF1	19	11663858	11670051	0.000187708	0.007900836
NM_004053.3	BYSL	6	41888926	41900784	0.000188059	0.007904288
NM_004326.2	BCL9	1	147013182	147098017	0.000188252	0.007904288
NM_057161.2	KLHDC3	6	42981951	42989036	0.000188758	0.007912708
NM_138364.2	PRMT10	4	148558936	148605381	0.000188915	0.007912708
NM_213619.1	ATP6V1H	8	54628117	54756118	0.000189167	0.007913578
NM_080651.1	MED30	8	118532952	118552501	0.000190947	0.00797829
NM_001320.5	CSNK2B	6	31633013	31641323	0.000191362	0.007983912
NM_004046.4	ATP5A1	18	43664110	43684300	0.000191549	0.007983912
NM_199297.1	THYN1	11	134118173	134123285	0.000193258	0.008045362
NM_015446.3	AHCTF1	1	247002400	247095280	0.000193971	0.008064086
NM_006242.3	PPP1R3D	20	58511894	58515352	0.000194281	0.008064086
NM_152485.2	C1orf74	1	209955661	209957904	0.000194415	0.008064086
NM_152835.2	PDIK1L	1	26437664	26452034	0.000195201	0.008077937
NM_015957.1	APIP	11	34874641	34938046	0.000195221	0.008077937
NM_003718.3	CDK13	7	39989636	40136733	0.000195566	0.008082451
NM_152906.3	C22orf25	22	20004537	20053449	0.000195861	0.008084887
NM_014171.3	CRIPT	2	46843555	46852881	0.000196366	0.008095962
NM_017736.3	THUMPD1	16	20744986	20753406	0.000197103	0.008103182
NM_006828.2	ASCC3	6	100956070	101329248	0.000197155	0.008103182
NM_018471.2	ZC3H15	2	187350883	187374090	0.000197252	0.008103182
NM_032015.3	RNF26	11	119205237	119208021	0.000198079	0.008127399
NM_006586.3	CNPY3	6	42896938	42910989	0.00019887	0.008150117
NM_002035.1	KDSR	18	60994971	61034743	0.000199589	0.008169778
NM_015635.2	GAPVD1	9	128024073	128129486	0.000199991	0.00817553
NM_023039.2	ANKRA2	5	72848160	72861511	0.000200231	0.00817553
NM_016488.5	PPHLN1	12	42719947	42842422	0.000200446	0.00817553
NM_024596.2	MCPH1	8	6264113	6506026	0.000201458	0.008199577
NM_024685.3	BBS10	12	76738254	76742207	0.00020164	0.008199577
NM_001048197.1	RCC1	1	28832496	28865607	0.000202102	0.008199577
XM_936354.2	FAM82B	8	87480486	87526586	0.000202187	0.008199577
NM_002804.4	PSMC3	11	47440320	47447993	0.000202234	0.008199577
NM_014413.2	EIF2AK1	7	6061881	6098861	0.00020253	0.008201879

NM_198486.2	RPL7L1	6	42847356	42857663	0.000205101	0.008287222
NM_018447.1	TMEM111	3	10004221	10052800	0.000205122	0.008287222
NM_032683.2	AC068499.1	19	18304028	18307552	0.000205383	0.00828798
NM_000303.1	PMM2	16	8891674	8943188	0.000205998	0.008303017
NM_001003796.1	NHP2L1	22	42069934	42086508	0.00020651	0.008313865
NM_032796.2	SYAP1	23	16737755	16783459	0.000207336	0.008337307
XM_034819.6	ZNF629	16	30789770	30798523	0.00020838	0.008353267
NM_001806.2	CEBPG	19	33864609	33873591	0.000208593	0.008353267
NM_000255.1	MUT	6	49398073	49430904	0.000208602	0.008353267
NM_021974.2	POLR2F	22	38348614	38437113	0.00020871	0.008353267
NM_016397.2	TH1L	20	57556296	57570188	0.000209095	0.008355457
NM_003473.2	STAM	10	17686124	17757913	0.00020933	0.008355457
NM_001007277.1	EI24	11	125439112	125454575	0.000209568	0.008355457
NM_138807.2	C3orf31	3	11831916	11888393	0.000209844	0.008355457
NM_012331.2	MSRA	8	9911778	10286401	0.000209985	0.008355457
NM_020412.3	CHMP1B	18	11851439	11854448	0.000210515	0.008366811
NM_018247.2	TMEM30A	6	75962640	75994684	0.000210805	0.008368632
NM_016630.3	SPG21	15	65255362	65282274	0.000211356	0.008380759
NM_134447.1	C19orf2	19	30433158	30506611	0.0002125	0.008397533
NM_001038.4	SCNN1A	12	6456015	6484715	0.000212653	0.008397533
NM_001008896.1	C1orf213	1	23695490	23698332	0.000212665	0.008397533
NM_001076677.1	CLTA	9	36190892	36304778	0.00021276	0.008397533
NM_013354.5	CNOT7	8	17086737	17104387	0.000214394	0.008452273
NM_194326.2	RPS19BP1	22	39925098	39928860	0.000214923	0.008463356
NM_018418.2	SPATA7	14	88852011	88904802	0.000215995	0.008495814
NM_032410.2	HOOK3	8	42752075	42883255	0.000216524	0.008506833
NM_145331.1	MAP3K7	6	91223292	91296764	0.000217548	0.008537262
NM_015926.3	TEX264	3	51696709	51738339	0.000218429	0.008558062
NM_014693.2	ECE2	3	183967438	184010819	0.000218578	0.008558062
NM_177924.2	ASAH1	8	17913925	17942494	0.000219879	0.008599167
NM_015937.3	PIGT	20	44044717	44054884	0.000220339	0.008600663
NM_001021.3	RPS17	15	82821158	82824972	0.00022042	0.008600663
NM_031488.4	L3MBTL2	22	41601209	41627275	0.000221052	0.008613184
NM_198896.1	RAB6A	11	73386683	73472166	0.000221245	0.008613184
NM_000859.1	HMGCR	5	74632154	74657929	0.000221622	0.008618066
NM_004766.1	COPB2	3	139074442	139108574	0.000222202	0.008630813
NM_145863.1	ASB3	2	53759810	54087297	0.00022296	0.008650459
NM_001024807.1	APLP1	19	36359401	36370699	0.000223484	0.008660955
NM_018416.2	FOXJ2	12	8185359	8208117	0.000224072	0.00866405
NM_004130.2	GYG1	3	148709128	148745419	0.000224323	0.00866405
NM_015176.1	FBXO28	1	224301789	224349749	0.000224594	0.008664706
NM_180976.1	PPP2R5D	6	42952237	42980080	0.000225449	0.008687903
NM_006947.3	SRP72	4	57333081	57369839	0.000225973	0.008697517
NM_001011515.1	PDLIM5	4	95373037	95589377	0.000226207	0.008697517
NM_001001651.1	MED8	1	43849588	43855483	0.000226876	0.00871347
NM_205861.1	DHDDS	1	26758773	26797793	0.000227344	0.008716535
NM_005768.5	LPCAT3	12	7085347	7125842	0.000227465	0.008716535
NM_005087.2	FXR1	3	180585929	180694950	0.000229215	0.008773759
NM_003952.2	RPS6KB2	11	67195931	67202872	0.000230127	0.008795754
NM_006330.2	LYPLA1	8	54958938	55014577	0.000230304	0.008795754
NM_182686.1	KIAA0319L	1	35899091	36023551	0.000232393	0.008857882
NM_001206.2	KLF9	9	72999503	73029540	0.000232659	0.008857882
NM_012162.1	FBXL6	8	145579091	145583036	0.000232707	0.008857882

NM_016128.3	COPG	3	128968449	128996614	0.000234335	0.008909932
NM_015001.2	SPEN	1	16174359	16266955	0.000235457	0.008942673
NM_138320.1	PCSK6	15	101844134	102030187	0.000238176	0.009021929
NM_004814.1	SNRNP40	1	31732417	31769629	0.000238208	0.009021929
NM_002532.3	NUP88	17	5289346	5323000	0.000238572	0.009021929
NM_144600.2	C16orf63	16	15959576	15982472	0.000238599	0.009021929
NM_152318.2	C12orf45	12	105380098	105388505	0.000239075	0.009029954
NM_003626.2	PPFIA1	11	70116806	70230509	0.000239379	0.009031489
NM_032758.3	PHF5A	22	41855721	41864729	0.000240513	0.009058977
NM_001078645.1	CDC16	13	115000362	115038198	0.000240637	0.009058977
NM_015654.3	NAT9	17	72766688	72772470	0.000241672	0.009087936
NM_001037633.1	SIL1	5	138282409	138629246	0.000241958	0.009088708
NM_025133.3	FBXO11	2	48016455	48132932	0.000242485	0.009095524
NM_020189.4	ENY2	8	110346553	110358182	0.000242671	0.009095524
NM_025180.3	CEP63	3	134204585	134293859	0.000243138	0.009095674
XM_001726504.1	AC008763.1	19	7694693	7696475	0.000243207	0.009095674
NM_025147.3	COQ10B	2	198318147	198340032	0.000243806	0.009108114
NM_024831.5	TGS1	8	56685701	56738007	0.000244674	0.0091306
NM_001080510.2	C17orf95	17	74722940	74729961	0.000244947	0.009130815
NM_033661.3	WDR4	21	44263204	44299678	0.000245442	0.009139322
NM_002957.3	RXRA	9	137208944	137332431	0.000247017	0.009179905
NM_016047.3	AC008073.5	2	24290454	24299313	0.000247069	0.009179905
NM_014671.1	UBE3C	7	156931607	157062066	0.000250066	0.009267339
NM_152912.3	MTIF3	13	28009780	28024728	0.000250105	0.009267339
NM_015102.2	NPHP4	1	5922871	6052533	0.000250234	0.009267339
NM_152328.3	ADSSL1	14	105190534	105213645	0.000250777	0.009272578
NM_153358.1	ZNF791	19	12721732	12742735	0.00025104	0.009272578
NM_007109.1	TCF19	6	31126319	31131992	0.000251189	0.009272578
NM_080597.2	OSBPL1A	18	21742008	21977823	0.000251856	0.009287177
NM_015455.3	CNOT6	5	179921412	180005405	0.000253331	0.00933151
NM_144563.2	RPIA	2	88991162	89050427	0.000254002	0.009342551
NM_180989.4	GPR180	13	95254157	95279698	0.00025458	0.009342551
NM_005147.3	DNAJA3	16	4475806	4506776	0.000254616	0.009342551
NM_144963.2	FAM91A1	8	124780696	124827692	0.000254722	0.009342551
NM_001009551.1	CNIH	14	54893646	54908149	0.000255165	0.009348776
NM_006129.2	BMP1	8	22022249	22069839	0.000257857	0.0094373
NM_013253.4	DKK3	11	11984547	12031316	0.000258322	0.009444196
NM_015690.2	STK36	2	219536749	219567439	0.000259134	0.009463772
NM_031903.1	MRPL32	7	42971799	42988557	0.000260539	0.009500923
NM_152477.3	ZNF565	19	36673188	36705986	0.000262154	0.009543513
XM_001128799.1	MAP3K2	2	128056306	128146041	0.00026257	0.009548511
NM_015511.3	C20orf4	20	34824381	34858840	0.000263362	0.009567138
NM_014874.2	MFN2	1	12040238	12073571	0.000264155	0.009585756
NM_016080.2	GLOD4	17	662550	686505	0.000265432	0.009621925
NM_017580.2	ZRANB1	10	126630692	126676758	0.000266268	0.009641995
NM_022740.2	HIPK2	7	139246316	139477577	0.00026657	0.009642742
NM_031490.2	LONP2	16	48278207	48389415	0.000267426	0.009650587
NM_016222.2	DDX41	5	176938578	176944470	0.00026758	0.009650587
NM_003003.2	SEC14L1	17	75137005	75213183	0.000267633	0.009650587
NM_007079.2	PTP4A3	8	142402093	142441620	0.000268472	0.009663659
NM_018840.2	C20orf24	20	35234137	35240960	0.000268734	0.009663659
NM_005966.3	NAB1	2	191511472	191557492	0.000268843	0.009663659
NM_080687.1	UPF3A	13	115047059	115071283	0.000271639	0.009753904

NM_001098615.1	PUS7L	12	44122416	44152596	0.000272469	0.009773457
NM_033363.1	MRPS12	19	39421348	39423657	0.000273063	0.009784504
NM_022761.2	C11orf1	11	111749659	111756699	0.000273651	0.009792982
NM_004162.3	RAB5A	3	19988571	20026667	0.000273888	0.009792982
U56251	TXLNG	23	16804550	16862642	0.000274158	0.009792982
NM_198244.1	EIF4G1	3	184032283	184053146	0.000274686	0.009798408
NM_001040428.2	SPATA7	14	88852011	88904802	0.000275054	0.009798408
NM_003366.2	UQCRC2	16	21963981	21994981	0.000275169	0.009798408
NM_001007027.2	ALG8	11	77811982	77850706	0.000276186	0.00982236
NM_001081640.1	AC103686.1	8	48685669	48872743	0.000276532	0.00982236
NM_002078.3	GOLGA4	3	37284668	37408242	0.000276702	0.00982236
NM_001098533.1	CDK10	16	89753078	89762772	0.000277669	0.009846445
NM_175874.2	C12orf60	12	14956506	15059520	0.000278393	0.009860926
NM_133375.2	DIS3L	15	66585555	66626236	0.000278874	0.009860926
NM_003861.1	DCAF5	14	69517638	69619914	0.000278941	0.009860926
NM_015609.2	C1orf144	1	16679070	16724640	0.000279635	0.009869978
NM_033643.2	RPL36	19	5690272	5691674	0.000279774	0.009869978
NM_016030.5	TTC15	2	3383446	3488865	0.000280396	0.009872981
NM_138998.1	DDX39	19	14519633	14530171	0.000280437	0.009872981
NM_145012.3	CCNY	10	35535953	35860852	0.00028301	0.009950446
NM_018979.2	WNK1	12	862089	1020618	0.000283218	0.009950446
NM_005724.4	TSPAN3	15	77336359	77363570	0.000284228	0.009975668
NM_003634.1	NIPSNAP1	22	29950797	29977326	0.00028499	0.00999216
NM_024313.1	NOL12	22	38077680	38170137	0.000286534	0.010036022
NM_016166.1	PIAS1	15	68346572	68480402	0.000287868	0.01007107
NM_014584.1	ERO1L	14	53106634	53162432	0.000288333	0.01007107
NM_024647.4	NUP43	6	150045451	150070801	0.000288463	0.01007107
NM_018075.3	ANO10	3	43396351	43733086	0.000289657	0.010093738
NM_173811.2	HARBI1	11	46624411	46639459	0.000290549	0.010114523
NM_004450.1	ERH	14	69846840	69865021	0.000293571	0.010209353
NM_033168.2	B3GALNT1	3	160801671	160823172	0.000294275	0.010217459
NM_031267.1	CDK13	7	39989636	40136733	0.000294628	0.010217459
NM_003194.3	TBP	6	170863390	170881944	0.00029501	0.010217459
NM_001951.3	E2F5	8	86089460	86129387	0.000295085	0.010217459
NM_015713.3	RRM2B	8	103216730	103251346	0.000295297	0.010217459
NM_018164.1	C12orf11	12	27058118	27091254	0.000296325	0.010242669
NM_173348.1	FAM149B1	10	74927877	75004262	0.000296843	0.010243594
NM_004546.2	NDUFB2	7	140390577	140422590	0.00029695	0.010243594
NM_025161.3	C17orf70	17	79506912	79519426	0.000297734	0.010258698
NM_020378.2	NAT14	19	55996594	55998935	0.000297988	0.010258698
NM_013254.2	TBK1	12	64845900	64895886	0.00030013	0.010322048
NM_004402.2	DFFB	1	3773845	3801993	0.000300478	0.010323648
NM_015902.4	UBR5	8	103264501	103425069	0.000301353	0.010339551
NM_017660.2	GATAD2A	19	19496639	19619740	0.000301545	0.010339551
NM_018256.2	WDR12	2	203744993	203879521	0.000302929	0.010367048
NM_016026.2	RDH11	14	68143521	68162510	0.000302953	0.010367048
NM_004309.3	ARHGDIA	17	79825598	79829238	0.000303469	0.010374339
XM_001130368.1	TOP1MT	8	144386554	144442149	0.000304404	0.010395902
NM_005485.3	PARP3	3	51976361	51982883	0.00030549	0.010422608
NM_016252.3	BIRC6	2	32582096	32843966	0.0003071	0.010467097
NM_014315.2	KLHDC2	14	50234326	50249909	0.000307644	0.010469018
NM_005793.3	NME6	3	48334754	48343175	0.000308332	0.010469018
AK124143	LNPEP	5	96271098	96373219	0.000308377	0.010469018

NM_152415.1	VPS37A	8	17104080	17159936	0.000308542	0.010469018
XM_096885.9	C15orf61	15	67813522	67819639	0.000308686	0.010469018
NM_014639.2	TTC37	5	94799599	94890711	0.000309026	0.010470177
NM_018170.2	RPRD1A	18	33569787	33647539	0.000311809	0.010554
NM_203456.1	PPIE	1	40157854	40229586	0.000312791	0.010576788
NM_016018.4	PHF20L1	8	133787618	133861052	0.00031358	0.010583089
NM_005239.4	ETS2	21	40177231	40196879	0.000313596	0.010583089
NM_006899.2	IDH3B	20	2639041	2644865	0.000314878	0.010615886
NM_133172.2	SRA1	5	139929752	139937895	0.000315673	0.010632203
NM_053053.2	TADA1	1	166825747	166845564	0.000317021	0.010644036
NM_007222.3	ZHX1	8	124260697	124287781	0.000317342	0.010644036
NM_174889.3	NDUFAF2	5	60240956	60448853	0.000317557	0.010644036
NM_018170.3	RPRD1A	18	33569787	33647539	0.000317607	0.010644036
NM_030805.2	LMAN2L	2	97371666	97405801	0.000318209	0.010644036
NM_003166.3	SULT1A3	16	30205743	30215649	0.000318276	0.010644036
NR_002315.1	H3F3A	1	226249552	226259702	0.000318423	0.010644036
NM_001039367.1	ATP6V1E1	22	18074902	18111584	0.000318547	0.010644036
NM_001013399.1	CCNC	6	99990256	100016849	0.000319051	0.010644036
NM_003093.1	SNRPC	6	34725183	34741571	0.000319134	0.010644036
NM_002013.2	FKBP3	14	45584803	45604522	0.000320049	0.010664145
NM_015999.2	ADIPOR1	1	202909951	202927700	0.000321815	0.010712558
NM_003316.3	TTC3	21	38445526	38575413	0.000322207	0.010713126
NM_020133.2	AGPAT4	6	161551057	161695093	0.000322458	0.010713126
NM_024545.2	SAP130	2	128698792	128785694	0.000323139	0.010722506
NM_020313.2	CIAPIN1	16	57462081	57481439	0.000323367	0.010722506
NM_015214.1	DDHD2	8	38082736	38133076	0.000323892	0.010729508
NM_019059.2	TOMM7	7	22850011	22862470	0.000324737	0.01074711
NM_134324.2	TARBP2	12	53894705	53900213	0.000326048	0.010767052
NM_205843.1	NFIC	19	3359616	3463603	0.000326214	0.010767052
NM_022165.2	LIN7B	19	49617581	49621717	0.000326733	0.010767052
XR_016048.1	AC132812.1	17	62745781	62758831	0.000327445	0.010767052
NM_018135.2	MRPS18A	6	43639040	43655528	0.000327454	0.010767052
NM_052873.1	C14orf179	14	76452096	76550092	0.000327588	0.010767052
NM_021627.2	SENP2	3	185300284	185351339	0.000327665	0.010767052
NM_015167.2	JMJD6	17	74708914	74722881	0.000328122	0.010767052
NM_001097615.1	POLR2J3	7	102178365	102232891	0.000328424	0.010767052
NM_003313.2	TSTA3	8	144694788	144700218	0.000328486	0.010767052
NM_015339.2	ADNP	20	49505585	49547750	0.000329362	0.010785421
NM_213674.1	TPM2	9	35681989	35691017	0.000331009	0.010825506
NM_017814.1	TMEM161A	19	19230429	19249270	0.000331219	0.010825506
NM_145860.1	PDCD10	3	167401086	167452727	0.000332261	0.010845858
NM_031434.2	TMUB1	7	150778167	150780633	0.000332475	0.010845858
NM_016207.2	CPSF3	2	9563697	9613230	0.000333807	0.010874898
NM_017520.2	MPHOSPH8	13	20207821	20247581	0.000334001	0.010874898
NM_017546.3	C2orf29	2	101869264	101886778	0.000334694	0.010880929
NM_005932.1	MIPEP	13	24304328	24463558	0.000334822	0.010880929
NM_022762.3	RMND5B	5	177557997	177577566	0.000337799	0.010967256
NM_001799.2	CDK7	5	68530668	68573250	0.000339459	0.011010689
NM_021999.2	ITM2B	13	48807294	48837063	0.000339859	0.011013226
NM_005484.2	PARP2	14	20811741	20826063	0.000340418	0.011020897
NM_022731.3	NUCKS1	1	205681947	205719404	0.000340757	0.011021459
NM_002945.2	RPA1	17	1733266	1802848	0.000342103	0.011053952
NM_002624.3	PFDN5	12	53689235	53693230	0.000342408	0.011053952

NM_178150.1	FBXO18	10	5932194	5979556	0.000343022	0.011063361
NM_006351.2	TIMM44	19	7991604	8008538	0.00034384	0.011071779
NM_031280.2	MRPS15	1	36921319	36930038	0.000344107	0.011071779
NM_005088.2	AKAP17A	23	1710486	1721407	0.000344254	0.011071779
NM_133635.4	POFUT2	21	46683843	46707813	0.00034473	0.011076665
NM_138436.2	C8orf40	8	42396298	42408151	0.000345607	0.011076741
NM_206839.1	MORF4L1	15	79132903	79190475	0.000345628	0.011076741
NM_006844.3	ILVBL	19	15225795	15236596	0.000345703	0.011076741
NM_004398.2	DDX10	11	108535752	108811657	0.000346416	0.01108919
NM_133433.2	NIPBL	5	36876861	37066515	0.000347248	0.011105459
NM_014765.1	TOMM20	1	235272651	235292251	0.000347605	0.011106496
NM_032983.2	CASP2	7	142985308	143004789	0.000348588	0.011124649
NM_001077198.1	ATG9A	2	220074494	220094439	0.000348824	0.011124649
NM_001037174.1	ARL5A	2	152645498	152685009	0.000349634	0.011134693
NM_153013.3	C5orf33	5	36192694	36242381	0.000349789	0.011134693
NM_003755.3	EIF3G	19	10225690	10230599	0.000350315	0.011141045
NM_016403.3	CWC15	11	94695793	94706776	0.000350968	0.011151452
NM_000411.4	HLCS	21	38123493	38362536	0.000351585	0.011160717
NM_014049.3	ACAD9	3	128598439	128634910	0.000353058	0.011197072
NM_144778.2	MBNL2	13	97873688	98046374	0.000353533	0.011201746
NM_002519.1	NPAT	11	108027942	108093369	0.000354469	0.011221025
NM_146387.1	MRPL4	19	10362640	10370736	0.000355642	0.011247754
NM_031420.2	MRPL9	1	151732119	151736040	0.000357289	0.011283117
NM_006985.1	NPIP	16	15016659	15045915	0.00035742	0.011283117
NM_024546.3	RNF219	13	79188426	79233314	0.00035795	0.011289455
NM_032478.2	MRPL38	17	73894724	73901494	0.000358599	0.011299496
NM_003683.5	RRP1	21	45209394	45225174	0.000359542	0.011309443
NM_022374.1	ATL2	2	38522022	38604432	0.000359818	0.011309443
NM_017996.2	DET1	15	89055714	89089912	0.000359906	0.011309443
XM_001129414.1	AC103686.1	8	48685669	48872743	0.000361041	0.011333941
NM_018477.2	ACTR10	14	58666833	58702351	0.000361348	0.011333941
NM_001005744.1	NUMB	14	73741918	73822477	0.000362232	0.011345245
NM_001380.3	DOCK1	10	128593978	129250781	0.000362372	0.011345245
NM_018473.2	ACOT13	6	24667299	24701942	0.000363393	0.011360638
NM_002916.3	RFC4	3	186507669	186524847	0.000363527	0.011360638
NM_002137.3	HNRNPA2B1	7	26229547	26241149	0.000364231	0.011372259
NM_152449.2	LYSMD4	15	100255906	100273766	0.000365719	0.011400615
NM_004649.5	AP001055.7	21	45548223	45563243	0.000366081	0.011400615
NM_023011.2	UPF3A	13	115047059	115071283	0.000366446	0.011400615
NM_024319.2	C1orf35	1	228288427	228293112	0.000366603	0.011400615
NM_001253.2	CDC5L	6	44355262	44418163	0.000366805	0.011400615
NM_014183.2	DYNLRB1	20	33104214	33128762	0.000367411	0.011409065
NM_006112.2	PPIE	1	40157854	40229586	0.000368272	0.011412841
NM_020738.1	KIDINS220	2	8865408	8977760	0.00036838	0.011412841
NM_018842.3	BAIAP2L1	7	97920963	98030380	0.000368533	0.011412841
NM_004152.2	OAZ1	19	2269598	2273107	0.000369257	0.011424945
NM_016213.3	TRIP4	15	64680003	64747502	0.000371176	0.011459676
NM_006925.3	SRSF5	14	70233829	70238722	0.000371353	0.011459676
NM_152564.3	VPS13B	8	100025494	100889808	0.00037144	0.011459676
NM_015638.2	TRPC4AP	20	33590207	33680674	0.000371719	0.011459676
NM_018188.2	ATAD3A	1	1447531	1470065	0.000372405	0.011461277
NM_020236.2	MRPL1	4	78783674	78873944	0.000372441	0.011461277
NM_001083946.1	C2orf56	2	37458774	37480546	0.000373042	0.011468435

NM_017970.2	C14orf102	14	90744398	90798285	0.000373344	0.011468435
NM_173655.2	EPHA6	3	96533425	97471304	0.000374111	0.011481014
NM_001688.4	ATP5F1	1	111991486	112005395	0.000374425	0.011481014
NM_003850.1	SUCLA2	13	48510622	48575462	0.000375498	0.011503103
NM_006392.2	NOP56	20	2632791	2639039	0.000376093	0.011503103
NM_004124.2	GMFB	14	54941203	54955744	0.000376153	0.011503103
NM_013943.1	CLIC4	1	25071848	25170815	0.000377524	0.011522952
NM_002907.2	RECQL	12	21621845	21657515	0.000377772	0.011522952
NM_023924.3	BRD9	5	850406	892939	0.000377813	0.011522952
NM_006754.2	SYPL1	7	105730949	105753022	0.000378316	0.011528038
NM_033015.2	FASTK	7	150773679	150777953	0.000381206	0.011605762
NM_003913.3	PRPF4B	6	4021501	4065217	0.000382076	0.011612187
NM_006356.2	ATP5H	17	73034955	73043074	0.000382975	0.011612187
NM_003610.3	RAE1	20	55926066	55954267	0.00038301	0.011612187
NM_015525.2	IBTK	6	82879700	82957471	0.000383081	0.011612187
XM_001131752.1	SRP54	14	35452231	35498773	0.000383331	0.011612187
NM_014311.1	SMUG1	12	54558529	54582778	0.000383572	0.011612187
NM_177436.1	CSE1L	20	47662838	47713489	0.000383969	0.011612187
NM_032479.2	MRPL36	5	1798500	1801480	0.000384328	0.011612187
NM_152778.1	MFSD8	4	128838960	128887150	0.000384471	0.011612187
NM_020195.1	SDR39U1	14	24908974	24912007	0.00038494	0.011616098
NM_032433.2	ZNF333	19	14800870	14831772	0.000387672	0.011688216
NM_032285.2	MRI1	19	13875337	13885096	0.000391378	0.011775822
NM_001040020.1	FAM3C	7	120988905	121036422	0.000391646	0.011775822
NM_018229.2	MUDENG	14	57735606	57756797	0.000391916	0.011775822
NM_024666.3	AAGAB	15	67493371	67547074	0.000391954	0.011775822
NM_198195.1	UBA3	3	69103881	69129559	0.000392454	0.011780492
NM_004850.3	ROCK2	2	11319887	11488456	0.000393396	0.011794263
NM_002715.2	PPP2CA	5	133530025	133561833	0.000393829	0.011794263
NM_153831.2	PTK2	8	141667999	142012315	0.000393946	0.011794263
NM_006803.2	AP3M2	8	42010464	42029191	0.000394863	0.011801481
NM_001384.4	DPH2	1	44435672	44439041	0.000394877	0.011801481
NM_032870.2	SFRS18	6	99845927	99873207	0.0003955	0.011801516
NM_180981.1	MRPL52	14	23299092	23304246	0.000395568	0.011801516
NM_017841.1	SDHAF2	11	61197597	61214225	0.000400207	0.011929506
NM_001969.3	EIF5	14	103800493	103811359	0.000401982	0.011971995
NM_004813.1	PEX16	11	45931220	45940363	0.000402671	0.011978318
NM_174856.1	IDH3B	20	2639041	2644865	0.000402894	0.011978318
NM_021930.4	RINT1	7	105172532	105208124	0.000404744	0.01202286
NM_212472.1	PRKAR1A	17	66508110	66528908	0.000405661	0.012039664
NM_182779.3	DVL1	1	1270656	1284730	0.000406128	0.012043055
NM_012428.2	NPTN	15	73852355	73925756	0.000407551	0.012074803
NM_014187.2	TMEM208	16	67261016	67263182	0.000408937	0.012105374
NM_016173.3	HEMK1	3	50606583	50622366	0.00040973	0.012118372
NM_014353.4	RAB26	16	2198625	2204141	0.000410156	0.012120509
NM_016645.2	NGRN	15	90808891	90816463	0.000411063	0.012136815
NM_014712.1	SETD1A	16	30968615	30995985	0.000412327	0.012163658
NM_001003696.1	ATP5J	21	27088815	27107984	0.000413379	0.012183789
NM_002359.2	MAFG	17	79876149	79885588	0.000413722	0.012183789
NM_012263.3	TTLL1	22	43414609	43485434	0.000414305	0.012190472
NM_024920.3	DNAJB14	4	100817405	100867883	0.000416825	0.012249112
NM_006035.3	CDC42BPB	14	103398717	103523799	0.000417014	0.012249112
NM_015530.3	GORASP2	2	171784974	171823639	0.000418717	0.012288598

NM_006985.1	RP11-166B2.1	16	12021116	12070500	0.000419717	0.0122996
NM_006232.2	POLR2H	3	184079506	184086384	0.000419811	0.0122996
NM_001607.2	ACAA1	3	38164201	38178733	0.000422059	0.012322816
NM_001695.4	ATP6V1C1	8	104033291	104085279	0.000422066	0.012322816
NM_032434.2	ZNF512	2	27805893	27873711	0.000422205	0.012322816
NM_004132.2	HABP2	10	115312785	115349358	0.00042237	0.012322816
NM_017871.4	CPSF3L	1	1246965	1260071	0.000422404	0.012322816
NM_018638.4	ETNK1	12	22778009	22843608	0.000423516	0.012326015
NM_145701.1	CDCA4	14	105475910	105487425	0.000424145	0.012326015
NM_013255.3	MKLN1	7	130794855	131181395	0.000424495	0.012326015
NM_020744.2	MTA3	2	42721709	42984087	0.000424709	0.012326015
NM_005056.1	KDM5A	12	389223	498620	0.000424716	0.012326015
NM_005735.2	ACTR1B	2	98272431	98280570	0.000425462	0.012326015
NM_199295.1	APITD1	1	10490159	10512210	0.000425962	0.012326015
NM_004341.3	CAD	2	27440258	27466660	0.00042619	0.012326015
NM_178588.1	PPP2R5C	14	102228222	102394075	0.000426203	0.012326015
NM_058169.2	LOH12CR1	12	12510020	12619838	0.000426463	0.012326015
NM_032308.1	RPAIN	17	5322961	5336196	0.000426802	0.012326015
BU753580	AL139008.5	9	33638033	33638492	0.000427106	0.012326015
NM_006036.3	PREPL	2	44543420	44588945	0.000427196	0.012326015
NM_024683.3	C17orf42	17	29226001	29233286	0.000429701	0.012387831
NM_018202.3	TMEM57	1	25757388	25826700	0.000430304	0.012394782
NM_002768.2	CHMP1A	16	89710846	89724129	0.000433385	0.012454994
NM_030640.1	DUSP16	12	12628831	12715317	0.00043366	0.012454994
NM_007080.2	LSM6	4	147096837	147121152	0.000433823	0.012454994
NM_021932.4	RIC8A	11	207511	215113	0.00043385	0.012454994
NM_000175.2	GPI	19	34855815	34891236	0.000435184	0.012476708
NM_017906.2	PAK1IP1	6	10694928	10710015	0.000435336	0.012476708
NM_138798.1	MITD1	2	99777890	99797521	0.000436136	0.012489194
NM_198398.1	ERGIC3	20	34129770	34145405	0.00043676	0.012490384
NM_032711.2	MAFG	17	79876149	79885588	0.000436908	0.012490384
NM_144716.2	CCDC12	3	46963216	47023500	0.00043745	0.012495431
NM_016305.2	SS18L2	3	42623332	42636606	0.000438428	0.012512928
NM_002687.3	PNN	14	39644425	39652422	0.000439013	0.012519188
NM_001040662.1	C14orf138	14	50575350	50583318	0.000440295	0.012543837
NM_016622.2	MRPL35	2	86426580	86440917	0.000440632	0.012543837
NM_017728.2	TMEM104	17	72772622	72835918	0.000440977	0.012543837
NM_007096.2	CLTA	9	36190892	36304778	0.000441386	0.012545054
NM_144664.3	FAM76B	11	95502106	95522954	0.000442463	0.012565212
NM_014657.1	TTI1	20	36611409	36661870	0.000443313	0.012570612
NM_016121.3	KCTD3	1	215740735	215795147	0.000443388	0.012570612
NM_001080415.1	AC018450.1	3	142720010	142779567	0.000446349	0.012644105
NM_016628.2	WAC	10	28821422	28909928	0.000446959	0.012645753
NM_017945.2	SLC35A5	3	112280556	112304424	0.000447147	0.012645753
NM_032560.3	SMEK1	14	91923955	91976644	0.000450983	0.012735848
NM_003929.1	RAB7L1	1	205737114	205744588	0.000451521	0.012735848
NM_018229.2	MUDENG	14	57735606	57756797	0.000451763	0.012735848
NM_012384.2	GMEB2	20	62218955	62258394	0.000452076	0.012735848
NM_021004.2	DHRS4	14	24422964	24438483	0.000452193	0.012735848
NM_002936.3	RNASEH1	2	3592383	3606206	0.000453708	0.012762602
NM_080794.2	MRPL39	21	26957968	26979829	0.000453889	0.012762602
NM_032885.4	ATG4D	19	10654593	10664093	0.000455107	0.01278633
NM_000283.1	PDE6B	4	619373	664571	0.000455668	0.012791604

NM_015049.1	TRAK2	2	202241930	202316302	0.000456398	0.012801579
NM_033632.2	FBXW7	4	153242410	153456172	0.000458946	0.012862529
NM_005052.2	RAC3	17	79989532	79992077	0.000459347	0.012863217
NM_181696.1	PRDX1	1	45976708	45988719	0.000460581	0.012880063
NM_002568.3	PABPC1	8	101698044	101735037	0.000460701	0.012880063
NM_007364.2	TMED3	15	79603404	79703846	0.00046144	0.012890192
NM_152720.1	NEK3	13	52706779	52733996	0.000461902	0.012892558
NM_002266.2	KPNA2	17	66031848	66042969	0.000466638	0.012994448
NM_145691.3	ATPAF2	17	17918373	17942523	0.000466691	0.012994448
NM_201281.1	MTMR2	11	95566046	95658479	0.000467121	0.012995848
BC037864	POU2F1	1	167190120	167396582	0.000468308	0.013018271
NR_003192.1	C3orf78	3	52568029	52613253	0.000469588	0.013033134
NM_004589.1	SCO1	17	10583654	10600885	0.000469648	0.013033134
NM_018034.2	WDR70	5	37379314	37753537	0.000470137	0.013033134
NM_032276.2	RHBDD1	2	227700297	227863931	0.000470403	0.013033134
NM_024580.5	EFTUD1	15	82422561	82555104	0.000470747	0.013033134
NM_001012661.1	SLC3A2	11	62623518	62656352	0.0004712	0.013033734
NM_003680.2	YARS	1	33240840	33283754	0.00047153	0.013033734
NM_024087.1	ASB9	23	15252900	15288281	0.000474627	0.013108224
NM_001382.2	DPAGT1	11	118967213	118979041	0.000475416	0.013108224
NM_001042734.1	SEC24B	4	110354928	110461612	0.000476013	0.013108224
NM_019044.3	CCDC93	2	118673054	118771709	0.00047665	0.013108224
NM_014177.1	C18orf55	18	71815746	71826197	0.000476983	0.013108224
NM_152713.2	STT3A	11	125461607	125495110	0.000477074	0.013108224
NM_015343.3	CTDNEP1	17	7146906	7155280	0.000477145	0.013108224
NM_013397.4	TOMM6	6	41755400	41757636	0.000477709	0.013108224
NM_175610.2	TJP1	15	29991571	30248497	0.000477784	0.013108224
NM_032117.2	MND1	4	154265801	154336270	0.000478056	0.013108224
NM_006473.2	TAF6L	11	62538775	62554814	0.000478938	0.013121915
NM_014106.2	ZNF770	15	35270542	35280474	0.000479407	0.013124256
NM_014463.1	LSM3	3	14219858	14242619	0.000481982	0.013184198
NM_012323.2	MAFF	22	38597889	38612518	0.000482416	0.013185534
NM_198590.1	BSG	19	571325	583492	0.000484606	0.013234819
NM_001003962.1	CAPNS1	19	36630918	36641254	0.000485338	0.013244222
NM_001099436.1	ULK3	15	75128459	75135552	0.000486433	0.013263542
BI024234	AL139008.5	9	33638033	33638492	0.000488219	0.01327996
NM_007266.2	ZNF512	2	27805893	27873711	0.000488243	0.01327996
NM_000996.2	RPL35A	3	197676858	197683481	0.000488614	0.01327996
NM_203463.1	LASS6	2	169312759	169631644	0.00048875	0.01327996
NM_153332.3	ERI1	8	8859657	8974256	0.000488976	0.01327996
NM_013285.1	GNL2	1	38032417	38061536	0.000490471	0.013287667
NM_002740.5	PRKCI	3	169940153	170023769	0.000490587	0.013287667
NM_013433.3	TNPO2	19	12810008	12834810	0.000490646	0.013287667
NM_022340.2	ZFYVE20	3	15111580	15140670	0.000491141	0.013287667
NM_001614.2	ACTG1	17	79476999	79479827	0.000491249	0.013287667
NM_020177.2	FEM1C	5	114856608	114880591	0.000491589	0.013287667
NM_018997.1	MRPS21	1	150266289	150281414	0.000492524	0.013298784
NM_181656.1	C17orf58	17	65987218	65989765	0.000492778	0.013298784
NM_138927.1	SON	21	34914924	34949812	0.000493858	0.013317426
NM_001745.2	CAMLG	5	134074191	134087847	0.000494487	0.013323897
NM_012208.2	HARS2	5	140071011	140078889	0.000495008	0.013325867
NM_020187.2	C3orf37	3	128997671	129024146	0.00049566	0.013325867
NM_015881.5	DKK3	11	11984547	12031316	0.000495901	0.013325867

NM_012392.2	PEF1	1	32095463	32110497	0.000496118	0.013325867
NM_002503.3	NFKBIB	19	39390615	39399531	0.000498611	0.013379475
NM_018944.2	C21orf45	21	33640530	33651380	0.000498896	0.013379475
NM_001009184.1	GRINA	8	145064226	145067583	0.000499998	0.013398526
NM_006421.3	ARFGEF1	8	68085747	68255912	0.000500757	0.013402132
NM_001018.3	RPS15	19	1438363	1440492	0.000500915	0.013402132
NM_001031727.2	MRI1	19	13875337	13885096	0.000501385	0.013404209
NM_015421.1	TMEM186	16	8889037	8891505	0.00050353	0.013451058
XM_001129672.1	DPY30	2	32092878	32264881	0.000505608	0.013460583
NM_007065.3	CDC37	19	10501809	10514271	0.000505801	0.013460583
NM_015373.3	CBY1	22	39052641	39069859	0.000505898	0.013460583
NM_014613.2	FAF2	5	175874629	175937075	0.000506566	0.013460583
NM_016004.2	IFT52	20	42219571	42275936	0.000506577	0.013460583
NM_006833.4	COPS6	7	99686577	99689823	0.000506957	0.013460583
NM_014982.2	PCNX	14	71374122	71582099	0.000507221	0.013460583
NM_001033604.1	BBS9	7	33168856	33645680	0.000507626	0.013460583
NM_001025.4	RPS23	5	81569177	81574396	0.000508184	0.013460583
NM_004275.3	MED20	6	41873092	41888877	0.000508801	0.013460583
NM_005649.2	ZNF354A	5	178138593	178157703	0.000509081	0.013460583
NM_004301.3	ACTL6A	3	179280668	179306196	0.00050951	0.013460583
NM_017772.2	TBC1D22B	6	37225548	37300746	0.000509952	0.013460583
NM_003576.3	STK24	13	99102455	99230194	0.000510193	0.013460583
NM_015176.1	FBXO28	1	224301789	224349749	0.000510445	0.013460583
NM_178863.2	KCTD13	16	29917657	29937549	0.000510452	0.013460583
NM_005698.2	FAM189B	1	155216996	155225274	0.000510661	0.013460583
XM_934796.2	SPCS2	11	74660292	74690076	0.000511212	0.013460583
NM_002874.3	RAD23B	9	110045418	110094475	0.000511361	0.013460583
NM_019024.1	HEATR5B	2	37195526	37311485	0.00051887	0.013647485
NM_001098402.1	ZNF295	21	43406940	43430496	0.000519609	0.013647485
NM_017510.4	TMED9	5	177019159	177023125	0.000519657	0.013647485
NM_004768.2	SRSF11	1	70671365	70718735	0.000523385	0.013734854
NM_002539.1	ODC1	2	10580094	10588630	0.000525432	0.013771858
NM_003591.2	CUL2	10	35297479	35379570	0.000526042	0.013771858
NM_199227.1	METAP1D	2	172864490	172947158	0.000526355	0.013771858
NM_020401.2	NUP107	12	69080731	69136472	0.000526405	0.013771858
NM_003744.5	NUMB	14	73741918	73822477	0.000526891	0.013774032
NM_020447.3	C15orf17	15	75192329	75199462	0.000529592	0.013834087
NM_020320.2	RARS2	6	88224096	88299735	0.000532155	0.013890428
NM_006703.2	NUDT3	6	34255997	34360451	0.000534726	0.013946882
NM_006702.3	PNPLA6	19	7599043	7626652	0.000535135	0.013946937
NM_031445.2	AMMECR1L	2	128619204	128643496	0.000535602	0.013948466
NM_024818.3	UBA5	3	132373290	132396941	0.000536669	0.013953206
NM_007236.3	AC012652.1	15	41523342	41574088	0.000537008	0.013953206
NM_001001.3	RPL36AL	14	50085407	50087361	0.000537528	0.013953206
NM_002477.1	MYL5	4	667369	675822	0.000537644	0.013953206
NM_005740.2	DNAL4	22	39174513	39190203	0.000537822	0.013953206
NM_004444.4	EPHB4	7	100400187	100425143	0.000539937	0.013988262
NM_002662.2	PLD1	3	171318195	171528740	0.000539991	0.013988262
NM_177988.1	MRPL47	3	179306073	179322442	0.00054076	0.013988714
NM_032358.2	CCDC77	12	498516	551811	0.000540937	0.013988714
NM_007044.2	KATNA1	6	149916009	149970108	0.000541544	0.013988714
NM_080597.2	OSBPL1A	18	21742008	21977823	0.000541644	0.013988714
NM_030811.3	MRPS26	20	3026591	3028900	0.000543196	0.014018222

NM_201627.1	TRIM41	5	180649499	180662809	0.000547077	0.014107755
NM_198329.1	UBA5	3	132373290	132396941	0.00054919	0.014151576
NM_007178.3	STRAP	12	16035288	16056403	0.000549621	0.014152026
NM_006190.3	ORC2	2	201773696	201828403	0.000551565	0.014191387
NM_016216.2	DBR1	3	137879854	137893791	0.000552831	0.014192555
NM_002810.2	PSMD4	1	151227179	151239955	0.000552899	0.014192555
NM_015084.1	MRPS27	5	71515236	71616473	0.000553182	0.014192555
NM_018142.2	INTS10	8	19674651	19709594	0.000553269	0.014192555
NM_139062.1	CSNK1D	17	80202246	80231573	0.000555024	0.014223968
NM_024772.3	ZMYM1	1	35525387	35581460	0.000555325	0.014223968
NM_001025300.1	RAB12	18	8609443	8639380	0.00055763	0.014244121
NM_005644.2	TAF12	1	28915835	28969597	0.000558437	0.014244121
NM_001024228.1	ARF1	1	228270361	228286910	0.000558514	0.014244121
NM_145030.1	C7orf47	7	100032905	100034188	0.000558543	0.014244121
NM_004719.2	SRSF2IP	12	46315192	46384363	0.000558646	0.014244121
NM_024345.3	DCAF10	9	37800499	37867663	0.000559104	0.014244121
NM_001009937.1	SLC25A26	3	66119285	66438130	0.000559422	0.014244121
NM_014110.3	PPP1R8	1	28157289	28178187	0.000559442	0.014244121
NM_025065.6	RPF1	1	84944942	84963473	0.000560597	0.014262912
NM_005536.2	IMPA1	8	82570196	82598928	0.00056327	0.014314371
NM_014516.2	CNOT3	19	54641444	54659419	0.000563881	0.014314371
NM_018443.2	ZNF302	19	35168544	35177302	0.000564246	0.014314371
NM_203385.1	RNH1	11	494512	507300	0.000564505	0.014314371
NM_016045.1	SLMO2	20	57608200	57617964	0.000564711	0.014314371
NM_003344.2	UBE2H	7	129470572	129592789	0.000567552	0.014374956
NM_006420.2	ARFGEF2	20	47538427	47653230	0.000567941	0.014374956
NM_176815.3	DHFRL1	3	93766680	93782233	0.000569648	0.014406426
NM_025264.3	THUMPD2	2	39963200	40006407	0.000570421	0.014406426
NM_021132.1	PPP3CB	10	75196186	75255782	0.000570447	0.014406426
NM_003136.2	SRP54	14	35452231	35498773	0.000574099	0.01448795
NM_006012.2	CLPP	19	6361463	6368915	0.000577208	0.014555675
NM_001048223.1	RP3-453C12.13	20	43991840	44039250	0.000579989	0.014615036
NM_032494.1	ZC3H8	2	112973254	113012713	0.000582082	0.01463997
NM_015491.1	SFRS18	6	99845927	99873207	0.000582361	0.01463997
NM_006621.4	AHCYL1	1	110527308	110566357	0.000582813	0.01463997
NM_181870.1	DVL1	1	1270656	1284730	0.000582827	0.01463997
NM_007108.2	TCEB2	16	2821415	2827278	0.000583117	0.01463997
NM_152902.3	TIPRL	1	168148171	168171346	0.000585082	0.014678519
NM_001001977.1	ATP5E	20	57600522	57607437	0.000585716	0.014680193
NM_015135.1	NUP205	7	135242667	135333505	0.000586007	0.014680193
NM_032138.3	KBTBD7	13	41765711	41768702	0.000587889	0.014716588
NM_003721.2	RFXANK	19	19303008	19312678	0.000589048	0.014734815
NM_002129.2	HMGB2	4	174252846	174256276	0.000590018	0.014748292
NM_021237.3	RP11-884K10.5	3	53918437	53926015	0.000590767	0.014756242
NM_001033723.1	ZNF704	8	81540686	81787016	0.000594179	0.014830628
NM_020647.2	JPH1	8	75146935	75233563	0.000595814	0.014860607
NM_198971.1	HINFP	11	118992288	119006752	0.000597008	0.014879548
NM_015317.1	PUM2	2	20448452	20551995	0.000597561	0.014882497
NM_005190.3	CCNC	6	99990256	100016849	0.000598142	0.01488569
NM_201280.1	MUTED	6	7881755	8064647	0.00059856	0.01488569
NM_003758.2	EIF3J	15	44829255	44855001	0.000600869	0.014932259
NM_003794.2	SNX4	3	125165495	125239041	0.000602373	0.014953258
NM_005219.3	DIAPH1	5	140894583	140998622	0.000602892	0.014953258

NM_024520.1	C2orf47	2	200820040	200873263	0.000603024	0.014953258
NM_006387.5	CHERP	19	16628700	16653263	0.000603906	0.014953648
NM_181463.1	MRPL55	1	228294380	228297013	0.000603914	0.014953648
NM_025137.3	SPG11	15	44854894	44955876	0.000605277	0.014970381
XM_939897.2	SIRPB2	20	1451386	1472233	0.000605925	0.014970381
NM_144588.4	ZFYVE27	10	99496880	99520652	0.000606041	0.014970381
NM_181472.1	CMTM7	3	32433163	32524559	0.000606796	0.014970381
AL137321	RAB2A	8	61429416	61536186	0.000607209	0.014970381
NM_003084.1	SNAPC3	9	15422702	15465951	0.000607215	0.014970381
NM_002490.3	NDUFA6	22	42481529	42486959	0.000608806	0.014991833
NM_000714.4	TSPO	22	43547520	43559248	0.000608961	0.014991833
XR_016048.1	AC132812.1	17	62745781	62758831	0.00061115	0.015029809
NM_025251.1	ARHGAP39	8	145754563	145831201	0.000611577	0.015029809
NM_031885.2	BBS2	16	56518366	56554195	0.000611821	0.015029809
NM_080599.1	UPF2	10	11962021	12085169	0.000612895	0.015045382
NM_012079.2	DGAT1	8	145539954	145550573	0.000615485	0.015087561
NM_021942.4	C4orf41	4	184580420	184634745	0.000615495	0.015087561
NM_025133.3	FBXO11	2	48016455	48132932	0.000617081	0.015115623
NM_020940.2	FAM160B1	10	116581503	116659591	0.000618915	0.015139302
NM_005481.2	MED16	19	867964	893218	0.000619313	0.015139302
NM_024585.2	ARMC7	17	73106082	73126360	0.000619375	0.015139302
NM_025176.4	NINL	20	25433341	25566153	0.0006203	0.015150831
NM_001003805.1	ATP5S	14	50779047	50802276	0.000620787	0.015150831
NM_006411.2	AGPAT1	6	32135989	32145873	0.000621426	0.015150831
NM_016075.2	VPS36	13	52986739	53024763	0.000621618	0.015150831
NM_006372.3	SYNCRIP	6	86267696	86353510	0.000622932	0.015172041
NM_016056.2	TMBIM4	12	66530328	66563807	0.000623925	0.015185432
NM_001007188.1	DEPDC5	22	32149944	32303001	0.000625104	0.0151932
NM_002492.2	NDUFB5	3	179322478	179345435	0.000625204	0.0151932
NM_003472.2	DEK	6	18224099	18265054	0.000625576	0.0151932
NM_015509.2	NECAP1	12	8234807	8250373	0.00062874	0.01525607
NM_007277.4	EXOC3	5	443273	472052	0.000629057	0.01525607
NM_002810.2	PSMD4	1	151227179	151239955	0.000630794	0.015287379
NM_001004333.2	RNASEK	17	6915798	6917850	0.000631958	0.015297959
NM_015102.2	NPHP4	1	5922871	6052533	0.000632252	0.015297959
NM_017588.2	WDR5	9	137001210	137025093	0.000632572	0.015297959
NM_015047.1	UBR4	1	19401000	19536770	0.000633341	0.015305743
NM_019600.2	KIAA1370	15	52873530	52970820	0.000634642	0.015326365
NM_006415.2	SPTLC1	9	94793427	94877690	0.000635578	0.01533813
NM_018270.3	C20orf20	20	61427805	61431945	0.000636971	0.015360919
NM_001077628.1	APH1A	1	150237799	150241980	0.000637866	0.015371684
NM_025185.3	TANC2	17	61086898	61505067	0.000638468	0.015375352
NM_001042410.1	ANKZF1	2	220094479	220101391	0.000639205	0.015382276
NM_004977.2	KCNC3	19	50815194	50836772	0.000640994	0.015403672
NM_032862.2	TIGD5	8	144680005	144682485	0.000643258	0.015447217
NM_024619.2	FN3KRP	17	80674582	80685892	0.000644942	0.015465792
NM_003825.2	SNAP23	15	42787801	42825259	0.000645129	0.015465792
NM_022085.3	TXNDC5	6	7881750	7911047	0.000645387	0.015465792
NM_002976.2	SCN7A	2	167260083	167350757	0.000647359	0.015502183
NM_037370.1	CCNDBP1	15	43477455	43487396	0.000648252	0.015512707
NM_006170.2	NOP2	12	6666029	6677480	0.00065063	0.015558584
NM_014521.2	SH3BP4	2	235860617	235964358	0.000651078	0.015558584
NM_006589.2	FAM189B	1	155216996	155225274	0.000651809	0.015565174

NM_012203.1	GRHPR	9	37422663	37436987	0.000654228	0.015605839
NM_001035006.1	RPL17	18	47014858	47018906	0.000654424	0.015605839
NM_016200.3	NAA38	7	117824086	117832878	0.000655408	0.015610092
NM_014771.2	RNF40	16	30773599	30787628	0.000655967	0.015610092
NM_005003.2	NDUFAB1	16	23592323	23607677	0.000656255	0.015610092
NM_022106.1	C20orf177	20	58508819	58523735	0.000656635	0.015610092
NM_016248.2	AKAP11	13	42846289	42897396	0.000656883	0.015610092
NM_024881.4	SLC35E1	19	16660650	16683193	0.000657744	0.015618509
NM_017528.2	WBSCR22	7	73097355	73119491	0.00065815	0.015618509
XR_018197.1	YBX1	1	43148098	43168020	0.000659542	0.015640695
NM_013943.1	CLIC4	1	25071848	25170815	0.000660846	0.015659709
NM_001033859.1	ACADVL	17	7123131	7128587	0.000661259	0.015659709
NM_015268.3	DNAJC13	3	132136370	132257876	0.000661884	0.015663675
NM_080836.2	STK35	20	2082257	2157684	0.000664866	0.015715441
NM_014704.2	KIAA0562	1	3728645	3773797	0.00066499	0.015715441
NM_016586.1	MBIP	14	36767764	36789882	0.000665667	0.015720574
NM_006844.3	ILVBL	19	15225795	15236596	0.000667336	0.015749122
NM_002806.2	PSMC6	14	53173890	53195305	0.00067249	0.015859832
NM_014159.4	SETD2	3	47057919	47205457	0.000674448	0.015895053
NM_003016.3	SRSF2	17	74730201	74733413	0.000677841	0.015955443
NM_018999.2	FAM190B	10	86088342	86278276	0.000677943	0.015955443
NM_002938.2	RNF4	4	2463947	2627047	0.000681133	0.016002173
NM_001136232.1	SEC13	3	10334815	10362862	0.00068155	0.016002173
NM_001003800.1	BICD2	9	95473645	95527094	0.000681608	0.016002173
NM_022553.4	VPS52	6	33218049	33239824	0.000681799	0.016002173
NM_014666.2	CLINT1	5	157212751	157286183	0.000683895	0.016040352
NM_181454.1	MRPL55	1	228294380	228297013	0.00068581	0.016074259
NM_032331.3	ECE2	3	183967438	184010819	0.000687415	0.016090683
NM_020905.2	RDH14	2	18735989	18741946	0.000687451	0.016090683
NM_017828.3	COMMD4	15	75628232	75634268	0.000691099	0.01616502
NM_004730.1	ETF1	5	137841784	137878989	0.000694742	0.01621793
NM_014319.3	LEMD3	12	65563371	65642107	0.000694996	0.01621793
NM_007111.3	TFDP1	13	114239036	114295504	0.000695557	0.01621793
NM_006986.3	MAGED1	23	51546103	51645453	0.000695695	0.01621793
NM_019030.2	DHX29	5	54552073	54603550	0.000695731	0.01621793
NM_020307.2	CCNL1	3	156864297	156878549	0.000696235	0.016218617
NM_006319.3	CDIPT	16	29869678	29875055	0.000698709	0.016263577
NM_020390.5	EIF5A2	3	170606204	170626482	0.000699436	0.016263577
NM_001990.2	EYA3	1	28296855	28415207	0.000699753	0.016263577
NM_024754.3	PTCD2	5	71616194	71656052	0.00070034	0.016263577
XM_936354.2	FAM82B	8	87480486	87526586	0.000700541	0.016263577
NM_003598.1	TEAD2	19	49843857	49865714	0.000701638	0.016277999
NM_198536.1	TMEM205	19	11453452	11456981	0.000703028	0.016299203
NM_001077665.2	AGAP6	10	51748078	51770259	0.00070585	0.016353534
XM_001130192.1	ISY1	3	128806418	128880136	0.000708013	0.016392552
NM_018412.3	ST7	7	116593292	116870157	0.000708916	0.016393268
NM_145808.2	MTPN	7	135611509	135662107	0.000709555	0.016393268
NM_021821.2	MRPS35	12	27863729	27909227	0.000710116	0.016393268
NM_018693.2	FBXO11	2	48016455	48132932	0.000710429	0.016393268
NM_005437.2	NCOA4	10	51565108	51590734	0.000710704	0.016393268
NM_005898.4	CAPRIN1	11	34073230	34124157	0.00071128	0.016393268
NM_018982.3	YIPF1	1	54317392	54355507	0.000713803	0.01642823
NM_001949.2	E2F3	6	20402398	20493941	0.000714624	0.01642823

NM_013328.2	PYCR2	1	226074396	226111978	0.000715172	0.01642823
NM_133466.2	ZFP82	19	36874593	36909558	0.000715813	0.01642823
NM_152594.1	SPRED1	15	38545052	38649450	0.000715861	0.01642823
NM_152373.2	ZNF684	1	40997233	41013841	0.000716606	0.01642823
NM_000398.4	CYB5R3	22	43014814	43045574	0.00071669	0.01642823
NM_001033506.1	CSTF3	11	33098734	33183917	0.000716891	0.01642823
NM_058181.1	C21orf57	21	47706251	47717665	0.000717537	0.01642823
NM_001921.2	DCTD	4	183811213	183839089	0.000717715	0.01642823
NM_152379.2	C1orf131	1	231359509	231376933	0.000719298	0.016453461
NM_006003.1	UQCRFS1	19	29698167	29704136	0.000722463	0.016514813
NM_001375.2	DNASE2	19	12986023	12992335	0.000723341	0.016523861
NM_003776.2	MRPL40	22	19419425	19423598	0.000724157	0.016531464
NM_006265.1	RAD21	8	117858174	117887105	0.00072653	0.016574571
NM_001931.2	DLAT	11	111895538	111935114	0.000727714	0.016590522
NM_014671.1	UBE3C	7	156931607	157062066	0.000729217	0.016613721
NM_005095.2	ZMYM4	1	35734568	35887659	0.00072979	0.016615531
NM_182972.2	IRF2BP2	1	234740015	234745271	0.000730572	0.016615531
NM_005189.1	CBX2	17	77751993	77761378	0.000730753	0.016615531
NM_016603.1	FAM13B	5	137273649	137387650	0.000732447	0.016632627
NM_001039457.1	ATP6V0B	1	44440159	44443967	0.000732477	0.016632627
NM_018184.2	ARL8B	3	5163905	5222596	0.00073308	0.016635286
NM_016013.2	NDUFAF1	15	41679547	41694658	0.000734481	0.016654403
NM_030900.2	TBRG4	7	45139699	45151646	0.000734896	0.016654403
NM_005137.2	DGCR2	22	19023799	19109967	0.000736989	0.016673618
NM_207304.1	MBNL2	13	97873688	98046374	0.00073726	0.016673618
NM_017542.3	POGK	1	166808684	166825581	0.000738091	0.016673618
NM_138442.2	CCDC124	19	18043824	18054793	0.000738491	0.016673618
NM_024595.1	AKIRIN1	1	39456895	39484692	0.000739241	0.016673618
NM_004565.2	PEX14	1	10532345	10690815	0.000739246	0.016673618
NM_001083613.1	TMEM219	16	29973351	29984373	0.00073936	0.016673618
NM_007175.5	ERLIN2	8	37594117	37616619	0.000739642	0.016673618
NM_145062.1	ZUFSP	6	116956781	116989957	0.000741151	0.016696631
NM_024629.2	MLF1IP	4	185615772	185655287	0.000742277	0.016697636
NM_015934.3	NOP58	2	203130439	203168389	0.000742648	0.016697636
NM_178037.1	ERC1	12	1100404	1605099	0.000743046	0.016697636
NM_001029998.2	SLC10A7	4	147175127	147443123	0.000743147	0.016697636
NM_017943.2	FBXO34	14	55738021	55820329	0.000744719	0.016720309
NM_138446.1	C7orf30	7	23338358	23351348	0.000745134	0.016720309
NM_006494.1	ERF	19	42751717	42759309	0.000747714	0.016767207
NM_022744.1	C16orf58	16	31500810	31519734	0.00075066	0.016822255
NM_033411.2	RWDD2A	6	83903098	83906256	0.000752139	0.016841001
NM_002901.1	RCN1	11	31833939	32127301	0.000752481	0.016841001
NM_016107.3	ZFR	5	32354456	32444867	0.000754308	0.016870852
NM_013366.3	ANAPC2	9	140069236	140083057	0.000757647	0.016925164
NM_012140.3	SLC25A10	17	79679337	79688041	0.000757725	0.016925164
NM_006170.2	NOP2	12	6666029	6677480	0.000758544	0.016932405
NM_018457.2	PRR13	12	53835433	53840426	0.000759219	0.016936426
NM_016097.3	IER3IP1	18	44681413	44702745	0.000759729	0.016936749
NM_007260.2	LYPLA2	1	24117460	24122029	0.000762422	0.016985723
NM_003258.2	TK1	17	76170160	76183314	0.00076293	0.016985985
NM_012316.3	KPNA6	1	32573639	32642169	0.000763861	0.016994905
NM_181837.1	ORC3	6	88299839	88377169	0.000764324	0.016994905
NM_058241.1	CCNT2	2	135675805	135716912	0.000765677	0.017013946

NM_018989.1	RBM27	5	145583113	145718814	0.00076826	0.017050779
NM_033428.1	C9orf123	9	7796490	7888380	0.000768331	0.017050779
NM_080875.1	MIB2	1	1550795	1565990	0.000769567	0.017067131
NM_001690.2	ATP6V1A	3	113465866	113530903	0.000770114	0.017068191
NM_004958.2	MTOR	1	11166592	11322564	0.000770623	0.017068434
NM_138367.1	ZNF251	8	145946298	145981802	0.000772346	0.017077216
NM_020765.2	UBR4	1	19401000	19536770	0.000772351	0.017077216
NM_015062.3	PPRC1	10	103892787	103910088	0.000772517	0.017077216
NM_139281.2	WDR36	5	110427414	110481518	0.000774248	0.01710443
NM_203364.2	CAPRIN1	11	34073230	34124157	0.000774972	0.017109385
NM_005783.3	TXNDC9	2	99935445	99957165	0.000777018	0.017142798
NM_170784.1	MKKS	20	10385428	10414870	0.000777488	0.017142798
NM_000140.2	FECH	18	55215515	55254004	0.000778517	0.017146509
NM_006463.3	STAMBP	2	74056086	74100786	0.00077902	0.017146509
NM_000317.1	PTS	11	112097088	112140678	0.000779322	0.017146509
NM_021239.1	RBM25	14	73525144	73588122	0.00077966	0.017146509
NM_002264.1	KPNA1	3	122140796	122233792	0.000782375	0.017195169
NM_177966.4	PDE12	3	57542003	57547684	0.000783983	0.017219447
NM_152726.1	EFHA1	13	22066836	22178353	0.000787103	0.017276881
NM_004251.3	RAB9A	23	13707244	13728625	0.000789374	0.017304534
NM_004528.2	MGST3	1	165600098	165631033	0.000790366	0.01731519
NM_022484.4	TMEM168	7	112405787	112430647	0.000793884	0.017381127
NM_144567.3	ANGEL2	1	213165524	213189168	0.000794857	0.017391312
XM_937968.2	C4orf48	4	2043689	2045697	0.000795537	0.017393948
NM_173831.3	ZNF707	8	144766622	144796068	0.000795994	0.017393948
NM_001006933.1	TCEAL3	23	102862379	102884618	0.000796548	0.017394943
NM_032124.4	HDHD2	18	44633782	44676871	0.000797549	0.01740569
NM_175921.4	C5orf51	5	41904290	41921738	0.000798148	0.017407665
NM_002553.2	ORC5	7	103766788	103848495	0.000798712	0.017408865
NM_030800.1	C15orf44	15	65871095	65903474	0.000799274	0.017410036
NM_199122.1	TBRG4	7	45139699	45151646	0.000801241	0.017441766
NM_203459.1	CAMSAP1L1	1	200708686	200829832	0.000802181	0.017451144
NM_014306.3	C22orf28	22	32783569	32808242	0.000806804	0.017540563
NM_021203.2	SRPRB	3	133502877	133544616	0.00081024	0.017604078
NM_016424.3	LUC7L3	17	48796905	48833574	0.000810913	0.017607506
NM_006745.3	SC4MOL	4	166248775	166264312	0.000812019	0.017620341
NM_030790.3	ITFG1	16	47188298	47495173	0.000815195	0.017670147
NM_003690.3	PRKRA	2	179296141	179316239	0.000815347	0.017670147
NM_199123.1	SETD3	14	99864083	99947216	0.000818243	0.017721703
XM_001126912.1	RP11-632K20.1	15	32736888	32747737	0.000819105	0.017722088
NM_001005861.2	RYK	3	133794023	133969494	0.000819297	0.017722088
NM_021145.2	DMTF1	7	86781677	86825648	0.000821261	0.01775255
NM_012475.4	USP21	1	161129240	161135513	0.000822087	0.01775255
NM_002717.2	PPP2R2A	8	26149007	26230196	0.000822262	0.01775255
NM_001564.2	ING2	4	184426147	184432249	0.000826416	0.017825178
NM_003842.3	TNFRSF10B	8	22877646	22926692	0.000826667	0.017825178
NM_006323.2	SEC24B	4	110354928	110461612	0.00082859	0.017847969
NM_182627.1	WDR53	3	196281056	196295545	0.000828767	0.017847969
NM_006345.3	SLC30A9	4	41992489	42089551	0.000829892	0.017857146
NM_018256.2	WDR12	2	203744993	203879521	0.000830237	0.017857146
NM_007171.3	POMT1	9	134378289	134399193	0.000830941	0.017861047
NM_004584.2	RAD9A	11	67159423	67165880	0.000834485	0.017918148
NM_018358.2	ABCF3	3	183903811	183911800	0.000834644	0.017918148

NM_030809.1	CSRNP2	12	51454990	51477333	0.000835822	0.017932175
NM_014691.2	AQR	15	35147750	35261956	0.000837077	0.017947861
NM_032758.3	PHF5A	22	41855721	41864729	0.000839487	0.017988256
NM_005700.3	DPP3	11	66247484	66277130	0.000840275	0.017993869
NM_032379.3	SYTL2	11	85405318	85522184	0.000841218	0.018002809
NM_006048.2	UBE4B	1	10092890	10241297	0.000844308	0.018057632
NM_018024.1	WDYHV1	8	124428965	124479470	0.000846892	0.018101586
NM_031286.3	SH3BGRL3	1	26605667	26608007	0.000847713	0.018107824
NM_020920.2	CHD8	14	21853358	21905404	0.000850767	0.018140479
NM_152991.1	EED	11	85955586	85989781	0.000851753	0.018140479
NM_080599.1	UPF2	10	11962021	12085169	0.000851844	0.018140479
NM_022483.3	C5orf28	5	43444354	43483995	0.00085205	0.018140479
NM_016645.2	NGRN	15	90808891	90816463	0.000852263	0.018140479
NM_024091.2	FASTKD3	5	7859272	7869150	0.000852475	0.018140479
NM_032436.1	ZNF828	13	115079988	115092796	0.00085372	0.018140479
NM_020338.2	ZMIZ1	10	80828792	81076285	0.000853866	0.018140479
NM_199482.2	MOBKL3	2	198380295	198418423	0.000854013	0.018140479
NM_001005332.1	MAGED1	23	51546103	51645453	0.000856117	0.018162909
NM_182746.1	MCM4	8	48872745	48890720	0.00085613	0.018162909
NM_182849.1	CCNB1IP1	14	20779529	20801457	0.000857917	0.018178776
XR_019588.1	RPL18	19	49118591	49122433	0.000858382	0.018178776
NM_018303.4	EXOC2	6	485133	693111	0.000858926	0.018178776
NM_015446.3	AHCTF1	1	247002400	247095280	0.000859379	0.018178776
NM_032334.1	UTP23	8	117778742	117861702	0.000859534	0.018178776
NM_001099437.1	ZNF30	19	35417807	35436074	0.000861385	0.018206671
NM_006311.2	NCOR1	17	15934718	16119010	0.000869018	0.01833126
NM_004229.3	MED14	23	40507558	40595110	0.000869348	0.01833126
NM_019001.2	XRN1	3	142025449	142166904	0.000869526	0.01833126
NM_015050.2	FTSJD2	6	37400907	37450603	0.000869774	0.01833126
NM_003514.2	HIST1H2AM	6	27860477	27860963	0.000869963	0.01833126
NM_001035513.1	SDHC	1	161284047	161332984	0.000870494	0.01833126
NM_020701.1	ISY1	3	128806418	128880136	0.000873934	0.01839015
NM_198157.1	UBE2L3	22	21903739	21978323	0.000874365	0.01839015
NM_138410.2	CMTM7	3	32433163	32524559	0.000876035	0.018409135
NR_003028.1	TAF1D	11	93463114	93517557	0.000876344	0.018409135
NM_006586.3	CNPY3	6	42896938	42910989	0.000876938	0.018410314
NM_138375.1	CABLES1	18	20715727	20840434	0.0008777	0.018415026
NM_016037.2	UTP11L	1	38474930	38490496	0.00088078	0.018468312
NM_024665.3	TBL1XR1	3	176737143	176915261	0.000881603	0.018474253
NM_001034194.1	EXOSC9	4	122722472	122738176	0.000882595	0.018483719
NM_004965.6	HMGN1	21	40714241	40721573	0.000884458	0.018505039
NM_152262.2	ZNF439	19	11959576	11980306	0.000884835	0.018505039
NM_001031733.1	CALML4	15	68483043	68498417	0.000885235	0.018505039
NM_057175.3	NAA15	4	140222609	140341187	0.000887579	0.018542715
NM_001002259.1	CAPRIN2	12	30862488	30907448	0.000889213	0.018560896
NM_001006639.1	TCEAL1	23	102883632	102885881	0.000889534	0.018560896
NM_001294.1	CLPTM1	19	45458638	45496598	0.00089071	0.018574096
NM_001026.3	RPS24	10	79793518	79816570	0.000896264	0.018678537
NM_002266.2	KPNA2	17	66031848	66042969	0.000898807	0.018715513
NM_006974.2	ZNF33A	10	38299578	38356282	0.000899132	0.018715513
NM_024612.3	DHX40	17	57642886	57685702	0.000901316	0.018749565
NM_003664.3	AP3B1	5	77296349	77590579	0.000902919	0.018771504
NM_001077238.1	AC004410.1	19	2328629	2353207	0.000905734	0.018815057

NM_007249.4	KLF12	13	74260150	74708394	0.000906878	0.018815057
NM_207332.1	ERICH1	8	564746	688106	0.000907133	0.018815057
NM_001006936.1	TCEAL4	23	102831159	102842657	0.00090745	0.018815057
NM_181696.1	PRDX1	1	45976708	45988719	0.000907763	0.018815057
NM_053053.2	TADA1	1	166825747	166845564	0.000909403	0.018815891
NM_015878.4	AZIN1	8	103838585	103906092	0.000909577	0.018815891
NM_172251.1	MRPL54	19	3762665	3767562	0.000910377	0.018815891
NM_020409.2	MRPL47	3	179306073	179322442	0.000910398	0.018815891
NM_005548.1	KARS	16	75661622	75681577	0.000910955	0.018815891
NM_032959.4	POLR2J2	7	102277496	102312107	0.000911103	0.018815891
NM_022366.1	TFB2M	1	246703862	246729626	0.000916409	0.018914053
NM_022575.2	VPS16	20	2821349	2847378	0.000918642	0.018937623
NM_016284.3	CNOT1	16	58553855	58663750	0.000918676	0.018937623
NM_017999.4	RNF31	14	24616659	24629870	0.000919211	0.018937623
XM_944877.1	TOP1MT	8	144386554	144442149	0.000922103	0.018985779
NM_006265.1	RAD21	8	117858174	117887105	0.000922707	0.018986781
NM_014153.2	ZC3H7A	16	11844442	11891099	0.000926328	0.019049837
NM_001042631.1	SDHAF1	19	36486101	36487213	0.0009295	0.019103607
NM_033453.2	ITPA	20	3189514	3204516	0.000930846	0.019116912
NM_014939.2	TRAPPC8	18	29409136	29523091	0.000931265	0.019116912
NM_024122.2	APOO	23	23851470	23926051	0.000932392	0.019128564
NM_013387.3	UQCR10	22	30163358	30166402	0.00093591	0.019185701
NM_015565.1	LTN1	21	30300466	30365270	0.000936473	0.019185701
NM_007326.2	CYB5R3	22	43014814	43045574	0.000936878	0.019185701
NM_032430.1	BRSK1	19	55795327	55823901	0.000937419	0.019185701
NM_006337.3	MCRS1	12	49952088	49961911	0.000945607	0.0193417
NM_003134.4	SRP14	15	40327891	40331389	0.000948271	0.019376076
NM_013388.4	PREB	2	27353624	27357543	0.00094842	0.019376076
NM_003628.3	PKP4	2	159313476	159539391	0.000950149	0.01939983
NM_024576.3	OGFRL1	6	71998506	72011973	0.000954078	0.019439273
NM_001032279.1	RCE1	11	66610306	66614017	0.000954137	0.019439273
NM_181656.3	C17orf58	17	65987218	65989765	0.000954323	0.019439273
NM_015517.3	HINFP	11	118992288	119006752	0.000954783	0.019439273
NM_001010924.1	FAM171A1	10	15253642	15413097	0.00095526	0.019439273
NM_006913.2	RNF5	6	32146131	32151169	0.00095549	0.019439273
NM_005274.1	GNG5	1	84964008	84972248	0.000958005	0.019475811
NM_012257.3	HBP1	7	106809406	106842974	0.000958424	0.019475811
NM_003252.3	TIAL1	10	121334199	121356541	0.000960506	0.019506539
NM_032626.5	RBBP6	16	24550908	24584184	0.0009633	0.019551673
NM_004814.2	SNRNP40	1	31732417	31769629	0.00096766	0.019621486
NM_002812.3	PSMD8	19	38865176	38874464	0.000967886	0.019621486
NM_024120.3	C20orf7	20	13765596	13799067	0.000972994	0.019709429
NM_016516.2	VPS54	2	64120035	64246214	0.000973376	0.019709429
NM_004984.2	KIF5A	12	57943847	57978554	0.000974887	0.019728345
NM_017996.2	DET1	15	89055714	89089912	0.000976597	0.019751275
NM_001319.5	CSNK1G2	19	1941161	1981309	0.000978882	0.01978579
NM_006965.1	ZNF24	18	32912176	32924420	0.000980302	0.019802787
NM_018708.2	FEM1A	19	4791728	4795571	0.00098119	0.019809049
NM_006077.2	CBARA1	10	74127098	74385869	0.000982106	0.019815837
NM_152682.2	RWDD4	4	184560788	184580378	0.000984292	0.019848237
NM_138417.2	KTI12	1	52497775	52499488	0.000988887	0.019915708
NM_016495.2	TBC1D7	6	13305183	13328776	0.000989045	0.019915708
NM_014382.2	ATP2C1	3	130569439	130735556	0.000989383	0.019915708

NM_145859.1	PDCD10	3	167401086	167452727	0.000990055	0.019916234
NM_005358.4	LMO7	13	76194570	76434004	0.000990574	0.019916234
NM_181552.1	CUX1	7	101459240	101927250	0.000992033	0.019933872
NM_002576.3	PAK1	11	77032752	77185680	0.000995825	0.019998317
NM_001002924.1	AP3S1	5	115177178	115274332	0.000999433	0.020051443
NM_018706.5	DHTKD1	10	12110971	12165224	0.000999642	0.020051443
NM_030777.3	SLC2A10	20	45338126	45364965	0.001002537	0.020097727
NM_001042492.1	NF1	17	29421945	29705949	0.00100425	0.020120272
NM_006595.2	API5	11	43333505	43366079	0.001006841	0.020160387
NM_002141.4	HOXA4	7	27168126	27192217	0.001008639	0.020184578
NM_173618.1	INO80E	16	30007031	30017114	0.001010287	0.020186322
NM_001014838.1	CUTA	6	33384219	33386094	0.001010664	0.020186322
NM_014612.3	FAM120A	9	96214002	96328397	0.001011464	0.020186322
NM_014494.2	TNRC6A	16	24741016	24837548	0.001011676	0.020186322
NM_133642.2	LARGE	22	33561991	34318829	0.001014129	0.020206936
NM_014296.2	CAPN7	3	15247659	15294425	0.001014369	0.020206936
NM_176787.4	PIGN	18	59711460	59854289	0.001015495	0.020206936
NM_178126.2	FAM134C	17	40731540	40761402	0.001015717	0.020206936
NM_032828.2	ZNF587	19	58361228	58376485	0.001016002	0.020206936
NM_018064.2	AKIRIN2	6	88384790	88411927	0.001016252	0.020206936
NM_138769.1	RHOT2	16	718086	724174	0.001018752	0.020244879
NM_152405.2	JMY	5	78531954	78623038	0.001023761	0.020332626
NM_006286.1	TFDP2	3	141669989	141868386	0.001025513	0.020348473
NM_033360.2	KRAS	12	25358182	25403854	0.001025848	0.020348473
NM_001204.5	BMPR2	2	203241580	203432474	0.001026343	0.020348473
NM_003376.4	VEGFA	6	43737921	43754224	0.001027981	0.020349658
NM_015356.3	SCRIB	8	144873090	144897549	0.001028097	0.020349658
NM_022098.2	XPNPEP3	22	41253081	41368585	0.001028187	0.020349658
NM_004329.2	BMPR1A	10	88516396	88684945	0.00102939	0.020356646
NM_012382.2	TTC33	5	40714577	40756077	0.00102973	0.020356646
NM_018146.2	RNMTL1	17	685513	695749	0.00103418	0.020432808
NM_004169.3	SHMT1	17	18231187	18266856	0.001035242	0.020433567
NM_013367.2	ANAPC4	4	25378835	25420120	0.001035747	0.020433567
NM_015382.1	HECTD1	14	31569324	31676689	0.001036073	0.020433567
NM_002107.3	H3F3A	1	226249552	226259702	0.001037121	0.020433567
NM_006463.3	STAMBP	2	74056086	74100786	0.001037204	0.020433567
NM_018238.2	AGK	7	141250989	141355044	0.001038133	0.020435674
NM_006761.3	YWHAE	17	1247566	1303505	0.001038505	0.020435674
XR_015917.1	RPL24	3	101399935	101405626	0.001039959	0.020443926
NM_024598.2	C16orf57	16	58035305	58055522	0.001040266	0.020443926
NM_001003789.1	RABL2B	22	51205920	51222091	0.001040717	0.020443926
NM_018206.3	VPS35	16	46691891	46723104	0.001041871	0.020454859
NM_017670.1	OTUB1	11	63753325	63765892	0.001043434	0.020464843
NM_012245.2	SNW1	14	78183946	78227496	0.001043575	0.020464843
NM_015693.2	INTU	4	128544426	128637930	0.001044772	0.020476583
NM_015947.2	ASCC1	10	73856278	73980083	0.001049094	0.020549516
NM_021627.2	SENP2	3	185300284	185351339	0.00105298	0.020603163
NM_002408.3	MGAT2	14	50087489	50090198	0.001053037	0.020603163
NM_078628.1	MSL3	23	11776278	11793870	0.001055075	0.020619796
NM_001079864.1	TAX1BP1	7	27778950	27880938	0.00105556	0.020619796
NM_001418.3	EIF4G2	11	10818593	10830657	0.00105631	0.020619796
NM_004894.1	C14orf2	14	104378626	104387903	0.001056699	0.020619796
NM_030777.3	SLC2A10	20	45338126	45364965	0.0010569	0.020619796

AI434856	AL139008.5	9	33638033	33638492	0.001058076	0.020630976
NM_015679.1	TRUB2	9	131071382	131085021	0.001063654	0.020727918
NM_173794.2	FUNDC1	23	44382885	44402247	0.001064747	0.020737426
NM_020382.3	SETD8	12	123868320	123893905	0.001065937	0.02074879
NM_181740.1	LINS	15	101107119	101142445	0.001068794	0.020792571
NM_170722.1	NLRX1	11	119037277	119054725	0.001071121	0.020823852
NM_018046.3	AGGF1	5	76325076	76361059	0.001072172	0.020823852
NM_005197.2	FOXN3	14	89622529	89883454	0.001072228	0.020823852
NM_033375.4	MYO1C	17	1367480	1395995	0.001074526	0.02084485
NM_139322.1	ATRN	20	3451665	3631769	0.001074527	0.02084485
NM_017850.1	C1orf109	1	38147242	38157914	0.001076988	0.02087838
NM_024604.1	RPAP3	12	48057075	48099774	0.001077476	0.02087838
NM_079421.2	CDKN2D	19	10677139	10679655	0.00107857	0.020887752
NM_006083.3	IK	5	140026643	140042064	0.001079586	0.020895605
NM_025219.1	DNAJC5	20	62526518	62567384	0.00108076	0.020906506
NM_004849.1	ATG5	6	106632351	106773672	0.001081772	0.020914251
NM_031452.2	FAM103A1	15	83654959	83659809	0.001084026	0.020946002
NM_004099.4	STOM	9	124101355	124132531	0.001085223	0.020957244
NM_023071.1	SPATS2	12	49760688	49921207	0.001085833	0.020957244
NM_001078645.1	CDC16	13	115000362	115038198	0.001088583	0.020993462
NM_001007189.1	C5orf53	5	139505521	139508391	0.001089308	0.020993462
NM_018241.2	TMEM184C	4	148538534	148593195	0.00108955	0.020993462
NM_017621.2	ALKBH4	7	102096685	102105323	0.001091692	0.021022903
NM_004559.3	YBX1	1	43148098	43168020	0.001097929	0.021117916
NM_178526.1	SLC25A42	19	19174808	19223697	0.001098351	0.021117916
NM_024638.2	QTRTD1	3	113724680	113807269	0.001098477	0.021117916
NM_001081640.1	AC103686.1	8	48685669	48872743	0.001105415	0.021239362
NM_001556.1	IKBKB	8	42128820	42189973	0.001106319	0.021244802
NM_017667.2	CCDC132	7	92861653	92988338	0.001107702	0.021251826
NM_001010867.1	C1orf69	1	228353516	228369958	0.001107927	0.021251826
NM_014673.3	TTC35	8	109455830	109499145	0.001110449	0.021288275
NM_012433.2	SF3B1	2	198256698	198299815	0.001111299	0.021292631
NM_181291.1	WDR20	14	102606212	102690007	0.001114782	0.021347424
NM_020468.2	SNX14	6	86215214	86303874	0.001120629	0.021438008
NM_022163.2	MRPL46	15	89002707	89010650	0.001120766	0.021438008
NM_030624.1	KLHL15	23	24001837	24045303	0.001121411	0.02143838
NM_006874.2	ELF2	4	139949266	140098372	0.001123372	0.021463875
NM_033112.2	RRP36	6	42989383	42997335	0.001126849	0.021518295
NM_015282.1	CLASP1	2	122095352	122407163	0.001127623	0.021521061
NM_012215.2	MGEA5	10	103544200	103578222	0.001129291	0.021527256
NM_016028.4	SUV420H1	11	67922330	67981295	0.001129465	0.021527256
NM_017570.2	OPLAH	8	145106167	145115606	0.001129835	0.021527256
NM_016558.2	SCAND1	20	34541539	34547394	0.001132341	0.021546442
NM_006346.2	PIBF1	13	73356197	73590592	0.001132537	0.021546442
NM_001080416.1	MYBL1	8	67474414	67526482	0.001132792	0.021546442
BX640652	HMBOX1	8	28747911	28922281	0.001133522	0.021546442
NM_003825.2	SNAP23	15	42787801	42825259	0.00113399	0.021546442
NM_006955.1	ZNF33B	10	43069633	43134285	0.001137415	0.021597234
NM_001017406.1	S100PBP	1	33282368	33324476	0.001137926	0.021597234
NM_199344.2	SFT2D2	1	168195176	168212378	0.001140622	0.02161887
NM_181890.1	UBE2D3	4	103715540	103790053	0.001140891	0.02161887
NM_021061.1	ZNF250	8	146092748	146127553	0.001140961	0.02161887
NM_007100.2	ATP5I	4	666225	668127	0.001141728	0.021621427

NM_173471.2	SLC25A26	3	66119285	66438130	0.001144184	0.021655956
NM_014294.4	TRAM1	8	71485677	71520622	0.001146437	0.021665286
NM_033301.1	RPL8	8	146015150	146017972	0.00114653	0.021665286
NM_006929.4	SKIV2L	6	31926581	31937532	0.001146576	0.021665286
NM_016021.2	UBE2J1	6	90039279	90062567	0.00114799	0.021680024
NM_015278.3	SASH1	6	148593440	148873186	0.001151287	0.02172052
NM_002540.3	ODF2	9	131218336	131263239	0.001151403	0.02172052
NM_006268.3	DPF2	11	65101225	65120720	0.001153224	0.021742874
NM_130434.3	DPP8	15	65734801	65810042	0.001154711	0.021758922
NM_015420.5	DCAF13	8	104426942	104455681	0.001156637	0.021783229
NM_032333.4	C10orf58	10	82167585	82192753	0.001159157	0.021818682
NM_002673.3	PLXNB1	3	48445261	48471594	0.001160028	0.021823072
NM_173614.2	NOMO1	16	14927538	14990017	0.001161826	0.021838126
NM_001032391.1	LCMT1	16	25123047	25189551	0.001162485	0.021838126
NM_015346.2	ZFYVE26	14	68194091	68283306	0.001162743	0.021838126
NM_020151.2	STARD7	2	96850597	96874573	0.001165227	0.021870658
NM_005858.2	AKAP8	19	15464341	15490603	0.001165753	0.021870658
NM_144999.2	LRRC45	17	79981280	79989024	0.001166468	0.021872082
NM_017974.3	ATG16L1	2	234118697	234204320	0.001168765	0.021895531
NM_006294.2	UQCRB	8	97238148	97247862	0.001168999	0.021895531
NM_002970.1	SAT1	23	23801290	23804343	0.001170046	0.021903164
NM_004261.3	RP4-604K5.1	1	87246147	87380107	0.001174901	0.021982026
NM_000681.2	ADRA2A	10	112836790	112840658	0.001177907	0.022026223
NM_003010.2	MAP2K4	17	11924141	12047140	0.001179899	0.022045677
NM_145160.1	MAP2K5	15	67835021	68099461	0.001180236	0.022045677
NM_182648.1	BAZ1A	14	35221938	35344853	0.001181321	0.022053898
NM_003255.4	TIMP2	17	76849059	76921469	0.001182779	0.022069075
NM_005067.5	SIAH2	3	150458914	150481264	0.00118748	0.022144722
NM_018448.2	CAND1	12	67663061	67708388	0.001188469	0.022151085
NM_205767.1	C19orf70	19	5678433	5680911	0.001192461	0.022203075
NM_138962.2	MSI2	17	55333931	55757299	0.001192556	0.022203075
NM_013442.1	STOML2	9	35099888	35103154	0.001195718	0.022249853
NM_080748.2	ROMO1	20	34287194	34288906	0.001196501	0.022252311
NM_001042535.1	AGAP3	7	150782918	150841523	0.001197204	0.022253293
NM_003331.3	TYK2	19	10461205	10491352	0.001198354	0.022262577
NM_006023.1	CDC123	10	12237964	12292588	0.001199655	0.022263402
NM_001641.2	APEX1	14	20923290	20925933	0.001199851	0.022263402
NM_020381.2	PDSS2	6	107473761	107780779	0.001200764	0.022263402
NM_018999.1	FAM190B	10	86088342	86278276	0.001201001	0.022263402
NM_017722.2	TRMT1	19	13215716	13227563	0.001203262	0.022277793
NM_016467.3	ORMDL1	2	190635049	190649097	0.001203298	0.022277793
NM_016126.1	HSPB11	1	54387234	54411975	0.00120373	0.022277793
NM_015127.3	GPSM2	1	109417972	109473044	0.001210247	0.022379172
NM_003971.3	SPAG9	17	49039535	49198226	0.001210516	0.022379172
NM_023071.3	SPATS2	12	49760688	49921207	0.001213862	0.022428911
NM_032816.3	CCDC123	19	33369908	33462869	0.001215351	0.022444304
NM_020162.2	DHX33	17	5344232	5372356	0.001224301	0.022593686
NM_001029998.2	SLC10A7	4	147175127	147443123	0.00122476	0.022593686
NM_001003701.1	ATP5J	21	27088815	27107984	0.001225826	0.022601173
NM_001080826.1	AC068353.1	8	8175258	8244008	0.001229754	0.022651829
NM_138785.1	C6orf72	6	149887410	149912884	0.001229898	0.022651829
NM_005087.2	FXR1	3	180585929	180694950	0.001233524	0.022706392
NM_018840.2	C20orf24	20	35234137	35240960	0.001234525	0.02271021

NM_007013.3	WWP1	8	87354967	87490649	0.001235516	0.02271021
NM_032527.3	ZGPAT	20	62338794	62370456	0.001235722	0.02271021
NM_015607.2	C1orf77	1	153606525	153618782	0.001238056	0.022737614
NM_004707.2	ATG12	5	115163897	115177555	0.001238542	0.022737614
NM_015447.1	CAMSAP1	9	138700333	138799074	0.001240859	0.022767935
NM_172373.2	ELF1	13	41506056	41635576	0.001244023	0.022798989
NM_017953.2	ZNHIT6	1	86118491	86174101	0.001244678	0.022798989
NM_171829.1	KCNMB3	3	178957530	178984790	0.001244796	0.022798989
NM_024646.1	ZYG11B	1	53192131	53293014	0.001245217	0.022798989
NM_024814.1	CBLL1	7	107384142	107400131	0.001246638	0.022806072
NM_001126.2	ADSS	1	244571796	244615436	0.001246936	0.022806072
NM_033418.1	C1orf156	1	169761670	169764107	0.001248626	0.022823178
NM_003449.3	TRIM26	6	30152232	30181204	0.001249205	0.022823178
NM_002096.1	GTF2F1	19	6379580	6393291	0.001250351	0.022831911
XM_928464.1	ATXN1L	16	71879899	71891231	0.001252817	0.022864751
NM_001494.2	GDI2	10	5807186	5884095	0.001254253	0.022872302
NM_004763.3	ITGB1BP1	2	9543604	9563676	0.001255101	0.022872302
NM_005896.2	IDH1	2	209100951	209130798	0.001255236	0.022872302
NM_005973.4	PRCC	1	156720402	156770607	0.001256694	0.022886678
NM_001040097.1	MOSPD3	7	100209725	100213007	0.001258548	0.022908248
NM_212461.1	PRKAG1	12	49396058	49412592	0.001262653	0.02296526
NM_080670.2	SLC35A4	5	139944041	139948688	0.001263022	0.02296526
NM_002107.3	H3F3A	1	226249552	226259702	0.00126438	0.022977738
NM_031885.2	BBS2	16	56518366	56554195	0.001266253	0.02299957
NM_001080452.1	GPR108	19	6729925	6737633	0.001274079	0.023129436
NM_001697.2	AP000304.12	21	34956993	35284635	0.001276613	0.023153496
NM_003415.1	ZNF268	12	133707214	133781730	0.001277714	0.023153496
NM_014066.3	COMMD5	8	146066427	146079121	0.001277771	0.023153496
NM_021145.2	DMTF1	7	86781677	86825648	0.001278111	0.023153496
NM_206831.1	DPH3	3	16299485	16306479	0.001279567	0.023167613
NM_002790.2	PSMA5	1	109944470	109969049	0.001281065	0.02317671
NM_016451.3	COPB1	11	14464986	14521573	0.001282324	0.023180734
NM_004379.3	CREB1	2	208394461	208468155	0.0012839	0.023188102
NM_017905.3	TMCO3	13	114145310	114204542	0.001284087	0.023188102
XM_001124642.1	PARG	10	51026325	51130715	0.001286458	0.023209091
NM_006699.3	MAN1A2	1	117910071	118071494	0.001286606	0.023209091
NM_005051.1	QARS	3	49133365	49142534	0.001288371	0.023228685
NM_003542.3	HIST1H4C	6	26104104	26104518	0.001289608	0.023238742
NM_133635.4	POFUT2	21	46683843	46707813	0.001291643	0.023263175
NM_031298.2	TMEM93	17	3572090	3572962	0.001294276	0.023298322
NM_004044.4	ATIC	2	216176540	216214487	0.00129567	0.023298649
NM_020677.2	NMRAL1	16	4511695	4524896	0.001296198	0.023298649
XM_926110.1	AC145291.1	8	145317003	145331186	0.001296336	0.023298649
NM_020854.3	KIAA1468	18	59854507	59974355	0.001298597	0.023316503
NM_003365.2	UQCRC1	3	48636435	48648409	0.001299671	0.023316503
NM_005510.3	DOM3Z	6	31937587	31940069	0.001299942	0.023316503
NM_005787.4	ALG3	3	183960089	183967336	0.001300524	0.023316503
XR_016147.1	SSBP3	1	54692190	54879152	0.00130157	0.023316503
NM_015608.2	C10orf137	10	127408084	127452712	0.001301636	0.023316503
NM_016127.4	TMEM66	8	29920528	29940723	0.001303052	0.023316503
NM_001010851.2	ZNF766	19	52772824	52795976	0.001303125	0.023316503
NM_006925.3	SRSF5	14	70233829	70238722	0.001303462	0.023316503
NM_001080537.1	SNTN	3	63638344	63664696	0.001309916	0.023419717

NM_003000.2	SDHB	1	17345217	17380665	0.001310657	0.023420728
NM_018209.2	ARFGAP1	20	61904147	61921145	0.001314782	0.023477779
NM_144628.2	TBC1D20	20	416124	443197	0.001315352	0.023477779
NM_004831.3	MED26	19	16685718	16739015	0.001315908	0.023477779
NM_199141.1	CARM1	19	10982253	11033448	0.001317599	0.023478096
NM_003310.1	TSSC1	2	3192696	3381653	0.001317647	0.023478096
NM_001042697.1	ZSWIM7	17	15879874	15903031	0.001317984	0.023478096
NM_006713.2	SUB1	5	32531739	32604185	0.001319351	0.023490214
NM_001008938.1	CKAP5	11	46764598	46867847	0.001320883	0.02350526
NM_024715.3	TXNDC15	5	134209460	134237323	0.001322037	0.023513566
NM_015514.1	NGDN	14	23924714	23947402	0.001327255	0.023594109
NM_016407.2	C20orf43	20	55043647	55093943	0.001329444	0.023620751
NM_004140.3	LLGL1	17	18128901	18148189	0.001332612	0.023664763
NM_177533.2	NUDT14	14	105639275	105647641	0.001335754	0.023708241
NM_003592.2	CUL1	7	148395006	148498201	0.001343135	0.023818831
NM_001077664.1	URGCP	7	43915493	43966010	0.001343376	0.023818831
NM_014183.2	DYNLRB1	20	33104214	33128762	0.001345128	0.02383754
NM_000937.2	POLR2A	17	7387866	7417933	0.001347223	0.023862296
NM_004295.3	TRAF4	17	27071036	27077974	0.00135035	0.023905312
NM_005955.2	MTF1	1	38279851	38325292	0.001352194	0.023925577
NM_014883.2	FAM13A	4	89647106	90032549	0.001353972	0.023937894
NM_012154.2	EIF2C2	8	141541264	141645718	0.001354289	0.023937894
NM_018154.2	ASF1B	19	14230321	14247768	0.001355451	0.023946069
NM_032477.1	MRPL41	9	140445651	140447007	0.001357002	0.0239611
NM_018715.1	RCC2	1	17733256	17766220	0.001362332	0.024034419
NM_025256.4	EHMT2	6	31847536	31865464	0.001362559	0.024034419
NM_006435.2	IFITM2	11	307631	315272	0.001363648	0.0240381
NM_024622.2	FASTKD1	2	170386259	170430424	0.00136456	0.0240381
NM_001099222.1	IFT74	9	26947037	27062928	0.001364875	0.0240381
NM_001008491.1	SEPT2	2	242254515	242293442	0.001366743	0.024058617
NM_007367.2	RALY	20	32581452	32696114	0.00137093	0.024119907
NM_203351.1	MAP3K3	17	61699801	61773667	0.001373101	0.024145688
NM_015633.1	FGFR1OP2	12	27091316	27118857	0.001374168	0.024152046
NM_004068.3	AP2M1	3	183892477	183901879	0.001374931	0.024153043
NM_024718.2	C9orf86	9	139694818	139735639	0.001377837	0.02419068
NM_001035235.2	SRA1	5	139929752	139937895	0.001378567	0.02419068
NM_015246.1	MGRN1	16	4674826	4740975	0.001379194	0.02419068
NM_016417.2	GLRX5	14	96001323	96011055	0.001380734	0.024205283
NM_004779.4	CNOT8	5	154237113	154256353	0.001381718	0.02420612
NM_001001520.1	AC011498.1	19	4472255	4502213	0.001382196	0.02420612
XM_001131304.1	DHRS4L1	14	24517356	24520580	0.001383624	0.024218724
NM_002896.2	RP11-658F2.1	11	66384097	66413944	0.001385338	0.02423633
NM_138369.1	BOD1	5	173034517	173043663	0.001386313	0.024240996
NM_016069.8	PAM16	16	4390252	4401373	0.001388489	0.024266653
NM_173569.2	UBN2	7	138912200	138992982	0.00139274	0.024323089
NM_207584.1	IFNAR2	21	34602206	34637969	0.001393632	0.024323089
NM_002676.1	PMM1	22	41972898	41985894	0.001394292	0.024323089
NM_012236.2	SCMH1	1	41492874	41707826	0.001394562	0.024323089
NM_203385.1	RNH1	11	494512	507300	0.001397505	0.024353524
NM_006572.3	GNA13	17	63005407	63052920	0.00139773	0.024353524
NM_172002.3	HSCB	22	29138019	29153503	0.001399952	0.024367724
NM_006295.2	VARS	6	31745295	31763730	0.001400737	0.024367724
NM_003610.3	RAE1	20	55926066	55954267	0.001401832	0.024367724

NM_177926.1	CSRP2BP	20	18118499	18169031	0.001402323	0.024367724
NM_013236.2	ATXN10	22	46067679	46241187	0.001402615	0.024367724
NM_001040443.1	PHF11	13	50069746	50103123	0.001402818	0.024367724
NM_016334.2	GPR89B	1	147400506	147465755	0.001403998	0.024375852
NM_130395.1	WRNIP1	6	2765648	2786927	0.001405981	0.024397899
NM_015483.1	KBTBD2	7	32907784	32933743	0.001410482	0.024463606
NM_004886.3	APBA3	19	3750771	3761673	0.001413653	0.02450619
NM_182533.2	C1orf86	1	2115903	2144159	0.001415083	0.024509945
NM_024884.1	L2HGDH	14	50704281	50778947	0.001415735	0.024509945
NM_176787.3	PIGN	18	59711460	59854289	0.001416432	0.024509945
NM_025182.2	KIAA1539	9	35104109	35115999	0.001416735	0.024509945
NM_181836.3	TMED7	5	114949205	114968689	0.001418308	0.024524762
NM_002088.3	GRIK5	19	42502477	42569957	0.001422017	0.024575148
NM_003972.2	BTAF1	10	93683822	93790082	0.001422937	0.024575148
NM_022780.2	RMND5A	2	86947296	87005164	0.001423482	0.024575148
NM_001378.1	DYNC1I2	2	172543919	172604930	0.001424741	0.024575148
NM_133452.1	RAVER1	19	10426895	10444314	0.001424813	0.024575148
NM_004870.1	MPDU1	17	7486974	7495790	0.001426744	0.024596066
NM_006098.4	GNB2L1	5	180663909	180675096	0.001429392	0.024629303
NM_022757.3	CCDC14	3	123616152	123680564	0.001430676	0.024639013
NM_018947.4	CYCS	7	25159710	25164980	0.00143158	0.02464218
NM_007292.4	ACOX1	17	73937596	73975444	0.001439041	0.024752608
NM_003979.3	GPRC5A	12	13043956	13066598	0.001439442	0.024752608
NM_003133.2	SRP9	1	225965531	225978168	0.001442029	0.024779108
NM_001079864.1	TAX1BP1	7	27778950	27880938	0.001442431	0.024779108
NM_006136.2	CAPZA2	7	116451124	116559315	0.001444222	0.024797423
NM_017747.1	ANKHD1	5	139781399	139929163	0.001450098	0.024884799
NM_002623.3	PFDN4	20	52824386	52844591	0.00145158	0.024884799
NM_002950.3	RPN1	3	128338817	128399918	0.00145197	0.024884799
NM_024836.1	ZNF672	1	249132409	249143716	0.001452219	0.024884799
NM_001987.4	ETV6	12	11802788	12048321	0.001455011	0.024920154
NM_006429.2	CCT7	2	73460548	73480132	0.001455764	0.024920579
NM_005762.2	TRIM28	19	59055836	59062082	0.001458034	0.024936248
NM_022079.2	HERC4	10	69681665	69835105	0.001458137	0.024936248
NM_003139.2	SRPR	11	126132833	126139039	0.001460052	0.024956535
NM_020773.1	TBC1D14	4	6910969	7034845	0.001465418	0.025035749
NM_018356.1	C5orf22	5	31532373	31555165	0.001466247	0.025037405
NM_020143.2	PNO1	2	68384976	68403370	0.001468693	0.025062759
NM_002669.2	PLRG1	4	155456158	155471587	0.001469995	0.025062759
NM_001032291.1	PSRC1	1	109822176	109825808	0.001470076	0.025062759
NM_178564.2	NRBP2	8	144915764	144924200	0.001470661	0.025062759
NM_001025593.1	ARFIP1	4	153701089	153839615	0.001471522	0.025064941
NM_024814.1	CBLL1	7	107384142	107400131	0.001473539	0.025073499
NM_001079866.1	BCS1L	2	219523487	219528166	0.001473829	0.025073499
NM_014803.3	ZNF518A	10	97889492	97923517	0.001474542	0.025073499
NM_021034.2	IFITM3	11	319669	321340	0.001474955	0.025073499
NM_152743.2	C7orf27	7	2577511	2595361	0.001477843	0.025110131
NM_001905.1	CTPS	1	41445007	41478235	0.001480616	0.025127021
NM_004330.1	BNIP2	15	59951345	59981733	0.001482042	0.025127021
NM_144988.2	ALG14	1	95448297	95538501	0.001482344	0.025127021
XM_942289.1	RSPH10B2	7	6793740	6838996	0.001482587	0.025127021
NM_005859.3	PURA	5	139487362	139496321	0.001482965	0.025127021
NM_018918.2	PCDHGA1	5	140710204	140892546	0.001483753	0.025127021

NM_016478.3	ZC3HC1	7	129658126	129691291	0.001483977	0.025127021
NM_001008800.1	CCT3	1	156278752	156337664	0.001485841	0.025146132
NM_002441.3	MSH5	6	31707725	31730611	0.001491325	0.025226462
NM_005999.2	TSNAX	1	231664287	231702270	0.00149259	0.025229018
NM_031946.3	AGAP3	7	150782918	150841523	0.00149295	0.025229018
NR_024273.1	SEC13	3	10334815	10362862	0.001493958	0.02523359
NM_031913.2	ESYT3	3	138153415	138200528	0.001497756	0.025264387
NM_007255.1	B4GALT7	5	177027101	177037348	0.001498141	0.025264387
NM_080653.3	ATP6V1E2	2	46717889	46769696	0.001498743	0.025264387
NM_017515.3	SLC35F2	11	107661717	107799019	0.00149973	0.025264387
NM_014881.2	DCLRE1A	10	115594488	115614142	0.001499921	0.025264387
NM_012264.3	TMEM184B	22	38615298	38669040	0.001500932	0.025264387
NM_025077.2	TOE1	1	45805342	45809647	0.00150095	0.025264387
NM_016626.3	MEX3C	18	48700920	48723690	0.00150271	0.025281575
NM_007111.3	TFDP1	13	114239036	114295504	0.0015037	0.025285802
NM_182513.1	SPC24	19	11256170	11266484	0.001505062	0.025296276
NM_014372.3	RNF11	1	51701943	51739127	0.001509571	0.025354159
NM_015895.3	GMNN	6	24775159	24786327	0.001509988	0.025354159
NM_001003725.1	DCAF7	17	61627822	61671639	0.001511902	0.025373849
NM_178545.2	TMEM52	1	1849029	1850712	0.001513259	0.025384184
NM_017892.3	PRPF40A	2	153508107	153574511	0.001514321	0.025389547
NM_012325.1	MAPRE1	20	31407699	31438211	0.00151507	0.025389669
NM_003859.1	DPM1	20	49551404	49575092	0.001517067	0.025410686
NM_152268.2	PARS2	1	55222571	55230187	0.001525013	0.025531289
NM_003850.1	SUCLA2	13	48510622	48575462	0.001529838	0.025599533
NM_017867.1	C4orf27	4	170650616	170679104	0.00153169	0.025618007
NM_001040431.1	CCDC56	17	40949653	40950719	0.001534797	0.025657435
NM_002802.2	PSMC1	14	90722894	90738966	0.001539495	0.025723397
NM_021121.2	EEF1B2	2	207024309	207027652	0.001543068	0.025770508
NM_001025190.1	MSLNL	16	819428	833370	0.001547475	0.025826089
NM_007027.2	TOPBP1	3	133317019	133380737	0.001547905	0.025826089
NM_139135.2	ARID1A	1	27022524	27108595	0.001549648	0.025831644
NM_175902.3	C17orf101	17	80350100	80376462	0.001549748	0.025831644
NM_032334.1	UTP23	8	117778742	117861702	0.001550925	0.025838677
NM_020228.2	PRDM10	11	129769601	129872730	0.001555542	0.025893996
NM_005645.3	TAF13	1	109605121	109618624	0.001556928	0.025893996
NM_020748.1	INTS2	17	59942728	60005377	0.001558079	0.025893996
NM_001065.2	TNFRSF1A	12	6437924	6451261	0.001558099	0.025893996
NM_032350.4	C7orf50	7	1036623	1177896	0.001558561	0.025893996
NM_001077523.1	AP3D1	19	2100993	2151556	0.001558785	0.025893996
NM_024588.3	C22orf46	22	42084943	42094140	0.001563868	0.025965817
NM_032151.3	PCBD2	5	134240596	134343649	0.001565813	0.025975511
NM_020754.1	ARHGAP31	3	119013220	119139561	0.001566143	0.025975511
NM_018090.3	NECAP2	1	16767167	16786573	0.001567739	0.025975511
NM_004331.2	BNIP3L	8	26240414	26363152	0.001568327	0.025975511
NM_130437.2	DYRK1A	21	38739236	38887680	0.001569008	0.025975511
NM_004396.2	DDX5	17	62495740	62502407	0.001569472	0.025975511
NM_001677.3	ATP1B1	1	169074935	169101960	0.001571128	0.025975511
NM_016041.3	DERL2	17	5374571	5389537	0.001571344	0.025975511
NM_053052.2	SNAP47	1	227916240	227968927	0.00157159	0.025975511
NM_001006610.1	SIAH1	16	48394442	48419361	0.001572123	0.025975511
NM_001009993.2	FAM168B	2	131805449	131851033	0.001572995	0.025975511
NM_002412.2	MGMT	10	131265448	131566271	0.00157356	0.025975511

NM_004689.3	MTA1	14	105886157	105937066	0.001575284	0.025989245
NM_020896.2	OSBPL5	11	3108346	3187969	0.001575911	0.025989245
NM_170783.1	ZNRD1	6	30026676	30032686	0.00157923	0.026004754
NM_004627.2	WRB	21	40752170	40800454	0.001579506	0.026004754
NM_018029.3	AC103559.1	3	73110810	73112488	0.001579921	0.026004754
NM_015043.3	TBC1D9B	5	179289066	179334859	0.001580429	0.026004754
NM_144566.1	ZNF700	19	12035900	12061588	0.001580725	0.026004754
NM_016059.3	PPIL1	6	36822603	36842800	0.001581411	0.026004754
NM_024556.2	FAM118B	11	126081309	126132881	0.001585261	0.026052328
NM_015721.2	GEMIN4	17	647661	655501	0.001586551	0.026052328
NM_006540.2	NCOA2	8	71015826	71316040	0.001586588	0.026052328
NM_020186.1	ACN9	7	96745902	96811075	0.001590482	0.026095784
NM_004206.2	SEC22C	3	42589461	42642572	0.00159076	0.026095784
NM_016487.2	C6orf203	6	107349407	107372546	0.001604451	0.026306335
NM_032014.2	MRPS24	7	43906157	43909492	0.001605683	0.026306335
NM_139279.3	MCFD2	2	47129013	47168994	0.001605901	0.026306335
NM_016472.3	C14orf129	14	96829863	96853623	0.001607279	0.026316313
NM_016617.1	UFM1	13	38923986	38937140	0.001609005	0.026331971
NM_015938.2	NMD3	3	160822484	160971320	0.001610907	0.026350503
NM_023039.2	ANKRA2	5	72848160	72861511	0.001612328	0.026361133
NM_004949.2	DSC2	18	28645940	28682388	0.001614113	0.026368582
NM_001009566.1	CLSTN1	1	9789084	9884584	0.001614324	0.026368582
NM_032558.2	HIATL1	9	97136833	97223324	0.001617105	0.026401394
NM_001636.2	SLC25A6	23	1505045	1511626	0.001618596	0.026410351
NM_199418.2	PRCP	11	82534544	82681626	0.00161934	0.026410351
NM_017778.2	WHSC1L1	8	38132544	38239790	0.001619969	0.026410351
NM_020365.2	EIF2B3	1	45316450	45452282	0.001622394	0.026437291
NM_006406.1	PRDX4	23	23682379	23704516	0.00162431	0.026455916
NM_001001433.1	STX16	20	57226328	57254582	0.001626234	0.02647465
NM_138775.1	ALKBH8	11	107373452	107436472	0.001629318	0.026512257
NR_015339.1	C8orf83	8	93895758	94029901	0.00163398	0.026575477
NM_004663.3	RAB11A	15	66161789	66184329	0.001637411	0.026600476
NM_001083335.1	ZFP112	19	44830706	44860856	0.001637566	0.026600476
AB074172	IL6ST	5	55230923	55290772	0.001637849	0.026600476
NM_016151.2	TAOK2	16	29985222	30003582	0.001645025	0.02670435
NM_005642.2	TAF7	5	140698058	140700351	0.001645857	0.026705179
NM_016340.4	RAPGEF6	5	130759614	130970929	0.001647108	0.026712812
NM_001418.3	EIF4G2	11	10818593	10830657	0.001648569	0.02671544
NM_032852.2	ATG4C	1	63249806	63331184	0.00164939	0.02671544
NM_024045.1	DDX50	10	70661034	70706603	0.001649612	0.02671544
NM_007311.3	TSPO	22	43547520	43559248	0.001650934	0.026724205
NM_000999.2	RPL38	17	72199721	72206676	0.001654578	0.026770518
NM_032776.1	JMJD1C	10	64926981	65225722	0.001660187	0.026848579
NM_024580.5	EFTUD1	15	82422561	82555104	0.001663064	0.026872499
NM_201628.1	RP1-21O18.1	1	14925200	15444539	0.001663237	0.026872499
NM_012075.1	NPRL3	16	134273	188859	0.001667323	0.026925804
NM_152608.3	C1orf55	1	226170403	226187066	0.001669549	0.026946372
NM_002336.2	LRP6	12	12268963	12419811	0.001670171	0.026946372
NM_133636.2	HELQ	4	84328496	84377022	0.001678063	0.027060941
NM_181656.3	C17orf58	17	65987218	65989765	0.00168002	0.027066835
NM_001006937.1	TCEAL4	23	102831159	102842657	0.001680388	0.027066835
NM_003589.2	CUL4A	13	113863086	113919399	0.001680849	0.027066835
NM_000123.2	ERCC5	13	103497194	103528345	0.001682915	0.027066835

NM_001135865.1	RP11-368J21.2	16	22524843	22547842	0.001682932	0.027066835
NM_153812.1	PHF13	1	6673745	6684093	0.001683174	0.027066835
NM_152991.1	EED	11	85955586	85989781	0.001686582	0.027108888
NM_022473.1	ZFP106	15	42705022	42749730	0.001693773	0.027211698
NM_138820.2	ARL10	5	175792471	175828866	0.001697751	0.027262812
NM_016091.2	EIF3L	22	38244875	38285414	0.001699307	0.027274987
NM_007081.2	RABL2B	22	51205920	51222091	0.001704473	0.027332835
NM_005776.2	CNIH	14	54893646	54908149	0.001704939	0.027332835
XM_001132317.1	AC073346.2	7	112756773	112758668	0.001706779	0.027332835
NM_002803.2	PSMC2	7	102984701	103009842	0.001706849	0.027332835
NM_012405.3	ICMT	1	6281252	6296032	0.001706985	0.027332835
NM_018337.2	ZNF444	19	56652556	56672261	0.00170775	0.027332835
NM_001040065.1	FAM75A1	9	39355666	39361956	0.001708502	0.027332835
NM_012330.2	MYST4	10	76585340	76792380	0.001715139	0.027415715
NM_005035.3	POLRMT	19	617223	633568	0.001715301	0.027415715
NM_004294.2	MTRF1	13	41790516	41837713	0.001716086	0.027415715
NM_152613.1	WBP2NL	22	42394729	42454460	0.001721413	0.02748758
NM_006002.3	UCHL3	13	76123619	76180085	0.001722542	0.02748758
NM_002793.2	PSMB1	6	170844205	170862429	0.001723805	0.02748758
NM_006827.5	TMED10	14	75598173	75643349	0.001724226	0.02748758
NM_001080411.1	ZNF433	19	12125532	12146556	0.001725902	0.02748758
NM_002372.2	MAN2A1	5	109025067	109205326	0.001727385	0.02748758
NM_005724.4	TSPAN3	15	77336359	77363570	0.001728345	0.02748758
NM_003477.1	PDHX	11	34937376	35042138	0.001729321	0.02748758
NM_015456.2	COBRA1	9	140149625	140167998	0.001729942	0.02748758
NM_007347.3	AP4E1	15	51200946	51298097	0.001730556	0.02748758
XM_001129650.1	OPLAH	8	145106167	145115606	0.001730725	0.02748758
NM_176806.2	MOCS2	5	52391512	52405893	0.001730973	0.02748758
NM_003821.5	RIPK2	8	90769975	90803291	0.001731027	0.02748758
NM_003071.3	HLTF	3	148747914	148804341	0.001731865	0.027488142
NM_012247.3	SEPHS1	10	13359424	13390297	0.001732997	0.02749335
NM_181836.3	TMED7	5	114949205	114968689	0.001734669	0.027496286
NM_001001683.2	MED11	17	4634708	4636888	0.001734789	0.027496286
NM_005466.2	MED6	14	71050957	71067384	0.001736334	0.027504167
NM_152416.2	C8orf38	8	95907995	96128683	0.001736894	0.027504167
NM_012088.2	PGLS	19	17622432	17632095	0.001738391	0.027515141
NM_198566.1	C5orf34	5	43486803	43515247	0.001743622	0.027573118
NM_024331.3	TTPAL	20	43104526	43123244	0.001743665	0.027573118
NM_019088.2	PAF1	19	39876275	39881746	0.00174448	0.027573265
NM_018566.3	YOD1	1	207217194	207226325	0.001747623	0.02760076
NM_198467.1	RSBN1L	7	77325760	77409008	0.001749126	0.02760076
NM_005301.2	GPR35	2	241544848	241570676	0.001750093	0.02760076
NM_001039708.1	SDCCAG3	9	139296377	139305061	0.001750222	0.02760076
NM_017646.3	TRIT1	1	40306708	40349183	0.00175091	0.02760076
NM_001099672.1	C8orf59	8	86126311	86132650	0.001751484	0.02760076
NM_015627.2	LDLRAP1	1	25870071	25895377	0.001751866	0.02760076
NM_012263.3	TTLL1	22	43414609	43485434	0.001753699	0.027616929
NM_139207.1	NAP1L1	12	76438682	76478738	0.001756061	0.027623084
NM_014016.2	SACM1L	3	45730548	45786901	0.001756164	0.027623084
NM_019022.3	TMX3	18	66340927	66382362	0.001756512	0.027623084
NM_004804.2	CIAO1	2	96931870	96939087	0.001760617	0.02766388
NM_016052.3	RRP15	1	218458629	218511325	0.001760723	0.02766388
NM_177423.1	PPFIA1	11	70116806	70230509	0.001762635	0.027681221

NM_173511.2	FAM117B	2	203499911	203634480	0.00176348	0.027681786
BX093678	AL139008.5	9	33638033	33638492	0.001767938	0.027739029
NM_002788.2	PSMA3	14	58711549	58738725	0.001770364	0.027764374
NM_012421.2	RLF	1	40627045	40706593	0.001771689	0.02777243
NM_001610.1	ACP2	11	47260853	47270457	0.00177599	0.027827094
NM_005109.2	OXSR1	3	38206580	38296979	0.001780735	0.027883926
NM_014463.1	LSM3	3	14219858	14242619	0.001781247	0.027883926
NM_030768.2	ILKAP	2	239079042	239112370	0.001785804	0.027942486
NM_032870.1	SFRS18	6	99845927	99873207	0.001788417	0.027965031
NM_015057.3	MYCBP2	13	77618792	77901185	0.001788879	0.027965031
NM_001011670.1	CHCHD7	8	57124245	57131357	0.001790719	0.027975614
NM_018050.2	MANSC1	12	12482218	12503169	0.001791191	0.027975614
NM_006036.2	PREPL	2	44543420	44588945	0.001795991	0.02803719
NM_018446.2	GLT8D1	3	52728505	52740048	0.001796773	0.02803719
NM_000365.4	TPI1	12	6976283	6980112	0.001798277	0.028047872
NM_018173.2	PLEKHG6	12	6419602	6437649	0.001809817	0.028214999
NM_001440.2	EXTL3	8	28457986	28613116	0.001813356	0.028257301
NM_004671.2	PIAS2	18	44392060	44497466	0.001814447	0.028261442
NM_020337.1	ANKRD50	4	125585207	125633887	0.001816453	0.02827889
NM_007216.3	HPS5	11	18300217	18343745	0.001817905	0.02827889
NM_024871.1	MAP6D1	3	183533664	183543382	0.001818047	0.02827889
NM_153374.1	LYSMD2	15	52015208	52043650	0.001821277	0.028305849
NM_018321.3	BRIX1	5	34915481	34926101	0.001821434	0.028305849
NM_181706.4	DNAJC24	11	31391387	31453396	0.001822533	0.028310071
NM_001008938.1	CKAP5	11	46764598	46867847	0.001825358	0.028331878
NM_002292.3	LAMB2	3	49158547	49170599	0.001825593	0.028331878
NM_015274.1	MAN2B2	4	6576902	6625089	0.001828625	0.028366074
NM_005166.3	APLP1	19	36359401	36370699	0.001829525	0.028367168
NM_004404.3	SEPT2	2	242254515	242293442	0.001832467	0.028393745
NM_007216.3	HPS5	11	18300217	18343745	0.001833501	0.028393745
NM_006496.1	GNAI3	1	110091233	110136975	0.001833751	0.028393745
NM_022893.3	BCL11A	2	60678302	60780702	0.00183486	0.028393745
NM_145863.1	ASB3	2	53759810	54087297	0.001835388	0.028393745
NM_139021.2	MAPK15	8	144798429	144804628	0.00183688	0.028403989
NM_000182.4	HADHA	2	26413504	26467594	0.001847856	0.028560807
NM_018479.2	ECHDC1	6	127609855	127664754	0.001849356	0.028571082
NM_018107.4	RBM23	14	23369855	23388396	0.001853594	0.028623639
NM_001017969.1	KIAA2026	9	5919008	6008003	0.001855849	0.028645541
NM_014925.2	R3HDM2	12	57647547	57824788	0.001858952	0.028674147
NM_018017.2	C10orf118	10	115880621	115933979	0.001859379	0.028674147
NM_032750.1	ABHD14B	3	52002526	52017425	0.001862251	0.028705516
NM_016133.2	INSIG2	2	118846028	118868573	0.001865689	0.028745558
NM_018359.1	UFSP2	4	186320694	186347139	0.001868922	0.02878241
NM_007240.1	DUSP12	1	161719548	161727028	0.001874715	0.02885282
NM_004494.1	HDGF	1	156711899	156736717	0.00187518	0.02885282
NM_212551.3	LYSMD1	1	151132224	151138424	0.001876998	0.028867802
NM_012308.1	KDM2A	11	66886740	67025141	0.001878738	0.028881581
NM_024641.2	MANEA	6	96025419	96057333	0.001881416	0.028909774
NM_001042432.1	CLN3	16	28488600	28503623	0.001887601	0.028991791
NM_138792.2	LEO1	15	52230222	52263997	0.001888983	0.028998557
NM_032336.1	GINS4	8	41386725	41402565	0.001889736	0.028998557
NM_019112.3	ABCA7	19	1040102	1065571	0.001891673	0.029004253
XM_001717454.1	KLHL29	2	23608088	23931481	0.001891803	0.029004253

NM_001039802.1	CDC42	1	22379120	22419437	0.00189738	0.029067184
BG461628	AL139008.5	9	33638033	33638492	0.001897939	0.029067184
NM_020207.1	C9orf102	9	98637900	98776845	0.001898835	0.029067184
NM_001012751.1	ABI1	10	27035522	27149959	0.001899981	0.029067184
NM_080867.2	SOCS4	14	55493844	55516206	0.001900467	0.029067184
NM_138771.3	CCDC126	7	23636688	23684327	0.001901004	0.029067184
NM_138570.2	SLC38A10	17	79218801	79269096	0.001901877	0.029067552
NM_058246.3	DNAJB6	7	157128075	157210133	0.001904776	0.029098859
NM_000183.2	HADHB	2	26466038	26513336	0.00190727	0.029123952
NM_017815.1	HAUS4	14	23415437	23426357	0.001915641	0.029238727
NM_001039842.1	C17orf90	17	79632067	79633618	0.001922442	0.02932913
NR_003505.2	PPP4R1L	20	56806188	56884495	0.001923278	0.02932913
NM_004719.2	SRSF2IP	12	46315192	46384363	0.001924836	0.029339817
NM_015305.2	ANGEL1	14	77253588	77279283	0.00193332	0.029446174
NM_017489.1	TERF1	8	73921099	73960357	0.001933535	0.029446174
NM_032378.2	EEF1D	8	144661892	144681711	0.001937686	0.029496264
NM_015920.3	RPS27L	15	63445539	63450220	0.001940482	0.029519088
NM_016334.3	GPR89A	1	145764411	145827073	0.00194091	0.029519088
NM_177552.2	SULT1A3	16	30205743	30215649	0.001942225	0.02952596
NM_016106.2	SCFD1	14	31091521	31209645	0.001943298	0.029529159
NM_020865.1	DHX36	3	153990335	154042286	0.001947983	0.02957748
NM_152641.2	ARID2	12	46123492	46301823	0.001948207	0.02957748
NM_001098477.1	GRSF1	4	71681499	71705662	0.001951699	0.02961735
NM_017647.2	FTSJ3	17	61896795	61905031	0.001961582	0.029754136
NM_001005861.1	RYK	3	133794023	133969494	0.001965009	0.02979291
NM_002158.3	FOXN2	2	48541776	48606433	0.0019663	0.029799285
NM_152834.2	TMEM18	2	667335	677439	0.001970408	0.029848328
NM_004889.2	ATP5J2	7	99046098	99063954	0.001975451	0.029905429
NM_014343.1	CLDN15	7	100875373	100882101	0.001975926	0.029905429
NR_002555.2	RP11-347C12.1	16	30234196	30278959	0.001979974	0.029953451
NM_001077204.1	CSPP1	8	67974661	68108498	0.001987938	0.030060639
NM_024793.1	CLUAP1	16	3550963	3587669	0.001990208	0.030081681
NM_032231.4	FAM96A	15	64364758	64386217	0.001993097	0.030112037
NM_001018108.2	SERF2	15	44069285	44094787	0.001996449	0.030149374
NM_003751.3	EIF3B	7	2393721	2420380	0.002000305	0.030171294
NM_016433.3	GLTP	12	110288751	110318293	0.002000395	0.030171294
NM_020235.3	BBX	3	107241783	107530171	0.002000545	0.030171294
NM_032207.2	C19orf44	19	16607205	16632163	0.00200308	0.030194525
NM_017515.3	SLC35F2	11	107661717	107799019	0.002003851	0.030194525
NM_013406.1	DHPS	19	12786537	12792682	0.002005481	0.030205787
NM_004629.1	VCP	9	35056061	35073246	0.002009724	0.030256379
NM_001009182.1	SIP1	14	39583427	39606177	0.002018551	0.030375902
NM_024956.3	TMEM62	15	43425722	43477333	0.002019719	0.030380118
H03033	AL139008.5	9	33638033	33638492	0.002024894	0.030444577
NM_001040633.1	PRKAG2	7	151253203	151574316	0.002028213	0.030467751
NM_024640.3	YRDC	1	38268616	38273857	0.002028302	0.030467751
XM_001129563.1	PLA2G2C	1	20487746	20503917	0.002029106	0.030467751
NM_199295.1	APITD1	1	10490159	10512210	0.002034366	0.030515956
NM_019071.2	ING3	7	120590803	120617270	0.002034827	0.030515956
NM_024958.1	NRSN2	20	327370	335512	0.002034992	0.030515956
NM_022371.3	TOR3A	1	179050512	179067158	0.002037555	0.030541006
BX538009	HIPK2	7	139246316	139477577	0.002042313	0.030598924
NM_015994.2	ATP6V1D	14	67804806	67826966	0.002046012	0.030628882

NM_015466.1	PTPN23	3	47422491	47454931	0.002046137	0.030628882
NM_024537.1	CARS2	13	111293759	111365882	0.002046997	0.030628882
NM_001001660.2	LYRM5	12	25348206	25359026	0.002056606	0.030759201
NM_001428.2	ENO1	1	8921061	8939308	0.002060631	0.030805933
NM_004956.3	ETV1	7	13930853	14031050	0.002064661	0.030852712
NM_001081559.1	CPSF4	7	99036545	99054994	0.00206753	0.030882093
NM_016456.2	TMEM9	1	201103900	201140702	0.002069832	0.030886785
NM_198513.1	PHF20L1	8	133787618	133861052	0.002070045	0.030886785
NM_144999.2	LRRC45	17	79981280	79989024	0.002070552	0.030886785
NM_014667.1	VGLL4	3	11597544	11762220	0.002071724	0.030890811
NM_152396.2	METTL6	3	15422782	15469047	0.002076981	0.030937759
NM_001077203.1	SENP7	3	101043049	101232085	0.002077582	0.030937759
NM_172341.1	PSENEN	19	36236494	36237903	0.002077585	0.030937759
NM_177536.1	SULT1A3	16	30205743	30215649	0.002079943	0.030959395
NM_000302.2	PLOD1	1	11994262	12035599	0.002085572	0.031027471
NM_016108.2	AIG1	6	143381633	143661441	0.002086738	0.031027471
NM_198480.2	ZNF615	19	52467594	52627345	0.002087237	0.031027471
NM_001350.3	DAXX	6	33286335	33297046	0.002092847	0.031076824
NM_000553.3	WRN	8	30891317	31031285	0.002092873	0.031076824
NM_013446.2	MKRN1	7	140152840	140179369	0.002093281	0.031076824
NM_001097616.1	GPR89C	1	145883868	145924373	0.002100626	0.031166339
NM_020210.2	SEMA4B	15	90728152	90772892	0.002101132	0.031166339
NM_032860.3	LTV1	6	144164481	144184949	0.002107258	0.031243662
NM_023008.3	KRI1	19	10663761	10676713	0.002115119	0.03132291
NM_013316.2	CNOT4	7	135046549	135194851	0.002115236	0.03132291
NM_002752.3	MAPK9	5	179660143	179719099	0.002115596	0.03132291
NM_013293.3	TRA2A	7	23544399	23571660	0.002116264	0.03132291
NM_199121.1	VWA1	1	1370241	1378262	0.002117832	0.031328246
NM_015914.5	TXNDC11	16	11772943	11836704	0.002119018	0.031328246
NM_004034.1	ANXA7	10	75135203	75173834	0.002119371	0.031328246
NM_020967.2	NCOA5	20	44689624	44718591	0.002122146	0.031355711
NM_181676.1	PPP2R2B	5	145967936	146464347	0.00212479	0.031381234
NM_017983.4	WIPI1	17	66417423	66453653	0.002126434	0.031391558
NM_019093.2	UGT1A3	2	234637754	234681945	0.002127324	0.031391558
NM_001009944.1	PKD1	16	2138711	2185899	0.002129476	0.031409778
NM_017838.3	NHP2	5	177576461	177580968	0.00213167	0.031428591
NM_031431.2	COG3	13	46039060	46110765	0.002133158	0.031436981
NM_001098495.1	ZNF419	19	57999079	58006048	0.002143332	0.03155985
NM_004046.4	ATP5A1	18	43664110	43684300	0.00214334	0.03155985
NM_032905.3	RBM17	10	6130949	6159420	0.002147123	0.031601959
NM_001865.2	COX7A2	6	75947391	75966492	0.002150427	0.03162916
NM_005998.3	CCT3	1	156278752	156337664	0.002151697	0.03162916
NM_001002002.1	GMPR2	14	24701648	24708445	0.002152847	0.03162916
NM_021915.2	ZNF69	19	11998599	12025144	0.002152898	0.03162916
NM_138458.2	WDR92	2	68350068	68384692	0.002153593	0.03162916
NM_005861.2	STUB1	16	730276	732753	0.002159446	0.031701519
NM_181472.1	CMTM7	3	32433163	32524559	0.002161885	0.031723711
NM_003162.2	STRN	2	37070783	37193615	0.002162922	0.031725323
NM_020204.2	LHX9	1	197881618	197904608	0.002164799	0.031739262
NM_004255.2	COX5A	15	75212132	75230509	0.002169625	0.031785067
AA668123	AL139008.5	9	33638033	33638492	0.002170528	0.031785067
NM_016063.2	HDDC2	6	125596496	125623282	0.00217071	0.031785067
NM_024294.2	C6orf106	6	34555065	34664636	0.002172381	0.03179593

XM_936739.1	AC105935.1	3	49977592	49980293	0.00217468	0.031815963
NM_003040.2	SLC4A2	7	150754297	150773614	0.002185612	0.031957663
NM_002896.2	RP11-658F2.1	11	66384097	66413944	0.002186233	0.031957663
NM_006773.3	DDX18	2	118572226	118589955	0.002191286	0.03201785
NM_024800.3	NEK11	3	130745694	131069309	0.002196472	0.032079924
NM_018648.3	NOP10	15	34633914	34635378	0.002198575	0.032096938
NM_005188.2	CBL	11	119076752	119177651	0.002201105	0.03212017
NM_014341.1	MTCH1	6	36935917	36954074	0.00220373	0.032129512
NM_003945.3	ATP6V0E1	5	172410760	172462448	0.002204538	0.032129512
NM_207040.1	TCF12	15	57210823	57582051	0.002204562	0.032129512
NM_007187.3	WBP4	13	41635410	41658137	0.00221009	0.032196366
NM_006348.2	COG5	7	106842189	107204959	0.002212749	0.032221395
NM_182830.2	MDGA2	14	47308826	48143953	0.002215811	0.032252259
NM_023019.1	DCTN1	2	74588281	74619214	0.002220766	0.032310639
NM_183236.1	RAB27A	15	55495164	55582001	0.002222332	0.032319683
NM_021210.3	TRAPPC1	17	7833663	7835266	0.00222618	0.032361888
NM_001388.3	DRG2	17	17991239	18011285	0.002230239	0.032407129
NM_139048.2	HLTF	3	148747914	148804341	0.002231349	0.032409488
NM_001001.3	RPL36AL	14	50085407	50087361	0.002235323	0.032453445
NM_032169.4	ACAD11	3	132276982	132379567	0.00223714	0.032465737
NM_178820.3	FBXO27	19	39514693	39523236	0.002238068	0.032465737
NM_016546.1	C1RL	12	7247153	7261793	0.00223935	0.032470576
NM_014969.4	WDR47	1	109512836	109584850	0.002241305	0.032476104
NM_152654.2	DAND5	19	13080432	13085567	0.002241629	0.032476104
NM_006624.3	ZMYND11	10	180405	300577	0.002251856	0.032610458
NM_001006639.1	TCEAL1	23	102883632	102885881	0.002259326	0.0327048
NM_014388.5	C1orf107	1	210001352	210030910	0.002264651	0.032768016
NM_001003931.1	PARP3	3	51976361	51982883	0.002266883	0.032774357
NM_017457.4	CYTH2	19	48972289	48985571	0.002267005	0.032774357
NM_013399.2	C16orf5	16	4560676	4588816	0.002271965	0.032832194
NM_017547.2	FOXRED1	11	126138950	126148026	0.002280322	0.03293906
NM_001105573.1	FBXO45	3	196295482	196315930	0.002283331	0.032968595
NM_005837.2	POP7	7	100303676	100305118	0.00229126	0.033055192
NM_001293.1	CLNS1A	11	77225981	77348850	0.002294807	0.033067652
NM_004830.2	MED23	6	131895106	131949369	0.002294829	0.033067652
NM_006118.3	HAX1	1	154244987	154248351	0.002295685	0.033067652
NM_016142.1	HSD17B12	11	43577986	43878167	0.002295989	0.033067652
NM_019059.2	TOMM7	7	22850011	22862470	0.002300063	0.033099928
NM_175852.3	TXLNA	1	32645287	32663886	0.002300164	0.033099928
NM_177422.1	EIF2C3	1	36396319	36522063	0.002303215	0.033129894
NM_198597.1	SEC24C	10	75504132	75531919	0.002309175	0.033180117
NM_022061.2	MRPL17	11	6702013	6704632	0.002309296	0.033180117
NM_020062.3	SLC2A4RG	20	62369623	62374858	0.002309753	0.033180117
NM_058191.3	GCFC1	21	34106210	34144169	0.002310585	0.033180117
NM_014254.1	TMEM5	12	64173664	64203300	0.002319338	0.033279047
XM_937518.2	OOEP	6	74078278	74104856	0.002319419	0.033279047
XR_015281.1	AGAP10	10	47191778	47243474	0.00232168	0.033297524
NM_000938.1	POLR2B	4	57843888	57897324	0.002323644	0.033311727
NM_023935.1	DDRGK1	20	3170996	3185331	0.002327108	0.033347419
NM_207113.1	SLC37A3	7	139993493	140104233	0.00232954	0.033368293
NM_052969.1	RPL39L	3	186838736	186898696	0.002332951	0.033403172
NM_006845.2	KIF2C	1	45205490	45233439	0.002335345	0.033417965
NM_001077692.1	FDXACB1	11	111656529	111750149	0.002335937	0.033417965

NM_198446.1	C1orf122	1	38272651	38275126	0.002340712	0.033471792
NM_017934.4	PHIP	6	79650263	79788011	0.002341656	0.033471792
NM_016567.2	BCCIP	10	127512115	127542264	0.002343828	0.033488849
NM_021141.2	XRCC5	2	216972187	217071026	0.002346469	0.033504191
NM_013263.2	BRD7	16	50352941	50402845	0.00234686	0.033504191
NM_017632.2	CDKN2AIP	4	184365744	184369351	0.002355604	0.033614996
NM_015037.2	KIAA0913	10	75545383	75561551	0.002357527	0.033617792
NM_052927.1	PWWP2A	5	159488808	159546430	0.002358616	0.033617792
NM_014761.2	KIAA0174	16	71929304	71962913	0.002358747	0.033617792
NM_001151.2	SLC25A4	4	186064395	186068434	0.002362388	0.033649722
NM_001079673.1	FNDC3A	13	49550048	49783888	0.002362954	0.033649722
NM_004747.3	DLG5	10	79550549	79686378	0.002366468	0.033685745
NM_153824.1	PYCR1	17	79890269	79895172	0.002369437	0.033713992
NM_001042442.1	CAST	5	95865525	96115299	0.002373016	0.033731739
NM_018248.1	NEIL3	4	178230990	178284097	0.002374606	0.033731739
NM_016586.1	MBIP	14	36767764	36789882	0.00237484	0.033731739
NM_014815.3	MED24	17	38175350	38210660	0.002375418	0.033731739
AL137257	UHMK1	1	162467633	162499400	0.002375613	0.033731739
NM_015374.1	SUN2	22	39130730	39190148	0.002377088	0.03373868
NM_020317.3	C1orf63	1	25568740	25594327	0.0023794	0.033744363
NM_032795.1	RPUSD4	11	126071993	126081587	0.002379461	0.033744363
NM_014988.1	LIMCH1	4	41361624	41702061	0.002384173	0.03379718
NM_001009182.1	SIP1	14	39583427	39606177	0.002387218	0.033826341
NM_016324.2	ZNF274	19	58694396	58724927	0.002389056	0.033838363
NM_182692.1	SRPK2	7	104751151	105039755	0.002390459	0.033844233
NM_003653.2	COPS3	17	17150134	17184607	0.002393076	0.033867275
NM_001002000.1	GMPR2	14	24701648	24708445	0.002396207	0.03387345
NM_006949.1	STXBP2	19	7701991	7712758	0.002397254	0.03387345
NM_014967.3	FAN1	15	31196107	31235308	0.00239741	0.03387345
NM_032864.3	PRPF38A	1	52870236	52883992	0.002397472	0.03387345
NM_152132.1	PSMA3	14	58711549	58738725	0.002399834	0.033892823
NM_016050.2	MRPL11	11	66202546	66234209	0.002406496	0.033972894
NM_005552.4	C14orf153	14	104029299	104167887	0.002410635	0.034009442
NM_019109.3	ALG1	16	5121820	5135395	0.002411073	0.034009442
NM_004260.2	RECQL4	8	145736671	145743229	0.002413449	0.03402893
NM_020826.1	SYT13	11	45261852	45307870	0.00241672	0.034061015
NM_024860.1	SETD6	16	58549383	58554431	0.00241783	0.034062631
NM_013238.2	DNAJC15	13	43597339	43683045	0.002424698	0.034145339
NM_017810.2	ZNF434	16	3432085	3451030	0.002435743	0.03428676
NM_006409.2	ARPC1A	7	98923521	98992424	0.002440724	0.034342755
NM_002208.4	ITGAE	17	3617923	3704544	0.002445364	0.03438128
NM_006479.3	RAD51AP1	12	4647950	4669213	0.002445471	0.03438128
NM_006145.1	DNAJB1	19	14625582	14629201	0.002449551	0.034424488
NM_001008489.2	PHOSPHO2	2	170550975	170558218	0.002452182	0.034447321
NM_013339.2	ALG6	1	63833261	63904233	0.002455255	0.034476327
NM_000122.1	ERCC3	2	128014866	128051752	0.00245673	0.034482889
NM_005854.1	RAMP2	17	40913205	40915059	0.00245997	0.034508133
NM_020761.2	RPTOR	17	78518625	78940173	0.002460684	0.034508133
NM_004470.2	FKBP2	11	64008413	64011606	0.002461553	0.034508133
NM_002047.2	GARS	7	30634297	30673649	0.002466067	0.034543125
NM_170721.1	MSI2	17	55333931	55757299	0.002466068	0.034543125
NM_145897.2	PFDN5	12	53689235	53693230	0.002468018	0.034556287
NM_032788.1	ZNF514	2	95813075	95831158	0.002479079	0.034696969

NM_180977.1	PPP2R5D	6	42952237	42980080	0.002481277	0.034713527
NM_024079.4	ALG8	11	77811982	77850706	0.002482813	0.03472083
NM_003899.2	ARHGEF7	13	111766906	111958084	0.002485508	0.034742463
NM_024653.3	PRKRIP1	7	102004319	102067123	0.002486391	0.034742463
NM_031288.2	INO80B	2	74682150	74688011	0.002490191	0.034781358
NM_001002018.1	HCFC1R1	16	3072626	3074287	0.002494832	0.034821591
NM_152834.2	TMEM18	2	667335	677439	0.002495106	0.034821591
NM_198038.1	NUDT9	4	88343734	88380606	0.002502766	0.034914252
NM_007079.2	PTP4A3	8	142402093	142441620	0.002504054	0.034917988
NM_005570.2	LMAN1	18	56995055	57026503	0.002506183	0.034925953
NM_001012398.1	AKTIP	16	53524952	53537170	0.002508764	0.03494091
NM_207327.4	C22orf40	22	46639908	46646576	0.002509865	0.03494091
NM_018128.4	TSR1	17	2225992	2240678	0.002510804	0.03494091
NM_001665.3	RHOG	11	3848208	3862213	0.002511944	0.034942577
NM_199129.1	TMEM189	20	48697661	48770271	0.002518662	0.035012122
NM_001013251.1	SLC3A2	11	62623518	62656352	0.002520079	0.035012122
NM_014183.2	DYNLRB1	20	33104214	33128762	0.002520492	0.035012122
NM_199482.1	MOBKL3	2	198380295	198418423	0.002521037	0.035012122
NM_014453.2	CHMP2A	19	59062934	59066486	0.002522654	0.035020373
NM_000190.3	HMBS	11	118955587	118964259	0.002525352	0.035043615
NM_004602.2	STAU1	20	47729878	47804904	0.002528698	0.035075812
NM_003003.2	SEC14L1	17	75137005	75213183	0.002534426	0.035141031
NM_022771.3	TBC1D15	12	72233487	72318097	0.002543892	0.035257998
NM_012096.2	APPL1	3	57261765	57307496	0.002545636	0.035267887
NM_001039847.1	GPX4	19	1103966	1106778	0.002546681	0.035268077
XM_930505.2	DAP3	1	155657751	155708803	0.002556229	0.035385984
AW630386	AL139008.5	9	33638033	33638492	0.002559187	0.035412615
NM_013291.2	CPSF1	8	145618444	145634753	0.002565187	0.035481282
NM_015043.3	TBC1D9B	5	179289066	179334859	0.002567509	0.035493416
NM_005841.1	SPRY1	4	124317950	124324910	0.002568284	0.035493416
NM_003133.1	SRP9	1	225965531	225978168	0.002569243	0.035493416
NM_138358.2	C19orf52	19	11039424	11040914	0.002571279	0.035493416
NM_001519.2	BRF1	14	105675623	105781914	0.002572264	0.035493416
NR_003501.1	EARS2	16	23533335	23568696	0.002572287	0.035493416
NM_000271.3	NPC1	18	21111401	21166470	0.002576908	0.035530109
NM_016587.2	CBX3	7	26240782	26252976	0.002577023	0.035530109
NM_001013439.1	FXR1	3	180585929	180694950	0.002581397	0.035576084
NM_007006.2	NUDT21	16	56463045	56485287	0.002583501	0.035590743
NM_005134.2	PPP4R1	18	9546789	9614567	0.002585373	0.035602198
XM_001131964.1	MZT2A	2	132222473	132250316	0.002594648	0.035707423
NM_004720.4	LPAR2	19	19734468	19739039	0.002595101	0.035707423
NM_002911.3	UPF1	19	18942744	18979038	0.002608535	0.035877842
NM_022912.1	REEP1	2	86441116	86565206	0.002613054	0.03592556
NM_153331.2	KCTD6	3	58477841	58488087	0.002618527	0.035986342
NM_078471.3	MYO18A	17	27400537	27507430	0.00262104	0.036006416
NM_005001.2	NDUFA7	19	8376234	8386280	0.002624507	0.036039581
NM_144999.2	LRRC45	17	79981280	79989024	0.002626504	0.036052535
NM_004804.2	CIAO1	2	96931870	96939087	0.002627929	0.036057624
NM_020850.1	RANBP10	16	67757005	67840555	0.002630496	0.036078388
NM_007234.3	DCTN3	9	34613548	34620478	0.002635571	0.036133509
NM_002162.2	ICAM3	19	10444454	10450345	0.002639732	0.036176068
NM_014904.1	RAB11FIP2	10	119764427	119806114	0.002647982	0.036274602
NM_144976.2	ZNF564	19	12636185	12662327	0.002659287	0.036414881

NM_001005361.1	DNM2	19	10824143	10942579	0.002662358	0.03644235
NM_198537.2	NDUFA13	19	19626540	19646885	0.002663684	0.036444315
NM_174926.1	TMEM136	11	120195838	120204391	0.002664849	0.036444315
NM_022130.3	GOLPH3	5	32124810	32174456	0.002665696	0.036444315
NM_014187.3	TMEM208	16	67261016	67263182	0.002668696	0.036470754
NM_005482.2	PIGK	1	77554675	77685115	0.002670518	0.036481086
NM_033426.2	KIAA1737	14	77564578	77583629	0.002674459	0.036506376
NM_014607.3	UBXN4	2	136499189	136542625	0.002675162	0.036506376
NM_018044.2	NSUN5	7	72716514	72722864	0.00267557	0.036506376
NM_018394.1	ABHD10	3	111697857	111712210	0.002677219	0.036514317
NM_002926.3	RGS12	4	3294755	3441640	0.002681824	0.036562556
NM_032856.2	WDR73	15	85186014	85197521	0.002687329	0.036601962
NM_024031.2	PRR14	16	30662223	30667761	0.002687607	0.036601962
XM_001129035.1	LEUTX	19	40267231	40276775	0.002688118	0.036601962
NM_015091.2	FAM179B	14	45431411	45543634	0.002688993	0.036601962
NM_080476.4	PIGU	20	33148346	33264910	0.002693899	0.036654162
NM_052950.2	WDFY2	13	52158644	52334135	0.002697451	0.036676851
NM_001097616.1	GPR89A	1	145764411	145827073	0.00269771	0.036676851
NM_033167.2	B3GALNT1	3	160801671	160823172	0.002704866	0.036759533
NM_203464.1	AK4	1	65613232	65697819	0.002706372	0.036765401
NM_019103.2	ZMAT5	22	30126945	30163000	0.002709697	0.036784112
NM_005993.4	TBCD	17	80709940	80901056	0.002709899	0.036784112
NM_016640.3	MRPS30	5	44809027	44820530	0.002721721	0.036929931
NM_018216.1	PANK4	1	2439972	2458050	0.002723769	0.036943073
NM_001003686.1	DTX2	7	76090972	76151394	0.002726225	0.036961734
NM_007149.1	ZNF184	6	27418522	27440897	0.002727478	0.036964083
NM_001010990.1	HERPUD1	16	56965748	56977793	0.002729031	0.036970483
NM_031431.2	COG3	13	46039060	46110765	0.002731972	0.036984836
NM_001003398.1	BICD1	12	32260182	32531141	0.002732252	0.036984836
NM_182573.1	LYPD5	19	44300080	44324808	0.002739733	0.037068949
NM_133173.2	SRA1	5	139929752	139937895	0.002740632	0.037068949
NM_148172.1	PEMT	17	17408877	17495022	0.002744812	0.037110211
AK026373	HNRNPA2B1	7	26229547	26241149	0.002745852	0.037110211
NM_019557.5	FAM54B	1	26145131	26159432	0.002749213	0.037140965
NM_003961.1	RHBDL1	16	725666	728268	0.002752511	0.037159429
NM_152653.2	UBE2E2	3	23244511	23633284	0.002752751	0.037159429
NM_024805.1	RBFA	18	77794358	77810651	0.0027572	0.037203524
NM_004548.1	NDUFB10	16	2009519	2011976	0.002758688	0.037203524
NM_181308.1	WDR20	14	102606212	102690007	0.002759279	0.037203524
NM_015295.2	SMCHD1	18	2655886	2805015	0.002760587	0.037206488
NM_018130.2	SHQ1	3	72798428	72911065	0.002762548	0.037212149
NM_001079872.1	CUL4B	23	119658464	119709649	0.002763182	0.037212149
NM_017693.2	BIVM	13	103451399	103493881	0.002765364	0.037226897
NM_004068.3	AP2M1	3	183892477	183901879	0.002767016	0.037234485
NM_178569.2	C5orf38	5	2752245	2755508	0.002769992	0.037259883
NM_001923.2	DDB1	11	61066920	61100666	0.002772205	0.037274993
NM_014654.2	SDC3	1	31342313	31381608	0.002776447	0.037317366
NM_005016.3	PCBP2	12	53845886	53874945	0.002778537	0.037330792
NM_020710.1	LRRC47	1	3696784	3713068	0.002783595	0.037384077
NM_138493.2	C6orf129	6	37450696	37467700	0.002784846	0.037386198
NM_182483.1	NSFL1C	20	1422807	1448337	0.002790503	0.037438508
NM_002810.2	PSMD4	1	151227179	151239955	0.00279093	0.037438508
NM_004724.2	ZW10	11	113603913	113644425	0.002796236	0.037494982

NM_001564.2	ING2	4	184426147	184432249	0.002799458	0.037523486
NM_000945.3	PPP3R1	2	68358370	68488362	0.002802131	0.037544603
NM_001080398.1	KIAA0368	9	114122972	114247025	0.002808352	0.037613235
NM_014577.1	BRD1	22	50166931	50221160	0.002812756	0.037654502
NM_207406.2	BEND4	4	42112955	42154895	0.002813634	0.037654502
NM_015912.3	FAM135B	8	139142266	139509065	0.002817951	0.037697524
NM_004935.2	CDK5	7	150750899	150755617	0.002828763	0.037817829
NM_021645.4	AL139082.1	13	52598827	52607701	0.002829154	0.037817829
NM_032226.2	ZCCHC7	9	37120536	37358146	0.002830311	0.037818522
NM_001048241.1	UBL5	19	9938568	9940797	0.002833751	0.037849714
NM_014864.2	FAM20B	1	178994939	179045697	0.002835917	0.037863857
NM_001077665.1	AGAP6	10	51748078	51770259	0.002841138	0.03791878
NM_005713.1	COL4A3BP	5	74664311	74807963	0.002846356	0.037973609
NM_017900.1	AURKAIP1	1	1309110	1310875	0.002851074	0.03802173
NM_014674.1	EDEM1	3	5229331	5261642	0.002852461	0.038025407
NM_001023.2	RPS20	8	56979854	56987069	0.002853647	0.038026399
NM_024612.3	DHX40	17	57642886	57685702	0.002858422	0.038075199
NM_032476.2	MRPS6	21	35445524	35515334	0.00286803	0.038188323
NM_001039712.1	DEDD	1	161090764	161102478	0.002870443	0.038205585
NM_173822.2	FAM126B	2	201843216	201936394	0.002876161	0.038266814
NM_005870.3	SAP18	13	21714653	21723221	0.002882658	0.038338343
NM_024029.3	YIPF2	19	11033444	11039357	0.002884364	0.038346127
NM_002265.4	KPNB1	17	45727275	45760998	0.002886039	0.038353497
NM_198097.2	C7orf28B	7	6836440	6866401	0.002887815	0.038356693
NM_198976.1	TH1L	20	57556296	57570188	0.002889317	0.038356693
NM_001080394.1	KIAA0146	8	48173167	48648868	0.002889674	0.038356693
NM_003172.2	SURF1	9	136218610	136223552	0.002891776	0.038356693
NM_012228.2	MSRB2	10	23384435	23410942	0.002892687	0.038356693
NM_001008215.1	C2orf64	2	99215773	99224978	0.002894017	0.038356693
NM_016641.3	GDE1	16	19513015	19533450	0.002894126	0.038356693
NM_005400.2	PRKCE	2	45878484	46415129	0.002926159	0.038766229
NM_138799.2	MBOAT2	2	8992820	9143942	0.002936858	0.038871424
NM_001031733.2	CALML4	15	68483043	68498417	0.002937347	0.038871424
NM_006447.2	USP16	21	30396950	30426809	0.002937507	0.038871424
NR_001450.2	RFPL3S	22	32755893	32767063	0.002940012	0.038889532
NM_181705.2	LYRM7	5	130506503	130541119	0.002944052	0.038927923
NM_017615.1	NSMCE4A	10	123716603	123734732	0.002950325	0.038982357
NM_003295.2	TPT1	13	45911008	45915505	0.002950447	0.038982357
NM_014051.3	TMEM14A	6	52535907	52551386	0.002960104	0.039094847
NM_004218.2	RAB11B	19	8455205	8469313	0.002966174	0.03915991
NM_020186.1	ACN9	7	96745902	96811075	0.002972961	0.039232998
NM_004870.2	MPDU1	17	7486974	7495790	0.002975584	0.039232998
NM_015995.2	KLF13	15	31619058	31670102	0.002975859	0.039232998
NM_007086.2	WDHD1	14	55405668	55493823	0.002976296	0.039232998
NM_001077352.1	RBM23	14	23369855	23388396	0.002978797	0.039237904
NM_012453.2	TBL2	7	72983990	72993121	0.002978962	0.039237904
NM_194282.1	LIN54	4	83831126	83934079	0.002982775	0.039249169
NM_145049.1	UBLCP1	5	158690089	158713044	0.002985525	0.039249169
XM_001126750.1	ABI2	2	204192942	204301606	0.002985626	0.039249169
NM_005657.1	TP53BP1	15	43699407	43802926	0.002985832	0.039249169
NM_025010.3	KLHL18	3	47324330	47388306	0.002986527	0.039249169
NM_020202.2	NIT2	3	100053545	100074449	0.002986731	0.039249169
NM_182917.2	EIF4G1	3	184032283	184053146	0.002987846	0.039249169

NM_030662.2	MAP2K2	19	4090319	4124126	0.002998781	0.039374202
NM_006425.4	SLU7	5	159828648	159848718	0.002999665	0.039374202
NM_024074.1	TMEM38A	19	16771938	16799814	0.003001484	0.039382969
NM_005829.3	AP3S2	15	90373831	90437574	0.003006032	0.039427527
NM_016121.3	KCTD3	1	215740735	215795147	0.003007898	0.039436881
NM_001077686.1	AGAP8	10	51224677	51252947	0.00301107	0.039463348
NM_017762.2	FAN1	15	31196107	31235308	0.003015675	0.039508575
NM_152892.1	LRWD1	7	102105376	102113615	0.003023901	0.03960118
NM_139286.3	CDC26	9	116018115	116037869	0.003026131	0.039615223
NM_005827.1	SLC35B1	17	47778305	47786376	0.003027819	0.039622169
NM_001008395.2	C7orf59	7	99746530	99753567	0.003038044	0.039740773
NM_013354.5	CNOT7	8	17086737	17104387	0.003044462	0.039808446
NM_004393.2	DAG1	3	49506146	49573044	0.003045544	0.039808446
NM_006974.2	ZNF33A	10	38299578	38356282	0.003048339	0.039829767
NM_002227.2	JAK1	1	65298912	65432187	0.003051985	0.03986218
NM_001035005.2	C18orf32	18	47008030	47013601	0.003061136	0.039954858
NM_014487.3	ZNF330	4	142142041	142155851	0.003061416	0.039954858
NM_005517.2	HMGN2	1	26798941	26802463	0.003065947	0.03999875
NM_007033.3	RER1	1	2323267	2335190	0.003068035	0.040010727
NM_032593.2	HINT2	9	35812957	35815351	0.003071341	0.040038581
NM_080475.1	SERPINB11	18	61314813	61391127	0.003073198	0.04004587
NM_080430.2	RP3-412A9.11	22	31500758	31516055	0.003074391	0.04004587
XR_018104.1	ATP5C1	10	7830092	7849778	0.00307541	0.04004587
NM_006020.2	ALKBH1	14	78138749	78174356	0.003079038	0.040077851
NM_023073.2	C5orf42	5	37106330	37247894	0.003082827	0.040101124
NM_018087.3	TMEM48	1	54231133	54304533	0.003083169	0.040101124
NM_025184.3	EFHC2	23	44007128	44202923	0.003088205	0.040151359
NM_001038618.1	NARF	17	80416060	80446141	0.003093857	0.040206193
NM_001013251.1	SLC3A2	11	62623518	62656352	0.003094772	0.040206193
NM_001359.1	DECR1	8	91013633	91064320	0.003098036	0.04020769
XM_495854.3	DUX4L4	4	191002090	191003541	0.003098182	0.04020769
NM_152902.3	TIPRL	1	168148171	168171346	0.003098928	0.04020769
NM_152293.2	TADA2B	4	7043626	7058368	0.003099588	0.04020769
NM_017822.3	C12orf41	12	49046997	49076035	0.003107645	0.040296937
NM_006572.3	GNA13	17	63005407	63052920	0.003110281	0.040315841
NM_178123.3	SESTD1	2	179966419	180129517	0.003111741	0.040319493
NM_001128.5	AP1G1	16	71762903	71842979	0.003115772	0.040348477
NM_144766.1	RGS13	1	192605275	192629390	0.003116336	0.040348477
NM_014366.4	GNL3	3	52715172	52728508	0.003119897	0.040379307
NM_032673.2	PCGF1	2	74732170	74735707	0.0031211	0.040379604
NM_033630.1	SCAND1	20	34541539	34547394	0.003122428	0.04038151
NM_017454.2	STAU1	20	47729878	47804904	0.003125491	0.040405859
NM_001042472.1	ABHD12	20	25275379	25371619	0.003127846	0.040419007
NM_144949.2	SOCS5	2	46926091	46990268	0.003128871	0.040419007
NM_003086.2	SNAPC4	9	139270029	139293249	0.003130471	0.040424428
NM_004487.3	GOLGB1	3	121382046	121468602	0.003142424	0.040563466
NM_005993.4	TBCD	17	80709940	80901056	0.003144215	0.040571283
NM_015203.2	RPRD2	1	150335567	150449042	0.003147773	0.040601886
NM_198708.1	HSD11B1L	19	5681035	5688532	0.00315484	0.040677698
NM_020699.1	GATAD2B	1	153777201	153895451	0.003156819	0.040687886
NM_006285.2	TESK1	9	35605367	35610038	0.003159521	0.040706814
NM_182662.1	AADAT	4	170981373	171012850	0.003160666	0.040706814
NM_013943.1	CLIC4	1	25071848	25170815	0.0031623	0.040711632

NM_181877.3	ZSCAN2	15	85144249	85166947	0.00316342	0.040711632
NM_001040181.1	CLDND1	3	98216756	98241910	0.003171751	0.040794662
NM_004775.2	B4GALT6	18	29202209	29264686	0.003172256	0.040794662
NM_183237.1	RNF7	3	141457046	141466402	0.003176247	0.040830637
NM_001025248.1	DUT	15	48623621	48635570	0.00317881	0.040845322
NM_203290.1	POLR1C	6	43477440	43497323	0.003179776	0.040845322
NM_001004318.1	AC011443.1	19	39574945	39601478	0.003182347	0.040863001
NM_030980.1	ISG20L2	1	156691683	156698591	0.003184087	0.040870019
NM_001018104.1	FAHD1	16	1876968	1890208	0.003186652	0.040876971
NM_175902.3	C17orf101	17	80350100	80376462	0.003187018	0.040876971
NM_181492.1	TCF20	22	42556019	42611448	0.003190224	0.040902757
NM_018132.3	CENPQ	6	49431096	49460820	0.003194295	0.040939621
NM_001127218.1	POLD2	7	44154286	44163957	0.003195552	0.040940399
NM_138473.2	SP1	12	53773979	53810221	0.003205912	0.04105775
NM_198489.1	CCDC84	11	118868852	118886501	0.003207858	0.041067298
NM_004489.4	GPS2	17	7215978	7222394	0.003212377	0.041109772
NM_024751.1	GSTCD	4	106629935	106768885	0.00321492	0.041118196
NM_001080502.1	RP11-551L14.1	12	31264591	31359088	0.003215438	0.041118196
NM_003185.3	TAF4	20	60528525	60640866	0.003219079	0.041137074
NM_014312.3	VSIG2	11	124617368	124622134	0.003219319	0.041137074
NM_003575.2	ZNF282	7	148892554	148923339	0.003224871	0.041177266
NM_005836.2	HRSP12	8	99114572	99129469	0.003226349	0.041180772
NM_018208.1	ETNK2	1	204100190	204121307	0.003229963	0.041211536
NM_006111.1	ACAA2	18	47309869	47340273	0.003236077	0.041274148
NM_001101800.1	FAM13B	5	137273649	137387650	0.003240889	0.041308217
NM_014984.2	AZI1	17	79163393	79196799	0.003241162	0.041308217
NM_007249.4	KLF12	13	74260150	74708394	0.003245256	0.041344992
NM_020424.2	LYRM1	16	20911557	20936328	0.003250196	0.041392516
NM_030593.1	SIRT2	19	39369197	39390502	0.003252835	0.041410721
NM_001002880.1	CBY1	22	39052641	39069859	0.003258079	0.04146205
NM_003195.4	TCEA2	20	62681189	62703700	0.003259569	0.04146559
NM_001688.4	ATP5F1	1	111991486	112005395	0.003263422	0.041490891
NM_012328.1	DNAJB9	7	108210012	108215294	0.003264378	0.041490891
NM_032352.3	BRMS1L	14	36295597	36341168	0.00326525	0.041490891
NM_005611.2	RBL2	16	53468361	53525560	0.003266407	0.041490891
NM_005896.2	IDH1	2	209100951	209130798	0.003270442	0.041507495
NM_032869.2	NUDCD1	8	110253148	110346614	0.003271304	0.041507495
NM_023929.2	ZBTB10	8	81397854	81438500	0.003271353	0.041507495
NM_018091.4	ELP3	8	27947190	28048673	0.003279115	0.041580552
NM_005587.2	MEF2A	15	100106133	100256627	0.003279541	0.041580552
XM_001128859.1	THAP4	2	242523820	242576864	0.003282151	0.041598228
NM_006519.1	DYNLT1	6	159057506	159065771	0.003285145	0.04162076
NM_001002010.1	NT5C3	7	33053742	33102409	0.003287213	0.041631548
NM_004720.5	LPAR2	19	19734468	19739039	0.003292339	0.041681031
NM_080748.1	ROMO1	20	34287194	34288906	0.003296094	0.041707623
NM_022841.5	RFX7	15	56379666	56535483	0.003297382	0.041707623
NM_130844.1	WWOX	16	78133551	79246031	0.003298095	0.041707623
NM_153282.1	NAT6	3	50333833	50336852	0.003299404	0.041708759
NM_001029991.1	METT11D1	14	21457946	21465189	0.003306844	0.041787372
NM_005776.2	CNIH	14	54893646	54908149	0.003311684	0.041831184
NM_015523.2	REXO2	11	114310108	114320998	0.003312756	0.041831184
NM_024102.2	WDR77	1	111982512	111991998	0.003314243	0.041834519
NM_153688.2	ZFP1	16	75182421	75206129	0.003318602	0.041860512

NM_006392.2	NOP56	20	2632791	2639039	0.003318748	0.041860512
NM_005663.2	WHSC2	4	1984441	2043630	0.003322138	0.041866928
XM_001126650.1	ZNF136	19	12273879	12300064	0.003322505	0.041866928
NM_198287.1	ING4	12	6759446	6772314	0.003323911	0.041866928
NM_138701.1	C7orf11	7	40172342	40174258	0.003324151	0.041866928
NM_001136262.1	ATXN7L3B	12	74931607	74935232	0.003331274	0.041909796
NM_001013406.1	KRIT1	7	91828283	91875480	0.003332363	0.041909796
XM_001131589.1	RNPS1	16	2303117	2318379	0.003332453	0.041909796
NM_138619.1	GGA3	17	73232695	73257677	0.003334605	0.041921446
NM_001080543.1	C19orf29	19	3610626	3626813	0.00333927	0.041964675
NM_032378.2	EEF1D	8	144661892	144681711	0.003341147	0.041972859
NM_001427.3	EN2	7	155250824	155257526	0.003346294	0.042022091
NM_017762.2	MTMR10	15	31231144	31283807	0.003358557	0.042150468
XM_932138.1	SLC12A6	15	34522197	34630265	0.003360544	0.042150468
NM_024106.1	ZNF426	19	9638683	9649303	0.003361403	0.042150468
NM_152636.1	METT5D1	11	28129795	28355054	0.003361796	0.042150468
NM_018070.3	SSBP3	1	54692190	54879152	0.003362676	0.042150468
NM_153613.2	LPCAT4	15	34651089	34659395	0.00336988	0.042225299
NM_015974.2	CRYL1	13	20977806	21099996	0.003374395	0.042266395
NM_019896.2	POLE4	2	75185619	75197255	0.00337714	0.042285296
XM_937211.1	RGN	23	46937775	46952712	0.003379845	0.042289181
NM_003589.2	CUL4A	13	113863086	113919399	0.003379921	0.042289181
NM_001039847.1	GPX4	19	1103966	1106778	0.003382608	0.042307332
NM_013337.2	TIMM22	17	900357	905388	0.003387656	0.042354984
NM_015929.2	LIPT1	2	99771418	99779620	0.003389015	0.042356497
NM_006785.2	MALT1	18	56338618	56421130	0.003395419	0.042395895
NM_178191.1	ATPIF1	1	28562620	28573417	0.003395445	0.042395895
NM_001040157.1	KIAA1712	4	175204828	175254531	0.003397456	0.042395895
NM_020853.1	KIAA1467	12	13197218	13236381	0.003398129	0.042395895
NM_001003656.1	PPP2R2D	10	133747955	133773331	0.003398362	0.042395895
NM_016631.3	GCFC1	21	34106210	34144169	0.003401768	0.042410994
NM_005436.2	CCDC6	10	61548521	61666818	0.003403227	0.042410994
NM_004373.2	COX6A1	12	120875904	120878532	0.00340329	0.042410994
NM_006085.4	BPNT1	1	220230824	220263191	0.003404628	0.042412221
NM_014977.2	ACIN1	14	23527776	23564823	0.003408872	0.04244964
NM_005016.5	PCBP2	12	53845886	53874945	0.00341782	0.042545579
NM_014138.3	FAM156B	23	52920336	52937587	0.003422373	0.04258677
NM_032731.3	TXNDC17	17	6544078	6547920	0.003434694	0.042711549
NM_001130404.1	PRR20C	13	57715052	57731216	0.003434897	0.042711549
NM_032444.2	SLX4	16	3631182	3661599	0.003438207	0.04273718
AK096179	C7orf40	7	45023488	45023784	0.003442768	0.042770214
NM_006357.2	UBE2E3	2	181831975	181941312	0.003443709	0.042770214
NM_020342.1	SLC39A10	2	196440701	196602426	0.003445812	0.042770214
NM_000179.1	MSH6	2	48010221	48034092	0.003445864	0.042770214
NM_033083.6	EAF1	3	15468862	15484120	0.003447323	0.042772806
NM_016567.2	BCCIP	10	127512115	127542264	0.003448812	0.042775767
NM_052963.1	TOP1MT	8	144386554	144442149	0.003450976	0.042787106
NM_001749.2	CAPNS1	19	36630918	36641254	0.003453441	0.042802156
NM_152707.2	SLC25A16	10	70242521	70287231	0.003458916	0.042848097
NM_017864.2	INTS8	8	95825539	95893974	0.003459652	0.042848097
NM_030755.4	TMX1	14	51706886	51724370	0.003465455	0.042904435
NM_144589.2	COMTD1	10	76993727	76995788	0.003468209	0.042923009
NM_001003802.1	SMARCD3	7	150935850	150974982	0.003475535	0.042978332

NM_139246.4	TSTD2	9	100362362	100395962	0.003475814	0.042978332
NM_198841.2	FAM120AOS	9	96208776	96215874	0.003476447	0.042978332
NM_130809.2	PRRC1	5	126853301	126890782	0.003484548	0.043062923
NM_006141.2	DYNC1LI2	16	66754796	66785701	0.003487176	0.04307984
NM_024887.2	DHDDS	1	26758773	26797793	0.003489222	0.043089557
NM_023937.2	MRPL34	19	17416477	17417652	0.003496127	0.043159253
NM_033100.1	CDHR1	10	85954410	85979377	0.003498658	0.043166965
NM_006904.6	AC103686.1	8	48685669	48872743	0.003499274	0.043166965
NM_004394.1	DAP	5	10679342	10761384	0.003505313	0.043225872
NM_001013843.1	SLTM	15	59171244	59225886	0.003522142	0.043399995
NM_020225.1	STOX2	4	184774584	184938894	0.003523681	0.043399995
NM_001040056.1	MAPK3	16	30125426	30134827	0.003524	0.043399995
NM_002958.3	RYK	3	133794023	133969494	0.003525087	0.043399995
NM_018991.2	STAG3L3	7	72440227	72476445	0.003525774	0.043399995
NM_018181.4	ZNF532	18	56529832	56653712	0.003538501	0.043540999
NM_000263.3	NAGLU	17	40687951	40696464	0.003555171	0.043728606
NM_006286.1	TFDP2	3	141669989	141868386	0.003556304	0.043728606
NM_005675.2	DGCR6	22	18893541	18901751	0.003560881	0.043769158
NM_032175.2	UTP15	5	72861268	72877794	0.003568512	0.043843184
NM_004891.2	MRPL33	2	27994584	28561583	0.003569466	0.043843184
NM_002340.3	LSS	21	47608360	47648738	0.00357186	0.043856851
NM_138793.2	CANT1	17	76987799	77005838	0.00357564	0.04387284
NM_004740.3	TIAF1	17	27400870	27401217	0.003576202	0.04387284
NM_001068.2	TOP2B	3	25639475	25706398	0.003577008	0.04387284
NM_153260.1	LRRC57	15	42834720	42841002	0.003587953	0.043987373
NM_002746.1	GDPD3	16	30116131	30125177	0.003588917	0.043987373
NM_052911.2	ESCO1	18	19109264	19180845	0.003592264	0.044012631
NM_032630.2	CINP	14	102814623	102829253	0.003594813	0.044027043
NM_001008495.2	TMEM64	8	91634223	91803860	0.003596014	0.044027043
NM_015226.1	CLEC16A	16	11038345	11276046	0.003607741	0.044140535
NM_020696.2	KIAA1143	3	44779153	44803154	0.003607863	0.044140535
NM_013436.3	NCKAP1	2	183789605	183903586	0.003609567	0.044143329
NM_020944.2	GBA2	9	35736863	35749225	0.003610671	0.044143329
NM_014034.1	ASF1A	6	119215384	119230332	0.003614396	0.044173084
NM_033015.2	FASTK	7	150773679	150777953	0.003617211	0.044191703
NM_001099650.1	GXYLT1	12	42475650	42538673	0.003620393	0.044214793
NM_001040443.1	PHF11	13	50069746	50103123	0.003625116	0.044240901
NM_138386.1	NAF1	4	164031225	164088073	0.003628178	0.044262479
NM_004359.1	CDC34	19	531733	542084	0.003631673	0.044289332
NM_006832.1	FERMT2	14	53323986	53417815	0.003638561	0.044357524
NM_012279.2	ZNF346	5	176449697	176508190	0.003641421	0.044376576
NM_198439.1	KBTBD3	11	105921825	105948492	0.003645242	0.044377929
NM_022551.2	RPS18	6	33239787	33244287	0.003645248	0.044377929
NM_003707.1	RUVBL1	3	127783625	127872757	0.003645423	0.044377929
NM_020755.2	SERINC1	6	122764496	122907269	0.003653029	0.04445471
NM_001040196.1	AGTRAP	1	11796141	11814859	0.00367106	0.044658259
NM_152931.1	CPNE1	20	34213953	34252878	0.003675821	0.04470028
NM_002896.1	RP11-658F2.1	11	66384097	66413944	0.00368085	0.044745538
NM_005488.1	TOM1	22	35695268	35743985	0.003682913	0.044754719
NM_001012614.1	CTBP1	4	1205236	1243741	0.003687095	0.044762518
NM_014017.2	ROBLD3	1	156024543	156028301	0.003687187	0.044762518
NM_017947.1	MOCOS	18	33767482	33852120	0.003688165	0.044762518
NM_018230.2	NUP133	1	229577044	229644088	0.003688787	0.044762518

NM_024570.1	RNASEH2B	13	51483814	51544592	0.003694366	0.044803118
NM_032608.5	MYO18B	22	26138120	26427007	0.003694752	0.044803118
NM_031314.1	HNRNPC	14	21677295	21737638	0.003697161	0.044816452
NM_004318.2	ASPH	8	62413788	62627199	0.00370185	0.0448574
NM_007097.2	CLTB	5	175819456	175843570	0.003703473	0.044861187
NM_001098784.1	FAM89B	11	65339820	65341669	0.003706476	0.044881667
NM_001019.4	RPS15A	16	18792617	18801656	0.003708943	0.044895661
NM_001001329.1	PRKCSH	19	11546269	11561783	0.003715782	0.044946734
CR603272	RNF168	3	196195654	196230639	0.003715789	0.044946734
NM_001047160.1	NET1	10	5454514	5500426	0.003717262	0.044948664
NM_001086521.1	C17orf89	17	79213111	79215098	0.003722536	0.044959392
NM_080702.2	BAT3	6	31606805	31620482	0.003723408	0.044959392
NM_004153.2	ORC1	1	52838501	52870131	0.00372374	0.044959392
NM_018222.2	PARVA	11	12399026	12552348	0.003725487	0.044959392
NM_001040194.1	AGTRAP	1	11796141	11814859	0.003726899	0.044959392
NM_001037126.1	EXOC4	7	132937829	133751342	0.003727193	0.044959392
NM_015360.3	SKIV2L2	5	54603588	54721409	0.003727346	0.044959392
NM_052875.3	VPS26B	11	134094539	134117686	0.003733437	0.045016984
NM_174928.1	N6AMT2	13	21296543	21348097	0.003737249	0.04504708
NM_138391.4	TMEM183A	1	202976514	202993976	0.003743757	0.04509653
NM_005066.1	SFPQ	1	35641979	35658749	0.003743987	0.04509653
NM_001862.2	COX5B	2	98262503	98264846	0.003749805	0.045150711
NM_001099281.1	HEATR7A	8	145202919	145316843	0.003751736	0.045158075
NM_025027.3	ZNF606	19	58488449	58514714	0.003755246	0.045184425
NM_025204.2	TRABD	22	50624344	50638027	0.00375852	0.045195143
NM_005513.1	GTF2E1	3	120461484	120501916	0.003760004	0.045195143
NM_182684.1	UPK3B	7	76139745	76144773	0.003760099	0.045195143
NM_014515.4	CNOT2	12	70636777	70748773	0.003761876	0.045200633
NM_000754.2	COMT	22	19929130	19957498	0.003775245	0.045345341
NM_005692.3	ABCF2	7	150904923	150924316	0.003776801	0.045348121
NM_152603.2	ZNF567	19	37180302	37212223	0.003780515	0.045376784
NM_000368.3	TSC1	9	135766735	135820020	0.003782415	0.045383672
NM_004716.2	PCSK7	11	117075053	117103241	0.00379022	0.045441129
NM_016142.2	HSD17B12	11	43577986	43878167	0.003791187	0.045441129
NM_015473.2	HEATR5A	14	31760994	31889739	0.003798022	0.045507114
NM_014633.3	CTR9	11	10772534	10801290	0.003806911	0.045584508
NM_016047.3	AC008073.5	2	24290454	24299313	0.003807146	0.045584508
NM_017444.3	CHRAC1	8	141521397	141527236	0.003811171	0.045616742
NM_001005849.1	SUMO2	17	73163825	73179098	0.003821883	0.04572896
NM_139159.3	DPP9	19	4675238	4723875	0.003823333	0.045730327
NM_033084.3	FANCD2	3	10068098	10143614	0.003829035	0.045782519
NM_001001485.1	ATP2C1	3	130569439	130735556	0.003833667	0.045821891
NM_020825.2	CRAMP1L	16	1662338	1736716	0.003836362	0.045838101
NM_198867.1	ALKBH6	19	36500023	36505141	0.003842988	0.045901241
NM_024063.1	SPATA5L1	15	45694529	45826686	0.003849946	0.0459683
NM_177442.1	FTSJ2	7	2273866	2281848	0.003856239	0.046016038
NM_017635.3	SUV420H1	11	67922330	67981295	0.003856634	0.046016038
NM_004156.2	PPP2CB	8	30631973	30671830	0.003858863	0.046026596
NM_004050.2	BCL2L2	14	23776026	23780968	0.003861545	0.046042539
NM_015423.2	AASDHPPT	11	105946228	105969437	0.003870634	0.046134832
NM_052849.2	C15orf57	15	40845298	40857252	0.003874526	0.046147601
NM_032402.1	PCDHGA8	5	140772381	140892546	0.003874855	0.046147601
NM_006367.2	CAP1	1	40505905	40538321	0.003875751	0.046147601

NM_022473.1	ZFP106	15	42705022	42749730	0.003879367	0.046171294
NM_004147.3	DRG1	22	31795509	31924726	0.003880885	0.046171294
NM_001100816.1	MED7	5	156564423	156586030	0.003881789	0.046171294
NM_017801.2	CMTM6	3	32522804	32544900	0.003886265	0.046208473
NM_002074.2	GNB1	1	1716725	1822495	0.003890638	0.046244397
NM_199129.1	TMEM189	20	48697661	48770271	0.003896495	0.046291655
NM_144723.1	ZMAT2	5	140079747	140086266	0.003897319	0.046291655
NM_024996.5	GFM1	3	158362067	158410364	0.003902019	0.0463314
NM_006638.2	RPP40	6	4994966	5004297	0.003922802	0.046562009
NM_003887.1	ASAP2	2	9346894	9545812	0.003929936	0.046630516
NM_053067.1	UBQLN1	9	86274878	86323118	0.003933974	0.046643334
NM_021922.2	FANCE	6	35420138	35434880	0.003935105	0.046643334
NM_006602.2	TCFL5	20	61472467	61493115	0.003940686	0.046693305
NM_006405.5	IPO4	14	24649425	24658124	0.003944858	0.046726566
NM_138408.2	GTF3C6	6	111279763	111289093	0.0039465	0.046729839
NM_031208.2	FAHD1	16	1876968	1890208	0.0039499	0.046753918
NM_013448.2	BAZ1A	14	35221938	35344853	0.00396013	0.046836198
NM_003848.1	SUCLG2	3	67410884	67705038	0.003960621	0.046836198
NM_012414.3	RAB3GAP2	1	220321635	220445796	0.003960957	0.046836198
NM_022753.2	S100PBP	1	33282368	33324476	0.003965464	0.04687329
NM_201542.2	MED8	1	43849588	43855483	0.003968462	0.046884902
NM_014487.2	ZNF330	4	142142041	142155851	0.003969186	0.046884902
NM_001099684.1	FAM156B	23	52920336	52937587	0.00397394	0.046924856
NM_006767.3	LZTR1	22	21333751	21353327	0.003977448	0.046937077
NM_032117.2	MND1	4	154265801	154336270	0.00397978	0.046937077
NM_018682.3	MLL5	7	104654626	104754808	0.003982453	0.046937077
NM_138774.2	C19orf22	19	896503	913225	0.00398273	0.046937077
NM_004477.2	FRG1	4	190861943	190884359	0.003982936	0.046937077
NM_001017921.2	VMAC	19	5904885	5910864	0.003983205	0.046937077
NM_001024603.1	AC004925.1	7	124417512	124430743	0.003985414	0.046946937
NM_022087.2	GALNT11	7	151722759	151820172	0.003988645	0.046947096
NM_013276.2	SHPK	17	3511893	3539616	0.003989631	0.046947096
NM_033198.2	PIGS	17	26880405	26898542	0.003990249	0.046947096
XM_001134465.1	OTUD1	10	23728198	23731310	0.003990915	0.046947096
NM_004593.1	TRA2B	3	185633694	185655924	0.003995011	0.046979134
NM_032924.3	ZNF3	7	99661656	99680171	0.00399905	0.047010473
NM_024658.3	IPO4	14	24649425	24658124	0.004002596	0.047035997
NM_012469.3	PRPF6	20	62612488	62664453	0.00400579	0.047057373
NM_006109.3	PRMT5	14	23389720	23398661	0.004011786	0.047103902
NM_016491.2	MRPL37	1	54649714	54686603	0.004012504	0.047103902
NM_001042416.1	ZNF596	8	182137	197342	0.004020211	0.047151136
NM_021927.1	GUF1	4	44680444	44702943	0.004021629	0.047151136
NM_052902.2	STK11IP	2	220462639	220480915	0.004021782	0.047151136
NM_012180.2	FBXO8	4	175157809	175205415	0.004022039	0.047151136
NM_000454.4	SOD1	21	33031935	33041244	0.004024077	0.047158875
NM_001080501.1	TMEM223	11	62539101	62559493	0.004042658	0.047360415
NM_006423.1	RABAC1	19	42460838	42463528	0.004046383	0.047387827
NM_139030.3	CD151	11	832843	839831	0.004048813	0.047397503
NM_018367.4	ACER3	11	76571911	76737841	0.00404998	0.047397503
NM_144638.1	TMEM42	3	44903361	44907162	0.00405568	0.047420832
NM_015134.2	MPRIP	17	16946074	17088874	0.004056069	0.047420832
NM_182639.1	HPS1	10	100175955	100206709	0.004057028	0.047420832
NM_138431.1	MFSD3	8	145734457	145736569	0.004057516	0.047420832

NM_138553.1	BCL11A	2	60678302	60780702	0.00406405	0.047464776
NM_015332.3	NUDCD3	7	44421969	44530479	0.004067484	0.047469118
NR_003032.1	TAF1D	11	93463114	93517557	0.004068102	0.047469118
NM_024294.2	C6orf106	6	34555065	34664636	0.004068939	0.047469118
NM_152326.2	ANKRD9	14	102973198	102976128	0.004071125	0.047469118
NM_006608.1	PHTF1	1	114239453	114302111	0.004071358	0.047469118
NM_144974.3	CCDC122	13	44398045	44453864	0.004073749	0.047480819
NM_006454.2	MXD4	4	2249159	2264021	0.004085689	0.047600415
NM_017730.2	QRICH1	3	49067140	49131796	0.004086792	0.047600415
NM_005628.1	SLC1A5	19	47278140	47291851	0.004089409	0.04761419
NM_001044385.1	ALS2CR4	2	202484907	202508293	0.004091518	0.04761419
NM_022459.3	XPO4	13	21351469	21477187	0.004092149	0.04761419
NM_000466.1	PEX1	7	92116334	92157845	0.004095303	0.047620768
NM_145808.1	MTPN	7	135611509	135662107	0.004095497	0.047620768
NM_006824.1	EBNA1BP2	1	43629855	43727589	0.004098483	0.047630488
NM_198513.1	PHF20L1	8	133787618	133861052	0.004099117	0.047630488
NM_001037663.1	EEF1B2	2	207024309	207027652	0.004101579	0.04764292
NM_006238.2	PPARD	6	35310335	35395955	0.004103502	0.047649086
NM_002651.1	PI4KB	1	151264273	151300191	0.004109812	0.047689987
NM_018449.2	UBAP2	9	33921691	34048947	0.004125208	0.047852415
NM_001099858.1	PSMA2	7	42948872	42971822	0.00413198	0.047910453
NM_001002010.1	NT5C3	7	33053742	33102409	0.004133012	0.047910453
NM_001031685.2	TP53BP2	1	223967596	224033674	0.004138588	0.047958854
NM_006368.4	CREB3	9	35732332	35737001	0.004140712	0.047961831
NM_007215.2	POLG2	17	62473904	62493169	0.004141648	0.047961831
NM_012397.2	SERPINB13	18	61254223	61271873	0.0041452	0.047965872
NM_003168.1	SUPT4H1	17	56422539	56429563	0.004146037	0.047965872
NM_138926.1	SON	21	34914924	34949812	0.004146203	0.047965872
NM_004180.2	TANK	2	161993419	162092732	0.004147621	0.047966065
NM_032314.3	COQ5	12	120941083	120966964	0.004149675	0.047973609
NM_147202.1	C9orf24	9	34379017	34397849	0.004159876	0.048056892
NM_174893.1	C17orf49	17	6918073	6920838	0.004161071	0.048056892
NM_022818.3	MAP1LC3B	16	87425406	87438385	0.004161092	0.048056892
NM_144973.3	DENND5B	12	31535157	31743952	0.004164651	0.048064552
XR_015176.2	TYW1	7	66460160	66704501	0.004166313	0.048064552
NM_001099415.1	POM121C	7	75046066	75115548	0.004166343	0.048064552
NM_004661.3	CDC23	5	137523339	137549032	0.004167374	0.048064552
NM_001024844.1	CD82	11	44585977	44641913	0.004169913	0.048077641
NM_031480.2	RIOK1	6	7389729	7418270	0.004177737	0.048143647
NM_012200.2	B3GAT3	11	62382768	62389647	0.004179565	0.048143647
NM_006156.2	NEDD8	14	24686058	24701660	0.004181095	0.048143647
NM_014906.3	PPM1E	17	56833232	57062532	0.004181266	0.048143647
NM_015948.2	SLC35B3	6	8413301	8435794	0.004182733	0.048144338
NM_032433.2	ZNF333	19	14800870	14831772	0.004190118	0.048213131
NM_032982.2	CASP2	7	142985308	143004789	0.004194752	0.048250227
NM_207012.2	AP3M1	10	75880016	75910821	0.004197018	0.048260065
NM_000980.2	RPL18A	19	17970731	17974124	0.004201317	0.048293266
NM_001003714.1	ATP5J2	7	99046098	99063954	0.004210659	0.048358792
NM_024874.3	KIAA0319L	1	35899091	36023551	0.004210685	0.048358792
CR603450	TRIM52	5	180681417	180688119	0.004211257	0.048358792
NM_004854.3	CHST10	2	101008327	101034118	0.004221091	0.048443062
NM_025238.3	BTBD1	15	83685174	83736106	0.004221426	0.048443062
NM_001002857.1	ANXA2	15	60639333	60690185	0.004222859	0.048443262

NM_017991.3	KIAA1310	2	97258907	97308524	0.004228372	0.04849024
NM_016462.2	TMEM14C	6	10723208	10731362	0.004232956	0.048526556
NM_033546.2	MYL12B	18	3261907	3278282	0.004237019	0.048547626
NM_012334.1	MYO10	5	16662019	16936385	0.004237632	0.048547626
NM_021639.3	GPBP1L1	1	46092976	46153785	0.004239767	0.048555828
NM_170783.1	ZNRD1	6	30026676	30032686	0.004241312	0.048557276
NM_021970.2	MAPKSP1	4	100799493	100815647	0.004242903	0.048559242
NM_020457.2	THAP11	16	67876213	67878097	0.004244706	0.048563636
NM_015199.2	ANKRD28	3	15708743	15901278	0.00424699	0.048573523
NM_002836.2	VPS16	20	2821349	2847378	0.004248725	0.048577132
NM_025015.2	HSPA12A	10	118430703	118502085	0.004252313	0.048601919
NM_000964.2	RARA	17	38465436	38513895	0.004255203	0.04860695
NM_001092.3	ABR	17	906758	1083131	0.004255594	0.04860695
NM_182679.1	GPATCH4	1	156564279	156571288	0.004258993	0.048629547
NM_006585.2	CCT8	21	30428126	30446118	0.004260875	0.048634803
NM_001037163.1	C7orf70	7	6369040	6388612	0.004265668	0.048638076
NM_002787.3	PSMA2	7	42948872	42971822	0.004265959	0.048638076
XM_927974.2	PGPEP1L	15	99511509	99548791	0.004266242	0.048638076
NM_005273.2	GNB2	7	100271154	100276797	0.004266847	0.048638076
NM_003432.1	ZNF131	5	43065278	43192123	0.004269579	0.048653008
NM_001087.3	AAMP	2	219128850	219134980	0.004271421	0.0486578
NM_001731.1	BTG1	12	92534074	92539673	0.004278341	0.048720405
NM_001015045.1	FAM13A	4	89647106	90032549	0.004280807	0.048732273
NM_005653.3	TFCP2	12	51488624	51566664	0.004287343	0.048779598
NM_002567.2	PEBP1	12	118573870	118583387	0.004289236	0.048779598
NM_004781.3	VAMP3	1	7831329	7841492	0.004289241	0.048779598
XM_942544.2	INTS1	7	1509913	1545489	0.004294324	0.048821185
NM_015367.2	BCL2L13	22	18111621	18213388	0.004301925	0.048891357
NM_005862.2	STAG1	3	136055077	136471220	0.004303849	0.048896986
NM_002708.3	PPP1CA	11	67165654	67188605	0.004307573	0.048923044
NM_015200.1	PDS5A	4	39824483	39979576	0.004310975	0.048927019
NM_004099.4	STOM	9	124101355	124132531	0.004313419	0.048927019
NM_005652.2	TERF2	16	69389464	69419859	0.00431412	0.048927019
NM_015286.5	SYNM	15	99645286	99675770	0.0043144	0.048927019
NM_024513.1	FYCO1	3	45959396	46037316	0.004315201	0.048927019
NM_007222.3	ZHX1	8	124260697	124287781	0.004316501	0.048927019
NM_020803.3	KLHL8	4	88081255	88141760	0.004320665	0.048958003
NM_198155.2	C21orf33	21	45553487	45565605	0.004328192	0.04902226
NM_022749.5	FAM160B2	8	21946670	21962232	0.004329201	0.04902226
NM_001007464.1	RWDD1	6	116892530	116918838	0.004331044	0.049026903
NM_004434.2	EML1	14	100259745	100408393	0.004342491	0.049140226
NM_001099645.1	RPL22L1	3	170582664	170588272	0.004345556	0.049158659
NM_005786.4	TSHZ1	18	72922752	73001905	0.004353097	0.049227682
NM_018983.3	GAR1	4	110736658	110745893	0.004363579	0.049327162
NM_002588.2	PCDHGC3	5	140855580	140892542	0.004364776	0.049327162
NM_006047.4	RBM12	20	34236847	34252878	0.004374319	0.049412515
XM_001126202.1	SPRY1	4	124317950	124324910	0.004375217	0.049412515
NM_001013649.1	C2orf68	2	85833777	85839189	0.004378734	0.049435917
NM_012191.2	NAT6	3	50333833	50336852	0.004380366	0.049438041
NM_020649.1	CBX8	17	77765931	77775482	0.004389891	0.049523808
NM_020335.1	VANGL2	1	160370376	160398468	0.004392448	0.049523808
NM_020135.2	WRNIP1	6	2765648	2786927	0.004393052	0.049523808
NM_001097590.1	RPP38	10	15139179	15148129	0.004393755	0.049523808

NM_021058.3	HIST1H2BJ	6	27100095	27100563	0.004403067	0.04960177
NM_030978.1	ARPC5L	9	127624409	127640003	0.004404701	0.04960177
NM_018235.1	CNDP2	18	72163443	72188363	0.004405424	0.04960177
NM_006405.5	TM9SF1	14	24658349	24664893	0.004407949	0.04960177
NM_015288.4	PHF15	5	133860003	133918918	0.00440853	0.04960177
NM_203494.3	USP50	15	50792759	50838905	0.004409368	0.04960177
NM_183050.1	BCKDHB	6	80816364	81055971	0.00441179	0.049612702
NM_006185.2	NUMA1	11	71713911	71791573	0.004415904	0.049642649
NM_015058.1	KIAA0564	13	42140961	42535256	0.004420339	0.049648832
NM_001115016.1	KIAA1310	2	97258907	97308524	0.004420517	0.049648832
NM_014961.2	RUFY3	4	71569921	71673032	0.004420806	0.049648832
NM_032306.2	ALKBH7	19	6372444	6375040	0.004424656	0.049648852
AA628763	AL139008.5	9	33638033	33638492	0.00442475	0.049648852
NM_144964.2	RG9MTD3	9	37753802	37778969	0.004425161	0.049648852
NM_001347.2	DGKQ	4	952675	980683	0.004427188	0.049655316
NM_013283.3	MAT2B	5	162930120	162946342	0.004431322	0.049685398
NM_001031693.2	HHLA3	1	70820488	70851022	0.004437759	0.049741278
NM_016096.3	ZNF706	8	102190106	102218421	0.004441527	0.049767204
NM_014390.2	SND1	7	127292202	127732661	0.004444003	0.049776039
NM_005433.3	YES1	18	721588	812327	0.004445224	0.049776039
NM_133436.1	ASNS	7	97481440	97501854	0.004449363	0.04977671
NM_018360.1	TXLNG	23	16804550	16862642	0.0044496	0.04977671
NM_145294.4	WDR90	16	699363	717829	0.004449648	0.04977671
NM_024755.2	SLTM	15	59171244	59225886	0.004456688	0.049839168
NM_030800.1	C15orf44	15	65871095	65903474	0.004459521	0.049854559
NM_001080425.1	BEX4	23	102470020	102472174	0.004466117	0.049886215
NM_172037.2	RDH10	8	74206847	74237516	0.004467241	0.049886215
XM_001134299.1	PRIM2	6	57179603	57513375	0.004467757	0.049886215
NM_001037334.1	USP14	18	158480	213739	0.004469263	0.049886215
NM_003945.3	ATP6V0E1	5	172410760	172462448	0.004469642	0.049886215
AI911352	AL139008.5	9	33638033	33638492	0.004473415	0.049894708
NM_004813.1	PEX16	11	45931220	45940363	0.004473563	0.049894708
NM_015999.2	ADIPOR1	1	202909951	202927700	0.004482748	0.049967313
NM_002503.3	NFKBIB	19	39390615	39399531	0.004484842	0.049974372
NM_033378.1	CGB2	19	49535169	49536495	0.004474777	0.049894708
NM_001007089.2	RESP18	2	220192131	220197899	0.00410865	0.047689987
NM_000525.3	KCNJ11	11	17406798	17410878	0.004060205	0.047436061
NM_013370.3	OSGIN1	16	83982672	83999937	0.003934292	0.046643334
NM_001765.2	CD1C	1	158259563	158264564	0.003790218	0.045441129
NM_144497.1	AKAP12	6	151561134	151679692	0.003622822	0.044228673
NM_005978.3	S100A2	1	153533584	153540366	0.003329282	0.041909796
NM_016540.2	GPR83	11	94110477	94134585	0.003221789	0.041153272
NM_014410.4	CLUL1	18	596998	650291	0.002506667	0.034925953
XM_944915.1	PTP4A2	1	32372022	32410457	0.002290487	0.033055192
NM_022128.1	RBKS	2	28004231	28113965	0.001281424	0.02317671
NM_030569.3	ITIH5	10	7601232	7708934	0.001010027	0.020186322
NM_018711.2	SVOP	12	109304658	109372440	0.000788793	0.017302878
BX101503	CXorf28	23	3189861	3202694	0.000711397	0.016393268
NM_004405.3	DLX2	2	172964166	172967628	0.000640349	0.015398983
NM_005215.2	DCC	18	49867158	51062269	0.000466144	0.012994448
NM_052939.3	FCRL3	1	157646271	157670775	0.000288712	0.01007107
NM_001001657.1	OR2Y1	5	180166042	180167099	0.000260706	0.009500923
NM_001007241.1	GP2	16	20320896	20338902	0.000224012	0.00866405

NM_000729.3	CCK	3	42299317	42307699	9.32E-05	0.005244285
NM_181600.1	KRTAP13-4	21	31802572	31803216	7.82E-05	0.004672725
