# Supplementary material for: Genomic and transcriptomic heterogeneity in metaplastic carcinomas of the breast
Source: NPJ Breast Cancer. 2017 Dec 1;3:48. doi: 10.1038/s41523-017-0048-0 (PMC5711926; doi:10.1038/s41523-017-0048-0)
Supplement: Supplementary file 29 — Supplementary Table 17 [file 41523_2017_48_MOESM29_ESM.pdf]

Supplementary Table 17: List of genes overexpressed when amplified in metaplastic carcinomas of the breast.

HUGO Gene Symbol	Chromosome	Start	End	Amplification fold	Wilcox.p.amp	Wilcox.adjp.amp
ZNF7	8	146052849	146072894	2.664606702	8.23E-05	0.004566022
MAF1	8	145159402	145162514	2.050969192	8.23E-05	0.004566022
RPL8	8	146015150	146017972	1.888677797	8.23E-05	0.004566022
VPS28	8	145649000	145653931	2.353514095	8.23E-05	0.004566022
ZNF16	8	146155744	146176274	1.783839352	8.23E-05	0.004566022
SLC39A4	8	145635126	145642279	2.06778749	8.23E-05	0.004566022
ANKRD46	8	101521980	101572012	2.520488146	8.23E-05	0.004566022
VPS28	8	145649000	145653931	2.189081966	8.23E-05	0.004566022
EXOSC4	8	145133529	145135550	2.868230912	8.23E-05	0.004566022
PYCRL	8	144686083	144691943	2.063382711	8.23E-05	0.004566022
CYHR1	8	145674965	145691060	1.916900687	8.23E-05	0.004566022
SHARPIN	8	145153536	145163027	2.22460353	8.23E-05	0.004566022
FBXL6	8	145579091	145583036	1.754010944	8.23E-05	0.004566022
TTC35	8	109455830	109499145	2.446157747	8.23E-05	0.004566022
PABPC1	8	101698044	101735037	2.15714186	0.000164541	0.004917255
OTUD6B	8	92082424	92099323	1.855328868	0.000161603	0.004917255
COX6C	8	100885428	100906290	2.232025789	0.000164541	0.004917255
ZC3H3	8	144519825	144623623	1.973194487	0.000164541	0.004917255
POLR2K	8	101162812	101166230	2.543026611	0.000164541	0.004917255
CYC1	8	145149930	145152428	2.471349404	0.000164541	0.004917255
NBN	8	90945564	91015456	2.387625742	0.000161603	0.004917255
GPR172A	8	145577795	145584932	2.116933945	0.000164541	0.004917255
YWHAZ	8	101930804	101965616	1.697059099	0.000164541	0.004917255
ZNF623	8	144718183	144738588	1.509334792	0.000164541	0.004917255
LRRC14	8	145743376	145750557	2.242677637	0.000164541	0.004917255
SNX16	8	82711816	82755101	2.441315665	0.000102838	0.004917255
RB1CC1	8	53535016	53658403	2.862149803	0.000323206	0.005327026
ARMC1	8	66514694	66546442	2.167799834	0.000323206	0.005327026
CSPP1	8	67974661	68108498	2.303025974	0.000323206	0.005327026
LACTB2	8	71547553	71581409	2.247929089	0.000323206	0.005327026
RRS1	8	67341263	67342966	3.889829833	0.000323206	0.005327026
ARFGEF1	8	68085747	68255912	2.814007304	0.000323206	0.005327026
MTFR1	8	66556124	66683496	2.57083952	0.000323206	0.005327026
TCEA1	8	54879112	54935089	2.312703933	0.000323206	0.005327026
GPAA1	8	145137493	145141119	2.194042911	0.000329083	0.005327026
RPL7	8	74202506	74213303	3.050711621	0.000323206	0.005327026
KIAA0196	8	126036502	126104082	2.102942073	0.000329083	0.005327026
C8orf33	8	146277764	146281416	2.891859151	0.000329083	0.005327026
C8orf82	8	145751117	145754516	1.875600032	0.000329083	0.005327026
EIF3E	8	109213445	109447562	1.704205974	0.000329083	0.005327026
PEX2	8	77892494	77913280	2.397612636	0.000323206	0.005327026
MRPL15	8	55047770	55060461	2.795996103	0.000323206	0.005327026
TCEA1	8	54879112	54935089	1.670383448	0.000323206	0.005327026
VCPIP1	8	67540722	67579452	2.094472665	0.000323206	0.005327026

ATP6V1C1	8	104033291	104085279	1.927453671	0.000329083	0.005327026
ARFGEF1	8	68085747	68255912	4.281590814	0.000323206	0.005327026
YWHAZ	8	101930804	101965616	3.491556674	0.000329083	0.005327026
OSGIN2	8	90914087	90940116	3.443327959	0.000323206	0.005327026
MTERFD1	8	97251626	97273838	1.918077432	0.000411353	0.006522889
PHF20L1	8	133787618	133861052	2.699309776	0.000575895	0.00758424
ZNF696	8	144371846	144380231	2.206766615	0.000575895	0.00758424
SQLE	8	126010739	126034525	2.412977678	0.000575895	0.00758424
SCRIB	8	144873090	144897549	2.488644226	0.000575895	0.00758424
C8orf33	8	146277764	146281416	3.188261783	0.000575895	0.00758424
FBXL6	8	145579091	145583036	2.122138437	0.000575895	0.00758424
ZNF517	8	146024261	146036554	1.627718862	0.000575895	0.00758424
TSTA3	8	144694788	144700218	1.803719413	0.000575895	0.00758424
TIGD5	8	144680005	144682485	1.915998124	0.000575895	0.00758424
ZNF251	8	145946298	145981802	1.410404564	0.000575895	0.00758424
LYPLA1	8	54958938	55014577	2.281920399	0.000646412	0.007610037
COPS5	8	67955314	67996018	2.108499473	0.000646412	0.007610037
ZFAND1	8	82613569	82645138	2.64764108	0.000646412	0.007610037
CHCHD7	8	57124245	57131357	2.568197229	0.000646412	0.007610037
RAB2A	8	61429416	61536186	2.25838167	0.000646412	0.007610037
ATP6V1H	8	54628117	54756118	2.213500843	0.000646412	0.007610037
CHD7	8	61591337	61779465	2.875064443	0.000646412	0.007610037
RPL30	8	99037079	99058697	1.731843535	0.000719868	0.008106344
CPNE3	8	87497059	87573726	1.956322957	0.000719868	0.008106344
MTERFD1	8	97251626	97273838	1.902932724	0.000719868	0.008106344
NSMCE2	8	126103921	126379362	1.833033268	0.000987248	0.009588647
TRMT12	8	125463048	125474391	2.131900308	0.000987248	0.009588647
SQLE	8	126010739	126034525	3.193451581	0.000987248	0.009588647
WDYHV1	8	124428965	124479470	1.704033411	0.000987248	0.009588647
DERL1	8	124025458	124054663	1.741592586	0.000987248	0.009588647
COMMD5	8	146066427	146079121	1.851298595	0.000987248	0.009588647
PUF60	8	144898514	144912029	2.224197097	0.000987248	0.009588647
EIF3H	8	117654369	117779164	1.912878843	0.000987248	0.009588647
VPS13B	8	100025494	100889808	3.270474534	0.000987248	0.009588647
ZNF707	8	144766622	144796068	1.64846797	0.000987248	0.009588647
LYNX1	8	143845752	143859640	1.90848281	0.000987248	0.009588647
TMEM64	8	91634223	91803860	2.164303967	0.001131222	0.010851349
PTDSS1	8	97273943	97349223	1.969188542	0.00123406	0.011693472
NCOA2	8	71015826	71316040	2.234622036	0.001292825	0.01195863
CSPP1	8	67974661	68108498	2.520611229	0.001292825	0.01195863
MRPL13	8	121393000	121457642	1.822759159	0.001563143	0.012784862
PTK2	8	141667999	142012315	2.04792879	0.001563143	0.012784862
TATDN1	8	125500726	125551699	2.39263648	0.001563143	0.012784862
OXR1	8	107282473	107764922	2.330579572	0.001563143	0.012784862
ENY2	8	110346553	110358182	1.942737322	0.001563143	0.012784862
TOP1MT	8	144386554	144442149	1.706456293	0.001563143	0.012784862
RAD21	8	117858174	117887105	2.546393025	0.001563143	0.012784862
AZIN1	8	103838585	103906092	1.829309591	0.001563143	0.012784862

SCRIB	8	144873090	144897549	1.676840015	0.001563143	0.012784862
EIF2C2	8	141541264	141645718	2.121064656	0.001563143	0.012784862
RNF19A	8	101269288	101348446	2.189455972	0.001563143	0.012784862
THAP1	8	42691817	42698468	1.377185897	0.001680672	0.013602941
OTUD6B	8	92082424	92099323	2.480988249	0.001939237	0.014739829
FAM82B	8	87480486	87526586	2.251858828	0.001953928	0.014739829
NBN	8	90945564	91015456	2.595266429	0.001939237	0.014739829
POP1	8	99129525	99172062	2.541148207	0.001953928	0.014739829
ZNF704	8	81540686	81787016	3.283366675	0.001939237	0.014739829
C8orf83	8	93895758	94029901	1.82747232	0.001939237	0.014739829
RBM12B	8	94741584	94753245	2.319564875	0.001939237	0.014739829
UBE2W	8	74650409	74791145	1.972578891	0.002262443	0.016115373
GRINA	8	145064226	145067583	1.925797512	0.00246812	0.016115373
TATDN1	8	125500726	125551699	2.069186295	0.00246812	0.016115373
UBE2V2	8	48920960	48976511	2.125271132	0.002262443	0.016115373
ZFP41	8	144328991	144358573	3.398808889	0.00246812	0.016115373
ZNF34	8	145997611	146012730	2.705351319	0.00246812	0.016115373
MED30	8	118532952	118552501	2.314389737	0.00246812	0.016115373
LYPLA1	8	54958938	55014577	2.564353865	0.002262443	0.016115373
RPL8	8	146015150	146017972	2.597292204	0.00246812	0.016115373
UTP23	8	117778742	117861702	2.002030926	0.00246812	0.016115373
TERF1	8	73921099	73960357	2.9712975	0.002262443	0.016115373
NUDCD1	8	110253148	110346614	2.149907973	0.00246812	0.016115373
PHF20L1	8	133787618	133861052	1.825791682	0.00246812	0.016115373
ZNF16	8	146155744	146176274	2.011325307	0.00246812	0.016115373
SNAI2	8	49830249	49834299	2.790818736	0.002262443	0.016115373
C8orf55	8	143808621	143818345	2.206207318	0.00246812	0.016115373
GOLGA7	8	41347915	41368499	1.81794588	0.002941176	0.018886728
MYST3	8	41786997	41909508	2.469656589	0.002941176	0.018886728
STK3	8	99413631	99955055	1.764739142	0.00308515	0.019177293
ZFAND1	8	82613569	82645138	2.197368889	0.003070459	0.019177293
E2F5	8	86089460	86129387	2.089306723	0.00308515	0.019177293
FAM82B	8	87480486	87526586	1.824245799	0.00308515	0.019177293
ADCK5	8	145596790	145618457	1.66372886	0.00370218	0.021467119
NDUFB9	8	125551344	125580751	2.46203472	0.00370218	0.021467119
HSF1	8	145515270	145538385	1.801091392	0.00370218	0.021467119
TAF2	8	120743015	120845103	1.643850496	0.00370218	0.021467119
GRINA	8	145064226	145067583	2.520226943	0.00370218	0.021467119
DGAT1	8	145539954	145550573	1.699993648	0.00370218	0.021467119
WDYHV1	8	124428965	124479470	1.69656825	0.00370218	0.021467119
ZNF250	8	146092748	146127553	1.605984381	0.00370218	0.021467119
SCXB	8	145321517	145323045	1.854223865	0.00370218	0.021467119
TCEB1	8	74857354	74884522	2.464766189	0.003878474	0.021680393
TERF1	8	73921099	73960357	2.460830881	0.003878474	0.021680393
MYBL1	8	67474414	67526482	2.781323263	0.003878474	0.021680393
TCEA1	8	54879112	54935089	1.721313491	0.003878474	0.021680393
SNAI2	8	49830249	49834299	2.899068809	0.003878474	0.021680393
HRSP12	8	99114572	99129469	1.773580946	0.004627725	0.025683875

MRPS28	8	80830952	80942524	2.23802421	0.004848093	0.026527946
FAM164A	8	79578282	79629935	2.037241104	0.004848093	0.026527946
C8orf76	8	124232196	124279627	1.763024316	0.005512135	0.028177163
UBR5	8	103264501	103425069	1.57525855	0.005512135	0.028177163
PHF20L1	8	133787618	133861052	2.172789202	0.005512135	0.028177163
BOP1	8	145486055	145515082	2.185749815	0.005512135	0.028177163
TRAPPC9	8	140742586	141468678	1.648114242	0.005512135	0.028177163
PABPC1	8	101698044	101735037	2.119043427	0.005512135	0.028177163
NRBP2	8	144915764	144924200	2.233025737	0.005512135	0.028177163
OPLAH	8	145106167	145115606	1.857997537	0.005512135	0.028177163
ASAP1	8	131064353	131455906	1.974633968	0.005512135	0.028177163
C8orf30B	8	145437880	145440953	1.662653467	0.005512135	0.028177163
TCEB1	8	74857354	74884522	1.956230716	0.006140918	0.029821833
AC103686.1	8	48685669	48872743	2.234633544	0.006140918	0.029821833
JPH1	8	75146935	75233563	4.964036598	0.006140918	0.029821833
MCM4	8	48872745	48890720	2.063505229	0.006140918	0.029821833
TRAM1	8	71485677	71520622	1.484695497	0.006140918	0.029821833
CHCHD7	8	57124245	57131357	2.496663177	0.006140918	0.029821833
AC103686.1	8	48685669	48872743	1.968964201	0.006140918	0.029821833
RDH10	8	74206847	74237516	3.155359238	0.006140918	0.029821833
MTDH	8	98656407	98740998	1.744924149	0.00678733	0.032157047
LAPTM4B	8	98787285	98865241	1.923352839	0.00678733	0.032157047
LAPTM4B	8	98787285	98865241	1.931377927	0.00678733	0.032157047
FAM82B	8	87480486	87526586	1.968417772	0.00678733	0.032157047
IMPA1	8	82570196	82598928	1.825263216	0.007110537	0.033282451
TMEM64	8	91634223	91803860	2.450296982	0.007110537	0.033282451
PTP4A3	8	142402093	142441620	1.790757779	0.007897984	0.035067051
ARHGAP39	8	145754563	145831201	3.837535283	0.007897984	0.035067051
OPLAH	8	145106167	145115606	3.569369945	0.007897984	0.035067051
MAPK15	8	144798429	144804628	6.280172186	0.007897984	0.035067051
TOP1MT	8	144386554	144442149	2.562855181	0.007897984	0.035067051
ST3GAL1	8	134467091	134584183	1.883641339	0.007897984	0.035067051
VPS13B	8	100025494	100889808	1.64338712	0.007897984	0.035067051
ST3GAL1	8	134467091	134584183	2.097515859	0.007897984	0.035067051
RNF19A	8	101269288	101348446	2.132504998	0.007897984	0.035067051
AC103686.1	8	48685669	48872743	1.577234073	0.00937298	0.040236494
ASPH	8	62413788	62627199	1.57593253	0.00937298	0.040236494
CRISPLD1	8	75896750	75946793	5.789375183	0.00937298	0.040236494
TMEM68	8	56608983	56685966	1.459465295	0.00937298	0.040236494
NSMAF	8	59496063	59572403	1.524459942	0.00937298	0.040236494
CRISPLD1	8	75896750	75946793	13.64736294	0.00937298	0.040236494
FNTA	8	42889337	42940931	2.169706757	0.010084034	0.04258312
HOOK3	8	42752075	42883255	1.532255941	0.010084034	0.04258312
HOOK3	8	42752075	42883255	3.236425126	0.010084034	0.04258312
DECR1	8	91013633	91064320	1.566492949	0.010342599	0.043438914
PHF20L1	8	133787618	133861052	1.401722387	0.011106541	0.044483412
UTP23	8	117778742	117861702	1.898859369	0.011106541	0.044483412
COMMD5	8	146066427	146079121	1.427177803	0.011106541	0.044483412

CPSF1	8	145618444	145634753	1.78155709	0.011106541	0.044483412
HEATR7A	8	145202919	145316843	1.383479297	0.011106541	0.044483412
MFSD3	8	145734457	145736569	2.008856301	0.011106541	0.044483412
PPP1R16A	8	145703352	145727504	2.783841335	0.011106541	0.044483412
EBAG9	8	110551940	110578225	1.670353755	0.011106541	0.044483412
PLEC	8	144989321	145050902	1.310638325	0.011106541	0.044483412
AGPAT6	8	41434706	41482520	1.736368247	0.011764706	0.046638655
SLC20A2	8	42273993	42397069	2.354523274	0.011764706	0.046638655
