# Supplementary material for: Genomic and transcriptomic heterogeneity in metaplastic carcinomas of the breast
Source: NPJ Breast Cancer. 2017 Dec 1;3:48. doi: 10.1038/s41523-017-0048-0 (PMC5711926; doi:10.1038/s41523-017-0048-0)
Supplement: Supplementary file 30 — Supplementary Table 18 [file 41523_2017_48_MOESM30_ESM.pdf]

Supplementary Table 18: Pathway analysis of the genes overexpressed when amplified using g:Profiler.

Number of Query Genes	Number of Term Genes	Number of Common Genes	Term ID	Source	Description	Genes in Overlap	*p*-value
6005	140	68	GO:0044249	BP	Cellular biosynthetic process	ST3GAL1,SNAI2,RB1CC1,NSMAF,TAF2,ARFGEF1,TRAM1,PABPC1,CPSF1,MYST3,CPNE3,NBN,STK3,EIF3E,TSTA3,PYCRL,MCM4,ENY2,COPS5,RDH10,THAP1,HRSP12,IMPA1,E2F5,MRPL15,NCOA2,GOLGA7,MRPS28,TERF1,RPL7,MTDH,POLR2K,EIF3H,ZNF7,TCEB1,AZIN1,PTDSS1,RPL30,AGPAT6,RPL8,PEX2,RAD21,MED30,COX6C,YWHAZ,TRAPPC9,COMMD5,ZNF16,CHD7,MRPL13,OPLAH,MAF1,PUF60,MAPK15,ZNF707,ZNF623,TOP1MT,DGAT1,HSF1,MYBL1,ZNF696,TCEA1,ZNF250,ZNF34,ZNF517,GPAA1,ZNF251,ASPH	3.40E-02
6457	140	73	GO:0034641	BP	Cellular nitrogen compound metabolic process	ST3GAL1,ZC3H3,SNAI2,RB1CC1,NSMAF,TAF2,TRAM1,PABPC1,CPSF1,MYST3,NBN,UBE2W,POP1,STK3,EIF3E,UBR5,TSTA3,PYCRL,MCM4,ENY2,COPS5,THAP1,HRSP12,E2F5,MRPL15,NCOA2,MRPS28,TERF1,RPL7,MTDH,POLR2K,EIF3H,UTP23,NDUFB9,TATDN1,ZNF7,TCEB1,AZIN1,RPL30,NSMCE2,AGPAT6,RPL8,PEX2,RAD21,MED30,COX6C,YWHAZ,TRAPPC9,UBE2V2,COMMD5,ZNF16,CHD7,MRPL13,OPLAH,EXOSC4,RRS1,MAF1,PUF60,MAPK15,ZNF707,ZNF623,TRMT12,TOP1MT,HSF1,MYBL1,ZNF696,TCEA1,ZNF250,ZNF34,ZNF517,ZNF251,ASPH,BOP1	1.16E-02
10602	140	106	GO:0043231	CC	Intracellular membrane-bounded organelle	ST3GAL1,ZC3H3,SNAI2,RB1CC1,ATP6V1H,TAF2,ARFGEF1,MTFR1,TRAM1,PABPC1,CPSF1,MYST3,CPNE3,NBN,DECR1,UBE2W,POP1,JPH1,STK3,RAB2A,EIF3E,ARMC1,SNX16,UBR5,SQLE,MCM4,NUDCD1,ENY2,LYPLA1,COPS5,RDH10,THAP1,HRSP12,E2F5,DERL1,MRPL15,NCOA2,GOLGA7,MRPS28,LACTB2,TERF1,RPL7,MTDH,EBAG9,POLR2K,UTP23,NDUFB9,TATDN1,ZNF7,ARHGAP39,SLC39A4,TCEB1,AZIN1,ATP6V1C1,PTDSS1,RPL30,WDYHV1,NSMCE2,AGPAT6,VPS28,RPL8,PEX2,RAD21,MED30,OXR1,COX6C,YWHAZ,KIAA0196,TRAPPC9,HOOK3,UBE2V2,PTK2,COMMD5,ZNF16,CHCHD7,CHD7,MRPL13,VCPIP1,GRINA,EXOSC4,RRS1,CYC1,SHARPIN,MAF1,TIGD5,PUF60,TMEM64,SCRIB,MAPK15,ZNF707,ZNF623,TOP1MT,PTP4A3,DGAT1,HSF1,MYBL1,ZNF696,TCEA1,CYHR1,ZNF250,ZNF34,ZNF517,GPAA1,ZNF251,ASPH,BOP1	9.66E-04
6110	140	69	GO:1901576	BP	Organic substance biosynthetic process	ST3GAL1,SNAI2,RB1CC1,NSMAF,TAF2,ARFGEF1,TRAM1,PABPC1,CPSF1,MYST3,CPNE3,NBN,STK3,EIF3E,TSTA3,PYCRL,SQLE,MCM4,ENY2,COPS5,RDH10,THAP1,HRSP12,IMPA1,E2F5,MRPL15,NCOA2,GOLGA7,MRPS28,TERF1,RPL7,MTDH,POLR2K,EIF3H,ZNF7,TCEB1,AZIN1,PTDSS1,RPL30,AGPAT6,RPL8,PEX2,RAD21,MED30,COX6C,YWHAZ,TRAPPC9,COMMD5,ZNF16,CHD7,MRPL13,OPLAH,MAF1,PUF60,MAPK15,ZNF707,ZNF623,TOP1MT,DGAT1,HSF1,MYBL1,ZNF696,TCEA1,ZNF250,ZNF34,ZNF517,GPAA1,ZNF251,ASPH	3.02E-02
6191	140	69	GO:0009058	BP	Biosynthetic process	ST3GAL1,SNAI2,RB1CC1,NSMAF,TAF2,ARFGEF1,TRAM1,PABPC1,CPSF1,MYST3,CPNE3,NBN,STK3,EIF3E,TSTA3,PYCRL,SQLE,MCM4,ENY2,COPS5,RDH10,THAP1,HRSP12,IMPA1,E2F5,MRPL15,NCOA2,GOLGA7,MRPS28,TERF1,RPL7,MTDH,POLR2K,EIF3H,ZNF7,TCEB1,AZIN1,PTDSS1,RPL30,AGPAT6,RPL8,PEX2,RAD21,MED30,COX6C,YWHAZ,TRAPPC9,COMMD5,ZNF16,CHD7,MRPL13,OPLAH,MAF1,PUF60,MAPK15,ZNF707,ZNF623,TOP1MT,DGAT1,HSF1,MYBL1,ZNF696,TCEA1,ZNF250,ZNF34,ZNF517,GPAA1,ZNF251,ASPH	4.92E-02
10144	140	99	GO:0044237	BP	Cellular metabolic process	ST3GAL1,ZC3H3,SNAI2,RB1CC1,RNF19A,NSMAF,TAF2,ARFGEF1,MTFR1,TRAM1,PABPC1,CPSF1,MYST3,CPNE3,NBN,DECR1,UBE2W,POP1,STK3,EIF3E,UBR5,TSTA3,PYCRL,SQLE,MCM4,ENY2,LYPLA1,COPS5,RDH10,THAP1,HRSP12,IMPA1,E2F5,DERL1,MRPL15,NCOA2,GOLGA7,MRPS28,TERF1,RPL7,MTDH,POLR2K,EIF3H,UTP23,NDUFB9,TATDN1,ZNF7,TCEB1,AZIN1,PTDSS1,RPL30,WDYHV1,NSMCE2,AGPAT6,VPS28,RPL8,PEX2,RAD21,MED30,COX6C,YWHAZ,TRAPPC9,FNTA,UBE2V2,PTK2,COMMD5,ZNF16,CHD7,MRPL13,ADCK5,VCPIP1,OPLAH,EXOSC4,RRS1,CYC1,SHARPIN,MAF1,PUF60,SCRIB,MAPK15,ZNF707,FBXL6,ZNF623,TRMT12,TOP1MT,PTP4A3,DGAT1,HSF1,NRBP2,MYBL1,ZNF696,TCEA1,ZNF250,ZNF34,ZNF517,GPAA1,ZNF251,ASPH,BOP1	2.88E-02
6763	140	75	GO:0006807	BP	Nitrogen compound metabolic process	ST3GAL1,ZC3H3,SNAI2,RB1CC1,NSMAF,TAF2,TRAM1,PABPC1,CPSF1,MYST3,NBN,UBE2W,POP1,STK3,EIF3E,UBR5,TSTA3,PYCRL,MCM4,ENY2,LYPLA1,COPS5,THAP1,HRSP12,E2F5,MRPL15,NCOA2,MRPS28,TERF1,RPL7,MTDH,POLR2K,EIF3H,UTP23,NDUFB9,TATDN1,ZNF7,TCEB1,AZIN1,PTDSS1,RPL30,NSMCE2,AGPAT6,RPL8,PEX2,RAD21,MED30,COX6C,YWHAZ,TRAPPC9,UBE2V2,COMMD5,ZNF16,CHD7,MRPL13,OPLAH,EXOSC4,RRS1,MAF1,PUF60,MAPK15,ZNF707,ZNF623,TRMT12,TOP1MT,HSF1,MYBL1,ZNF696,TCEA1,ZNF250,ZNF34,ZNF517,ZNF251,ASPH,BOP1	1.60E-02
3966	140	52	GO:0003676	MF	Nucleic acid binding	ZC3H3,SNAI2,TAF2,PABPC1,CPSF1,MYST3,CPNE3,NBN,POP1,EIF3E,UBR5,MCM4,COPS5,THAP1,HRSP12,E2F5,MRPL15,NCOA2,MRPS28,TERF1,RPL7,MTDH,POLR2K,EIF3H,UTP23,ZNF7,RPL30,RPL8,YWHAZ,ZNF16,CHD7,MRPL13,PLEC,EXOSC4,RRS1,MAF1,TIGD5,PUF60,ZNF707,ZFP41,ZNF623,RBM12B,TOP1MT,HSF1,MYBL1,ZNF696,TCEA1,ZNF250,ZNF34,ZNF517,ZNF251,BOP1	1.23E-02
5013	140	60	GO:0090304	BP	Nucleic acid metabolic process	ZC3H3,SNAI2,RB1CC1,TAF2,PABPC1,CPSF1,MYST3,NBN,UBE2W,POP1,STK3,EIF3E,UBR5,MCM4,ENY2,COPS5,THAP1,HRSP12,E2F5,NCOA2,TERF1,RPL7,MTDH,POLR2K,UTP23,TATDN1,ZNF7,TCEB1,RPL30,NSMCE2,RPL8,PEX2,RAD21,MED30,COX6C,YWHAZ,TRAPPC9,UBE2V2,COMMD5,ZNF16,CHD7,EXOSC4,RRS1,MAF1,PUF60,MAPK15,ZNF707,ZNF623,TRMT12,TOP1MT,HSF1,MYBL1,ZNF696,TCEA1,ZNF250,ZNF34,ZNF517,ZNF251,ASPH,BOP1	2.98E-02
