# Supplementary material for: Genomic and transcriptomic heterogeneity in metaplastic carcinomas of the breast
Source: NPJ Breast Cancer. 2017 Dec 1;3:48. doi: 10.1038/s41523-017-0048-0 (PMC5711926; doi:10.1038/s41523-017-0048-0)
Supplement: Supplementary file 31 — Supplementary Table 19 [file 41523_2017_48_MOESM31_ESM.pdf]

Supplementary Table 19: Fusion transcripts identified by deFuse and/ or ChimeraScan.

Sample ID	Gene 5'	Gene 3'	Breakpoints	Split reads	Total reads	Frame	Fusion calling algorithm	Validated
META55	MAP2K3	HMGCLL1	chr17:21208440>chr6:55381401	10	16	In-frame	deFuse & ChimeraScan	Y
META39	WAPAL	CDHR1	chr10:88205999>chr10:85965583	33	53	In-frame	deFuse & ChimeraScan	Y
META64	PSMA6	SHMT1	chr14:35780101>chr17:18236602	48	58	In-frame	deFuse & ChimeraScan	Y
META39	AAK1	ARNT2	chr2:69870010>chr15:80743221	3	13	In-frame	deFuse	Y
META37	TNKS1BP1	SPARC	chr11:57087553>chr5:151055762	5	16	In-frame	deFuse	Y
META37	PARG	BMS1	chr10:51093249>chr10:43287075	8	25	In-frame	deFuse	Y
META52	MBTPS1	TCEANC2	chr16:84108202>chr1:54561958	19	48	In-frame	deFuse	Y
META47	FN1	ICAM1	chr2:216270961>chr19:10394157	33	58	In-frame	deFuse	Y
META52	TBL1XR1	PIK3CA	chr3:176738542>chr3:178866311	2	4	In-frame	ChimeraScan	Y
META41	MFGE8	HAPLN3	chr15:89444802>chr15:89430576	2	7	In-frame	deFuse	
META64	VCL	SEC24C	chr10:75860855>chr10:75528568	2	7	In-frame	deFuse	
META52	WWOX	CCDC101	chr16:78466649>chr16:28592376	2	9	In-frame	deFuse	
META53	HSF4	NOL3	chr16:67203004>chr16:67208065	4	9	Out of frame	deFuse	
META30	LSM14A	WTIP	chr19:34663668>chr19:34981281	2	9	In-frame	deFuse	
META59	COL3A1	COL1A1	chr2:189873947>chr17:48263868	5	10	In-frame	deFuse	
META64	IVD	PAK6	chr15:40707681>chr15:40556982	4	10	In-frame	deFuse	
META53	VPS45	PLEKHO1	chr1:150082742>chr1:150123102	2	10	Out of frame	deFuse	
META32	DDX5	POLG2	chr17:62496667>chr17:62489138	5	10	In-frame	deFuse	
META49	UNC5B	SLC29A3	chr10:73058992>chr10:73079067	5	10	In-frame	deFuse	
META59	PGS1	AFMID	chr17:76400170>chr17:76198580	6	11	In-frame	deFuse	
META42	PLAUR	CADM4	chr19:44156377>chr19:44131942	5	12	In-frame	deFuse	
META42	C15orf57	CBX3	chr15:40854180>chr7:26241365	3	12	Out of frame	deFuse	
META52	MORN4	MCMBP	chr10:99393104>chr10:121609088	2	12	Out of frame	deFuse	
META37	MX2	MX1	chr21:42762630>chr21:42811621	6	13	In-frame	deFuse	
META30	TARBP1	F5	chr1:234556448>chr1:169515830	4	15	In-frame	deFuse	
META42	FARSA	SYCE2	chr19:13034965>chr19:13015480	5	15	In-frame	deFuse	
META52	ANKS3	ZNF500	chr16:4755096>chr16:4816077	4	15	Out of frame	deFuse	
META32	MXRA8	DVL1	chr1:1289228>chr1:1278138	7	16	Out of frame	deFuse	
META52	ANKS3	SEPT12	chr16:4755096>chr16:4828138	11	16	In-frame	deFuse	
META42	XPO4	N6AMT2	chr13:21395843>chr13:21311944	6	17	In-frame	deFuse	
META52	NUP133	ABCB10	chr1:229625702>chr1:229654622	5	21	In-frame	deFuse	
META31	MFGE8	HAPLN3	chr15:89444782>chr15:89430576	8	21	In-frame	deFuse	
META42	CRIP2	CRIP1	chr14:105945972>chr14:105953636	9	27	Out of frame	deFuse	
META30	MOGS	CCDC142	chr2:74691835>chr2:74702180	18	30	Out of frame	deFuse	
META42	DMKN	KRTDAP	chr19:35989618>chr19:35979742	12	35	In-frame	deFuse	

META41	RASSF1	TUSC2	chr3:50374604>chr3:50363908	7	41	Out of frame	deFuse	
META53	CHCHD10	VPREB3	chr22:24108315>chr22:24095385	17	47	In-frame	deFuse	
META62	MPHOSPH8	CD163L1	chr13:20221431>chr12:7586290	2	6	Out of frame	ChimeraScan	
META52	DCHS2	KDM1B	chr4:155410456>chr6:18222140	2	7	In-frame	ChimeraScan	
META49	TMEM236	MRC1	chr10:17818169>chr10:18112044	2	5	In-frame	ChimeraScan	
META42	PBRM1	NPAS1	chr3:52668618>chr19:47535536	8	27	Out of frame	ChimeraScan	
META39	SLC20A2	SCN10A	chr8:42358549>chr3:38770392	5	25	Out of frame	ChimeraScan	
META52	PDXP	VAT1L	chr22:38055363>chr16:77910267	14	31	Out of frame	ChimeraScan	
