# Supplementary material for: Genomic and transcriptomic heterogeneity in metaplastic carcinomas of the breast
Source: NPJ Breast Cancer. 2017 Dec 1;3:48. doi: 10.1038/s41523-017-0048-0 (PMC5711926; doi:10.1038/s41523-017-0048-0)
Supplement: Supplementary file 32 — Supplementary Table 20 [file 41523_2017_48_MOESM32_ESM.pdf]

Supplementary Table 20: List of non-synonymous mutations in cancer genes identified from RNA-sequencing analysis of 17 metaplastic carcinomas of the breast.

Sample	Gene	Mutation	Effect	Hotspot	Chromosome	Position	Reference allele	Alternate allele	CHASM	Mutation Taster	COSMIC	cancer_gene_census	kandoth	lawrence
META30T	MXRA5	p.N2814fs	frameshift_variant	.	X	3227802	G	GT			.	.	.	TRUE
META30T	HSP90AA1	p.V705fs	frameshift_variant	.	14	102549379	C	CT			.	TRUE	.	.
META31T	TSC2	p.A306T	missense_variant	.	16	2108815	G	A	Driver	Deleterious	.	TRUE	.	.
META31T	KTN1	p.K270N	missense_variant	.	14	56084830	A	C	Driver	Deleterious	.	TRUE	.	.
META31T	TET2	p.G1861R	missense_variant	.	4	106197248	G	A	Driver	Deleterious	.	TRUE	TRUE	TRUE
META31T	TCF7L2	p.R465K	missense_variant&splice_region_variant	.	10	114925316	G	A		Deleterious	.	TRUE	.	TRUE
META32T	TP53	c.673-1G>T	splice_acceptor_variant&intron_variant	HOTSPOT	17	7577609	C	A			TRUE	TRUE	TRUE	TRUE
META32T	TP53	p.W91*	stop_gained	.	17	7579414	C	T			TRUE	TRUE	TRUE	TRUE
META36T	ZNF471	p.K293E	missense_variant	.	19	57036313	A	G		Deleterious	.	.	.	TRUE
META36T	SEPT7	p.P195S	missense_variant	.	17	75398647	C	T		Deleterious	.	TRUE	.	.
META37T	TP53	p.R273C	missense_variant	HOTSPOT	17	7577121	G	A	Driver	Deleterious	TRUE	TRUE	TRUE	TRUE
META37T	KIF5B	p.V664I	missense_variant	.	10	32311110	C	T		Deleterious	.	TRUE	.	.
META37T	JAK1	p.L346P	missense_variant	.	1	65330609	A	G		Deleterious	.	TRUE	.	.
META39T	TP53	p.C275R	missense_variant	HOTSPOT	17	7577115	A	G	Driver	Deleterious	TRUE	TRUE	TRUE	TRUE
META40T	TP53	p.N239S	missense_variant	HOTSPOT	17	7577565	T	C	Driver	Deleterious	TRUE	TRUE	TRUE	TRUE
META40T	SMARCA4	p.Y1118F	missense_variant	.	19	11138597	A	T		Deleterious	.	TRUE	.	TRUE
META40T	NTN4	p.Y185fs	frameshift_variant	.	12	96180747	GT	G			.	.	.	TRUE
META40T	GNPTAB	p.E632Q	missense_variant	.	12	102158801	C	G		Deleterious	.	.	.	TRUE
META40T	MLLT4	p.L1701V	missense_variant	.	6	168366719	C	G		Deleterious	.	TRUE	.	.
META41T	TP53	p.P278L	missense_variant	HOTSPOT	17	7577105	G	A	Driver	Deleterious	TRUE	TRUE	TRUE	TRUE
META41T	SMAD4	p.Asp52fs	frameshift_variant	.	18	48573563	G	GA			.	TRUE	TRUE	TRUE
META42T	TP53	p.C176F	missense_variant	HOTSPOT	17	7578403	C	A	Driver	Deleterious	TRUE	TRUE	TRUE	TRUE
META42T	ARHGAP35	c.3826+2T>C	splice_donor_variant&intron_variant	.	19	47440667	T	C			.	.	TRUE	TRUE
META42T	NIN	p.G1117fs	frameshift_variant	.	14	51224399	C	CA			.	TRUE	.	.
META42T	HERPUD1	p.T163S	missense_variant	.	16	56973204	A	T		Deleterious	.	TRUE	.	.
META42T	PAFAH1B2	p.K133R	missense_variant	.	11	117034595	A	G		Deleterious	.	TRUE	.	.
META42T	KMT2A	p.L3462F	missense_variant	.	11	118376991	C	T	Driver	Deleterious	TRUE	TRUE	.	TRUE
META47T	TCF12	p.S295P	missense_variant	.	15	57524967	T	C	Driver	Deleterious	.	TRUE	.	.
META47T	DDX5	p.A592T	missense_variant	.	17	62496112	C	T		Deleterious	.	TRUE	.	TRUE
META47T	HIP1	p.M188I	missense_variant	.	7	75210593	C	G		Deleterious	.	TRUE	.	.
META47T	CUX1	p.N33K	missense_variant	.	7	101559430	C	A	Driver	Deleterious	.	TRUE	.	TRUE
META47T	PIK3CA	p.His1047R	missense_variant	HOTSPOT	3	178952085	A	G	Driver	Deleterious	TRUE	TRUE	TRUE	TRUE
META49T	CBFB	c.495+2T>C	splice_donor_variant&intron_variant	.	16	67116213	T	C			.	TRUE	TRUE	TRUE
META52T	EIF3E	p.V404E	missense_variant	.	8	109215300	A	T		Deleterious	.	TRUE	.	.
META52T	IDH1	p.V125G	missense_variant	.	2	209113133	A	C		Deleterious	.	TRUE	TRUE	TRUE
META53T	PDE4DIP	p.E1024*	stop_gained	.	1	144886164	C	A			.	TRUE	.	.
META59T	TSC2	p.N1182S	missense_variant	.	16	2130313	A	G	Driver		.	TRUE	.	.
META59T	TP53	p.S127F	missense_variant	.	17	7578550	G	A		Deleterious	TRUE	TRUE	TRUE	TRUE
META59T	SLC1A3	p.A243S	missense_variant	.	5	36677153	G	T		Deleterious	.	.	.	TRUE
META59T	HSP90AB1	p.T599P	missense_variant	.	6	44220845	A	C		Deleterious	.	TRUE	.	.
META59T	MSH6	p.R1321T	missense_variant	.	2	48033751	G	C		Deleterious	.	TRUE	.	.
META62T	TP53	p.C124fs	frameshift_variant	.	17	7579315	G	GC			TRUE	TRUE	TRUE	TRUE
META62T	DDX5	p.R359T	missense_variant	.	17	62498575	C	G	Driver	Deleterious	.	TRUE	.	TRUE
META64T	TP53	p.R249G	missense_variant	HOTSPOT	17	7577536	T	C	Driver	Deleterious	TRUE	TRUE	TRUE	TRUE
META64T	HIST1H2BD	p.S125fs	frameshift_variant&stop_gained	.	6	26158768	G	GCTCCAAGTAAACA			.	.	TRUE	.
META64T	POLE	c.6137-2A>C	splice_acceptor_variant&intron_variant	.	12	133209096	T	G			.	.	.	TRUE
META64T	PIK3CA	p.His1047R	missense_variant	HOTSPOT	3	178952085	A	G	Driver	Deleterious	TRUE	TRUE	TRUE	TRUE
