# Supplementary material for: Genomic and transcriptomic heterogeneity in metaplastic carcinomas of the breast
Source: NPJ Breast Cancer. 2017 Dec 1;3:48. doi: 10.1038/s41523-017-0048-0 (PMC5711926; doi:10.1038/s41523-017-0048-0)
Supplement: Supplementary file 33 — Supplementary Table 21 [file 41523_2017_48_MOESM33_ESM.pdf]

Supplementary Table 21: Summary of antibodies, clones, dilutions and antigen retrieval methods.

Marker	Antibody clone	Dilution	Antigen retrieval	Company
**CK17**	E3	1/100	18 min, MW,citrate, pH 6.0	DAKO, Glostrup, Denmark
**PTEN**	6H2.1	1/100	20 mins, 98°C, DAKO antibody retrieval solution, pH 9.0	DAKO, Glostrup, Denmark
**ER**	ID5	1/200	2 min, PC, citrate, pH 6.0	DAKO, Glostrup, Denmark
**PR**	PgR 636	1/200	3 min, PC, citrate, pH 6.0	DAKO, Glostrup, Denmark
**HER2**	Polyclonal	1/200	41 min water bath, DAKO antibody retrieval solution, pH 6.0	DAKO, Glostrup, Denmark
**P53**	DO7	1/200	2 min, PC, citrate, pH 6.0	DAKO, Glostrup, Denmark
**P63**	P634A4	1/200	18 min, MW, citrate, pH 6.0	Santa Cruz, Santa Cruz, CA, USA
**CK14**	LL002	1/40	18 min, MW, citrate pH 6.0	BioGenex, Fremont, USA
**EGFR**	31G7	1/50	10 mins, 37°C, 0.1% pronase	Zymed, San Francisco, CA, USA
**C-KIT**	CD117	1/50	No antigen retrieval required	DAKO, Glostrup, Denmark
**CK5/6**	D5/16B4	1/600	18 min, MW, pH 6.0	Chemicon, Temecula, CA,USA

ER: oestrogen receptor, PR: progesterone receptor, EGFR: epidermal growth factor receptor, CK: Cytokeratin, PC: pressure cooker, MW: microwave oven.
