# Supplementary material for: Genomic and transcriptomic heterogeneity in metaplastic carcinomas of the breast
Source: NPJ Breast Cancer. 2017 Dec 1;3:48. doi: 10.1038/s41523-017-0048-0 (PMC5711926; doi:10.1038/s41523-017-0048-0)
Supplement: Supplementary file 34 — Supplementary Table 22 [file 41523_2017_48_MOESM34_ESM.pdf]

Supplementary Table 22: Oligonucleotide primers used for the validation of fusion transcripts by reverse transcription PCR (RT-PCR).

Fusion Gene	Strand	Sequence
AAK1-ARNT2	Forward	TCACAGTGGACGAGGTGTTG
	Reverse	AATTTACTGGGGCCTTCACC
FN1-ICAM1	Forward	CCATCAGCAGGAACACCTTT
	Reverse	GGGTAAGGTTCTTGCCCACT
MAP2K3-HMGCLL1	Forward	TGAGAGGATCAACCCAGAGC
	Reverse	TCTCAGTAGCTCCAGCAGCA
MBTPS1-TCEANC2	Forward	GAGGAATGCCGACAGTTGTT
	Reverse	CAGCTCGGTGCTTTAATGTG
PARG-BMS1	Forward	CTCTCTCTCTGTGTGACCCTG
	Reverse	ATAACTGTAATAAAACGGCCCAG
PSMA6-SHMT1	Forward	GAAATGAGGCCTCTTGGTTG
	Reverse	ACCATCTGTGCCTTTGGAAC
TNKS1BP1-SPARC	Forward	GGAGGGGCCAGTAAAGTCTC
	Reverse	CCAGGCAAAGGAGAAAGAAG
WAPAL-CDHR1	Forward	ATCAAAGATGCTCCCACCAC
	Reverse	ATGGAGATGGCTCCAGATGT
TBL1XR1-PIK3CA	Forward	TGGTGGAGGCTCTTTGAAGT
	Reverse	TTCACCTGATGATGGTCGTG
